# MiRNA Profiling in Pectoral Muscle Throughout Pre- to Post-Natal Stages of Chicken Development

**DOI:** 10.3389/fgene.2020.00570

**Published:** 2020-06-23

**Authors:** Min Chen, Shaolan Zhang, Zhongxian Xu, Jian Gao, Shailendra Kumar Mishra, Qing Zhu, Xiaoling Zhao, Yan Wang, Huadong Yin, Xiaolan Fan, Bo Zeng, Mingyao Yang, Deying Yang, Qingyong Ni, Yan Li, Mingwang Zhang, Diyan Li

**Affiliations:** ^1^Farm Animal Genetic Resources Exploration and Innovation Key Laboratory of Sichuan Province, Sichuan Agricltural University, Wenjiang, China; ^2^Department of Science and Technology, Chengdu Medical College, Chengdu, China; ^3^Institute of Biopharming, West China Hospital, Sichuan University, Chengdu, China

**Keywords:** chicken, miRNA, myoblast, differentiation, gga-miRNA-454

## Abstract

MicroRNA (miRNA) is known to be an important regulator of muscle growth and development. The regulation of microRNA on the skeletal muscle phenotype of animals is mainly achieved by regulating the proliferation and differentiation of myoblasts. In this study, we sequenced a total of 60 samples from 15 developing stages of the pectoral muscle and five other tissues at 300 days of Tibetan chicken. We characterized the expression patterns of miRNAs across muscle developmental stages, and found that the chicken growth and development stage was divided into early-embryonic and late-embryonic as well as postnatal stages. We identified 81 and 21 DE-miRNAs by comparing the miRNA profiles of pectoral muscle of three broad periods and different tissues, respectively; and 271 miRNAs showed time-course patterns. Their potential targets were predicted and used for functional enrichment to understand their regulatory functions. Significantly, GgmiRNA-454 is a time-dependent and tissue-differential expression miRNA. In order to elucidate the role of gga-miRNA-454 in the differentiation of myoblasts, we cultured chicken myoblasts in vitro. The results show that although gga-miRNA-454-3p initiates increase and thereafter decrease during the chicken myoblasts differentiation, it had no effect on primary myoblasts proliferation. Furthermore, we confirm that gga-miRNA-454 inhibits myoblast differentiation by targeting the myotube-associated protein SBF2.

## Introduction

Skeletal muscle is a type of striated muscle tissue responsible for all voluntary movement in animals. It accounts for half of the total body weight of the chicken. It is an important organization involved in regulation of animal metabolism, and strength (Pavlath and Horsley, 2003). The growth and development of skeletal muscle have an essential influence on meat production performance of poultry (Anne et al., 2016). Previous research on Tibetan chicken muscles involved physical characteristics and processing properties (Qiu et al., 2011). Some assessed the adaptation of lowland chickens to highland from several aspects of liquid characteristics, blood gas and blood volume (León and Monge, 2004). Previous reports on Tibetan chickens have focused mainly on the physiological, biochemical and molecular mechanisms related to the adaptation of high-altitude environments (Monge and Leónvelarde, 1991; Xiao et al., 2005; Weber, 2007). However, there are few studies on the growth and development of Tibetan chickens, especially the regulation of miRNA on muscle growth and development of Tibetan chicken.

Skeletal muscle development is a complicated biological process controlled by various regulatory factors and signaling pathways. The muscles in avian development are composed of multiple myogenic groups (Cossu and Molinaro, 1987; Stockdale and Jeffrey, 1987). Myoblasts from embryonic and adult chicken development exhibit intrinsically distinct classes of myogenic populations. Embryonic myoblasts are most abundant on day 5, whereas fetal myoblasts are most abundant between days 8 and 12 (Stockdale, 1993). In chicken, during early embryonic and late fetal development, several myoblasts fuse to form myotubes containing multiple muscle colonies with various types of fast and slow myosin heavy chain (Bentzinger et al., 2012). Moreover, it has been reported that the initial stage of myogenesis in poultry is 3–7 days in the embryonic stage, and the subsequent fetal stage or the second stage is 8–12 days in the embryonic stage (Cossu and Molinaro, 1987). The proliferation of chicken embryonic myoblasts and the process of their differentiation into myotubes largely determines the number of muscle fibers after birth (Brown, 1987; Goldspink and Ward, 1979). MiRNAs are involved in various aspects of skeletal muscle development by targeting transcription factors at different stages (Güller and Aaron, 2010). Due to the tissue specificity of miRNA expression, several muscle-specific miRNAs (“myomiRs”), such as miRNA-1, miRNA-133, miRNA206 and miRNA-499 have been identified that control signaling pathways mediating skeletal myogenesis (Horak et al., 2016). MiRNAs are considered as an integral part of muscle formation regulatory network, and some miRNAs are colinearly expressed in time and space during body development (Wienholds and Ronald, 2005). Of note, some miRNAs are expressed in diverse tissues of animals and belong to non-specific miRNAs. However, the expressions of miR-23–27–24 clusters have limited effect on muscle growth and development in animals (Lee et al., 2019).

The study of chicken miRNA regulation mainly involves development in many aspects, including embryos, bones, gonads, and neuro development, the rest are in terms of immune function as well as viral infection and treatment (Hornstein et al., 2005; Dahlberg and Lund, 2007; Rodriguez et al., 2007; Hicks et al., 2008; McGlinn et al., 2009; Zhao et al., 2009; Bannister et al., 2011; Burnside and Morgan, 2011; Song et al., 2013). Studies of miRNAs in chicken embryos have shown that fibroblast growth factor (FGF)-mediated signaling negatively regulates the initiation of miR-206 gene expression, demonstrating for the first time that developmental signaling pathways impact miRNA expression (Sweetman and Tina, 2006). In addition, by comparing the miRNA expression profiles of myoblast in the proliferative stage, it was found that miR-221 and miR-222 were significantly down-regulated during chicken myoblast differentiation (Cardinali et al., 2009). Expression of the cell cycle inhibitor protein gene (p27) regulates differentiation and maturation of skeletal muscle cells (Cardinali et al., 2009). Recently study showed that under hypoxia conditions, specific microRNA (miRNA) regulates lung development, and hypoxia induces the elevation of miR-15a, thereby inhibiting the expression of Bcl-2 protein in Tibetan chicken (Du et al., 2017).

This study constructed 13 miRNA libraries of pectoral muscles and 6 different tissues at different growth stages, screened time-dependent and tissue-differential expression miRNAs, and further analyzed possible target genes and related regulatory pathways of these miRNAs to enrich chicken miRNA The information reveals the spatiotemporal specific expression characteristics of miRNAs in muscles, and lays the foundation for a deep understanding of their role in regulating muscle growth and development.

## Materials and Methods

### Ethics Statement

All experimental protocols were subject to the Institutional Animal Care and Use Committee in the College of Animal Science and Technology, Sichuan Agricultural University, China.

### Sample Collection and RNA Extraction

The muscle tissue used in embryonic period of this experiment was collected by incubation of the eggs in time, and all the fertile eggs were bought from Jiuding yuan Ecological Livestock and Poultry Breeding Co., Ltd. located in Mao County, Aba Tibetan and Qiang Autonomous Prefecture, Sichuan Province. The entire embryo organization was collected on the 5th day (E5) and 7th day (E7) of the embryonic stage. We collected pectoral muscle tissue from six experimental periods at the embryonic stage of 9th day (E9), 12th day (E12), 15th day (E15), 18th day (E18), 20th day (E20) and the first day after hatching (D1). Further, we collected pectoral muscle tissue from seven periods post-hatching, including the 36th day (D36), 100th day (D100), 300th day (D300), 2nd year (2Y), 5th year (5Y), 8th year (8Y) and 12th year (12Y), and six tissues originated from different germ layers at the age of 300th day including brain, liver, ovary, spleen, kidney, pectoral muscle. All samples were collected in triplicates. To collect various tissues, birds were slaughtered after giving anesthesia, and samples were wrapped in aluminum foil, flash frozen in liquid nitrogen and transported to laboratory, then stored at −80°C until RNA extraction. Total RNA was extracted from individual sample using RNAiso Plus reagent (TaKaRa) following manufacturer’s recommendations. RNA was quantified by Nanodrop ND-2000 spectrophotometer (Thermo Fisher Scientific, United States); and RNA purity was evaluated by agarose gel electrophoresis. All RNA samples were stored at −80°C for subsequent study.

### Small RNA Sequencing and Data Analyses

Both Small RNA library construction and sequencing were performed Illumina (Solexa) platform (ANNOROAD, Beijing, China). The raw reads stored in FASTQ-formatted files were subjected to quality filtering using fast-tool kit software to remove low quality reads (>20% bases with a mass value <30), thereby the high-quality reads were obtained. Cutadapt software (Martin, 2011) was used to further remove the sequencing adapters and fragments <18 nt and >30 nt in length. Subsequently, the remaining 18∼30 nt clean reads were aligned to Repbase database^1^ to exclude transfer RNA (tRNA), ribosomal RNA (rRNA), small nuclear RNA (snRNA), small nucleolar RNA (snoRNA) using bowtie2 software (Langmead, 2010) with perfect matches. The high quality reads were mapped to Gallus gallus genome to identify known mature miRNAs or pre-miRNAs using miRBase database v.21^2^. The unannotated sequences were mapped to reference genome of zebra finch (Taeniopygia guttata) is closely related species with chicken in order to predict novel miRNAs using mapper.pl script from mirDeep2 (Friedlander et al., 2008). We take miDeep2 scores ≥5 and the secondary structure p value as yes as candidate miRNAs. A candidate novel miRNA predicted by at least two samples was considered as a novel miRNA.

### Analysis of miRNA Expression Profiles

Hierarchical clustering analysis was performed based on a distance matrix of the Pearson correlation of the samples. Principal component analysis finds low-dimensional linear combinations of data with maximal variability. To identify miRNAs which were differentially expressed across development, we separated 13 time points to three periods based on the HCL results and used *t*-test to detect differentially expressed miRNAs (DE-miRNA) between the three periods and six tissues of D300, respectively, genes with *P*-value ≤ 0.05, fold change (FC) ≥2 or ≤0.5 were denoted as DE-miRNAs (Ying et al., 2014). The R package, maSigPro (Nueda et al., 2014) was used for time course analysis of expression data. A cut-off value for the R-square of the regression model was taken as 0.6.

### Functional Analysis of Target Genes

Prediction of DE-miRNAs targets was performed by the intersection of miRDB^3^ and TargetScan^4^. Next, Gene Ontology (GO) and the Kyoto Encyclopedia of Genes and Genomes (KEGG) pathway enrichment analysis for functional annotation of target genes were performed using Metascape^5^ (Zhou et al., 2019). All genes in the genome were used as enrichment background with *p*-value < 0.01, a minimum count of three, and an enrichment factor >1.5.

### Quantitative Real-Time PCR Analysis

We perform Real-time quantitative PCR analysis for randomly selected 5 known differentially expressed miRNAs and 3 novel miRNAs. First-strand cDNA was synthesized using miRNA first-strand cDNA synthesis kit (Aidlab Biotechnology Co. Ltd., Beijing, ChinaqRT-PCR was carried out using the TransStart^®^ Top Green qPCR SuperMix (TransGen Biotech, Beijing, China). The qRT-PCR was performed using mRNA-specific primers and a universal miRNA reverse primer 5′-TCTAGAGGCCGAGGCGGCCGACATGT-3′. The primer sequences are listed in the Table 1. U6 gene and β-actin gene were used as endogenous internal controls for normalization. We collected cells before transfection as a control group. The cells in this control group were not treated with mimic, inhibitor, mimic NC, inhibitor NC. When studying the expression of gga-miR-454 during the growth of primary myoblasts, we counted the gga-miR-454 expression collected at 24 h as 1, and compared it with other time points to calculate the difference. The 2^–ΔΔCT^ method was used to determine the relative miRNA and mRNA abundance (Livak and Schmittgen, 2001).

**TABLE 1:** The primer information of Q-PCR.

Gene	Primer Sequence	Tm/°C	Product size/bp
*Myhc*	F: CTCCTCACGCTTTGGTAA	58	213
	R: TGATAGTCGTATGGGTTGGT		
*Myog*	F: GGAGGCTGAAGAAGGTGA	59	149
	R: CTGGTTGAGGCTGCTGA		
*SBF2*	F: AAATCCCTCCCAACAAAG	57	78
	R: GCCAAAAGGTCACTAACG		
*β-actin*	F: GCCAACAGAGAGAAGATGACAC	60	140
	R: GTAACACCATCACCAGAGTCCA		
gga-miR-454-3p	F: CAGUGCAAUAGUAUUGUCAAAGCAU	60	/
	R: ATTCTAGAGGCCGAGGCGGCCGACATGT		
gga-miR-301b-3p	F: GCGGCAGTGCAATAGTATTGTCAAAGCAT	60	/
	R: ATTCTAGAGGCCGAGGCGGCCGACATGT		
gga-let-7a-2-3p	F: CTGTACAACCTCCTAGCTTTCC		
	R: ATTCTAGAGGCCGAGGCGGCCGACATGT		
gga-miR-101-3p	F: GGGTACAGTACTGTGATAACTGA		
	R: ATTCTAGAGGCCGAGGCGGCCGACATGT		
gga- miR-133c-3p	F: TTGGTCCCCTTCAACCAGCT		
	R: ATTCTAGAGGCCGAGGCGGCCGACATGT		
gga-miR-204	F: TTCCCTTTGTCATCCTATGCCT		
	R: ATTCTAGAGGCCGAGGCGGCCGACATGT		
gga-miR-144-3p	F: GGCTACAGTATAGATGATGTACTC		
	R: ATTCTAGAGGCCGAGGCGGCCGACATGT		
new-miRNA-11	F: TCCAGCATCAGTGATTTTGTTG		
	R: ATTCTAGAGGCCGAGGCGGCCGACATGT		
new-miRNA-15	F: AAGCCCTTACCCCAAAAAGCAT		
	R: ATTCTAGAGGCCGAGGCGGCCGACATGT		
U6	F: GGAACGATACAGAGAAGATTAGC	58	Du et al., 2017
	R: TGGAACGCTTCACGAATTTGCG		

### Preparation of Chicken Embryo Extract

The entire embryo was collected by removing the head and internal organs and washed with DMEM and minced into small fragments with sterilized scissors. The fragments were mixed with DMEM/F12 medium in a 1:1 volume with a 50 mL syringe repeatedly. Repeated freeze-thaw three times in −80°C. or liquid nitrogen (Kita et al., 1998). After centrifugation at 10,000 *g* for 10 min, supernatant was carefully collected into a 15 mL centrifuge tube and stored frozen at −20°C. Aggregates were removed by centrifugation at 700 *g* for 10 min prior to use of the reagents, followed by the addition of 5% complete medium supernatant.

### Isolation and Culture of Primary Myoblasts

Embryonated chicken eggs (Arbor Acres) were purchased from Large Poultry Breeding professional cooperative in Xinjin County, Chengdu, Sichuan province, China. We refer to the methods reported in the previous literature (Gerstenfeld et al., 1984; Yablonka and Nameroff, 1987; Hartley et al., 1992). Eggs were maintained in incubator at 37.5°C with a relative humidity of 60% for 10 days. Myoblasts were isolated from pectoralis muscle by employing enzyme digestion and Percoll density centrifugation. Briefly, the pectoral muscle tissue from 10 embryos was collected under aseptic conditions and washed with phosphate-buffered saline (PBS) containing penicillin (100 units/mL) and streptomycin (100 μg/mL). Then muscle tissues were minced with sterilized sharp scissors into small fragments of about 1mm3. The fragments were digested with 0.1% concentration of the type I collagen enzyme (Solarbio C8140) of two volumes of meat for 30 min at 37°C, and the supernatant was discarded after centrifugation at 300*g* for 5 min. Digestion was repeated with 0.25% trypsin (Gibco) of three volume of remaining muscle tissue for 20 min at 37°C. Then, the muscle lysate was sieved sequentially filtered with a 200 and 400 mesh stainless steel strainer to remove large debris and the myoblasts were collected by centrifugation of 2000 rpm for 10 min. A cell population that contained skeletal myogenic precursor cells was recovered from 20/60% percoll interface (Yablonka et al., 1988). The isolated cells were seeded in growth medium (GM) containing DMEM/F12, 5% chicken embryo extract and 15% fetal bovine serum (FBS). To induce myoblasts differentiation, cells were seeded in differentiation medium (DM) containing 2% horse serum in DMEM.

### Cell Staining

The concentration of myoblast cells was adjusted to about 4 × 104 cells/well and then inoculated into the 24-well plates (5% CO2 incubator at 37°C), which was then induced to differentiate after 24 h. It is fixed and dead at the time point of 24, 48, and 72 h of the paving slab, respectively. Specifically, the cells were first washed with preheated PBS for 5 min each, then fixed in methanol for dehydration (5 min), and then dried on the ultra-clean table for 10 min. Cells were then incubated with May-Grünwald dilution solution (1:3 in the sodium phosphate buffer) for 5 min and washed twice with distilled water. Finally, cells were stained with Giemsa dilution solution (1:5 in distilled water) for 20 min and then rinsed thrice with distilled water. The cells visualized under an optical microscope and microscopic images were captured with 100× magnifications.

### Chicken Myoblasts Transfection

When chicken myoblasts reached approximately 80% confluence, cells were treated with micrON^TM^ miRNA mimic (100 nM) and micrOFF^TM^ miRNA inhibitor (200 nM) supplied by Ruibo Biotechnology Co., Ltd., Guangzhou. Negative controls for mimics and inhibitors were provided by Ruibo Biotechnology Co., Ltd., Guangzhou, using 100 nM and 200 nM transfection concentrations, respectively. In this in vitro transfection cell experiment, we used used miRNA mimic NC (100 nM) and miRNA inhibitor NC (200 nM) as negative controls, respectively. In addition, the recommended range is 10∼200 nM by Ruibo Biological, usually a larger number of miRNA inhibitors are needed to observe a better inhibitory effect, which is equivalent to several times the miRNA mimic amount, which may be the mechanism of competitive inhibition with miRNA inhibitors and efficiency. The cells were transfected by using Lipofectamine 3000 reagent (Invitrogen, United States), according to manufacturer’s direction.

### Immunofluorescence Analysis

The cells were fixed in 4% paraformaldehyde for 15 min at room temperature in a 24-well plate. Permeable cells were treated with 1% Trion X-100 for 10 min at 4°C. The cells were then blocked with 2% bovine serum albumin for 30 min at 37°C. Subsequently, cells were stained with MyHC primary antibody (1:100, F59 Santa) at 4°C overnight and were incubated with goat anti-mouse IgG conjugated to TRITC (1:100) (Zenbio). Finally, the cells nuclei were stained with DAPI (1 μg/Ml) to protect the cells from counterstaining by incubating the cells for 10 min at room temperature. The cells were observed and photographed using a fluorescent microscope.

### MiRNA Target Identification

miRNA and potential mRNA interaction binding sites were predicted by Targetscan^6^ (Lewis et al., 2005)andRNAhybrid2.2^7^ (Gruber et al., 2008). QPCR was used to detect the expression of target genes after transfection of mimics and inhibitors.

### Dual-Luciferase Assay

The 3′-UTR of *SBF2* was amplified and cloned into the pmirGLO dual-luciferase reporter eukaryotic expression vector completed by Shanghai Bioengineering Co., Ltd., Shanghai. The mutant *SBF2* 3′UTR plasmids were generated by missing the seven gga-miRNA-454 binding sites (5′-TGCACTA-3′). DF-1 cell (Fudan University Cell Bank) were co-transfected with 100 ng of the wild or mutant *SBF2* 3′UTR dual-luciferase reporter and 0.25 μL of the miR-454 mimic or negative control duplexes using Lipofectamine 3000 reagent (Invitrogen, United States) in 24 well plate. After transferring into the cells for 48 h, the cells were collected and the reaction intensity of firefly fluorescence and sea-renal fluorescence was measured, and the ratio of the two reaction intensities was calculated to correct.

### Statistical Analysis

Expression of all genes and miRNAs were calculated using 2^–ΔΔCT^ method, all data were represented as mean ± SEM based on at least 3 replicates for each treatment. The ANOVA program of IBM SPSS20 Statistics (SPSS Inc, Chicago, IL, United States) software was used to analyze the relative expression of each gene, and the multiple comparison was performed by Duncan method. The final result is plotted using GraphPad Prism 5 (GraphPad Software, Inc, San Diego, CA, United States).

## Results

### RNA-Seq Result

Collect 13 different periods of pectoral muscle tissue and two chicken embryos and five other tissues of D300, three replicates per sample, a total of 60 samples were used for next-generation sequencing. We obtained a total of 745,165,420 raw reads, with an average of 12,419,423 reads per sample. The raw reads Q20 was between 98.89–95.75% of these 60 samples. Q30 was between 97.88 and 91.6% (Supplementary Table S1). Then the information of the novel miRNAs of all samples was predicted, and the miRNA of at least two samples were predicted as the novel miRNA. Statistics of miRNA species in different developmental stages and in different tissues (Supplementary Figure S1). We calculated the base lengths of Clean data in 15 periods and found that the miRNA length distributions in different periods were basically the same, mainly concentrated between 21–24 nt (the percentage of totals ranged from 69.89 to 90.43) (Supplementary Figure S2). The number of miRNA expression gradually decreases with the development of muscles and eventually stabilizes. A total of 834 miRNAs including 631 preciously identified miRNAs and 203 novel miRNA candidates were obtained across 13 pectoral muscle developmental stages. Additionally, a total of 672 miRNAs including 548 known miRNAs and 124 novel miRNAs were identified among six tissues collected at the age of 300 days (Supplementary Table S2). Sequencing data has been uploaded to NCBI (GSE 139304).

### MiRNAs Can Be Classified as Three Time-Dependent Patterns According Their Expression Profiles

In order to obtain an overview of the miRNA expression profiles of 60 Tibetan chicken samples, we performed hierarchical clustering analysis and principal component analysis. PCA and HCL analysis was performed using the expression profiles of different periods of miRNAs. Three clusters containing early -embryonic (Pre_e, including E9 and E12), late embryonic (Lat_e, including E15, E18, E20, and D1) and postnatal stages (Bir_g, including D36, D100, D300, Y2, Y5, Y8, and Y12) were found (Supplementary Figures S3B, S3D). Indicates that major distinctions in the miRNA expression profiles occurred during these three time periods. Clustering analysis revealed that the lowest miRNA expression similarity was shown between Y8 and Y12 (Pearson correlation, *R* = 0.809), whereas E9 and E12 showed the highest expression correlation (Pearson correlation, *R* = 0.996), suggesting the correlation between embryonic prophase were relatively weak compared to with the post-embryonic growth periods stages. Further, the different tissue samples examined were separated based on tissue type, in which the brain, liver, kidney and heart were clearly separated (Supplementary Figures S3A, S3C).

### GgmiRNA-454 Is a Time-Dependent miRNA

**FIGURE 1:**
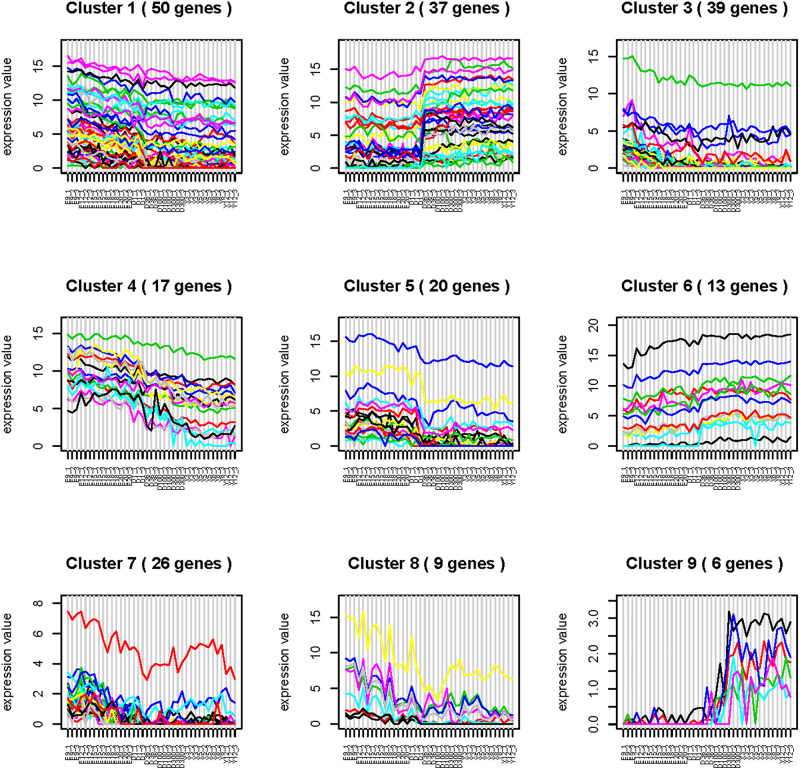
Time-series analysis.

**FIGURE 2:**
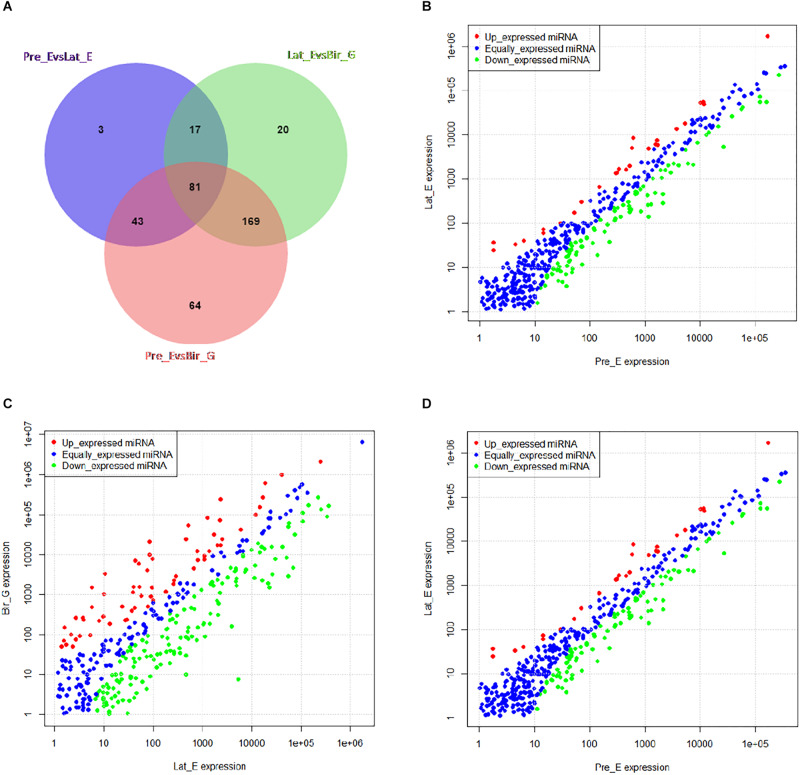
Time difference analysis: **(A)** The Venn diagram indicates the number of dynamic miRNAs; **(B)** miRNAs differentially expressed between Pre_E and Lat_E; **(C)** miRNAs differentially expressed between Lat_E and Bir_G; **(D)** miRNAs differentially expressed between Pre_E and Bir_G.

**FIGURE 3:**
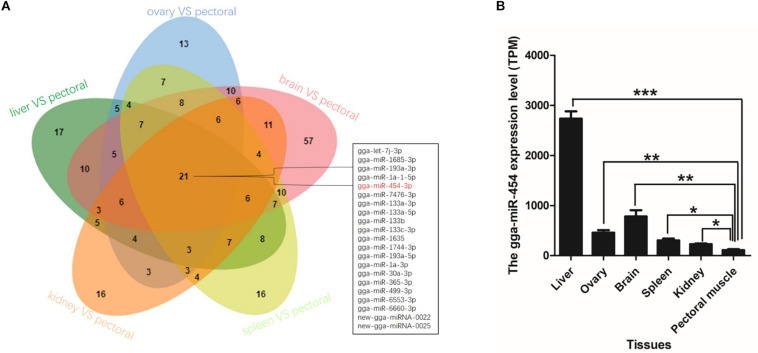
Organizational difference analysis: **(A)** The Venn diagram indicates the number of different tissues miRNAs; **(B)** Expression of gga-miRNA-454 in different tissues.

Time series analysis was performed to explore the developmental dynamics of miRNAs across chicken pectoral muscle. A total of 271 miRNAs showed time-course patterns during the stage of chicken growth and developmental curve, divided into 9 clusters (Supplementary Tables S7, S8). Gga-miRNA-454 showed a trend of increasing first, then decreasing, and finally stabilizing in the four cluster (Figure 1). To evaluate the stage-dependent transcriptomic activities across the life cycle of the chicken, we performed differential miRNA expression analysis by comparing any two adjacent developmental stages. These developmental stages include early -embryonic, late embryonic and postnatal stages. A total of 81 dynamic DE-miRNAs were obtained by time difference analysis (Figure 2A). The results showed that 144 miRNAs (32 up-regulated 112 down-regulated)were detected in early -embryonic stage and late embryo stage (Pre_E vs Lat_E), and 287 miRNAs (88 up-regulated, 199 Down-regulation) were detected in late postnatal and postnatal growth stages (Lat_E vs Bir_G), and 357 miRNAs (82 up-regulation, 275 down-regulation) were detected in early -embryonic and post-natal growth stages (Pre_E vs Bir_G) (Figures 2B–D). GgmiRNA-454 is differentially expressed in the above three periods. In addition, a total of 21 miRNAs from tissue differential analysis also predicted targets for 291 unique genes (Figure 3A). Gga-miR-454 has significant differences compared with other tissues (Figure 3B).

### Target Prediction and Functional Roles

**FIGURE 4:**
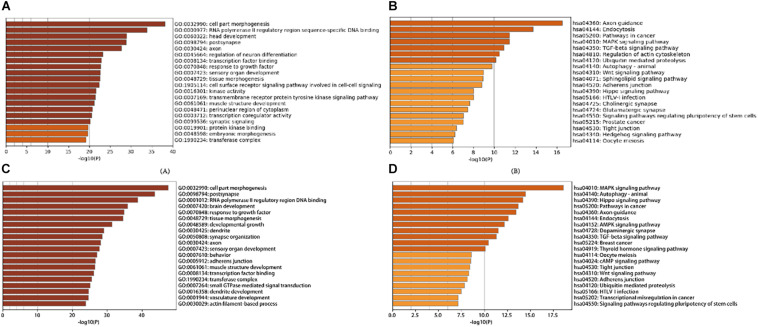
Gene Ontology and KEGG Pathway Enrichment Analysis of target genes of stage development and tissue differentially expressed miRNAs: **(A)** Heatmap of Gene Ontology (GO) of target genes of tissue differentially expressed miRNAs enriched terms colored by *p*-values; **(B)** Heatmap of Kyoto Encyclopedia of Genes and Genomes (KEGG) of target genes of tissue differentially expressed miRNAs enriched terms colored by *p*-values; **(C)** Heatmap of Gene Ontology (GO) of target genes of stage development differentially expressed miRNAs enriched terms colored by *p*-values; **(D)** Heatmap of Kyoto Encyclopedia of Genes and Genomes (KEGG) of target genes of stage development differentially expressed miRNAs enriched terms colored by *p*-values.

A total of 21 DE-miRNAs from tissue differential analysis predicted a target of 291 unique genes. GO functional classification and enrichment analysis of each gene was annotated by Metascape. The top 20 GO enrichment items were classified into three functional groups: biological process group (11 items), molecular function group (5 items), and cellular component group (4 items). Target genes were mainly enriched in Oocyte meiosis, Hedgehog signaling pathway, Tight junction, Regulation of actin cytoskeleton, Signaling pathways regulating pluripotency of stem cells, Wnt signaling pathway, TGF-beta signaling pathway, MAPK signaling pathway, Pathways in cancer, etc. (Figures 4A,B and Supplementary Tables S3, S4).

There are 58 miRNAs that overlap in dynamic DE-miRNA and time-course miRNA. A total of 58 time-dependent miRNAs predicted a target of 2,546 unique genes. In addition, enrichment results of 58 time-dependent miRNAs were obtained (Figures 4C,D and Supplementary Tables S5,S6). The top 20 GO enrichment items were classified into three functional groups: biological process group (13 items), molecular function group (2 items), and cellular component group (5 items). Regarding KEGG, Target genes were significantly enriched in 20 canonical pathways including Thyroid hormone signaling pathway, Breast cancer, TGF-beta signaling pathway, Dopaminergic synapse, AMPK signaling pathway, Endocytosis Axon guidance, Pathways in cancer, Hippo signaling pathway, Autophagy -animal, MAPK signaling pathway, etc.

### Verify the Accuracy of the High-Throughput Sequencing by q-PCR

In order to verify the accuracy of high-throughput sequencing, we randomly performed q-PCR detection of 6 known differentially expressed miRNAs and 3 novel miRNAs. The results showed that q-PCR expression of miRNAs were completely consistent with our sequencing data (Supplementary Figure S4). We found that miR-133c-3p had highest expression level in discrete periods among nine miRNAs, and the trend of expression level increased with time. MiR-133-3p is a key regulatory molecule of MiR-133 family, specifically expressed in muscle and is capable of inducing myoblast proliferation (Chen et al., 2006).

### Primary Chicken Myoblasts Show Muscle Fusion and Achieve Transfection Efficiency

**FIGURE 5:**
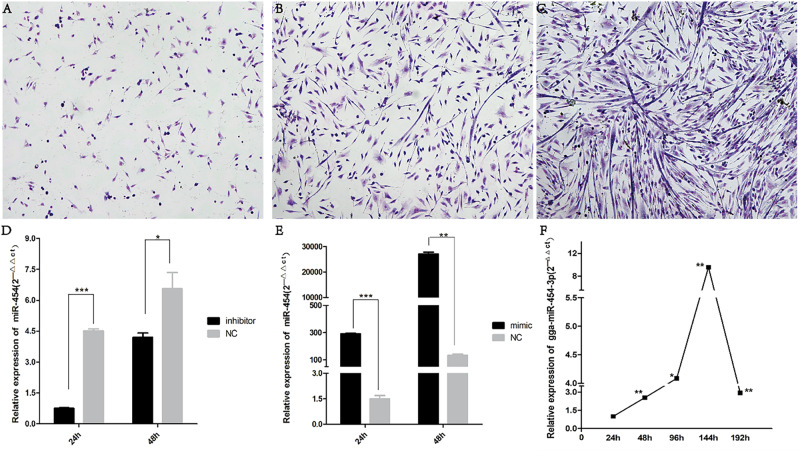
Cell staining and transfection efficiency detection: **(A)** 24-h Giemsa staining (100×); **(B)** 48-h Giemsa staining (100×); **(C)** 72-h Giemsa staining (100×); **(D)** expression of gga-miR-454 after cell transfection of inhibitor; **(E)** expression of gga-miR-454 after cell transfection of mimic; **(F)** expression of gga-miR-454 during the growth of chicken primary myoblasts.

Cells were stained at the 24, 48, and 72th of differentiation to visualize myotubes, cytoplasm and nuclei, the nucleus and cytoplasm of the cells can be clearly seen (Figures 5A–C). From the attachment of the cells for 24 h, the cells are found in a single state. At 48 h, the cells become full and elongated at a dense concentration, and many long spindle-like solitary nuclei appear in the field of view, then 72 h, saw the number of myotubes increased, the shape became thicker. At the same time, we measured the transfection efficiency of cells cultured in vitro (Figures 5D,E).

### Gga-miRNA-454 Has no Effect on Myoblast Proliferation but Inhibits Myoblast Differentiation

**FIGURE 6:**
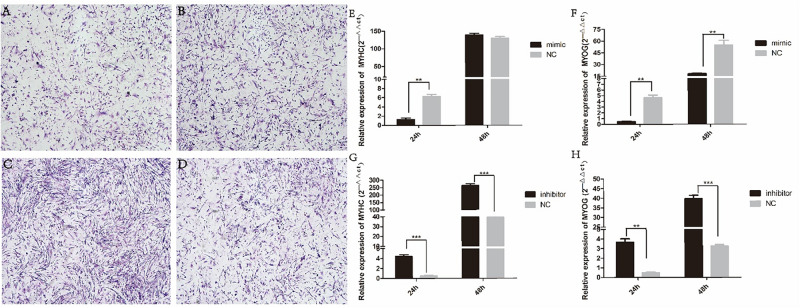
Effect of transfection of gga-miRNA-454 mimics and inhibitors on myoblast differentiation: **(A)** Giemsa staining of transfected gga-miRNA-454 mimic cells (40×); **(B)** Giemsa staining of transfected mimic-NC cells (40×); **(C)** Giemsa staining of transfected gga-miRNA-454 inhibitor cells (40×); **(D)** Giemsa staining of transfected inhibitor-NC cells (40×); **(E–H)** Detection of expression of differentiated genes after transfection of mimetics and inhibitors by QPCR.

**FIGURE 7:**
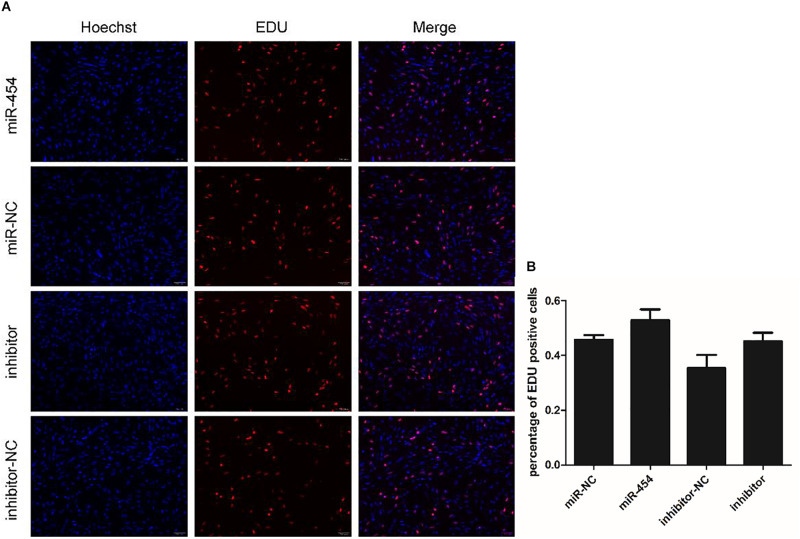
Effect of gga-miR-454 on myoblast proliferation: **(A)** EDU detection of myoblasts by mimics and inhibitors of gga-miR-454; **(B)** Percentage of EDU-positive cells transfected with gga-miR-454 mimics and inhibitors.

**FIGURE 8:**
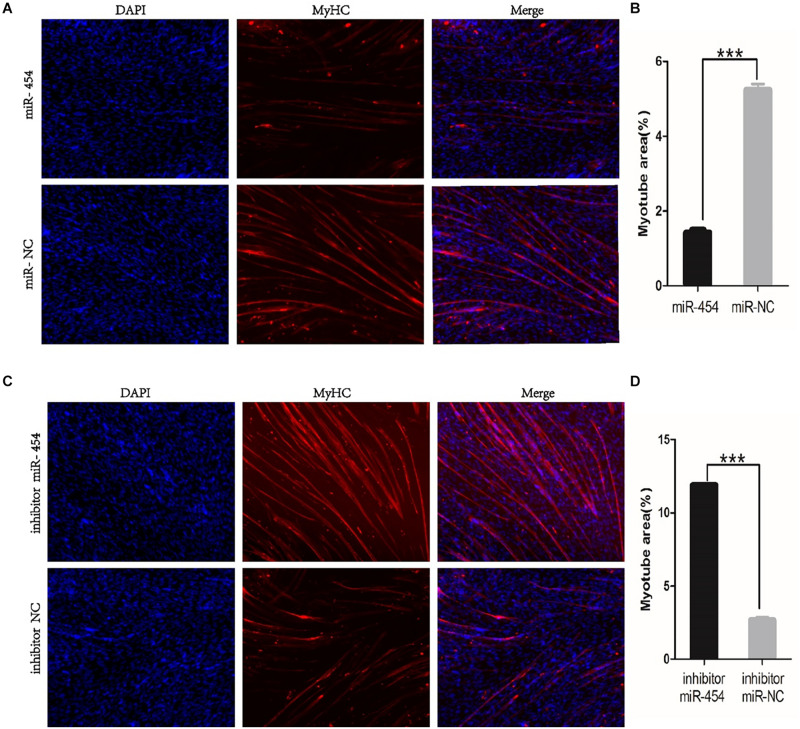
Immunofluorescence of transfected gga-miR-454 mimic and inhibitor (100×): **(A)** Immunofluorescence of transfected gga-miR-454 mimic; **(B)** Immunofluorescence area statistics of different transfected gga-miR-454 mimic; **(C)** Immunofluorescence of transfected gga-miR-454 inhibitor; **(D)** Immunofluorescence area statistics of different transfected gga-miR-454 mimic.

We found that miR-454 showed a trend of increasing initially and then decreasing during the process of myoblast proliferation and differentiation, suggesting its potential involvement in myoblast proliferation and differentiation processes (Figure 5F). To observe the effects of gga-miRNA-454 on myoblast proliferation, we transfected chicken primary myoblast cultured in GM with an miR-454 mimic and inhibitors or scrambled negative control duplexes. Giemsa staining demonstrated that proliferation rate of miR-454-transfected cells was remarkably reduced compared with that of the control cells with larger myotube density in the visual field (Figures 6A–D), whereas miR-454 loss-of-function promoted cell differentiation rate, indicating that miR-454 can inhibit chicken myoblast proliferation. The QPCR assay showed that the mRNA expression level of *MyHC* and *MyoG* in the gga-miRNA-454 mimic group was significantly decreased at 24 and 48 h after transfection (Figures 6E,F). NC in the Figure 6E and Figure 6F represents miRNA mimic negative control. In the gga-miRNA-454 inhibitor group, the mRNA expression levels of *MyHC* and *MyoG* showed a significant increase at 24 and 48 h (Figures 6G,H). NC in the Figure 6G and Figure 6H represents miRNA inhibitor negative control. 5-Ethynyl-2′-deoxyuridine (EdU) cell proliferation assay showed that miR-454-transfected cells had no effect on myoblast proliferation (Figure 7). In addition, miR-454 reduced the formation of myotubes, and the area of myotube leveled with MyHC was significantly decreased (*P* < 0.001) after 48 h of differentiation in the transfected gga-miR-454 mimic compared with that of control group (Figures 8A,B). In contrast, the area of MyHC fluorescently labeled protein was significantly increased after gga-miR-454 inhibitor transfection (*P* < 0.001) (Figures 8C,D). Therefore, the above results showed that gga-miRNA-454 play an inhibitory role in the differentiation of chicken myoblasts.

### Gga-miRNA-454 Inhibits Myoblast Differentiation by Targeting the Myotube-Associated Protein SBF2

**FIGURE 9:**
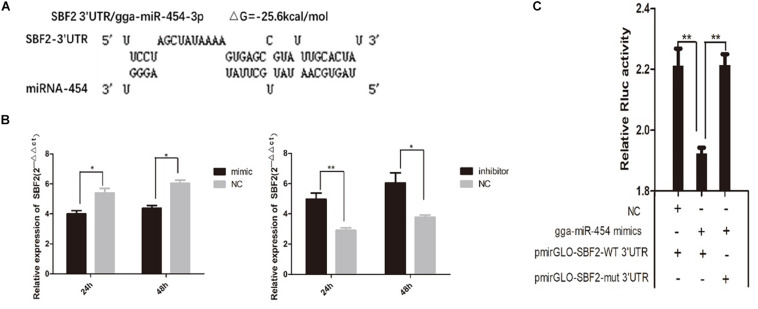
Targeting relationship between gga-miRNA-454 and SBF2 gene: **(A)** Target gene prediction; **(B)** Detection of SBF2 gene expression after transfection of mimic and inhibitor by QPCR; **(C)** Dual luciferase activity assay.

Target gene prediction results showed that chicken SBF2 3′UTR has a potential binding site with miR-454 with a good target relationship (ΔG = −25.6 kcal/mol) (Figure 9A). Here, we studied the involvement of SBF2 in the inhibition of miR-454 during chicken myoblast proliferation. QPCR results shown that SBF2 gene expression levels were significantly inhibited in the gga-miRNA-454 mimic group compared with the control group, whereas significantly increased in the gga-miRNA-454 inhibitor group. SBF2 can be a potential target gene for miRNA-454 (Figure 9B). The transfection of myoblasts with miR-454 in GM downregulated SBF2 mRNA expression level, and the inhibition of endogenous miR-454 in GM using miR-454 inhibitor increased SBF2 mRNA expression. In addition, to validate whether *SBF2* is a target gene of miR-454, we constructed two dual-luciferase reporters with the wide-type and mutant 3′-UTRs of *SBF2*, respectively. Compared with miR-NC, pmirGLO-miR-454 induced a significant decrease in normalized luciferase activity of the vector containing the putative miRNA-binding site. In addition, the mutation of the miR-454-responsive elements in the binding site of SBF2-3′-UTR (SBF2-mut) resulted in loss of the inhibitory effects of miR-454 (Figure 9C). These results suggested that the predicted site is a target of miR-454 and is responsible for miR-454 targeting of the SBF2-3′-UTR.

## Discussion

As a public data for miRNA expression of Tibetan chicken, we have generated a comprehensive chicken RNA-Seq transcriptomic encompassing five organs across 15 developmental stages from juvenile to old age for both sexes. The main muscle fibers of birds are formed within six days of hatching and the secondary muscle fibers are mainly formed during the embryo development at 12–16 days (Liu, 2012). Taking into account this, the embryonic period of day 5, 7, 9, 12, 15 and day 1 after birth has become a common time point for studying muscle development. Currently, there are few reports on the role of miRNAs in chicken muscle growth and development. Here, we predicted 203 and 124 novel miRNAs in Tibetan chicken from sequencing of samples from different periods and different tissue, respectively. Further functional characterization of these miRNAs had a deeper understanding of Tibetan chicken muscle specificity and developmental process dependence. In this study, a total of 21 DE-miRNAs were screened based on tissue difference analysis, Of them, miR-1 and miR-133 were known for their key roles in skeletal muscle development (Keith and Wen, 2015). In addition, miR-193 has been reported to be associated with myotonic dystrophy type 2 (DM2) (Greco et al., 2012) and miR-365 associated with inhibition of vascular smooth muscle cell proliferation (Peng et al., 2014). There are also some miRNAs to be taken into account, such as miR-6553-3p, miR-1744-3p, miR-1635.

Based on the results of sample correlation analysis, it is found that miRNAs of stage E9 and E12 were closely interrelated, E15, E18, E20, and D1 shared a similar gene expression signature, whereas D36, D100, D300, Y3, Y5, Y8, and Y12 were clustered together. This result is consistent with the stage of chicken muscle growth and development. Therefore, we divide the 13 periods into three major periods: early -embryonic, late embryo and postnatal stages. Finding miRNAs related to muscle development using differential expression analysis and single time series analysis methods in different periods. A total of 271 time-course miRNAs were screened by applying biological methods, including some muscle-specific miRNAs, such as miR-1 and miR-133, and some non-specific miRNAs, such as miR-23a, miR-26a, miR-181, miR-222 have been reported to be involved in the regulation of muscle growth and development (Dey et al., 2012; Hudson et al., 2014; Bloch et al., 2015). A total of 58 time-dependent miRNAs are both DE-miRNAs and show time-course patterns. The time-dependent miRNAs included let-7, miR-133, miR-208b miR-499. Many miRNAs had been found to participate in the differentiation of muscle fiber types in developing embryos or adult muscles. The regulatory roles of these miRNAs are mainly related to TGF-beta signaling pathway, AMPK signaling pathway, MAPK signaling pathway, etc. These pathways had been reported to be involved in muscle growth and development (Schiaffino et al., 2013; Thomson, 2018; Du et al., 2019). The most significantly enriched pathway was MAPK signaling pathway. Regulation of extracellular growth factors through activation of this pathway affects myoblast differentiation. Notably, Endocytosis Axon guidance, Pathways in cancer, Hippo signaling pathway, Autophagy -animal, Thyroid hormone signaling pathway, Breast cancer, also related to muscle growth and development. When the miRNA mimic transfection concentration reached 100 nM and the miRNA inhibitor concentration reached 200 nM, it could achieve the effect of over-expression and inhibit miRNA-454 (Figures 5D,E).

Among these miRNAs, miR-454 attracted our attention because it is a time-dependent and tissue-differential expression miRNA. In poultry, only little research on chicken gga-miR-454 have been reported. Recently, gga-miR-454 has been identified as direct inhibiter of infectious bursal disease virus (IBDV) replication by targeting the viral genomic segment B (Fu et al., 2018). In addition, gga-miR-454 expression was found lower in eight diverse tissues of chickens not infected with Marek’s disease virus, which may play a major role in the pathogenesis of Marek’s disease and tumor transformation (Yao et al., 2008). The miR-454 has been shown to be down-regulated in certain human malignancies and is associated with tumor progression. It plays a major role in colorectal cancer, prostate cancer, gastric cancer, lung cancer, liver cancer, and osteosarcoma (Wu et al., 2014; Liang et al., 2015; Song et al., 2017).

The miR-454 regulates triglyceride synthesis in bovine mammary epithelial cells by targeting PPAR-γ, which may be a crucial factor in enhancing the quality of dairy products (Zhang et al., 2019). Hitherto, the function of miRNA-454 has been explored in several human diseases, and its response to pathogenic infection, however, its role in muscle development has not yet been reported (Wu et al., 2014; Liang et al., 2015; Song et al., 2017). We have shown that gga-miR-454 was up-regulated during the proliferation and then gradually down-regulated during of chicken myoblasts differentiation. Our results demonstrated that gga-miR-454 inhibits the expression of MyHC and MyoG gene, and inhibit myoblast differentiation.

The dual luciferase reporter gene assay indicated that the *SBF2* gene is a target gene of gga-miR-454. The SBF2 gene encodes a member of the pseudo phosphatase which belongs to myotube-associated protein family (Othmane et al., 1999). In addition, interference with *SBF2* expression can inhibit the proliferation and invasion of human oral cancer cells and induce apoptosis, suggesting that the role of *SBF2* gene can be determined by inhibiting the TGF-β pathway (Tian et al., 2016). Two representative TGF-β family members including TGF-β and BMP are endogenously control myogenesis the TGF-β pathway in a phase-specific manner (Furutani et al., 2011). We assumed that the *SBF2* gene may also exert an effect on myoblast differentiation by acting on TGF-β pathway. However, the precise adjustment mechanism still worth for further study.

## Conclusion

In this study, we sequenced a total of 60 samples from 15 developing stages of the pectoral muscle and five other tissues at 300 days of Tibetan chicken. We characterized the expression patterns of miRNAs across muscle developmental stages, and found that the chicken growth and development stage was divided into early -embryonic, late embryo and postnatal stages. We identified 81 and 21 DE-miRNAs by comparing the miRNA profiles of pectoral muscle of 3 broad periods and different tissues, respectively; and 271 miRNAs showed time-course patterns. Their potential targets were predicted and used for functional enrichment to understand their regulatory functions. Significantly, GgmiRNA-454 is a time-dependent and tissue-differential expression miRNA. In order to elucidate the role of gga-miRNA-454 in the differentiation of myoblasts, we cultured chicken myoblasts in vitro. The results show that although gga-miRNA-454-3p initiates increase and thereafter decrease during the chicken myoblasts differentiation, it had no effect on primary myoblasts proliferation. Furthermore, we confirm that gga-miRNA-454 inhibits myoblast differentiation by targeting the myotube-associated protein SBF2.

## Data Availability Statement

The datasets generated for this study can be found in the GSE139304.

## Ethics Statement

All experimental protocols were subject to the Institutional Animal Care and Use Committee in the College of Animal Science and Technology, Sichuan Agricultural University, China.

## Author Contributions

MC, SZ, and ZX were involved in the conceptualization and wrote the original draft of the manuscript and pervised the experiments, analyzed the data, and supported the work. JG performed the experiments and data analysis. All authors read and approved the final version of the manuscript. All authors are responsible and guarantors for the work.

## Conflict of Interest

The authors declare that the research was conducted in the absence of any commercial or financial relationships that could be construed as a potential conflict of interest.

## Footnotes

1 http://www.girinst.org/

2 http://www.mirbase.org/

3 http://www.mirdb.org/

4 http://www.targetscan.org/vert_72/

5 http://metascape.org/

6 http://www.targetscan.org/vert_72/

7 https://bibiserv.cebitec.un-i-bielefeld.de/rnahybrid/
