# Supplementary material for: MiRNA Profiling in Pectoral Muscle Throughout Pre- to Post-Natal Stages of Chicken Development
Source: Front Genet. 2020 Jun 23;11:570. doi: 10.3389/fgene.2020.00570 (PMC7324647; doi:10.3389/fgene.2020.00570)
Supplement: Supplementary file 1 [file Data_Sheet_1.ZIP › Supplementary Material/Figure S3.pdf]

Fig S3. Correlation of samples in chickens

(A) Pearson correlation coefficient between samples of different organizations
(B) Pearson correlation coefficient between samples of different developmental stages
(C) Principal component analysis between samples of different organizations
(D) Principal component analysis between samples of different developmental stages

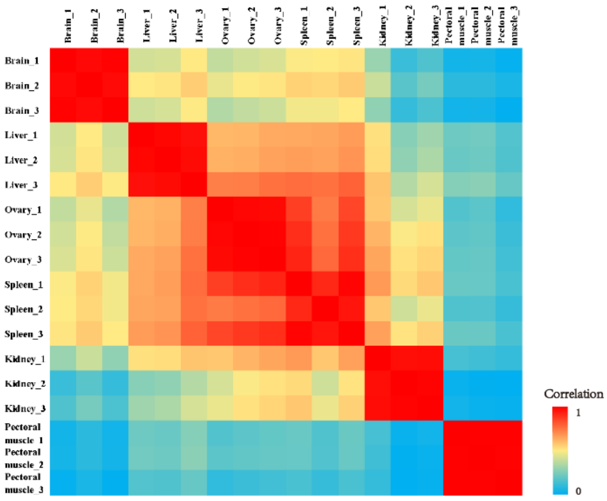

(A)

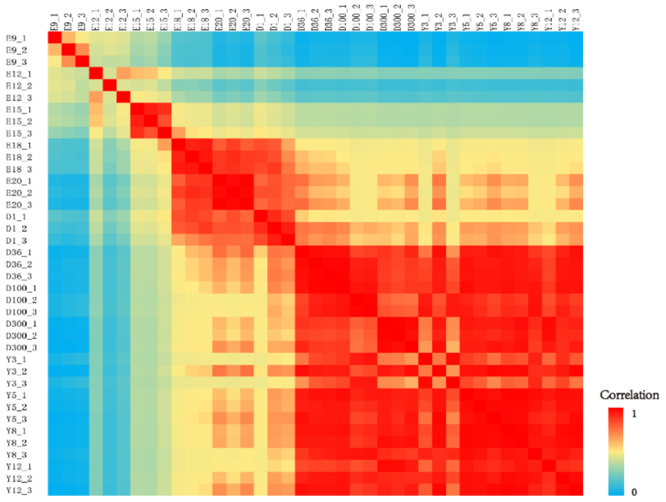

(B)

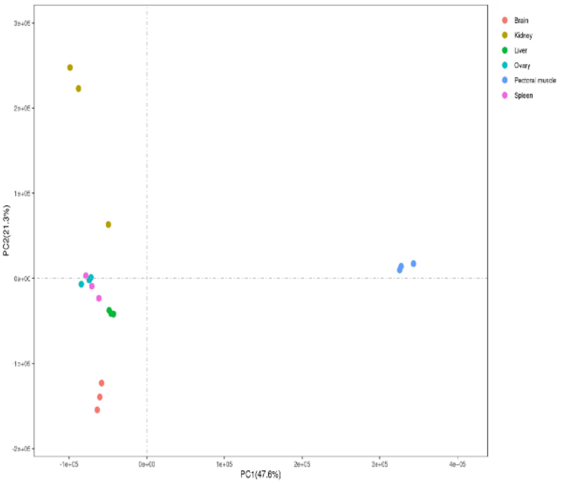

(C)

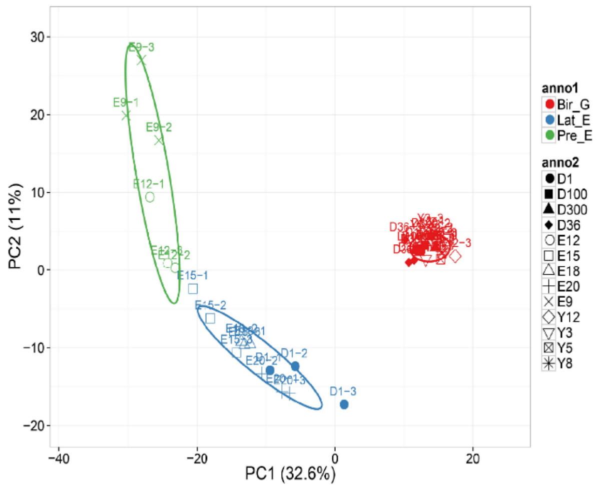

(D)
