# Supplementary material for: MiRNA Profiling in Pectoral Muscle Throughout Pre- to Post-Natal Stages of Chicken Development
Source: Front Genet. 2020 Jun 23;11:570. doi: 10.3389/fgene.2020.00570 (PMC7324647; doi:10.3389/fgene.2020.00570)
Supplement: Supplementary file 1 [file Data_Sheet_1.ZIP › Supplementary Material/Figure S4.pdf]

Fig S4. Q-PCR verification sequencing results

(A) High throughput sequencing of miRNA expression;
(B) MiRNA expression using q-PCR;

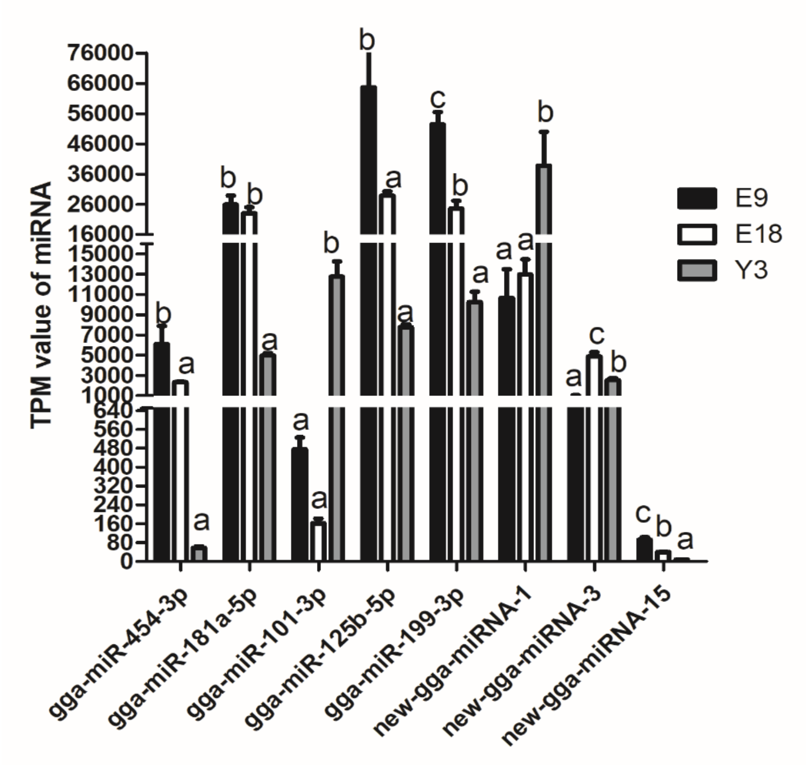

(A)

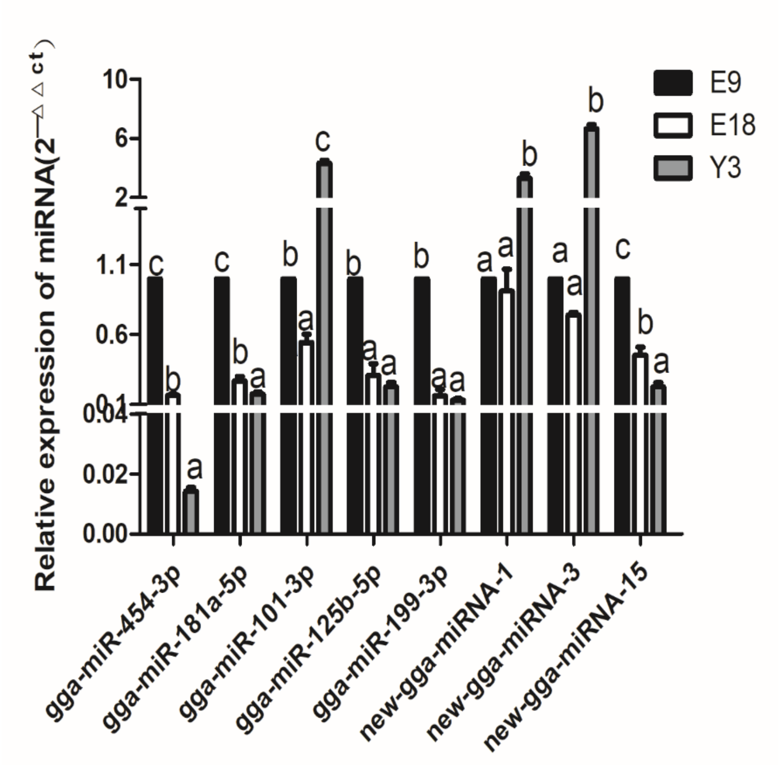

(B)
