# Supplementary material for: MiRNA Profiling in Pectoral Muscle Throughout Pre- to Post-Natal Stages of Chicken Development
Source: Front Genet. 2020 Jun 23;11:570. doi: 10.3389/fgene.2020.00570 (PMC7324647; doi:10.3389/fgene.2020.00570)
Supplement: Supplementary file 1 [file Data_Sheet_1.ZIP › Supplementary Material/supplemental table 1.pdf]

Table S1. Descriptive summary of non-coding RNAs reads.

Stage	Tissue	Replicate	Raw Reads	Raw Bases	Clean reads	Clean Bases	Q20 (%)	Q30 (%)	Clean ratio (%)
Embryonic day 5	Whole embryoe	E5_1	11717838	585891900	9445172	472258600	97.97	95.8	80.61
		E5_2	12459492	622974600	10647581	532379050	97.91	95.56	85.46
		E5_3	13705971	685298550	11070098	553504900	97.58	95.04	80.77
Embryonic day 7	Whole embryo	E7_1	13355853	667792650	11490277	574513850	97.73	95.38	86.03
		E7_2	11775403	588770150	10091937	504596850	97.77	95.44	85.70
		E7_3	10966536	548326800	9451567	472578350	98.25	96.39	86.19
Embryonic day 9	Pectoral muscle	E9_1	11105972	555298600	10177680	508884000	98.26	96.45	91.64
		E9_2	11922959	596147950	9576481	478824050	98.09	96.12	80.32
		E9_3	11623606	581180300	9909581	495479050	98.20	96.33	85.25
Embryonic day 12	Pectoral muscle	E12_1	12753867	637693350	11514871	575743550	98.23	96.34	90.29
		E12_2	11367638	568381900	9347754	467387700	98.05	95.99	82.23
		E12_3	12110097	605504850	10506872	525343600	98.12	96.13	86.76
Embryonic day 15	Pectoral muscle	E15_1	11233648	561682400	9644079	482203950	98.13	96.18	85.85
		E15_2	12315150	615757500	10274887	513744350	97.94	95.78	83.43
		E15_3	12766831	638341550	10472098	523604900	97.75	95.37	82.03
Embryonic day 18	Pectoral muscle	E18_1	12020461	601023050	10499106	524955300	97.60	95.11	87.34
		E18_2	11253272	562663600	9855109	492755450	97.99	95.82	87.58
		E18_3	11774589	588729450	9800409	490020450	97.98	95.84	83.23
Embryonic day 20	Pectoral muscle	E20_1	11565011	578250550	9878278	493913900	97.92	95.70	85.42
		E20_2	11423191	571159550	10289627	514481350	97.77	95.46	90.08
		E20_3	12933807	646690350	10229127	511456350	97.70	95.22	79.09
At the age of 1 days	Pectoral muscle	D1_1	12265316	613265800	10408394	520419700	98.10	96.11	84.86
		D1_2	13286996	664349800	11757924	587896200	98.02	95.95	88.49
		D1_3	12004696	600234800	10633176	531658800	97.61	95.18	88.58
At the age of 36 days	Pectoral muscle	D36_1	12841066	642053300	10643398	532169900	96.32	92.56	82.89
		D36_2	13363195	668159750	10366623	518331150	96.73	92.94	77.58
		D36_3	12663948	633197400	11113703	555685150	98.35	96.81	87.76
At the age of 100 days	Pectoral muscle	D100_1	12763733	638186650	11052306	552615300	98.27	96.64	86.59
		D100_2	12380272	619013600	11329241	566462050	98.32	96.76	91.51
		D100_3	11503337	575166850	9987185	499359250	98.21	96.56	86.82
At the age of 300 days	Pectoral muscle	D300_1	12966139	648306950	12233982	611699100	98.85	97.81	94.35
		D300_2	13311823	665591150	12419973	620998650	98.89	97.88	93.30
		D300_3	13389267	669463350	12611099	630554950	98.85	97.82	94.19
At the age of 3 years	Pectoral muscle	Y3_1	11700956	585047800	10259958	512997900	97.95	95.97	87.68
		Y3_2	12653478	632673900	10666588	533329400	96.26	92.45	84.30
		Y3_3	11091700	554585000	9757042	487852100	98.22	96.53	87.97
At the age of 5 years	Pectoral muscle	Y5_1	12357792	617889600	10773149	538657450	98.11	96.35	87.18
		Y5_2	12648050	632402500	10643780	532189000	98.10	96.24	84.15
		Y5_3	10897281	544864050	9793541	489677050	98.28	96.67	89.87
At the age of 8 years	Pectoral muscle	Y8_1	10875309	543765450	9807527	490376350	98.29	96.71	90.18
		Y8_2	11804560	590228000	9886534	494326700	98.15	96.47	83.75

		Y8_3	11624472	581223600	10710795	535539750	98.18	96.44	92.14
At the age of 12 years	Pectoral muscle	Y12_1	14637612	731880600	13010995	650549750	98.75	97.45	88.89
		Y12_2	11834791	591739550	10331616	516580800	98.27	96.63	87.30
		Y12_3	13394371	669718550	11386894	569344700	95.75	91.60	85.01
At the age of 300 days	Brain	Brain_1	14427259	721362950	12625077	276362294	98.34	96.22	87.51
		Brain_2	13955210	697760500	12251469	274973013	98.14	95.87	87.79
		Brain_3	11726536	586326800	10241526	224477010	98.21	96.02	87.34
At the age of 300 days	Liver	Liver_1	12578136	628906800	10961286	241736535	98.08	95.82	87.15
		Liver_2	12179928	608996400	10752474	241519038	97.77	95.43	88.28
		Liver_3	11874646	593732300	10397486	232176820	98.06	95.84	87.56
At the age of 300 days	Ovary	Ovary_1	12938349	646917450	11639429	254221914	98.41	96.40	89.96
		Ovary_2	13680766	684038300	12525439	285239706	98.08	95.77	91.56
		Ovary_3	12786946	639347300	11675159	268383488	98.34	96.26	91.31
At the age of 300 days	Spleen	Spleen_1	12840466	642023300	11208342	246962511	98.21	96.06	87.29
		Spleen_2	14119721	705986050	12531655	279558347	98.13	95.87	88.75
		Spleen_3	13246252	662312600	11556617	254881453	98.27	96.11	87.24
At the age of 300 days	Kidney	Kidney_1	12367339	618366950	10805639	257513828	98.25	96.02	87.37
		Kidney_2	13962934	698146700	12622332	283292019	98.15	95.88	90.4
		Kidney_3	12069580	603479000	10789336	241986024	98.41	96.36	89.39
