# Supplementary material for: MiRNA Profiling in Pectoral Muscle Throughout Pre- to Post-Natal Stages of Chicken Development
Source: Front Genet. 2020 Jun 23;11:570. doi: 10.3389/fgene.2020.00570 (PMC7324647; doi:10.3389/fgene.2020.00570)
Supplement: Supplementary file 1 [file Data_Sheet_1.ZIP › Supplementary Material/supplemental table 2 .pdf]

new_name	consensus mature sequence	consensus star sequence	consensus precursor sequence
new-gga-miRNA-1	ugagaugaagcacuguagcucg	ggugcagugcugcaucucugguc	ggugcagugcugcaucucuggucaauugggagucugagaugaagcacuguagcucg
new-gga-miRNA-2	caacggaaucccaaaagcagcug	cugcccugggauuucguuacc	caacggaaucccaaaagcagcuguccccgcagcgcccagcugcccugggauuucguuacc
new-gga-miRNA-3	aaugacacgaucacucccgcugag	caucggggaugucgugucugucc	aaugacacgaucacucccgcugagcgcgcagccacagagccaucggggaugucgugucugucc
new-gga-miRNA-4	acccuguagacucgaauuugug	caaauucgucucuaggggaau	acccuguagacucgaauuugugugagcgucuccgagucgcaaauucgucucuaggggaau
new-gga-miRNA-5	uuuggcaagguagaacucacacu	ugguucuagacuugccaacu	uuuggcaagguagaacucacacuggugcgcucgcaggauccgguguucuagacuugccaacu
new-gga-miRNA-6	aggacugucuaaccugagaau	aucucagguucgucagcccauga	aggacugucuaaccugagaauggugaaacaugagggucaaucucagguucgucagcccauga
new-gga-miRNA-7	ugagguagucgguuguauuguu	cgaugcagccgacuacuuuccc	ugagguagucgguuguauuguugggggccgggauggggccccacacucgagguaacgaugcagccgacuacuuuccc
new-gga-miRNA-8	aacaguaagaguugaugugcgg	ugcaugugagcuccuacugccg	aacaguaagaguugaugugcggcgaacgcagccugcaugugagcuccuacugccg
new-gga-miRNA-9	ucagugcaucacagaacuuggu	gagguucuguccuacacuccggcu	gagguucuguccuacacuccggcuguggcuaugggcagucagugcaucacagaacuuggu
new-gga-miRNA-10	agccacugacuaacgcacauug	cugugcgugugacagcggcu	agccacugacuaacgcacauugugcucucggcgacuccacugugcgugugacagcggcu
new-gga-miRNA-11	uccagcaucagugauuuuguug	aacaauauccuggugcugagu	aacaauauccuggugcugagugaguugcacacagagacuccagcaucagugauuuuguug
new-gga-miRNA-12	ccucgacacaagcuuuuguc	cugauucugggucggggu	cugauucugggucgggguucguacguagcagagcagcucccucgcugcgaucuauugagaguagcccucgacacaagcuuuu
new-gga-miRNA-13	uaacagucuacagucauggcu	accuuggcucuagacugcuuacu	accuuggcucuagacugcuuacugcugcguagggacaggacaguaacagucuacagucauggcu
new-gga-miRNA-14	accguggcuuuagauuguuacu	uaacagucuacagccauggucg	accguggcuuuagauuguuacugugcugcugggggggguaacagucuacagccauggucg
new-gga-miRNA-15	aagcccuuaccccaaaaagcau	cuuuuugcggucugggcuugc	cuuuuugcggucugggcuugcuguacauaacuaccuacccgggaagcccuuaccccaaaaagcau
new-gga-miRNA-16	gcucgacacaagcuuuugu	cggggccggguccggcggg	gcucgacacaagcuuuugucggagcgcggaggcgcgcgagcgcgcgcgugcggcgccccggcgcggggccggguccggcg
new-gga-miRNA-17	cccccaucgauuugcucuacg	aagggcaaacccgaugggcac	cccccaucgauuugcucuacgaacugaugaagcagaagggcaaacccgaugggcac
new-gga-miRNA-18	cacagcaaguguagacaggca	ccugucugugccugcuguaca	ccugucugugccugcuguacaggugagaggauguucugcacagcaaguguagacaggca
new-gga-miRNA-19	cuuuuugcggucugggcuugc	aagcccuuaccccaaaaaggau	cuuuuugcggucugggcuugcugugccccacagcucucaggaagcccuuaccccaaaaaggau
new-gga-miRNA-20	gaugaaaucccccuauggccugu	ggauaggugggguuucauugaau	gaugaaaucccccuauggccuguuugugaugguaccgcaggauaggugggguuucauugaau
new-gga-miRNA-21	uucccaaguacguugguaguca	acuccaaacaugccugggaagcuu	acuccaaacaugccugggaagcuuuaguaaaugaauaaaaauucccaaguacguugguaguca
new-gga-miRNA-22	uccgacacaagcuuuugu	uugauucugggucggggg	uugauucugggucggggguuucguacguagcagacgcagcucccucgcugcgaucuauugagagucagcccuccgacacaagc
new-gga-miRNA-23	aggcaguguauuguuaguuagcu	agcaacuaacuacacugccaca	aggcaguguauuguuaguuagcugcucuucauacaccagcaacuaacuacacugccaca
new-gga-miRNA-24	cugaccgcucucucccgcaga	cagcgggggcgggccgggccgcg	cagcgggggcgggccgggccgcggcgcagcgcugaccgcucucucccgcaga
new-gga-miRNA-25	uuugggugccagccccguugcgg	ggacggggcugggauccaggac	ggacggggcugggauccaggaccugcugcgggcauuggaaguguuuugggugccagccccguugcgg
new-gga-miRNA-26	gccgacgccggcgugcggcugc	agcugcgcaccggcggcgucgu	gccgacgccggcgugcggcugcuggacgugcagcugcgcaccggcggcgucgu
new-gga-miRNA-27	uuuccaaccuucgugauucuga	agaaucaugaagguugggaaaga	uuuccaaccuucgugauucugauucuucauuagggaucacagaaucaugaagguugggaaaga
new-gga-miRNA-28	cccgggguggcacgcuguac	cccagcacgccuaccccgggcc	cccgggguggcacgcuguacggcacgcagcggcugccccagcacgccuaccccgggcc
new-gga-miRNA-29	auggcuguucuagcaguagauc	ccuacugcuagguuugcccuu	ccuacugcuagguuugcccuuaaacuauggugcucucuuaauggcuguucuagcaguagauc
new-gga-miRNA-30	ucuugucugcuggggauggacaa	aaguccacugcauaugagaggug	aaguccacugcauaugagaggugaaaguaacaguauuucaucuugucugcuggggauggacaacu
new-gga-miRNA-31	ucugaugucccuuugccccgcag	augguacggggcuugcaguca	augguacggggcuugcagucauucugaugucccuuugccccgcag
new-gga-miRNA-32	cuggaccgcccgcuugguuaga	ugaucaagugguggugacagcu	ugaucaagugguggugacagcuauuucuaaaucugcuggaccgcccgcuugguuaga
new-gga-miRNA-33	ucugggggugcggagguacuga	gggccucccgcacgcuucuguc	gggccucccgcacgcuucugucggggaucgaaauagcgcgacgucugggggugcggagguacuga
new-gga-miRNA-34	cccgccgguggcgccgggc	cccgccggccgccgugcg	cccgccggccgccgugcgcauaaaacccgccgguggcgccgggc
new-gga-miRNA-35	ucauggugcucuucucuuccagg	cuggugagaagcgugcuguggag	cuggugagaagcgugcuguggagcgugguauggcaaagcuuggugcucauggugcucuucucuuccagg
new-gga-miRNA-36	gaucucccaaucugcgcgguugc	uaacugucguaaauggagagaga	gaucucccaaucugcgcgguugcauuugcuugaaguaguguaacugucguaaauggagagaga
new-gga-miRNA-37	uaguucagcugucugugaaugag	ucaucagagaggcugggcucugu	uaguucagcugucugugaaugagggcagagaccucaucagagaggcugggcucugu
new-gga-miRNA-38	gcagggcgaggagcugccccggu	agggcagcuccgcagccccuuccc	agggcagcuccgcagccccuucccccggcagcaccagggcagggcgaggagcugccccggu
new-gga-miRNA-39	uuuggcacuagcacauuuuugcu	caauuauguguagugccaauau	uuuggcacuagcacauuuuugcuuugcuuuauguguuuugagcaauuauguguagugccaauau
new-gga-miRNA-40	ccgggcagggccgagcugagc	cccggcccccgcccugccccgc	cccggcccccgcccugccccgccucacgugccgccgggcagggccgagcugagc
new-gga-miRNA-41	ccggagcccucgcuucucuugugc	cacagaggggcgcgggcccaac	ccggagcccucgcuucucuugugcucagaggcguagcacagaggggcgcgggcccaac
new-gga-miRNA-42	ugcgcugcgcucccugcacag	ccugcagggagcgcugcggccgu	ccugcagggagcgcugcggccgugcgcugagcccugcgcugcgcucccugcacag
new-gga-miRNA-43	aggcaguauugaaguggaugag	ucacugcuucucucucugcccag	aggcaguauugaaguggaugagggugccuguuucucacugcuucucucucugcccag
new-gga-miRNA-44	ugcacgaggaugugggcucauu	agagcacacacccagcuugcaca	agagcacacacccagcuugcacacggacacagcgugcacgaggaugugggcucauu
new-gga-miRNA-45	ucaaugagagcacggucugcc	cagacccugaaccucauugcca	ucaaugagagcacggucugccuuaugggacacgagcggaggcagacccugaaccucauugcca
new-gga-miRNA-46	guucagggcucagcacugggc	ucagugcugugcucagacccc	ucagugcugugcucagaccccgguguaggguuuaggguucagggcucagcacugggc
new-gga-miRNA-47	ugacaccgcugugcucugccccag	cugggggggcuaggggugucccu	cugggggggcuaggggugucccuuggugugugcuacgugugagugacaccgcugugcucugccccagg
new-gga-miRNA-48	cggacgaggcuucccgcgcugu	ccgcgcgggaagccucggccga	ccgcgcgggaagccucggccgacgagcaaccggcggacgaggcuucccgcgcugu
new-gga-miRNA-49	ucugucacugcuucuugcagg	cugugagaugcagaugaa	cugugagaugcagaugaagccacaggccugaguucaaaccucugucacugcuucuugcagg
new-gga-miRNA-50	ugcaccucgcgcugcgggccc	agccugcagccugcuaagcgcaug	agccugcagccugcuaagcgcauggugagaggccauuccaugcaccucgcgcugcgggccc
new-gga-miRNA-51	agcuuggcugcugagggacucu	agucccucuuucaaccaagcccu	agucccucuuucaaccaagcccuugugacaacaacagcuuggcugcugagggacucu
new-gga-miRNA-52	uuccgcgaaccgccuucgguc	ccaaagguggcucgcggaacuga	uuccgcgaaccgccuucggucgcggugucugcggagaccaaagguggcucgcggaacuga
new-gga-miRNA-53	ccccauugggaccccaaug	auugggaccccacugacccc	ccccauugggaccccaaugaccccauuugggaccccauagggaccccauagaucccauugggaccccacugacccc
new-gga-miRNA-54	guggagagcauuugacuga	ggaggugcgugugcaugugu	ggaggugcgugugcaugugugaggagauguggagagcauuugacuga
new-gga-miRNA-55	cucgggagggcaucgcgcggagg	cccgcgcagcccggccgcagcgc	cccgcgcagcccggccgcagcgcuuagucaccgcgcucgggagggcaucgcgcggagg
new-gga-miRNA-56	gacuccgguggaaugaaggac	gcuucacuccacugaaaucug	gcuucacuccacugaaaucugguuagguacgagacagacuccgguggaaugaaggac
new-gga-miRNA-57	ucucuauagggucucuaugggg	cuaugggguccccuaugggg	cuaugggguccccuaugggggcucuaugggcuuucuauggaucucuauggguucuaugggaucucuauggaucucuauggcuc

new-gga-miRNA-58	cggacccccguaccgcgacccc	gucgcggcucggggauccgccc	gucgcggcucggggauccgcccuguacagaguaggcgggcggacccccguaccgcgacccc
new-gga-miRNA-59	cgacacccccggugacagccgu	ggggucgccccgggucucggugu	cgacacccccggugacagccgugguccccucagaugcggggucgccccgggucucggugu
new-gga-miRNA-60	ccccgcgccgugcggugccggc	cggcgcugagcucggcguggcgg	ccccgcgccgugcggugccggcggugugugcggugccggcgcugagcucggcguggcggcu
new-gga-miRNA-61	uuucacucucagcaggcuccgc	ugccugcugugagugaaauugu	ugccugcugugagugaaauugucugagcuggugccgcacuuuaucaauuucacucucagcaggcuccgc
new-gga-miRNA-62	ccccgcgcugcucgugccccgc	agggccacguccgcgucgggcc	agggccacguccgcgucgggcccgagguggucaccgaccccgcgcugcucgugccccgc
new-gga-miRNA-63	ugcggaaccuggcccgcgcgcac	aagcgcgagcggguggcccgc	aagcgcgagcggguggcccgcaacgagcucaaccggcugcggaaccuggcccgcgcgcac
new-gga-miRNA-64	ugugcccacgcagacguacugc	cagaugucugcgugugccucugu	cagaugucugcgugugccucugugcauacauuuuacaugugcccacgcagacguacugc
new-gga-miRNA-65	ugugcugaggacuccacaucu	ugaggucuuaccagcacugagu	ugugcugaggacuccacaucuggacgcagugcuccaggugaggucuuaccagcacugagu
new-gga-miRNA-66	cagcgcugggcagugcacugc	agugcagucuguugcagcguggc	agugcagucuguugcagcguggcacaguacguugcucccugcagcgcugggcagugcacugc
new-gga-miRNA-67	ugccucuuugucuaucugcaga	gugcagguaggcagggggagcag	gugcagguaggcagggggagcaggcugugugcugguugccuaccgucugccucuuugucuaucugcaga
new-gga-miRNA-68	ugaacccccccgacuccguagg	ggcggggucugggggcucucagg	ggcggggucugggggcucucaggguuaggggagagccuugaugcagcccugaacccccccgacuccguagg
new-gga-miRNA-69	accauggcuguagacuguuac	uaacaaucuaaagccacggucg	accauggcuguagacuguuacccccccagcagcacaguaacaaucuaaagccacggucg
new-gga-miRNA-70	ccucccgcaccgccuguacgcc	cgugcgggcgcugcgggagccc	ccucccgcaccgccuguacgccgcgcacggggcggccguggcgcugcuggacgugcgggcgcugcgggagccc
new-gga-miRNA-71	cucccgcgggggcggccc	gccgcucagcgccgcgccg	cucccgcgggggcggccccuccgccgcucagcgccgcgccg
new-gga-miRNA-72	uugguggcugcaugcucucagc	ugagggcagagcugcagccuca	ugagggcagagcugcagccucacauuucuucuuuuguuggacuugguggcugcaugcucucagc
new-gga-miRNA-73	ucacagcccuuccgccccccu	uggggcugcagggcugggagau	uggggcugcagggcugggagauguggggcucugcagccccucgacaucacagcccuuccgccccccu
new-gga-miRNA-74	uucuccucccuucgcacggcgc	ugccggggcgcuggggaggggca	ugccggggcgcuggggaggggcagcucccccuuugcugcccugcuucuccucccuucgcacggcgc
new-gga-miRNA-75	ugcugugggcucugcucacc	ggugggcagcacccugggcaugg	ggugggcagcacccugggcauggcgcucugcccugcugugggcucugcucacc
new-gga-miRNA-76	cggaggucgggcagcuucagc	cgggagcucucggccggccgcgu	cggaggucgggcagcuucagcagcaguuuggugaagcgggagcucucggccggccgcgu
new-gga-miRNA-77	gcgcugggagucgucguguggc	ggcgcgacgaucccagggggcug	ggcgcgacgaucccagggggcuguugagcuccgugcagcgcugggagucgucguguggc
new-gga-miRNA-78	ccccgagcgaaccccggagggg	cgcuccggggucguacucaga	cgcuccggggucguacucagagcccuuggucagcacaagccccgagcgaaccccggagggg
new-gga-miRNA-79	ugcgcacacacgcggccugaag	gcugggacgcgcgugcgcgcac	gcugggacgcgcgugcgcgcacguuuguacgcucaggugugcgcacacacgcggccugaag
new-gga-miRNA-80	caccgcuucgccuucggccgc	agucgcaggcggagcgcugc	agucgcaggcggagcgcugcgccucaaacccaacgcaccgcuucgccuucggccgc
new-gga-miRNA-81	ccacucugugugccugcccacc	ggggaggccgcagugcagggc	ggggaggccgcagugcagggcaaagacaaacugccacucugugugccugcccacc
new-gga-miRNA-82	uggcagggaugacacacugagcug	aagcucaggcuguacuuuuucucc	uggcagggaugacacacugagcuggcugggucccauuuuuuccaucugaauugaagcucaggcuguacuuuuucuccacuccu
new-gga-miRNA-83	ucccuguaccgcugcgaccucu	acgccgucucgcugcaggggg	acgccgucucgcugcagggggcggcccgcgacaugcugagcucccuguaccgcugcgaccucu
new-gga-miRNA-84	ccgagcgagcucugcagagccu	cugucugcagggccugcuccaucc	cugucugcagggccugcuccaucccgugcugcaaggccgagcgagcucugcagagccu
new-gga-miRNA-85	ugcggaaggcgcaggcgucga	ugcgccucgcccuucuucacc	ugcgccucgcccuucuucacccagugccgguaggugcggaaggcgcaggcgucga
new-gga-miRNA-86	ccccagacccagggagucagg	ugacauccguguguuugguaaug	ugacauccguguguuugguaaugugcuguuugauuuccaccccagacccagggagucagg
new-gga-miRNA-87	uagggccaggcugggucacccac	agcuggcccaccucgccucccug	agcuggcccaccucgccucccugcugcccacccugcagguagggccaggcugggucacccac
new-gga-miRNA-88	ccgcugcccgucccccgagcugc	agcccguggugguacagcgcagcg	ccgcugcccgucccccgagcugccgucucccgcuugcagcccguggugguacagcgcagcgu
new-gga-miRNA-89	ucagccgucuugcagccuccu	auggcugugagugcggcugaggu	auggcugugagugcggcugaggugucagagcauccuccucagccgucuugcagccuccu
new-gga-miRNA-90	aaccaccucaguuguuucuacc	caggaacaacugaagugaauu	aaccaccucaguuguuucuacccuuucuguaaagcaggcaggaacaacugaagugaauu
new-gga-miRNA-91	acaucaacucuuacuguucggg	cggcaguaggagcucacaugc	cggcaguaggagcucacaugcaggcugcguucgccgcacaucaacucuuacuguucggg
new-gga-miRNA-92	gaagugucacacucacgggcucu	agccuguuggugaggccuucu	agccuguuggugaggccuucuuuugcuucaacuucaagaagugucacacucacgggcucu
new-gga-miRNA-93	ccgcgccgggccgagcccccgc	ggcggcuccuccugcgucaggug	ggcggcuccuccugcgucaggugcggcccucagagcuccgccccgcgccgggccgagcccccgc
new-gga-miRNA-94	caucuuggaguauaagguaccug	guaccugcaccccaagaugacu	caucuuggaguauaagguaccugauagagggugcagcagguaccugcaccccaagaugacu
new-gga-miRNA-95	cugaaacagucugugcaccccagg	cuggggcacaaacuguugugcu	cuggggcacaaacuguugugcuuucacagaccaagcugaaacagucugugcaccccagg
new-gga-miRNA-96	uaagcaugguauuucugugagg	ccucacagaaauaccaugcuua	ccucacagaaauaccaugcuuaugaaacaaguuccauuauauuuaagcaugguauuucugugagg
new-gga-miRNA-97	aaaccuauccaugacaggggau	cccucugccuugggcaggguugca	cccucugccuugggcaggguugcaaauguagugguuggcaaaccuauccaugacaggggau
new-gga-miRNA-98	ccagaacacugcgcaucgaccc	gucgaugcgcagugcugugguc	gucgaugcgcagugcuguggucauggauagaugagaaugaccagaacacugcgcaucgaccc
new-gga-miRNA-99	uauggcaaggaggcugcagacc	accugcgaucccugccuuuga	uauggcaaggaggcugcagaccauccucuccugccacuuggaccugcgaucccugccuuuga
new-gga-miRNA-100	ugcagcgcuuccucgugggguccu	cucucgggggccgcuguccgu	ugcagcgcuuccucgugggguccuuugaccuuucucgaggcucucgggggccgcuguccgu
new-gga-miRNA-101	cugagccuucugucccugcaga	cagcagggaugggggcagugc	cagcagggaugggggcagugccggcccguugugcugagccuucugucccugcaga
new-gga-miRNA-102	ccugggggcacgucggggucg	gaccccgcgcgaggccgccagcg	ccugggggcacgucggggucggaccgcuccgccuccccauccccgaccccgcgcgaggccgccagcg
new-gga-miRNA-103	uaugauucuacgauucccucag	gagggaaucauagaaucauau	gagggaaucauagaaucauaucauuguagaaucacagaguaugauucuacgauucccucag
new-gga-miRNA-104	agcggacaaaggacgauggcac	ugccaucguccuuuguccgcugc	agcggacaaaggacgauggcacacgaagugcucuggaggccugugacaguucaugugccaucguccuuuguccgcugc
new-gga-miRNA-105	cugugacaucucuguccccagc	uuggggaagggaggugaugggc	uuggggaagggaggugaugggcacacugcuggcagcugcugugacaucucuguccccagc
new-gga-miRNA-106	ugccugcuugcuccucacuugc	uaaaugaugaacaguuguggcau	uaaaugaugaacaguuguggcaugugaaaaaaguagcucaugccugcuugcuccucacuugc
new-gga-miRNA-107	ugcuuucgcuugcucugugggga	uugcggagcggcugacugagcgc	uugcggagcggcugacugagcgcacgcugcuuucugcgugcuuucgcuugcucugugggga
new-gga-miRNA-108	ccgggcugcgggcaccgccagc	ggggcgggugagggcccgggug	ccgggcugcgggcaccgccagcgucggacggcgcgggaagggggcgggugagggcccgggug
new-gga-miRNA-109	ucagagagcccccagccaaaug	uugggcugggggaucccucgc	ucagagagcccccagccaaaugugcagugcuguccguugggcugggggaucccucgc
new-gga-miRNA-110	auauguagcuuccuucuaucuccu	aagauagauguggccuaccaugc	aagauagauguggccuaccaugcuuaaccugaaugcauauguagcuuccuucuaucuccu
new-gga-miRNA-111	ucuggcuuagggcugcuccauc	cggcgcagagcccguugccguggg	cggcgcagagcccguugccgugggguguuucacacuccccucuggcuuagggcugcuccauc
new-gga-miRNA-112	cggccauugcuaggcgacgcugcc	cgcgccggcuguaauggcagca	cggccauugcuaggcgacgcugccgcggaugcucgcgccgcgccggcuguaauggcagca
new-gga-miRNA-113	ccgcgccggagcugcgggugccg	gucccccagacccgcgccgcc	gucccccagacccgcgccgccggggccaccacggagcggccgcgccggagcugcgggugccg
new-gga-miRNA-114	aaugcuuacuugucuggacaga	agcccagacaaguugaguauuu	agcccagacaaguugaguauuuauucuccuccugccaauggaauguguaaugcuuacuugucuggacaga
new-gga-miRNA-115	cugcucccucccggcugccugc	cgcagcacgcaggagagccgag	cgcagcacgcaggagagccgaggcacuauggaaacacucugcucccucccggcugccugc

new-gga-miRNA-116	acuccaccuccuguuugguuuug	auaccauucugagguggucugu	acuccaccuccuguuugguuuuguguuuuaugaaucauuugauaccauucugagguggucugu
new-gga-miRNA-117	aggucugaccacauauaucccu	ggacaugugagguuggaccuuu	aggucugaccacauauaucccuccuuauucuauacaggacaugugagguuggaccuuu
new-gga-miRNA-118	ucuggaauguuuauagugaggcu	ucuacccuauaaacauucugaug	ucuggaauguuuauagugaggcugagcuuuauaggucuacccuauaaacauucugaug
new-gga-miRNA-119	ucacgguggggaugguagaucug	ugaucccuguccccucccugca	ucacgguggggaugguagaucugcagcagauguugaucccuguccccucccugca
new-gga-miRNA-120	ucaagcauggagacagcagacacu	ugugcugcucuccaugccugaaa	ucaagcauggagacagcagacacuugucagcaguugugcugcucuccaugccugaaa
new-gga-miRNA-121	ucaagaacaaagcauguggccu	agcacugcacuguucucguagg	ucaagaacaaagcauguggccucauguuuugcagaaaaaagagcacugcacuguucucguagg
new-gga-miRNA-122	caacacugauacuuggcccugcag	cugggggugauggcagugg	cugggggugauggcagugguggccgugcucuuuggauggugcaacacugauacuuggcccugcag
new-gga-miRNA-123	agugcaaauuaugaauaaugga	auugauucauucuuugcucuucu	auugauucauucuuugcucuucuaauuauaagaggaagugcaaauuaugaauaaugga
new-gga-miRNA-124	ccaucuccaccagcagcacugu	cagugccgacgugugggggaugcg	cagugccgacgugugggggaugcgccguggggcagagcccaucuccaccagcagcacugu
new-gga-miRNA-125	ugucugggguccgcagugaugua	uacagcacugcuuucccacaac	ugucugggguccgcagugauguaguagaucagaguuugaccuuacagcacugcuuucccacaac
new-gga-miRNA-126	caccacgugucgcgauauaggc	agauaucgcgauaugugaggcgu	agauaucgcgauaugugaggcgucacaauaaucagcugucacacauugcaccacgugucgcgauauaggc
new-gga-miRNA-127	gugaggcggcggucgggacgcg	cggcccgccgcggcccucacgcc	gugaggcggcggucgggacgcgcggcaucuccgccccgcggcccgccgcggcccucacgcc
new-gga-miRNA-128	guggcgccgccgggcugggc	cggccggggggcgggcgg	cggccggggggcgggcggcgacgggacagggcgggaggagaccugagaaggaacuguggcgccgccgggcugggc
new-gga-miRNA-129	gucccucgccgcgcggucgggc	gcggcugcgccguguuggggccg	gcggcugcgccguguuggggccgggcccgcgcugccgucccucgccgcgcggucgggc
new-gga-miRNA-130	agcuggugggcaggcacacagagu	cugcggccuccccaccauuugc	agcuggugggcaggcacacagaguggcaguuugucuuugcccugcacugcggccuccccaccauuugc
new-gga-miRNA-131	uacggcgacagggaagaaguaa	ucugcuuugcuguccccgcucc	uacggcgacagggaagaaguaagugcgaagggcuuucugcuuugcuguccccgcucc
new-gga-miRNA-132	ucgugaccuucucuuuguauu	aucaaagugaaaggucaagauu	aucaaagugaaaggucaagauuuuaagucaugguugaucgugaccuucucuuuguauu
new-gga-miRNA-133	cacucggcccagcucuggugu	cccagagccauggcccagggca	cccagagccauggcccagggcaggagcagcuaucgugcacucggcccagcucuggugu
new-gga-miRNA-134	cauuggguugcacguacuuagu	uaaguacguugaacagccuaagug	uaaguacguugaacagccuaagugcugccuguuagcagucauuggguugcacguacuuagu
new-gga-miRNA-135	cgggcgggagguccggcg	cgggcgggcgggcggucg	cgggcgggcgggcggucggcagggggcgcgggcuuuagugugacgagugcugugcggggcgacgggcgggagguccggcg
new-gga-miRNA-136	cugccugaccuggauguacagcu	agagacaccagcucaggcuugg	cugccugaccuggauguacagcucugcuguggaccccaucagagacaccagcucaggcuugg
new-gga-miRNA-137	cccuaggagacguuggccgaac	ccggcaccaacgucacuggucccc	ccggcaccaacgucacuggucccauugucagcuguuccuggcccuaggagacguuggccgaac
new-gga-miRNA-138	uguuccugcugaacugagccag	auaucagcucaguaggcaccgga	uguuccugcugaacugagccaguauguaaaaucagaacugauaucagcucaguaggcaccgga
new-gga-miRNA-139	guaagugccuccggguguggggg	ccgcacccugagaugcucacagg	guaagugccuccgggugugggggauggcgucggcugccccgcacccugagaugcucacagg
new-gga-miRNA-140	ggggugcaccagugucauuggc	ucagugacaccugugcaaucc	ucagugacaccugugcaauccaccugaaggcucuggggugcaccagugucauuggc
new-gga-miRNA-141	ucaggaggugcugcacaugacu	ucuuuuguguggcuuaccuuga	ucuuuuguguggcuuaccuugagggagauagucaucucaggaggugcugcacaugacu
new-gga-miRNA-142	aucaccaaggcugggugcuguga	ggagcagccagcuuugggauggg	aucaccaaggcugggugcugugagagccacaugcagaucggagcagccagcuuugggaugggc
new-gga-miRNA-143	accuggcugagggcugcacugc	acugcagcccucugccagacu	acugcagcccucugccagacugcgggguguaaccuggcugagggcugcacugc
new-gga-miRNA-144	ucagccugggcacggccccaagu	gcagggcugugcgccaggcuucag	gcagggcugugcgccaggcuucaggucagugaguccauggucagccugggcacggccccaagu
new-gga-miRNA-145	agccagcccagcucugcucagc	agagcagagcuggagcugauugc	agagcagagcuggagcugauugcuuuugcagccagcccagcucugcucagc
new-gga-miRNA-146	ggcugagcugcggcgcuuccu	gaagcaccgcugcucuuccug	ggcugagcugcggcgcuuccuucugcuuuguguacagggaagcaccgcugcucuuccug
new-gga-miRNA-147	uuguagcaagcuggcucugacu	ucagagccagcuuguuacaagc	ucagagccagcuuguuacaagcuuguagccaguuuguagcaagcuggcucugacu
new-gga-miRNA-148	uacugcgugcuguccucacggc	uuugagcacagcacugcuggcgc	uacugcgugcuguccucacggccguuauuccaacagggcuuugagcacagcacugcuggcgc
new-gga-miRNA-149	aggcagagcucugguggcacag	cagccacuggagcuccuccugu	cagccacuggagcuccuccuguccugauggaaugaauggcacucaggcagagcucugguggcacag
new-gga-miRNA-150	cugaugugugagcgcugugcacu	ugcccgugccucacgcucaagg	cugaugugugagcgcugugcacuggauccuauggaagugcccgugccucacgcucaagg
new-gga-miRNA-151	accgccccggcccccgggcugc	ugcucggggaucggggcagugc	ugcucggggaucggggcagugcuccgggaucauucuguaccgccccggcccccgggcugc
new-gga-miRNA-152	ucagcugcucugagccccagc	uggggcugaggcuuaguguggag	uggggcugaggcuuaguguggagcugugcuuacuccugcugcucagcugcucugagccccagc
new-gga-miRNA-153	cuucccaggcauguuuggaguc	acuaccaacguacuugggaauuu	acuaccaacguacuugggaauuuuuauucauuuacuaaagcuucccaggcauguuuggaguc
new-gga-miRNA-154	uauggggcugccaguggggccgg	ccccccuuccagcccuauaaa	uauggggcugccaguggggccgggaugcugggaccgcucccccccuuccagcccuauaaa
new-gga-miRNA-155	agccugcugagagugaaauuga	aauuucacucacagcaggcacuu	agccugcugagagugaaauugauaaagugcggcaccagcucagacaauuucacucacagcaggcacuu
new-gga-miRNA-156	aaaggcgcagugcugcagugcu	ugcugcagagcuguagaccugcu	ugcugcagagcuguagaccugcugggaaucuugugccagaaaggcgcagugcugcagugcu
new-gga-miRNA-157	cugcccgcggcgcccggccgcg	cggccggucgccgcgggcaac	cugcccgcggcgcccggccgcggcucaggcgcggccggucgccgcgggcaac
new-gga-miRNA-158	agaagagcauuuaaaugugugc	acacauuucagugcuucccugaa	agaagagcauuuaaaugugugcuuacaaacgaaacacacauuucagugcuucccugaa
new-gga-miRNA-159	uacagccggagcugagggugc	ugccugcaccgccggcugugc	ugccugcaccgccggcugugcuuuggcuguggccuauuuugguacagccggagcugagggugc
new-gga-miRNA-160	caggggaguuggggugccucagc	ucaccggcccuccaaucccuagc	caggggaguuggggugccucagcacaugacccccugcucaccggcccuccaauccccuagc
new-gga-miRNA-161	uuccuuaccuucuuugcaguacuc	uacuguggagaugguguauaaau	uuccuuaccuucuuugcaguacuccagucuauuaaguacuguggagaugguguauaaau
new-gga-miRNA-162	cccccaugguccauggauggau	cccccaugguccaugggauggcu	cccccaugguccauggauggaucguuccauggcuguaucccccaugguccaugggauggcu
new-gga-miRNA-163	cgccgcuuccuuaccguuaaccu	gugcgggccggaggcgcugccc	gugcgggccggaggcgcugcccguugucugcgaggggcgccgcuuccuuaccguuaaccu
new-gga-miRNA-164	cuacugcagugcuggaugcccugg	agggccccugcagagcagugg	cuacugcagugcuggaugcccugggguacuuggcucauccagggccccugcagagcagugg
new-gga-miRNA-165	uagggagaaaaugugaagcagcu	cugcuacucuuuuccuccuaga	uagggagaaaaugugaagcagcuuggagcagcaguguaaaacuaauacuucuccugcuacucuuuuccuccuaga
new-gga-miRNA-166	gaagagguccaggcugagccac	agcuuagccugugucucuguugc	agcuuagccugugucucuguugcugcauuuggacaugaagagguccaggcugagccac
new-gga-miRNA-167	uuggguggacgguuggacuuga	uuuuccaaccuucuucccccccu	uuggguggacgguuggacuugagguuguagugcucuuuuccaaccuucuucccccccu
new-gga-miRNA-168	uuugucccucccauuguuucucc	agacacauggacgcaaggacaaag	agacacauggacgcaaggacaaagagacauuuggaccccuuugucccucccauuguuucucc
new-gga-miRNA-169	cacuguauggugcuguacgcaug	ggggugcagcaccauacagugug	cacuguauggugcuguacgcaugggcaggagaagggcaugggggugcagcaccauacagugug
new-gga-miRNA-170	cgcgggccgcgcgcucaccgc	gugggugcucgcggcccgcgcc	gugggugcucgcggcccgcgcccucccccgccccgaggggucgcgggccgcgcgcucaccgc
new-gga-miRNA-171	acugaugacagagcauugcccu	agcugugcucuguuccucaguu	agcugugcucuguuccucaguucuggaaguuuuaaucgcugcaauguacugaugacagagcauugcccu
new-gga-miRNA-172	cuggggagaaugucugccuuucu	agugggcaucauucucccuucc	cuggggagaaugucugccuuucugauaugccgugauugcagugggcaucauucucccuucc
new-gga-miRNA-173	aagcugcugcccguguucaggug	ccugcccacgugcgcggcuccc	aagcugcugcccguguucaggugcucucugcacacacgugugguuugcacgccugcccacgugcgcggcuccc

new-gga-miRNA-174	uccagcauuaacgcaggugacugc	agcaccuccuuagugcugggaa	uccagcauuaacgcaggugacugcacguggaaucccugcagcaccuccuuagugcugggaa
new-gga-miRNA-175	uaggaacuuucccugggaggu	ccucacagcuauuggaaguuccc	uaggaacuuucccugggaggugucauuuucuuuuuaccucacagcuauuggaaguuccc
new-gga-miRNA-176	caggccgcgugugugcgcaca	ugcgcgcacgcgcgucccagc	caggccgcgugugugcgcacaccugagcguacaaacgugcgcgcacgcgcgucccagc
new-gga-miRNA-177	ccagcugucgcugcuguccggg	ugggcugcagagccaagcagcuga	ugggcugcagagccaagcagcugaugggugagaacagcccccagcugucgcugcuguccggg
new-gga-miRNA-178	ucucacugcuccucuuuccucaga	ggggguguggggagcauggagg	ggggguguggggagcauggagggcucagggcugcacagcuccucucacugcuccucuuuccucaga
new-gga-miRNA-179	uacaucacgugggugugggcguc	cgucauaugucacgugggggugug	uacaucacgugggugugggcgucguaugucacgcgggugugacgucauaugucacgugggggugug
new-gga-miRNA-180	uugggcugggcuugcugcgugg	acgcugcugacccagcccacgu	acgcugcugacccagcccacgucccaccccuuugcuuuggguuugggcugggcuugcugcgugg
new-gga-miRNA-181	uucucagguuagacaguccucg	caugggcugacgaaccugagauu	caugggcugacgaaccugagauugacccucauguuucaccauucucagguuagacaguccucg
new-gga-miRNA-182	cugaggcgcucccugcauccc	cagggagcgccucagcagccc	cugaggcgcucccugcaucccugcuugcgggaucccugcgggaugcagggagcgccucagcagccc
new-gga-miRNA-183	cgcuccgcgcccgcaggaccgu	uccgcgggugcggagcggcuca	cgcuccgcgcccgcaggaccgucagagaagcgguuccgcgggugcggagcggcuca
new-gga-miRNA-184	cacgccgggaugcagcgaggcg	cucgcugcaucccggcguaccc	cacgccgggaugcagcgaggcgcuuccgccucgcugcaucccggcguaccc
new-gga-miRNA-185	cugcguaccguguggugauguca	gacaucaccagcggggcggcacu	gacaucaccagcggggcggcacucauagccacugggggagugcugcguaccguguggugaugucacu
new-gga-miRNA-186	ucagcaccccuccguccccgcagu	cgggggcgcggggugcugagc	cgggggcgcggggugcugagccguccuuuggggucucuccccgcgugggccucccccgcucagcaccccuccguccccgcagu
new-gga-miRNA-187	aacgaaaucccagggcagcugg	ugcuuuugggauuccguugccc	aacgaaaucccagggcagcugggcgcugcggggacagcugcuuuugggauuccguugccc
new-gga-miRNA-188	guccaguuuucccaggaaucccu	auuccuggaaauacuguucuu	guccaguuuucccaggaaucccuuaggcgcuacguuggggauuccuggaaauacuguucuu
new-gga-miRNA-189	augugcguuagucaguggcuca	uuagccgcugucacacgcaca	uuagccgcugucacacgcacaguggagucgccgagagcacaaugugcguuagucaguggcuca
new-gga-miRNA-190	cucaugcccucucugugccagg	ucugggcacaaagagggcagugg	ucugggcacaaagagggcagugggagcagggagagccucaugcccucucugugccagg
new-gga-miRNA-191	ugcgcuuucucaucccggcagg	uugggaugggauggugcugcac	uugggaugggauggugcugcacugcccagcucugauggcgcugcgcuuucucaucccggcagg
new-gga-miRNA-192	cugcuggagcugcuccugguugc	cuggagcugcuccugguugcacu	cuggagcugcuccugguugcacucagucugcuccuggugcugcuggagcugcuccugguugc
new-gga-miRNA-193	uugcagaccaccgaugagaac	uuucucaucgguggucugcucaga	uugcagaccaccgaugagaacggaaaagauucccuuuucucaucgguggucugcucaga
new-gga-miRNA-194	ucuggcugcucagugcucugcu	agcaggagcacugggugacgagga	agcaggagcacugggugacgaggagccucgcacagcucuggcugcucagugcucugcu
new-gga-miRNA-195	uuucggcuguuuccggcgacugc	uuccggaaaucgccgaagcgag	uuccggaaaucgccgaagcgagccgaacgcgcugccauuucuguuucggcuguuuccggcgacugc
new-gga-miRNA-196	uguacugagaacuccucaucugg	ugaggucucaccagugcagagc	uguacugagaacuccucaucuggaugcaguacgccaggugaggucucaccagugcagagc
new-gga-miRNA-197	cacagcgcaucgcugggggcuga	gcaucccggccaugaagcucac	gcaucccggccaugaagcucaccuggcugagugcccacagcgcaucgcugggggcuga
new-gga-miRNA-198	ucgguacuggucgguacuggua	guacugguuuguacuggucugu	guacugguuuguacuggucuguacugguuuaugcuggucgguacuggucgguacuggua
new-gga-miRNA-199	cacacacauguauauauaca	uauauauaugugcauguguguau	uauauauaugugcauguguguauauguauacauguauauacacacacauguauauauaca
new-gga-miRNA-200	uuuagugugauaauggcguuug	aacaccauugucacacuccaua	uuuagugugauaauggcguuugauagauuggacacaaacaccauugucacacuccaua
new-gga-miRNA-201	cauaguaggaaaaggaaguaucu	uuccuuuuccuacucuuguaga	uuccuuuuccuacucuuguagauuuucacuguaaucuacauaguaggaaaaggaaguaucu
new-gga-miRNA-202	aggugcacaggggccaucugacu	cucccugaugacaaaacu	cucccugaugacaaaacuaggugcacaggggccaucugacu
new-gga-miRNA-203	ucgcacugcucguaaccuuuugac	ugaauagguuccauuccag	ucgcacugcucguaaccuuuugacagcauauuccugugaauagguuccauuccag

precursor coordinate

chr13:8493108..8493164:-

chr12:11670979..11671039:+

chr12:11671363..11671426:+

chr33:1093264..1093324:+

chr1:843242..843306:-

chr20:9710469..9710531:+

chrUn_NT_467319v1:10243..10320:-

chr19:5515026..5515081:+

chr33:1171914..1171974:+

chr5:15887078..15887137:+

chr18:9383535..9383595:+

chr1_NT_456790v1_random:3306..3391:+

chr19:5515770..5515834:-

chr19:5515202..5515261:-

chr5:21516911..21516976:+

chr1_NT_456305v1_random:9360..9444:+

chrW_NT_463533v1_random:23..79:-

chr8:4658341..4658400:-

chrUn_NT_474180v1:2343..2406:+

chr2:412762..412823:+

chr4:63482790..63482853:-

chr1_NT_456560v1_random:2532..2621:-

chrZ:16702090..16702149:-

chr28:662516..662568:+

chr5:85802..85869:+

chrUn_NT_474236v1:2804..2857:+

chr4:89099769..89099832:-

chr22_NT_463025v1_random:5999..6058:-

chr3:87380940..87381002:+

chr3:45733506..45733571:+

chr15:10903030..10903076:+

chr4:35126567..35126624:-

chr2:32178060..32178126:-

chr15:9383953..9383997:-

chr12:3154776..3154845:+

chr14:6179802..6179865:-

chr13:17162740..17162796:-

chr4:58156674..58156735:-

chr1:844265..844329:-

chr1:72122792..72122846:+

chr10:776513..776571:-

chr19:86485..86540:+

chr2_NT_457899v1_random:8716..8773:+

chr24:4442640..4442696:-

chr23:1881170..1881233:-

chr11:17925070..17925127:-

chr15:28362..28430:-

chr3:81811344..81811399:+

chr5:14017742..14017803:-

chr4:50090806..50090867:-

chr10:14678610..14678667:-

chr4:39800704..39800764:-

chrUn_NT_479370v1:1076..1152:-

chrZ:43054244..43054291:-

chr3:87201449..87201507:-

chr26:3076089..3076146:-

chrUn_NT_474824v1:4149..4250:-

chr2:31320562..31320623:+
chr33:1576506..1576565:-
chr18:6449723..6449784:+
chr1:144520190..144520259:+
chr3:110315775..110315834:+
chr2:115487968..115488028:+
chr21:3969023..3969082:-
chr11:18806839..18806899:+
chr3:105077421..105077483:-
chr3:30349125..30349194:+
chrUn_NT_464587v1:10306..10377:-
chr19:5515205..5515264:+
chr9:4696186..4696259:+
chr4:43046174..43046215:+
chr19:7378734..7378798:+
chrUn_NT_464201v1:17930..17997:+
chr8:28362266..28362331:-
chr4:5200274..5200327:+
chr27:4778500..4778559:+
chr25:2556624..2556683:+
chr2:62161165..62161226:+
chrUn_NT_477504v1:1263..1324:-
chr20:11274922..11274978:-
chr10:17093538..17093593:+
chr4:60454305..60454388:-
chr9:10684842..10684905:+
chr8:7496343..7496401:+
chr19:4883279..4883334:+
chr13:13593415..13593475:+
chr21:621901..621963:-
chr4:25598798..25598859:+
chr3:96912434..96912493:-
chr8:24660322..24660381:+
chr19:5515021..5515080:-
chrZ:43198296..43198356:+
chrUn_NT_471281v1:4376..4440:-
chr2:145259936..145259997:-
chr15:10062034..10062093:-
chr2:60473961..60474026:-
chr2:147405012..147405073:-
chr18:2872432..2872494:+
chr25:1578295..1578357:-
chr24:290173..290233:+
chr15:10874914..10874969:+
chr3:104943602..104943669:+
chr2:115940207..115940268:-
chr12:5317530..5317608:+
chr15:7128431..7128491:+
chr1:96374526..96374588:+
chr2:111558033..111558093:-
chr19:4311319..4311381:-
chr13:2583452..2583509:+
chr4:81134340..81134400:-
chr10:19224975..19225037:-
chr2:44942265..44942325:-
chr12:11670237..11670299:+
chr28:3936705..3936775:+
chr1:142053..142113:+

chr4:275566..275629:-

chr22:2664364..2664422:+

chr19:6035263..6035321:+

chr4:90158100..90158155:-

chr13:14454465..14454522:-

chr28:1588180..1588243:-

chr21:580202..580267:+

chr19:8077899..8077957:+

chr17:1481029..1481088:+

chr5:18631105..18631169:-

chrUn_NT_476753v1:1940..2010:+

chr15:743032..743093:+

chrZ:18071582..18071657:+

chr19:9118371..9118429:+

chr10:17093528..17093596:-

chr3:59496755..59496812:-

chr1:50452618..50452677:-

chr24:4180002..4180060:+

chr4:60872218..60872279:-

chr1_NT_456372v1_random:327..407:+

chr4:292851..292913:+

chrZ:1191599..1191662:+

chrZ:41649935..41649998:-

chrUn_NT_468272v1:5616..5676:+

chr4:74238850..74238906:+

chr1:62218231..62218290:-

chr18:9969045..9969109:-

chr19:7286198..7286251:-

chr11:17833758..17833821:-

chr14:4515271..4515322:+

chr4:3569339..3569398:-

chr1:177088318..177088373:-

chr12:2189087..2189149:-

chr3:109826725..109826791:+

chr1:120628360..120628419:-

chr3:30865630..30865690:+

chr12:9880189..9880252:+

chr4:63482793..63482855:+

chrUn_NT_465998v1:7388..7449:-

chr1:144520186..144520254:-

chr20:8554637..8554698:+

chr4:77944504..77944556:+

chr1:168016562..168016620:-

chr4:1044199..1044262:-

chr20:10257577..10257638:+

chr3:26170237..26170295:-

chr27:4527663..4527725:-

chr3:51316645..51316705:+

chr12:19390016..19390077:-

chr3:42183113..42183188:+

chr10:15334895..15334953:-

chr21:5006615..5006673:-

chr28:29061..29123:-

chr20:9815103..9815165:-

chr5:11706170..11706232:-

chrZ:78517883..78517952:-

chr13:8512966..8513027:-

chr28:3078580..3078652:+

chr23:5238151..5238212:-

chr1:22959266..22959325:-

chrUn_NT_477504v1:1267..1325:+

chr17:7275426..7275489:-

chr27:5284831..5284897:-

chrUn_NT_469680v1:596..660:+

chr2:926178..926242:-

chr20:9710466..9710529:-

chr18:3536305..3536371:+

chr5:17593195..17593251:+

chrUn_NT_465151v1:2038..2089:-

chrUn_NT_472355v1:309..377:+

chr10:1255074..1255158:+

chr12:11670975..11671035:-

chr13:8492496..8492557:-

chr5:15887075..15887138:-

chr10:20135923..20135981:-

chr23:1281695..1281758:+

chr27:4542764..4542826:-

chr10:14704761..14704820:-

chr32:15532..15590:+

chr2:32251637..32251703:-

chr27:512934..512994:+

chr27:5200919..5200977:-

chrUn_NT_472922v1:3667..3726:+

chr11:7747549..7747609:+

chrW:2525199..2525257:+

chrZ:40033855..40033916:-

chr5:23428240..23428281:+

chr5:13119888..13119943:+

new_name	consensus mature sequence	consensus star sequence	consensus precursor sequence
new-gga-miRNA-1	ugagaugaagcacuguagcucg	ggugcagugcugcaucucugguc	ggugcagugcugcaucucuggucaauugggagucugagaugaagcacuguagcucg
new-gga-miRNA-2	caacggaaucccaaaagcagcug	cugcccugggauuucguuacc	caacggaaucccaaaagcagcugucccccgcagcgcccagcugcccugggauuucguuacc
new-gga-miRNA-3	aaugacacgaucacucccgcugagc	caucggggaugucgugucugucc	aaugacacgaucacucccgcugagcgcgcagccacagagccaucggggaugucgugucugucc
new-gga-miRNA-4	aggacugucuaaccugagaau	aucucagguucgucagcccauga	aggacugucuaaccugagaauggugaaacaugagggucaaucucagguucgucagcccauga
new-gga-miRNA-5	uacccuguagacucgaauuugu	caaauucgucucuaggggaau	uacccuguagacucgaauuugugugagcgucuccgagucgcaaauucgucucuaggggaau
new-gga-miRNA-6	aacaguaagaguugaugugcgg	ugcaugugagcuccuacugccg	aacaguaagaguugaugugcggcgaacgcagccugcaugugagcuccuacugccg
new-gga-miRNA-7	accguggcuuuagauuguuacu	uaacagucuacagccauggucg	accguggcuuuagauuguuacugugcugcugggggguaacagucuacagccauggucg
new-gga-miRNA-8	uccagcaucagugauuuuguug	aacaauauccuggugcugagu	aacaauauccuggugcugagugaguugcacacagagacuccagcaucagugauuuuguug
new-gga-miRNA-9	uaacagucuacagucauggcu	accuuggcucuagacugcuuacu	accuuggcucuagacugcuuacugcugcguagggacaggacaguaacagucuacagucauggcu
new-gga-miRNA-10	ugagguagucgguuguauuguu	cgaugcagccgacuacuuuccc	ugagguagucgguuguauuguugggggccgggauggggccccacacucgagguaacgaugcagccgacuacuuuccc
new-gga-miRNA-11	ucagugcaucacagaacuuggu	gagguucuguccuacacuccggcu	gagguucuguccuacacuccggcuguggcuaugggcagucagugcaucacagaacuuggu
new-gga-miRNA-12	agccacugacuaacgcacauug	cugugcgugugacagcggcu	agccacugacuaacgcacauugugcucucggcgacuccacugugcgugugacagcggcu
new-gga-miRNA-13	cuuuuugcggucugggcuugc	aagcccuuaccccaaaaagcau	cuuuuugcggucugggcuugcuguacauaacuaccuacccgggaagcccuuaccccaaaaagcau
new-gga-miRNA-14	aggcaguguauuguuaguuagcu	gcaacuaacuacacugccaca	aggcaguguauuguuaguuagcugcucuucauacaccagcaacuaacuacacugccaca
new-gga-miRNA-18	ccucgacacaagcuuuuguc	gaccugauucugggucggggu	gaccugauucugggucgggguucguacguagcagagcagcucccucgcugcgaucuauugagaguagcccucgacacaagcuuuu
new-gga-miRNA-20	gaugaaaucccccuauggccugu	aggauagguggguuucauugaau	gaugaaaucccccuauggccuguuugugaugguaccgcaggauagguggguuucauugaau
new-gga-miRNA-21	guucagggcucagcacugggc	ucagugcugugcucagacccc	ucagugcugugcucagaccccgguguaggguuuaggguucagggcucagcacugggc
new-gga-miRNA-22	cacagcaaguguagacaggca	ccugucugugccugcuguacagg	ccugucugugccugcuguacaggugagaggauguucugcacagcaaguguagacaggca
new-gga-miRNA-23	uucccaaguacguugguaguca	acuccaaacaugccugggaagcuu	acuccaaacaugccugggaagcuuuaguaaaugaauaaaaauucccaaguacguugguaguca
new-gga-miRNA-25	cccccaucgauuugcucuacg	aagggcaaacccgaugggcacc	cccccaucgauuugcucuacgaacugaugaagcagaagggcaaacccgaugggcacc
new-gga-miRNA-27	acaucaacucuuacuguucggg	cggcaguaggagcucacaugc	cggcaguaggagcucacaugcaggcugcguucgccgcacaucaacucuuacuguucggg
new-gga-miRNA-28	auggcuguucuagcaguagauc	ccuacugcuagguuugcccuua	ccuacugcuagguuugcccuuaaacuauggugcucucuuaauggcuguucuagcaguagauc
new-gga-miRNA-29	agagaaucauagaauggccug	ggaccacaaugaucaucaagu	agagaaucauagaauggccuguguugaaaaggaccacaaugaucaucaagu
new-gga-miRNA-31	aagcagaggauauggaacugucu	agagauuccacauccucugauga	agagauuccacauccucugaugagcaagauucaaggucuaccacuggugaagaagcagaggauauggaacugucu
new-gga-miRNA-32	gccgacgccggcgugcggcugc	agcugcgcaccggcggcgucgu	gccgacgccggcgugcggcugcuggacgugcagcugcgcaccggcggcgucgu
new-gga-miRNA-33	accauggcuguagacuguuac	uaacaaucuaaagccacggucg	accauggcuguagacuguuaccccccagcagcacaguaacaaucuaaagccacggucg
new-gga-miRNA-34	uuguggcaaagaacaucaggggaga	cgaccccugggauucuggagu	uuguggcaaagaacaucaggggagacccgaggccgaccccugggauucuggagu
new-gga-miRNA-35	uuugggugccagccccguugcgg	ggacggggcugggauccaggac	ggacggggcugggauccaggaccugcugcgggcauuggaaguguuuugggugccagccccguugcgg
new-gga-miRNA-36	uuuggcacuagcacauuuuugcu	caauuauguguagugccaauau	uuuggcacuagcacauuuuugcuuugcuuuauguguuuugagcaauuauguguagugccaauau
new-gga-miRNA-37	ugauauguuugauauuagguug	acuaaauaucaaacauauucu	ugauauguuugauauuagguuguuugauuggaaaccaacuaaauaucaaacauauucu
new-gga-miRNA-38	cacugccggcucggcgcccccgc	ccgggcggccgagccgaggucg	ccgggcggccgagccgaggucguugcacugccggcucggcgcccccgc
new-gga-miRNA-39	gaucucccaaucugcgcgguugc	uaacugucguaaauggagagaga	gaucucccaaucugcgcgguugcauuugcuugaaguaguguaacugucguaaauggagagaga
new-gga-miRNA-40	uuccgcgaaccgccuucgguc	ccaaagguggcucgcggaacuga	uuccgcgaaccgccuucggucgcggugucugcggagaccaaagguggcucgcggaacuga
new-gga-miRNA-41	uugggcuguguuuccgccccuggg	ccagguauacugaacaacccaccag	uugggcuguguuuccgccccuggggcaaacaguuccagguauacugaacaacccaccaggu
new-gga-miRNA-42	cccugcucugugacagcgcucc	agcugcugucugugugcagaug	agcugcugucugugugcagaugagaugaaaggagcucccugcucugugacagcgcucc
new-gga-miRNA-43	cgacacccccggugacagccgu	ggggucgccccgggucucggugu	cgacacccccggugacagccguggucccucagaugcggggucgccccgggucucggugu
new-gga-miRNA-44	ucauggugcucuucucuuccagg	uggugagaagcgugcuguggagc	uggugagaagcgugcuguggagcgugguauggcaaagcuuggugcucauggugcucuucucuuccagg
new-gga-miRNA-45	gacuccgguggaaugaaggaca	gcuucacuccacugaaaucug	gcuucacuccacugaaaucugguuagguacgagacagacuccgguggaaugaaggaca
new-gga-miRNA-46	ucaucagagaggcugggcucugu	uaguucagcugucugugaaugag	uaguucagcugucugugaaugagggcagagaccucaucagagaggcugggcucugu
new-gga-miRNA-47	acacgcgugugguuguccccagg	uuggggacacgcgugugacacg	uuggggacacgcgugugacacgcacggggacacgugugacacgcgugugacacguugcugacacgcgugugguuguccccagg
new-gga-miRNA-49	ccccgcgccgugcggugccggc	cggcgcugagcucggcguggcggc	ccccgcgccgugcggugccggcggugugugcggugccggcgcugagcucggcguggcggcu
new-gga-miRNA-50	agaagagcauuuaaaugugugc	acacauuucagugcuucccugaa	agaagagcauuuaaaugugugcuuacaaacgaaacacacauuucagugcuucccugaa
new-gga-miRNA-51	auuucacucucagcaggcucc	ugccugcugugagugaaauugu	ugccugcugugagugaaauugucugagcuggugccgcacuuuaucaauuucacucucagcaggcucc
new-gga-miRNA-52	auggcugugagugcggcugagg	ucagccgucuugcagccuccu	auggcugugagugcggcugaggugucagagcauccuccucagccgucuugcagccuccu
new-gga-miRNA-53	ccggagcccucgcuucucuugugcu	cacagaggggcgcgggcccaac	ccggagcccucgcuucucuugugcucagaggcguagcacagaggggcgcgggcccaac
new-gga-miRNA-55	ugcgcacacacgcggccugaag	gcugggacgcgcgugcgcgcac	gcugggacgcgcgugcgcgcacguuuguacgcucaggugugcgcacacacgcggccugaag
new-gga-miRNA-56	ggccauguugucugugggcucu	acccacaaacaacauggcggcu	acccacaaacaacauggcggcugugguuacaggcggccauguugucugugggcucu
new-gga-miRNA-57	cccgggguggg cacgcuguacgg	cccagcacgccuaccccgggcc	cccgggguggg cacgcuguacggcacgcagcggcugccccagcacgccuaccccgggcc
new-gga-miRNA-59	ucaaugagagcacggucugcc	cagacccugaaccucauugcca	ucaaugagagcacggucugccuuaugggacacgagcggaggcagacccugaaccucauugcca
new-gga-miRNA-61	ccccgcgcugcucgugccccgc	agggccacguccgcgucgggcc	agggccacguccgcgucgggcccgagguggucaccgaccccgcgcugcucgugccccgc
new-gga-miRNA-62	cggacgaggcuucccgcgcugu	ccgcgcgggaagccucggccga	ccgcgcgggaagccucggccgacgagcaaccggcggacgaggcuucccgcgcugu
new-gga-miRNA-63	cacuguaugggugcuguacgcaug	ggggugcagcaccauacaguguga	cacuguauggugcuguacgcaugggcaggagaagggcauggggugcagcaccauacaguguga
new-gga-miRNA-64	uugggcugcguuuccaccccuggg	ccagguauacugaacaacccaccac	uugggcugcguuuccaccccuggggcaaacaguuccagguauacugaacaacccaccacgug
new-gga-miRNA-65	ugcugugggcucugcucacc	gugggcagcacccugggcaugg	gugggcagcacccugggcauggcgcucugcccugcugugggcucugcucacc
new-gga-miRNA-66	cugaugugugagcgcugugcacu	ugcccgugccucacgcucaagg	cugaugugugagcgcugugcacuggauccuauggaagugcccgugccucacgcucaagg
new-gga-miRNA-69	ccuuaucaguucuccguccaac	ugggcuggaaaagugauaagggg	ccuuaucaguucuccguccaacagucagagcgaagaagcugggcuggaaaagugauaagggg
new-gga-miRNA-70	cugcugggaauacugaguaagg	uugcucaguauugcagacagcu	uugcucaguauugcagacagcuguuacugucucuagcugcugggaauacugaguaagg

new-gga-miRNA-72	aggcgggcuggaugucaacagc	cguugacagccaggacgacccugc	cguugacagccaggacgacccugccgcaguacagccccaggcaggcgggcuggaugucaacagc
new-gga-miRNA-76	agugcaaauuaugaauaaugga	cauugauucauucuuugcucu	cauugauucauucuuugcucuucuaauuauaagaggaagugcaaauuaugaauaaugga
new-gga-miRNA-77	aaccaccucaguuguuucuacc	caggaacaacugaagugaauu	aaccaccucaguuguuucuacccuuucuguaaagcaggcaggaacaacugaagugaauu
new-gga-miRNA-78	uucagggugcugcagagagc	ucccugcuguucccugggagu	ucccugcuguucccugggagugcacgacccccuuucagggugcugcagagagc
new-gga-miRNA-79	ucgugaccuucucuuuguauu	aucaaagugaaaggucaagauu	aucaaagugaaaggucaagauuuuaagucauggguugaucgugaccuucucuuuguauu
new-gga-miRNA-80	uuccagaaccucuuaugcacagu	ugugaugacugguacuggucca	uuccagaaccucuuaugcacaguguaccaugguaaaaucugcugugaugacugguacuggucca
new-gga-miRNA-81	ccgagcgagcucugcagagccu	cugucugcagggccugcuccaucc	cugucugcagggccugcuccaucccgugcugcaaggccgagcgagcucugcagagccu
new-gga-miRNA-83	aaugcuuacuugucuggacaga	agcccagacaaguugaguauuuau	agcccagacaaguugaguauuuauucuccuccugccaauggaauguguaaugcuuacuugucuggacaga
new-gga-miRNA-84	ucacagcccuuccgcccccu	uggggcugcagggcugggagaug	uggggcugcagggcugggagaugugggggcucugcagccccucgacaucacagcccuuccgcccccu
new-gga-miRNA-87	gaagugucacacucacgggcucu	agccuguuggugaggccuucu	agccuguuggugaggccuucuuuugcuucaacuucaagaagugucacacucacgggcucu
new-gga-miRNA-88	cgggcgggcgggcggucg	cgggcgggagguccggcg	cgggcgggcgggcggucggcagggggcgcgggcuuuagugugacgagugcugugcggggcgacgggcgggagguccggcg
new-gga-miRNA-90	acugaugacagagcauugcccu	agcugugcucuguuccucaguucu	agcugugcucuguuccucaguucuggaaguuuuaaucgcugcaauguacugaugacagagcauugcccu
new-gga-miRNA-92	ugaaauccuggccucagcgaguga	cuguugaguucaggauuucu	ugaaauccuggccucagcgagugaggucaaacugcuguugaguucaggauuucu
new-gga-miRNA-93	gugaggcggcggucgggacgc	cggcccgccgcggcccucacgc	gugaggcggcggucgggacgcgcggcaucuccgccccgcggcccgccgcggcccucacgc
new-gga-miRNA-94	guuguucugaaucuaucuugga	caagauagauucagaacaacu	guuguucugaaucuaucuuggaagaaaaaaaucccagcaaauuucaccucuguucaaaguuaacuuccaagauagauucagaacaac
new-gga-miRNA-95	caagagaacaaucugggcaauuu	uugcccacauuuucucuucagu	uugcccacauuuucucuucaguaagagacaaaaaucucaagagaacaaucugggcaauuu
new-gga-miRNA-97	ugugcccacgcagacguacugc	cagaugucugcgugugccucugu	cagaugucugcgugugccucugugcauacauuuuacaugugcccacgcagacguacugc
new-gga-miRNA-98	agcggacaaaggacgauggcac	ugccaucguccuuuguccgcugc	agcggacaaaggacgauggcacacgaagugcucuggaggccugugacaguucaugugccaucguccuuuguccgcugc
new-gga-miRNA-99	cagcgcugggcagugcacugc	agugcagucuguugcagcguggc	agugcagucuguugcagcguggcacaguacguugcucccugcagcgcugggcagugcacugc
new-gga-miRNA-103	ucagcaccccuccguccccgcagu	gcgggggcgcggggugcugagc	gcgggggcgcggggugcugagccguccuuuggggucucuccccgcguggggccuccccccgcucagcaccccuccguccccgcagu
new-gga-miRNA-104	guagugcucuauggggcaccaug	auggggcucuauggggcacc	auggggcucuauggggcaccaugcggugguuauagggcuguguagugcucuauggggcaccaug
new-gga-miRNA-107	acucggcaucgggucugugcc	ugcagauccggagcuaagagugcu	ugcagauccggagcuaagagugcuuuucugcuggcugcacucggcaucgggucugugcc
new-gga-miRNA-109	uucucaucgguggucugcucaga	uuugcagaccaccgaugaga	uuugcagaccaccgaugagaacggaaaagauucccuuuucucaucgguggucugcucaga
new-gga-miRNA-110	augcagaacaguuuucuggacu	auccugaaaauuguaucgcaugg	auccugaaaauuguaucgcaugggguaaagcacagcacugcccaugcagaacaguuuucuggacu
new-gga-miRNA-112	ccccucacccuacuguuccccaga	uggggagcagugaugugggggc	uggggagcagugaugugggggcuccccuggcugcccccucacccuacuguuccccaga
new-gga-miRNA-113	ccccucacccuacuguuccccaga	uggggagcagugaugugggggc	uggggagcagugaugugggggcuccccuggcugcccccucacccuacuguuccccaga
new-gga-miRNA-114	acuugcugaauguuuauggagacc	auggaugugagaacagcgaggugа	acuugcugaauguuuauggagaccaaaugauggaugugagaacagcgaggugau
new-gga-miRNA-115	cuguacgugucccuguuugcu	gggagcuggacaagcagca	cuguacgugucccuguuugcugcaggggagcuggacaagcagca
new-gga-miRNA-119	cauuggguugcacguacuuagu	uaaguacguugaacagccuaagu	uaaguacguugaacagccuaagugcugccuguuagcagucauuggguugcacguacuuagu
new-gga-miRNA-125	ccaucuccaccagcagcacugu	cagugccgacguguggggaugc	cagugccgacguguggggaugcgccgugggggcagagcccaucuccaccagcagcacugu
new-gga-miRNA-126	cuguaacaaauguaaaaaaacu	uuuuuucaacuuguuacagc	cuguaacaaauguaaaaaaacuaaacgugaauuaauuaguuuuuuucaacuuguuacagc
new-gga-miRNA-131	ucuggaauguuuauagugaggcu	ucuacccuauaaacauucugau	ucuggaauguuuauagugaggcugagcuuuauaggucuacccuauaaacauucugau
new-gga-miRNA-134	uuucucugucagugggguaggaggc	uuccuacccauguggagaaaga	uuccuacccauguggagaaagacgcuggucaugucugcuuucucugucagugggguaggaggc
new-gga-miRNA-135	ucagagagcccccagccaaaug	uugggcugggggaucccucgcu	ucagagagcccccagccaaaugugcagugcuguccguugggcugggggaucccucgcu
new-gga-miRNA-136	cccccccaggggacaccagg	gugggcucuggggacaccaggacc	guggcucuggggacaccaggaccccccccaggggacaccagg
new-gga-miRNA-137	cagcagccaugccaucaccgugc	cgguggcagcguggagcuggca	cgguggcagcguggagcuggcacggggaccccaggcugugccagcagccaugccaucaccgugc
new-gga-miRNA-140	gugaggagcucggcacagccagugu	cuggcucugccuccucacuca	gugaggagcucggcacagccaguguauccccauuggcauccccuggcucugccuccucacuca
new-gga-miRNA-141	cugcguaccgugugguggauguc	acaucaccagcggggcggcacuca	acaucaccagcggggcggcacucauagccacugggggggagugcugcguaccguguggugauguc
new-gga-miRNA-145	uccuggcaaauguguagcuuc	aaggugcacguuugccaguucc	uccuggcaaauguguagcuucaggcaaaaugaaaugaaggugcacguuugccaguucc
new-gga-miRNA-149	gagcagcagaaggaucugagu	cuuagauacugcucugcacgac	gagcagcagaaggaucugagugucuguacuaauccucacuuagauacugcucugcacgac
new-gga-miRNA-154	caccacgugucgcgauauaggc	agauaucgcgauaugugaggcguc	agauaucgcgauaugugaggcgucacaauaaucagcugucacacauugcaccacgugucgcgauauaggc
new-gga-miRNA-158	ccaagcagcacuucuagggcacg	ugcuccagaugucugcaggag	ugcuccagaugucugcaggagugccauagucuaauccaagcagcacuucuagggcacg
new-gga-miRNA-159	ccagaacacugcgcaucgaccc	gucgaugcgcagugcugugguca	gucgaugcgcagugcugugggucauggauagaugagaaugaccagaacacugcgcaucgaccc
new-gga-miRNA-160	ucagcugcucugagccccagc	ggggcugaggcuuaguguggagc	ggggcugaggcuuaguguggagcugugcuuacuccugcugcucagcugcucugagccccagc
new-gga-miRNA-161	ugugcaccagcugguggguuggac	ccuacaaaccaccuggugcugg	ugugcaccagcugguggguuggacuggaaaagaagcccuacaaaccaccuggugcugg
new-gga-miRNA-163	ugccacuguagccgggccacc	ugggccgcugcuucagauggcacc	ugggccgcugcuucagauggcaccuguuaugguugguugaagggugccacuguagccgggccacc
new-gga-miRNA-164	cuucccaggcauguuuggaguc	acuaccaacguacuugggaauuu	acuaccaacguacuugggaauuuuuauucauuuacuaaagcuucccaggcauguuuggaguc
new-gga-miRNA-170	cccgcucugucacuguccggc	cgggcagagcucgcagggcgggaa	cccgcucugucacuguccggcugugagguaaccgcgccgggcagagcucgcagggcgggaa
new-gga-miRNA-172	uguuauaguaucccaccuaccc	gugggugggaauaguauaac	gugggugggaauaguauaacaauaugcuaaauguuguuauaguaucccaccuaccc
new-gga-miRNA-176	ucucuccacccuguguccccagu	uuggggcugcaaggugaggcaaag	uuggggcugcaaggugaggcaaagggauaacugcccucucuccacccuguguccccagu
new-gga-miRNA-179	ugguguccugagccugucacagc	agugcacaugccacggac	agugcacaugccacggacuucggggacaugugacagcauccccugguguccugagccugucacagc
new-gga-miRNA-182	ccguaauccucuuagugcuuuga	aagcacaaagaggagcacugcu	aagcacaaagaggagcacugcuucucuccuuggaggccuaagccguaauccucuuagugcuuuga
new-gga-miRNA-187	cuccccucagccgcggcgcugc	cgcuccgcugugaggggagcggg	cuccccucagccgcggcgcugcgcgggaagagccgcuccgcugugaggggagcggg
new-gga-miRNA-188	cuccccucagccgcggcgcugc	cgcuccgcugugaggggagcggg	cuccccucagccgcggcgcugcgcgggaagagccgcuccgcugugaggggagcggg
new-gga-miRNA-191	cauccagcauccuugcccacggu	caugagcaggagaugcuggauaug	caugagcaggagaugcuggauaugaacacuacucccugaagcuucauccagcauccuugcccacggu
new-gga-miRNA-218	gagcaguccuaguguagcaccc	guggcacuggggcugcuguag	gagcaguccuaguguagcacccagcagugaggcuugguggcacuggggcugcuguag
new-gga-miRNA-234	cugagugucacagaacuuugcu	aaaguucuguagugcacugacu	aaaguucuguagugcacugacugcuaucguaaccagagucugagugucacagaacuuugcu
new-gga-miRNA-250	ucucacugcuccucuuuccucaga	gggggugugggggagcauggaggc	gggggugugggggagcauggagggcucagggcugcacagcuccucucacugcuccucuuuccucaga

new-gga-miRNA-252	caggagcuccugcacaucugc	aggugugcaggggcagcucugca	aggugugcaggggcagcucugcagcauuuacuagggcugcaggagcuccugcacaucugc
new-gga-miRNA-253	uacaucacgugggguguggcguc	cgucauaugucacgugggggugug	uacaucacgugggguguggcgucguaugucacgcgggugugacgucauaugucacgugggggugug
new-gga-miRNA-254	uacaucacgugggguguggcguc	ugucaugcgucaugugugugug	ugucaugcgucaugugugugugacgucauacggggggggggggcgucguaagggugcaugaugucauacaucacgugggugug
new-gga-miRNA-257	uacaucacgugggguguggcguc	cgucauaugucacgugggugug	uacaucacgugggguguggcgucguaugucacgcgggugugacgucauaugucacgugggugug
new-gga-miRNA-261	uacaucacgugggguguggcguc	ugucaugcgucaugugugugug	ugucaugcgucaugugugugugacgucauacggggggggggggggcgucguaagggugcaugaugucauacaucacgugggug
new-gga-miRNA-301	cacgccgggaugcagcgaggcg	cgcugcaucccggcguacccc	cacgccgggaugcagcgaggcgcuuccgccucgcugcaucccggcguacccc
new-gga-miRNA-329	caucuuggaguauaagguaccug	uaccugcaccccaagaugacugg	caucuuggaguauaagguaccugauagagggugcagcagguaccugcaccccaagaugacugg
new-gga-miRNA-361	cucaugcccucucugugccagg	ucugggcacaaagagggcagugg	ucugggcacaaagagggcagugggagcagggagagccucaugcccucucugugccagg
new-gga-miRNA-377	cugcuugcccaggaugggggu	agccccaccaugggcagggaga	cugcuugcccaggauggggguauguagugggcgcacagccccaccaugggcagggaga
new-gga-miRNA-408	uuucggcuguuuccggcgacugc	uuccggaaaucgccgaagcgag	uuccggaaaucgccgaagcgagccgaacgcgcugccauuucuguuucggcuguuuccggcgacugc
new-gga-miRNA-416	ugucugaacccagugcagcucucu	agggugcugcuggauuug	ugucugaacccagugcagcucucuuugauuagcuagggugcugcuggauuug
new-gga-miRNA-461	cacacacauguauauauacaca	uauauauaugugcaugugugugua	uauauauaugugcaugugugugauauguauacauguauauacacacacauguauauauacaca
new-gga-miRNA-467	caccuugggagagauuauuggagg	ccuauaaaauagaauggccuuggaa	ccuauaaaauagaauggccuuggaaggugcaaguaggacugaagugguguggccuacuugcaccuugggagagauuauuggagg
new-gga-miRNA-484	ucaggaggugcugcacaugacu	ucuuuugugugggcuuaccuuga	ucuuuugugugggcuuaccuugagggagauagucaucucaggaggugcugcacaugacu
new-gga-miRNA-485	cuggaccgcccgcuugguuaga	acugaucaagugguggugacag	acugaucaagugguggugacagcuauuucuaaaucugcuggaccgcccgcuugguuaga
new-gga-miRNA-486	cauaguaggaaaaggaaguaucu	uuccuuuuccuacucuuguagau	uuccuuuuccuacucuuguagauuuucacuguaaucuacauaguaggaaaaggaaguaucu
new-gga-miRNA-526	uuuggcaaugguagaacucacacu	ggugguucuagacuugccaacu	uuuggcaaugguagaacucacacuggugcgcucgcaggauccggugguucuagacuugccaacu
new-gga-miRNA-548	cugguuguacucugcucccagg	cgugggggggaaagugcaauccu	cgugggggggaaagugcaauccugcauuuggaaaccugguuguacucugcucccagg
new-gga-miRNA-637	aagaggauugaauugcaagacu	uuguaauuuuuuccuucuaga	aagaggauugaauugcaagacuuuguuuuguuaacaucuuguaauuuuuuccuucuaga
new-gga-miRNA-255	caugggcugacgaaccugagauu	uucucagguuagacaguccucg	caugggcugacgaaccugagauugacccucauguuucaccauucucagguuagacaguccucg
new-gga-miRNA-33*	uaacaaucuaaagccacggucg	accauggcuguagacuguuac	accauggcuguagacuguuacccccccagcagcacaguaacaaucuaaagccacggucgc
new-gga-miRNA-55*	gcugggacgcgcgugcgcgcac	ugcgcacacacgcggccugaag	gcugggacgcgcgugcgcgcacguuuguacgcucaggugugcgcacacacgcggccugaag

precursor coordinate

chr13:8493108..8493164:-

chr12:11670979..11671039:+

chr12:11671363..11671426:+

chr20:9710469..9710531:+

chr33:1093263..1093324:+

chr19:5515026..5515081:+

chr19:5515202..5515261:-

chr18:9383535..9383595:+

chr19:5515770..5515834:-

chrUn_NT_467319v1:10243..10320:-

chr33:1171914..1171974:+

chr5:15887078..15887137:+

chr5:21516911..21516976:+

chrZ:16702090..16702149:-

chr1_NT_456790v1_random:3303..3391:+

chr2:412762..412823:+

chr11:17925070..17925127:-

chr8:4658341..4658400:-

chr4:63482790..63482853:-

chr30_NT_463422v1_random:25881..25938:-

chr19:5515021..5515080:-

chr3:87380940..87381002:+

chr3:104167657..104167708:-

chr1:105405945..105406020:+

chrUn_NT_474236v1:2804..2857:+

chr19:5515205..5515264:+

chrW:4032982..4033036:+

chr5:85802..85869:+

chr1:844265..844329:-

chr25:2120335..2120393:-

chrZ:70733193..70733241:+

chr14:6179802..6179865:-

chr4:39800704..39800764:-

chrUn_NT_470743v1:5875..5936:+

chr4:2679511..2679569:+

chr33:1576506..1576565:-

chr12:3154777..3154845:+

chr26:3076088..3076146:-

chr13:17162740..17162796:-

chrUn_NT_467787v1:10611..10694:-

chr18:6449723..6449784:+

chr1:168016562..168016620:-

chr1:144520190..144520257:+

chr3:96912434..96912493:-

chr10:776513..776571:-

chrUn_NT_477504v1:1263..1324:-

chr3:87049050..87049106:+

chr22_NT_463025v1_random:5999..6058:-

chr23:1881170..1881233:-

chr3:110315775..110315834:+

chr3:81811344..81811399:+

chr20:9815102..9815165:-

chrUn_NT_465451v1:14005..14067:+

chr4:5200275..5200327:+

chr1:120628360..120628419:-

chr10:19996779..19996841:-

chr2:138962172..138962230:+

chr5:54895096..54895160:+

chr19:8077898..8077957:+

chr8:24660322..24660381:+

chr4_NT_459802v1_random:59958..60011:+

chr1:50452618..50452677:-

chr1:38476097..38476161:+

chr8:7496343..7496401:+

chr28:3936705..3936775:+

chrUn_NT_464201v1:17930..17997:+

chrZ:43198296..43198356:+

chr1_NT_456372v1_random:327..407:+

chrZ:78517883..78517952:-

chr2:77455962..77456016:+

chr15:743032..743092:+

chr1:54275698..54275786:+

chr4:81629139..81629199:+

chr21:3969023..3969082:-

chr12:5317530..5317608:+

chr3:105077421..105077483:-

chr10:1255073..1255158:+

chrUn_NT_472274v1:5224..5288:+

chr13:17076576..17076635:-

chr10:14704761..14704821:-

chr10:12646179..12646243:+

chr1:673523..673581:+

chr1:669624..669682:+

chr7:8442018..8442072:-

chr5:49858552..49858596:+

chr4:60872218..60872279:-

chr17:1481029..1481088:+

chr2:10086790..10086850:-

chr19:6035263..6035320:+

chr6:30237281..30237343:+

chr13:2583452..2583510:+

chrUn_NT_464199v1:35433..35475:+

chr12:3212327..3212391:-

chr2:4083125..4083188:+

chrUn_NT_472355v1:310..374:+

chr10:11190072..11190130:-

chr4:81308881..81308941:-

chrUn_NT_476753v1:1940..2010:+

chr12:11377981..11378039:-

chr18:2872432..2872494:+

chr12:9880190..9880252:+

chr1:94675708..94675766:+

chr18:47469..47534:-

chr4:63482793..63482855:+

chr10:10695490..10695551:+

chr4:21653671..21653726:+

chr4:12350734..12350793:-

chr1:56702638..56702704:-

chr23:5297200..5297265:-

chr7_NT_461159v1_random:2192..2248:+

chr7:30573447..30573503:-

chr1:92125249..92125316:+

chr5:59355708..59355765:+

chr2:32178259..32178320:+

chr27:5284831..5284897:-

chr15:10688663..10688723:-

chrUn_NT_469680v1:596..660:+

chrUn_NT_469680v1:530..618:+

chrUn_NT_471667v1:612..675:+

chrUn_NT_471667v1:544..634:+

chrUn_NT_465151v1:2037..2089:-

chr2:145259934..145259997:-

chr10:20135923..20135981:-

chr26:4908131..4908189:+

chr2:32251637..32251703:-

chr1:51713695..51713747:+

chr11:7747549..7747611:+

chr4:21963808..21963893:-

chr1:62218231..62218290:-

chr4:35126567..35126626:-

chrZ:40033855..40033916:-

chr1:843242..843306:-

chr8:21706755..21706811:-

chr22:854558..854617:-

chr20:9710466..9710529:-

chr19:5515205..5515265:+

chrUn_NT_477504v1:1263..1324:-
