# Supplementary material for: MiRNA Profiling in Pectoral Muscle Throughout Pre- to Post-Natal Stages of Chicken Development
Source: Front Genet. 2020 Jun 23;11:570. doi: 10.3389/fgene.2020.00570 (PMC7324647; doi:10.3389/fgene.2020.00570)
Supplement: Supplementary file 1 [file Data_Sheet_1.ZIP › Supplementary Material/table S3 Gene Ontology metascape_result.docx]

| **GroupID** | **Category** | **Term** | **Description** | **LogP** | **Log(q-value)** | **InTerm_InList** | **Genes** | **Symbols** |
| --- | --- | --- | --- | --- | --- | --- | --- | --- |
| 1_Summary | GO Biological Processes | GO:0032990 | cell part morphogenesis | -38.12812356 | -33.982 | 168/685 | 60,102,288,323,596,627,659,780,998,1002,1123,1385,1499,1749,1808,1826,2042,2043,2044,2045,2046,2048,2049,2115,2258,2263,2332,2668,2674,2736,2737,2803,2823,2886,2887,3251,3475,3688,3800,3925,4036,4076,4131,4133,4534,4628,4684,4781,4897,4916,4929,5048,5080,5291,5295,5306,5361,5530,5578,5590,5594,5727,5728,5764,5789,5797,5879,5911,6091,6092,6093,6383,6446,6477,6642,6643,6683,6709,6711,6812,6857,6860,7080,7204,7225,7314,7337,7414,7430,7804,8013,8239,8301,8609,8660,8829,8874,8976,9201,9211,9252,9331,9334,9355,9373,9448,9693,9699,9706,9723,9839,10048,10087,10371,10479,10501,10507,10512,10716,10752,10787,10818,10890,11127,11252,22871,22903,22941,22999,23011,23022,23032,23077,23189,23191,23327,23394,23405,23543,23603,23767,26039,26052,26053,27020,27185,51062,55619,55740,56920,56947,56956,57154,57453,57520,57688,64855,64919,80031,84189,84631,84665,84894,137970,152330,153090,285220,494470,87,367,1293,1303,1399,1955,2033,2195,2241,2244,2266,3915,4208,4233,4627,5629,6507,6781,7010,8324,8452,9948,10979,26084,51441,55435,57580,64856,79834,133584,1268,3717,4685,5792,9314,9315,23162,146760,1130,1237,1906,2150,2252,2255,3084,3680,3815,5156,5580,5587,5898,6558,7042,7057,7060,7114,7412,8600,9547,9732,10928,27347,55219,64094,158747 | ACTB,ADAM10,ANK3,APBB2,BCL2,BDNF,BMPR2,DDR1,CDC42,CDH4,CHN1,CREB1,CTNNB1,DLX5,DPYSL2,DSCAM,EPHA3,EPHA4,EPHA5,EPHA7,EPHA8,EPHB2,EPHB3,ETV1,FGF13,FGFR2,FMR1,GDNF,GFRA1,GLI2,GLI3,GOLGA4,GPM6A,GRB7,GRB10,HPRT1,IFRD1,ITGB1,KIF5C,STMN1,LRP2,CAPRIN1,MAP1B,MAP2,MTM1,MYH10,NCAM1,NFIB,NRCAM,NTRK3,NR4A2,PAFAH1B1,PAX6,PIK3CB,PIK3R1,PITPNA,PLXNA1,PPP3CA,PRKCA,PRKCZ,MAPK1,PTCH1,PTEN,PTN,PTPRD,PTPRM,RAC1,RAP2A,ROBO1,ROBO2,ROCK1,SDC2,SGK1,SIAH1,SNX1,SNX2,SPAST,SPTAN1,SPTBN1,STXBP1,SYT1,SYT4,NKX2-1,TRIO,TRPC6,UBB,UBE3A,VCL,EZR,LRP8,NR4A3,USP9X,PICALM,KLF7,IRS2,NRP1,ARHGEF7,WASL,DCLK1,LGI1,RPS6KA5,B4GALT6,B4GALT5,LHX2,PLAA,MAP4K4,RAPGEF2,RIMS2,ULK2,SEMA3E,ZEB2,RANBP9,CERT1,SEMA3A,SLC9A6,SEMA6B,SEMA4D,SEMA3C,TBR1,CHL1,NCKAP1,FRS2,RAB10,KIF3A,PACSIN2,NLGN1,BTBD3,SHANK2,RIMS1,RAB21,PALLD,USP33,MYCBP2,KANK1,CYFIP1,NEDD4L,ADNP,DICER1,RBFOX2,CORO1C,FLRT3,SS18L1,DNM3,AUTS2,NPTN,DISC1,ATL1,DOCK10,ENAH,SEMA3G,MFF,LHX9,SMURF1,DSCAML1,HECW2,ZSWIM6,NIBAN2,BCL11B,SEMA6D,SLITRK6,SLITRK2,MYPN,LINGO1,UNC5D,CNTN4,DAB2IP,EPHA6,RNF165,ACTN1,AR,COL6A3,COL12A1,CRKL,MEGF9,EP300,FAT1,FER,FGB,FGG,LAMC1,MEF2C,MET,MYH9,PROX1,SLC1A3,STC1,TEK,FZD7,CUL3,WDR1,FERMT2,ARHGEF26,YTHDF2,AP1AR,PREX1,VWA1,PEAK1,EGFLAM,CNR1,JAK2,NCAM2,PTPRF,KLF4,NREP,MAPK8IP3,RTN4RL1,LYST,CCR8,EDN1,F2RL1,FGF7,FGF10,NRG1,ITGA9,KIT,PDGFRA,PRKCD,PRKD1,RALA,SLC12A2,TGFB2,THBS1,THBS4,TMSB4X,VCAM1,TNFSF11,CXCL14,DOCK4,RALBP1,STK39,MACO1,SMOC2,MOSPD2 |
| 1_Member | GO Biological Processes | GO:0032990 | cell part morphogenesis | -38.12812356 | -33.982 | 168/685 | 60,102,288,323,596,627,659,780,998,1002,1123,1385,1499,1749,1808,1826,2042,2043,2044,2045,2046,2048,2049,2115,2258,2263,2332,2668,2674,2736,2737,2803,2823,2886,2887,3251,3475,3688,3800,3925,4036,4076,4131,4133,4534,4628,4684,4781,4897,4916,4929,5048,5080,5291,5295,5306,5361,5530,5578,5590,5594,5727,5728,5764,5789,5797,5879,5911,6091,6092,6093,6383,6446,6477,6642,6643,6683,6709,6711,6812,6857,6860,7080,7204,7225,7314,7337,7414,7430,7804,8013,8239,8301,8609,8660,8829,8874,8976,9201,9211,9252,9331,9334,9355,9373,9448,9693,9699,9706,9723,9839,10048,10087,10371,10479,10501,10507,10512,10716,10752,10787,10818,10890,11127,11252,22871,22903,22941,22999,23011,23022,23032,23077,23189,23191,23327,23394,23405,23543,23603,23767,26039,26052,26053,27020,27185,51062,55619,55740,56920,56947,56956,57154,57453,57520,57688,64855,64919,80031,84189,84631,84665,84894,137970,152330,153090,285220,494470 | ACTB,ADAM10,ANK3,APBB2,BCL2,BDNF,BMPR2,DDR1,CDC42,CDH4,CHN1,CREB1,CTNNB1,DLX5,DPYSL2,DSCAM,EPHA3,EPHA4,EPHA5,EPHA7,EPHA8,EPHB2,EPHB3,ETV1,FGF13,FGFR2,FMR1,GDNF,GFRA1,GLI2,GLI3,GOLGA4,GPM6A,GRB7,GRB10,HPRT1,IFRD1,ITGB1,KIF5C,STMN1,LRP2,CAPRIN1,MAP1B,MAP2,MTM1,MYH10,NCAM1,NFIB,NRCAM,NTRK3,NR4A2,PAFAH1B1,PAX6,PIK3CB,PIK3R1,PITPNA,PLXNA1,PPP3CA,PRKCA,PRKCZ,MAPK1,PTCH1,PTEN,PTN,PTPRD,PTPRM,RAC1,RAP2A,ROBO1,ROBO2,ROCK1,SDC2,SGK1,SIAH1,SNX1,SNX2,SPAST,SPTAN1,SPTBN1,STXBP1,SYT1,SYT4,NKX2-1,TRIO,TRPC6,UBB,UBE3A,VCL,EZR,LRP8,NR4A3,USP9X,PICALM,KLF7,IRS2,NRP1,ARHGEF7,WASL,DCLK1,LGI1,RPS6KA5,B4GALT6,B4GALT5,LHX2,PLAA,MAP4K4,RAPGEF2,RIMS2,ULK2,SEMA3E,ZEB2,RANBP9,CERT1,SEMA3A,SLC9A6,SEMA6B,SEMA4D,SEMA3C,TBR1,CHL1,NCKAP1,FRS2,RAB10,KIF3A,PACSIN2,NLGN1,BTBD3,SHANK2,RIMS1,RAB21,PALLD,USP33,MYCBP2,KANK1,CYFIP1,NEDD4L,ADNP,DICER1,RBFOX2,CORO1C,FLRT3,SS18L1,DNM3,AUTS2,NPTN,DISC1,ATL1,DOCK10,ENAH,SEMA3G,MFF,LHX9,SMURF1,DSCAML1,HECW2,ZSWIM6,NIBAN2,BCL11B,SEMA6D,SLITRK6,SLITRK2,MYPN,LINGO1,UNC5D,CNTN4,DAB2IP,EPHA6,RNF165 |
| 1_Member | GO Biological Processes | GO:0048858 | cell projection morphogenesis | -38.0320541 | -33.982 | 165/666 | 60,102,288,323,596,627,659,780,998,1002,1123,1385,1499,1749,1808,1826,2042,2043,2044,2045,2046,2048,2049,2115,2258,2263,2332,2668,2674,2736,2737,2803,2823,2886,2887,3251,3475,3688,3800,3925,4036,4076,4131,4133,4628,4684,4781,4897,4916,4929,5048,5080,5291,5295,5306,5361,5530,5578,5590,5594,5727,5728,5764,5789,5797,5879,5911,6091,6092,6093,6383,6446,6477,6642,6643,6683,6709,6711,6812,6857,6860,7080,7204,7225,7314,7337,7414,7430,7804,8013,8239,8301,8609,8660,8829,8874,8976,9201,9211,9252,9331,9334,9355,9373,9448,9693,9699,9706,9723,9839,10048,10371,10479,10501,10507,10512,10716,10752,10787,10818,10890,11127,11252,22871,22903,22941,22999,23011,23022,23032,23077,23189,23191,23327,23394,23405,23543,23603,23767,26039,26052,26053,27020,27185,51062,55619,55740,56920,56956,57154,57453,57520,57688,64855,64919,80031,84189,84631,84665,84894,137970,152330,153090,285220,494470 | ACTB,ADAM10,ANK3,APBB2,BCL2,BDNF,BMPR2,DDR1,CDC42,CDH4,CHN1,CREB1,CTNNB1,DLX5,DPYSL2,DSCAM,EPHA3,EPHA4,EPHA5,EPHA7,EPHA8,EPHB2,EPHB3,ETV1,FGF13,FGFR2,FMR1,GDNF,GFRA1,GLI2,GLI3,GOLGA4,GPM6A,GRB7,GRB10,HPRT1,IFRD1,ITGB1,KIF5C,STMN1,LRP2,CAPRIN1,MAP1B,MAP2,MYH10,NCAM1,NFIB,NRCAM,NTRK3,NR4A2,PAFAH1B1,PAX6,PIK3CB,PIK3R1,PITPNA,PLXNA1,PPP3CA,PRKCA,PRKCZ,MAPK1,PTCH1,PTEN,PTN,PTPRD,PTPRM,RAC1,RAP2A,ROBO1,ROBO2,ROCK1,SDC2,SGK1,SIAH1,SNX1,SNX2,SPAST,SPTAN1,SPTBN1,STXBP1,SYT1,SYT4,NKX2-1,TRIO,TRPC6,UBB,UBE3A,VCL,EZR,LRP8,NR4A3,USP9X,PICALM,KLF7,IRS2,NRP1,ARHGEF7,WASL,DCLK1,LGI1,RPS6KA5,B4GALT6,B4GALT5,LHX2,PLAA,MAP4K4,RAPGEF2,RIMS2,ULK2,SEMA3E,ZEB2,RANBP9,SEMA3A,SLC9A6,SEMA6B,SEMA4D,SEMA3C,TBR1,CHL1,NCKAP1,FRS2,RAB10,KIF3A,PACSIN2,NLGN1,BTBD3,SHANK2,RIMS1,RAB21,PALLD,USP33,MYCBP2,KANK1,CYFIP1,NEDD4L,ADNP,DICER1,RBFOX2,CORO1C,FLRT3,SS18L1,DNM3,AUTS2,NPTN,DISC1,ATL1,DOCK10,ENAH,SEMA3G,LHX9,SMURF1,DSCAML1,HECW2,ZSWIM6,NIBAN2,BCL11B,SEMA6D,SLITRK6,SLITRK2,MYPN,LINGO1,UNC5D,CNTN4,DAB2IP,EPHA6,RNF165 |
| 1_Member | GO Biological Processes | GO:0120039 | plasma membrane bounded cell projection morphogenesis | -37.79559651 | -33.922 | 164/662 | 60,102,288,323,596,627,659,780,998,1002,1123,1385,1499,1749,1808,1826,2042,2043,2044,2045,2046,2048,2049,2115,2258,2263,2332,2668,2674,2736,2737,2803,2823,2886,2887,3251,3475,3688,3800,3925,4036,4076,4131,4133,4628,4684,4781,4897,4916,4929,5048,5080,5291,5295,5306,5361,5530,5578,5590,5594,5727,5728,5764,5789,5797,5879,5911,6091,6092,6093,6383,6446,6477,6642,6643,6683,6709,6711,6812,6857,6860,7080,7204,7225,7314,7337,7414,7430,7804,8013,8239,8301,8609,8660,8829,8874,8976,9201,9211,9252,9331,9334,9355,9373,9448,9693,9699,9706,9723,9839,10048,10371,10479,10501,10507,10512,10716,10752,10787,10818,10890,11127,22871,22903,22941,22999,23011,23022,23032,23077,23189,23191,23327,23394,23405,23543,23603,23767,26039,26052,26053,27020,27185,51062,55619,55740,56920,56956,57154,57453,57520,57688,64855,64919,80031,84189,84631,84665,84894,137970,152330,153090,285220,494470 | ACTB,ADAM10,ANK3,APBB2,BCL2,BDNF,BMPR2,DDR1,CDC42,CDH4,CHN1,CREB1,CTNNB1,DLX5,DPYSL2,DSCAM,EPHA3,EPHA4,EPHA5,EPHA7,EPHA8,EPHB2,EPHB3,ETV1,FGF13,FGFR2,FMR1,GDNF,GFRA1,GLI2,GLI3,GOLGA4,GPM6A,GRB7,GRB10,HPRT1,IFRD1,ITGB1,KIF5C,STMN1,LRP2,CAPRIN1,MAP1B,MAP2,MYH10,NCAM1,NFIB,NRCAM,NTRK3,NR4A2,PAFAH1B1,PAX6,PIK3CB,PIK3R1,PITPNA,PLXNA1,PPP3CA,PRKCA,PRKCZ,MAPK1,PTCH1,PTEN,PTN,PTPRD,PTPRM,RAC1,RAP2A,ROBO1,ROBO2,ROCK1,SDC2,SGK1,SIAH1,SNX1,SNX2,SPAST,SPTAN1,SPTBN1,STXBP1,SYT1,SYT4,NKX2-1,TRIO,TRPC6,UBB,UBE3A,VCL,EZR,LRP8,NR4A3,USP9X,PICALM,KLF7,IRS2,NRP1,ARHGEF7,WASL,DCLK1,LGI1,RPS6KA5,B4GALT6,B4GALT5,LHX2,PLAA,MAP4K4,RAPGEF2,RIMS2,ULK2,SEMA3E,ZEB2,RANBP9,SEMA3A,SLC9A6,SEMA6B,SEMA4D,SEMA3C,TBR1,CHL1,NCKAP1,FRS2,RAB10,KIF3A,NLGN1,BTBD3,SHANK2,RIMS1,RAB21,PALLD,USP33,MYCBP2,KANK1,CYFIP1,NEDD4L,ADNP,DICER1,RBFOX2,CORO1C,FLRT3,SS18L1,DNM3,AUTS2,NPTN,DISC1,ATL1,DOCK10,ENAH,SEMA3G,LHX9,SMURF1,DSCAML1,HECW2,ZSWIM6,NIBAN2,BCL11B,SEMA6D,SLITRK6,SLITRK2,MYPN,LINGO1,UNC5D,CNTN4,DAB2IP,EPHA6,RNF165 |
| 1_Member | GO Biological Processes | GO:0000904 | cell morphogenesis involved in differentiation | -37.65896019 | -33.910 | 176/745 | 60,87,102,288,323,367,596,627,659,998,1002,1123,1293,1303,1385,1399,1499,1749,1808,1826,1955,2033,2042,2043,2044,2045,2046,2048,2049,2115,2195,2241,2244,2258,2263,2266,2332,2668,2674,2736,2737,2803,2886,2887,3251,3475,3688,3800,3915,3925,4076,4131,4133,4208,4233,4627,4628,4684,4781,4897,4916,4929,5048,5080,5291,5295,5306,5361,5530,5578,5594,5629,5727,5728,5764,5789,5797,5879,5911,6091,6092,6093,6383,6477,6507,6683,6709,6711,6781,6812,7010,7080,7204,7225,7337,7414,7430,7804,8013,8239,8301,8324,8452,8609,8660,8829,8874,8976,9201,9211,9252,9331,9334,9355,9693,9706,9723,9839,9948,10048,10371,10479,10501,10507,10512,10716,10752,10818,10890,10979,11127,22871,22903,22941,23011,23022,23032,23077,23189,23191,23327,23394,23543,23603,23767,26039,26052,26053,26084,27020,27185,51062,51441,55435,55619,55740,56920,56956,57453,57520,57580,57688,64855,64856,64919,79834,80031,84189,84631,84665,84894,133584,137970,152330,285220,494470 | ACTB,ACTN1,ADAM10,ANK3,APBB2,AR,BCL2,BDNF,BMPR2,CDC42,CDH4,CHN1,COL6A3,COL12A1,CREB1,CRKL,CTNNB1,DLX5,DPYSL2,DSCAM,MEGF9,EP300,EPHA3,EPHA4,EPHA5,EPHA7,EPHA8,EPHB2,EPHB3,ETV1,FAT1,FER,FGB,FGF13,FGFR2,FGG,FMR1,GDNF,GFRA1,GLI2,GLI3,GOLGA4,GRB7,GRB10,HPRT1,IFRD1,ITGB1,KIF5C,LAMC1,STMN1,CAPRIN1,MAP1B,MAP2,MEF2C,MET,MYH9,MYH10,NCAM1,NFIB,NRCAM,NTRK3,NR4A2,PAFAH1B1,PAX6,PIK3CB,PIK3R1,PITPNA,PLXNA1,PPP3CA,PRKCA,MAPK1,PROX1,PTCH1,PTEN,PTN,PTPRD,PTPRM,RAC1,RAP2A,ROBO1,ROBO2,ROCK1,SDC2,SIAH1,SLC1A3,SPAST,SPTAN1,SPTBN1,STC1,STXBP1,TEK,NKX2-1,TRIO,TRPC6,UBE3A,VCL,EZR,LRP8,NR4A3,USP9X,PICALM,FZD7,CUL3,KLF7,IRS2,NRP1,ARHGEF7,WASL,DCLK1,LGI1,RPS6KA5,B4GALT6,B4GALT5,LHX2,RAPGEF2,ULK2,SEMA3E,ZEB2,WDR1,RANBP9,SEMA3A,SLC9A6,SEMA6B,SEMA4D,SEMA3C,TBR1,CHL1,FRS2,RAB10,FERMT2,KIF3A,NLGN1,BTBD3,SHANK2,RAB21,PALLD,USP33,MYCBP2,KANK1,CYFIP1,NEDD4L,ADNP,RBFOX2,CORO1C,FLRT3,SS18L1,DNM3,AUTS2,ARHGEF26,NPTN,DISC1,ATL1,YTHDF2,AP1AR,DOCK10,ENAH,SEMA3G,LHX9,DSCAML1,HECW2,PREX1,ZSWIM6,NIBAN2,VWA1,BCL11B,PEAK1,SEMA6D,SLITRK6,SLITRK2,MYPN,LINGO1,EGFLAM,UNC5D,CNTN4,EPHA6,RNF165 |
| 1_Member | GO Biological Processes | GO:0048812 | neuron projection morphogenesis | -36.08940602 | -32.438 | 159/648 | 60,102,288,323,596,627,659,780,998,1002,1123,1385,1499,1749,1808,1826,2042,2043,2044,2045,2046,2048,2049,2115,2258,2263,2332,2668,2674,2736,2737,2803,2823,2886,2887,3251,3475,3688,3800,3925,4036,4076,4131,4133,4628,4684,4781,4897,4916,4929,5048,5080,5291,5295,5306,5361,5530,5578,5590,5594,5727,5728,5764,5789,5797,5879,5911,6091,6092,6093,6383,6446,6477,6683,6709,6711,6812,6857,6860,7080,7204,7225,7314,7337,7414,7430,7804,8013,8239,8301,8609,8660,8829,8976,9201,9211,9252,9331,9334,9355,9373,9448,9693,9699,9706,9723,9839,10048,10371,10479,10501,10507,10512,10716,10752,10787,10818,10890,11127,22871,22903,22941,22999,23011,23022,23032,23077,23191,23327,23394,23405,23543,23767,26039,26052,26053,27020,27185,51062,55619,55740,56920,56956,57154,57453,57520,57688,64855,64919,80031,84189,84631,84665,84894,137970,152330,153090,285220,494470 | ACTB,ADAM10,ANK3,APBB2,BCL2,BDNF,BMPR2,DDR1,CDC42,CDH4,CHN1,CREB1,CTNNB1,DLX5,DPYSL2,DSCAM,EPHA3,EPHA4,EPHA5,EPHA7,EPHA8,EPHB2,EPHB3,ETV1,FGF13,FGFR2,FMR1,GDNF,GFRA1,GLI2,GLI3,GOLGA4,GPM6A,GRB7,GRB10,HPRT1,IFRD1,ITGB1,KIF5C,STMN1,LRP2,CAPRIN1,MAP1B,MAP2,MYH10,NCAM1,NFIB,NRCAM,NTRK3,NR4A2,PAFAH1B1,PAX6,PIK3CB,PIK3R1,PITPNA,PLXNA1,PPP3CA,PRKCA,PRKCZ,MAPK1,PTCH1,PTEN,PTN,PTPRD,PTPRM,RAC1,RAP2A,ROBO1,ROBO2,ROCK1,SDC2,SGK1,SIAH1,SPAST,SPTAN1,SPTBN1,STXBP1,SYT1,SYT4,NKX2-1,TRIO,TRPC6,UBB,UBE3A,VCL,EZR,LRP8,NR4A3,USP9X,PICALM,KLF7,IRS2,NRP1,WASL,DCLK1,LGI1,RPS6KA5,B4GALT6,B4GALT5,LHX2,PLAA,MAP4K4,RAPGEF2,RIMS2,ULK2,SEMA3E,ZEB2,RANBP9,SEMA3A,SLC9A6,SEMA6B,SEMA4D,SEMA3C,TBR1,CHL1,NCKAP1,FRS2,RAB10,KIF3A,NLGN1,BTBD3,SHANK2,RIMS1,RAB21,PALLD,USP33,MYCBP2,CYFIP1,NEDD4L,ADNP,DICER1,RBFOX2,FLRT3,SS18L1,DNM3,AUTS2,NPTN,DISC1,ATL1,DOCK10,ENAH,SEMA3G,LHX9,SMURF1,DSCAML1,HECW2,ZSWIM6,NIBAN2,BCL11B,SEMA6D,SLITRK6,SLITRK2,MYPN,LINGO1,UNC5D,CNTN4,DAB2IP,EPHA6,RNF165 |
| 1_Member | GO Biological Processes | GO:0048667 | cell morphogenesis involved in neuron differentiation | -32.41918359 | -28.972 | 143/583 | 60,102,288,323,596,627,659,998,1002,1123,1385,1749,1808,1826,2042,2043,2044,2045,2046,2048,2049,2115,2258,2263,2332,2668,2674,2736,2737,2803,2886,2887,3251,3475,3688,3800,3925,4076,4131,4133,4208,4628,4684,4781,4897,4916,4929,5048,5080,5291,5295,5306,5361,5530,5578,5594,5727,5728,5764,5789,5797,5879,5911,6091,6092,6383,6477,6507,6683,6709,6711,6812,7080,7204,7225,7337,7414,7430,7804,8013,8239,8301,8609,8660,8829,8976,9201,9211,9252,9331,9334,9355,9693,9706,9723,9839,10048,10371,10479,10501,10507,10512,10716,10752,10818,10890,11127,22871,22903,22941,23011,23022,23032,23077,23191,23327,23394,23543,23767,26039,26052,26053,27020,27185,51062,55619,55740,56920,56956,57453,57520,57688,64855,64919,80031,84189,84631,84665,84894,137970,152330,285220,494470 | ACTB,ADAM10,ANK3,APBB2,BCL2,BDNF,BMPR2,CDC42,CDH4,CHN1,CREB1,DLX5,DPYSL2,DSCAM,EPHA3,EPHA4,EPHA5,EPHA7,EPHA8,EPHB2,EPHB3,ETV1,FGF13,FGFR2,FMR1,GDNF,GFRA1,GLI2,GLI3,GOLGA4,GRB7,GRB10,HPRT1,IFRD1,ITGB1,KIF5C,STMN1,CAPRIN1,MAP1B,MAP2,MEF2C,MYH10,NCAM1,NFIB,NRCAM,NTRK3,NR4A2,PAFAH1B1,PAX6,PIK3CB,PIK3R1,PITPNA,PLXNA1,PPP3CA,PRKCA,MAPK1,PTCH1,PTEN,PTN,PTPRD,PTPRM,RAC1,RAP2A,ROBO1,ROBO2,SDC2,SIAH1,SLC1A3,SPAST,SPTAN1,SPTBN1,STXBP1,NKX2-1,TRIO,TRPC6,UBE3A,VCL,EZR,LRP8,NR4A3,USP9X,PICALM,KLF7,IRS2,NRP1,WASL,DCLK1,LGI1,RPS6KA5,B4GALT6,B4GALT5,LHX2,RAPGEF2,ULK2,SEMA3E,ZEB2,RANBP9,SEMA3A,SLC9A6,SEMA6B,SEMA4D,SEMA3C,TBR1,CHL1,FRS2,RAB10,KIF3A,NLGN1,BTBD3,SHANK2,RAB21,PALLD,USP33,MYCBP2,CYFIP1,NEDD4L,ADNP,RBFOX2,FLRT3,SS18L1,DNM3,AUTS2,NPTN,DISC1,ATL1,DOCK10,ENAH,SEMA3G,LHX9,DSCAML1,HECW2,ZSWIM6,NIBAN2,BCL11B,SEMA6D,SLITRK6,SLITRK2,MYPN,LINGO1,UNC5D,CNTN4,EPHA6,RNF165 |
| 1_Member | GO Biological Processes | GO:0061564 | axon development | -29.12410941 | -25.728 | 127/514 | 60,288,323,596,627,659,780,1002,1123,1268,1385,1749,1808,1826,2042,2043,2044,2045,2046,2048,2049,2115,2258,2263,2668,2674,2736,2737,2803,2886,2887,3475,3688,3717,3800,3925,4131,4133,4628,4684,4685,4781,4897,4916,4929,5048,5080,5291,5295,5306,5361,5578,5594,5727,5728,5764,5792,5797,5879,6091,6092,6477,6683,6709,6711,6812,7080,7204,7414,7430,8013,8239,8301,8609,8660,8829,9201,9211,9252,9314,9315,9331,9334,9355,9706,9723,9839,10048,10371,10479,10501,10507,10512,10716,10752,10818,10890,11127,23011,23022,23032,23077,23162,23191,23394,23767,26053,27020,27185,51062,55740,56920,56956,57453,57688,64855,64919,80031,84189,84631,84665,84894,137970,146760,152330,285220,494470 | ACTB,ANK3,APBB2,BCL2,BDNF,BMPR2,DDR1,CDH4,CHN1,CNR1,CREB1,DLX5,DPYSL2,DSCAM,EPHA3,EPHA4,EPHA5,EPHA7,EPHA8,EPHB2,EPHB3,ETV1,FGF13,FGFR2,GDNF,GFRA1,GLI2,GLI3,GOLGA4,GRB7,GRB10,IFRD1,ITGB1,JAK2,KIF5C,STMN1,MAP1B,MAP2,MYH10,NCAM1,NCAM2,NFIB,NRCAM,NTRK3,NR4A2,PAFAH1B1,PAX6,PIK3CB,PIK3R1,PITPNA,PLXNA1,PRKCA,MAPK1,PTCH1,PTEN,PTN,PTPRF,PTPRM,RAC1,ROBO1,ROBO2,SIAH1,SPAST,SPTAN1,SPTBN1,STXBP1,NKX2-1,TRIO,VCL,EZR,NR4A3,USP9X,PICALM,KLF7,IRS2,NRP1,DCLK1,LGI1,RPS6KA5,KLF4,NREP,B4GALT6,B4GALT5,LHX2,ULK2,SEMA3E,ZEB2,RANBP9,SEMA3A,SLC9A6,SEMA6B,SEMA4D,SEMA3C,TBR1,CHL1,FRS2,RAB10,KIF3A,RAB21,PALLD,USP33,MYCBP2,MAPK8IP3,CYFIP1,ADNP,FLRT3,AUTS2,NPTN,DISC1,ATL1,ENAH,SEMA3G,LHX9,DSCAML1,ZSWIM6,NIBAN2,BCL11B,SEMA6D,SLITRK6,SLITRK2,MYPN,LINGO1,UNC5D,RTN4RL1,CNTN4,EPHA6,RNF165 |
| 1_Member | GO Biological Processes | GO:0007409 | axonogenesis | -27.33466786 | -24.127 | 117/468 | 60,288,323,596,627,659,1002,1123,1385,1749,1808,1826,2042,2043,2044,2045,2046,2048,2049,2115,2258,2263,2668,2674,2736,2737,2803,2886,2887,3475,3688,3800,3925,4131,4133,4628,4684,4781,4897,4916,4929,5048,5080,5291,5295,5306,5361,5578,5594,5727,5728,5797,5879,6091,6092,6477,6683,6709,6711,6812,7080,7204,7414,7430,8013,8239,8301,8609,8660,8829,9201,9211,9252,9331,9334,9355,9706,9723,9839,10048,10371,10479,10501,10507,10512,10716,10752,10818,10890,11127,23011,23022,23032,23077,23191,23394,23767,26053,27020,27185,51062,55740,56920,56956,57453,57688,64855,64919,80031,84189,84631,84665,84894,137970,152330,285220,494470 | ACTB,ANK3,APBB2,BCL2,BDNF,BMPR2,CDH4,CHN1,CREB1,DLX5,DPYSL2,DSCAM,EPHA3,EPHA4,EPHA5,EPHA7,EPHA8,EPHB2,EPHB3,ETV1,FGF13,FGFR2,GDNF,GFRA1,GLI2,GLI3,GOLGA4,GRB7,GRB10,IFRD1,ITGB1,KIF5C,STMN1,MAP1B,MAP2,MYH10,NCAM1,NFIB,NRCAM,NTRK3,NR4A2,PAFAH1B1,PAX6,PIK3CB,PIK3R1,PITPNA,PLXNA1,PRKCA,MAPK1,PTCH1,PTEN,PTPRM,RAC1,ROBO1,ROBO2,SIAH1,SPAST,SPTAN1,SPTBN1,STXBP1,NKX2-1,TRIO,VCL,EZR,NR4A3,USP9X,PICALM,KLF7,IRS2,NRP1,DCLK1,LGI1,RPS6KA5,B4GALT6,B4GALT5,LHX2,ULK2,SEMA3E,ZEB2,RANBP9,SEMA3A,SLC9A6,SEMA6B,SEMA4D,SEMA3C,TBR1,CHL1,FRS2,RAB10,KIF3A,RAB21,PALLD,USP33,MYCBP2,CYFIP1,ADNP,FLRT3,AUTS2,NPTN,DISC1,ATL1,ENAH,SEMA3G,LHX9,DSCAML1,ZSWIM6,NIBAN2,BCL11B,SEMA6D,SLITRK6,SLITRK2,MYPN,LINGO1,UNC5D,CNTN4,EPHA6,RNF165 |
| 1_Member | GO Biological Processes | GO:0007411 | axon guidance | -23.23051609 | -20.110 | 80/276 | 323,627,659,1002,1123,1749,1808,1826,2042,2043,2044,2045,2046,2048,2049,2115,2668,2674,2736,2737,2886,2887,3800,4628,4684,4781,4897,5080,5291,5295,5361,5578,5594,5727,5797,5879,6091,6092,6477,6709,6711,7080,7204,7430,8013,8609,8660,8829,9211,9252,9355,9723,10048,10371,10501,10507,10512,10716,10752,10818,11127,23022,23032,23077,23191,23767,27020,55740,56920,56956,57453,57688,64855,64919,80031,84665,137970,152330,285220,494470 | APBB2,BDNF,BMPR2,CDH4,CHN1,DLX5,DPYSL2,DSCAM,EPHA3,EPHA4,EPHA5,EPHA7,EPHA8,EPHB2,EPHB3,ETV1,GDNF,GFRA1,GLI2,GLI3,GRB7,GRB10,KIF5C,MYH10,NCAM1,NFIB,NRCAM,PAX6,PIK3CB,PIK3R1,PLXNA1,PRKCA,MAPK1,PTCH1,PTPRM,RAC1,ROBO1,ROBO2,SIAH1,SPTAN1,SPTBN1,NKX2-1,TRIO,EZR,NR4A3,KLF7,IRS2,NRP1,LGI1,RPS6KA5,LHX2,SEMA3E,RANBP9,SEMA3A,SEMA6B,SEMA4D,SEMA3C,TBR1,CHL1,FRS2,KIF3A,PALLD,USP33,MYCBP2,CYFIP1,FLRT3,NPTN,ENAH,SEMA3G,LHX9,DSCAML1,ZSWIM6,NIBAN2,BCL11B,SEMA6D,MYPN,UNC5D,CNTN4,EPHA6,RNF165 |
| 1_Member | GO Biological Processes | GO:0097485 | neuron projection guidance | -23.11842826 | -20.047 | 80/277 | 323,627,659,1002,1123,1749,1808,1826,2042,2043,2044,2045,2046,2048,2049,2115,2668,2674,2736,2737,2886,2887,3800,4628,4684,4781,4897,5080,5291,5295,5361,5578,5594,5727,5797,5879,6091,6092,6477,6709,6711,7080,7204,7430,8013,8609,8660,8829,9211,9252,9355,9723,10048,10371,10501,10507,10512,10716,10752,10818,11127,23022,23032,23077,23191,23767,27020,55740,56920,56956,57453,57688,64855,64919,80031,84665,137970,152330,285220,494470 | APBB2,BDNF,BMPR2,CDH4,CHN1,DLX5,DPYSL2,DSCAM,EPHA3,EPHA4,EPHA5,EPHA7,EPHA8,EPHB2,EPHB3,ETV1,GDNF,GFRA1,GLI2,GLI3,GRB7,GRB10,KIF5C,MYH10,NCAM1,NFIB,NRCAM,PAX6,PIK3CB,PIK3R1,PLXNA1,PRKCA,MAPK1,PTCH1,PTPRM,RAC1,ROBO1,ROBO2,SIAH1,SPTAN1,SPTBN1,NKX2-1,TRIO,EZR,NR4A3,KLF7,IRS2,NRP1,LGI1,RPS6KA5,LHX2,SEMA3E,RANBP9,SEMA3A,SEMA6B,SEMA4D,SEMA3C,TBR1,CHL1,FRS2,KIF3A,PALLD,USP33,MYCBP2,CYFIP1,FLRT3,NPTN,ENAH,SEMA3G,LHX9,DSCAML1,ZSWIM6,NIBAN2,BCL11B,SEMA6D,MYPN,UNC5D,CNTN4,EPHA6,RNF165 |
| 1_Member | GO Biological Processes | GO:0042330 | taxis | -14.83322766 | -12.491 | 116/647 | 102,323,627,659,1002,1123,1130,1237,1385,1399,1749,1808,1826,1906,2033,2042,2043,2044,2045,2046,2048,2049,2115,2150,2241,2252,2255,2668,2674,2736,2737,2886,2887,3084,3680,3800,3815,4233,4628,4684,4781,4897,4916,5080,5156,5291,5295,5361,5578,5580,5587,5594,5727,5764,5797,5879,5898,6091,6092,6477,6558,6709,6711,7042,7057,7060,7080,7114,7204,7412,7430,8013,8600,8609,8660,8829,9211,9252,9355,9547,9723,9732,10048,10371,10501,10507,10512,10716,10752,10818,10928,11127,23022,23032,23077,23191,23767,27020,27347,55219,55740,56920,56956,57453,57580,57688,64094,64855,64919,80031,84665,137970,152330,158747,285220,494470 | ADAM10,APBB2,BDNF,BMPR2,CDH4,CHN1,LYST,CCR8,CREB1,CRKL,DLX5,DPYSL2,DSCAM,EDN1,EP300,EPHA3,EPHA4,EPHA5,EPHA7,EPHA8,EPHB2,EPHB3,ETV1,F2RL1,FER,FGF7,FGF10,GDNF,GFRA1,GLI2,GLI3,GRB7,GRB10,NRG1,ITGA9,KIF5C,KIT,MET,MYH10,NCAM1,NFIB,NRCAM,NTRK3,PAX6,PDGFRA,PIK3CB,PIK3R1,PLXNA1,PRKCA,PRKCD,PRKD1,MAPK1,PTCH1,PTN,PTPRM,RAC1,RALA,ROBO1,ROBO2,SIAH1,SLC12A2,SPTAN1,SPTBN1,TGFB2,THBS1,THBS4,NKX2-1,TMSB4X,TRIO,VCAM1,EZR,NR4A3,TNFSF11,KLF7,IRS2,NRP1,LGI1,RPS6KA5,LHX2,CXCL14,SEMA3E,DOCK4,RANBP9,SEMA3A,SEMA6B,SEMA4D,SEMA3C,TBR1,CHL1,FRS2,RALBP1,KIF3A,PALLD,USP33,MYCBP2,CYFIP1,FLRT3,NPTN,STK39,MACO1,ENAH,SEMA3G,LHX9,DSCAML1,PREX1,ZSWIM6,SMOC2,NIBAN2,BCL11B,SEMA6D,MYPN,UNC5D,CNTN4,MOSPD2,EPHA6,RNF165 |
| 1_Member | GO Biological Processes | GO:0006935 | chemotaxis | -14.53184884 | -12.215 | 115/645 | 102,323,627,659,1002,1123,1130,1237,1385,1399,1749,1808,1826,1906,2042,2043,2044,2045,2046,2048,2049,2115,2150,2241,2252,2255,2668,2674,2736,2737,2886,2887,3084,3680,3800,3815,4233,4628,4684,4781,4897,4916,5080,5156,5291,5295,5361,5578,5580,5587,5594,5727,5764,5797,5879,5898,6091,6092,6477,6558,6709,6711,7042,7057,7060,7080,7114,7204,7412,7430,8013,8600,8609,8660,8829,9211,9252,9355,9547,9723,9732,10048,10371,10501,10507,10512,10716,10752,10818,10928,11127,23022,23032,23077,23191,23767,27020,27347,55219,55740,56920,56956,57453,57580,57688,64094,64855,64919,80031,84665,137970,152330,158747,285220,494470 | ADAM10,APBB2,BDNF,BMPR2,CDH4,CHN1,LYST,CCR8,CREB1,CRKL,DLX5,DPYSL2,DSCAM,EDN1,EPHA3,EPHA4,EPHA5,EPHA7,EPHA8,EPHB2,EPHB3,ETV1,F2RL1,FER,FGF7,FGF10,GDNF,GFRA1,GLI2,GLI3,GRB7,GRB10,NRG1,ITGA9,KIF5C,KIT,MET,MYH10,NCAM1,NFIB,NRCAM,NTRK3,PAX6,PDGFRA,PIK3CB,PIK3R1,PLXNA1,PRKCA,PRKCD,PRKD1,MAPK1,PTCH1,PTN,PTPRM,RAC1,RALA,ROBO1,ROBO2,SIAH1,SLC12A2,SPTAN1,SPTBN1,TGFB2,THBS1,THBS4,NKX2-1,TMSB4X,TRIO,VCAM1,EZR,NR4A3,TNFSF11,KLF7,IRS2,NRP1,LGI1,RPS6KA5,LHX2,CXCL14,SEMA3E,DOCK4,RANBP9,SEMA3A,SEMA6B,SEMA4D,SEMA3C,TBR1,CHL1,FRS2,RALBP1,KIF3A,PALLD,USP33,MYCBP2,CYFIP1,FLRT3,NPTN,STK39,MACO1,ENAH,SEMA3G,LHX9,DSCAML1,PREX1,ZSWIM6,SMOC2,NIBAN2,BCL11B,SEMA6D,MYPN,UNC5D,CNTN4,MOSPD2,EPHA6,RNF165 |
| 2_Summary | GO Molecular Functions | GO:0000977 | RNA polymerase II regulatory region sequence-specific DNA binding | -33.76811578 | -30.196 | 175/788 | 60,196,367,467,571,604,1046,1106,1316,1385,1390,1602,1749,1750,1871,1958,1997,2004,2018,2033,2113,2115,2117,2294,2295,2296,2313,2494,2624,2736,2737,2908,2959,3021,3096,3131,3190,3192,3202,3204,3205,3217,3239,3516,3607,3642,3720,4005,4084,4091,4094,4097,4208,4211,4223,4286,4335,4487,4603,4613,4661,4760,4773,4775,4781,4783,4841,4929,5080,5090,5308,5451,5468,5629,5887,5915,5978,5991,6095,6096,6299,6304,6421,6591,6598,6603,6670,6908,6913,6929,6934,6935,7020,7022,7029,7050,7068,7080,7150,7182,7403,7528,7690,7702,7707,8013,8204,8531,8545,8553,8609,8726,8850,9314,9355,9480,9519,9555,9575,9705,9925,9960,9967,10153,10336,10499,10716,10725,11278,23040,23269,23314,23512,23635,26298,26468,27316,28999,29942,50804,51274,51621,51804,54880,55145,55506,55553,55636,55749,55922,56956,60468,63973,63974,64864,64919,79365,79733,79776,83741,84159,84333,84678,93986,121536,130497,132660,144455,145258,152485,153222,253738,339488,359948,376940,1387,7003,1879,3175,7490,9839,10274,2308,23186,25803,26137,84295 | ACTB,AHR,AR,ATF3,BACH1,BCL6,CDX4,CHD2,KLF6,CREB1,CREM,DACH1,DLX5,DLX6,E2F3,EGR1,ELF1,ELK3,EMX2,EP300,ETS1,ETV1,ETV3,FOXF1,FOXF2,FOXC1,FLI1,NR5A2,GATA2,GLI2,GLI3,NR3C1,GTF2B,H3-3B,HIVEP1,HLF,HNRNPK,HNRNPU,HOXA5,HOXA7,HOXA9,HOXB7,HOXD13,RBPJ,FOXK2,INSM1,JARID2,LMO2,MXD1,SMAD6,MAF,MAFG,MEF2C,MEIS1,MEOX2,MITF,MNT,MSX1,MYBL1,MYCN,MYT1,NEUROD1,NFATC2,NFATC3,NFIB,NFIL3,NONO,NR4A2,PAX6,PBX3,PITX2,POU2F1,PPARG,PROX1,RAD23B,RARB,REST,RFX3,RORA,RORB,SALL1,SATB1,SFPQ,SNAI2,SMARCB1,SMARCD2,SP3,TBP,TBX15,TCF3,TCF7L2,ZEB1,TFAP2A,TFAP2C,TFDP2,TGIF1,THRB,NKX2-1,TOP1,NR2C2,KDM6A,YY1,ZNF131,ZNF143,ZNF148,NR4A3,NRIP1,YBX3,CGGBP1,BHLHE40,KLF7,EED,KAT2B,KLF4,LHX2,ONECUT2,TBPL1,MACROH2A1,CLOCK,ST18,ZBTB5,USP3,THRAP3,CEBPZ,PCGF3,NCOA2,TBR1,NFAT5,KLF12,MYT1L,MGA,SATB2,SUZ12,SSBP2,EHF,LHX6,RBMX,KLF15,PURG,MYEF2,KLF3,KLF13,SIX4,BCOR,THAP1,MACROH2A2,SOX6,CHD7,CCAR1,NKRF,LHX9,BACH2,NEUROG2,NEUROD6,RFX7,BCL11B,BHLHE41,E2F8,ZFHX4,TFAP2D,ARID5B,PCGF5,KDM2B,FOXP2,AEBP2,OSR1,LIN54,E2F7,GSC,ZNF827,CREBRF,EBF3,TFAP2E,IRF2BP2,ZC3H6,CREBBP,TEAD1,EBF1,ONECUT1,WT1,ZEB2,STAG1,FOXO1,RCOR1,SPDEF,ZBTB20,PHF6 |
| 2_Member | GO Molecular Functions | GO:0000977 | RNA polymerase II regulatory region sequence-specific DNA binding | -33.76811578 | -30.196 | 175/788 | 60,196,367,467,571,604,1046,1106,1316,1385,1390,1602,1749,1750,1871,1958,1997,2004,2018,2033,2113,2115,2117,2294,2295,2296,2313,2494,2624,2736,2737,2908,2959,3021,3096,3131,3190,3192,3202,3204,3205,3217,3239,3516,3607,3642,3720,4005,4084,4091,4094,4097,4208,4211,4223,4286,4335,4487,4603,4613,4661,4760,4773,4775,4781,4783,4841,4929,5080,5090,5308,5451,5468,5629,5887,5915,5978,5991,6095,6096,6299,6304,6421,6591,6598,6603,6670,6908,6913,6929,6934,6935,7020,7022,7029,7050,7068,7080,7150,7182,7403,7528,7690,7702,7707,8013,8204,8531,8545,8553,8609,8726,8850,9314,9355,9480,9519,9555,9575,9705,9925,9960,9967,10153,10336,10499,10716,10725,11278,23040,23269,23314,23512,23635,26298,26468,27316,28999,29942,50804,51274,51621,51804,54880,55145,55506,55553,55636,55749,55922,56956,60468,63973,63974,64864,64919,79365,79733,79776,83741,84159,84333,84678,93986,121536,130497,132660,144455,145258,152485,153222,253738,339488,359948,376940 | ACTB,AHR,AR,ATF3,BACH1,BCL6,CDX4,CHD2,KLF6,CREB1,CREM,DACH1,DLX5,DLX6,E2F3,EGR1,ELF1,ELK3,EMX2,EP300,ETS1,ETV1,ETV3,FOXF1,FOXF2,FOXC1,FLI1,NR5A2,GATA2,GLI2,GLI3,NR3C1,GTF2B,H3-3B,HIVEP1,HLF,HNRNPK,HNRNPU,HOXA5,HOXA7,HOXA9,HOXB7,HOXD13,RBPJ,FOXK2,INSM1,JARID2,LMO2,MXD1,SMAD6,MAF,MAFG,MEF2C,MEIS1,MEOX2,MITF,MNT,MSX1,MYBL1,MYCN,MYT1,NEUROD1,NFATC2,NFATC3,NFIB,NFIL3,NONO,NR4A2,PAX6,PBX3,PITX2,POU2F1,PPARG,PROX1,RAD23B,RARB,REST,RFX3,RORA,RORB,SALL1,SATB1,SFPQ,SNAI2,SMARCB1,SMARCD2,SP3,TBP,TBX15,TCF3,TCF7L2,ZEB1,TFAP2A,TFAP2C,TFDP2,TGIF1,THRB,NKX2-1,TOP1,NR2C2,KDM6A,YY1,ZNF131,ZNF143,ZNF148,NR4A3,NRIP1,YBX3,CGGBP1,BHLHE40,KLF7,EED,KAT2B,KLF4,LHX2,ONECUT2,TBPL1,MACROH2A1,CLOCK,ST18,ZBTB5,USP3,THRAP3,CEBPZ,PCGF3,NCOA2,TBR1,NFAT5,KLF12,MYT1L,MGA,SATB2,SUZ12,SSBP2,EHF,LHX6,RBMX,KLF15,PURG,MYEF2,KLF3,KLF13,SIX4,BCOR,THAP1,MACROH2A2,SOX6,CHD7,CCAR1,NKRF,LHX9,BACH2,NEUROG2,NEUROD6,RFX7,BCL11B,BHLHE41,E2F8,ZFHX4,TFAP2D,ARID5B,PCGF5,KDM2B,FOXP2,AEBP2,OSR1,LIN54,E2F7,GSC,ZNF827,CREBRF,EBF3,TFAP2E,IRF2BP2,ZC3H6 |
| 2_Member | GO Molecular Functions | GO:0001012 | RNA polymerase II regulatory region DNA binding | -33.32782834 | -29.822 | 175/794 | 60,196,367,467,571,604,1046,1106,1316,1385,1390,1602,1749,1750,1871,1958,1997,2004,2018,2033,2113,2115,2117,2294,2295,2296,2313,2494,2624,2736,2737,2908,2959,3021,3096,3131,3190,3192,3202,3204,3205,3217,3239,3516,3607,3642,3720,4005,4084,4091,4094,4097,4208,4211,4223,4286,4335,4487,4603,4613,4661,4760,4773,4775,4781,4783,4841,4929,5080,5090,5308,5451,5468,5629,5887,5915,5978,5991,6095,6096,6299,6304,6421,6591,6598,6603,6670,6908,6913,6929,6934,6935,7020,7022,7029,7050,7068,7080,7150,7182,7403,7528,7690,7702,7707,8013,8204,8531,8545,8553,8609,8726,8850,9314,9355,9480,9519,9555,9575,9705,9925,9960,9967,10153,10336,10499,10716,10725,11278,23040,23269,23314,23512,23635,26298,26468,27316,28999,29942,50804,51274,51621,51804,54880,55145,55506,55553,55636,55749,55922,56956,60468,63973,63974,64864,64919,79365,79733,79776,83741,84159,84333,84678,93986,121536,130497,132660,144455,145258,152485,153222,253738,339488,359948,376940 | ACTB,AHR,AR,ATF3,BACH1,BCL6,CDX4,CHD2,KLF6,CREB1,CREM,DACH1,DLX5,DLX6,E2F3,EGR1,ELF1,ELK3,EMX2,EP300,ETS1,ETV1,ETV3,FOXF1,FOXF2,FOXC1,FLI1,NR5A2,GATA2,GLI2,GLI3,NR3C1,GTF2B,H3-3B,HIVEP1,HLF,HNRNPK,HNRNPU,HOXA5,HOXA7,HOXA9,HOXB7,HOXD13,RBPJ,FOXK2,INSM1,JARID2,LMO2,MXD1,SMAD6,MAF,MAFG,MEF2C,MEIS1,MEOX2,MITF,MNT,MSX1,MYBL1,MYCN,MYT1,NEUROD1,NFATC2,NFATC3,NFIB,NFIL3,NONO,NR4A2,PAX6,PBX3,PITX2,POU2F1,PPARG,PROX1,RAD23B,RARB,REST,RFX3,RORA,RORB,SALL1,SATB1,SFPQ,SNAI2,SMARCB1,SMARCD2,SP3,TBP,TBX15,TCF3,TCF7L2,ZEB1,TFAP2A,TFAP2C,TFDP2,TGIF1,THRB,NKX2-1,TOP1,NR2C2,KDM6A,YY1,ZNF131,ZNF143,ZNF148,NR4A3,NRIP1,YBX3,CGGBP1,BHLHE40,KLF7,EED,KAT2B,KLF4,LHX2,ONECUT2,TBPL1,MACROH2A1,CLOCK,ST18,ZBTB5,USP3,THRAP3,CEBPZ,PCGF3,NCOA2,TBR1,NFAT5,KLF12,MYT1L,MGA,SATB2,SUZ12,SSBP2,EHF,LHX6,RBMX,KLF15,PURG,MYEF2,KLF3,KLF13,SIX4,BCOR,THAP1,MACROH2A2,SOX6,CHD7,CCAR1,NKRF,LHX9,BACH2,NEUROG2,NEUROD6,RFX7,BCL11B,BHLHE41,E2F8,ZFHX4,TFAP2D,ARID5B,PCGF5,KDM2B,FOXP2,AEBP2,OSR1,LIN54,E2F7,GSC,ZNF827,CREBRF,EBF3,TFAP2E,IRF2BP2,ZC3H6 |
| 2_Member | GO Molecular Functions | GO:0000987 | proximal promoter sequence-specific DNA binding | -25.90752949 | -22.733 | 128/560 | 60,196,367,467,1046,1106,1316,1385,1387,1602,1749,1750,1871,1997,2004,2018,2033,2113,2115,2313,2494,2624,2736,2737,2908,3190,3192,3202,3204,3205,3217,3239,3516,3607,3642,4005,4084,4091,4094,4097,4208,4211,4223,4286,4603,4613,4760,4773,4775,4781,4783,4841,4929,5080,5308,5451,5468,5629,5887,5978,5991,6095,6096,6299,6421,6591,6598,6603,6670,6908,6913,6929,6934,6935,7003,7020,7022,7050,7080,7150,7182,7403,7528,7702,7707,8013,8204,8553,8609,8726,9314,9355,9480,9519,9575,9705,9960,10153,10499,10716,10725,23314,23512,23635,26298,27316,28999,50804,51621,51804,55145,55553,55636,55922,63973,63974,64864,64919,79365,79733,79776,84678,93986,121536,144455,145258,253738,339488 | ACTB,AHR,AR,ATF3,CDX4,CHD2,KLF6,CREB1,CREBBP,DACH1,DLX5,DLX6,E2F3,ELF1,ELK3,EMX2,EP300,ETS1,ETV1,FLI1,NR5A2,GATA2,GLI2,GLI3,NR3C1,HNRNPK,HNRNPU,HOXA5,HOXA7,HOXA9,HOXB7,HOXD13,RBPJ,FOXK2,INSM1,LMO2,MXD1,SMAD6,MAF,MAFG,MEF2C,MEIS1,MEOX2,MITF,MYBL1,MYCN,NEUROD1,NFATC2,NFATC3,NFIB,NFIL3,NONO,NR4A2,PAX6,PITX2,POU2F1,PPARG,PROX1,RAD23B,REST,RFX3,RORA,RORB,SALL1,SFPQ,SNAI2,SMARCB1,SMARCD2,SP3,TBP,TBX15,TCF3,TCF7L2,ZEB1,TEAD1,TFAP2A,TFAP2C,TGIF1,NKX2-1,TOP1,NR2C2,KDM6A,YY1,ZNF143,ZNF148,NR4A3,NRIP1,BHLHE40,KLF7,EED,KLF4,LHX2,ONECUT2,TBPL1,CLOCK,ST18,USP3,CEBPZ,NCOA2,TBR1,NFAT5,SATB2,SUZ12,SSBP2,EHF,RBMX,KLF15,MYEF2,KLF13,SIX4,THAP1,SOX6,CHD7,NKRF,NEUROG2,NEUROD6,RFX7,BCL11B,BHLHE41,E2F8,ZFHX4,KDM2B,FOXP2,AEBP2,E2F7,GSC,EBF3,TFAP2E |
| 2_Member | GO Molecular Functions | GO:0000978 | RNA polymerase II proximal promoter sequence-specific DNA binding | -25.52724828 | -22.381 | 125/544 | 60,196,367,467,1046,1106,1316,1385,1602,1749,1750,1997,2004,2018,2033,2113,2115,2313,2494,2624,2736,2737,2908,3190,3192,3202,3204,3205,3217,3239,3516,3607,3642,4005,4084,4091,4094,4097,4208,4211,4223,4286,4603,4613,4760,4773,4775,4781,4783,4841,4929,5080,5308,5451,5468,5629,5887,5978,5991,6095,6096,6299,6421,6591,6598,6603,6670,6908,6913,6929,6934,6935,7020,7022,7050,7080,7150,7182,7403,7528,7702,7707,8013,8204,8553,8609,8726,9314,9355,9480,9519,9575,9705,9960,10153,10499,10716,10725,23314,23512,23635,26298,27316,28999,50804,51621,51804,55145,55553,55636,55922,63973,63974,64864,64919,79365,79733,79776,84678,93986,121536,144455,145258,253738,339488 | ACTB,AHR,AR,ATF3,CDX4,CHD2,KLF6,CREB1,DACH1,DLX5,DLX6,ELF1,ELK3,EMX2,EP300,ETS1,ETV1,FLI1,NR5A2,GATA2,GLI2,GLI3,NR3C1,HNRNPK,HNRNPU,HOXA5,HOXA7,HOXA9,HOXB7,HOXD13,RBPJ,FOXK2,INSM1,LMO2,MXD1,SMAD6,MAF,MAFG,MEF2C,MEIS1,MEOX2,MITF,MYBL1,MYCN,NEUROD1,NFATC2,NFATC3,NFIB,NFIL3,NONO,NR4A2,PAX6,PITX2,POU2F1,PPARG,PROX1,RAD23B,REST,RFX3,RORA,RORB,SALL1,SFPQ,SNAI2,SMARCB1,SMARCD2,SP3,TBP,TBX15,TCF3,TCF7L2,ZEB1,TFAP2A,TFAP2C,TGIF1,NKX2-1,TOP1,NR2C2,KDM6A,YY1,ZNF143,ZNF148,NR4A3,NRIP1,BHLHE40,KLF7,EED,KLF4,LHX2,ONECUT2,TBPL1,CLOCK,ST18,USP3,CEBPZ,NCOA2,TBR1,NFAT5,SATB2,SUZ12,SSBP2,EHF,RBMX,KLF15,MYEF2,KLF13,SIX4,THAP1,SOX6,CHD7,NKRF,NEUROG2,NEUROD6,RFX7,BCL11B,BHLHE41,E2F8,ZFHX4,KDM2B,FOXP2,AEBP2,E2F7,GSC,EBF3,TFAP2E |
| 2_Member | GO Molecular Functions | GO:0001228 | DNA-binding transcription activator activity, RNA polymerase II-specific | -14.70651776 | -12.377 | 89/439 | 367,467,571,1046,1316,1385,1749,1871,1879,1958,1997,2004,2033,2113,2115,2294,2295,2296,2313,2494,2624,2736,2737,2908,3131,3175,3190,3202,3204,3217,3239,3516,3607,4005,4094,4097,4208,4211,4223,4286,4487,4603,4613,4661,4760,4773,4775,4781,4929,5080,5090,5978,6095,6096,6913,7020,7022,7029,7182,7490,7690,7702,8013,8609,9314,9355,9480,9519,9575,9705,9839,10153,10274,10716,10725,23040,23269,23314,26298,28999,51621,51804,55922,63974,64919,153222,253738,339488,359948 | AR,ATF3,BACH1,CDX4,KLF6,CREB1,DLX5,E2F3,EBF1,EGR1,ELF1,ELK3,EP300,ETS1,ETV1,FOXF1,FOXF2,FOXC1,FLI1,NR5A2,GATA2,GLI2,GLI3,NR3C1,HLF,ONECUT1,HNRNPK,HOXA5,HOXA7,HOXB7,HOXD13,RBPJ,FOXK2,LMO2,MAF,MAFG,MEF2C,MEIS1,MEOX2,MITF,MSX1,MYBL1,MYCN,MYT1,NEUROD1,NFATC2,NFATC3,NFIB,NR4A2,PAX6,PBX3,REST,RORA,RORB,TBX15,TFAP2A,TFAP2C,TFDP2,NR2C2,WT1,ZNF131,ZNF143,NR4A3,KLF7,KLF4,LHX2,ONECUT2,TBPL1,CLOCK,ST18,ZEB2,CEBPZ,STAG1,TBR1,NFAT5,MYT1L,MGA,SATB2,EHF,KLF15,KLF13,SIX4,NKRF,NEUROD6,BCL11B,CREBRF,EBF3,TFAP2E,IRF2BP2 |
| 2_Member | GO Molecular Functions | GO:0001227 | DNA-binding transcription repressor activity, RNA polymerase II-specific | -13.54023751 | -11.304 | 59/242 | 467,571,604,1390,1602,2004,2018,2117,2308,3096,3607,3642,3720,4084,4286,4335,4487,4773,4775,4783,5080,5468,5629,5978,6299,6304,6591,6670,6913,6929,6935,7020,7022,7050,7528,7690,7707,8531,8545,8553,9839,9925,11278,23040,23186,25803,26137,29942,55145,55553,60468,79365,79733,84159,84295,93986,121536,144455,145258 | ATF3,BACH1,BCL6,CREM,DACH1,ELK3,EMX2,ETV3,FOXO1,HIVEP1,FOXK2,INSM1,JARID2,MXD1,MITF,MNT,MSX1,NFATC2,NFATC3,NFIL3,PAX6,PPARG,PROX1,REST,SALL1,SATB1,SNAI2,SP3,TBX15,TCF3,ZEB1,TFAP2A,TFAP2C,TGIF1,YY1,ZNF131,ZNF148,YBX3,CGGBP1,BHLHE40,ZEB2,ZBTB5,KLF12,MYT1L,RCOR1,SPDEF,ZBTB20,PURG,THAP1,SOX6,BACH2,BHLHE41,E2F8,ARID5B,PHF6,FOXP2,AEBP2,E2F7,GSC |
| 3_Summary | GO Biological Processes | GO:0060322 | head development | -28.90128609 | -25.551 | 164/777 | 18,60,357,372,546,596,650,657,659,664,998,1272,1385,1399,1499,1749,1750,1808,2018,2033,2044,2045,2048,2049,2066,2113,2145,2181,2186,2255,2258,2263,2296,2334,2624,2736,2737,2895,2903,3021,3084,3184,3251,3309,3516,3624,3688,3746,3747,3799,3845,3925,3985,4036,4040,4487,4628,4760,4781,4929,5048,5049,5080,5156,5270,5308,5530,5592,5594,5629,5727,5728,5764,5793,5915,6091,6092,6095,6299,6310,6331,6497,6506,6538,6546,6547,6857,6860,7048,7080,7314,7337,7531,7707,7804,8013,8289,8455,8660,8829,8854,8867,8925,9201,9231,9355,9693,9839,9919,10000,10159,10371,10395,10716,10818,10891,10971,22903,22931,22941,23314,23543,23767,26468,27164,27185,28514,29072,54407,54510,54806,55219,55506,55636,55773,57282,57453,57526,57626,57688,58155,63974,64919,65009,79633,80031,83690,83741,84002,84159,84376,84678,84879,85458,93986,136319,145258,146057,146760,152330,153090,317762,342371,388662 | ABAT,ACTB,SHROOM2,ARCN1,ATRX,BCL2,BMP2,BMPR1A,BMPR2,BNIP3,CDC42,CNTN1,CREB1,CRKL,CTNNB1,DLX5,DLX6,DPYSL2,EMX2,EP300,EPHA5,EPHA7,EPHB2,EPHB3,ERBB4,ETS1,EZH1,ACSL3,BPTF,FGF10,FGF13,FGFR2,FOXC1,AFF2,GATA2,GLI2,GLI3,GRID2,GRIN2A,H3-3B,NRG1,HNRNPD,HPRT1,HSPA5,RBPJ,INHBA,ITGB1,KCNC1,KCNC2,KIF5B,KRAS,STMN1,LIMK2,LRP2,LRP6,MSX1,MYH10,NEUROD1,NFIB,NR4A2,PAFAH1B1,PAFAH1B2,PAX6,PDGFRA,SERPINE2,PITX2,PPP3CA,PRKG1,MAPK1,PROX1,PTCH1,PTEN,PTN,PTPRG,RARB,ROBO1,ROBO2,RORA,SALL1,ATXN1,SCN5A,SKI,SLC1A2,SLC6A11,SLC8A1,SLC8A3,SYT1,SYT4,TGFBR2,NKX2-1,UBB,UBE3A,YWHAE,ZNF148,LRP8,NR4A3,ARID1A,ATRN,IRS2,NRP1,ALDH1A2,SYNJ1,HERC1,DCLK1,DLG5,LHX2,RAPGEF2,ZEB2,SEC16A,AKT3,ATP6AP2,SEMA3A,DLC1,TBR1,FRS2,PPARGC1A,YWHAQ,BTBD3,RAB18,SHANK2,SATB2,RBFOX2,FLRT3,LHX6,SALL3,DISC1,DLL1,SETD2,SLC38A2,PCDH18,AHI1,MACO1,MACROH2A2,CHD7,TBC1D23,SLC4A10,DSCAML1,PCDH19,KLHL1,ZSWIM6,PTBP2,NEUROD6,BCL11B,NDRG4,FAT4,SEMA6D,CRISPLD1,TFAP2D,B3GNT5,ARID5B,HOOK3,KDM2B,MFSD2A,DIXDC1,FOXP2,MTPN,GSC,TTBK2,RTN4RL1,CNTN4,DAB2IP,CCDC85C,ATXN1L,SLC6A17 |
| 3_Member | GO Biological Processes | GO:0060322 | head development | -28.90128609 | -25.551 | 164/777 | 18,60,357,372,546,596,650,657,659,664,998,1272,1385,1399,1499,1749,1750,1808,2018,2033,2044,2045,2048,2049,2066,2113,2145,2181,2186,2255,2258,2263,2296,2334,2624,2736,2737,2895,2903,3021,3084,3184,3251,3309,3516,3624,3688,3746,3747,3799,3845,3925,3985,4036,4040,4487,4628,4760,4781,4929,5048,5049,5080,5156,5270,5308,5530,5592,5594,5629,5727,5728,5764,5793,5915,6091,6092,6095,6299,6310,6331,6497,6506,6538,6546,6547,6857,6860,7048,7080,7314,7337,7531,7707,7804,8013,8289,8455,8660,8829,8854,8867,8925,9201,9231,9355,9693,9839,9919,10000,10159,10371,10395,10716,10818,10891,10971,22903,22931,22941,23314,23543,23767,26468,27164,27185,28514,29072,54407,54510,54806,55219,55506,55636,55773,57282,57453,57526,57626,57688,58155,63974,64919,65009,79633,80031,83690,83741,84002,84159,84376,84678,84879,85458,93986,136319,145258,146057,146760,152330,153090,317762,342371,388662 | ABAT,ACTB,SHROOM2,ARCN1,ATRX,BCL2,BMP2,BMPR1A,BMPR2,BNIP3,CDC42,CNTN1,CREB1,CRKL,CTNNB1,DLX5,DLX6,DPYSL2,EMX2,EP300,EPHA5,EPHA7,EPHB2,EPHB3,ERBB4,ETS1,EZH1,ACSL3,BPTF,FGF10,FGF13,FGFR2,FOXC1,AFF2,GATA2,GLI2,GLI3,GRID2,GRIN2A,H3-3B,NRG1,HNRNPD,HPRT1,HSPA5,RBPJ,INHBA,ITGB1,KCNC1,KCNC2,KIF5B,KRAS,STMN1,LIMK2,LRP2,LRP6,MSX1,MYH10,NEUROD1,NFIB,NR4A2,PAFAH1B1,PAFAH1B2,PAX6,PDGFRA,SERPINE2,PITX2,PPP3CA,PRKG1,MAPK1,PROX1,PTCH1,PTEN,PTN,PTPRG,RARB,ROBO1,ROBO2,RORA,SALL1,ATXN1,SCN5A,SKI,SLC1A2,SLC6A11,SLC8A1,SLC8A3,SYT1,SYT4,TGFBR2,NKX2-1,UBB,UBE3A,YWHAE,ZNF148,LRP8,NR4A3,ARID1A,ATRN,IRS2,NRP1,ALDH1A2,SYNJ1,HERC1,DCLK1,DLG5,LHX2,RAPGEF2,ZEB2,SEC16A,AKT3,ATP6AP2,SEMA3A,DLC1,TBR1,FRS2,PPARGC1A,YWHAQ,BTBD3,RAB18,SHANK2,SATB2,RBFOX2,FLRT3,LHX6,SALL3,DISC1,DLL1,SETD2,SLC38A2,PCDH18,AHI1,MACO1,MACROH2A2,CHD7,TBC1D23,SLC4A10,DSCAML1,PCDH19,KLHL1,ZSWIM6,PTBP2,NEUROD6,BCL11B,NDRG4,FAT4,SEMA6D,CRISPLD1,TFAP2D,B3GNT5,ARID5B,HOOK3,KDM2B,MFSD2A,DIXDC1,FOXP2,MTPN,GSC,TTBK2,RTN4RL1,CNTN4,DAB2IP,CCDC85C,ATXN1L,SLC6A17 |
| 3_Member | GO Biological Processes | GO:0007420 | brain development | -27.33097552 | -24.127 | 155/734 | 18,60,357,372,546,596,650,657,659,664,998,1272,1385,1399,1499,1749,1808,2018,2044,2045,2048,2049,2066,2113,2145,2181,2186,2255,2258,2263,2296,2334,2624,2736,2737,2895,2903,3021,3084,3184,3251,3309,3516,3624,3688,3746,3747,3799,3845,3925,4036,4040,4487,4628,4760,4781,4929,5048,5049,5080,5270,5308,5530,5592,5629,5727,5728,5764,5793,5915,6091,6092,6095,6299,6310,6331,6497,6506,6538,6546,6547,6857,6860,7048,7080,7314,7337,7531,7707,7804,8013,8289,8455,8660,8829,8854,8867,8925,9201,9231,9355,9693,9839,9919,10000,10371,10395,10716,10818,10891,10971,22903,22931,22941,23314,23543,26468,27164,27185,28514,29072,54407,54510,54806,55219,55506,55636,55773,57282,57453,57526,57626,57688,58155,63974,64919,65009,79633,80031,83741,84002,84376,84678,84879,85458,93986,136319,145258,146057,146760,152330,153090,317762,342371,388662 | ABAT,ACTB,SHROOM2,ARCN1,ATRX,BCL2,BMP2,BMPR1A,BMPR2,BNIP3,CDC42,CNTN1,CREB1,CRKL,CTNNB1,DLX5,DPYSL2,EMX2,EPHA5,EPHA7,EPHB2,EPHB3,ERBB4,ETS1,EZH1,ACSL3,BPTF,FGF10,FGF13,FGFR2,FOXC1,AFF2,GATA2,GLI2,GLI3,GRID2,GRIN2A,H3-3B,NRG1,HNRNPD,HPRT1,HSPA5,RBPJ,INHBA,ITGB1,KCNC1,KCNC2,KIF5B,KRAS,STMN1,LRP2,LRP6,MSX1,MYH10,NEUROD1,NFIB,NR4A2,PAFAH1B1,PAFAH1B2,PAX6,SERPINE2,PITX2,PPP3CA,PRKG1,PROX1,PTCH1,PTEN,PTN,PTPRG,RARB,ROBO1,ROBO2,RORA,SALL1,ATXN1,SCN5A,SKI,SLC1A2,SLC6A11,SLC8A1,SLC8A3,SYT1,SYT4,TGFBR2,NKX2-1,UBB,UBE3A,YWHAE,ZNF148,LRP8,NR4A3,ARID1A,ATRN,IRS2,NRP1,ALDH1A2,SYNJ1,HERC1,DCLK1,DLG5,LHX2,RAPGEF2,ZEB2,SEC16A,AKT3,SEMA3A,DLC1,TBR1,FRS2,PPARGC1A,YWHAQ,BTBD3,RAB18,SHANK2,SATB2,RBFOX2,LHX6,SALL3,DISC1,DLL1,SETD2,SLC38A2,PCDH18,AHI1,MACO1,MACROH2A2,CHD7,TBC1D23,SLC4A10,DSCAML1,PCDH19,KLHL1,ZSWIM6,PTBP2,NEUROD6,BCL11B,NDRG4,FAT4,SEMA6D,TFAP2D,B3GNT5,HOOK3,KDM2B,MFSD2A,DIXDC1,FOXP2,MTPN,GSC,TTBK2,RTN4RL1,CNTN4,DAB2IP,CCDC85C,ATXN1L,SLC6A17 |
| 3_Member | GO Biological Processes | GO:0030900 | forebrain development | -19.8117584 | -17.105 | 91/382 | 546,650,657,664,1385,1399,1499,1749,2018,2044,2048,2049,2066,2113,2145,2255,2258,2263,2624,2736,2737,3084,3251,3516,3624,3746,3747,3799,3845,4036,4040,4487,4628,4760,4781,4929,5048,5080,5308,5592,5629,5728,5764,5915,6091,6092,6299,6331,6497,6506,6546,6547,7080,7314,7531,7804,8013,8289,8829,8854,9201,9355,9693,9839,10371,10395,10716,10818,10891,22903,23314,26468,27164,27185,29072,54407,55636,57282,57688,63974,64919,79633,84376,84678,84879,85458,93986,145258,146760,153090,317762 | ATRX,BMP2,BMPR1A,BNIP3,CREB1,CRKL,CTNNB1,DLX5,EMX2,EPHA5,EPHB2,EPHB3,ERBB4,ETS1,EZH1,FGF10,FGF13,FGFR2,GATA2,GLI2,GLI3,NRG1,HPRT1,RBPJ,INHBA,KCNC1,KCNC2,KIF5B,KRAS,LRP2,LRP6,MSX1,MYH10,NEUROD1,NFIB,NR4A2,PAFAH1B1,PAX6,PITX2,PRKG1,PROX1,PTEN,PTN,RARB,ROBO1,ROBO2,SALL1,SCN5A,SKI,SLC1A2,SLC8A1,SLC8A3,NKX2-1,UBB,YWHAE,LRP8,NR4A3,ARID1A,NRP1,ALDH1A2,DCLK1,LHX2,RAPGEF2,ZEB2,SEMA3A,DLC1,TBR1,FRS2,PPARGC1A,BTBD3,SATB2,LHX6,SALL3,DISC1,SETD2,SLC38A2,CHD7,SLC4A10,ZSWIM6,NEUROD6,BCL11B,FAT4,HOOK3,KDM2B,MFSD2A,DIXDC1,FOXP2,GSC,RTN4RL1,DAB2IP,CCDC85C |
| 3_Member | GO Biological Processes | GO:0021537 | telencephalon development | -13.18446551 | -10.961 | 60/253 | 650,664,1399,1499,1749,2018,2044,2048,2049,2066,2145,2258,2737,3084,3251,3624,3799,4040,4628,4760,4781,5048,5080,5629,5728,5915,6091,6092,6299,6331,6497,6506,6546,6547,7080,7531,7804,8013,8829,9355,9839,10371,10716,22903,26468,27164,27185,54407,55636,57688,63974,64919,79633,84678,84879,85458,93986,146760,153090,317762 | BMP2,BNIP3,CRKL,CTNNB1,DLX5,EMX2,EPHA5,EPHB2,EPHB3,ERBB4,EZH1,FGF13,GLI3,NRG1,HPRT1,INHBA,KIF5B,LRP6,MYH10,NEUROD1,NFIB,PAFAH1B1,PAX6,PROX1,PTEN,RARB,ROBO1,ROBO2,SALL1,SCN5A,SKI,SLC1A2,SLC8A1,SLC8A3,NKX2-1,YWHAE,LRP8,NR4A3,NRP1,LHX2,ZEB2,SEMA3A,TBR1,BTBD3,LHX6,SALL3,DISC1,SLC38A2,CHD7,ZSWIM6,NEUROD6,BCL11B,FAT4,KDM2B,MFSD2A,DIXDC1,FOXP2,RTN4RL1,DAB2IP,CCDC85C |
| 3_Member | GO Biological Processes | GO:0021543 | pallium development | -6.71234425 | -5.036 | 36/172 | 664,1399,1499,2018,2044,2145,2258,2737,3799,4040,4760,5048,5080,5629,5728,6091,7080,7531,7804,8013,8829,9355,9839,10371,10716,22903,26468,27185,54407,63974,79633,84879,85458,93986,153090,317762 | BNIP3,CRKL,CTNNB1,EMX2,EPHA5,EZH1,FGF13,GLI3,KIF5B,LRP6,NEUROD1,PAFAH1B1,PAX6,PROX1,PTEN,ROBO1,NKX2-1,YWHAE,LRP8,NR4A3,NRP1,LHX2,ZEB2,SEMA3A,TBR1,BTBD3,LHX6,DISC1,SLC38A2,NEUROD6,FAT4,MFSD2A,DIXDC1,FOXP2,DAB2IP,CCDC85C |
| 3_Member | GO Biological Processes | GO:0021987 | cerebral cortex development | -5.61151284 | -4.079 | 26/116 | 664,1399,1499,2018,2258,2737,4040,5048,5080,6091,7080,7531,7804,8829,9355,10371,10716,22903,26468,27185,54407,79633,85458,93986,153090,317762 | BNIP3,CRKL,CTNNB1,EMX2,FGF13,GLI3,LRP6,PAFAH1B1,PAX6,ROBO1,NKX2-1,YWHAE,LRP8,NRP1,LHX2,SEMA3A,TBR1,BTBD3,LHX6,DISC1,SLC38A2,FAT4,DIXDC1,FOXP2,DAB2IP,CCDC85C |
| 3_Member | GO Biological Processes | GO:0021761 | limbic system development | -5.089393797 | -3.618 | 24/109 | 1399,2018,2044,2113,2145,2258,2263,2737,3799,4760,5048,5629,5728,7080,7314,7531,7804,8013,8829,9839,10371,10716,63974,84879 | CRKL,EMX2,EPHA5,ETS1,EZH1,FGF13,FGFR2,GLI3,KIF5B,NEUROD1,PAFAH1B1,PROX1,PTEN,NKX2-1,UBB,YWHAE,LRP8,NR4A3,NRP1,ZEB2,SEMA3A,TBR1,NEUROD6,MFSD2A |
| 3_Member | GO Biological Processes | GO:0021766 | hippocampus development | -4.028590842 | -2.696 | 18/81 | 1399,2018,2044,2145,2258,2737,3799,4760,5048,5629,5728,7080,7531,7804,8013,9839,63974,84879 | CRKL,EMX2,EPHA5,EZH1,FGF13,GLI3,KIF5B,NEUROD1,PAFAH1B1,PROX1,PTEN,NKX2-1,YWHAE,LRP8,NR4A3,ZEB2,NEUROD6,MFSD2A |
| 3_Member | GO Biological Processes | GO:0021795 | cerebral cortex cell migration | -3.854659947 | -2.551 | 12/43 | 1499,2258,2737,5048,6091,7080,8829,10371,26468,27185,85458,153090 | CTNNB1,FGF13,GLI3,PAFAH1B1,ROBO1,NKX2-1,NRP1,SEMA3A,LHX6,DISC1,DIXDC1,DAB2IP |
| 3_Member | GO Biological Processes | GO:0021885 | forebrain cell migration | -3.415585805 | -2.189 | 14/61 | 1499,2018,2258,2737,3084,5048,6091,7080,8829,10371,26468,27185,85458,153090 | CTNNB1,EMX2,FGF13,GLI3,NRG1,PAFAH1B1,ROBO1,NKX2-1,NRP1,SEMA3A,LHX6,DISC1,DIXDC1,DAB2IP |
| 3_Member | GO Biological Processes | GO:0022029 | telencephalon cell migration | -3.107646396 | -1.940 | 13/58 | 1499,2258,2737,3084,5048,6091,7080,8829,10371,26468,27185,85458,153090 | CTNNB1,FGF13,GLI3,NRG1,PAFAH1B1,ROBO1,NKX2-1,NRP1,SEMA3A,LHX6,DISC1,DIXDC1,DAB2IP |
| 4_Summary | GO Cellular Components | GO:0098794 | postsynapse | -28.82863273 | -25.519 | 141/613 | 60,102,288,320,321,375,659,664,775,793,998,1007,1129,1211,1212,1272,1399,1499,1739,1740,1741,1759,1977,1994,2043,2045,2048,2060,2066,2332,2554,2742,2743,2823,2891,2895,2898,2900,2903,2911,2913,2915,3064,3688,3708,3717,3745,3746,3747,3756,3759,3781,4131,4133,4208,4628,4842,4897,4916,5028,5062,5170,5332,5530,5577,5590,5594,5727,5728,5764,5867,5868,5879,6009,6457,6529,6538,6546,6547,6616,6622,6711,6717,6801,6812,7402,7804,8087,8301,8829,8943,9101,9201,9231,9456,9568,9758,9829,9863,10142,10564,10814,22849,22871,22883,22941,23191,23345,23362,23373,23613,23767,26045,26052,26115,27020,27185,27253,27445,53616,54476,54477,55619,55737,56899,57120,57534,57554,58489,64506,80059,80315,81831,81832,91752,94056,115207,140735,149111,220988,347731,87,1268,1488,1607,2239,3084,5520,5573,5789,6506,6780,6857,6860,7531,7534,8618,9211,10082,10397,11252,81873,84631,84961,388662,108,115,627,657,774,1826,2044,2049,2258,2781,2782,3192,3800,4036,4076,4139,4140,5563,5599,5796,6416,6421,7855,8725,9312,9532,10371,10479,10752,10891,23046,23162,23394,23405,23774,24141,27252,29966,54407,54464,57282,57580,57626,92737,153090,342371,776,2572,8573,8867,9627,9699,9892,22999,23085,23390,53942,134957,9465,11069,59,2182,3267,4040,5048,5066,5792,6323,6507,7042,7150,7314,7430,7432,8874,9590,9693,10146,10159,10846,10857,11113,22853,23001,23032,57154,57565,57605,58155,89848,135138,140767,143187,146760,8411 | ACTB,ADAM10,ANK3,APBA1,APBA2,ARF1,BMPR2,BNIP3,CACNA1C,CALB1,CDC42,CDH9,CHRM2,CLTA,CLTB,CNTN1,CRKL,CTNNB1,DLG1,DLG2,DLG3,DNM1,EIF4E,ELAVL1,EPHA4,EPHA7,EPHB2,EPS15,ERBB4,FMR1,GABRA1,GLRA2,GLRB,GPM6A,GRIA2,GRID2,GRIK2,GRIK4,GRIN2A,GRM1,GRM3,GRM5,HTT,ITGB1,ITPR1,JAK2,KCNB1,KCNC1,KCNC2,KCNH1,KCNJ2,KCNN2,MAP1B,MAP2,MEF2C,MYH10,NOS1,NRCAM,NTRK3,P2RY1,PAK2,PDPK1,PLCB4,PPP3CA,PRKAR2B,PRKCZ,MAPK1,PTCH1,PTEN,PTN,RAB4A,RAB5A,RAC1,RHEB,SH3GL3,SLC6A1,SLC6A11,SLC8A1,SLC8A3,SNAP25,SNCA,SPTBN1,SRI,STRN,STXBP1,UTRN,LRP8,FXR1,PICALM,NRP1,AP3D1,USP8,DCLK1,DLG5,HOMER1,GABBR2,FRMPD4,DNAJC6,MAGI2,AKAP9,ARFGEF2,CPLX2,CPEB3,NLGN1,CLSTN1,SHANK2,CYFIP1,SYNE1,PSD3,CRTC1,ZMYND8,FLRT3,LRRTM2,DNM3,TANC2,NPTN,DISC1,PCDH17,PCLO,ADAM22,RNF216,PLEKHA5,DOCK10,VPS35,ANKS1B,GOPC,MIB1,LRRC7,ABHD17C,CPEB1,LRRTM4,CPEB4,NETO2,NETO1,ZNF804A,SYAP1,KCTD12,DYNLL2,CNIH3,HNRNPA3,LRRTM3,ACTN1,CNR1,CTBP2,DGKB,GPC4,NRG1,PPP2R2A,PRKAR1A,PTPRD,SLC1A2,STAU1,SYT1,SYT4,YWHAE,YWHAZ,CADPS,LGI1,GPC6,NDRG1,PACSIN2,ARPC5L,SLITRK2,FBXL20,SLC6A17,ADCY2,ADCY9,BDNF,BMPR1A,CACNA1B,DSCAM,EPHA5,EPHB3,FGF13,GNAZ,GNB1,HNRNPU,KIF5C,LRP2,CAPRIN1,MARK1,MARK3,PRKAA2,MAPK8,PTPRK,MAP2K4,SFPQ,FZD5,URI1,KCNB2,BAG2,SEMA3A,SLC9A6,CHL1,PPARGC1A,KIF21B,MAPK8IP3,ADNP,DICER1,BRD1,LAMP5,KLHL20,STRN3,SLC38A2,XRN1,SLC4A10,PREX1,KLHL1,DNER,DAB2IP,ATXN1L,CACNA1D,GAD2,CASK,SYNJ1,SNCAIP,RIMS2,SNAP91,RIMS1,ERC1,ZDHHC17,CNTN5,STXBP5,AKAP7,RAPGEF4,ACTA2,ACSL4,AGFG1,LRP6,PAFAH1B1,PAM,PTPRF,SCN1A,SLC1A3,TGFB2,TOP1,UBB,EZR,VIP,ARHGEF7,AKAP12,RAPGEF2,G3BP1,ATP6AP2,PDE10A,PGRMC1,CIT,LMTK2,WDFY3,USP33,SMURF1,KLHL14,PITPNM2,PTBP2,FCHSD1,PACRG,NRSN1,VTI1A,RTN4RL1,EEA1 |
| 4_Member | GO Cellular Components | GO:0098794 | postsynapse | -28.82863273 | -25.519 | 141/613 | 60,102,288,320,321,375,659,664,775,793,998,1007,1129,1211,1212,1272,1399,1499,1739,1740,1741,1759,1977,1994,2043,2045,2048,2060,2066,2332,2554,2742,2743,2823,2891,2895,2898,2900,2903,2911,2913,2915,3064,3688,3708,3717,3745,3746,3747,3756,3759,3781,4131,4133,4208,4628,4842,4897,4916,5028,5062,5170,5332,5530,5577,5590,5594,5727,5728,5764,5867,5868,5879,6009,6457,6529,6538,6546,6547,6616,6622,6711,6717,6801,6812,7402,7804,8087,8301,8829,8943,9101,9201,9231,9456,9568,9758,9829,9863,10142,10564,10814,22849,22871,22883,22941,23191,23345,23362,23373,23613,23767,26045,26052,26115,27020,27185,27253,27445,53616,54476,54477,55619,55737,56899,57120,57534,57554,58489,64506,80059,80315,81831,81832,91752,94056,115207,140735,149111,220988,347731 | ACTB,ADAM10,ANK3,APBA1,APBA2,ARF1,BMPR2,BNIP3,CACNA1C,CALB1,CDC42,CDH9,CHRM2,CLTA,CLTB,CNTN1,CRKL,CTNNB1,DLG1,DLG2,DLG3,DNM1,EIF4E,ELAVL1,EPHA4,EPHA7,EPHB2,EPS15,ERBB4,FMR1,GABRA1,GLRA2,GLRB,GPM6A,GRIA2,GRID2,GRIK2,GRIK4,GRIN2A,GRM1,GRM3,GRM5,HTT,ITGB1,ITPR1,JAK2,KCNB1,KCNC1,KCNC2,KCNH1,KCNJ2,KCNN2,MAP1B,MAP2,MEF2C,MYH10,NOS1,NRCAM,NTRK3,P2RY1,PAK2,PDPK1,PLCB4,PPP3CA,PRKAR2B,PRKCZ,MAPK1,PTCH1,PTEN,PTN,RAB4A,RAB5A,RAC1,RHEB,SH3GL3,SLC6A1,SLC6A11,SLC8A1,SLC8A3,SNAP25,SNCA,SPTBN1,SRI,STRN,STXBP1,UTRN,LRP8,FXR1,PICALM,NRP1,AP3D1,USP8,DCLK1,DLG5,HOMER1,GABBR2,FRMPD4,DNAJC6,MAGI2,AKAP9,ARFGEF2,CPLX2,CPEB3,NLGN1,CLSTN1,SHANK2,CYFIP1,SYNE1,PSD3,CRTC1,ZMYND8,FLRT3,LRRTM2,DNM3,TANC2,NPTN,DISC1,PCDH17,PCLO,ADAM22,RNF216,PLEKHA5,DOCK10,VPS35,ANKS1B,GOPC,MIB1,LRRC7,ABHD17C,CPEB1,LRRTM4,CPEB4,NETO2,NETO1,ZNF804A,SYAP1,KCTD12,DYNLL2,CNIH3,HNRNPA3,LRRTM3 |
| 4_Member | GO Cellular Components | GO:0098978 | glutamatergic synapse | -20.93084332 | -18.124 | 88/349 | 60,87,102,320,375,793,1129,1268,1488,1607,1739,1740,1741,1759,1977,1994,2043,2045,2048,2060,2066,2239,2823,2895,2898,2903,2911,2913,3084,3688,3717,4628,4897,4916,5028,5062,5332,5520,5530,5573,5577,5590,5789,5879,6457,6506,6616,6711,6780,6812,6857,6860,7531,7534,8087,8618,8829,8943,9101,9211,9456,9758,10082,10142,10397,10814,11252,22871,22883,23373,23767,26045,26052,26115,27020,27253,53616,54476,54477,55737,58489,81832,81873,84631,84961,140735,347731,388662 | ACTB,ACTN1,ADAM10,APBA1,ARF1,CALB1,CHRM2,CNR1,CTBP2,DGKB,DLG1,DLG2,DLG3,DNM1,EIF4E,ELAVL1,EPHA4,EPHA7,EPHB2,EPS15,ERBB4,GPC4,GPM6A,GRID2,GRIK2,GRIN2A,GRM1,GRM3,NRG1,ITGB1,JAK2,MYH10,NRCAM,NTRK3,P2RY1,PAK2,PLCB4,PPP2R2A,PPP3CA,PRKAR1A,PRKAR2B,PRKCZ,PTPRD,RAC1,SH3GL3,SLC1A2,SNAP25,SPTBN1,STAU1,STXBP1,SYT1,SYT4,YWHAE,YWHAZ,FXR1,CADPS,NRP1,AP3D1,USP8,LGI1,HOMER1,FRMPD4,GPC6,AKAP9,NDRG1,CPLX2,PACSIN2,NLGN1,CLSTN1,CRTC1,FLRT3,LRRTM2,DNM3,TANC2,NPTN,PCDH17,ADAM22,RNF216,PLEKHA5,VPS35,ABHD17C,NETO1,ARPC5L,SLITRK2,FBXL20,DYNLL2,LRRTM3,SLC6A17 |
| 4_Member | GO Cellular Components | GO:0030425 | dendrite | -20.25117822 | -17.492 | 125/620 | 102,108,115,288,320,321,627,657,659,664,774,775,793,998,1129,1741,1759,1826,2043,2044,2045,2048,2049,2258,2332,2554,2743,2781,2782,2823,2891,2895,2898,2903,2911,2913,3064,3192,3688,3745,3746,3747,3756,3759,3781,3800,4036,4076,4131,4133,4139,4140,4628,4842,5028,5332,5530,5563,5577,5594,5599,5727,5728,5796,5868,5879,6416,6421,6546,6547,6717,6780,6801,6860,7804,7855,8087,8725,9101,9312,9456,9532,9758,9863,10142,10371,10479,10564,10752,10814,10891,22849,22871,22883,22941,23046,23162,23191,23373,23394,23405,23613,23774,24141,26052,27020,27252,29966,54407,54464,55619,56899,57120,57282,57580,57626,58489,64506,80315,91752,92737,94056,149111,153090,342371 | ADAM10,ADCY2,ADCY9,ANK3,APBA1,APBA2,BDNF,BMPR1A,BMPR2,BNIP3,CACNA1B,CACNA1C,CALB1,CDC42,CHRM2,DLG3,DNM1,DSCAM,EPHA4,EPHA5,EPHA7,EPHB2,EPHB3,FGF13,FMR1,GABRA1,GLRB,GNAZ,GNB1,GPM6A,GRIA2,GRID2,GRIK2,GRIN2A,GRM1,GRM3,HTT,HNRNPU,ITGB1,KCNB1,KCNC1,KCNC2,KCNH1,KCNJ2,KCNN2,KIF5C,LRP2,CAPRIN1,MAP1B,MAP2,MARK1,MARK3,MYH10,NOS1,P2RY1,PLCB4,PPP3CA,PRKAA2,PRKAR2B,MAPK1,MAPK8,PTCH1,PTEN,PTPRK,RAB5A,RAC1,MAP2K4,SFPQ,SLC8A1,SLC8A3,SRI,STAU1,STRN,SYT4,LRP8,FZD5,FXR1,URI1,USP8,KCNB2,HOMER1,BAG2,FRMPD4,MAGI2,AKAP9,SEMA3A,SLC9A6,ARFGEF2,CHL1,CPLX2,PPARGC1A,CPEB3,NLGN1,CLSTN1,SHANK2,KIF21B,MAPK8IP3,CYFIP1,CRTC1,ADNP,DICER1,ZMYND8,BRD1,LAMP5,DNM3,NPTN,KLHL20,STRN3,SLC38A2,XRN1,DOCK10,ANKS1B,GOPC,SLC4A10,PREX1,KLHL1,ABHD17C,CPEB1,CPEB4,ZNF804A,DNER,SYAP1,CNIH3,DAB2IP,ATXN1L |
| 4_Member | GO Cellular Components | GO:0097447 | dendritic tree | -20.12757105 | -17.406 | 125/622 | 102,108,115,288,320,321,627,657,659,664,774,775,793,998,1129,1741,1759,1826,2043,2044,2045,2048,2049,2258,2332,2554,2743,2781,2782,2823,2891,2895,2898,2903,2911,2913,3064,3192,3688,3745,3746,3747,3756,3759,3781,3800,4036,4076,4131,4133,4139,4140,4628,4842,5028,5332,5530,5563,5577,5594,5599,5727,5728,5796,5868,5879,6416,6421,6546,6547,6717,6780,6801,6860,7804,7855,8087,8725,9101,9312,9456,9532,9758,9863,10142,10371,10479,10564,10752,10814,10891,22849,22871,22883,22941,23046,23162,23191,23373,23394,23405,23613,23774,24141,26052,27020,27252,29966,54407,54464,55619,56899,57120,57282,57580,57626,58489,64506,80315,91752,92737,94056,149111,153090,342371 | ADAM10,ADCY2,ADCY9,ANK3,APBA1,APBA2,BDNF,BMPR1A,BMPR2,BNIP3,CACNA1B,CACNA1C,CALB1,CDC42,CHRM2,DLG3,DNM1,DSCAM,EPHA4,EPHA5,EPHA7,EPHB2,EPHB3,FGF13,FMR1,GABRA1,GLRB,GNAZ,GNB1,GPM6A,GRIA2,GRID2,GRIK2,GRIN2A,GRM1,GRM3,HTT,HNRNPU,ITGB1,KCNB1,KCNC1,KCNC2,KCNH1,KCNJ2,KCNN2,KIF5C,LRP2,CAPRIN1,MAP1B,MAP2,MARK1,MARK3,MYH10,NOS1,P2RY1,PLCB4,PPP3CA,PRKAA2,PRKAR2B,MAPK1,MAPK8,PTCH1,PTEN,PTPRK,RAB5A,RAC1,MAP2K4,SFPQ,SLC8A1,SLC8A3,SRI,STAU1,STRN,SYT4,LRP8,FZD5,FXR1,URI1,USP8,KCNB2,HOMER1,BAG2,FRMPD4,MAGI2,AKAP9,SEMA3A,SLC9A6,ARFGEF2,CHL1,CPLX2,PPARGC1A,CPEB3,NLGN1,CLSTN1,SHANK2,KIF21B,MAPK8IP3,CYFIP1,CRTC1,ADNP,DICER1,ZMYND8,BRD1,LAMP5,DNM3,NPTN,KLHL20,STRN3,SLC38A2,XRN1,DOCK10,ANKS1B,GOPC,SLC4A10,PREX1,KLHL1,ABHD17C,CPEB1,CPEB4,ZNF804A,DNER,SYAP1,CNIH3,DAB2IP,ATXN1L |
| 4_Member | GO Cellular Components | GO:0097060 | synaptic membrane | -19.14476657 | -16.510 | 97/432 | 102,288,320,375,775,776,1007,1129,1211,1212,1268,1272,1399,1499,1739,1740,1741,1759,2043,2045,2048,2066,2239,2332,2554,2572,2742,2743,2823,2891,2895,2898,2900,2903,2911,2913,2915,3688,3745,3746,3747,3756,4897,4916,5028,5728,6506,6529,6538,6546,6547,6616,6801,6812,6857,7402,8301,8573,8829,8867,9231,9456,9568,9627,9699,9892,10142,22849,22871,22883,22941,22999,23085,23345,23362,23390,23767,26045,26052,27020,27185,27253,53616,53942,56899,57120,57554,58489,64506,80059,80315,81832,94056,115207,134957,149111,347731 | ADAM10,ANK3,APBA1,ARF1,CACNA1C,CACNA1D,CDH9,CHRM2,CLTA,CLTB,CNR1,CNTN1,CRKL,CTNNB1,DLG1,DLG2,DLG3,DNM1,EPHA4,EPHA7,EPHB2,ERBB4,GPC4,FMR1,GABRA1,GAD2,GLRA2,GLRB,GPM6A,GRIA2,GRID2,GRIK2,GRIK4,GRIN2A,GRM1,GRM3,GRM5,ITGB1,KCNB1,KCNC1,KCNC2,KCNH1,NRCAM,NTRK3,P2RY1,PTEN,SLC1A2,SLC6A1,SLC6A11,SLC8A1,SLC8A3,SNAP25,STRN,STXBP1,SYT1,UTRN,PICALM,CASK,NRP1,SYNJ1,DLG5,HOMER1,GABBR2,SNCAIP,RIMS2,SNAP91,AKAP9,CPEB3,NLGN1,CLSTN1,SHANK2,RIMS1,ERC1,SYNE1,PSD3,ZDHHC17,FLRT3,LRRTM2,DNM3,NPTN,DISC1,PCDH17,ADAM22,CNTN5,ANKS1B,GOPC,LRRC7,ABHD17C,CPEB1,LRRTM4,CPEB4,NETO1,SYAP1,KCTD12,STXBP5,CNIH3,LRRTM3 |
| 4_Member | GO Cellular Components | GO:0098984 | neuron to neuron synapse | -17.98942501 | -15.410 | 83/350 | 102,375,659,664,775,793,1129,1499,1739,1740,1741,1759,2043,2045,2066,2332,2891,2895,2898,2900,2903,2911,2913,2915,3708,3756,4131,4133,4842,4897,5028,5062,5170,5332,5590,5594,5727,6009,6457,6711,6801,6857,7534,7804,8087,9101,9201,9231,9456,9465,9758,9829,9863,10142,10564,11069,22849,22871,22883,22941,23362,23373,26045,26052,26115,27020,27185,27445,53616,54477,55737,56899,57120,57534,57554,58489,64506,80315,81831,81832,140735,220988,347731 | ADAM10,ARF1,BMPR2,BNIP3,CACNA1C,CALB1,CHRM2,CTNNB1,DLG1,DLG2,DLG3,DNM1,EPHA4,EPHA7,ERBB4,FMR1,GRIA2,GRID2,GRIK2,GRIK4,GRIN2A,GRM1,GRM3,GRM5,ITPR1,KCNH1,MAP1B,MAP2,NOS1,NRCAM,P2RY1,PAK2,PDPK1,PLCB4,PRKCZ,MAPK1,PTCH1,RHEB,SH3GL3,SPTBN1,STRN,SYT1,YWHAZ,LRP8,FXR1,USP8,DCLK1,DLG5,HOMER1,AKAP7,FRMPD4,DNAJC6,MAGI2,AKAP9,ARFGEF2,RAPGEF4,CPEB3,NLGN1,CLSTN1,SHANK2,PSD3,CRTC1,LRRTM2,DNM3,TANC2,NPTN,DISC1,PCLO,ADAM22,PLEKHA5,VPS35,ANKS1B,GOPC,MIB1,LRRC7,ABHD17C,CPEB1,CPEB4,NETO2,NETO1,DYNLL2,HNRNPA3,LRRTM3 |
| 4_Member | GO Cellular Components | GO:0032279 | asymmetric synapse | -16.46594587 | -13.985 | 77/328 | 102,375,659,664,775,1129,1499,1739,1740,1741,1759,2043,2045,2066,2332,2891,2895,2898,2903,2911,2913,2915,3708,3756,4131,4133,4842,4897,5028,5062,5170,5332,5590,5594,5727,6009,6457,6711,6801,7804,8087,9101,9201,9231,9456,9758,9829,9863,10142,10564,22849,22871,22883,22941,23362,23373,26045,26052,26115,27020,27185,27445,53616,54477,55737,56899,57120,57534,57554,58489,64506,80315,81831,81832,140735,220988,347731 | ADAM10,ARF1,BMPR2,BNIP3,CACNA1C,CHRM2,CTNNB1,DLG1,DLG2,DLG3,DNM1,EPHA4,EPHA7,ERBB4,FMR1,GRIA2,GRID2,GRIK2,GRIN2A,GRM1,GRM3,GRM5,ITPR1,KCNH1,MAP1B,MAP2,NOS1,NRCAM,P2RY1,PAK2,PDPK1,PLCB4,PRKCZ,MAPK1,PTCH1,RHEB,SH3GL3,SPTBN1,STRN,LRP8,FXR1,USP8,DCLK1,DLG5,HOMER1,FRMPD4,DNAJC6,MAGI2,AKAP9,ARFGEF2,CPEB3,NLGN1,CLSTN1,SHANK2,PSD3,CRTC1,LRRTM2,DNM3,TANC2,NPTN,DISC1,PCLO,ADAM22,PLEKHA5,VPS35,ANKS1B,GOPC,MIB1,LRRC7,ABHD17C,CPEB1,CPEB4,NETO2,NETO1,DYNLL2,HNRNPA3,LRRTM3 |
| 4_Member | GO Cellular Components | GO:0014069 | postsynaptic density | -15.69708935 | -13.277 | 75/324 | 102,375,659,664,775,1499,1739,1740,1741,1759,2043,2045,2066,2332,2891,2895,2898,2903,2911,2913,2915,3708,3756,4131,4133,4842,4897,5028,5062,5170,5332,5590,5594,5727,6009,6457,6711,6801,7804,8087,9101,9201,9231,9456,9758,9829,9863,10142,22849,22871,22883,22941,23362,23373,26045,26052,26115,27020,27185,27445,53616,54477,55737,56899,57120,57534,57554,58489,64506,80315,81831,81832,140735,220988,347731 | ADAM10,ARF1,BMPR2,BNIP3,CACNA1C,CTNNB1,DLG1,DLG2,DLG3,DNM1,EPHA4,EPHA7,ERBB4,FMR1,GRIA2,GRID2,GRIK2,GRIN2A,GRM1,GRM3,GRM5,ITPR1,KCNH1,MAP1B,MAP2,NOS1,NRCAM,P2RY1,PAK2,PDPK1,PLCB4,PRKCZ,MAPK1,PTCH1,RHEB,SH3GL3,SPTBN1,STRN,LRP8,FXR1,USP8,DCLK1,DLG5,HOMER1,FRMPD4,DNAJC6,MAGI2,AKAP9,CPEB3,NLGN1,CLSTN1,SHANK2,PSD3,CRTC1,LRRTM2,DNM3,TANC2,NPTN,DISC1,PCLO,ADAM22,PLEKHA5,VPS35,ANKS1B,GOPC,MIB1,LRRC7,ABHD17C,CPEB1,CPEB4,NETO2,NETO1,DYNLL2,HNRNPA3,LRRTM3 |
| 4_Member | GO Cellular Components | GO:0045211 | postsynaptic membrane | -15.23902714 | -12.852 | 74/323 | 102,288,375,775,1007,1129,1272,1399,1499,1739,1740,1741,1759,2043,2045,2048,2066,2332,2554,2742,2743,2891,2895,2898,2900,2903,2911,2913,2915,3745,3746,3747,3756,4897,4916,5028,5728,6529,6538,6546,6547,6801,7402,8301,8829,9231,9456,9568,10142,22849,22871,22883,22941,23345,23362,23767,26045,26052,27020,27185,27253,53616,56899,57120,57554,58489,64506,80059,80315,81832,94056,115207,149111,347731 | ADAM10,ANK3,ARF1,CACNA1C,CDH9,CHRM2,CNTN1,CRKL,CTNNB1,DLG1,DLG2,DLG3,DNM1,EPHA4,EPHA7,EPHB2,ERBB4,FMR1,GABRA1,GLRA2,GLRB,GRIA2,GRID2,GRIK2,GRIK4,GRIN2A,GRM1,GRM3,GRM5,KCNB1,KCNC1,KCNC2,KCNH1,NRCAM,NTRK3,P2RY1,PTEN,SLC6A1,SLC6A11,SLC8A1,SLC8A3,STRN,UTRN,PICALM,NRP1,DLG5,HOMER1,GABBR2,AKAP9,CPEB3,NLGN1,CLSTN1,SHANK2,SYNE1,PSD3,FLRT3,LRRTM2,DNM3,NPTN,DISC1,PCDH17,ADAM22,ANKS1B,GOPC,LRRC7,ABHD17C,CPEB1,LRRTM4,CPEB4,NETO1,SYAP1,KCTD12,CNIH3,LRRTM3 |
| 4_Member | GO Cellular Components | GO:0099572 | postsynaptic specialization | -14.43350122 | -12.134 | 76/348 | 102,375,659,664,775,1499,1739,1740,1741,1759,2043,2045,2066,2332,2554,2891,2895,2898,2903,2911,2913,2915,3708,3756,4131,4133,4842,4897,5028,5062,5170,5332,5590,5594,5727,6009,6457,6711,6801,7804,8087,9101,9201,9231,9456,9758,9829,9863,10142,22849,22871,22883,22941,23362,23373,26045,26052,26115,27020,27185,27445,53616,54477,55737,56899,57120,57534,57554,58489,64506,80315,81831,81832,140735,220988,347731 | ADAM10,ARF1,BMPR2,BNIP3,CACNA1C,CTNNB1,DLG1,DLG2,DLG3,DNM1,EPHA4,EPHA7,ERBB4,FMR1,GABRA1,GRIA2,GRID2,GRIK2,GRIN2A,GRM1,GRM3,GRM5,ITPR1,KCNH1,MAP1B,MAP2,NOS1,NRCAM,P2RY1,PAK2,PDPK1,PLCB4,PRKCZ,MAPK1,PTCH1,RHEB,SH3GL3,SPTBN1,STRN,LRP8,FXR1,USP8,DCLK1,DLG5,HOMER1,FRMPD4,DNAJC6,MAGI2,AKAP9,CPEB3,NLGN1,CLSTN1,SHANK2,PSD3,CRTC1,LRRTM2,DNM3,TANC2,NPTN,DISC1,PCLO,ADAM22,PLEKHA5,VPS35,ANKS1B,GOPC,MIB1,LRRC7,ABHD17C,CPEB1,CPEB4,NETO2,NETO1,DYNLL2,HNRNPA3,LRRTM3 |
| 4_Member | GO Cellular Components | GO:0044297 | cell body | -14.17197626 | -11.897 | 104/564 | 59,102,657,659,774,775,793,998,1129,1741,1826,2043,2044,2045,2048,2182,2332,2781,2782,2823,2898,3267,3745,3746,3747,3756,3759,3781,3800,4040,4131,4133,4628,5028,5048,5066,5170,5563,5577,5590,5594,5792,5796,5868,6323,6416,6507,6547,6622,6780,6801,6860,7042,7150,7314,7430,7432,7804,7855,8087,8301,8829,8874,9312,9590,9627,9693,10142,10146,10159,10814,10846,10857,10891,11113,22853,22941,23001,23032,23162,23191,23373,23394,23774,27185,29966,54407,54464,55737,57154,57282,57565,57605,57626,58155,89848,91752,92737,94056,135138,140767,143187,146760,153090 | ACTA2,ADAM10,BMPR1A,BMPR2,CACNA1B,CACNA1C,CALB1,CDC42,CHRM2,DLG3,DSCAM,EPHA4,EPHA5,EPHA7,EPHB2,ACSL4,FMR1,GNAZ,GNB1,GPM6A,GRIK2,AGFG1,KCNB1,KCNC1,KCNC2,KCNH1,KCNJ2,KCNN2,KIF5C,LRP6,MAP1B,MAP2,MYH10,P2RY1,PAFAH1B1,PAM,PDPK1,PRKAA2,PRKAR2B,PRKCZ,MAPK1,PTPRF,PTPRK,RAB5A,SCN1A,MAP2K4,SLC1A3,SLC8A3,SNCA,STAU1,STRN,SYT4,TGFB2,TOP1,UBB,EZR,VIP,LRP8,FZD5,FXR1,PICALM,NRP1,ARHGEF7,KCNB2,AKAP12,SNCAIP,RAPGEF2,AKAP9,G3BP1,ATP6AP2,CPLX2,PDE10A,PGRMC1,PPARGC1A,CIT,LMTK2,SHANK2,WDFY3,USP33,MAPK8IP3,CYFIP1,CRTC1,ADNP,BRD1,DISC1,STRN3,SLC38A2,XRN1,VPS35,SMURF1,SLC4A10,KLHL14,PITPNM2,KLHL1,PTBP2,FCHSD1,ZNF804A,DNER,SYAP1,PACRG,NRSN1,VTI1A,RTN4RL1,DAB2IP |
| 4_Member | GO Cellular Components | GO:0043025 | neuronal cell body | -13.12239716 | -10.912 | 93/497 | 102,657,659,774,775,793,998,1129,1741,1826,2043,2044,2045,2048,2182,2332,2823,2898,3267,3745,3746,3747,3756,3759,3781,3800,4040,4131,4133,4628,5048,5066,5170,5563,5577,5590,5594,5792,5796,5868,6323,6416,6507,6547,6622,6780,6801,6860,7042,7150,7314,7432,7804,7855,8087,8301,8829,8874,9312,9590,9627,9693,10142,10146,10814,10846,10857,10891,11113,22853,22941,23001,23191,23373,23394,23774,29966,54407,54464,55737,57154,57282,57565,57626,58155,89848,91752,92737,94056,140767,143187,146760,153090 | ADAM10,BMPR1A,BMPR2,CACNA1B,CACNA1C,CALB1,CDC42,CHRM2,DLG3,DSCAM,EPHA4,EPHA5,EPHA7,EPHB2,ACSL4,FMR1,GPM6A,GRIK2,AGFG1,KCNB1,KCNC1,KCNC2,KCNH1,KCNJ2,KCNN2,KIF5C,LRP6,MAP1B,MAP2,MYH10,PAFAH1B1,PAM,PDPK1,PRKAA2,PRKAR2B,PRKCZ,MAPK1,PTPRF,PTPRK,RAB5A,SCN1A,MAP2K4,SLC1A3,SLC8A3,SNCA,STAU1,STRN,SYT4,TGFB2,TOP1,UBB,VIP,LRP8,FZD5,FXR1,PICALM,NRP1,ARHGEF7,KCNB2,AKAP12,SNCAIP,RAPGEF2,AKAP9,G3BP1,CPLX2,PDE10A,PGRMC1,PPARGC1A,CIT,LMTK2,SHANK2,WDFY3,CYFIP1,CRTC1,ADNP,BRD1,STRN3,SLC38A2,XRN1,VPS35,SMURF1,SLC4A10,KLHL14,KLHL1,PTBP2,FCHSD1,ZNF804A,DNER,SYAP1,NRSN1,VTI1A,RTN4RL1,DAB2IP |
| 4_Member | GO Cellular Components | GO:0044309 | neuron spine | -11.72243582 | -9.604 | 45/171 | 102,320,321,793,998,1759,2043,2332,2823,2891,2895,2903,2913,3688,3759,3781,4131,4628,4842,5530,5577,5728,5879,6546,6547,6717,6801,8087,8411,9101,9456,9758,10479,10564,22871,22883,22941,23191,23613,26052,55619,56899,58489,80315,91752 | ADAM10,APBA1,APBA2,CALB1,CDC42,DNM1,EPHA4,FMR1,GPM6A,GRIA2,GRID2,GRIN2A,GRM3,ITGB1,KCNJ2,KCNN2,MAP1B,MYH10,NOS1,PPP3CA,PRKAR2B,PTEN,RAC1,SLC8A1,SLC8A3,SRI,STRN,FXR1,EEA1,USP8,HOMER1,FRMPD4,SLC9A6,ARFGEF2,NLGN1,CLSTN1,SHANK2,CYFIP1,ZMYND8,DNM3,DOCK10,ANKS1B,ABHD17C,CPEB4,ZNF804A |
| 4_Member | GO Cellular Components | GO:0043197 | dendritic spine | -10.70857686 | -8.674 | 43/169 | 102,320,321,793,998,1759,2043,2332,2823,2891,2895,2903,2913,3688,3759,3781,4131,4628,4842,5530,5577,5728,5879,6546,6547,6717,6801,8087,9101,9456,9758,10564,22871,22883,22941,23191,23613,26052,55619,56899,58489,80315,91752 | ADAM10,APBA1,APBA2,CALB1,CDC42,DNM1,EPHA4,FMR1,GPM6A,GRIA2,GRID2,GRIN2A,GRM3,ITGB1,KCNJ2,KCNN2,MAP1B,MYH10,NOS1,PPP3CA,PRKAR2B,PTEN,RAC1,SLC8A1,SLC8A3,SRI,STRN,FXR1,USP8,HOMER1,FRMPD4,ARFGEF2,NLGN1,CLSTN1,SHANK2,CYFIP1,ZMYND8,DNM3,DOCK10,ANKS1B,ABHD17C,CPEB4,ZNF804A |
| 5_Summary | GO Cellular Components | GO:0030424 | axon | -27.67256561 | -24.401 | 138/607 | 60,115,288,323,627,667,793,1129,1176,1268,1385,1739,1740,1741,1759,1826,1837,2043,2044,2048,2258,2332,2571,2572,2617,2823,2898,2913,3064,3084,3745,3746,3747,3756,3786,3799,3800,4036,4131,4133,4628,4685,4781,4842,4897,4916,4919,5048,5101,5563,5590,5594,5599,5727,5796,5868,6091,6092,6323,6328,6331,6416,6506,6529,6571,6616,6622,6683,6711,6717,6812,6857,6860,7042,7402,7531,7804,7855,8087,8239,8411,8829,8867,8943,9371,9456,9532,10371,10479,10814,11127,22848,22853,22941,23001,23011,23022,23046,23064,23077,23162,23191,23394,23405,23767,24141,26052,26053,26230,27020,27185,27252,51062,53616,54407,55207,55219,55638,55714,57154,57282,57453,57554,57580,57616,58155,63915,80315,81846,84665,91752,94056,130074,136319,140767,152330,153090,253559,143187 | ACTB,ADCY9,ANK3,APBB2,BDNF,DST,CALB1,CHRM2,AP3S1,CNR1,CREB1,DLG1,DLG2,DLG3,DNM1,DSCAM,DTNA,EPHA4,EPHA5,EPHB2,FGF13,FMR1,GAD1,GAD2,GARS1,GPM6A,GRIK2,GRM3,HTT,NRG1,KCNB1,KCNC1,KCNC2,KCNH1,KCNQ3,KIF5B,KIF5C,LRP2,MAP1B,MAP2,MYH10,NCAM2,NFIB,NOS1,NRCAM,NTRK3,ROR1,PAFAH1B1,PCDH9,PRKAA2,PRKCZ,MAPK1,MAPK8,PTCH1,PTPRK,RAB5A,ROBO1,ROBO2,SCN1A,SCN3A,SCN5A,MAP2K4,SLC1A2,SLC6A1,SLC18A2,SNAP25,SNCA,SPAST,SPTBN1,SRI,STXBP1,SYT1,SYT4,TGFB2,UTRN,YWHAE,LRP8,FZD5,FXR1,USP9X,EEA1,NRP1,SYNJ1,AP3D1,KIF3B,HOMER1,BAG2,SEMA3A,SLC9A6,CPLX2,KIF3A,AAK1,LMTK2,SHANK2,WDFY3,RAB21,PALLD,KIF21B,SETX,MYCBP2,MAPK8IP3,CYFIP1,ADNP,DICER1,FLRT3,LAMP5,DNM3,AUTS2,TIAM2,NPTN,DISC1,KLHL20,ATL1,ADAM22,SLC38A2,ARL8B,MACO1,SYBU,TENM3,SMURF1,SLC4A10,DSCAML1,LRRC7,PREX1,TSHZ3,PTBP2,BLOC1S5,CPEB4,SBF2,MYPN,ZNF804A,SYAP1,FAM168B,MTPN,NRSN1,CNTN4,DAB2IP,CADM2,VTI1A |
| 5_Member | GO Cellular Components | GO:0030424 | axon | -27.67256561 | -24.401 | 138/607 | 60,115,288,323,627,667,793,1129,1176,1268,1385,1739,1740,1741,1759,1826,1837,2043,2044,2048,2258,2332,2571,2572,2617,2823,2898,2913,3064,3084,3745,3746,3747,3756,3786,3799,3800,4036,4131,4133,4628,4685,4781,4842,4897,4916,4919,5048,5101,5563,5590,5594,5599,5727,5796,5868,6091,6092,6323,6328,6331,6416,6506,6529,6571,6616,6622,6683,6711,6717,6812,6857,6860,7042,7402,7531,7804,7855,8087,8239,8411,8829,8867,8943,9371,9456,9532,10371,10479,10814,11127,22848,22853,22941,23001,23011,23022,23046,23064,23077,23162,23191,23394,23405,23767,24141,26052,26053,26230,27020,27185,27252,51062,53616,54407,55207,55219,55638,55714,57154,57282,57453,57554,57580,57616,58155,63915,80315,81846,84665,91752,94056,130074,136319,140767,152330,153090,253559 | ACTB,ADCY9,ANK3,APBB2,BDNF,DST,CALB1,CHRM2,AP3S1,CNR1,CREB1,DLG1,DLG2,DLG3,DNM1,DSCAM,DTNA,EPHA4,EPHA5,EPHB2,FGF13,FMR1,GAD1,GAD2,GARS1,GPM6A,GRIK2,GRM3,HTT,NRG1,KCNB1,KCNC1,KCNC2,KCNH1,KCNQ3,KIF5B,KIF5C,LRP2,MAP1B,MAP2,MYH10,NCAM2,NFIB,NOS1,NRCAM,NTRK3,ROR1,PAFAH1B1,PCDH9,PRKAA2,PRKCZ,MAPK1,MAPK8,PTCH1,PTPRK,RAB5A,ROBO1,ROBO2,SCN1A,SCN3A,SCN5A,MAP2K4,SLC1A2,SLC6A1,SLC18A2,SNAP25,SNCA,SPAST,SPTBN1,SRI,STXBP1,SYT1,SYT4,TGFB2,UTRN,YWHAE,LRP8,FZD5,FXR1,USP9X,EEA1,NRP1,SYNJ1,AP3D1,KIF3B,HOMER1,BAG2,SEMA3A,SLC9A6,CPLX2,KIF3A,AAK1,LMTK2,SHANK2,WDFY3,RAB21,PALLD,KIF21B,SETX,MYCBP2,MAPK8IP3,CYFIP1,ADNP,DICER1,FLRT3,LAMP5,DNM3,AUTS2,TIAM2,NPTN,DISC1,KLHL20,ATL1,ADAM22,SLC38A2,ARL8B,MACO1,SYBU,TENM3,SMURF1,SLC4A10,DSCAML1,LRRC7,PREX1,TSHZ3,PTBP2,BLOC1S5,CPEB4,SBF2,MYPN,ZNF804A,SYAP1,FAM168B,MTPN,NRSN1,CNTN4,DAB2IP,CADM2 |
| 5_Member | GO Cellular Components | GO:0033267 | axon part | -15.06827673 | -12.700 | 82/382 | 60,288,323,667,793,1129,1176,1268,1739,1740,1741,1759,1826,2043,2258,2332,2571,2823,2898,3746,3747,3786,3799,3800,4131,4133,4628,4842,4897,4919,5048,5101,5590,5727,5868,6092,6323,6506,6571,6616,6622,6683,6711,6717,7402,7531,8239,8411,8829,8867,8943,9371,10479,10814,11127,22848,22853,22941,23011,23022,23046,23064,23162,23191,23405,23767,24141,26053,26230,27185,55207,55638,57282,57554,57580,57616,58155,63915,80315,91752,94056,140767 | ACTB,ANK3,APBB2,DST,CALB1,CHRM2,AP3S1,CNR1,DLG1,DLG2,DLG3,DNM1,DSCAM,EPHA4,FGF13,FMR1,GAD1,GPM6A,GRIK2,KCNC1,KCNC2,KCNQ3,KIF5B,KIF5C,MAP1B,MAP2,MYH10,NOS1,NRCAM,ROR1,PAFAH1B1,PCDH9,PRKCZ,PTCH1,RAB5A,ROBO2,SCN1A,SLC1A2,SLC18A2,SNAP25,SNCA,SPAST,SPTBN1,SRI,UTRN,YWHAE,USP9X,EEA1,NRP1,SYNJ1,AP3D1,KIF3B,SLC9A6,CPLX2,KIF3A,AAK1,LMTK2,SHANK2,RAB21,PALLD,KIF21B,SETX,MAPK8IP3,CYFIP1,DICER1,FLRT3,LAMP5,AUTS2,TIAM2,DISC1,ARL8B,SYBU,SLC4A10,LRRC7,PREX1,TSHZ3,PTBP2,BLOC1S5,CPEB4,ZNF804A,SYAP1,NRSN1 |
| 5_Member | GO Cellular Components | GO:0150034 | distal axon | -10.83147845 | -8.780 | 60/285 | 60,323,793,1129,1268,1741,1826,2043,2258,2332,2571,2823,2898,3746,3747,3799,3800,4131,4133,4628,4842,4919,5048,5101,5727,5868,6571,6616,6622,6717,7402,7531,8239,8829,8867,8943,10479,10814,22848,22853,22941,23022,23046,23064,23162,23191,23405,23767,24141,26053,26230,27185,57282,57580,57616,58155,80315,91752,94056,140767 | ACTB,APBB2,CALB1,CHRM2,CNR1,DLG3,DSCAM,EPHA4,FGF13,FMR1,GAD1,GPM6A,GRIK2,KCNC1,KCNC2,KIF5B,KIF5C,MAP1B,MAP2,MYH10,NOS1,ROR1,PAFAH1B1,PCDH9,PTCH1,RAB5A,SLC18A2,SNAP25,SNCA,SRI,UTRN,YWHAE,USP9X,NRP1,SYNJ1,AP3D1,SLC9A6,CPLX2,AAK1,LMTK2,SHANK2,PALLD,KIF21B,SETX,MAPK8IP3,CYFIP1,DICER1,FLRT3,LAMP5,AUTS2,TIAM2,DISC1,SLC4A10,PREX1,TSHZ3,PTBP2,CPEB4,ZNF804A,SYAP1,NRSN1 |
| 5_Member | GO Cellular Components | GO:0030426 | growth cone | -9.950701931 | -7.992 | 42/171 | 323,1268,1741,1826,2043,2258,2332,2823,3799,3800,4131,4133,4628,5048,5101,5727,6616,6622,7402,7531,8239,8829,22853,22941,23022,23046,23064,23162,23191,23405,23767,24141,26053,26230,27185,57580,57616,58155,80315,91752,94056,140767 | APBB2,CNR1,DLG3,DSCAM,EPHA4,FGF13,FMR1,GPM6A,KIF5B,KIF5C,MAP1B,MAP2,MYH10,PAFAH1B1,PCDH9,PTCH1,SNAP25,SNCA,UTRN,YWHAE,USP9X,NRP1,LMTK2,SHANK2,PALLD,KIF21B,SETX,MAPK8IP3,CYFIP1,DICER1,FLRT3,LAMP5,AUTS2,TIAM2,DISC1,PREX1,TSHZ3,PTBP2,CPEB4,ZNF804A,SYAP1,NRSN1 |
| 5_Member | GO Cellular Components | GO:0030427 | site of polarized growth | -9.530246172 | -7.601 | 42/176 | 323,1268,1741,1826,2043,2258,2332,2823,3799,3800,4131,4133,4628,5048,5101,5727,6616,6622,7402,7531,8239,8829,22853,22941,23022,23046,23064,23162,23191,23405,23767,24141,26053,26230,27185,57580,57616,58155,80315,91752,94056,140767 | APBB2,CNR1,DLG3,DSCAM,EPHA4,FGF13,FMR1,GPM6A,KIF5B,KIF5C,MAP1B,MAP2,MYH10,PAFAH1B1,PCDH9,PTCH1,SNAP25,SNCA,UTRN,YWHAE,USP9X,NRP1,LMTK2,SHANK2,PALLD,KIF21B,SETX,MAPK8IP3,CYFIP1,DICER1,FLRT3,LAMP5,AUTS2,TIAM2,DISC1,PREX1,TSHZ3,PTBP2,CPEB4,ZNF804A,SYAP1,NRSN1 |
| 5_Member | GO Cellular Components | GO:0044306 | neuron projection terminus | -4.64107101 | -3.221 | 27/138 | 60,793,1129,2043,2332,2571,2898,3746,3747,4842,4919,5868,6571,6622,6717,6857,6860,8867,8943,10479,10814,22848,23191,23767,55219,57282,143187 | ACTB,CALB1,CHRM2,EPHA4,FMR1,GAD1,GRIK2,KCNC1,KCNC2,NOS1,ROR1,RAB5A,SLC18A2,SNCA,SRI,SYT1,SYT4,SYNJ1,AP3D1,SLC9A6,CPLX2,AAK1,CYFIP1,FLRT3,MACO1,SLC4A10,VTI1A |
| 5_Member | GO Cellular Components | GO:0043679 | axon terminus | -3.963519483 | -2.645 | 23/119 | 60,793,1129,2043,2332,2571,2898,3746,3747,4842,4919,5868,6571,6622,6717,8867,8943,10479,10814,22848,23191,23767,57282 | ACTB,CALB1,CHRM2,EPHA4,FMR1,GAD1,GRIK2,KCNC1,KCNC2,NOS1,ROR1,RAB5A,SLC18A2,SNCA,SRI,SYNJ1,AP3D1,SLC9A6,CPLX2,AAK1,CYFIP1,FLRT3,SLC4A10 |
| 6_Summary | GO Biological Processes | GO:0045664 | regulation of neuron differentiation | -23.1919862 | -20.097 | 136/656 | 27,102,375,596,604,627,650,659,1002,1123,1268,1272,1399,1656,1808,1826,1977,2033,2042,2043,2045,2048,2049,2138,2258,2332,2624,2736,2737,2803,2895,3309,3475,3655,4076,4131,4133,4139,4208,4211,4760,4897,4916,5048,5080,5361,5376,5530,5587,5597,5629,5728,5756,5764,5789,5792,5793,5911,5915,5978,6091,6092,6093,6383,6457,6616,6857,6860,6929,6935,7068,7225,7337,7804,8724,8829,9043,9231,9314,9315,9373,9448,9693,9699,9706,9723,9839,9863,10371,10501,10507,10512,10716,22849,22871,22941,22999,23011,23064,23077,23189,23191,23258,23327,23373,23394,23531,23613,26039,26052,27020,27185,28514,29116,55704,55714,55754,56920,57154,57520,57534,57554,57580,57688,60436,64919,65009,80031,84894,85458,91752,114788,146760,152330,153090,221935,324,998,1540,2150,2241,2823,3064,3815,5580,5868,5879,5898,7046,7419,7430,7813,8558,8874,8976,9456,9474,9712,10178,10787,11138,23603,26053,54874,55296,55737,57489,64786,89795,202018,51762,133,320,321,367,546,657,780,1027,1028,1293,1303,1385,1499,1906,2066,2252,2255,2261,2263,2296,3021,3202,3239,3516,3688,3720,4040,4322,4534,5573,5590,5594,5727,6299,6416,6506,6781,7042,7048,7403,7414,7458,7490,7528,8239,8324,8455,8470,8531,8614,9201,9318,9355,9472,10252,10479,10959,11221,23168,23414,23767,25937,26524,51804,55636,55777,55841,57178,57282,57599,64856,79718,84159,84189,85407,114789,136319,157506,285590,4036,5270,5468,6009,7058,7162,9921,22883,23013,23060,23376,23405,26045,55179,57633,84631,158038,347731,2244,2266,5991,8600,10221,64855,390,1739,2768,4627,4628,5028,5921,7750,9948,10395,10672,10979,23370,55023,55435,55785,56990,57381,58480,118987,152503,192670,221061,254065,323,694,734,817,1457,1654,1982,2487,3624,4487,5062,5515,6446,6595,8725,9211,9306,9655,10276,10477,23678,29967,51542,54617,55031,55602,79577,80314,83737,84289,84678,116224,131566,361,1182,1185,6558,9821,10000 | ABL2,ADAM10,ARF1,BCL2,BCL6,BDNF,BMP2,BMPR2,CDH4,CHN1,CNR1,CNTN1,CRKL,DDX6,DPYSL2,DSCAM,EIF4E,EP300,EPHA3,EPHA4,EPHA7,EPHB2,EPHB3,EYA1,FGF13,FMR1,GATA2,GLI2,GLI3,GOLGA4,GRID2,HSPA5,IFRD1,ITGA6,CAPRIN1,MAP1B,MAP2,MARK1,MEF2C,MEIS1,NEUROD1,NRCAM,NTRK3,PAFAH1B1,PAX6,PLXNA1,PMP22,PPP3CA,PRKD1,MAPK6,PROX1,PTEN,TWF1,PTN,PTPRD,PTPRF,PTPRG,RAP2A,RARB,REST,ROBO1,ROBO2,ROCK1,SDC2,SH3GL3,SNAP25,SYT1,SYT4,TCF3,ZEB1,THRB,TRPC6,UBE3A,LRP8,SNX3,NRP1,SPAG9,DLG5,KLF4,NREP,PLAA,MAP4K4,RAPGEF2,RIMS2,ULK2,SEMA3E,ZEB2,MAGI2,SEMA3A,SEMA6B,SEMA4D,SEMA3C,TBR1,CPEB3,NLGN1,SHANK2,RIMS1,RAB21,SETX,MYCBP2,KANK1,CYFIP1,DENND5A,NEDD4L,CRTC1,ADNP,MMD,ZMYND8,SS18L1,DNM3,NPTN,DISC1,DLL1,MYLIP,CCDC88A,TENM3,TMEM30A,SEMA3G,SMURF1,HECW2,MIB1,LRRC7,PREX1,ZSWIM6,TGIF2,BCL11B,NDRG4,SEMA6D,LINGO1,DIXDC1,ZNF804A,CSMD3,RTN4RL1,CNTN4,DAB2IP,SDK1,APC,CDC42,CYLD,F2RL1,FER,GPM6A,HTT,KIT,PRKCD,RAB5A,RAC1,RALA,TGFBR1,VDAC3,EZR,EVI5,CDK10,ARHGEF7,WASL,HOMER1,ATG5,USP6NL,TENM1,NCKAP1,TBC1D8,CORO1C,AUTS2,FNBP1L,TBC1D19,VPS35,ODF2L,TBC1D15,NAV3,TAPT1,RAB8B,ADM,APBA1,APBA2,AR,ATRX,BMPR1A,DDR1,CDKN1B,CDKN1C,COL6A3,COL12A1,CREB1,CTNNB1,EDN1,ERBB4,FGF7,FGF10,FGFR3,FGFR2,FOXC1,H3-3B,HOXA5,HOXD13,RBPJ,ITGB1,JARID2,LRP6,MMP13,MTM1,PRKAR1A,PRKCZ,MAPK1,PTCH1,SALL1,MAP2K4,SLC1A2,STC1,TGFB2,TGFBR2,KDM6A,VCL,EIF4H,WT1,YY1,USP9X,FZD7,ATRN,SORBS2,YBX3,STC2,DCLK1,COPS2,LHX2,AKAP6,SPRY1,SLC9A6,TMED2,DUSP10,RTF1,ZFPM2,FLRT3,WWTR1,LATS2,SIX4,CHD7,MBD5,WWC3,ZMIZ1,SLC4A10,WDR48,VWA1,TBL1XR1,ARID5B,SLITRK6,NKD1,SLC25A25,MTPN,RDH10,SH3PXD2B,LRP2,SERPINE2,PPARG,RHEB,THBS2,TPBG,RNF10,CLSTN1,SPEN,ZNF609,UFL1,DICER1,LRRTM2,FAIM,LRRN1,SLITRK2,LINGO2,LRRTM3,FGB,FGG,RFX3,TNFSF11,TRIB1,NIBAN2,RND3,DLG1,GNA12,MYH9,MYH10,P2RY1,RASA1,ZMYM2,WDR1,DLC1,GNA13,FERMT2,ARHGEF18,PHIP,AP1AR,FGD6,CDC42SE2,RHOJ,RHOU,PDZD8,SH3D19,AGO4,FAM171A1,BRWD3,APBB2,BTG1,OSGIN2,CAMK2D,CSNK2A1,DDX3X,EIF4G2,FRZB,INHBA,MSX1,PAK2,PPP2CA,SGK1,SMARCA2,URI1,LGI1,SOCS6,SOCS5,NET1,UBE2E3,SGK3,LRP12,VPS54,INO80,USP47,CDKN2AIP,CDC73,EPC1,ITCH,ING5,KDM2B,FAM122A,DCBLD2,AQP4,CLCN3,CLCN6,SLC12A2,RB1CC1,AKT3 |
| 6_Member | GO Biological Processes | GO:0045664 | regulation of neuron differentiation | -23.1919862 | -20.097 | 136/656 | 27,102,375,596,604,627,650,659,1002,1123,1268,1272,1399,1656,1808,1826,1977,2033,2042,2043,2045,2048,2049,2138,2258,2332,2624,2736,2737,2803,2895,3309,3475,3655,4076,4131,4133,4139,4208,4211,4760,4897,4916,5048,5080,5361,5376,5530,5587,5597,5629,5728,5756,5764,5789,5792,5793,5911,5915,5978,6091,6092,6093,6383,6457,6616,6857,6860,6929,6935,7068,7225,7337,7804,8724,8829,9043,9231,9314,9315,9373,9448,9693,9699,9706,9723,9839,9863,10371,10501,10507,10512,10716,22849,22871,22941,22999,23011,23064,23077,23189,23191,23258,23327,23373,23394,23531,23613,26039,26052,27020,27185,28514,29116,55704,55714,55754,56920,57154,57520,57534,57554,57580,57688,60436,64919,65009,80031,84894,85458,91752,114788,146760,152330,153090,221935 | ABL2,ADAM10,ARF1,BCL2,BCL6,BDNF,BMP2,BMPR2,CDH4,CHN1,CNR1,CNTN1,CRKL,DDX6,DPYSL2,DSCAM,EIF4E,EP300,EPHA3,EPHA4,EPHA7,EPHB2,EPHB3,EYA1,FGF13,FMR1,GATA2,GLI2,GLI3,GOLGA4,GRID2,HSPA5,IFRD1,ITGA6,CAPRIN1,MAP1B,MAP2,MARK1,MEF2C,MEIS1,NEUROD1,NRCAM,NTRK3,PAFAH1B1,PAX6,PLXNA1,PMP22,PPP3CA,PRKD1,MAPK6,PROX1,PTEN,TWF1,PTN,PTPRD,PTPRF,PTPRG,RAP2A,RARB,REST,ROBO1,ROBO2,ROCK1,SDC2,SH3GL3,SNAP25,SYT1,SYT4,TCF3,ZEB1,THRB,TRPC6,UBE3A,LRP8,SNX3,NRP1,SPAG9,DLG5,KLF4,NREP,PLAA,MAP4K4,RAPGEF2,RIMS2,ULK2,SEMA3E,ZEB2,MAGI2,SEMA3A,SEMA6B,SEMA4D,SEMA3C,TBR1,CPEB3,NLGN1,SHANK2,RIMS1,RAB21,SETX,MYCBP2,KANK1,CYFIP1,DENND5A,NEDD4L,CRTC1,ADNP,MMD,ZMYND8,SS18L1,DNM3,NPTN,DISC1,DLL1,MYLIP,CCDC88A,TENM3,TMEM30A,SEMA3G,SMURF1,HECW2,MIB1,LRRC7,PREX1,ZSWIM6,TGIF2,BCL11B,NDRG4,SEMA6D,LINGO1,DIXDC1,ZNF804A,CSMD3,RTN4RL1,CNTN4,DAB2IP,SDK1 |
| 6_Member | GO Biological Processes | GO:0120035 | regulation of plasma membrane bounded cell projection organization | -22.89872842 | -19.849 | 141/698 | 27,102,324,375,627,659,998,1002,1123,1268,1272,1399,1540,1808,1826,2033,2042,2043,2045,2048,2049,2150,2241,2258,2332,2803,2823,2895,3064,3309,3475,3655,3815,4076,4131,4133,4139,4208,4897,4916,5048,5361,5376,5530,5580,5587,5597,5728,5756,5764,5789,5792,5793,5868,5879,5898,5911,6091,6092,6383,6616,6857,6860,7046,7225,7337,7419,7430,7804,7813,8558,8724,8829,8874,8976,9231,9314,9373,9448,9456,9474,9693,9699,9706,9712,9723,9839,9863,10178,10371,10501,10507,10512,10716,10787,11138,22849,22871,22941,22999,23011,23064,23077,23189,23191,23258,23327,23373,23394,23603,23613,26039,26052,26053,27020,27185,29116,54874,55296,55704,55714,55737,55754,56920,57154,57489,57520,57554,57580,57688,64786,65009,80031,84894,89795,91752,114788,146760,153090,202018,221935 | ABL2,ADAM10,APC,ARF1,BDNF,BMPR2,CDC42,CDH4,CHN1,CNR1,CNTN1,CRKL,CYLD,DPYSL2,DSCAM,EP300,EPHA3,EPHA4,EPHA7,EPHB2,EPHB3,F2RL1,FER,FGF13,FMR1,GOLGA4,GPM6A,GRID2,HTT,HSPA5,IFRD1,ITGA6,KIT,CAPRIN1,MAP1B,MAP2,MARK1,MEF2C,NRCAM,NTRK3,PAFAH1B1,PLXNA1,PMP22,PPP3CA,PRKCD,PRKD1,MAPK6,PTEN,TWF1,PTN,PTPRD,PTPRF,PTPRG,RAB5A,RAC1,RALA,RAP2A,ROBO1,ROBO2,SDC2,SNAP25,SYT1,SYT4,TGFBR1,TRPC6,UBE3A,VDAC3,EZR,LRP8,EVI5,CDK10,SNX3,NRP1,ARHGEF7,WASL,DLG5,KLF4,PLAA,MAP4K4,HOMER1,ATG5,RAPGEF2,RIMS2,ULK2,USP6NL,SEMA3E,ZEB2,MAGI2,TENM1,SEMA3A,SEMA6B,SEMA4D,SEMA3C,TBR1,NCKAP1,TBC1D8,CPEB3,NLGN1,SHANK2,RIMS1,RAB21,SETX,MYCBP2,KANK1,CYFIP1,DENND5A,NEDD4L,CRTC1,ADNP,CORO1C,ZMYND8,SS18L1,DNM3,AUTS2,NPTN,DISC1,MYLIP,FNBP1L,TBC1D19,CCDC88A,TENM3,VPS35,TMEM30A,SEMA3G,SMURF1,ODF2L,HECW2,LRRC7,PREX1,ZSWIM6,TBC1D15,NDRG4,SEMA6D,LINGO1,NAV3,ZNF804A,CSMD3,RTN4RL1,DAB2IP,TAPT1,SDK1 |
| 6_Member | GO Biological Processes | GO:0031344 | regulation of cell projection organization | -22.74732594 | -19.739 | 142/708 | 27,102,324,375,627,659,998,1002,1123,1268,1272,1399,1540,1808,1826,2033,2042,2043,2045,2048,2049,2150,2241,2258,2332,2803,2823,2895,3064,3309,3475,3655,3815,4076,4131,4133,4139,4208,4897,4916,5048,5361,5376,5530,5580,5587,5597,5728,5756,5764,5789,5792,5793,5868,5879,5898,5911,6091,6092,6383,6616,6857,6860,7046,7225,7337,7419,7430,7804,7813,8558,8724,8829,8874,8976,9231,9314,9373,9448,9456,9474,9693,9699,9706,9712,9723,9839,9863,10178,10371,10501,10507,10512,10716,10787,11138,22849,22871,22941,22999,23011,23064,23077,23189,23191,23258,23327,23373,23394,23603,23613,26039,26052,26053,27020,27185,29116,51762,54874,55296,55704,55714,55737,55754,56920,57154,57489,57520,57554,57580,57688,64786,65009,80031,84894,89795,91752,114788,146760,153090,202018,221935 | ABL2,ADAM10,APC,ARF1,BDNF,BMPR2,CDC42,CDH4,CHN1,CNR1,CNTN1,CRKL,CYLD,DPYSL2,DSCAM,EP300,EPHA3,EPHA4,EPHA7,EPHB2,EPHB3,F2RL1,FER,FGF13,FMR1,GOLGA4,GPM6A,GRID2,HTT,HSPA5,IFRD1,ITGA6,KIT,CAPRIN1,MAP1B,MAP2,MARK1,MEF2C,NRCAM,NTRK3,PAFAH1B1,PLXNA1,PMP22,PPP3CA,PRKCD,PRKD1,MAPK6,PTEN,TWF1,PTN,PTPRD,PTPRF,PTPRG,RAB5A,RAC1,RALA,RAP2A,ROBO1,ROBO2,SDC2,SNAP25,SYT1,SYT4,TGFBR1,TRPC6,UBE3A,VDAC3,EZR,LRP8,EVI5,CDK10,SNX3,NRP1,ARHGEF7,WASL,DLG5,KLF4,PLAA,MAP4K4,HOMER1,ATG5,RAPGEF2,RIMS2,ULK2,USP6NL,SEMA3E,ZEB2,MAGI2,TENM1,SEMA3A,SEMA6B,SEMA4D,SEMA3C,TBR1,NCKAP1,TBC1D8,CPEB3,NLGN1,SHANK2,RIMS1,RAB21,SETX,MYCBP2,KANK1,CYFIP1,DENND5A,NEDD4L,CRTC1,ADNP,CORO1C,ZMYND8,SS18L1,DNM3,AUTS2,NPTN,DISC1,MYLIP,RAB8B,FNBP1L,TBC1D19,CCDC88A,TENM3,VPS35,TMEM30A,SEMA3G,SMURF1,ODF2L,HECW2,LRRC7,PREX1,ZSWIM6,TBC1D15,NDRG4,SEMA6D,LINGO1,NAV3,ZNF804A,CSMD3,RTN4RL1,DAB2IP,TAPT1,SDK1 |
| 6_Member | GO Biological Processes | GO:0048589 | developmental growth | -21.11655251 | -18.297 | 134/674 | 133,320,321,367,546,596,627,657,659,780,1002,1027,1028,1293,1303,1385,1499,1808,1826,1906,2033,2045,2066,2252,2255,2258,2261,2263,2296,2736,2737,2803,3021,3202,3239,3475,3516,3688,3720,4040,4131,4133,4208,4211,4322,4534,4897,4916,5048,5361,5530,5573,5590,5594,5629,5727,5728,5764,5915,6299,6416,6506,6781,6857,6860,7042,7046,7048,7337,7403,7414,7430,7458,7490,7528,8239,8324,8455,8470,8531,8614,8829,9201,9318,9355,9373,9472,9699,9706,9723,9839,9863,10252,10371,10479,10501,10507,10512,10959,11221,22941,22999,23011,23168,23191,23327,23394,23414,23767,25937,26053,26524,27185,28514,51804,55636,55777,55841,56920,57154,57178,57282,57599,64856,65009,79718,80031,84159,84189,85407,114789,136319,157506,285590 | ADM,APBA1,APBA2,AR,ATRX,BCL2,BDNF,BMPR1A,BMPR2,DDR1,CDH4,CDKN1B,CDKN1C,COL6A3,COL12A1,CREB1,CTNNB1,DPYSL2,DSCAM,EDN1,EP300,EPHA7,ERBB4,FGF7,FGF10,FGF13,FGFR3,FGFR2,FOXC1,GLI2,GLI3,GOLGA4,H3-3B,HOXA5,HOXD13,IFRD1,RBPJ,ITGB1,JARID2,LRP6,MAP1B,MAP2,MEF2C,MEIS1,MMP13,MTM1,NRCAM,NTRK3,PAFAH1B1,PLXNA1,PPP3CA,PRKAR1A,PRKCZ,MAPK1,PROX1,PTCH1,PTEN,PTN,RARB,SALL1,MAP2K4,SLC1A2,STC1,SYT1,SYT4,TGFB2,TGFBR1,TGFBR2,UBE3A,KDM6A,VCL,EZR,EIF4H,WT1,YY1,USP9X,FZD7,ATRN,SORBS2,YBX3,STC2,NRP1,DCLK1,COPS2,LHX2,PLAA,AKAP6,RIMS2,ULK2,SEMA3E,ZEB2,MAGI2,SPRY1,SEMA3A,SLC9A6,SEMA6B,SEMA4D,SEMA3C,TMED2,DUSP10,SHANK2,RIMS1,RAB21,RTF1,CYFIP1,NEDD4L,ADNP,ZFPM2,FLRT3,WWTR1,AUTS2,LATS2,DISC1,DLL1,SIX4,CHD7,MBD5,WWC3,SEMA3G,SMURF1,ZMIZ1,SLC4A10,WDR48,VWA1,NDRG4,TBL1XR1,SEMA6D,ARID5B,SLITRK6,NKD1,SLC25A25,MTPN,RDH10,SH3PXD2B |
| 6_Member | GO Biological Processes | GO:0010975 | regulation of neuron projection development | -19.46190843 | -16.810 | 107/499 | 27,102,375,627,659,1002,1123,1268,1272,1399,1808,1826,2033,2042,2043,2045,2048,2049,2258,2332,2803,2895,3309,3475,3655,4076,4131,4133,4139,4208,4897,4916,5048,5361,5376,5530,5587,5597,5728,5756,5764,5789,5792,5793,5911,6091,6092,6383,6616,6857,6860,7225,7337,7804,8724,8829,9231,9314,9373,9448,9693,9699,9706,9723,9839,9863,10371,10501,10507,10512,10716,22849,22871,22941,22999,23011,23064,23077,23189,23191,23258,23327,23373,23394,23613,26039,26052,27020,27185,29116,55704,55714,55754,56920,57154,57520,57554,57580,57688,65009,80031,84894,91752,114788,146760,153090,221935 | ABL2,ADAM10,ARF1,BDNF,BMPR2,CDH4,CHN1,CNR1,CNTN1,CRKL,DPYSL2,DSCAM,EP300,EPHA3,EPHA4,EPHA7,EPHB2,EPHB3,FGF13,FMR1,GOLGA4,GRID2,HSPA5,IFRD1,ITGA6,CAPRIN1,MAP1B,MAP2,MARK1,MEF2C,NRCAM,NTRK3,PAFAH1B1,PLXNA1,PMP22,PPP3CA,PRKD1,MAPK6,PTEN,TWF1,PTN,PTPRD,PTPRF,PTPRG,RAP2A,ROBO1,ROBO2,SDC2,SNAP25,SYT1,SYT4,TRPC6,UBE3A,LRP8,SNX3,NRP1,DLG5,KLF4,PLAA,MAP4K4,RAPGEF2,RIMS2,ULK2,SEMA3E,ZEB2,MAGI2,SEMA3A,SEMA6B,SEMA4D,SEMA3C,TBR1,CPEB3,NLGN1,SHANK2,RIMS1,RAB21,SETX,MYCBP2,KANK1,CYFIP1,DENND5A,NEDD4L,CRTC1,ADNP,ZMYND8,SS18L1,DNM3,NPTN,DISC1,MYLIP,CCDC88A,TENM3,TMEM30A,SEMA3G,SMURF1,HECW2,LRRC7,PREX1,ZSWIM6,NDRG4,SEMA6D,LINGO1,ZNF804A,CSMD3,RTN4RL1,DAB2IP,SDK1 |
| 6_Member | GO Biological Processes | GO:0051962 | positive regulation of nervous system development | -17.22380576 | -14.686 | 108/541 | 27,375,596,604,627,650,659,1002,1268,1272,1399,1499,1826,2033,2042,2043,2048,2049,2332,2624,2736,2737,2803,2895,3309,3655,3815,4036,4076,4131,4208,4760,4897,4916,5048,5080,5270,5361,5468,5587,5597,5629,5728,5756,5764,5789,5915,5978,6009,6091,6092,6457,6857,6860,6929,6935,7058,7162,7225,7804,8724,8829,9043,9231,9373,9693,9699,9839,9863,9921,10371,10507,22849,22871,22883,22941,22999,23011,23013,23060,23064,23191,23327,23373,23376,23394,23405,23531,23613,23767,26039,26045,27020,27185,55179,55714,55754,57154,57554,57633,60436,65009,84189,84631,91752,153090,158038,347731 | ABL2,ARF1,BCL2,BCL6,BDNF,BMP2,BMPR2,CDH4,CNR1,CNTN1,CRKL,CTNNB1,DSCAM,EP300,EPHA3,EPHA4,EPHB2,EPHB3,FMR1,GATA2,GLI2,GLI3,GOLGA4,GRID2,HSPA5,ITGA6,KIT,LRP2,CAPRIN1,MAP1B,MEF2C,NEUROD1,NRCAM,NTRK3,PAFAH1B1,PAX6,SERPINE2,PLXNA1,PPARG,PRKD1,MAPK6,PROX1,PTEN,TWF1,PTN,PTPRD,RARB,REST,RHEB,ROBO1,ROBO2,SH3GL3,SYT1,SYT4,TCF3,ZEB1,THBS2,TPBG,TRPC6,LRP8,SNX3,NRP1,SPAG9,DLG5,PLAA,RAPGEF2,RIMS2,ZEB2,MAGI2,RNF10,SEMA3A,SEMA4D,CPEB3,NLGN1,CLSTN1,SHANK2,RIMS1,RAB21,SPEN,ZNF609,SETX,CYFIP1,NEDD4L,CRTC1,UFL1,ADNP,DICER1,MMD,ZMYND8,FLRT3,SS18L1,LRRTM2,NPTN,DISC1,FAIM,TENM3,TMEM30A,SMURF1,LRRC7,LRRN1,TGIF2,NDRG4,SLITRK6,SLITRK2,ZNF804A,DAB2IP,LINGO2,LRRTM3 |
| 6_Member | GO Biological Processes | GO:0010720 | positive regulation of cell development | -16.50896446 | -14.016 | 108/553 | 27,375,596,604,627,650,659,998,1002,1268,1272,1399,1499,1826,1906,2033,2042,2043,2048,2244,2266,2332,2624,2736,2737,2803,3309,3655,3815,4036,4076,4131,4208,4760,4897,4916,5048,5080,5270,5361,5468,5587,5597,5629,5728,5756,5764,5789,5879,5915,5978,5991,6009,6091,6092,6457,6857,6860,6929,6935,7225,7804,8600,8724,8829,8874,9043,9231,9373,9472,9693,9699,9839,9863,10221,10371,10507,10512,22849,22871,22941,22999,23011,23013,23060,23064,23191,23327,23373,23376,23394,23405,23531,23613,26039,27020,27185,55179,55714,55754,57154,57554,57580,60436,64855,65009,91752,153090 | ABL2,ARF1,BCL2,BCL6,BDNF,BMP2,BMPR2,CDC42,CDH4,CNR1,CNTN1,CRKL,CTNNB1,DSCAM,EDN1,EP300,EPHA3,EPHA4,EPHB2,FGB,FGG,FMR1,GATA2,GLI2,GLI3,GOLGA4,HSPA5,ITGA6,KIT,LRP2,CAPRIN1,MAP1B,MEF2C,NEUROD1,NRCAM,NTRK3,PAFAH1B1,PAX6,SERPINE2,PLXNA1,PPARG,PRKD1,MAPK6,PROX1,PTEN,TWF1,PTN,PTPRD,RAC1,RARB,REST,RFX3,RHEB,ROBO1,ROBO2,SH3GL3,SYT1,SYT4,TCF3,ZEB1,TRPC6,LRP8,TNFSF11,SNX3,NRP1,ARHGEF7,SPAG9,DLG5,PLAA,AKAP6,RAPGEF2,RIMS2,ZEB2,MAGI2,TRIB1,SEMA3A,SEMA4D,SEMA3C,CPEB3,NLGN1,SHANK2,RIMS1,RAB21,SPEN,ZNF609,SETX,CYFIP1,NEDD4L,CRTC1,UFL1,ADNP,DICER1,MMD,ZMYND8,SS18L1,NPTN,DISC1,FAIM,TENM3,TMEM30A,SMURF1,LRRC7,PREX1,TGIF2,NIBAN2,NDRG4,ZNF804A,DAB2IP |
| 6_Member | GO Biological Processes | GO:0022604 | regulation of cell morphogenesis | -16.09966316 | -13.652 | 98/484 | 102,390,627,659,998,1002,1123,1399,1739,1808,1826,2043,2045,2048,2049,2244,2258,2266,2332,2768,2803,3475,3815,4076,4131,4133,4627,4628,4897,4916,5028,5048,5361,5530,5728,5764,5789,5879,5911,5921,6091,6092,6383,6857,6860,7225,7337,7430,7750,7804,8829,8874,9373,9693,9699,9706,9723,9839,9948,10371,10395,10501,10507,10512,10672,10716,10979,22871,22999,23011,23077,23189,23191,23327,23370,23394,23603,26039,26052,27185,55023,55435,55785,56920,56990,57154,57381,57520,57580,57688,58480,80031,84894,118987,152503,192670,221061,254065 | ADAM10,RND3,BDNF,BMPR2,CDC42,CDH4,CHN1,CRKL,DLG1,DPYSL2,DSCAM,EPHA4,EPHA7,EPHB2,EPHB3,FGB,FGF13,FGG,FMR1,GNA12,GOLGA4,IFRD1,KIT,CAPRIN1,MAP1B,MAP2,MYH9,MYH10,NRCAM,NTRK3,P2RY1,PAFAH1B1,PLXNA1,PPP3CA,PTEN,PTN,PTPRD,RAC1,RAP2A,RASA1,ROBO1,ROBO2,SDC2,SYT1,SYT4,TRPC6,UBE3A,EZR,ZMYM2,LRP8,NRP1,ARHGEF7,PLAA,RAPGEF2,RIMS2,ULK2,SEMA3E,ZEB2,WDR1,SEMA3A,DLC1,SEMA6B,SEMA4D,SEMA3C,GNA13,TBR1,FERMT2,NLGN1,RIMS1,RAB21,MYCBP2,KANK1,CYFIP1,NEDD4L,ARHGEF18,ADNP,CORO1C,SS18L1,DNM3,DISC1,PHIP,AP1AR,FGD6,SEMA3G,CDC42SE2,SMURF1,RHOJ,HECW2,PREX1,ZSWIM6,RHOU,SEMA6D,LINGO1,PDZD8,SH3D19,AGO4,FAM171A1,BRWD3 |
| 6_Member | GO Biological Processes | GO:0050769 | positive regulation of neurogenesis | -15.32634335 | -12.930 | 95/474 | 27,375,596,604,627,650,659,1002,1268,1272,1399,1499,1826,2033,2042,2043,2048,2332,2624,2736,2737,2803,3309,3655,3815,4036,4076,4131,4208,4760,4897,4916,5048,5080,5270,5361,5468,5587,5597,5629,5728,5756,5764,5789,5915,5978,6009,6091,6092,6457,6857,6860,6929,6935,7225,7804,8724,8829,9043,9231,9373,9693,9699,9839,9863,10371,10507,22849,22871,22941,22999,23011,23013,23060,23064,23191,23327,23373,23376,23394,23405,23531,23613,26039,27020,27185,55179,55714,55754,57154,57554,60436,65009,91752,153090 | ABL2,ARF1,BCL2,BCL6,BDNF,BMP2,BMPR2,CDH4,CNR1,CNTN1,CRKL,CTNNB1,DSCAM,EP300,EPHA3,EPHA4,EPHB2,FMR1,GATA2,GLI2,GLI3,GOLGA4,HSPA5,ITGA6,KIT,LRP2,CAPRIN1,MAP1B,MEF2C,NEUROD1,NRCAM,NTRK3,PAFAH1B1,PAX6,SERPINE2,PLXNA1,PPARG,PRKD1,MAPK6,PROX1,PTEN,TWF1,PTN,PTPRD,RARB,REST,RHEB,ROBO1,ROBO2,SH3GL3,SYT1,SYT4,TCF3,ZEB1,TRPC6,LRP8,SNX3,NRP1,SPAG9,DLG5,PLAA,RAPGEF2,RIMS2,ZEB2,MAGI2,SEMA3A,SEMA4D,CPEB3,NLGN1,SHANK2,RIMS1,RAB21,SPEN,ZNF609,SETX,CYFIP1,NEDD4L,CRTC1,UFL1,ADNP,DICER1,MMD,ZMYND8,SS18L1,NPTN,DISC1,FAIM,TENM3,TMEM30A,SMURF1,LRRC7,TGIF2,NDRG4,ZNF804A,DAB2IP |
| 6_Member | GO Biological Processes | GO:0031346 | positive regulation of cell projection organization | -14.50761804 | -12.194 | 81/383 | 27,324,375,627,659,998,1002,1268,1272,1826,2033,2042,2043,2150,2332,2803,2823,3064,3309,3655,3815,4076,4131,4916,5048,5361,5587,5597,5756,5764,5789,5879,5898,6091,6092,6857,6860,7046,7804,8724,8829,8874,8976,9231,9373,9693,9699,9839,9863,10178,10507,10787,22849,22871,22941,22999,23011,23064,23191,23327,23373,23394,23603,23613,26039,26052,26053,27020,27185,51762,54874,55704,55714,55754,57154,57554,65009,89795,91752,153090,202018 | ABL2,APC,ARF1,BDNF,BMPR2,CDC42,CDH4,CNR1,CNTN1,DSCAM,EP300,EPHA3,EPHA4,F2RL1,FMR1,GOLGA4,GPM6A,HTT,HSPA5,ITGA6,KIT,CAPRIN1,MAP1B,NTRK3,PAFAH1B1,PLXNA1,PRKD1,MAPK6,TWF1,PTN,PTPRD,RAC1,RALA,ROBO1,ROBO2,SYT1,SYT4,TGFBR1,LRP8,SNX3,NRP1,ARHGEF7,WASL,DLG5,PLAA,RAPGEF2,RIMS2,ZEB2,MAGI2,TENM1,SEMA4D,NCKAP1,CPEB3,NLGN1,SHANK2,RIMS1,RAB21,SETX,CYFIP1,NEDD4L,CRTC1,ADNP,CORO1C,ZMYND8,SS18L1,DNM3,AUTS2,NPTN,DISC1,RAB8B,FNBP1L,CCDC88A,TENM3,TMEM30A,SMURF1,LRRC7,NDRG4,NAV3,ZNF804A,DAB2IP,TAPT1 |
| 6_Member | GO Biological Processes | GO:0045666 | positive regulation of neuron differentiation | -14.3362391 | -12.046 | 79/371 | 27,375,596,604,627,650,659,1002,1268,1272,1826,2033,2042,2043,2332,2624,2736,2803,3309,3655,4076,4131,4208,4760,4897,4916,5048,5361,5587,5597,5629,5728,5756,5764,5789,5915,5978,6091,6092,6457,6857,6860,6929,6935,7225,7804,8724,8829,9043,9231,9373,9693,9699,9839,9863,10507,22849,22871,22941,22999,23011,23064,23191,23327,23373,23394,23531,23613,26039,27020,27185,55714,55754,57154,57554,60436,65009,91752,153090 | ABL2,ARF1,BCL2,BCL6,BDNF,BMP2,BMPR2,CDH4,CNR1,CNTN1,DSCAM,EP300,EPHA3,EPHA4,FMR1,GATA2,GLI2,GOLGA4,HSPA5,ITGA6,CAPRIN1,MAP1B,MEF2C,NEUROD1,NRCAM,NTRK3,PAFAH1B1,PLXNA1,PRKD1,MAPK6,PROX1,PTEN,TWF1,PTN,PTPRD,RARB,REST,ROBO1,ROBO2,SH3GL3,SYT1,SYT4,TCF3,ZEB1,TRPC6,LRP8,SNX3,NRP1,SPAG9,DLG5,PLAA,RAPGEF2,RIMS2,ZEB2,MAGI2,SEMA4D,CPEB3,NLGN1,SHANK2,RIMS1,RAB21,SETX,CYFIP1,NEDD4L,CRTC1,ADNP,MMD,ZMYND8,SS18L1,NPTN,DISC1,TENM3,TMEM30A,SMURF1,LRRC7,TGIF2,NDRG4,ZNF804A,DAB2IP |
| 6_Member | GO Biological Processes | GO:0060560 | developmental growth involved in morphogenesis | -13.57144208 | -11.328 | 58/235 | 627,659,780,1002,1499,1808,1826,2045,2255,2258,2263,2803,3239,3475,3688,4040,4131,4133,4897,4916,5048,5361,5590,6299,6857,6860,7048,7414,8239,8829,9201,9355,9373,9699,9706,9723,9839,9863,10252,10371,10479,10501,10507,10512,22999,23011,23191,23327,23394,23767,26053,27185,51804,56920,57154,80031,85407,157506 | BDNF,BMPR2,DDR1,CDH4,CTNNB1,DPYSL2,DSCAM,EPHA7,FGF10,FGF13,FGFR2,GOLGA4,HOXD13,IFRD1,ITGB1,LRP6,MAP1B,MAP2,NRCAM,NTRK3,PAFAH1B1,PLXNA1,PRKCZ,SALL1,SYT1,SYT4,TGFBR2,VCL,USP9X,NRP1,DCLK1,LHX2,PLAA,RIMS2,ULK2,SEMA3E,ZEB2,MAGI2,SPRY1,SEMA3A,SLC9A6,SEMA6B,SEMA4D,SEMA3C,RIMS1,RAB21,CYFIP1,NEDD4L,ADNP,FLRT3,AUTS2,DISC1,SIX4,SEMA3G,SMURF1,SEMA6D,NKD1,RDH10 |
| 6_Member | GO Biological Processes | GO:0040008 | regulation of growth | -13.55070921 | -11.311 | 117/682 | 102,323,367,596,604,627,657,659,694,734,780,817,998,1002,1027,1385,1399,1457,1654,1808,1826,1906,1982,2045,2066,2258,2261,2263,2296,2487,2803,3021,3475,3516,3624,3720,4131,4133,4208,4211,4487,4534,4897,4916,5048,5062,5270,5468,5515,5594,5629,5727,5728,6446,6595,6857,6860,7042,7046,7048,7430,7490,7528,8455,8531,8614,8725,8829,9211,9306,9373,9472,9655,9699,9706,9723,10276,10371,10477,10501,10507,10512,11221,22883,22999,23011,23191,23327,23394,23414,23678,26524,27185,28514,29967,51542,51804,54617,55031,55602,55636,55777,55841,56920,57154,79577,80031,80314,83737,84289,84678,85407,116224,131566,136319,153090,285590 | ADAM10,APBB2,AR,BCL2,BCL6,BDNF,BMPR1A,BMPR2,BTG1,OSGIN2,DDR1,CAMK2D,CDC42,CDH4,CDKN1B,CREB1,CRKL,CSNK2A1,DDX3X,DPYSL2,DSCAM,EDN1,EIF4G2,EPHA7,ERBB4,FGF13,FGFR3,FGFR2,FOXC1,FRZB,GOLGA4,H3-3B,IFRD1,RBPJ,INHBA,JARID2,MAP1B,MAP2,MEF2C,MEIS1,MSX1,MTM1,NRCAM,NTRK3,PAFAH1B1,PAK2,SERPINE2,PPARG,PPP2CA,MAPK1,PROX1,PTCH1,PTEN,SGK1,SMARCA2,SYT1,SYT4,TGFB2,TGFBR1,TGFBR2,EZR,WT1,YY1,ATRN,YBX3,STC2,URI1,NRP1,LGI1,SOCS6,PLAA,AKAP6,SOCS5,RIMS2,ULK2,SEMA3E,NET1,SEMA3A,UBE2E3,SEMA6B,SEMA4D,SEMA3C,DUSP10,CLSTN1,RIMS1,RAB21,CYFIP1,NEDD4L,ADNP,ZFPM2,SGK3,LATS2,DISC1,DLL1,LRP12,VPS54,SIX4,INO80,USP47,CDKN2AIP,CHD7,MBD5,WWC3,SEMA3G,SMURF1,CDC73,SEMA6D,EPC1,ITCH,ING5,KDM2B,NKD1,FAM122A,DCBLD2,MTPN,DAB2IP,SH3PXD2B |
| 6_Member | GO Biological Processes | GO:0016049 | cell growth | -13.24074522 | -11.014 | 92/487 | 102,323,596,604,627,659,694,734,780,817,998,1002,1027,1399,1457,1499,1654,1808,1826,1906,1982,2045,2258,2487,2803,3021,3475,3624,3688,4131,4133,4487,4897,4916,5048,5270,5361,5468,5515,5590,6416,6446,6595,6857,6860,7042,7046,7048,7414,7490,7528,8239,8470,8725,8829,9201,9211,9355,9373,9472,9699,9706,9723,9839,10276,10371,10479,10501,10507,10512,22883,22999,23011,23191,23327,23394,23678,23767,26053,27185,54617,55031,55602,56920,57154,79577,80031,83737,84678,116224,131566,136319 | ADAM10,APBB2,BCL2,BCL6,BDNF,BMPR2,BTG1,OSGIN2,DDR1,CAMK2D,CDC42,CDH4,CDKN1B,CRKL,CSNK2A1,CTNNB1,DDX3X,DPYSL2,DSCAM,EDN1,EIF4G2,EPHA7,FGF13,FRZB,GOLGA4,H3-3B,IFRD1,INHBA,ITGB1,MAP1B,MAP2,MSX1,NRCAM,NTRK3,PAFAH1B1,SERPINE2,PLXNA1,PPARG,PPP2CA,PRKCZ,MAP2K4,SGK1,SMARCA2,SYT1,SYT4,TGFB2,TGFBR1,TGFBR2,VCL,WT1,YY1,USP9X,SORBS2,URI1,NRP1,DCLK1,LGI1,LHX2,PLAA,AKAP6,RIMS2,ULK2,SEMA3E,ZEB2,NET1,SEMA3A,SLC9A6,SEMA6B,SEMA4D,SEMA3C,CLSTN1,RIMS1,RAB21,CYFIP1,NEDD4L,ADNP,SGK3,FLRT3,AUTS2,DISC1,INO80,USP47,CDKN2AIP,SEMA3G,SMURF1,CDC73,SEMA6D,ITCH,KDM2B,FAM122A,DCBLD2,MTPN |
| 6_Member | GO Biological Processes | GO:0010769 | regulation of cell morphogenesis involved in differentiation | -12.70666864 | -10.532 | 66/301 | 102,627,659,998,1002,1123,1399,1808,1826,2043,2045,2048,2049,2244,2258,2266,2332,2803,3475,4076,4131,4133,4897,4916,5048,5361,5530,5728,5789,5879,5911,6091,6092,6383,7225,7337,7804,8829,8874,9693,9706,9723,9839,10371,10501,10507,10512,10716,22871,23011,23077,23189,23191,23327,23394,23603,26039,26052,27185,55435,56920,57520,57580,57688,80031,84894 | ADAM10,BDNF,BMPR2,CDC42,CDH4,CHN1,CRKL,DPYSL2,DSCAM,EPHA4,EPHA7,EPHB2,EPHB3,FGB,FGF13,FGG,FMR1,GOLGA4,IFRD1,CAPRIN1,MAP1B,MAP2,NRCAM,NTRK3,PAFAH1B1,PLXNA1,PPP3CA,PTEN,PTPRD,RAC1,RAP2A,ROBO1,ROBO2,SDC2,TRPC6,UBE3A,LRP8,NRP1,ARHGEF7,RAPGEF2,ULK2,SEMA3E,ZEB2,SEMA3A,SEMA6B,SEMA4D,SEMA3C,TBR1,NLGN1,RAB21,MYCBP2,KANK1,CYFIP1,NEDD4L,ADNP,CORO1C,SS18L1,DNM3,DISC1,AP1AR,SEMA3G,HECW2,PREX1,ZSWIM6,SEMA6D,LINGO1 |
| 6_Member | GO Biological Processes | GO:0048638 | regulation of developmental growth | -12.53438444 | -10.371 | 72/347 | 367,596,627,657,659,1002,1027,1385,1808,1826,1906,2045,2066,2258,2261,2263,2296,2803,3475,3516,3720,4131,4133,4208,4211,4534,4897,4916,5048,5594,5629,5727,5728,6857,6860,7046,7048,7430,7490,7528,8455,8531,8614,8829,9373,9472,9699,9706,9723,10371,10501,10507,10512,11221,22999,23011,23191,23327,23394,23414,26524,27185,28514,51804,55636,55777,55841,56920,57154,80031,85407,285590 | AR,BCL2,BDNF,BMPR1A,BMPR2,CDH4,CDKN1B,CREB1,DPYSL2,DSCAM,EDN1,EPHA7,ERBB4,FGF13,FGFR3,FGFR2,FOXC1,GOLGA4,IFRD1,RBPJ,JARID2,MAP1B,MAP2,MEF2C,MEIS1,MTM1,NRCAM,NTRK3,PAFAH1B1,MAPK1,PROX1,PTCH1,PTEN,SYT1,SYT4,TGFBR1,TGFBR2,EZR,WT1,YY1,ATRN,YBX3,STC2,NRP1,PLAA,AKAP6,RIMS2,ULK2,SEMA3E,SEMA3A,SEMA6B,SEMA4D,SEMA3C,DUSP10,RIMS1,RAB21,CYFIP1,NEDD4L,ADNP,ZFPM2,LATS2,DISC1,DLL1,SIX4,CHD7,MBD5,WWC3,SEMA3G,SMURF1,SEMA6D,NKD1,SH3PXD2B |
| 6_Member | GO Biological Processes | GO:0010976 | positive regulation of neuron projection development | -11.10055566 | -9.026 | 60/281 | 27,375,627,659,1002,1268,1272,1826,2033,2042,2043,2332,2803,3309,3655,4076,4131,4916,5048,5361,5587,5597,5756,5764,5789,6091,6092,6857,6860,7804,8724,8829,9231,9373,9693,9699,9839,9863,10507,22849,22871,22941,22999,23011,23064,23191,23327,23373,23394,23613,26039,27020,27185,55714,55754,57154,57554,65009,91752,153090 | ABL2,ARF1,BDNF,BMPR2,CDH4,CNR1,CNTN1,DSCAM,EP300,EPHA3,EPHA4,FMR1,GOLGA4,HSPA5,ITGA6,CAPRIN1,MAP1B,NTRK3,PAFAH1B1,PLXNA1,PRKD1,MAPK6,TWF1,PTN,PTPRD,ROBO1,ROBO2,SYT1,SYT4,LRP8,SNX3,NRP1,DLG5,PLAA,RAPGEF2,RIMS2,ZEB2,MAGI2,SEMA4D,CPEB3,NLGN1,SHANK2,RIMS1,RAB21,SETX,CYFIP1,NEDD4L,CRTC1,ADNP,ZMYND8,SS18L1,NPTN,DISC1,TENM3,TMEM30A,SMURF1,LRRC7,NDRG4,ZNF804A,DAB2IP |
| 6_Member | GO Biological Processes | GO:0048588 | developmental cell growth | -10.90482333 | -8.844 | 53/234 | 627,659,780,1002,1499,1808,1826,1906,2045,2258,2803,3475,3688,4131,4133,4897,4916,5048,5361,5590,6416,6857,6860,7048,7414,7528,8239,8470,8829,9201,9355,9373,9472,9699,9706,9723,9839,10371,10479,10501,10507,10512,22999,23011,23191,23327,23394,23767,26053,27185,56920,57154,80031 | BDNF,BMPR2,DDR1,CDH4,CTNNB1,DPYSL2,DSCAM,EDN1,EPHA7,FGF13,GOLGA4,IFRD1,ITGB1,MAP1B,MAP2,NRCAM,NTRK3,PAFAH1B1,PLXNA1,PRKCZ,MAP2K4,SYT1,SYT4,TGFBR2,VCL,YY1,USP9X,SORBS2,NRP1,DCLK1,LHX2,PLAA,AKAP6,RIMS2,ULK2,SEMA3E,ZEB2,SEMA3A,SLC9A6,SEMA6B,SEMA4D,SEMA3C,RIMS1,RAB21,CYFIP1,NEDD4L,ADNP,FLRT3,AUTS2,DISC1,SEMA3G,SMURF1,SEMA6D |
| 6_Member | GO Biological Processes | GO:1990138 | neuron projection extension | -10.80017151 | -8.755 | 43/168 | 659,780,1002,1499,1808,1826,2803,3475,3688,4131,4133,4897,4916,5048,5361,5590,6857,6860,7414,8239,8829,9201,9355,9373,9699,9706,9723,10371,10479,10501,10507,10512,22999,23011,23191,23327,23394,23767,26053,27185,56920,57154,80031 | BMPR2,DDR1,CDH4,CTNNB1,DPYSL2,DSCAM,GOLGA4,IFRD1,ITGB1,MAP1B,MAP2,NRCAM,NTRK3,PAFAH1B1,PLXNA1,PRKCZ,SYT1,SYT4,VCL,USP9X,NRP1,DCLK1,LHX2,PLAA,RIMS2,ULK2,SEMA3E,SEMA3A,SLC9A6,SEMA6B,SEMA4D,SEMA3C,RIMS1,RAB21,CYFIP1,NEDD4L,ADNP,FLRT3,AUTS2,DISC1,SEMA3G,SMURF1,SEMA6D |
| 6_Member | GO Biological Processes | GO:0001558 | regulation of cell growth | -10.57397238 | -8.558 | 77/419 | 102,323,596,604,627,659,694,734,780,817,998,1002,1027,1399,1457,1654,1808,1826,1906,1982,2045,2258,2487,2803,3021,3475,3624,4131,4133,4487,4897,4916,5048,5270,5468,5515,6446,6595,6857,6860,7042,7046,7490,7528,8725,8829,9211,9373,9472,9699,9706,9723,10276,10371,10501,10507,10512,22883,22999,23011,23191,23327,23394,23678,27185,54617,55031,55602,56920,57154,79577,80031,83737,84678,116224,131566,136319 | ADAM10,APBB2,BCL2,BCL6,BDNF,BMPR2,BTG1,OSGIN2,DDR1,CAMK2D,CDC42,CDH4,CDKN1B,CRKL,CSNK2A1,DDX3X,DPYSL2,DSCAM,EDN1,EIF4G2,EPHA7,FGF13,FRZB,GOLGA4,H3-3B,IFRD1,INHBA,MAP1B,MAP2,MSX1,NRCAM,NTRK3,PAFAH1B1,SERPINE2,PPARG,PPP2CA,SGK1,SMARCA2,SYT1,SYT4,TGFB2,TGFBR1,WT1,YY1,URI1,NRP1,LGI1,PLAA,AKAP6,RIMS2,ULK2,SEMA3E,NET1,SEMA3A,SEMA6B,SEMA4D,SEMA3C,CLSTN1,RIMS1,RAB21,CYFIP1,NEDD4L,ADNP,SGK3,DISC1,INO80,USP47,CDKN2AIP,SEMA3G,SMURF1,CDC73,SEMA6D,ITCH,KDM2B,FAM122A,DCBLD2,MTPN |
| 6_Member | GO Biological Processes | GO:0045927 | positive regulation of growth | -9.421768369 | -7.499 | 55/270 | 102,596,627,657,659,998,1002,1385,1457,1654,1826,1906,1982,2066,2263,2803,3021,3516,4131,4208,4534,4916,5048,5594,5629,6857,6860,7042,7046,7048,7430,7490,8531,8829,9211,9373,9472,9699,10507,22999,23191,23327,23394,23414,27185,28514,54617,55031,55602,55636,57154,84678,116224,136319,285590 | ADAM10,BCL2,BDNF,BMPR1A,BMPR2,CDC42,CDH4,CREB1,CSNK2A1,DDX3X,DSCAM,EDN1,EIF4G2,ERBB4,FGFR2,GOLGA4,H3-3B,RBPJ,MAP1B,MEF2C,MTM1,NTRK3,PAFAH1B1,MAPK1,PROX1,SYT1,SYT4,TGFB2,TGFBR1,TGFBR2,EZR,WT1,YBX3,NRP1,LGI1,PLAA,AKAP6,RIMS2,SEMA4D,RIMS1,CYFIP1,NEDD4L,ADNP,ZFPM2,DISC1,DLL1,INO80,USP47,CDKN2AIP,CHD7,SMURF1,KDM2B,FAM122A,MTPN,SH3PXD2B |
| 6_Member | GO Biological Processes | GO:0048675 | axon extension | -7.993528474 | -6.192 | 31/121 | 659,1002,1808,1826,2803,3475,3688,4131,4133,4897,4916,5048,7414,8239,8829,9201,9355,9706,9723,10371,10479,10501,10507,10512,23011,23191,23394,26053,27185,56920,80031 | BMPR2,CDH4,DPYSL2,DSCAM,GOLGA4,IFRD1,ITGB1,MAP1B,MAP2,NRCAM,NTRK3,PAFAH1B1,VCL,USP9X,NRP1,DCLK1,LHX2,ULK2,SEMA3E,SEMA3A,SLC9A6,SEMA6B,SEMA4D,SEMA3C,RAB21,CYFIP1,ADNP,AUTS2,DISC1,SEMA3G,SEMA6D |
| 6_Member | GO Biological Processes | GO:0050770 | regulation of axonogenesis | -7.93562659 | -6.141 | 40/183 | 627,659,1002,1123,1808,1826,2043,2045,2048,2049,2258,2803,3475,4131,4133,4897,4916,5048,5361,5728,6091,6092,8829,9706,9723,9839,10371,10501,10507,10512,10716,23011,23077,23191,23394,27185,56920,57688,80031,84894 | BDNF,BMPR2,CDH4,CHN1,DPYSL2,DSCAM,EPHA4,EPHA7,EPHB2,EPHB3,FGF13,GOLGA4,IFRD1,MAP1B,MAP2,NRCAM,NTRK3,PAFAH1B1,PLXNA1,PTEN,ROBO1,ROBO2,NRP1,ULK2,SEMA3E,ZEB2,SEMA3A,SEMA6B,SEMA4D,SEMA3C,TBR1,RAB21,MYCBP2,CYFIP1,ADNP,DISC1,SEMA3G,ZSWIM6,SEMA6D,LINGO1 |
| 6_Member | GO Biological Processes | GO:0030307 | positive regulation of cell growth | -7.926437797 | -6.133 | 38/169 | 102,596,627,659,998,1002,1457,1654,1826,1906,1982,2803,3021,4131,4916,5048,6857,6860,7042,7046,8829,9211,9373,9472,9699,10507,22999,23191,23327,23394,27185,54617,55031,55602,57154,84678,116224,136319 | ADAM10,BCL2,BDNF,BMPR2,CDC42,CDH4,CSNK2A1,DDX3X,DSCAM,EDN1,EIF4G2,GOLGA4,H3-3B,MAP1B,NTRK3,PAFAH1B1,SYT1,SYT4,TGFB2,TGFBR1,NRP1,LGI1,PLAA,AKAP6,RIMS2,SEMA4D,RIMS1,CYFIP1,NEDD4L,ADNP,DISC1,INO80,USP47,CDKN2AIP,SMURF1,KDM2B,FAM122A,MTPN |
| 6_Member | GO Biological Processes | GO:0048639 | positive regulation of developmental growth | -7.8649967 | -6.076 | 40/184 | 596,627,657,659,1002,1385,1826,1906,2066,2263,2803,3516,4131,4208,4534,4916,5048,5594,5629,6857,6860,7048,7430,7490,8531,8829,9373,9472,9699,10507,22999,23191,23327,23394,23414,27185,28514,55636,57154,285590 | BCL2,BDNF,BMPR1A,BMPR2,CDH4,CREB1,DSCAM,EDN1,ERBB4,FGFR2,GOLGA4,RBPJ,MAP1B,MEF2C,MTM1,NTRK3,PAFAH1B1,MAPK1,PROX1,SYT1,SYT4,TGFBR2,EZR,WT1,YBX3,NRP1,PLAA,AKAP6,RIMS2,SEMA4D,RIMS1,CYFIP1,NEDD4L,ADNP,ZFPM2,DISC1,DLL1,CHD7,SMURF1,SH3PXD2B |
| 6_Member | GO Biological Processes | GO:0008361 | regulation of cell size | -7.64381977 | -5.878 | 39/180 | 361,627,659,1002,1182,1185,1385,1808,1826,1906,2045,2258,2803,3475,4131,4133,4897,4916,5048,5728,5879,6558,7430,8829,9706,9723,9821,10000,10371,10501,10507,10512,23011,23191,23394,27185,56920,80031,136319 | AQP4,BDNF,BMPR2,CDH4,CLCN3,CLCN6,CREB1,DPYSL2,DSCAM,EDN1,EPHA7,FGF13,GOLGA4,IFRD1,MAP1B,MAP2,NRCAM,NTRK3,PAFAH1B1,PTEN,RAC1,SLC12A2,EZR,NRP1,ULK2,SEMA3E,RB1CC1,AKT3,SEMA3A,SEMA6B,SEMA4D,SEMA3C,RAB21,CYFIP1,ADNP,DISC1,SEMA3G,SEMA6D,MTPN |
| 6_Member | GO Biological Processes | GO:0061387 | regulation of extent of cell growth | -6.639078425 | -4.972 | 27/110 | 627,659,1002,1808,1826,2045,2258,2803,3475,4131,4133,4897,4916,5048,8829,9706,9723,10371,10501,10507,10512,23011,23191,23394,27185,56920,80031 | BDNF,BMPR2,CDH4,DPYSL2,DSCAM,EPHA7,FGF13,GOLGA4,IFRD1,MAP1B,MAP2,NRCAM,NTRK3,PAFAH1B1,NRP1,ULK2,SEMA3E,SEMA3A,SEMA6B,SEMA4D,SEMA3C,RAB21,CYFIP1,ADNP,DISC1,SEMA3G,SEMA6D |
| 6_Member | GO Biological Processes | GO:0030516 | regulation of axon extension | -5.559581928 | -4.030 | 23/96 | 659,1002,1808,1826,2803,3475,4131,4133,4897,4916,5048,8829,9723,10371,10501,10507,10512,23011,23191,23394,27185,56920,80031 | BMPR2,CDH4,DPYSL2,DSCAM,GOLGA4,IFRD1,MAP1B,MAP2,NRCAM,NTRK3,PAFAH1B1,NRP1,SEMA3E,SEMA3A,SEMA6B,SEMA4D,SEMA3C,RAB21,CYFIP1,ADNP,DISC1,SEMA3G,SEMA6D |
| 6_Member | GO Biological Processes | GO:0010770 | positive regulation of cell morphogenesis involved in differentiation | -5.044884237 | -3.577 | 30/154 | 627,659,998,1002,1399,1826,2043,2244,2266,2803,4076,4131,4916,5048,5361,5789,5879,6091,6092,7804,8829,8874,9839,10507,23011,23191,23394,26039,27185,57580 | BDNF,BMPR2,CDC42,CDH4,CRKL,DSCAM,EPHA4,FGB,FGG,GOLGA4,CAPRIN1,MAP1B,NTRK3,PAFAH1B1,PLXNA1,PTPRD,RAC1,ROBO1,ROBO2,LRP8,NRP1,ARHGEF7,ZEB2,SEMA4D,RAB21,CYFIP1,ADNP,SS18L1,DISC1,PREX1 |
| 6_Member | GO Biological Processes | GO:0050772 | positive regulation of axonogenesis | -3.263712158 | -2.061 | 17/85 | 627,659,1002,1826,2803,4131,4916,5048,5361,6091,6092,8829,9839,10507,23191,23394,27185 | BDNF,BMPR2,CDH4,DSCAM,GOLGA4,MAP1B,NTRK3,PAFAH1B1,PLXNA1,ROBO1,ROBO2,NRP1,ZEB2,SEMA4D,CYFIP1,ADNP,DISC1 |
| 6_Member | GO Biological Processes | GO:0045773 | positive regulation of axon extension | -3.222555921 | -2.030 | 11/43 | 659,1002,1826,2803,4131,4916,5048,8829,23191,23394,27185 | BMPR2,CDH4,DSCAM,GOLGA4,MAP1B,NTRK3,PAFAH1B1,NRP1,CYFIP1,ADNP,DISC1 |
| 7_Summary | GO Molecular Functions | GO:0008134 | transcription factor binding | -22.86807817 | -19.840 | 136/661 | 60,196,323,367,596,1385,1387,1390,1488,1499,1654,1655,1810,1871,1875,1977,2033,2113,2186,2295,2296,2308,2624,2736,2957,2959,3184,3192,3204,3301,3516,3720,4005,4208,4664,4760,4773,4775,4848,4929,5080,5295,5308,5468,5594,5629,5757,5901,5915,5978,6095,6096,6497,6598,6717,6801,6873,6874,6875,6883,6908,6913,6929,6934,6935,6996,7003,7004,7029,7068,7088,7158,7329,7534,7874,7994,8013,8202,8204,8289,8543,8553,8554,8621,8850,9079,9314,9320,9324,9355,9519,9686,9734,9967,9969,10054,10221,10463,10499,10716,10725,10891,11124,23013,23186,23269,23368,23373,23414,23543,23613,25879,26043,28514,28951,29883,29969,51141,51701,54880,55689,55758,55832,57178,57649,57708,79365,79733,91748,92140,93986,144455,145258,150094,166968,221037,865,1602,2294,2494,3182,3205,4091,4211,4841,5090,5451,5991,6421,7080,7528,9329,9443,9575,10625,23314,25937,51804,80155,83741,1024,1490,2908,2969,4306,5431,5728,7182,9794,22976,29959,55534,4286,8467,23064 | ACTB,AHR,APBB2,AR,BCL2,CREB1,CREBBP,CREM,CTBP2,CTNNB1,DDX3X,DDX5,DR1,E2F3,E2F5,EIF4E,EP300,ETS1,BPTF,FOXF2,FOXC1,FOXO1,GATA2,GLI2,GTF2A1,GTF2B,HNRNPD,HNRNPU,HOXA7,DNAJA1,RBPJ,JARID2,LMO2,MEF2C,NAB1,NEUROD1,NFATC2,NFATC3,CNOT2,NR4A2,PAX6,PIK3R1,PITX2,PPARG,MAPK1,PROX1,PTMA,RAN,RARB,REST,RORA,RORB,SKI,SMARCB1,SRI,STRN,TAF2,TAF4,TAF4B,TAF12,TBP,TBX15,TCF3,TCF7L2,ZEB1,TDG,TEAD1,TEAD4,TFDP2,THRB,TLE1,TP53BP1,UBE2I,YWHAZ,USP7,KAT6A,NR4A3,NCOA3,NRIP1,ARID1A,LMO4,BHLHE40,PIAS1,CDK13,KAT2B,LDB2,KLF4,TRIP12,HMGN3,LHX2,TBPL1,VGLL4,HDAC9,THRAP3,MED13,UBA2,TRIB1,SLC30A9,NCOA2,TBR1,NFAT5,PPARGC1A,FAF1,SPEN,RCOR1,MGA,PPP1R13B,CRTC1,ZFPM2,RBFOX2,ZMYND8,DCAF13,UBXN7,DLL1,TRIB2,CNOT7,MDFIC,INSIG2,NLK,BCOR,YEATS2,RCOR3,CAND1,ZMIZ1,PHF12,MIER1,BHLHE41,E2F8,ELMSAN1,MTDH,FOXP2,E2F7,GSC,SIK1,MIER3,JMJD1C,CBFB,DACH1,FOXF1,NR5A2,HNRNPAB,HOXA9,SMAD6,MEIS1,NONO,PBX3,POU2F1,RFX3,SFPQ,NKX2-1,YY1,GTF3C4,MED7,CLOCK,IVNS1ABP,SATB2,WWTR1,SIX4,NAA15,TFAP2D,CDK8,CCN2,NR3C1,GTF2I,NR3C2,POLR2B,PTEN,NR2C2,MAML1,PAXIP1,NRBP1,MAML3,MITF,SMARCA5,SETX |
| 7_Member | GO Molecular Functions | GO:0008134 | transcription factor binding | -22.86807817 | -19.840 | 136/661 | 60,196,323,367,596,1385,1387,1390,1488,1499,1654,1655,1810,1871,1875,1977,2033,2113,2186,2295,2296,2308,2624,2736,2957,2959,3184,3192,3204,3301,3516,3720,4005,4208,4664,4760,4773,4775,4848,4929,5080,5295,5308,5468,5594,5629,5757,5901,5915,5978,6095,6096,6497,6598,6717,6801,6873,6874,6875,6883,6908,6913,6929,6934,6935,6996,7003,7004,7029,7068,7088,7158,7329,7534,7874,7994,8013,8202,8204,8289,8543,8553,8554,8621,8850,9079,9314,9320,9324,9355,9519,9686,9734,9967,9969,10054,10221,10463,10499,10716,10725,10891,11124,23013,23186,23269,23368,23373,23414,23543,23613,25879,26043,28514,28951,29883,29969,51141,51701,54880,55689,55758,55832,57178,57649,57708,79365,79733,91748,92140,93986,144455,145258,150094,166968,221037 | ACTB,AHR,APBB2,AR,BCL2,CREB1,CREBBP,CREM,CTBP2,CTNNB1,DDX3X,DDX5,DR1,E2F3,E2F5,EIF4E,EP300,ETS1,BPTF,FOXF2,FOXC1,FOXO1,GATA2,GLI2,GTF2A1,GTF2B,HNRNPD,HNRNPU,HOXA7,DNAJA1,RBPJ,JARID2,LMO2,MEF2C,NAB1,NEUROD1,NFATC2,NFATC3,CNOT2,NR4A2,PAX6,PIK3R1,PITX2,PPARG,MAPK1,PROX1,PTMA,RAN,RARB,REST,RORA,RORB,SKI,SMARCB1,SRI,STRN,TAF2,TAF4,TAF4B,TAF12,TBP,TBX15,TCF3,TCF7L2,ZEB1,TDG,TEAD1,TEAD4,TFDP2,THRB,TLE1,TP53BP1,UBE2I,YWHAZ,USP7,KAT6A,NR4A3,NCOA3,NRIP1,ARID1A,LMO4,BHLHE40,PIAS1,CDK13,KAT2B,LDB2,KLF4,TRIP12,HMGN3,LHX2,TBPL1,VGLL4,HDAC9,THRAP3,MED13,UBA2,TRIB1,SLC30A9,NCOA2,TBR1,NFAT5,PPARGC1A,FAF1,SPEN,RCOR1,MGA,PPP1R13B,CRTC1,ZFPM2,RBFOX2,ZMYND8,DCAF13,UBXN7,DLL1,TRIB2,CNOT7,MDFIC,INSIG2,NLK,BCOR,YEATS2,RCOR3,CAND1,ZMIZ1,PHF12,MIER1,BHLHE41,E2F8,ELMSAN1,MTDH,FOXP2,E2F7,GSC,SIK1,MIER3,JMJD1C |
| 7_Member | GO Cellular Components | GO:0005667 | transcription factor complex | -11.41860295 | -9.318 | 72/365 | 196,865,1385,1390,1499,1602,1871,1875,2033,2113,2294,2295,2494,2957,2959,3182,3192,3205,3516,4005,4091,4211,4760,4773,4775,4841,4929,5090,5308,5451,5468,5915,5991,6421,6497,6873,6874,6875,6883,6908,6929,6934,6935,7003,7004,7029,7068,7080,7088,7528,8013,8543,9079,9314,9329,9443,9519,9575,9734,10625,10725,23186,23314,25937,51804,55758,79733,80155,83741,91748,144455,145258 | AHR,CBFB,CREB1,CREM,CTNNB1,DACH1,E2F3,E2F5,EP300,ETS1,FOXF1,FOXF2,NR5A2,GTF2A1,GTF2B,HNRNPAB,HNRNPU,HOXA9,RBPJ,LMO2,SMAD6,MEIS1,NEUROD1,NFATC2,NFATC3,NONO,NR4A2,PBX3,PITX2,POU2F1,PPARG,RARB,RFX3,SFPQ,SKI,TAF2,TAF4,TAF4B,TAF12,TBP,TCF3,TCF7L2,ZEB1,TEAD1,TEAD4,TFDP2,THRB,NKX2-1,TLE1,YY1,NR4A3,LMO4,LDB2,KLF4,GTF3C4,MED7,TBPL1,CLOCK,HDAC9,IVNS1ABP,NFAT5,RCOR1,SATB2,WWTR1,SIX4,RCOR3,E2F8,NAA15,TFAP2D,ELMSAN1,E2F7,GSC |
| 7_Member | GO Biological Processes | GO:0006367 | transcription initiation from RNA polymerase II promoter | -9.121012132 | -7.225 | 43/188 | 367,865,1024,1385,1387,1490,1810,1871,2494,2908,2957,2959,2969,3516,4306,4929,5431,5468,5728,5915,6095,6096,6873,6874,6875,6883,6908,7003,7004,7068,7182,8013,8850,9443,9794,9967,9969,10891,22976,25937,29959,55534,55832 | AR,CBFB,CDK8,CREB1,CREBBP,CCN2,DR1,E2F3,NR5A2,NR3C1,GTF2A1,GTF2B,GTF2I,RBPJ,NR3C2,NR4A2,POLR2B,PPARG,PTEN,RARB,RORA,RORB,TAF2,TAF4,TAF4B,TAF12,TBP,TEAD1,TEAD4,THRB,NR2C2,NR4A3,KAT2B,MED7,MAML1,THRAP3,MED13,PPARGC1A,PAXIP1,WWTR1,NRBP1,MAML3,CAND1 |
| 7_Member | GO Biological Processes | GO:0006352 | DNA-templated transcription, initiation | -8.409172432 | -6.578 | 50/249 | 367,865,1024,1385,1387,1490,1499,1810,1871,2494,2908,2957,2959,2969,3516,4286,4306,4929,5431,5468,5728,5915,6095,6096,6598,6873,6874,6875,6883,6908,7003,7004,7068,7182,8013,8467,8850,9329,9443,9519,9794,9967,9969,10891,22976,23064,25937,29959,55534,55832 | AR,CBFB,CDK8,CREB1,CREBBP,CCN2,CTNNB1,DR1,E2F3,NR5A2,NR3C1,GTF2A1,GTF2B,GTF2I,RBPJ,MITF,NR3C2,NR4A2,POLR2B,PPARG,PTEN,RARB,RORA,RORB,SMARCB1,TAF2,TAF4,TAF4B,TAF12,TBP,TEAD1,TEAD4,THRB,NR2C2,NR4A3,SMARCA5,KAT2B,GTF3C4,MED7,TBPL1,MAML1,THRAP3,MED13,PPARGC1A,PAXIP1,SETX,WWTR1,NRBP1,MAML3,CAND1 |
| 7_Member | GO Cellular Components | GO:0044798 | nuclear transcription factor complex | -4.653787382 | -3.232 | 35/201 | 865,1385,1499,1871,1875,2494,2957,2959,3182,3192,4760,4773,4775,4841,5451,5468,5915,6421,6873,6874,6875,6883,6908,6929,6934,7003,7029,7068,7088,9314,9329,9519,10725,79733,144455 | CBFB,CREB1,CTNNB1,E2F3,E2F5,NR5A2,GTF2A1,GTF2B,HNRNPAB,HNRNPU,NEUROD1,NFATC2,NFATC3,NONO,POU2F1,PPARG,RARB,SFPQ,TAF2,TAF4,TAF4B,TAF12,TBP,TCF3,TCF7L2,TEAD1,TFDP2,THRB,TLE1,KLF4,GTF3C4,TBPL1,NFAT5,E2F8,E2F7 |
| 7_Member | GO Cellular Components | GO:0090575 | RNA polymerase II transcription factor complex | -4.546183052 | -3.141 | 30/163 | 865,1385,1499,1871,1875,2494,2957,2959,3182,3192,4760,4841,5451,5468,5915,6421,6873,6874,6875,6883,6908,6929,6934,7003,7029,7068,7088,9519,79733,144455 | CBFB,CREB1,CTNNB1,E2F3,E2F5,NR5A2,GTF2A1,GTF2B,HNRNPAB,HNRNPU,NEUROD1,NONO,POU2F1,PPARG,RARB,SFPQ,TAF2,TAF4,TAF4B,TAF12,TBP,TCF3,TCF7L2,TEAD1,TFDP2,THRB,TLE1,TBPL1,E2F8,E2F7 |
| 8_Summary | GO Biological Processes | GO:0070848 | response to growth factor | -22.71301199 | -19.724 | 146/739 | 90,92,627,650,657,659,753,858,1028,1385,1387,1399,1490,1499,1655,1749,1783,1906,1958,2033,2066,2201,2241,2252,2255,2261,2263,2296,2729,2730,2734,2887,2908,3096,3175,3309,3516,3688,3746,3747,4036,4052,4091,4131,4208,4487,4691,4842,4916,5028,5151,5156,5170,5431,5494,5587,5590,5594,5725,5728,5764,5770,5796,6091,6093,6198,6497,6591,6622,6653,6660,6885,6935,7042,7046,7048,7050,7057,7073,7080,7314,7321,7323,7337,7412,7804,8036,8239,8573,8725,8829,9101,9197,9314,9315,9372,9392,9475,9480,9508,9530,9693,9863,9958,10140,10252,10468,10479,10818,10891,10979,23064,23090,23191,23213,23370,23543,23592,23767,26060,27020,27250,28514,51496,51552,51701,54206,54778,55553,56937,56975,57154,64094,64399,64750,64855,79633,83891,94056,147372,147409,152831,153090,161742,222008,494470,651,3624,3625,4012,6711,25937,60436,1290,5080,7528,9839 | ACVR1,ACVR2A,BDNF,BMP2,BMPR1A,BMPR2,LDLRAD4,CAV2,CDKN1C,CREB1,CREBBP,CRKL,CCN2,CTNNB1,DDX5,DLX5,DYNC1LI2,EDN1,EGR1,EP300,ERBB4,FBN2,FER,FGF7,FGF10,FGFR3,FGFR2,FOXC1,GCLC,GCLM,GLG1,GRB10,NR3C1,HIVEP1,ONECUT1,HSPA5,RBPJ,ITGB1,KCNC1,KCNC2,LRP2,LTBP1,SMAD6,MAP1B,MEF2C,MSX1,NCL,NOS1,NTRK3,P2RY1,PDE8A,PDGFRA,PDPK1,POLR2B,PPM1A,PRKD1,PRKCZ,MAPK1,PTBP1,PTEN,PTN,PTPN1,PTPRK,ROBO1,ROCK1,RPS6KB1,SKI,SNAI2,SNCA,SORL1,SOX5,MAP3K7,ZEB1,TGFB2,TGFBR1,TGFBR2,TGIF1,THBS1,TIAL1,NKX2-1,UBB,UBE2D1,UBE2D3,UBE3A,VCAM1,LRP8,SHOC2,USP9X,CASK,URI1,NRP1,USP8,SLC33A1,KLF4,NREP,ZFYVE9,TGFBRAP1,ROCK2,ONECUT2,ADAMTS3,BAG4,RAPGEF2,MAGI2,USP15,TOB1,SPRY1,FST,SLC9A6,FRS2,PPARGC1A,FERMT2,SETX,ZNF423,CYFIP1,SULF1,ARHGEF18,RBFOX2,LEMD3,FLRT3,APPL1,NPTN,PDCD4,DLL1,CTDSPL2,RAB14,NLK,ERRFI1,RNF111,SOX6,PMEPA1,FAM20C,SMURF1,SMOC2,HHIP,SMURF2,NIBAN2,FAT4,SNX25,SYAP1,CCBE1,DSG4,KLB,DAB2IP,SPRED1,VSTM2A,RNF165,BMP3,INHBA,INHBB,LNPEP,SPTBN1,WWTR1,TGIF2,COL5A2,PAX6,YY1,ZEB2 |
| 8_Member | GO Biological Processes | GO:0070848 | response to growth factor | -22.71301199 | -19.724 | 146/739 | 90,92,627,650,657,659,753,858,1028,1385,1387,1399,1490,1499,1655,1749,1783,1906,1958,2033,2066,2201,2241,2252,2255,2261,2263,2296,2729,2730,2734,2887,2908,3096,3175,3309,3516,3688,3746,3747,4036,4052,4091,4131,4208,4487,4691,4842,4916,5028,5151,5156,5170,5431,5494,5587,5590,5594,5725,5728,5764,5770,5796,6091,6093,6198,6497,6591,6622,6653,6660,6885,6935,7042,7046,7048,7050,7057,7073,7080,7314,7321,7323,7337,7412,7804,8036,8239,8573,8725,8829,9101,9197,9314,9315,9372,9392,9475,9480,9508,9530,9693,9863,9958,10140,10252,10468,10479,10818,10891,10979,23064,23090,23191,23213,23370,23543,23592,23767,26060,27020,27250,28514,51496,51552,51701,54206,54778,55553,56937,56975,57154,64094,64399,64750,64855,79633,83891,94056,147372,147409,152831,153090,161742,222008,494470 | ACVR1,ACVR2A,BDNF,BMP2,BMPR1A,BMPR2,LDLRAD4,CAV2,CDKN1C,CREB1,CREBBP,CRKL,CCN2,CTNNB1,DDX5,DLX5,DYNC1LI2,EDN1,EGR1,EP300,ERBB4,FBN2,FER,FGF7,FGF10,FGFR3,FGFR2,FOXC1,GCLC,GCLM,GLG1,GRB10,NR3C1,HIVEP1,ONECUT1,HSPA5,RBPJ,ITGB1,KCNC1,KCNC2,LRP2,LTBP1,SMAD6,MAP1B,MEF2C,MSX1,NCL,NOS1,NTRK3,P2RY1,PDE8A,PDGFRA,PDPK1,POLR2B,PPM1A,PRKD1,PRKCZ,MAPK1,PTBP1,PTEN,PTN,PTPN1,PTPRK,ROBO1,ROCK1,RPS6KB1,SKI,SNAI2,SNCA,SORL1,SOX5,MAP3K7,ZEB1,TGFB2,TGFBR1,TGFBR2,TGIF1,THBS1,TIAL1,NKX2-1,UBB,UBE2D1,UBE2D3,UBE3A,VCAM1,LRP8,SHOC2,USP9X,CASK,URI1,NRP1,USP8,SLC33A1,KLF4,NREP,ZFYVE9,TGFBRAP1,ROCK2,ONECUT2,ADAMTS3,BAG4,RAPGEF2,MAGI2,USP15,TOB1,SPRY1,FST,SLC9A6,FRS2,PPARGC1A,FERMT2,SETX,ZNF423,CYFIP1,SULF1,ARHGEF18,RBFOX2,LEMD3,FLRT3,APPL1,NPTN,PDCD4,DLL1,CTDSPL2,RAB14,NLK,ERRFI1,RNF111,SOX6,PMEPA1,FAM20C,SMURF1,SMOC2,HHIP,SMURF2,NIBAN2,FAT4,SNX25,SYAP1,CCBE1,DSG4,KLB,DAB2IP,SPRED1,VSTM2A,RNF165 |
| 8_Member | GO Biological Processes | GO:0071363 | cellular response to growth factor stimulus | -21.76298109 | -18.844 | 140/709 | 90,92,627,650,657,659,753,858,1028,1385,1387,1399,1490,1499,1655,1749,1783,1906,1958,2033,2066,2201,2252,2255,2261,2263,2296,2729,2730,2734,2887,2908,3096,3175,3309,3516,3688,4036,4052,4091,4131,4208,4487,4691,4842,4916,5151,5156,5170,5431,5494,5587,5590,5594,5725,5728,5764,5770,5796,6091,6198,6497,6591,6622,6653,6660,6885,6935,7042,7046,7048,7050,7057,7073,7080,7314,7321,7323,7337,7412,7804,8036,8239,8573,8725,8829,9101,9197,9314,9315,9372,9392,9480,9508,9530,9693,9863,9958,10140,10252,10468,10479,10818,10891,10979,23064,23090,23191,23213,23370,23543,23592,23767,26060,27020,27250,28514,51496,51552,51701,54206,54778,55553,56937,56975,57154,64094,64399,64750,64855,79633,83891,94056,147372,147409,152831,153090,161742,222008,494470 | ACVR1,ACVR2A,BDNF,BMP2,BMPR1A,BMPR2,LDLRAD4,CAV2,CDKN1C,CREB1,CREBBP,CRKL,CCN2,CTNNB1,DDX5,DLX5,DYNC1LI2,EDN1,EGR1,EP300,ERBB4,FBN2,FGF7,FGF10,FGFR3,FGFR2,FOXC1,GCLC,GCLM,GLG1,GRB10,NR3C1,HIVEP1,ONECUT1,HSPA5,RBPJ,ITGB1,LRP2,LTBP1,SMAD6,MAP1B,MEF2C,MSX1,NCL,NOS1,NTRK3,PDE8A,PDGFRA,PDPK1,POLR2B,PPM1A,PRKD1,PRKCZ,MAPK1,PTBP1,PTEN,PTN,PTPN1,PTPRK,ROBO1,RPS6KB1,SKI,SNAI2,SNCA,SORL1,SOX5,MAP3K7,ZEB1,TGFB2,TGFBR1,TGFBR2,TGIF1,THBS1,TIAL1,NKX2-1,UBB,UBE2D1,UBE2D3,UBE3A,VCAM1,LRP8,SHOC2,USP9X,CASK,URI1,NRP1,USP8,SLC33A1,KLF4,NREP,ZFYVE9,TGFBRAP1,ONECUT2,ADAMTS3,BAG4,RAPGEF2,MAGI2,USP15,TOB1,SPRY1,FST,SLC9A6,FRS2,PPARGC1A,FERMT2,SETX,ZNF423,CYFIP1,SULF1,ARHGEF18,RBFOX2,LEMD3,FLRT3,APPL1,NPTN,PDCD4,DLL1,CTDSPL2,RAB14,NLK,ERRFI1,RNF111,SOX6,PMEPA1,FAM20C,SMURF1,SMOC2,HHIP,SMURF2,NIBAN2,FAT4,SNX25,SYAP1,CCBE1,DSG4,KLB,DAB2IP,SPRED1,VSTM2A,RNF165 |
| 8_Member | GO Biological Processes | GO:0007178 | transmembrane receptor protein serine/threonine kinase signaling pathway | -13.58930774 | -11.343 | 75/353 | 90,92,650,651,657,659,753,858,1028,1385,1387,1655,1749,1958,2033,2201,2255,2734,3096,3175,3309,3516,3624,3625,3688,4012,4036,4052,4091,4487,5170,5494,5590,5796,6497,6653,6711,6885,6935,7042,7046,7048,7057,7080,7314,7321,7323,8239,9197,9315,9372,9392,9480,9863,9958,10140,10468,10979,23090,23213,23370,23592,25937,26060,27250,51496,51701,54778,56937,57154,60436,64750,83891,147409,494470 | ACVR1,ACVR2A,BMP2,BMP3,BMPR1A,BMPR2,LDLRAD4,CAV2,CDKN1C,CREB1,CREBBP,DDX5,DLX5,EGR1,EP300,FBN2,FGF10,GLG1,HIVEP1,ONECUT1,HSPA5,RBPJ,INHBA,INHBB,ITGB1,LNPEP,LRP2,LTBP1,SMAD6,MSX1,PDPK1,PPM1A,PRKCZ,PTPRK,SKI,SORL1,SPTBN1,MAP3K7,ZEB1,TGFB2,TGFBR1,TGFBR2,THBS1,NKX2-1,UBB,UBE2D1,UBE2D3,USP9X,SLC33A1,NREP,ZFYVE9,TGFBRAP1,ONECUT2,MAGI2,USP15,TOB1,FST,FERMT2,ZNF423,SULF1,ARHGEF18,LEMD3,WWTR1,APPL1,PDCD4,CTDSPL2,NLK,RNF111,PMEPA1,SMURF1,TGIF2,SMURF2,SNX25,DSG4,RNF165 |
| 8_Member | GO Biological Processes | GO:0071559 | response to transforming growth factor beta | -10.7014673 | -8.671 | 56/258 | 90,92,657,659,753,858,1028,1385,1387,1399,1906,2033,2201,2263,2734,2908,3175,3309,3688,4052,4091,4208,5170,5494,5590,5796,6093,6497,6660,6885,6935,7042,7046,7048,7057,7080,7314,8239,9315,9372,9392,9475,9480,9958,10891,10979,23370,23592,26060,51701,54778,55553,56937,57154,64750,83891 | ACVR1,ACVR2A,BMPR1A,BMPR2,LDLRAD4,CAV2,CDKN1C,CREB1,CREBBP,CRKL,EDN1,EP300,FBN2,FGFR2,GLG1,NR3C1,ONECUT1,HSPA5,ITGB1,LTBP1,SMAD6,MEF2C,PDPK1,PPM1A,PRKCZ,PTPRK,ROCK1,SKI,SOX5,MAP3K7,ZEB1,TGFB2,TGFBR1,TGFBR2,THBS1,NKX2-1,UBB,USP9X,NREP,ZFYVE9,TGFBRAP1,ROCK2,ONECUT2,USP15,PPARGC1A,FERMT2,ARHGEF18,LEMD3,APPL1,NLK,RNF111,SOX6,PMEPA1,SMURF1,SMURF2,SNX25 |
| 8_Member | GO Biological Processes | GO:0071560 | cellular response to transforming growth factor beta stimulus | -10.12157968 | -8.148 | 54/252 | 90,92,657,659,753,858,1028,1385,1387,1399,1906,2033,2201,2263,2734,2908,3175,3309,3688,4052,4091,4208,5170,5494,5590,5796,6497,6660,6885,6935,7042,7046,7048,7057,7080,7314,8239,9315,9372,9392,9480,9958,10891,10979,23370,23592,26060,51701,54778,55553,56937,57154,64750,83891 | ACVR1,ACVR2A,BMPR1A,BMPR2,LDLRAD4,CAV2,CDKN1C,CREB1,CREBBP,CRKL,EDN1,EP300,FBN2,FGFR2,GLG1,NR3C1,ONECUT1,HSPA5,ITGB1,LTBP1,SMAD6,MEF2C,PDPK1,PPM1A,PRKCZ,PTPRK,SKI,SOX5,MAP3K7,ZEB1,TGFB2,TGFBR1,TGFBR2,THBS1,NKX2-1,UBB,USP9X,NREP,ZFYVE9,TGFBRAP1,ONECUT2,USP15,PPARGC1A,FERMT2,ARHGEF18,LEMD3,APPL1,NLK,RNF111,SOX6,PMEPA1,SMURF1,SMURF2,SNX25 |
| 8_Member | GO Molecular Functions | GO:0046332 | SMAD binding | -9.953784224 | -7.992 | 27/80 | 90,92,650,657,659,753,1290,1499,1655,4091,5080,5494,6497,7046,7048,7050,7528,8239,9392,9839,9863,9958,10140,54778,56937,57154,64750 | ACVR1,ACVR2A,BMP2,BMPR1A,BMPR2,LDLRAD4,COL5A2,CTNNB1,DDX5,SMAD6,PAX6,PPM1A,SKI,TGFBR1,TGFBR2,TGIF1,YY1,USP9X,TGFBRAP1,ZEB2,MAGI2,USP15,TOB1,RNF111,PMEPA1,SMURF1,SMURF2 |
| 8_Member | GO Biological Processes | GO:0007179 | transforming growth factor beta receptor signaling pathway | -9.640577878 | -7.700 | 46/202 | 90,92,657,659,753,858,1028,1385,1387,2033,2201,2734,3175,3309,3688,4052,4091,5170,5494,5590,5796,6497,6885,6935,7042,7046,7048,7057,7080,7314,8239,9315,9372,9392,9480,9958,10979,23370,23592,26060,51701,54778,56937,57154,64750,83891 | ACVR1,ACVR2A,BMPR1A,BMPR2,LDLRAD4,CAV2,CDKN1C,CREB1,CREBBP,EP300,FBN2,GLG1,ONECUT1,HSPA5,ITGB1,LTBP1,SMAD6,PDPK1,PPM1A,PRKCZ,PTPRK,SKI,MAP3K7,ZEB1,TGFB2,TGFBR1,TGFBR2,THBS1,NKX2-1,UBB,USP9X,NREP,ZFYVE9,TGFBRAP1,ONECUT2,USP15,FERMT2,ARHGEF18,LEMD3,APPL1,NLK,RNF111,PMEPA1,SMURF1,SMURF2,SNX25 |
| 8_Member | GO Biological Processes | GO:0090287 | regulation of cellular response to growth factor stimulus | -9.3917347 | -7.473 | 58/293 | 659,753,858,1028,1387,1499,2033,2201,2255,2734,2887,3175,3309,3516,4036,4052,4091,4487,4916,5170,5494,5770,6091,6497,6653,6935,7046,7048,7057,7080,7314,8573,9315,9480,9508,10140,10252,10468,10479,23090,23191,23213,23592,27020,28514,51496,54778,56937,56975,57154,64094,64399,64750,64855,83891,147372,153090,494470 | BMPR2,LDLRAD4,CAV2,CDKN1C,CREBBP,CTNNB1,EP300,FBN2,FGF10,GLG1,GRB10,ONECUT1,HSPA5,RBPJ,LRP2,LTBP1,SMAD6,MSX1,NTRK3,PDPK1,PPM1A,PTPN1,ROBO1,SKI,SORL1,ZEB1,TGFBR1,TGFBR2,THBS1,NKX2-1,UBB,CASK,NREP,ONECUT2,ADAMTS3,TOB1,SPRY1,FST,SLC9A6,ZNF423,CYFIP1,SULF1,LEMD3,NPTN,DLL1,CTDSPL2,RNF111,PMEPA1,FAM20C,SMURF1,SMOC2,HHIP,SMURF2,NIBAN2,SNX25,CCBE1,DAB2IP,RNF165 |
| 8_Member | GO Biological Processes | GO:0090092 | regulation of transmembrane receptor protein serine/threonine kinase signaling pathway | -9.261740346 | -7.357 | 51/243 | 90,92,650,651,657,659,753,858,1028,1387,2033,2201,2255,2734,3175,3309,3516,3624,3625,4036,4052,4091,4487,5170,5494,6497,6653,6711,6935,7042,7046,7048,7057,7080,7314,9315,9480,9863,10140,10468,23090,23213,23592,25937,51496,54778,56937,57154,64750,83891,494470 | ACVR1,ACVR2A,BMP2,BMP3,BMPR1A,BMPR2,LDLRAD4,CAV2,CDKN1C,CREBBP,EP300,FBN2,FGF10,GLG1,ONECUT1,HSPA5,RBPJ,INHBA,INHBB,LRP2,LTBP1,SMAD6,MSX1,PDPK1,PPM1A,SKI,SORL1,SPTBN1,ZEB1,TGFB2,TGFBR1,TGFBR2,THBS1,NKX2-1,UBB,NREP,ONECUT2,MAGI2,TOB1,FST,ZNF423,SULF1,LEMD3,WWTR1,CTDSPL2,RNF111,PMEPA1,SMURF1,SMURF2,SNX25,RNF165 |
| 8_Member | GO Biological Processes | GO:0017015 | regulation of transforming growth factor beta receptor signaling pathway | -6.29097405 | -4.661 | 28/121 | 753,858,1028,1387,2033,2201,2734,3175,3309,4052,4091,5170,5494,6497,6935,7046,7048,7057,7080,7314,9315,9480,23592,54778,56937,57154,64750,83891 | LDLRAD4,CAV2,CDKN1C,CREBBP,EP300,FBN2,GLG1,ONECUT1,HSPA5,LTBP1,SMAD6,PDPK1,PPM1A,SKI,ZEB1,TGFBR1,TGFBR2,THBS1,NKX2-1,UBB,NREP,ONECUT2,LEMD3,RNF111,PMEPA1,SMURF1,SMURF2,SNX25 |
| 8_Member | GO Biological Processes | GO:1903844 | regulation of cellular response to transforming growth factor beta stimulus | -6.136516539 | -4.525 | 28/123 | 753,858,1028,1387,2033,2201,2734,3175,3309,4052,4091,5170,5494,6497,6935,7046,7048,7057,7080,7314,9315,9480,23592,54778,56937,57154,64750,83891 | LDLRAD4,CAV2,CDKN1C,CREBBP,EP300,FBN2,GLG1,ONECUT1,HSPA5,LTBP1,SMAD6,PDPK1,PPM1A,SKI,ZEB1,TGFBR1,TGFBR2,THBS1,NKX2-1,UBB,NREP,ONECUT2,LEMD3,RNF111,PMEPA1,SMURF1,SMURF2,SNX25 |
| 8_Member | GO Biological Processes | GO:0090101 | negative regulation of transmembrane receptor protein serine/threonine kinase signaling pathway | -5.912477607 | -4.330 | 28/126 | 90,753,858,2201,2734,3175,3309,4036,4052,4091,5170,5494,6497,6653,7046,7048,7080,7314,9480,9863,10140,10468,23592,51496,56937,57154,64750,83891 | ACVR1,LDLRAD4,CAV2,FBN2,GLG1,ONECUT1,HSPA5,LRP2,LTBP1,SMAD6,PDPK1,PPM1A,SKI,SORL1,TGFBR1,TGFBR2,NKX2-1,UBB,ONECUT2,MAGI2,TOB1,FST,LEMD3,CTDSPL2,PMEPA1,SMURF1,SMURF2,SNX25 |
| 8_Member | GO Biological Processes | GO:0030509 | BMP signaling pathway | -5.77731978 | -4.218 | 32/157 | 90,92,650,657,659,1655,1749,1958,3096,3516,4036,4091,4487,5494,6497,6653,7321,7323,8239,9197,9958,10140,10468,23090,23213,23592,27250,51496,57154,64750,147409,494470 | ACVR1,ACVR2A,BMP2,BMPR1A,BMPR2,DDX5,DLX5,EGR1,HIVEP1,RBPJ,LRP2,SMAD6,MSX1,PPM1A,SKI,SORL1,UBE2D1,UBE2D3,USP9X,SLC33A1,USP15,TOB1,FST,ZNF423,SULF1,LEMD3,PDCD4,CTDSPL2,SMURF1,SMURF2,DSG4,RNF165 |
| 8_Member | GO Biological Processes | GO:0030512 | negative regulation of transforming growth factor beta receptor signaling pathway | -5.721173779 | -4.171 | 21/81 | 753,858,2201,2734,3175,3309,4052,4091,5170,5494,6497,7046,7048,7080,7314,9480,23592,56937,57154,64750,83891 | LDLRAD4,CAV2,FBN2,GLG1,ONECUT1,HSPA5,LTBP1,SMAD6,PDPK1,PPM1A,SKI,TGFBR1,TGFBR2,NKX2-1,UBB,ONECUT2,LEMD3,PMEPA1,SMURF1,SMURF2,SNX25 |
| 8_Member | GO Biological Processes | GO:1903845 | negative regulation of cellular response to transforming growth factor beta stimulus | -5.536211647 | -4.011 | 21/83 | 753,858,2201,2734,3175,3309,4052,4091,5170,5494,6497,7046,7048,7080,7314,9480,23592,56937,57154,64750,83891 | LDLRAD4,CAV2,FBN2,GLG1,ONECUT1,HSPA5,LTBP1,SMAD6,PDPK1,PPM1A,SKI,TGFBR1,TGFBR2,NKX2-1,UBB,ONECUT2,LEMD3,PMEPA1,SMURF1,SMURF2,SNX25 |
| 8_Member | GO Biological Processes | GO:0071773 | cellular response to BMP stimulus | -5.447839977 | -3.931 | 33/170 | 90,92,650,657,659,1655,1749,1958,3096,3516,4036,4091,4487,5494,6497,6653,7321,7323,8239,9197,9958,10140,10468,23090,23213,23592,27250,51496,57154,64750,147409,222008,494470 | ACVR1,ACVR2A,BMP2,BMPR1A,BMPR2,DDX5,DLX5,EGR1,HIVEP1,RBPJ,LRP2,SMAD6,MSX1,PPM1A,SKI,SORL1,UBE2D1,UBE2D3,USP9X,SLC33A1,USP15,TOB1,FST,ZNF423,SULF1,LEMD3,PDCD4,CTDSPL2,SMURF1,SMURF2,DSG4,VSTM2A,RNF165 |
| 8_Member | GO Biological Processes | GO:0071772 | response to BMP | -5.447839977 | -3.931 | 33/170 | 90,92,650,657,659,1655,1749,1958,3096,3516,4036,4091,4487,5494,6497,6653,7321,7323,8239,9197,9958,10140,10468,23090,23213,23592,27250,51496,57154,64750,147409,222008,494470 | ACVR1,ACVR2A,BMP2,BMPR1A,BMPR2,DDX5,DLX5,EGR1,HIVEP1,RBPJ,LRP2,SMAD6,MSX1,PPM1A,SKI,SORL1,UBE2D1,UBE2D3,USP9X,SLC33A1,USP15,TOB1,FST,ZNF423,SULF1,LEMD3,PDCD4,CTDSPL2,SMURF1,SMURF2,DSG4,VSTM2A,RNF165 |
| 8_Member | GO Biological Processes | GO:0090288 | negative regulation of cellular response to growth factor stimulus | -5.236831269 | -3.746 | 32/166 | 753,858,2201,2734,3175,3309,4036,4052,4091,5170,5494,5770,6497,6653,7046,7048,7057,7080,7314,8573,9480,10140,10252,23213,23592,51496,56937,57154,64750,64855,83891,153090 | LDLRAD4,CAV2,FBN2,GLG1,ONECUT1,HSPA5,LRP2,LTBP1,SMAD6,PDPK1,PPM1A,PTPN1,SKI,SORL1,TGFBR1,TGFBR2,THBS1,NKX2-1,UBB,CASK,ONECUT2,TOB1,SPRY1,SULF1,LEMD3,CTDSPL2,PMEPA1,SMURF1,SMURF2,NIBAN2,SNX25,DAB2IP |
| 8_Member | GO Biological Processes | GO:0030510 | regulation of BMP signaling pathway | -2.913819214 | -1.778 | 17/91 | 659,3516,4036,4091,4487,5494,6497,6653,10140,10468,23090,23213,23592,51496,57154,64750,494470 | BMPR2,RBPJ,LRP2,SMAD6,MSX1,PPM1A,SKI,SORL1,TOB1,FST,ZNF423,SULF1,LEMD3,CTDSPL2,SMURF1,SMURF2,RNF165 |
| 9_Summary | GO Biological Processes | GO:0007423 | sensory organ development | -22.59691959 | -19.627 | 120/548 | 102,357,596,650,659,775,780,793,1027,1028,1290,1301,1499,1641,1739,1749,1750,1826,1906,2043,2048,2138,2195,2201,2255,2263,2295,2296,2487,2624,2736,2737,2782,2823,3150,3516,3624,3638,3714,3784,3815,3985,4040,4094,4211,4286,4487,4628,4760,4916,4919,5048,5080,5156,5308,5594,5629,5764,5797,5915,6096,6299,6497,6670,6935,7020,7042,7046,7048,7068,7111,7490,7528,7855,8013,8289,8549,8829,8854,9314,9355,9474,9497,9547,9839,10194,10265,10804,10818,22931,23047,26018,26146,28514,51141,51804,54806,55084,55636,55714,57381,60436,64093,64919,79633,81029,84189,84678,85407,93986,114789,121227,125488,130497,145258,157506,170692,204851,221935,285590,10371 | ADAM10,SHROOM2,BCL2,BMP2,BMPR2,CACNA1C,DDR1,CALB1,CDKN1B,CDKN1C,COL5A2,COL11A1,CTNNB1,DCX,DLG1,DLX5,DLX6,DSCAM,EDN1,EPHA4,EPHB2,EYA1,FAT1,FBN2,FGF10,FGFR2,FOXF2,FOXC1,FRZB,GATA2,GLI2,GLI3,GNB1,GPM6A,HMGN1,RBPJ,INHBA,INSIG1,JAG2,KCNQ1,KIT,LIMK2,LRP6,MAF,MEIS1,MITF,MSX1,MYH10,NEUROD1,NTRK3,ROR1,PAFAH1B1,PAX6,PDGFRA,PITX2,MAPK1,PROX1,PTN,PTPRM,RARB,RORB,SALL1,SKI,SP3,ZEB1,TFAP2A,TGFB2,TGFBR1,TGFBR2,THRB,TMOD1,WT1,YY1,FZD5,NR4A3,ARID1A,LGR5,NRP1,ALDH1A2,KLF4,LHX2,ATG5,SLC4A7,CXCL14,ZEB2,TSHZ1,IRX5,GJB6,FRS2,RAB18,PDS5B,LRIG1,TRAF3IP1,DLL1,INSIG2,SIX4,AHI1,SOBP,CHD7,TENM3,RHOJ,TGIF2,SMOC1,BCL11B,FAT4,WNT5B,SLITRK6,KDM2B,NKD1,FOXP2,SLC25A25,LRIG3,TTC39C,OSR1,GSC,RDH10,ADAMTS18,HIPK1,SDK1,SH3PXD2B,SEMA3A |
| 9_Member | GO Biological Processes | GO:0007423 | sensory organ development | -22.59691959 | -19.627 | 120/548 | 102,357,596,650,659,775,780,793,1027,1028,1290,1301,1499,1641,1739,1749,1750,1826,1906,2043,2048,2138,2195,2201,2255,2263,2295,2296,2487,2624,2736,2737,2782,2823,3150,3516,3624,3638,3714,3784,3815,3985,4040,4094,4211,4286,4487,4628,4760,4916,4919,5048,5080,5156,5308,5594,5629,5764,5797,5915,6096,6299,6497,6670,6935,7020,7042,7046,7048,7068,7111,7490,7528,7855,8013,8289,8549,8829,8854,9314,9355,9474,9497,9547,9839,10194,10265,10804,10818,22931,23047,26018,26146,28514,51141,51804,54806,55084,55636,55714,57381,60436,64093,64919,79633,81029,84189,84678,85407,93986,114789,121227,125488,130497,145258,157506,170692,204851,221935,285590 | ADAM10,SHROOM2,BCL2,BMP2,BMPR2,CACNA1C,DDR1,CALB1,CDKN1B,CDKN1C,COL5A2,COL11A1,CTNNB1,DCX,DLG1,DLX5,DLX6,DSCAM,EDN1,EPHA4,EPHB2,EYA1,FAT1,FBN2,FGF10,FGFR2,FOXF2,FOXC1,FRZB,GATA2,GLI2,GLI3,GNB1,GPM6A,HMGN1,RBPJ,INHBA,INSIG1,JAG2,KCNQ1,KIT,LIMK2,LRP6,MAF,MEIS1,MITF,MSX1,MYH10,NEUROD1,NTRK3,ROR1,PAFAH1B1,PAX6,PDGFRA,PITX2,MAPK1,PROX1,PTN,PTPRM,RARB,RORB,SALL1,SKI,SP3,ZEB1,TFAP2A,TGFB2,TGFBR1,TGFBR2,THRB,TMOD1,WT1,YY1,FZD5,NR4A3,ARID1A,LGR5,NRP1,ALDH1A2,KLF4,LHX2,ATG5,SLC4A7,CXCL14,ZEB2,TSHZ1,IRX5,GJB6,FRS2,RAB18,PDS5B,LRIG1,TRAF3IP1,DLL1,INSIG2,SIX4,AHI1,SOBP,CHD7,TENM3,RHOJ,TGIF2,SMOC1,BCL11B,FAT4,WNT5B,SLITRK6,KDM2B,NKD1,FOXP2,SLC25A25,LRIG3,TTC39C,OSR1,GSC,RDH10,ADAMTS18,HIPK1,SDK1,SH3PXD2B |
| 9_Member | GO Biological Processes | GO:0001654 | eye development | -15.98562264 | -13.544 | 81/362 | 357,596,659,775,793,1027,1028,1290,1499,1641,1739,1826,2048,2195,2201,2255,2295,2296,2737,2782,2823,3150,3624,3985,4040,4094,4211,4286,4628,4760,4916,5080,5156,5308,5629,5764,5797,5915,6096,6497,6670,6935,7020,7042,7046,7048,7068,7111,7490,7528,7855,8289,8829,8854,9314,9355,9839,10265,10818,22931,23047,26146,28514,54806,55636,55714,57381,60436,64093,64919,81029,84189,84678,85407,93986,114789,157506,170692,204851,221935,285590 | SHROOM2,BCL2,BMPR2,CACNA1C,CALB1,CDKN1B,CDKN1C,COL5A2,CTNNB1,DCX,DLG1,DSCAM,EPHB2,FAT1,FBN2,FGF10,FOXF2,FOXC1,GLI3,GNB1,GPM6A,HMGN1,INHBA,LIMK2,LRP6,MAF,MEIS1,MITF,MYH10,NEUROD1,NTRK3,PAX6,PDGFRA,PITX2,PROX1,PTN,PTPRM,RARB,RORB,SKI,SP3,ZEB1,TFAP2A,TGFB2,TGFBR1,TGFBR2,THRB,TMOD1,WT1,YY1,FZD5,ARID1A,NRP1,ALDH1A2,KLF4,LHX2,ZEB2,IRX5,FRS2,RAB18,PDS5B,TRAF3IP1,DLL1,AHI1,CHD7,TENM3,RHOJ,TGIF2,SMOC1,BCL11B,WNT5B,SLITRK6,KDM2B,NKD1,FOXP2,SLC25A25,RDH10,ADAMTS18,HIPK1,SDK1,SH3PXD2B |
| 9_Member | GO Biological Processes | GO:0048880 | sensory system development | -15.84435562 | -13.413 | 82/371 | 357,596,659,775,793,1027,1028,1290,1499,1641,1739,1826,2048,2195,2201,2255,2295,2296,2737,2782,2823,3150,3624,3985,4040,4094,4211,4286,4628,4760,4916,5080,5156,5308,5629,5764,5797,5915,6096,6497,6670,6935,7020,7042,7046,7048,7068,7111,7490,7528,7855,8289,8829,8854,9314,9355,9839,10265,10371,10818,22931,23047,26146,28514,54806,55636,55714,57381,60436,64093,64919,81029,84189,84678,85407,93986,114789,157506,170692,204851,221935,285590 | SHROOM2,BCL2,BMPR2,CACNA1C,CALB1,CDKN1B,CDKN1C,COL5A2,CTNNB1,DCX,DLG1,DSCAM,EPHB2,FAT1,FBN2,FGF10,FOXF2,FOXC1,GLI3,GNB1,GPM6A,HMGN1,INHBA,LIMK2,LRP6,MAF,MEIS1,MITF,MYH10,NEUROD1,NTRK3,PAX6,PDGFRA,PITX2,PROX1,PTN,PTPRM,RARB,RORB,SKI,SP3,ZEB1,TFAP2A,TGFB2,TGFBR1,TGFBR2,THRB,TMOD1,WT1,YY1,FZD5,ARID1A,NRP1,ALDH1A2,KLF4,LHX2,ZEB2,IRX5,SEMA3A,FRS2,RAB18,PDS5B,TRAF3IP1,DLL1,AHI1,CHD7,TENM3,RHOJ,TGIF2,SMOC1,BCL11B,WNT5B,SLITRK6,KDM2B,NKD1,FOXP2,SLC25A25,RDH10,ADAMTS18,HIPK1,SDK1,SH3PXD2B |
| 9_Member | GO Biological Processes | GO:0090596 | sensory organ morphogenesis | -15.78132983 | -13.355 | 65/256 | 357,596,793,1290,1301,1499,1749,1750,1826,1906,2048,2138,2195,2201,2255,2263,2295,2487,2624,2736,2737,3150,3638,4040,4211,4487,5080,5308,5594,5629,5764,5797,5915,6096,6299,6497,6670,6935,7020,7068,7528,7855,8013,8289,10194,10265,10804,10818,26018,28514,51141,51804,54806,55084,55636,55714,84189,84678,85407,121227,125488,130497,145258,204851,221935 | SHROOM2,BCL2,CALB1,COL5A2,COL11A1,CTNNB1,DLX5,DLX6,DSCAM,EDN1,EPHB2,EYA1,FAT1,FBN2,FGF10,FGFR2,FOXF2,FRZB,GATA2,GLI2,GLI3,HMGN1,INSIG1,LRP6,MEIS1,MSX1,PAX6,PITX2,MAPK1,PROX1,PTN,PTPRM,RARB,RORB,SALL1,SKI,SP3,ZEB1,TFAP2A,THRB,YY1,FZD5,NR4A3,ARID1A,TSHZ1,IRX5,GJB6,FRS2,LRIG1,DLL1,INSIG2,SIX4,AHI1,SOBP,CHD7,TENM3,SLITRK6,KDM2B,NKD1,LRIG3,TTC39C,OSR1,GSC,HIPK1,SDK1 |
| 9_Member | GO Biological Processes | GO:0150063 | visual system development | -15.69294434 | -13.277 | 81/366 | 357,596,659,775,793,1027,1028,1290,1499,1641,1739,1826,2048,2195,2201,2255,2295,2296,2737,2782,2823,3150,3624,3985,4040,4094,4211,4286,4628,4760,4916,5080,5156,5308,5629,5764,5797,5915,6096,6497,6670,6935,7020,7042,7046,7048,7068,7111,7490,7528,7855,8289,8829,8854,9314,9355,9839,10265,10818,22931,23047,26146,28514,54806,55636,55714,57381,60436,64093,64919,81029,84189,84678,85407,93986,114789,157506,170692,204851,221935,285590 | SHROOM2,BCL2,BMPR2,CACNA1C,CALB1,CDKN1B,CDKN1C,COL5A2,CTNNB1,DCX,DLG1,DSCAM,EPHB2,FAT1,FBN2,FGF10,FOXF2,FOXC1,GLI3,GNB1,GPM6A,HMGN1,INHBA,LIMK2,LRP6,MAF,MEIS1,MITF,MYH10,NEUROD1,NTRK3,PAX6,PDGFRA,PITX2,PROX1,PTN,PTPRM,RARB,RORB,SKI,SP3,ZEB1,TFAP2A,TGFB2,TGFBR1,TGFBR2,THRB,TMOD1,WT1,YY1,FZD5,ARID1A,NRP1,ALDH1A2,KLF4,LHX2,ZEB2,IRX5,FRS2,RAB18,PDS5B,TRAF3IP1,DLL1,AHI1,CHD7,TENM3,RHOJ,TGIF2,SMOC1,BCL11B,WNT5B,SLITRK6,KDM2B,NKD1,FOXP2,SLC25A25,RDH10,ADAMTS18,HIPK1,SDK1,SH3PXD2B |
| 9_Member | GO Biological Processes | GO:0043010 | camera-type eye development | -15.40209826 | -13.001 | 73/314 | 357,659,775,793,1027,1028,1499,1641,1739,1826,2048,2195,2201,2255,2295,2296,2737,2782,2823,3150,3624,3985,4040,4094,4211,4286,4628,4760,4916,5080,5156,5308,5629,5764,5797,6096,6497,6670,6935,7020,7042,7046,7048,7068,7111,7490,7528,7855,8289,8829,8854,9314,9355,9839,10265,10818,23047,26146,28514,54806,55636,55714,57381,60436,64919,81029,84189,84678,93986,114789,157506,204851,221935 | SHROOM2,BMPR2,CACNA1C,CALB1,CDKN1B,CDKN1C,CTNNB1,DCX,DLG1,DSCAM,EPHB2,FAT1,FBN2,FGF10,FOXF2,FOXC1,GLI3,GNB1,GPM6A,HMGN1,INHBA,LIMK2,LRP6,MAF,MEIS1,MITF,MYH10,NEUROD1,NTRK3,PAX6,PDGFRA,PITX2,PROX1,PTN,PTPRM,RORB,SKI,SP3,ZEB1,TFAP2A,TGFB2,TGFBR1,TGFBR2,THRB,TMOD1,WT1,YY1,FZD5,ARID1A,NRP1,ALDH1A2,KLF4,LHX2,ZEB2,IRX5,FRS2,PDS5B,TRAF3IP1,DLL1,AHI1,CHD7,TENM3,RHOJ,TGIF2,BCL11B,WNT5B,SLITRK6,KDM2B,FOXP2,SLC25A25,RDH10,HIPK1,SDK1 |
| 9_Member | GO Biological Processes | GO:0048593 | camera-type eye morphogenesis | -10.19531242 | -8.214 | 33/112 | 357,793,1499,1826,2048,2195,2295,2737,3150,4040,4211,5080,5308,5629,5764,5797,6096,6497,6670,6935,7020,7068,7528,7855,8289,10265,10818,28514,54806,55714,84678,204851,221935 | SHROOM2,CALB1,CTNNB1,DSCAM,EPHB2,FAT1,FOXF2,GLI3,HMGN1,LRP6,MEIS1,PAX6,PITX2,PROX1,PTN,PTPRM,RORB,SKI,SP3,ZEB1,TFAP2A,THRB,YY1,FZD5,ARID1A,IRX5,FRS2,DLL1,AHI1,TENM3,KDM2B,HIPK1,SDK1 |
| 9_Member | GO Biological Processes | GO:0048592 | eye morphogenesis | -9.577839523 | -7.644 | 38/149 | 357,596,793,1290,1499,1826,2048,2195,2201,2295,2737,3150,4040,4211,5080,5308,5629,5764,5797,5915,6096,6497,6670,6935,7020,7068,7528,7855,8289,10265,10818,28514,54806,55714,84678,85407,204851,221935 | SHROOM2,BCL2,CALB1,COL5A2,CTNNB1,DSCAM,EPHB2,FAT1,FBN2,FOXF2,GLI3,HMGN1,LRP6,MEIS1,PAX6,PITX2,PROX1,PTN,PTPRM,RARB,RORB,SKI,SP3,ZEB1,TFAP2A,THRB,YY1,FZD5,ARID1A,IRX5,FRS2,DLL1,AHI1,TENM3,KDM2B,NKD1,HIPK1,SDK1 |
| 9_Member | GO Biological Processes | GO:0060041 | retina development in camera-type eye | -5.191076824 | -3.706 | 29/144 | 659,793,1641,1826,2782,2823,4040,4628,4760,5080,5156,5629,5764,5797,6096,6497,7020,7042,7068,8829,9355,10265,28514,54806,55636,57381,60436,204851,221935 | BMPR2,CALB1,DCX,DSCAM,GNB1,GPM6A,LRP6,MYH10,NEUROD1,PAX6,PDGFRA,PROX1,PTN,PTPRM,RORB,SKI,TFAP2A,TGFB2,THRB,NRP1,LHX2,IRX5,DLL1,AHI1,CHD7,RHOJ,TGIF2,HIPK1,SDK1 |
| 9_Member | GO Biological Processes | GO:0060042 | retina morphogenesis in camera-type eye | -4.528186333 | -3.127 | 14/49 | 793,1826,4040,5629,5764,5797,6096,7020,7068,10265,28514,54806,204851,221935 | CALB1,DSCAM,LRP6,PROX1,PTN,PTPRM,RORB,TFAP2A,THRB,IRX5,DLL1,AHI1,HIPK1,SDK1 |
| 9_Member | GO Biological Processes | GO:0003407 | neural retina development | -3.338509479 | -2.122 | 14/62 | 793,1826,2823,4760,5764,5797,6096,7020,7042,7068,10265,54806,204851,221935 | CALB1,DSCAM,GPM6A,NEUROD1,PTN,PTPRM,RORB,TFAP2A,TGFB2,THRB,IRX5,AHI1,HIPK1,SDK1 |
| 9_Member | GO Biological Processes | GO:0010842 | retina layer formation | -2.822345478 | -1.706 | 7/22 | 793,1826,5797,7020,54806,204851,221935 | CALB1,DSCAM,PTPRM,TFAP2A,AHI1,HIPK1,SDK1 |
| 10_Summary | GO Biological Processes | GO:0048729 | tissue morphogenesis | -22.51666819 | -19.564 | 137/674 | 59,90,133,367,596,650,657,659,780,998,1173,1213,1293,1301,1303,1499,1739,1741,1832,1906,2043,2045,2138,2239,2252,2255,2263,2294,2295,2296,2487,2668,2736,2737,3084,3202,3217,3239,3516,3624,3688,3714,3845,4036,4040,4208,4233,4300,4487,4604,4638,4919,5048,5361,5567,5573,5613,5629,5711,5727,5728,5879,5898,5911,6091,6092,6299,6497,6591,6781,7020,7042,7046,7048,7080,7403,7414,7430,7490,7855,8013,8289,8324,8543,8549,8829,8854,9231,9314,9355,9723,9788,9839,9863,9948,10082,10252,10371,10395,10512,10672,10818,10890,10959,11019,11235,23002,23122,23198,23213,23414,23767,26146,28514,29072,51804,54806,55636,55727,55966,57154,57534,64398,64399,64750,64856,65009,79633,80000,81839,84133,84678,85407,94134,130497,157506,166336,793,1027,1028,1399,1958,2018,2048,2049,2624,3655,4091,4643,5125,5156,5915,6492,7010,7337,9510,25937,28999,84159 | ACTA2,ACVR1,ADM,AR,BCL2,BMP2,BMPR1A,BMPR2,DDR1,CDC42,AP2M1,CLTC,COL6A3,COL11A1,COL12A1,CTNNB1,DLG1,DLG3,DSP,EDN1,EPHA4,EPHA7,EYA1,GPC4,FGF7,FGF10,FGFR2,FOXF1,FOXF2,FOXC1,FRZB,GDNF,GLI2,GLI3,NRG1,HOXA5,HOXB7,HOXD13,RBPJ,INHBA,ITGB1,JAG2,KRAS,LRP2,LRP6,MEF2C,MET,MLLT3,MSX1,MYBPC1,MYLK,ROR1,PAFAH1B1,PLXNA1,PRKACB,PRKAR1A,PRKX,PROX1,PSMD5,PTCH1,PTEN,RAC1,RALA,RAP2A,ROBO1,ROBO2,SALL1,SKI,SNAI2,STC1,TFAP2A,TGFB2,TGFBR1,TGFBR2,NKX2-1,KDM6A,VCL,EZR,WT1,FZD5,NR4A3,ARID1A,FZD7,LMO4,LGR5,NRP1,ALDH1A2,DLG5,KLF4,LHX2,SEMA3E,MTSS1,ZEB2,MAGI2,WDR1,GPC6,SPRY1,SEMA3A,DLC1,SEMA3C,GNA13,FRS2,RAB10,TMED2,LIAS,PDCD10,DAAM1,CLASP2,PSME4,SULF1,ZFPM2,FLRT3,TRAF3IP1,DLL1,SETD2,SIX4,AHI1,CHD7,BTBD7,AJAP1,SMURF1,MIB1,MPP5,HHIP,SMURF2,VWA1,NDRG4,FAT4,GREB1L,VANGL1,ZNRF3,KDM2B,NKD1,ARHGAP12,OSR1,RDH10,PRICKLE2,CALB1,CDKN1B,CDKN1C,CRKL,EGR1,EMX2,EPHB2,EPHB3,GATA2,ITGA6,SMAD6,MYO1E,PCSK5,PDGFRA,RARB,SIM1,TEK,UBE3A,ADAMTS1,WWTR1,KLF15,ARID5B |
| 10_Member | GO Biological Processes | GO:0048729 | tissue morphogenesis | -22.51666819 | -19.564 | 137/674 | 59,90,133,367,596,650,657,659,780,998,1173,1213,1293,1301,1303,1499,1739,1741,1832,1906,2043,2045,2138,2239,2252,2255,2263,2294,2295,2296,2487,2668,2736,2737,3084,3202,3217,3239,3516,3624,3688,3714,3845,4036,4040,4208,4233,4300,4487,4604,4638,4919,5048,5361,5567,5573,5613,5629,5711,5727,5728,5879,5898,5911,6091,6092,6299,6497,6591,6781,7020,7042,7046,7048,7080,7403,7414,7430,7490,7855,8013,8289,8324,8543,8549,8829,8854,9231,9314,9355,9723,9788,9839,9863,9948,10082,10252,10371,10395,10512,10672,10818,10890,10959,11019,11235,23002,23122,23198,23213,23414,23767,26146,28514,29072,51804,54806,55636,55727,55966,57154,57534,64398,64399,64750,64856,65009,79633,80000,81839,84133,84678,85407,94134,130497,157506,166336 | ACTA2,ACVR1,ADM,AR,BCL2,BMP2,BMPR1A,BMPR2,DDR1,CDC42,AP2M1,CLTC,COL6A3,COL11A1,COL12A1,CTNNB1,DLG1,DLG3,DSP,EDN1,EPHA4,EPHA7,EYA1,GPC4,FGF7,FGF10,FGFR2,FOXF1,FOXF2,FOXC1,FRZB,GDNF,GLI2,GLI3,NRG1,HOXA5,HOXB7,HOXD13,RBPJ,INHBA,ITGB1,JAG2,KRAS,LRP2,LRP6,MEF2C,MET,MLLT3,MSX1,MYBPC1,MYLK,ROR1,PAFAH1B1,PLXNA1,PRKACB,PRKAR1A,PRKX,PROX1,PSMD5,PTCH1,PTEN,RAC1,RALA,RAP2A,ROBO1,ROBO2,SALL1,SKI,SNAI2,STC1,TFAP2A,TGFB2,TGFBR1,TGFBR2,NKX2-1,KDM6A,VCL,EZR,WT1,FZD5,NR4A3,ARID1A,FZD7,LMO4,LGR5,NRP1,ALDH1A2,DLG5,KLF4,LHX2,SEMA3E,MTSS1,ZEB2,MAGI2,WDR1,GPC6,SPRY1,SEMA3A,DLC1,SEMA3C,GNA13,FRS2,RAB10,TMED2,LIAS,PDCD10,DAAM1,CLASP2,PSME4,SULF1,ZFPM2,FLRT3,TRAF3IP1,DLL1,SETD2,SIX4,AHI1,CHD7,BTBD7,AJAP1,SMURF1,MIB1,MPP5,HHIP,SMURF2,VWA1,NDRG4,FAT4,GREB1L,VANGL1,ZNRF3,KDM2B,NKD1,ARHGAP12,OSR1,RDH10,PRICKLE2 |
| 10_Member | GO Biological Processes | GO:0002009 | morphogenesis of an epithelium | -20.14031111 | -17.406 | 114/540 | 90,133,367,596,650,780,998,1173,1213,1499,1739,1741,1906,2043,2045,2138,2239,2252,2255,2263,2294,2295,2487,2668,2736,2737,3202,3217,3239,3516,3714,3845,4036,4040,4208,4233,4300,4919,5048,5361,5567,5613,5629,5711,5727,5728,5879,5898,5911,6299,6497,6591,7020,7042,7048,7080,7403,7414,7430,7490,7855,8289,8324,8543,8549,8829,8854,9231,9314,9355,9723,9788,9839,9863,9948,10082,10252,10371,10395,10512,10672,10818,10890,10959,11019,11235,23002,23122,23198,23213,23767,26146,28514,29072,51804,54806,55727,55966,57154,57534,64398,64399,64750,65009,79633,80000,81839,84133,84678,85407,94134,130497,157506,166336 | ACVR1,ADM,AR,BCL2,BMP2,DDR1,CDC42,AP2M1,CLTC,CTNNB1,DLG1,DLG3,EDN1,EPHA4,EPHA7,EYA1,GPC4,FGF7,FGF10,FGFR2,FOXF1,FOXF2,FRZB,GDNF,GLI2,GLI3,HOXA5,HOXB7,HOXD13,RBPJ,JAG2,KRAS,LRP2,LRP6,MEF2C,MET,MLLT3,ROR1,PAFAH1B1,PLXNA1,PRKACB,PRKX,PROX1,PSMD5,PTCH1,PTEN,RAC1,RALA,RAP2A,SALL1,SKI,SNAI2,TFAP2A,TGFB2,TGFBR2,NKX2-1,KDM6A,VCL,EZR,WT1,FZD5,ARID1A,FZD7,LMO4,LGR5,NRP1,ALDH1A2,DLG5,KLF4,LHX2,SEMA3E,MTSS1,ZEB2,MAGI2,WDR1,GPC6,SPRY1,SEMA3A,DLC1,SEMA3C,GNA13,FRS2,RAB10,TMED2,LIAS,PDCD10,DAAM1,CLASP2,PSME4,SULF1,FLRT3,TRAF3IP1,DLL1,SETD2,SIX4,AHI1,BTBD7,AJAP1,SMURF1,MIB1,MPP5,HHIP,SMURF2,NDRG4,FAT4,GREB1L,VANGL1,ZNRF3,KDM2B,NKD1,ARHGAP12,OSR1,RDH10,PRICKLE2 |
| 10_Member | GO Biological Processes | GO:0061138 | morphogenesis of a branching epithelium | -11.88960761 | -9.762 | 47/182 | 90,133,367,596,650,780,1499,1739,1906,2138,2252,2255,2263,2294,2668,2736,2737,3202,3217,3239,3845,4040,4233,5361,5629,5727,6299,6591,7048,7080,7490,7855,8829,9231,9723,10252,10371,10512,10672,10818,23213,51804,55727,64399,79633,80000,157506 | ACVR1,ADM,AR,BCL2,BMP2,DDR1,CTNNB1,DLG1,EDN1,EYA1,FGF7,FGF10,FGFR2,FOXF1,GDNF,GLI2,GLI3,HOXA5,HOXB7,HOXD13,KRAS,LRP6,MET,PLXNA1,PROX1,PTCH1,SALL1,SNAI2,TGFBR2,NKX2-1,WT1,FZD5,NRP1,DLG5,SEMA3E,SPRY1,SEMA3A,SEMA3C,GNA13,FRS2,SULF1,SIX4,BTBD7,HHIP,FAT4,GREB1L,RDH10 |
| 10_Member | GO Biological Processes | GO:0001763 | morphogenesis of a branching structure | -11.79649129 | -9.671 | 49/196 | 90,133,367,596,650,780,1499,1739,1906,2045,2138,2252,2255,2263,2294,2668,2736,2737,3202,3217,3239,3845,4040,4233,5361,5629,5727,6299,6591,7048,7080,7490,7855,8829,9231,9723,10252,10371,10512,10672,10818,23213,29072,51804,55727,64399,79633,80000,157506 | ACVR1,ADM,AR,BCL2,BMP2,DDR1,CTNNB1,DLG1,EDN1,EPHA7,EYA1,FGF7,FGF10,FGFR2,FOXF1,GDNF,GLI2,GLI3,HOXA5,HOXB7,HOXD13,KRAS,LRP6,MET,PLXNA1,PROX1,PTCH1,SALL1,SNAI2,TGFBR2,NKX2-1,WT1,FZD5,NRP1,DLG5,SEMA3E,SPRY1,SEMA3A,SEMA3C,GNA13,FRS2,SULF1,SETD2,SIX4,BTBD7,HHIP,FAT4,GREB1L,RDH10 |
| 10_Member | GO Biological Processes | GO:0001655 | urogenital system development | -11.66434065 | -9.552 | 68/331 | 59,367,596,650,793,1027,1028,1399,1499,1739,1958,2018,2043,2045,2048,2049,2138,2255,2263,2294,2296,2624,2668,2736,2737,3217,3239,3655,4091,4208,4643,5125,5156,5613,5629,5727,5728,5915,6092,6299,6492,7010,7020,7042,7046,7337,7490,8549,8829,8854,9231,9510,9788,9863,10252,10818,23213,25937,26146,28514,28999,51804,54806,79633,80000,84159,130497,157506 | ACTA2,AR,BCL2,BMP2,CALB1,CDKN1B,CDKN1C,CRKL,CTNNB1,DLG1,EGR1,EMX2,EPHA4,EPHA7,EPHB2,EPHB3,EYA1,FGF10,FGFR2,FOXF1,FOXC1,GATA2,GDNF,GLI2,GLI3,HOXB7,HOXD13,ITGA6,SMAD6,MEF2C,MYO1E,PCSK5,PDGFRA,PRKX,PROX1,PTCH1,PTEN,RARB,ROBO2,SALL1,SIM1,TEK,TFAP2A,TGFB2,TGFBR1,UBE3A,WT1,LGR5,NRP1,ALDH1A2,DLG5,ADAMTS1,MTSS1,MAGI2,SPRY1,FRS2,SULF1,WWTR1,TRAF3IP1,DLL1,KLF15,SIX4,AHI1,FAT4,GREB1L,ARID5B,OSR1,RDH10 |
| 10_Member | GO Biological Processes | GO:0060562 | epithelial tube morphogenesis | -10.37046656 | -8.376 | 64/322 | 90,133,367,596,650,780,1499,1739,1906,2043,2045,2138,2255,2263,2294,2668,2736,2737,3202,3217,3516,3845,4036,4040,4208,4233,5567,5613,5629,5727,5898,6299,6497,7042,7048,7080,7403,7490,8289,8543,8549,8829,9231,9355,9723,9788,9839,10252,10395,10672,10959,11019,28514,29072,51804,54806,57534,64399,65009,79633,80000,84678,130497,157506 | ACVR1,ADM,AR,BCL2,BMP2,DDR1,CTNNB1,DLG1,EDN1,EPHA4,EPHA7,EYA1,FGF10,FGFR2,FOXF1,GDNF,GLI2,GLI3,HOXA5,HOXB7,RBPJ,KRAS,LRP2,LRP6,MEF2C,MET,PRKACB,PRKX,PROX1,PTCH1,RALA,SALL1,SKI,TGFB2,TGFBR2,NKX2-1,KDM6A,WT1,ARID1A,LMO4,LGR5,NRP1,DLG5,LHX2,SEMA3E,MTSS1,ZEB2,SPRY1,DLC1,GNA13,TMED2,LIAS,DLL1,SETD2,SIX4,AHI1,MIB1,HHIP,NDRG4,FAT4,GREB1L,KDM2B,OSR1,RDH10 |
| 10_Member | GO Biological Processes | GO:0072001 | renal system development | -9.332291667 | -7.418 | 58/294 | 59,596,650,793,1028,1499,1739,1958,2018,2043,2045,2138,2255,2263,2294,2296,2668,2736,2737,3217,3655,4091,4208,4643,5125,5156,5613,5629,5727,5915,6092,6299,6492,7010,7020,7042,7046,7490,8549,8829,8854,9231,9510,9788,9863,10252,23213,25937,26146,28514,28999,51804,54806,79633,80000,84159,130497,157506 | ACTA2,BCL2,BMP2,CALB1,CDKN1C,CTNNB1,DLG1,EGR1,EMX2,EPHA4,EPHA7,EYA1,FGF10,FGFR2,FOXF1,FOXC1,GDNF,GLI2,GLI3,HOXB7,ITGA6,SMAD6,MEF2C,MYO1E,PCSK5,PDGFRA,PRKX,PROX1,PTCH1,RARB,ROBO2,SALL1,SIM1,TEK,TFAP2A,TGFB2,TGFBR1,WT1,LGR5,NRP1,ALDH1A2,DLG5,ADAMTS1,MTSS1,MAGI2,SPRY1,SULF1,WWTR1,TRAF3IP1,DLL1,KLF15,SIX4,AHI1,FAT4,GREB1L,ARID5B,OSR1,RDH10 |
| 10_Member | GO Biological Processes | GO:0001822 | kidney development | -8.429254936 | -6.596 | 54/279 | 59,596,650,793,1028,1499,1739,1958,2043,2045,2138,2255,2263,2296,2668,2736,2737,3217,4091,4208,4643,5125,5156,5613,5629,5727,5915,6092,6299,6492,7010,7020,7042,7046,7490,8829,8854,9231,9510,9788,9863,10252,23213,25937,26146,28514,28999,51804,54806,79633,80000,84159,130497,157506 | ACTA2,BCL2,BMP2,CALB1,CDKN1C,CTNNB1,DLG1,EGR1,EPHA4,EPHA7,EYA1,FGF10,FGFR2,FOXC1,GDNF,GLI2,GLI3,HOXB7,SMAD6,MEF2C,MYO1E,PCSK5,PDGFRA,PRKX,PROX1,PTCH1,RARB,ROBO2,SALL1,SIM1,TEK,TFAP2A,TGFB2,TGFBR1,WT1,NRP1,ALDH1A2,DLG5,ADAMTS1,MTSS1,MAGI2,SPRY1,SULF1,WWTR1,TRAF3IP1,DLL1,KLF15,SIX4,AHI1,FAT4,GREB1L,ARID5B,OSR1,RDH10 |
| 10_Member | GO Biological Processes | GO:0048754 | branching morphogenesis of an epithelial tube | -7.790422095 | -6.004 | 35/150 | 90,367,596,650,780,1499,1739,1906,2138,2255,2263,2294,2668,2736,2737,3202,3217,3845,4040,4233,5727,6299,7048,7080,7490,8829,9231,9723,10252,10672,51804,64399,79633,80000,157506 | ACVR1,AR,BCL2,BMP2,DDR1,CTNNB1,DLG1,EDN1,EYA1,FGF10,FGFR2,FOXF1,GDNF,GLI2,GLI3,HOXA5,HOXB7,KRAS,LRP6,MET,PTCH1,SALL1,TGFBR2,NKX2-1,WT1,NRP1,DLG5,SEMA3E,SPRY1,GNA13,SIX4,HHIP,FAT4,GREB1L,RDH10 |
| 10_Member | GO Biological Processes | GO:0072073 | kidney epithelium development | -7.499169887 | -5.748 | 33/140 | 596,650,793,1499,1739,2043,2045,2138,2263,2296,2668,2737,3217,4091,4208,4643,5727,5915,6092,6299,6492,7490,9788,9863,10252,25937,28514,28999,51804,54806,79633,80000,130497 | BCL2,BMP2,CALB1,CTNNB1,DLG1,EPHA4,EPHA7,EYA1,FGFR2,FOXC1,GDNF,GLI3,HOXB7,SMAD6,MEF2C,MYO1E,PTCH1,RARB,ROBO2,SALL1,SIM1,WT1,MTSS1,MAGI2,SPRY1,WWTR1,DLL1,KLF15,SIX4,AHI1,FAT4,GREB1L,OSR1 |
| 10_Member | GO Biological Processes | GO:0072006 | nephron development | -5.729248071 | -4.178 | 30/143 | 59,596,650,793,1499,1739,1958,2138,2296,2668,2737,3217,4208,4643,5156,5727,6299,7010,7490,9788,9863,23213,25937,28514,28999,51804,54806,79633,80000,130497 | ACTA2,BCL2,BMP2,CALB1,CTNNB1,DLG1,EGR1,EYA1,FOXC1,GDNF,GLI3,HOXB7,MEF2C,MYO1E,PDGFRA,PTCH1,SALL1,TEK,WT1,MTSS1,MAGI2,SULF1,WWTR1,DLL1,KLF15,SIX4,AHI1,FAT4,GREB1L,OSR1 |
| 10_Member | GO Biological Processes | GO:0001823 | mesonephros development | -5.623495001 | -4.086 | 24/102 | 596,650,793,1499,1739,2138,2255,2263,2296,2668,2737,3217,4091,5727,5915,6092,6299,6492,7490,10252,51804,79633,80000,130497 | BCL2,BMP2,CALB1,CTNNB1,DLG1,EYA1,FGF10,FGFR2,FOXC1,GDNF,GLI3,HOXB7,SMAD6,PTCH1,RARB,ROBO2,SALL1,SIM1,WT1,SPRY1,SIX4,FAT4,GREB1L,OSR1 |
| 10_Member | GO Biological Processes | GO:0072009 | nephron epithelium development | -5.613432255 | -4.080 | 25/109 | 596,650,793,1499,1739,2138,2296,2668,2737,3217,4208,4643,5727,6299,7490,9788,9863,25937,28514,28999,51804,54806,79633,80000,130497 | BCL2,BMP2,CALB1,CTNNB1,DLG1,EYA1,FOXC1,GDNF,GLI3,HOXB7,MEF2C,MYO1E,PTCH1,SALL1,WT1,MTSS1,MAGI2,WWTR1,DLL1,KLF15,SIX4,AHI1,FAT4,GREB1L,OSR1 |
| 10_Member | GO Biological Processes | GO:0001657 | ureteric bud development | -5.477251095 | -3.956 | 23/97 | 596,650,793,1499,1739,2138,2263,2296,2668,2737,3217,4091,5727,5915,6092,6299,6492,7490,10252,51804,79633,80000,130497 | BCL2,BMP2,CALB1,CTNNB1,DLG1,EYA1,FGFR2,FOXC1,GDNF,GLI3,HOXB7,SMAD6,PTCH1,RARB,ROBO2,SALL1,SIM1,WT1,SPRY1,SIX4,FAT4,GREB1L,OSR1 |
| 10_Member | GO Biological Processes | GO:0072164 | mesonephric tubule development | -5.396287741 | -3.888 | 23/98 | 596,650,793,1499,1739,2138,2263,2296,2668,2737,3217,4091,5727,5915,6092,6299,6492,7490,10252,51804,79633,80000,130497 | BCL2,BMP2,CALB1,CTNNB1,DLG1,EYA1,FGFR2,FOXC1,GDNF,GLI3,HOXB7,SMAD6,PTCH1,RARB,ROBO2,SALL1,SIM1,WT1,SPRY1,SIX4,FAT4,GREB1L,OSR1 |
| 10_Member | GO Biological Processes | GO:0072163 | mesonephric epithelium development | -5.396287741 | -3.888 | 23/98 | 596,650,793,1499,1739,2138,2263,2296,2668,2737,3217,4091,5727,5915,6092,6299,6492,7490,10252,51804,79633,80000,130497 | BCL2,BMP2,CALB1,CTNNB1,DLG1,EYA1,FGFR2,FOXC1,GDNF,GLI3,HOXB7,SMAD6,PTCH1,RARB,ROBO2,SALL1,SIM1,WT1,SPRY1,SIX4,FAT4,GREB1L,OSR1 |
| 10_Member | GO Biological Processes | GO:0061005 | cell differentiation involved in kidney development | -5.316072428 | -3.819 | 16/54 | 59,1499,2668,2737,4208,4643,5613,5727,6299,7490,9788,9863,25937,28999,79633,130497 | ACTA2,CTNNB1,GDNF,GLI3,MEF2C,MYO1E,PRKX,PTCH1,SALL1,WT1,MTSS1,MAGI2,WWTR1,KLF15,FAT4,OSR1 |
| 10_Member | GO Biological Processes | GO:0035850 | epithelial cell differentiation involved in kidney development | -5.125380487 | -3.645 | 14/44 | 59,1499,2668,4208,4643,5613,6299,7490,9788,9863,25937,28999,79633,130497 | ACTA2,CTNNB1,GDNF,MEF2C,MYO1E,PRKX,SALL1,WT1,MTSS1,MAGI2,WWTR1,KLF15,FAT4,OSR1 |
| 10_Member | GO Biological Processes | GO:0061326 | renal tubule development | -5.089590988 | -3.618 | 22/95 | 596,650,793,1499,1739,2138,2668,2737,3217,4208,5727,6299,7490,8549,9788,25937,28514,51804,54806,79633,80000,130497 | BCL2,BMP2,CALB1,CTNNB1,DLG1,EYA1,GDNF,GLI3,HOXB7,MEF2C,PTCH1,SALL1,WT1,LGR5,MTSS1,WWTR1,DLL1,SIX4,AHI1,FAT4,GREB1L,OSR1 |
| 10_Member | GO Biological Processes | GO:0001656 | metanephros development | -4.941238803 | -3.486 | 21/90 | 596,793,1499,1958,2138,2255,2668,2737,5156,5727,6092,6299,7490,9231,10252,25937,51804,79633,80000,130497,157506 | BCL2,CALB1,CTNNB1,EGR1,EYA1,FGF10,GDNF,GLI3,PDGFRA,PTCH1,ROBO2,SALL1,WT1,DLG5,SPRY1,WWTR1,SIX4,FAT4,GREB1L,OSR1,RDH10 |
| 10_Member | GO Biological Processes | GO:0061333 | renal tubule morphogenesis | -4.813560431 | -3.367 | 19/78 | 596,650,1499,1739,2138,2668,2737,3217,4208,5727,6299,7490,8549,9788,51804,54806,79633,80000,130497 | BCL2,BMP2,CTNNB1,DLG1,EYA1,GDNF,GLI3,HOXB7,MEF2C,PTCH1,SALL1,WT1,LGR5,MTSS1,SIX4,AHI1,FAT4,GREB1L,OSR1 |
| 10_Member | GO Biological Processes | GO:0072080 | nephron tubule development | -4.708668327 | -3.279 | 21/93 | 596,650,793,1499,1739,2138,2668,2737,3217,4208,5727,6299,7490,9788,25937,28514,51804,54806,79633,80000,130497 | BCL2,BMP2,CALB1,CTNNB1,DLG1,EYA1,GDNF,GLI3,HOXB7,MEF2C,PTCH1,SALL1,WT1,MTSS1,WWTR1,DLL1,SIX4,AHI1,FAT4,GREB1L,OSR1 |
| 10_Member | GO Biological Processes | GO:0060993 | kidney morphogenesis | -4.056273846 | -2.721 | 20/95 | 596,650,793,1499,1739,2138,2255,2668,2737,3217,5613,5727,6299,7490,25937,51804,54806,79633,80000,130497 | BCL2,BMP2,CALB1,CTNNB1,DLG1,EYA1,FGF10,GDNF,GLI3,HOXB7,PRKX,PTCH1,SALL1,WT1,WWTR1,SIX4,AHI1,FAT4,GREB1L,OSR1 |
| 10_Member | GO Biological Processes | GO:0032835 | glomerulus development | -3.807825812 | -2.510 | 15/63 | 59,596,1958,2296,4208,4643,5156,7010,7490,9788,9863,23213,25937,28999,130497 | ACTA2,BCL2,EGR1,FOXC1,MEF2C,MYO1E,PDGFRA,TEK,WT1,MTSS1,MAGI2,SULF1,WWTR1,KLF15,OSR1 |
| 10_Member | GO Biological Processes | GO:0001658 | branching involved in ureteric bud morphogenesis | -3.576004176 | -2.322 | 14/59 | 596,650,1499,1739,2138,2668,2737,3217,5727,6299,7490,51804,79633,80000 | BCL2,BMP2,CTNNB1,DLG1,EYA1,GDNF,GLI3,HOXB7,PTCH1,SALL1,WT1,SIX4,FAT4,GREB1L |
| 10_Member | GO Biological Processes | GO:0072171 | mesonephric tubule morphogenesis | -3.568774758 | -2.316 | 15/66 | 596,650,1499,1739,2138,2668,2737,3217,5727,6299,7490,51804,79633,80000,130497 | BCL2,BMP2,CTNNB1,DLG1,EYA1,GDNF,GLI3,HOXB7,PTCH1,SALL1,WT1,SIX4,FAT4,GREB1L,OSR1 |
| 10_Member | GO Biological Processes | GO:0072078 | nephron tubule morphogenesis | -3.502802204 | -2.259 | 16/74 | 596,650,1499,1739,2138,2668,2737,3217,5727,6299,7490,51804,54806,79633,80000,130497 | BCL2,BMP2,CTNNB1,DLG1,EYA1,GDNF,GLI3,HOXB7,PTCH1,SALL1,WT1,SIX4,AHI1,FAT4,GREB1L,OSR1 |
| 10_Member | GO Biological Processes | GO:0072088 | nephron epithelium morphogenesis | -3.364622691 | -2.145 | 16/76 | 596,650,1499,1739,2138,2668,2737,3217,5727,6299,7490,51804,54806,79633,80000,130497 | BCL2,BMP2,CTNNB1,DLG1,EYA1,GDNF,GLI3,HOXB7,PTCH1,SALL1,WT1,SIX4,AHI1,FAT4,GREB1L,OSR1 |
| 10_Member | GO Biological Processes | GO:0072028 | nephron morphogenesis | -3.23238413 | -2.037 | 16/78 | 596,650,1499,1739,2138,2668,2737,3217,5727,6299,7490,51804,54806,79633,80000,130497 | BCL2,BMP2,CTNNB1,DLG1,EYA1,GDNF,GLI3,HOXB7,PTCH1,SALL1,WT1,SIX4,AHI1,FAT4,GREB1L,OSR1 |
| 10_Member | GO Biological Processes | GO:0060675 | ureteric bud morphogenesis | -3.118914121 | -1.948 | 14/65 | 596,650,1499,1739,2138,2668,2737,3217,5727,6299,7490,51804,79633,80000 | BCL2,BMP2,CTNNB1,DLG1,EYA1,GDNF,GLI3,HOXB7,PTCH1,SALL1,WT1,SIX4,FAT4,GREB1L |
| 10_Member | GO Biological Processes | GO:0072160 | nephron tubule epithelial cell differentiation | -3.073518547 | -1.911 | 6/15 | 1499,4208,9788,25937,79633,130497 | CTNNB1,MEF2C,MTSS1,WWTR1,FAT4,OSR1 |
| 11_Summary | GO Biological Processes | GO:1905114 | cell surface receptor signaling pathway involved in cell-cell signaling | -22.31231404 | -19.377 | 130/625 | 18,324,650,865,998,1173,1213,1457,1499,1540,1654,1749,1896,1958,2239,2255,2263,2308,2487,2736,2737,2742,2743,2776,2782,2887,2895,2898,2903,3516,4040,4139,4208,4233,4286,4300,4919,4929,5218,5494,5515,5530,5534,5563,5590,5711,5728,5868,5879,6299,6497,6500,6591,6622,6801,6885,6934,7088,7314,7320,7403,7855,8324,8434,8452,8549,8724,9101,9209,9314,9559,9679,9699,9736,9839,9863,10042,10082,10146,10159,10979,22871,22941,22999,23002,23112,23168,23189,23198,23213,23291,23613,25937,26524,26959,27123,27185,27303,27327,29969,51699,51701,53944,55031,55182,55681,55737,55789,57154,57690,64750,79577,79718,80114,81029,81832,81839,84133,85407,85458,122011,145258,147495,153090,166336,169200,192670,219287,219771,100113407 | ABAT,APC,BMP2,CBFB,CDC42,AP2M1,CLTC,CSNK2A1,CTNNB1,CYLD,DDX3X,DLX5,EDA,EGR1,GPC4,FGF10,FGFR2,FOXO1,FRZB,GLI2,GLI3,GLRA2,GLRB,GNAQ,GNB1,GRB10,GRID2,GRIK2,GRIN2A,RBPJ,LRP6,MARK1,MEF2C,MET,MITF,MLLT3,ROR1,NR4A2,CDK14,PPM1A,PPP2CA,PPP3CA,PPP3R1,PRKAA2,PRKCZ,PSMD5,PTEN,RAB5A,RAC1,SALL1,SKI,SKP1,SNAI2,SNCA,STRN,MAP3K7,TCF7L2,TLE1,UBB,UBE2B,KDM6A,FZD5,FZD7,RECK,CUL3,LGR5,SNX3,USP8,LRRFIP2,KLF4,VPS26A,FAM53B,RIMS2,USP34,ZEB2,MAGI2,HMGXB4,GPC6,G3BP1,ATP6AP2,FERMT2,NLGN1,SHANK2,RIMS1,DAAM1,TNRC6B,RTF1,KANK1,PSME4,SULF1,FBXW11,ZMYND8,WWTR1,LATS2,HBP1,DKK2,DISC1,RBMS3,TNRC6A,MDFIC,VPS29,NLK,CSNK1G1,USP47,RNF220,SCYL2,VPS35,DEPDC1B,SMURF1,TNRC6C,SMURF2,CDC73,TBL1XR1,BICC1,WNT5B,NETO1,VANGL1,ZNRF3,NKD1,DIXDC1,CSNK1A1L,GSC,APCDD1,DAB2IP,PRICKLE2,TMEM64,AGO4,AMER2,CCNY,TMEM170B |
| 11_Member | GO Biological Processes | GO:1905114 | cell surface receptor signaling pathway involved in cell-cell signaling | -22.31231404 | -19.377 | 130/625 | 18,324,650,865,998,1173,1213,1457,1499,1540,1654,1749,1896,1958,2239,2255,2263,2308,2487,2736,2737,2742,2743,2776,2782,2887,2895,2898,2903,3516,4040,4139,4208,4233,4286,4300,4919,4929,5218,5494,5515,5530,5534,5563,5590,5711,5728,5868,5879,6299,6497,6500,6591,6622,6801,6885,6934,7088,7314,7320,7403,7855,8324,8434,8452,8549,8724,9101,9209,9314,9559,9679,9699,9736,9839,9863,10042,10082,10146,10159,10979,22871,22941,22999,23002,23112,23168,23189,23198,23213,23291,23613,25937,26524,26959,27123,27185,27303,27327,29969,51699,51701,53944,55031,55182,55681,55737,55789,57154,57690,64750,79577,79718,80114,81029,81832,81839,84133,85407,85458,122011,145258,147495,153090,166336,169200,192670,219287,219771,100113407 | ABAT,APC,BMP2,CBFB,CDC42,AP2M1,CLTC,CSNK2A1,CTNNB1,CYLD,DDX3X,DLX5,EDA,EGR1,GPC4,FGF10,FGFR2,FOXO1,FRZB,GLI2,GLI3,GLRA2,GLRB,GNAQ,GNB1,GRB10,GRID2,GRIK2,GRIN2A,RBPJ,LRP6,MARK1,MEF2C,MET,MITF,MLLT3,ROR1,NR4A2,CDK14,PPM1A,PPP2CA,PPP3CA,PPP3R1,PRKAA2,PRKCZ,PSMD5,PTEN,RAB5A,RAC1,SALL1,SKI,SKP1,SNAI2,SNCA,STRN,MAP3K7,TCF7L2,TLE1,UBB,UBE2B,KDM6A,FZD5,FZD7,RECK,CUL3,LGR5,SNX3,USP8,LRRFIP2,KLF4,VPS26A,FAM53B,RIMS2,USP34,ZEB2,MAGI2,HMGXB4,GPC6,G3BP1,ATP6AP2,FERMT2,NLGN1,SHANK2,RIMS1,DAAM1,TNRC6B,RTF1,KANK1,PSME4,SULF1,FBXW11,ZMYND8,WWTR1,LATS2,HBP1,DKK2,DISC1,RBMS3,TNRC6A,MDFIC,VPS29,NLK,CSNK1G1,USP47,RNF220,SCYL2,VPS35,DEPDC1B,SMURF1,TNRC6C,SMURF2,CDC73,TBL1XR1,BICC1,WNT5B,NETO1,VANGL1,ZNRF3,NKD1,DIXDC1,CSNK1A1L,GSC,APCDD1,DAB2IP,PRICKLE2,TMEM64,AGO4,AMER2,CCNY,TMEM170B |
| 11_Member | GO Biological Processes | GO:0016055 | Wnt signaling pathway | -21.38392234 | -18.525 | 114/522 | 324,650,865,998,1173,1213,1457,1499,1540,1654,1749,1896,1958,2239,2255,2263,2308,2487,2737,2776,2782,2887,3516,4040,4139,4233,4286,4300,4919,4929,5218,5494,5515,5530,5534,5563,5711,5728,5868,5879,6299,6497,6500,6591,6801,6885,6934,7088,7314,7320,7403,7855,8324,8434,8452,8549,8724,9101,9209,9314,9559,9679,9736,9839,9863,10042,10082,10146,10159,10979,23002,23112,23168,23189,23198,23213,23291,25937,26524,26959,27123,27185,27303,27327,29969,51699,51701,53944,55031,55182,55681,55737,55789,57154,57690,64750,79577,79718,80114,81029,81839,84133,85407,85458,122011,145258,147495,153090,166336,169200,192670,219287,219771,100113407 | APC,BMP2,CBFB,CDC42,AP2M1,CLTC,CSNK2A1,CTNNB1,CYLD,DDX3X,DLX5,EDA,EGR1,GPC4,FGF10,FGFR2,FOXO1,FRZB,GLI3,GNAQ,GNB1,GRB10,RBPJ,LRP6,MARK1,MET,MITF,MLLT3,ROR1,NR4A2,CDK14,PPM1A,PPP2CA,PPP3CA,PPP3R1,PRKAA2,PSMD5,PTEN,RAB5A,RAC1,SALL1,SKI,SKP1,SNAI2,STRN,MAP3K7,TCF7L2,TLE1,UBB,UBE2B,KDM6A,FZD5,FZD7,RECK,CUL3,LGR5,SNX3,USP8,LRRFIP2,KLF4,VPS26A,FAM53B,USP34,ZEB2,MAGI2,HMGXB4,GPC6,G3BP1,ATP6AP2,FERMT2,DAAM1,TNRC6B,RTF1,KANK1,PSME4,SULF1,FBXW11,WWTR1,LATS2,HBP1,DKK2,DISC1,RBMS3,TNRC6A,MDFIC,VPS29,NLK,CSNK1G1,USP47,RNF220,SCYL2,VPS35,DEPDC1B,SMURF1,TNRC6C,SMURF2,CDC73,TBL1XR1,BICC1,WNT5B,VANGL1,ZNRF3,NKD1,DIXDC1,CSNK1A1L,GSC,APCDD1,DAB2IP,PRICKLE2,TMEM64,AGO4,AMER2,CCNY,TMEM170B |
| 11_Member | GO Biological Processes | GO:0198738 | cell-cell signaling by wnt | -21.24212008 | -18.397 | 114/524 | 324,650,865,998,1173,1213,1457,1499,1540,1654,1749,1896,1958,2239,2255,2263,2308,2487,2737,2776,2782,2887,3516,4040,4139,4233,4286,4300,4919,4929,5218,5494,5515,5530,5534,5563,5711,5728,5868,5879,6299,6497,6500,6591,6801,6885,6934,7088,7314,7320,7403,7855,8324,8434,8452,8549,8724,9101,9209,9314,9559,9679,9736,9839,9863,10042,10082,10146,10159,10979,23002,23112,23168,23189,23198,23213,23291,25937,26524,26959,27123,27185,27303,27327,29969,51699,51701,53944,55031,55182,55681,55737,55789,57154,57690,64750,79577,79718,80114,81029,81839,84133,85407,85458,122011,145258,147495,153090,166336,169200,192670,219287,219771,100113407 | APC,BMP2,CBFB,CDC42,AP2M1,CLTC,CSNK2A1,CTNNB1,CYLD,DDX3X,DLX5,EDA,EGR1,GPC4,FGF10,FGFR2,FOXO1,FRZB,GLI3,GNAQ,GNB1,GRB10,RBPJ,LRP6,MARK1,MET,MITF,MLLT3,ROR1,NR4A2,CDK14,PPM1A,PPP2CA,PPP3CA,PPP3R1,PRKAA2,PSMD5,PTEN,RAB5A,RAC1,SALL1,SKI,SKP1,SNAI2,STRN,MAP3K7,TCF7L2,TLE1,UBB,UBE2B,KDM6A,FZD5,FZD7,RECK,CUL3,LGR5,SNX3,USP8,LRRFIP2,KLF4,VPS26A,FAM53B,USP34,ZEB2,MAGI2,HMGXB4,GPC6,G3BP1,ATP6AP2,FERMT2,DAAM1,TNRC6B,RTF1,KANK1,PSME4,SULF1,FBXW11,WWTR1,LATS2,HBP1,DKK2,DISC1,RBMS3,TNRC6A,MDFIC,VPS29,NLK,CSNK1G1,USP47,RNF220,SCYL2,VPS35,DEPDC1B,SMURF1,TNRC6C,SMURF2,CDC73,TBL1XR1,BICC1,WNT5B,VANGL1,ZNRF3,NKD1,DIXDC1,CSNK1A1L,GSC,APCDD1,DAB2IP,PRICKLE2,TMEM64,AGO4,AMER2,CCNY,TMEM170B |
| 11_Member | GO Biological Processes | GO:0030111 | regulation of Wnt signaling pathway | -12.27123062 | -10.125 | 74/366 | 324,650,865,1457,1499,1540,1654,1749,1896,1958,2255,2263,2308,2487,2737,2776,2887,3516,4040,4300,5218,5494,5515,5711,6299,6497,6591,6934,7088,7320,8324,8434,8452,8549,8724,9101,9679,9736,9839,10042,10146,10159,23189,23198,23213,25937,26524,27123,27185,27303,29969,51701,53944,55031,55182,55681,55737,55789,64750,79577,79718,80114,81029,84133,85407,85458,122011,145258,147495,153090,169200,219287,219771,100113407 | APC,BMP2,CBFB,CSNK2A1,CTNNB1,CYLD,DDX3X,DLX5,EDA,EGR1,FGF10,FGFR2,FOXO1,FRZB,GLI3,GNAQ,GRB10,RBPJ,LRP6,MLLT3,CDK14,PPM1A,PPP2CA,PSMD5,SALL1,SKI,SNAI2,TCF7L2,TLE1,UBE2B,FZD7,RECK,CUL3,LGR5,SNX3,USP8,FAM53B,USP34,ZEB2,HMGXB4,G3BP1,ATP6AP2,KANK1,PSME4,SULF1,WWTR1,LATS2,DKK2,DISC1,RBMS3,MDFIC,NLK,CSNK1G1,USP47,RNF220,SCYL2,VPS35,DEPDC1B,SMURF2,CDC73,TBL1XR1,BICC1,WNT5B,ZNRF3,NKD1,DIXDC1,CSNK1A1L,GSC,APCDD1,DAB2IP,TMEM64,AMER2,CCNY,TMEM170B |
| 11_Member | GO Biological Processes | GO:0060070 | canonical Wnt signaling pathway | -9.617068837 | -7.678 | 64/335 | 324,650,1499,1540,1654,1749,1896,1958,2255,2263,2308,2487,2737,2776,3516,4040,4286,4300,4929,5218,5494,5711,5728,5868,6591,6934,7088,7320,7403,7855,8324,8434,8452,8549,9101,9314,9679,9736,10146,23189,23198,25937,26524,27123,27185,27303,53944,55031,55182,55681,55737,64750,79718,80114,81029,84133,85407,85458,122011,153090,169200,219287,219771,100113407 | APC,BMP2,CTNNB1,CYLD,DDX3X,DLX5,EDA,EGR1,FGF10,FGFR2,FOXO1,FRZB,GLI3,GNAQ,RBPJ,LRP6,MITF,MLLT3,NR4A2,CDK14,PPM1A,PSMD5,PTEN,RAB5A,SNAI2,TCF7L2,TLE1,UBE2B,KDM6A,FZD5,FZD7,RECK,CUL3,LGR5,USP8,KLF4,FAM53B,USP34,G3BP1,KANK1,PSME4,WWTR1,LATS2,DKK2,DISC1,RBMS3,CSNK1G1,USP47,RNF220,SCYL2,VPS35,SMURF2,TBL1XR1,BICC1,WNT5B,ZNRF3,NKD1,DIXDC1,CSNK1A1L,DAB2IP,TMEM64,AMER2,CCNY,TMEM170B |
| 11_Member | GO Biological Processes | GO:0060828 | regulation of canonical Wnt signaling pathway | -8.468082937 | -6.629 | 55/286 | 324,650,1499,1540,1654,1749,1896,1958,2255,2263,2308,2487,2737,2776,3516,4040,4300,5218,5494,5711,6591,6934,7088,7320,8324,8434,8452,8549,9101,9679,9736,10146,23189,23198,25937,26524,27123,27303,53944,55031,55182,55681,55737,64750,79718,80114,81029,84133,85407,122011,153090,169200,219287,219771,100113407 | APC,BMP2,CTNNB1,CYLD,DDX3X,DLX5,EDA,EGR1,FGF10,FGFR2,FOXO1,FRZB,GLI3,GNAQ,RBPJ,LRP6,MLLT3,CDK14,PPM1A,PSMD5,SNAI2,TCF7L2,TLE1,UBE2B,FZD7,RECK,CUL3,LGR5,USP8,FAM53B,USP34,G3BP1,KANK1,PSME4,WWTR1,LATS2,DKK2,RBMS3,CSNK1G1,USP47,RNF220,SCYL2,VPS35,SMURF2,TBL1XR1,BICC1,WNT5B,ZNRF3,NKD1,CSNK1A1L,DAB2IP,TMEM64,AMER2,CCNY,TMEM170B |
| 11_Member | GO Biological Processes | GO:0030177 | positive regulation of Wnt signaling pathway | -6.266452931 | -4.640 | 36/179 | 650,1457,1654,1749,1896,2255,2263,3516,4300,5494,5711,6299,6497,7320,8434,8549,9101,9679,9736,9839,10159,23189,23198,23213,27123,27185,53944,55031,55182,55737,55789,64750,79577,79718,85407,85458 | BMP2,CSNK2A1,DDX3X,DLX5,EDA,FGF10,FGFR2,RBPJ,MLLT3,PPM1A,PSMD5,SALL1,SKI,UBE2B,RECK,LGR5,USP8,FAM53B,USP34,ZEB2,ATP6AP2,KANK1,PSME4,SULF1,DKK2,DISC1,CSNK1G1,USP47,RNF220,VPS35,DEPDC1B,SMURF2,CDC73,TBL1XR1,NKD1,DIXDC1 |
| 11_Member | GO Biological Processes | GO:0030178 | negative regulation of Wnt signaling pathway | -4.116996048 | -2.772 | 35/213 | 324,650,1540,1958,2308,2487,2737,2887,4040,4300,5711,6591,6934,7088,8452,10042,10146,23198,25937,26524,27123,27303,51701,55681,80114,81029,84133,85407,122011,145258,147495,153090,169200,219287,100113407 | APC,BMP2,CYLD,EGR1,FOXO1,FRZB,GLI3,GRB10,LRP6,MLLT3,PSMD5,SNAI2,TCF7L2,TLE1,CUL3,HMGXB4,G3BP1,PSME4,WWTR1,LATS2,DKK2,RBMS3,NLK,SCYL2,BICC1,WNT5B,ZNRF3,NKD1,CSNK1A1L,GSC,APCDD1,DAB2IP,TMEM64,AMER2,TMEM170B |
| 11_Member | GO Biological Processes | GO:0090090 | negative regulation of canonical Wnt signaling pathway | -3.81886151 | -2.519 | 30/178 | 324,650,1540,1958,2308,2487,2737,4040,4300,5711,6591,6934,7088,8452,10146,23198,25937,26524,27123,27303,55681,80114,81029,84133,85407,122011,153090,169200,219287,100113407 | APC,BMP2,CYLD,EGR1,FOXO1,FRZB,GLI3,LRP6,MLLT3,PSMD5,SNAI2,TCF7L2,TLE1,CUL3,G3BP1,PSME4,WWTR1,LATS2,DKK2,RBMS3,SCYL2,BICC1,WNT5B,ZNRF3,NKD1,CSNK1A1L,DAB2IP,TMEM64,AMER2,TMEM170B |
| 11_Member | GO Biological Processes | GO:0090263 | positive regulation of canonical Wnt signaling pathway | -2.63032966 | -1.556 | 23/147 | 1654,1749,1896,2255,2263,3516,5494,5711,7320,8434,8549,9101,9679,9736,23189,23198,27123,53944,55031,55182,55737,64750,79718 | DDX3X,DLX5,EDA,FGF10,FGFR2,RBPJ,PPM1A,PSMD5,UBE2B,RECK,LGR5,USP8,FAM53B,USP34,KANK1,PSME4,DKK2,CSNK1G1,USP47,RNF220,VPS35,SMURF2,TBL1XR1 |
| 12_Summary | GO Molecular Functions | GO:0016301 | kinase activity | -21.57401496 | -18.671 | 148/774 | 27,90,92,657,659,780,817,894,1024,1119,1457,1607,1739,1740,2042,2043,2044,2045,2046,2048,2049,2066,2241,2261,2263,2984,3717,3815,3985,4052,4139,4140,4214,4233,4293,4638,4751,4916,4919,5062,5156,5170,5209,5218,5286,5291,5305,5563,5567,5578,5580,5587,5590,5592,5594,5597,5599,5613,5756,6093,6198,6416,6446,6733,6885,7010,7046,7048,7150,7204,8445,8491,8503,8558,8573,8621,8812,8829,9031,9061,9201,9252,9414,9448,9475,9706,9748,9833,9874,9891,9917,10000,10087,10114,10221,10290,10420,11011,11113,11183,22848,22853,23049,23097,23531,23678,26524,26750,27330,27347,28951,29110,29959,51347,51422,51701,51727,53944,54619,54861,54899,55300,55312,55500,55589,55681,56975,57551,65220,79616,79834,81617,83440,122011,139596,140609,146057,149420,150094,160851,166929,204851,255967,259230,260425,283209,285220,415116,596,2589,2590,3093,7225,161742,4842,6622,9530,10142,10178,11235,23228,50859,57600,133584,253260 | ABL2,ACVR1,ACVR2A,BMPR1A,BMPR2,DDR1,CAMK2D,CCND2,CDK8,CHKA,CSNK2A1,DGKB,DLG1,DLG2,EPHA3,EPHA4,EPHA5,EPHA7,EPHA8,EPHB2,EPHB3,ERBB4,FER,FGFR3,FGFR2,GUCY2C,JAK2,KIT,LIMK2,LTBP1,MARK1,MARK3,MAP3K1,MET,MAP3K9,MYLK,NEK2,NTRK3,ROR1,PAK2,PDGFRA,PDPK1,PFKFB3,CDK14,PIK3C2A,PIK3CB,PIP4K2A,PRKAA2,PRKACB,PRKCA,PRKCD,PRKD1,PRKCZ,PRKG1,MAPK1,MAPK6,MAPK8,PRKX,TWF1,ROCK1,RPS6KB1,MAP2K4,SGK1,SRPK2,MAP3K7,TEK,TGFBR1,TGFBR2,TOP1,TRIO,DYRK2,MAP4K3,PIK3R3,CDK10,CASK,CDK13,CCNK,NRP1,BAZ1B,PAPSS1,DCLK1,RPS6KA5,TJP2,MAP4K4,ROCK2,ULK2,SLK,MELK,TLK1,NUAK1,FAM20B,AKT3,CERT1,HIPK3,TRIB1,SPEG,TESK2,TLK2,CIT,MAP4K5,AAK1,LMTK2,SMG1,CDK19,MMD,SGK3,LATS2,RPS6KC1,RPS6KA6,STK39,TRIB2,TBK1,NRBP1,TAOK3,PRKAG2,NLK,CMPK1,CSNK1G1,CCNJ,SNRK,PXK,PI4K2B,RFK,ETNK1,BMP2K,SCYL2,FAM20C,TAOK1,NADK,CCNJL,PEAK1,CAB39L,ADPGK,CSNK1A1L,UPRT,NEK7,TTBK2,PDIK1L,SIK1,DGKH,SGMS2,HIPK1,PAN3,SGMS1,MAGI3,PGM2L1,EPHA6,PIM3,BCL2,GALNT1,GALNT2,UBE2K,TRPC6,SPRED1,NOS1,SNCA,BAG4,AKAP9,TENM1,PDCD10,PLCL2,SPOCK3,FNIP2,EGFLAM,RICTOR |
| 12_Member | GO Molecular Functions | GO:0016301 | kinase activity | -21.57401496 | -18.671 | 148/774 | 27,90,92,657,659,780,817,894,1024,1119,1457,1607,1739,1740,2042,2043,2044,2045,2046,2048,2049,2066,2241,2261,2263,2984,3717,3815,3985,4052,4139,4140,4214,4233,4293,4638,4751,4916,4919,5062,5156,5170,5209,5218,5286,5291,5305,5563,5567,5578,5580,5587,5590,5592,5594,5597,5599,5613,5756,6093,6198,6416,6446,6733,6885,7010,7046,7048,7150,7204,8445,8491,8503,8558,8573,8621,8812,8829,9031,9061,9201,9252,9414,9448,9475,9706,9748,9833,9874,9891,9917,10000,10087,10114,10221,10290,10420,11011,11113,11183,22848,22853,23049,23097,23531,23678,26524,26750,27330,27347,28951,29110,29959,51347,51422,51701,51727,53944,54619,54861,54899,55300,55312,55500,55589,55681,56975,57551,65220,79616,79834,81617,83440,122011,139596,140609,146057,149420,150094,160851,166929,204851,255967,259230,260425,283209,285220,415116 | ABL2,ACVR1,ACVR2A,BMPR1A,BMPR2,DDR1,CAMK2D,CCND2,CDK8,CHKA,CSNK2A1,DGKB,DLG1,DLG2,EPHA3,EPHA4,EPHA5,EPHA7,EPHA8,EPHB2,EPHB3,ERBB4,FER,FGFR3,FGFR2,GUCY2C,JAK2,KIT,LIMK2,LTBP1,MARK1,MARK3,MAP3K1,MET,MAP3K9,MYLK,NEK2,NTRK3,ROR1,PAK2,PDGFRA,PDPK1,PFKFB3,CDK14,PIK3C2A,PIK3CB,PIP4K2A,PRKAA2,PRKACB,PRKCA,PRKCD,PRKD1,PRKCZ,PRKG1,MAPK1,MAPK6,MAPK8,PRKX,TWF1,ROCK1,RPS6KB1,MAP2K4,SGK1,SRPK2,MAP3K7,TEK,TGFBR1,TGFBR2,TOP1,TRIO,DYRK2,MAP4K3,PIK3R3,CDK10,CASK,CDK13,CCNK,NRP1,BAZ1B,PAPSS1,DCLK1,RPS6KA5,TJP2,MAP4K4,ROCK2,ULK2,SLK,MELK,TLK1,NUAK1,FAM20B,AKT3,CERT1,HIPK3,TRIB1,SPEG,TESK2,TLK2,CIT,MAP4K5,AAK1,LMTK2,SMG1,CDK19,MMD,SGK3,LATS2,RPS6KC1,RPS6KA6,STK39,TRIB2,TBK1,NRBP1,TAOK3,PRKAG2,NLK,CMPK1,CSNK1G1,CCNJ,SNRK,PXK,PI4K2B,RFK,ETNK1,BMP2K,SCYL2,FAM20C,TAOK1,NADK,CCNJL,PEAK1,CAB39L,ADPGK,CSNK1A1L,UPRT,NEK7,TTBK2,PDIK1L,SIK1,DGKH,SGMS2,HIPK1,PAN3,SGMS1,MAGI3,PGM2L1,EPHA6,PIM3 |
| 12_Member | GO Molecular Functions | GO:0016773 | phosphotransferase activity, alcohol group as acceptor | -21.48555917 | -18.597 | 139/706 | 27,90,92,657,659,780,817,894,1024,1119,1457,1607,2042,2043,2044,2045,2046,2048,2049,2066,2241,2261,2263,2984,3717,3815,3985,4052,4139,4140,4214,4233,4293,4638,4751,4916,4919,5062,5156,5170,5209,5218,5286,5291,5305,5563,5567,5578,5580,5587,5590,5592,5594,5597,5599,5613,5756,6093,6198,6416,6446,6733,6885,7010,7046,7048,7150,7204,8445,8491,8503,8558,8573,8621,8812,8829,9031,9061,9201,9252,9448,9475,9706,9748,9833,9874,9891,9917,10000,10114,10221,10290,10420,11011,11113,11183,22848,22853,23049,23097,23531,23678,26524,26750,27330,27347,28951,29110,29959,51347,51422,51701,53944,54619,54861,54899,55300,55312,55500,55589,55681,56975,57551,65220,79616,79834,81617,83440,122011,140609,146057,149420,150094,160851,204851,255967,283209,285220,415116 | ABL2,ACVR1,ACVR2A,BMPR1A,BMPR2,DDR1,CAMK2D,CCND2,CDK8,CHKA,CSNK2A1,DGKB,EPHA3,EPHA4,EPHA5,EPHA7,EPHA8,EPHB2,EPHB3,ERBB4,FER,FGFR3,FGFR2,GUCY2C,JAK2,KIT,LIMK2,LTBP1,MARK1,MARK3,MAP3K1,MET,MAP3K9,MYLK,NEK2,NTRK3,ROR1,PAK2,PDGFRA,PDPK1,PFKFB3,CDK14,PIK3C2A,PIK3CB,PIP4K2A,PRKAA2,PRKACB,PRKCA,PRKCD,PRKD1,PRKCZ,PRKG1,MAPK1,MAPK6,MAPK8,PRKX,TWF1,ROCK1,RPS6KB1,MAP2K4,SGK1,SRPK2,MAP3K7,TEK,TGFBR1,TGFBR2,TOP1,TRIO,DYRK2,MAP4K3,PIK3R3,CDK10,CASK,CDK13,CCNK,NRP1,BAZ1B,PAPSS1,DCLK1,RPS6KA5,MAP4K4,ROCK2,ULK2,SLK,MELK,TLK1,NUAK1,FAM20B,AKT3,HIPK3,TRIB1,SPEG,TESK2,TLK2,CIT,MAP4K5,AAK1,LMTK2,SMG1,CDK19,MMD,SGK3,LATS2,RPS6KC1,RPS6KA6,STK39,TRIB2,TBK1,NRBP1,TAOK3,PRKAG2,NLK,CSNK1G1,CCNJ,SNRK,PXK,PI4K2B,RFK,ETNK1,BMP2K,SCYL2,FAM20C,TAOK1,NADK,CCNJL,PEAK1,CAB39L,ADPGK,CSNK1A1L,NEK7,TTBK2,PDIK1L,SIK1,DGKH,HIPK1,PAN3,PGM2L1,EPHA6,PIM3 |
| 12_Member | GO Molecular Functions | GO:0004672 | protein kinase activity | -20.37550097 | -17.605 | 123/603 | 27,90,92,657,659,780,817,894,1024,1457,2042,2043,2044,2045,2046,2048,2049,2066,2241,2261,2263,2984,3717,3815,3985,4052,4139,4140,4214,4233,4293,4638,4751,4916,4919,5062,5156,5170,5218,5563,5567,5578,5580,5587,5590,5592,5594,5597,5599,5613,5756,6093,6198,6416,6446,6733,6885,7010,7046,7048,7150,7204,8445,8491,8558,8573,8621,8812,8829,9031,9201,9252,9448,9475,9706,9748,9833,9874,9891,10000,10114,10221,10290,10420,11011,11113,11183,22848,22853,23049,23097,23531,23678,26524,26750,27330,27347,28951,29110,29959,51347,51422,51701,53944,54619,54861,54899,55589,55681,56975,57551,79616,79834,81617,122011,140609,146057,149420,150094,204851,255967,285220,415116 | ABL2,ACVR1,ACVR2A,BMPR1A,BMPR2,DDR1,CAMK2D,CCND2,CDK8,CSNK2A1,EPHA3,EPHA4,EPHA5,EPHA7,EPHA8,EPHB2,EPHB3,ERBB4,FER,FGFR3,FGFR2,GUCY2C,JAK2,KIT,LIMK2,LTBP1,MARK1,MARK3,MAP3K1,MET,MAP3K9,MYLK,NEK2,NTRK3,ROR1,PAK2,PDGFRA,PDPK1,CDK14,PRKAA2,PRKACB,PRKCA,PRKCD,PRKD1,PRKCZ,PRKG1,MAPK1,MAPK6,MAPK8,PRKX,TWF1,ROCK1,RPS6KB1,MAP2K4,SGK1,SRPK2,MAP3K7,TEK,TGFBR1,TGFBR2,TOP1,TRIO,DYRK2,MAP4K3,CDK10,CASK,CDK13,CCNK,NRP1,BAZ1B,DCLK1,RPS6KA5,MAP4K4,ROCK2,ULK2,SLK,MELK,TLK1,NUAK1,AKT3,HIPK3,TRIB1,SPEG,TESK2,TLK2,CIT,MAP4K5,AAK1,LMTK2,SMG1,CDK19,MMD,SGK3,LATS2,RPS6KC1,RPS6KA6,STK39,TRIB2,TBK1,NRBP1,TAOK3,PRKAG2,NLK,CSNK1G1,CCNJ,SNRK,PXK,BMP2K,SCYL2,FAM20C,TAOK1,CCNJL,PEAK1,CAB39L,CSNK1A1L,NEK7,TTBK2,PDIK1L,SIK1,HIPK1,PAN3,EPHA6,PIM3 |
| 12_Member | GO Molecular Functions | GO:0004674 | protein serine/threonine kinase activity | -14.12196874 | -11.851 | 88/441 | 90,92,657,659,817,1024,1457,3985,4052,4139,4140,4214,4293,4638,4751,5062,5170,5218,5563,5567,5578,5580,5587,5590,5592,5594,5597,5599,5613,6093,6198,6416,6446,6733,6885,7046,7048,7150,7204,8445,8491,8558,8573,8621,8812,9201,9252,9448,9475,9706,9748,9833,9874,9891,10000,10114,10290,10420,11011,11113,11183,22848,22853,23049,23097,23678,26524,26750,27330,27347,29110,29959,51347,51422,51701,53944,54861,55589,56975,57551,81617,122011,140609,146057,149420,150094,204851,415116 | ACVR1,ACVR2A,BMPR1A,BMPR2,CAMK2D,CDK8,CSNK2A1,LIMK2,LTBP1,MARK1,MARK3,MAP3K1,MAP3K9,MYLK,NEK2,PAK2,PDPK1,CDK14,PRKAA2,PRKACB,PRKCA,PRKCD,PRKD1,PRKCZ,PRKG1,MAPK1,MAPK6,MAPK8,PRKX,ROCK1,RPS6KB1,MAP2K4,SGK1,SRPK2,MAP3K7,TGFBR1,TGFBR2,TOP1,TRIO,DYRK2,MAP4K3,CDK10,CASK,CDK13,CCNK,DCLK1,RPS6KA5,MAP4K4,ROCK2,ULK2,SLK,MELK,TLK1,NUAK1,AKT3,HIPK3,SPEG,TESK2,TLK2,CIT,MAP4K5,AAK1,LMTK2,SMG1,CDK19,SGK3,LATS2,RPS6KC1,RPS6KA6,STK39,TBK1,NRBP1,TAOK3,PRKAG2,NLK,CSNK1G1,SNRK,BMP2K,FAM20C,TAOK1,CAB39L,CSNK1A1L,NEK7,TTBK2,PDIK1L,SIK1,HIPK1,PIM3 |
| 12_Member | GO Biological Processes | GO:0018210 | peptidyl-threonine modification | -7.436086573 | -5.689 | 32/134 | 90,596,817,1457,2589,2590,3093,5170,5578,5580,5587,5594,5599,6093,7046,7048,7225,8445,8558,9475,10114,11113,22853,27347,29110,51422,51701,53944,122011,146057,161742,204851 | ACVR1,BCL2,CAMK2D,CSNK2A1,GALNT1,GALNT2,UBE2K,PDPK1,PRKCA,PRKCD,PRKD1,MAPK1,MAPK8,ROCK1,TGFBR1,TGFBR2,TRPC6,DYRK2,CDK10,ROCK2,HIPK3,CIT,LMTK2,STK39,TBK1,PRKAG2,NLK,CSNK1G1,CSNK1A1L,TTBK2,SPRED1,HIPK1 |
| 12_Member | GO Biological Processes | GO:0018209 | peptidyl-serine modification | -7.021323144 | -5.307 | 56/322 | 596,817,1457,2589,2590,4139,4140,4842,4916,5062,5170,5563,5567,5578,5580,5587,5590,5592,5594,5599,5613,6093,6198,6446,6622,7046,7048,7150,8445,9201,9252,9475,9530,9874,10000,10114,10142,10178,11011,11235,22853,23049,23228,23678,26524,27347,29110,50859,51701,53944,57600,122011,133584,146057,204851,253260 | BCL2,CAMK2D,CSNK2A1,GALNT1,GALNT2,MARK1,MARK3,NOS1,NTRK3,PAK2,PDPK1,PRKAA2,PRKACB,PRKCA,PRKCD,PRKD1,PRKCZ,PRKG1,MAPK1,MAPK8,PRKX,ROCK1,RPS6KB1,SGK1,SNCA,TGFBR1,TGFBR2,TOP1,DYRK2,DCLK1,RPS6KA5,ROCK2,BAG4,TLK1,AKT3,HIPK3,AKAP9,TENM1,TLK2,PDCD10,LMTK2,SMG1,PLCL2,SGK3,LATS2,STK39,TBK1,SPOCK3,NLK,CSNK1G1,FNIP2,CSNK1A1L,EGFLAM,TTBK2,HIPK1,RICTOR |
| 12_Member | GO Biological Processes | GO:0018107 | peptidyl-threonine phosphorylation | -6.983568583 | -5.277 | 30/126 | 90,596,817,1457,3093,5170,5578,5580,5587,5594,5599,6093,7046,7048,7225,8445,8558,9475,10114,11113,22853,27347,29110,51422,51701,53944,122011,146057,161742,204851 | ACVR1,BCL2,CAMK2D,CSNK2A1,UBE2K,PDPK1,PRKCA,PRKCD,PRKD1,MAPK1,MAPK8,ROCK1,TGFBR1,TGFBR2,TRPC6,DYRK2,CDK10,ROCK2,HIPK3,CIT,LMTK2,STK39,TBK1,PRKAG2,NLK,CSNK1G1,CSNK1A1L,TTBK2,SPRED1,HIPK1 |
| 12_Member | GO Biological Processes | GO:0018105 | peptidyl-serine phosphorylation | -6.568833939 | -4.913 | 52/299 | 596,817,1457,4139,4140,4842,4916,5062,5170,5563,5567,5578,5580,5587,5590,5592,5594,5599,5613,6093,6198,6446,6622,7046,7048,7150,8445,9201,9252,9475,9530,9874,10000,10114,10142,10178,11011,11235,22853,23049,23228,23678,26524,27347,29110,51701,53944,57600,122011,146057,204851,253260 | BCL2,CAMK2D,CSNK2A1,MARK1,MARK3,NOS1,NTRK3,PAK2,PDPK1,PRKAA2,PRKACB,PRKCA,PRKCD,PRKD1,PRKCZ,PRKG1,MAPK1,MAPK8,PRKX,ROCK1,RPS6KB1,SGK1,SNCA,TGFBR1,TGFBR2,TOP1,DYRK2,DCLK1,RPS6KA5,ROCK2,BAG4,TLK1,AKT3,HIPK3,AKAP9,TENM1,TLK2,PDCD10,LMTK2,SMG1,PLCL2,SGK3,LATS2,STK39,TBK1,NLK,CSNK1G1,FNIP2,CSNK1A1L,TTBK2,HIPK1,RICTOR |
| 13_Summary | GO Biological Processes | GO:0007169 | transmembrane receptor protein tyrosine kinase signaling pathway | -21.4468931 | -18.573 | 141/722 | 60,324,367,523,526,528,627,650,780,858,868,998,1123,1173,1176,1399,1490,1499,1759,2042,2043,2044,2045,2046,2048,2049,2060,2066,2241,2252,2255,2261,2263,2296,2308,2674,2886,2887,3084,3516,3717,3815,3925,4233,4643,4916,4919,5062,5156,5170,5286,5291,5295,5431,5578,5580,5587,5590,5594,5725,5728,5770,5774,5791,5793,5801,5879,5921,6091,6093,6198,6383,6622,6653,6760,7010,7057,7073,7879,8013,8027,8036,8065,8503,8660,8717,8829,8874,8976,9185,9252,9475,9508,9655,9678,9693,9788,10019,10135,10252,10479,10552,10580,10787,10818,22853,23064,23189,23191,23213,23294,23543,23767,26060,27020,27131,28514,51552,54206,54806,55023,55294,55658,55777,56034,56975,57732,64094,64399,64855,65009,79633,84159,84959,114882,143686,147372,152831,153090,161742,285220,108,115,196,546,781,1129,1290,1385,1906,1958,2332,2554,2729,2730,2742,2776,2782,2903,2915,3184,3309,3625,3747,3784,4131,4208,4929,5028,5029,5105,5468,5567,5573,5576,5577,5888,5908,6430,6546,6547,6781,6935,7184,7186,7412,7430,7490,8850,9314,9472,9734,10142,10890,10891,22849,23175,23304,27244,28999,51274,51762,54464,57492,57724,58528,64506,80315,81035,94056,116372,132864,197131,865,1655,1977,2033,2180,2244,2494,2908,3192,3301,3624,4005,4306,4848,5563,5901,5915,5978,6092,6095,6096,7068,7182,7332,7337,7357,8202,8204,8289,8549,8554,8725,9101,9575,10499,10521,11057,23376,26524,29966,55915,56937,114907,153222,133,694,774,1027,2770,4040,4322,6429,8614,11143,51141,139596 | ACTB,APC,AR,ATP6V1A,ATP6V1B2,ATP6V1C1,BDNF,BMP2,DDR1,CAV2,CBLB,CDC42,CHN1,AP2M1,AP3S1,CRKL,CCN2,CTNNB1,DNM1,EPHA3,EPHA4,EPHA5,EPHA7,EPHA8,EPHB2,EPHB3,EPS15,ERBB4,FER,FGF7,FGF10,FGFR3,FGFR2,FOXC1,FOXO1,GFRA1,GRB7,GRB10,NRG1,RBPJ,JAK2,KIT,STMN1,MET,MYO1E,NTRK3,ROR1,PAK2,PDGFRA,PDPK1,PIK3C2A,PIK3CB,PIK3R1,POLR2B,PRKCA,PRKCD,PRKD1,PRKCZ,MAPK1,PTBP1,PTEN,PTPN1,PTPN3,PTPRE,PTPRG,PTPRR,RAC1,RASA1,ROBO1,ROCK1,RPS6KB1,SDC2,SNCA,SORL1,SS18,TEK,THBS1,TIAL1,RAB7A,NR4A3,STAM,SHOC2,CUL5,PIK3R3,IRS2,TRADD,NRP1,ARHGEF7,WASL,REPS2,RPS6KA5,ROCK2,ADAMTS3,SOCS5,PHF14,RAPGEF2,MTSS1,SH2B3,NAMPT,SPRY1,SLC9A6,ARPC1A,SORBS1,NCKAP1,FRS2,LMTK2,SETX,KANK1,CYFIP1,SULF1,ANKS1A,RBFOX2,FLRT3,APPL1,NPTN,SNX5,DLL1,RAB14,ERRFI1,AHI1,PHIP,FBXW7,RNF126,MBD5,PDGFC,FAM20C,ZFYVE28,SMOC2,HHIP,NIBAN2,NDRG4,FAT4,ARID5B,UBASH3B,OSBPL8,SESN3,CCBE1,KLB,DAB2IP,SPRED1,EPHA6,ADCY2,ADCY9,AHR,ATRX,CACNA2D1,CHRM2,COL5A2,CREB1,EDN1,EGR1,FMR1,GABRA1,GCLC,GCLM,GLRA2,GNAQ,GNB1,GRIN2A,GRM5,HNRNPD,HSPA5,INHBB,KCNC2,KCNQ1,MAP1B,MEF2C,NR4A2,P2RY1,P2RY2,PCK1,PPARG,PRKACB,PRKAR1A,PRKAR2A,PRKAR2B,RAD51,RAP1B,SRSF5,SLC8A1,SLC8A3,STC1,ZEB1,HSP90B1,TRAF2,VCAM1,EZR,WT1,KAT2B,KLF4,AKAP6,HDAC9,AKAP9,RAB10,PPARGC1A,CPEB3,LPIN1,UBR2,SESN1,KLF15,KLF3,RAB8B,XRN1,ARID1B,EPG5,RRAGD,CPEB1,CPEB4,COLEC12,SYAP1,LYPD1,CPEB2,UBR1,CBFB,DDX5,EIF4E,EP300,ACSL1,FGB,NR5A2,NR3C1,HNRNPU,DNAJA1,INHBA,LMO2,NR3C2,CNOT2,PRKAA2,RAN,RARB,REST,ROBO2,RORA,RORB,THRB,NR2C2,UBE2L3,UBE3A,UGCG,NCOA3,NRIP1,ARID1A,LGR5,PIAS1,URI1,USP8,CLOCK,NCOA2,DDX17,ABHD2,UFL1,LATS2,STRN3,LANCL2,PMEPA1,FBXO32,CREBRF,ADM,BTG1,CACNA1B,CDKN1B,GNAI1,LRP6,MMP13,SRSF4,STC2,KAT7,INSIG2,UPRT |
| 13_Member | GO Biological Processes | GO:0007169 | transmembrane receptor protein tyrosine kinase signaling pathway | -21.4468931 | -18.573 | 141/722 | 60,324,367,523,526,528,627,650,780,858,868,998,1123,1173,1176,1399,1490,1499,1759,2042,2043,2044,2045,2046,2048,2049,2060,2066,2241,2252,2255,2261,2263,2296,2308,2674,2886,2887,3084,3516,3717,3815,3925,4233,4643,4916,4919,5062,5156,5170,5286,5291,5295,5431,5578,5580,5587,5590,5594,5725,5728,5770,5774,5791,5793,5801,5879,5921,6091,6093,6198,6383,6622,6653,6760,7010,7057,7073,7879,8013,8027,8036,8065,8503,8660,8717,8829,8874,8976,9185,9252,9475,9508,9655,9678,9693,9788,10019,10135,10252,10479,10552,10580,10787,10818,22853,23064,23189,23191,23213,23294,23543,23767,26060,27020,27131,28514,51552,54206,54806,55023,55294,55658,55777,56034,56975,57732,64094,64399,64855,65009,79633,84159,84959,114882,143686,147372,152831,153090,161742,285220 | ACTB,APC,AR,ATP6V1A,ATP6V1B2,ATP6V1C1,BDNF,BMP2,DDR1,CAV2,CBLB,CDC42,CHN1,AP2M1,AP3S1,CRKL,CCN2,CTNNB1,DNM1,EPHA3,EPHA4,EPHA5,EPHA7,EPHA8,EPHB2,EPHB3,EPS15,ERBB4,FER,FGF7,FGF10,FGFR3,FGFR2,FOXC1,FOXO1,GFRA1,GRB7,GRB10,NRG1,RBPJ,JAK2,KIT,STMN1,MET,MYO1E,NTRK3,ROR1,PAK2,PDGFRA,PDPK1,PIK3C2A,PIK3CB,PIK3R1,POLR2B,PRKCA,PRKCD,PRKD1,PRKCZ,MAPK1,PTBP1,PTEN,PTPN1,PTPN3,PTPRE,PTPRG,PTPRR,RAC1,RASA1,ROBO1,ROCK1,RPS6KB1,SDC2,SNCA,SORL1,SS18,TEK,THBS1,TIAL1,RAB7A,NR4A3,STAM,SHOC2,CUL5,PIK3R3,IRS2,TRADD,NRP1,ARHGEF7,WASL,REPS2,RPS6KA5,ROCK2,ADAMTS3,SOCS5,PHF14,RAPGEF2,MTSS1,SH2B3,NAMPT,SPRY1,SLC9A6,ARPC1A,SORBS1,NCKAP1,FRS2,LMTK2,SETX,KANK1,CYFIP1,SULF1,ANKS1A,RBFOX2,FLRT3,APPL1,NPTN,SNX5,DLL1,RAB14,ERRFI1,AHI1,PHIP,FBXW7,RNF126,MBD5,PDGFC,FAM20C,ZFYVE28,SMOC2,HHIP,NIBAN2,NDRG4,FAT4,ARID5B,UBASH3B,OSBPL8,SESN3,CCBE1,KLB,DAB2IP,SPRED1,EPHA6 |
| 13_Member | GO Biological Processes | GO:1901699 | cellular response to nitrogen compound | -14.98603794 | -12.627 | 117/652 | 60,108,115,196,324,523,526,528,546,781,858,1129,1176,1290,1385,1399,1499,1906,1958,2043,2241,2308,2332,2554,2729,2730,2742,2776,2782,2886,2887,2903,2915,3184,3309,3625,3717,3747,3784,4131,4208,4929,5028,5029,5105,5156,5170,5286,5295,5468,5567,5573,5576,5577,5580,5590,5594,5728,5770,5791,5888,5908,6093,6198,6430,6546,6547,6622,6653,6781,6935,7184,7186,7412,7430,7490,8013,8503,8660,8850,9314,9472,9475,9693,9734,10135,10142,10580,10890,10891,22849,23175,23189,23191,23304,26060,27244,28999,51274,51762,54206,54464,55023,55777,56034,57492,57724,58528,64506,80315,81035,94056,114882,116372,132864,143686,197131 | ACTB,ADCY2,ADCY9,AHR,APC,ATP6V1A,ATP6V1B2,ATP6V1C1,ATRX,CACNA2D1,CAV2,CHRM2,AP3S1,COL5A2,CREB1,CRKL,CTNNB1,EDN1,EGR1,EPHA4,FER,FOXO1,FMR1,GABRA1,GCLC,GCLM,GLRA2,GNAQ,GNB1,GRB7,GRB10,GRIN2A,GRM5,HNRNPD,HSPA5,INHBB,JAK2,KCNC2,KCNQ1,MAP1B,MEF2C,NR4A2,P2RY1,P2RY2,PCK1,PDGFRA,PDPK1,PIK3C2A,PIK3R1,PPARG,PRKACB,PRKAR1A,PRKAR2A,PRKAR2B,PRKCD,PRKCZ,MAPK1,PTEN,PTPN1,PTPRE,RAD51,RAP1B,ROCK1,RPS6KB1,SRSF5,SLC8A1,SLC8A3,SNCA,SORL1,STC1,ZEB1,HSP90B1,TRAF2,VCAM1,EZR,WT1,NR4A3,PIK3R3,IRS2,KAT2B,KLF4,AKAP6,ROCK2,RAPGEF2,HDAC9,NAMPT,AKAP9,SORBS1,RAB10,PPARGC1A,CPEB3,LPIN1,KANK1,CYFIP1,UBR2,APPL1,SESN1,KLF15,KLF3,RAB8B,ERRFI1,XRN1,PHIP,MBD5,PDGFC,ARID1B,EPG5,RRAGD,CPEB1,CPEB4,COLEC12,SYAP1,OSBPL8,LYPD1,CPEB2,SESN3,UBR1 |
| 13_Member | GO Biological Processes | GO:0071417 | cellular response to organonitrogen compound | -14.11369882 | -11.846 | 107/589 | 60,108,115,196,324,523,526,528,781,858,1176,1290,1385,1499,1906,1958,2043,2241,2308,2554,2729,2730,2742,2782,2886,2887,2903,2915,3184,3309,3625,3717,3784,4131,4208,4929,5028,5029,5105,5156,5170,5286,5295,5468,5567,5573,5576,5577,5580,5590,5594,5728,5770,5791,5888,5908,6093,6198,6430,6546,6547,6622,6653,6781,6935,7184,7412,7430,7490,8013,8503,8660,8850,9314,9472,9475,9693,9734,10135,10142,10580,10890,10891,22849,23175,23189,23191,23304,26060,27244,28999,51274,51762,54206,54464,55023,55777,56034,57492,58528,64506,80315,94056,114882,132864,143686,197131 | ACTB,ADCY2,ADCY9,AHR,APC,ATP6V1A,ATP6V1B2,ATP6V1C1,CACNA2D1,CAV2,AP3S1,COL5A2,CREB1,CTNNB1,EDN1,EGR1,EPHA4,FER,FOXO1,GABRA1,GCLC,GCLM,GLRA2,GNB1,GRB7,GRB10,GRIN2A,GRM5,HNRNPD,HSPA5,INHBB,JAK2,KCNQ1,MAP1B,MEF2C,NR4A2,P2RY1,P2RY2,PCK1,PDGFRA,PDPK1,PIK3C2A,PIK3R1,PPARG,PRKACB,PRKAR1A,PRKAR2A,PRKAR2B,PRKCD,PRKCZ,MAPK1,PTEN,PTPN1,PTPRE,RAD51,RAP1B,ROCK1,RPS6KB1,SRSF5,SLC8A1,SLC8A3,SNCA,SORL1,STC1,ZEB1,HSP90B1,VCAM1,EZR,WT1,NR4A3,PIK3R3,IRS2,KAT2B,KLF4,AKAP6,ROCK2,RAPGEF2,HDAC9,NAMPT,AKAP9,SORBS1,RAB10,PPARGC1A,CPEB3,LPIN1,KANK1,CYFIP1,UBR2,APPL1,SESN1,KLF15,KLF3,RAB8B,ERRFI1,XRN1,PHIP,MBD5,PDGFC,ARID1B,RRAGD,CPEB1,CPEB4,SYAP1,OSBPL8,CPEB2,SESN3,UBR1 |
| 13_Member | GO Biological Processes | GO:0032870 | cellular response to hormone stimulus | -14.10475064 | -11.841 | 121/703 | 108,115,324,367,523,526,528,858,865,1129,1176,1385,1399,1499,1655,1906,1977,2033,2046,2180,2241,2244,2308,2494,2729,2730,2776,2782,2886,2887,2908,3184,3192,3301,3624,3625,3717,3815,4005,4131,4208,4306,4848,4929,5105,5170,5286,5295,5468,5563,5567,5573,5576,5577,5580,5590,5728,5770,5791,5901,5908,5915,5978,6092,6093,6095,6096,6198,6430,6653,6781,7068,7182,7332,7337,7357,7490,8013,8202,8204,8289,8503,8549,8554,8660,8725,8850,9101,9475,9575,9734,10135,10499,10521,10580,10890,10891,11057,23175,23189,23191,23376,23543,26060,26524,29966,51762,54206,55023,55777,55915,56937,57492,64506,94056,114882,114907,116372,132864,143686,153222 | ADCY2,ADCY9,APC,AR,ATP6V1A,ATP6V1B2,ATP6V1C1,CAV2,CBFB,CHRM2,AP3S1,CREB1,CRKL,CTNNB1,DDX5,EDN1,EIF4E,EP300,EPHA8,ACSL1,FER,FGB,FOXO1,NR5A2,GCLC,GCLM,GNAQ,GNB1,GRB7,GRB10,NR3C1,HNRNPD,HNRNPU,DNAJA1,INHBA,INHBB,JAK2,KIT,LMO2,MAP1B,MEF2C,NR3C2,CNOT2,NR4A2,PCK1,PDPK1,PIK3C2A,PIK3R1,PPARG,PRKAA2,PRKACB,PRKAR1A,PRKAR2A,PRKAR2B,PRKCD,PRKCZ,PTEN,PTPN1,PTPRE,RAN,RAP1B,RARB,REST,ROBO2,ROCK1,RORA,RORB,RPS6KB1,SRSF5,SORL1,STC1,THRB,NR2C2,UBE2L3,UBE3A,UGCG,WT1,NR4A3,NCOA3,NRIP1,ARID1A,PIK3R3,LGR5,PIAS1,IRS2,URI1,KAT2B,USP8,ROCK2,CLOCK,HDAC9,NAMPT,NCOA2,DDX17,SORBS1,RAB10,PPARGC1A,ABHD2,LPIN1,KANK1,CYFIP1,UFL1,RBFOX2,APPL1,LATS2,STRN3,RAB8B,ERRFI1,PHIP,MBD5,LANCL2,PMEPA1,ARID1B,CPEB1,SYAP1,OSBPL8,FBXO32,LYPD1,CPEB2,SESN3,CREBRF |
| 13_Member | GO Biological Processes | GO:0071375 | cellular response to peptide hormone stimulus | -8.993518304 | -7.111 | 61/323 | 108,115,324,523,526,528,858,1176,1385,1906,2241,2308,2729,2886,2887,3625,3717,4131,4929,5105,5170,5286,5295,5468,5567,5573,5576,5577,5580,5590,5728,5770,5791,5908,6093,6198,6430,6653,8013,8503,8660,8850,9475,9734,10135,10580,10890,23175,23189,23191,26060,51762,54206,55023,55777,57492,64506,94056,114882,132864,143686 | ADCY2,ADCY9,APC,ATP6V1A,ATP6V1B2,ATP6V1C1,CAV2,AP3S1,CREB1,EDN1,FER,FOXO1,GCLC,GRB7,GRB10,INHBB,JAK2,MAP1B,NR4A2,PCK1,PDPK1,PIK3C2A,PIK3R1,PPARG,PRKACB,PRKAR1A,PRKAR2A,PRKAR2B,PRKCD,PRKCZ,PTEN,PTPN1,PTPRE,RAP1B,ROCK1,RPS6KB1,SRSF5,SORL1,NR4A3,PIK3R3,IRS2,KAT2B,ROCK2,HDAC9,NAMPT,SORBS1,RAB10,LPIN1,KANK1,CYFIP1,APPL1,RAB8B,ERRFI1,PHIP,MBD5,ARID1B,CPEB1,SYAP1,OSBPL8,CPEB2,SESN3 |
| 13_Member | GO Biological Processes | GO:1901653 | cellular response to peptide | -8.863112431 | -6.991 | 69/389 | 108,115,324,523,526,528,781,858,1176,1385,1906,2043,2241,2308,2729,2886,2887,2903,2915,3625,3717,4131,4929,5105,5170,5286,5295,5468,5567,5573,5576,5577,5580,5590,5728,5770,5791,5908,6093,6198,6430,6653,7412,8013,8503,8660,8850,9314,9475,9734,10135,10580,10890,23175,23189,23191,26060,28999,51274,51762,54206,55023,55777,57492,64506,94056,114882,132864,143686 | ADCY2,ADCY9,APC,ATP6V1A,ATP6V1B2,ATP6V1C1,CACNA2D1,CAV2,AP3S1,CREB1,EDN1,EPHA4,FER,FOXO1,GCLC,GRB7,GRB10,GRIN2A,GRM5,INHBB,JAK2,MAP1B,NR4A2,PCK1,PDPK1,PIK3C2A,PIK3R1,PPARG,PRKACB,PRKAR1A,PRKAR2A,PRKAR2B,PRKCD,PRKCZ,PTEN,PTPN1,PTPRE,RAP1B,ROCK1,RPS6KB1,SRSF5,SORL1,VCAM1,NR4A3,PIK3R3,IRS2,KAT2B,KLF4,ROCK2,HDAC9,NAMPT,SORBS1,RAB10,LPIN1,KANK1,CYFIP1,APPL1,KLF15,KLF3,RAB8B,ERRFI1,PHIP,MBD5,ARID1B,CPEB1,SYAP1,OSBPL8,CPEB2,SESN3 |
| 13_Member | GO Biological Processes | GO:1901652 | response to peptide | -8.768875145 | -6.911 | 85/524 | 108,115,133,324,523,526,528,694,774,781,858,1027,1176,1385,1490,1759,1906,1958,2043,2241,2308,2729,2770,2886,2887,2903,2915,3625,3717,4040,4131,4322,4929,5105,5170,5286,5295,5468,5567,5573,5576,5577,5580,5590,5728,5770,5791,5908,6093,6198,6429,6430,6653,7010,7412,8013,8503,8614,8660,8850,9314,9475,9734,10135,10580,10890,11143,23175,23189,23191,26060,28999,51141,51274,51762,54206,55023,55777,57492,64506,94056,114882,132864,139596,143686 | ADCY2,ADCY9,ADM,APC,ATP6V1A,ATP6V1B2,ATP6V1C1,BTG1,CACNA1B,CACNA2D1,CAV2,CDKN1B,AP3S1,CREB1,CCN2,DNM1,EDN1,EGR1,EPHA4,FER,FOXO1,GCLC,GNAI1,GRB7,GRB10,GRIN2A,GRM5,INHBB,JAK2,LRP6,MAP1B,MMP13,NR4A2,PCK1,PDPK1,PIK3C2A,PIK3R1,PPARG,PRKACB,PRKAR1A,PRKAR2A,PRKAR2B,PRKCD,PRKCZ,PTEN,PTPN1,PTPRE,RAP1B,ROCK1,RPS6KB1,SRSF4,SRSF5,SORL1,TEK,VCAM1,NR4A3,PIK3R3,STC2,IRS2,KAT2B,KLF4,ROCK2,HDAC9,NAMPT,SORBS1,RAB10,KAT7,LPIN1,KANK1,CYFIP1,APPL1,KLF15,INSIG2,KLF3,RAB8B,ERRFI1,PHIP,MBD5,ARID1B,CPEB1,SYAP1,OSBPL8,CPEB2,UPRT,SESN3 |
| 13_Member | GO Biological Processes | GO:0043434 | response to peptide hormone | -8.490928337 | -6.651 | 74/438 | 108,115,133,324,523,526,528,694,858,1027,1176,1385,1490,1906,1958,2241,2308,2729,2770,2886,2887,3625,3717,4040,4131,4929,5105,5170,5286,5295,5468,5567,5573,5576,5577,5580,5590,5728,5770,5791,5908,6093,6198,6429,6430,6653,7010,8013,8503,8614,8660,8850,9475,9734,10135,10580,10890,23175,23189,23191,26060,28999,51141,51762,54206,55023,55777,57492,64506,94056,114882,132864,139596,143686 | ADCY2,ADCY9,ADM,APC,ATP6V1A,ATP6V1B2,ATP6V1C1,BTG1,CAV2,CDKN1B,AP3S1,CREB1,CCN2,EDN1,EGR1,FER,FOXO1,GCLC,GNAI1,GRB7,GRB10,INHBB,JAK2,LRP6,MAP1B,NR4A2,PCK1,PDPK1,PIK3C2A,PIK3R1,PPARG,PRKACB,PRKAR1A,PRKAR2A,PRKAR2B,PRKCD,PRKCZ,PTEN,PTPN1,PTPRE,RAP1B,ROCK1,RPS6KB1,SRSF4,SRSF5,SORL1,TEK,NR4A3,PIK3R3,STC2,IRS2,KAT2B,ROCK2,HDAC9,NAMPT,SORBS1,RAB10,LPIN1,KANK1,CYFIP1,APPL1,KLF15,INSIG2,RAB8B,ERRFI1,PHIP,MBD5,ARID1B,CPEB1,SYAP1,OSBPL8,CPEB2,UPRT,SESN3 |
| 13_Member | GO Biological Processes | GO:0032869 | cellular response to insulin stimulus | -7.552955517 | -5.799 | 44/218 | 324,523,526,528,858,1176,2241,2308,2729,2886,2887,3625,5105,5170,5286,5295,5468,5580,5590,5728,5770,5791,6198,6430,6653,8503,8660,8850,9734,10135,10580,10890,23175,23189,23191,26060,51762,54206,55023,64506,94056,114882,132864,143686 | APC,ATP6V1A,ATP6V1B2,ATP6V1C1,CAV2,AP3S1,FER,FOXO1,GCLC,GRB7,GRB10,INHBB,PCK1,PDPK1,PIK3C2A,PIK3R1,PPARG,PRKCD,PRKCZ,PTEN,PTPN1,PTPRE,RPS6KB1,SRSF5,SORL1,PIK3R3,IRS2,KAT2B,HDAC9,NAMPT,SORBS1,RAB10,LPIN1,KANK1,CYFIP1,APPL1,RAB8B,ERRFI1,PHIP,CPEB1,SYAP1,OSBPL8,CPEB2,SESN3 |
| 13_Member | GO Biological Processes | GO:0032868 | response to insulin | -7.005914904 | -5.294 | 50/274 | 133,324,523,526,528,858,1176,1958,2241,2308,2729,2886,2887,3625,5105,5170,5286,5295,5468,5580,5590,5728,5770,5791,6198,6429,6430,6653,8503,8660,8850,9734,10135,10580,10890,23175,23189,23191,26060,28999,51141,51762,54206,55023,64506,94056,114882,132864,139596,143686 | ADM,APC,ATP6V1A,ATP6V1B2,ATP6V1C1,CAV2,AP3S1,EGR1,FER,FOXO1,GCLC,GRB7,GRB10,INHBB,PCK1,PDPK1,PIK3C2A,PIK3R1,PPARG,PRKCD,PRKCZ,PTEN,PTPN1,PTPRE,RPS6KB1,SRSF4,SRSF5,SORL1,PIK3R3,IRS2,KAT2B,HDAC9,NAMPT,SORBS1,RAB10,LPIN1,KANK1,CYFIP1,APPL1,KLF15,INSIG2,RAB8B,ERRFI1,PHIP,CPEB1,SYAP1,OSBPL8,CPEB2,UPRT,SESN3 |
| 13_Member | GO Biological Processes | GO:0008286 | insulin receptor signaling pathway | -4.355866059 | -2.979 | 27/143 | 324,523,526,528,858,1176,2241,2308,2886,2887,5286,5295,5580,5590,5770,5791,6198,6653,8503,8660,10135,10580,23189,26060,55023,114882,143686 | APC,ATP6V1A,ATP6V1B2,ATP6V1C1,CAV2,AP3S1,FER,FOXO1,GRB7,GRB10,PIK3C2A,PIK3R1,PRKCD,PRKCZ,PTPN1,PTPRE,RPS6KB1,SORL1,PIK3R3,IRS2,NAMPT,SORBS1,KANK1,APPL1,PHIP,OSBPL8,SESN3 |
| 14_Summary | GO Biological Processes | GO:0061061 | muscle structure development | -21.11655251 | -18.297 | 134/674 | 90,133,467,488,596,627,650,657,663,694,858,998,1073,1106,1293,1301,1310,1385,1499,1655,1832,1906,1958,2033,2066,2115,2255,2263,2294,2296,3021,3084,3131,3192,3344,3475,3516,3688,3720,3756,3815,3845,4036,4154,4208,4211,4223,4487,4534,4604,4627,4628,4638,4773,4775,4842,5156,5530,5573,5594,5629,5725,5728,5915,5978,6095,6416,6444,6497,6546,6717,6840,6929,6934,6935,7004,7042,7046,7048,7111,7402,7490,7528,8038,8087,8289,8324,8470,8531,8554,8763,8819,9043,9318,9444,9456,9472,9474,9547,9734,9794,9948,10290,10472,10521,10818,23060,23345,23414,26057,27250,28514,50804,51460,51804,55553,55636,55796,56261,56704,57496,64855,79365,80314,84159,84665,85407,92737,93986,136319,145258,150094,389072,494470,659,1739,2138,8854,10512,10891,65009,130497,5308,7412,10252 | ACVR1,ADM,ATF3,ATP2A2,BCL2,BDNF,BMP2,BMPR1A,BNIP2,BTG1,CAV2,CDC42,CFL2,CHD2,COL6A3,COL11A1,COL19A1,CREB1,CTNNB1,DDX5,DSP,EDN1,EGR1,EP300,ERBB4,ETV1,FGF10,FGFR2,FOXF1,FOXC1,H3-3B,NRG1,HLF,HNRNPU,FOXN2,IFRD1,RBPJ,ITGB1,JARID2,KCNH1,KIT,KRAS,LRP2,MBNL1,MEF2C,MEIS1,MEOX2,MSX1,MTM1,MYBPC1,MYH9,MYH10,MYLK,NFATC2,NFATC3,NOS1,PDGFRA,PPP3CA,PRKAR1A,MAPK1,PROX1,PTBP1,PTEN,RARB,REST,RORA,MAP2K4,SGCD,SKI,SLC8A1,SRI,SVIL,TCF3,TCF7L2,ZEB1,TEAD4,TGFB2,TGFBR1,TGFBR2,TMOD1,UTRN,WT1,YY1,ADAM12,FXR1,ARID1A,FZD7,SORBS2,YBX3,PIAS1,CD164,SAP30,SPAG9,COPS2,QKI,HOMER1,AKAP6,ATG5,CXCL14,HDAC9,MAML1,WDR1,SPEG,ZBTB18,DDX17,FRS2,ZNF609,SYNE1,ZFPM2,ANKRD17,PDCD4,DLL1,MYEF2,SFMBT1,SIX4,SOX6,CHD7,MBNL3,GPCPD1,JPH1,MRTFB,NIBAN2,BHLHE41,EPC1,ARID5B,MYPN,NKD1,DNER,FOXP2,MTPN,GSC,SIK1,PLEKHM3,RNF165,BMPR2,DLG1,EYA1,ALDH1A2,SEMA3C,PPARGC1A,NDRG4,OSR1,PITX2,VCAM1,SPRY1 |
| 14_Member | GO Biological Processes | GO:0061061 | muscle structure development | -21.11655251 | -18.297 | 134/674 | 90,133,467,488,596,627,650,657,663,694,858,998,1073,1106,1293,1301,1310,1385,1499,1655,1832,1906,1958,2033,2066,2115,2255,2263,2294,2296,3021,3084,3131,3192,3344,3475,3516,3688,3720,3756,3815,3845,4036,4154,4208,4211,4223,4487,4534,4604,4627,4628,4638,4773,4775,4842,5156,5530,5573,5594,5629,5725,5728,5915,5978,6095,6416,6444,6497,6546,6717,6840,6929,6934,6935,7004,7042,7046,7048,7111,7402,7490,7528,8038,8087,8289,8324,8470,8531,8554,8763,8819,9043,9318,9444,9456,9472,9474,9547,9734,9794,9948,10290,10472,10521,10818,23060,23345,23414,26057,27250,28514,50804,51460,51804,55553,55636,55796,56261,56704,57496,64855,79365,80314,84159,84665,85407,92737,93986,136319,145258,150094,389072,494470 | ACVR1,ADM,ATF3,ATP2A2,BCL2,BDNF,BMP2,BMPR1A,BNIP2,BTG1,CAV2,CDC42,CFL2,CHD2,COL6A3,COL11A1,COL19A1,CREB1,CTNNB1,DDX5,DSP,EDN1,EGR1,EP300,ERBB4,ETV1,FGF10,FGFR2,FOXF1,FOXC1,H3-3B,NRG1,HLF,HNRNPU,FOXN2,IFRD1,RBPJ,ITGB1,JARID2,KCNH1,KIT,KRAS,LRP2,MBNL1,MEF2C,MEIS1,MEOX2,MSX1,MTM1,MYBPC1,MYH9,MYH10,MYLK,NFATC2,NFATC3,NOS1,PDGFRA,PPP3CA,PRKAR1A,MAPK1,PROX1,PTBP1,PTEN,RARB,REST,RORA,MAP2K4,SGCD,SKI,SLC8A1,SRI,SVIL,TCF3,TCF7L2,ZEB1,TEAD4,TGFB2,TGFBR1,TGFBR2,TMOD1,UTRN,WT1,YY1,ADAM12,FXR1,ARID1A,FZD7,SORBS2,YBX3,PIAS1,CD164,SAP30,SPAG9,COPS2,QKI,HOMER1,AKAP6,ATG5,CXCL14,HDAC9,MAML1,WDR1,SPEG,ZBTB18,DDX17,FRS2,ZNF609,SYNE1,ZFPM2,ANKRD17,PDCD4,DLL1,MYEF2,SFMBT1,SIX4,SOX6,CHD7,MBNL3,GPCPD1,JPH1,MRTFB,NIBAN2,BHLHE41,EPC1,ARID5B,MYPN,NKD1,DNER,FOXP2,MTPN,GSC,SIK1,PLEKHM3,RNF165 |
| 14_Member | GO Biological Processes | GO:0060537 | muscle tissue development | -15.20855185 | -12.826 | 86/409 | 90,467,596,650,657,659,858,1073,1301,1310,1385,1499,1655,1739,1832,1906,1958,2033,2066,2138,2263,2296,3084,3131,3192,3344,3475,3516,3688,3720,4036,4208,4211,4223,4534,4604,4628,4638,5156,5530,5573,5594,5629,5728,5915,6416,6444,6497,6546,6840,7042,7046,7048,7490,7528,8289,8324,8470,8531,8819,8854,9318,9456,9472,9474,9734,9794,10472,10512,10521,10818,10891,23414,28514,51804,55553,55636,56261,57496,64855,65009,92737,93986,130497,136319,150094 | ACVR1,ATF3,BCL2,BMP2,BMPR1A,BMPR2,CAV2,CFL2,COL11A1,COL19A1,CREB1,CTNNB1,DDX5,DLG1,DSP,EDN1,EGR1,EP300,ERBB4,EYA1,FGFR2,FOXC1,NRG1,HLF,HNRNPU,FOXN2,IFRD1,RBPJ,ITGB1,JARID2,LRP2,MEF2C,MEIS1,MEOX2,MTM1,MYBPC1,MYH10,MYLK,PDGFRA,PPP3CA,PRKAR1A,MAPK1,PROX1,PTEN,RARB,MAP2K4,SGCD,SKI,SLC8A1,SVIL,TGFB2,TGFBR1,TGFBR2,WT1,YY1,ARID1A,FZD7,SORBS2,YBX3,SAP30,ALDH1A2,COPS2,HOMER1,AKAP6,ATG5,HDAC9,MAML1,ZBTB18,SEMA3C,DDX17,FRS2,PPARGC1A,ZFPM2,DLL1,SIX4,SOX6,CHD7,GPCPD1,MRTFB,NIBAN2,NDRG4,DNER,FOXP2,OSR1,MTPN,SIK1 |
| 14_Member | GO Biological Processes | GO:0014706 | striated muscle tissue development | -14.46136184 | -12.154 | 82/391 | 90,467,596,650,657,659,858,1073,1301,1310,1385,1499,1655,1832,1906,1958,2033,2066,2138,2263,2296,3084,3131,3192,3344,3475,3516,3688,3720,4036,4208,4211,4223,4534,4604,4628,5156,5530,5573,5594,5629,5728,5915,6416,6444,6497,6546,6840,7042,7046,7048,7490,7528,8289,8324,8470,8531,8819,8854,9318,9456,9472,9474,9734,9794,10472,10512,10521,10818,23414,28514,51804,55553,55636,56261,57496,64855,65009,92737,93986,136319,150094 | ACVR1,ATF3,BCL2,BMP2,BMPR1A,BMPR2,CAV2,CFL2,COL11A1,COL19A1,CREB1,CTNNB1,DDX5,DSP,EDN1,EGR1,EP300,ERBB4,EYA1,FGFR2,FOXC1,NRG1,HLF,HNRNPU,FOXN2,IFRD1,RBPJ,ITGB1,JARID2,LRP2,MEF2C,MEIS1,MEOX2,MTM1,MYBPC1,MYH10,PDGFRA,PPP3CA,PRKAR1A,MAPK1,PROX1,PTEN,RARB,MAP2K4,SGCD,SKI,SLC8A1,SVIL,TGFB2,TGFBR1,TGFBR2,WT1,YY1,ARID1A,FZD7,SORBS2,YBX3,SAP30,ALDH1A2,COPS2,HOMER1,AKAP6,ATG5,HDAC9,MAML1,ZBTB18,SEMA3C,DDX17,FRS2,ZFPM2,DLL1,SIX4,SOX6,CHD7,GPCPD1,MRTFB,NIBAN2,NDRG4,DNER,FOXP2,MTPN,SIK1 |
| 14_Member | GO Biological Processes | GO:0042692 | muscle cell differentiation | -12.82456547 | -10.635 | 78/387 | 90,133,488,596,627,650,663,858,998,1073,1499,1906,2255,2263,2294,3021,3084,3192,3475,3516,3688,3756,3815,3845,4208,4487,4604,4627,4628,4773,4775,4842,5156,5530,5573,5629,5725,5915,6095,6416,6444,6497,6546,6929,6935,7111,7490,7528,8038,8289,8324,8470,8554,9043,9444,9456,9472,9474,9734,9794,9948,10290,10818,23345,26057,27250,28514,50804,51804,55553,57496,64855,79365,80314,84665,92737,136319,150094 | ACVR1,ADM,ATP2A2,BCL2,BDNF,BMP2,BNIP2,CAV2,CDC42,CFL2,CTNNB1,EDN1,FGF10,FGFR2,FOXF1,H3-3B,NRG1,HNRNPU,IFRD1,RBPJ,ITGB1,KCNH1,KIT,KRAS,MEF2C,MSX1,MYBPC1,MYH9,MYH10,NFATC2,NFATC3,NOS1,PDGFRA,PPP3CA,PRKAR1A,PROX1,PTBP1,RARB,RORA,MAP2K4,SGCD,SKI,SLC8A1,TCF3,ZEB1,TMOD1,WT1,YY1,ADAM12,ARID1A,FZD7,SORBS2,PIAS1,SPAG9,QKI,HOMER1,AKAP6,ATG5,HDAC9,MAML1,WDR1,SPEG,FRS2,SYNE1,ANKRD17,PDCD4,DLL1,MYEF2,SIX4,SOX6,MRTFB,NIBAN2,BHLHE41,EPC1,MYPN,DNER,MTPN,SIK1 |
| 14_Member | GO Biological Processes | GO:0007517 | muscle organ development | -11.47925521 | -9.377 | 78/410 | 467,596,650,657,858,1073,1106,1293,1301,1310,1385,1499,1655,1832,1906,1958,2033,2066,2115,2263,2296,3084,3131,3344,3516,3720,4036,4208,4211,4223,4487,4534,4604,4638,5530,5594,5629,5728,6444,6497,6717,6840,7004,7042,7046,7048,7402,7490,7528,8087,8324,8531,8763,8819,9318,9456,9472,9734,10290,10472,10521,10818,23060,23414,28514,51460,51804,55553,55636,56261,56704,57496,64855,84159,92737,93986,136319,145258 | ATF3,BCL2,BMP2,BMPR1A,CAV2,CFL2,CHD2,COL6A3,COL11A1,COL19A1,CREB1,CTNNB1,DDX5,DSP,EDN1,EGR1,EP300,ERBB4,ETV1,FGFR2,FOXC1,NRG1,HLF,FOXN2,RBPJ,JARID2,LRP2,MEF2C,MEIS1,MEOX2,MSX1,MTM1,MYBPC1,MYLK,PPP3CA,MAPK1,PROX1,PTEN,SGCD,SKI,SRI,SVIL,TEAD4,TGFB2,TGFBR1,TGFBR2,UTRN,WT1,YY1,FXR1,FZD7,YBX3,CD164,SAP30,COPS2,HOMER1,AKAP6,HDAC9,SPEG,ZBTB18,DDX17,FRS2,ZNF609,ZFPM2,DLL1,SFMBT1,SIX4,SOX6,CHD7,GPCPD1,JPH1,MRTFB,NIBAN2,ARID5B,DNER,FOXP2,MTPN,GSC |
| 14_Member | GO Biological Processes | GO:0048738 | cardiac muscle tissue development | -8.891168637 | -7.016 | 49/234 | 90,650,657,659,1301,1385,1832,1906,2066,2263,2296,3084,3192,3516,3688,3720,4036,4208,4211,4604,4628,5156,5573,5594,5629,5728,5915,6416,6444,6546,7042,7046,7048,7490,7528,8289,8324,8470,8854,9472,9474,9794,10818,23414,28514,55553,55636,65009,150094 | ACVR1,BMP2,BMPR1A,BMPR2,COL11A1,CREB1,DSP,EDN1,ERBB4,FGFR2,FOXC1,NRG1,HNRNPU,RBPJ,ITGB1,JARID2,LRP2,MEF2C,MEIS1,MYBPC1,MYH10,PDGFRA,PRKAR1A,MAPK1,PROX1,PTEN,RARB,MAP2K4,SGCD,SLC8A1,TGFB2,TGFBR1,TGFBR2,WT1,YY1,ARID1A,FZD7,SORBS2,ALDH1A2,AKAP6,ATG5,MAML1,FRS2,ZFPM2,DLL1,SOX6,CHD7,NDRG4,SIK1 |
| 14_Member | GO Biological Processes | GO:0051146 | striated muscle cell differentiation | -7.55846085 | -5.802 | 54/295 | 90,596,627,650,858,1073,1906,3084,3192,3516,3688,3756,3845,4208,4487,4604,4627,4628,4773,4842,5156,5530,5573,5629,5915,6416,6444,6497,6546,7111,7490,7528,8038,8289,8324,8470,9043,9456,9472,9474,9734,9794,9948,10818,28514,50804,51804,55553,64855,79365,84665,92737,136319,150094 | ACVR1,BCL2,BDNF,BMP2,CAV2,CFL2,EDN1,NRG1,HNRNPU,RBPJ,ITGB1,KCNH1,KRAS,MEF2C,MSX1,MYBPC1,MYH9,MYH10,NFATC2,NOS1,PDGFRA,PPP3CA,PRKAR1A,PROX1,RARB,MAP2K4,SGCD,SKI,SLC8A1,TMOD1,WT1,YY1,ADAM12,ARID1A,FZD7,SORBS2,SPAG9,HOMER1,AKAP6,ATG5,HDAC9,MAML1,WDR1,FRS2,DLL1,MYEF2,SIX4,SOX6,NIBAN2,BHLHE41,MYPN,DNER,MTPN,SIK1 |
| 14_Member | GO Biological Processes | GO:0035051 | cardiocyte differentiation | -6.981496056 | -5.277 | 36/168 | 90,650,1906,3084,3192,3516,3688,4208,4604,4628,5156,5308,5594,5629,5915,5978,6416,6444,6546,7042,7412,7490,7528,8289,8324,8470,9472,9474,9794,10252,10512,10818,27250,28514,55553,150094 | ACVR1,BMP2,EDN1,NRG1,HNRNPU,RBPJ,ITGB1,MEF2C,MYBPC1,MYH10,PDGFRA,PITX2,MAPK1,PROX1,RARB,REST,MAP2K4,SGCD,SLC8A1,TGFB2,VCAM1,WT1,YY1,ARID1A,FZD7,SORBS2,AKAP6,ATG5,MAML1,SPRY1,SEMA3C,FRS2,PDCD4,DLL1,SOX6,SIK1 |
| 14_Member | GO Biological Processes | GO:0055007 | cardiac muscle cell differentiation | -5.490154012 | -3.968 | 28/132 | 90,650,1906,3084,3192,3516,3688,4208,4604,4628,5156,5629,5915,6416,6444,6546,7490,7528,8289,8324,8470,9472,9474,9794,10818,28514,55553,150094 | ACVR1,BMP2,EDN1,NRG1,HNRNPU,RBPJ,ITGB1,MEF2C,MYBPC1,MYH10,PDGFRA,PROX1,RARB,MAP2K4,SGCD,SLC8A1,WT1,YY1,ARID1A,FZD7,SORBS2,AKAP6,ATG5,MAML1,FRS2,DLL1,SOX6,SIK1 |
| 14_Member | GO Biological Processes | GO:0055001 | muscle cell development | -4.594797106 | -3.180 | 33/186 | 133,488,596,858,1073,1906,3192,3688,4208,4604,4628,4773,5156,5530,5573,5629,6416,6444,6497,6546,7111,7528,8470,9456,9472,9474,9734,9794,9948,51804,64855,84665,92737 | ADM,ATP2A2,BCL2,CAV2,CFL2,EDN1,HNRNPU,ITGB1,MEF2C,MYBPC1,MYH10,NFATC2,PDGFRA,PPP3CA,PRKAR1A,PROX1,MAP2K4,SGCD,SKI,SLC8A1,TMOD1,YY1,SORBS2,HOMER1,AKAP6,ATG5,HDAC9,MAML1,WDR1,SIX4,NIBAN2,MYPN,DNER |
| 14_Member | GO Biological Processes | GO:0055002 | striated muscle cell development | -4.44156442 | -3.051 | 31/173 | 596,858,1073,1906,3192,3688,4208,4604,4628,4773,5156,5530,5573,5629,6416,6444,6497,6546,7111,7528,8470,9456,9472,9474,9734,9794,9948,51804,64855,84665,92737 | BCL2,CAV2,CFL2,EDN1,HNRNPU,ITGB1,MEF2C,MYBPC1,MYH10,NFATC2,PDGFRA,PPP3CA,PRKAR1A,PROX1,MAP2K4,SGCD,SKI,SLC8A1,TMOD1,YY1,SORBS2,HOMER1,AKAP6,ATG5,HDAC9,MAML1,WDR1,SIX4,NIBAN2,MYPN,DNER |
| 15_Summary | GO Cellular Components | GO:0048471 | perinuclear region of cytoplasm | -20.74007901 | -17.946 | 138/711 | 102,320,324,375,392,488,627,636,663,858,1490,1499,1540,1656,1739,1974,1977,2044,2181,2195,2332,2572,2589,2590,3064,3301,3624,3625,3688,3708,3799,4012,4131,4430,4745,4842,5048,5062,5066,5295,5468,5567,5577,5578,5580,5590,5727,5756,5764,5797,5801,5867,5888,6331,6418,6547,6616,6622,6683,6812,6860,7184,7430,7458,7529,7855,8087,8099,8125,8301,8470,8531,8614,8854,8867,9043,9227,9472,9693,9748,9811,9863,9919,10079,10087,10178,10397,10523,10564,10565,10890,10955,10966,11011,11076,11124,22853,23032,23162,23191,23368,23369,23607,25843,26019,26031,26052,27185,27252,29979,50640,51542,51552,51762,54521,54602,55294,55626,55681,55737,55749,56180,57414,57551,57580,57630,57724,80315,81846,92140,94056,114880,115827,130074,136319,143187,148867,202915 | ADAM10,APBA1,APC,ARF1,ARHGAP1,ATP2A2,BDNF,BICD1,BNIP2,CAV2,CCN2,CTNNB1,CYLD,DDX6,DLG1,EIF4A2,EIF4E,EPHA5,ACSL3,FAT1,FMR1,GAD2,GALNT1,GALNT2,HTT,DNAJA1,INHBA,INHBB,ITGB1,ITPR1,KIF5B,LNPEP,MAP1B,MYO1B,NELL1,NOS1,PAFAH1B1,PAK2,PAM,PIK3R1,PPARG,PRKACB,PRKAR2B,PRKCA,PRKCD,PRKCZ,PTCH1,TWF1,PTN,PTPRM,PTPRR,RAB4A,RAD51,SCN5A,SET,SLC8A3,SNAP25,SNCA,SPAST,STXBP1,SYT4,HSP90B1,EZR,EIF4H,YWHAB,FZD5,FXR1,CDK2AP1,ANP32A,PICALM,SORBS2,YBX3,STC2,ALDH1A2,SYNJ1,SPAG9,LRAT,AKAP6,RAPGEF2,SLK,CTIF,MAGI2,SEC16A,ATP9A,CERT1,TENM1,NDRG1,CHERP,ARFGEF2,ARFGEF1,RAB10,SERINC3,RAB40B,TLK2,TPPP,FAF1,LMTK2,USP33,MAPK8IP3,CYFIP1,PPP1R13B,PUM2,CD2AP,MOB4,UPF2,OSBPL3,DNM3,DISC1,KLHL20,UBQLN1,PNPLA8,VPS54,RAB14,RAB8B,WDR44,NDFIP2,FBXW7,AMBRA1,SCYL2,VPS35,CCAR1,MOSPD1,RHBDD2,TAOK1,PREX1,SH3RF1,EPG5,CPEB4,SBF2,MTDH,SYAP1,OSBPL6,RAB3C,FAM168B,MTPN,VTI1A,SLC30A7,TMEM184A |
| 15_Member | GO Cellular Components | GO:0048471 | perinuclear region of cytoplasm | -20.74007901 | -17.946 | 138/711 | 102,320,324,375,392,488,627,636,663,858,1490,1499,1540,1656,1739,1974,1977,2044,2181,2195,2332,2572,2589,2590,3064,3301,3624,3625,3688,3708,3799,4012,4131,4430,4745,4842,5048,5062,5066,5295,5468,5567,5577,5578,5580,5590,5727,5756,5764,5797,5801,5867,5888,6331,6418,6547,6616,6622,6683,6812,6860,7184,7430,7458,7529,7855,8087,8099,8125,8301,8470,8531,8614,8854,8867,9043,9227,9472,9693,9748,9811,9863,9919,10079,10087,10178,10397,10523,10564,10565,10890,10955,10966,11011,11076,11124,22853,23032,23162,23191,23368,23369,23607,25843,26019,26031,26052,27185,27252,29979,50640,51542,51552,51762,54521,54602,55294,55626,55681,55737,55749,56180,57414,57551,57580,57630,57724,80315,81846,92140,94056,114880,115827,130074,136319,143187,148867,202915 | ADAM10,APBA1,APC,ARF1,ARHGAP1,ATP2A2,BDNF,BICD1,BNIP2,CAV2,CCN2,CTNNB1,CYLD,DDX6,DLG1,EIF4A2,EIF4E,EPHA5,ACSL3,FAT1,FMR1,GAD2,GALNT1,GALNT2,HTT,DNAJA1,INHBA,INHBB,ITGB1,ITPR1,KIF5B,LNPEP,MAP1B,MYO1B,NELL1,NOS1,PAFAH1B1,PAK2,PAM,PIK3R1,PPARG,PRKACB,PRKAR2B,PRKCA,PRKCD,PRKCZ,PTCH1,TWF1,PTN,PTPRM,PTPRR,RAB4A,RAD51,SCN5A,SET,SLC8A3,SNAP25,SNCA,SPAST,STXBP1,SYT4,HSP90B1,EZR,EIF4H,YWHAB,FZD5,FXR1,CDK2AP1,ANP32A,PICALM,SORBS2,YBX3,STC2,ALDH1A2,SYNJ1,SPAG9,LRAT,AKAP6,RAPGEF2,SLK,CTIF,MAGI2,SEC16A,ATP9A,CERT1,TENM1,NDRG1,CHERP,ARFGEF2,ARFGEF1,RAB10,SERINC3,RAB40B,TLK2,TPPP,FAF1,LMTK2,USP33,MAPK8IP3,CYFIP1,PPP1R13B,PUM2,CD2AP,MOB4,UPF2,OSBPL3,DNM3,DISC1,KLHL20,UBQLN1,PNPLA8,VPS54,RAB14,RAB8B,WDR44,NDFIP2,FBXW7,AMBRA1,SCYL2,VPS35,CCAR1,MOSPD1,RHBDD2,TAOK1,PREX1,SH3RF1,EPG5,CPEB4,SBF2,MTDH,SYAP1,OSBPL6,RAB3C,FAM168B,MTPN,VTI1A,SLC30A7,TMEM184A |
| 16_Summary | GO Molecular Functions | GO:0003712 | transcription coregulator activity | -20.56383783 | -17.781 | 118/563 | 87,467,694,862,865,1385,1387,1488,1499,1810,2033,2145,2296,2957,3084,3192,4084,4335,4664,4760,4783,4848,5468,5784,5915,5994,6427,6497,6595,6598,6602,6603,6671,6760,6874,6883,6929,6935,6942,6996,7020,7029,7050,7068,7088,7158,7182,7332,7337,7342,7528,7543,7994,8202,8204,8289,8531,8553,8554,8609,8725,8819,8850,9079,9318,9443,9519,9541,9734,9794,9967,9969,10138,10140,10463,10472,10499,10521,10771,10891,11278,23013,23175,23389,23414,23429,23543,23613,25937,26039,29883,29915,51132,51460,54880,55534,55578,55749,55758,56956,57178,57492,57496,57649,57708,58508,64855,79365,79718,79733,84159,92140,116931,121536,124359,144455,166968,221895 | ACTN1,ATF3,BTG1,RUNX1T1,CBFB,CREB1,CREBBP,CTBP2,CTNNB1,DR1,EP300,EZH1,FOXC1,GTF2A1,NRG1,HNRNPU,MXD1,MNT,NAB1,NEUROD1,NFIL3,CNOT2,PPARG,PTPN14,RARB,RFXAP,SRSF2,SKI,SMARCA2,SMARCB1,SMARCD1,SMARCD2,SP4,SS18,TAF4,TAF12,TCF3,ZEB1,TCF20,TDG,TFAP2A,TFDP2,TGIF1,THRB,TLE1,TP53BP1,NR2C2,UBE2L3,UBE3A,UBP1,YY1,ZFX,KAT6A,NCOA3,NRIP1,ARID1A,YBX3,BHLHE40,PIAS1,KLF7,URI1,SAP30,KAT2B,LDB2,COPS2,MED7,TBPL1,CIR1,HDAC9,MAML1,THRAP3,MED13,YAF2,TOB1,SLC30A9,ZBTB18,NCOA2,DDX17,ZMYND11,PPARGC1A,KLF12,SPEN,LPIN1,MED13L,ZFPM2,RYBP,RBFOX2,ZMYND8,WWTR1,SS18L1,CNOT7,HCFC2,RLIM,SFMBT1,BCOR,MAML3,SUPT20H,CCAR1,RCOR3,LHX9,ZMIZ1,ARID1B,MRTFB,PHF12,MIER1,KMT2C,NIBAN2,BHLHE41,TBL1XR1,E2F8,ARID5B,MTDH,MED12L,AEBP2,CDYL2,E2F7,MIER3,JAZF1 |
| 16_Member | GO Molecular Functions | GO:0003712 | transcription coregulator activity | -20.56383783 | -17.781 | 118/563 | 87,467,694,862,865,1385,1387,1488,1499,1810,2033,2145,2296,2957,3084,3192,4084,4335,4664,4760,4783,4848,5468,5784,5915,5994,6427,6497,6595,6598,6602,6603,6671,6760,6874,6883,6929,6935,6942,6996,7020,7029,7050,7068,7088,7158,7182,7332,7337,7342,7528,7543,7994,8202,8204,8289,8531,8553,8554,8609,8725,8819,8850,9079,9318,9443,9519,9541,9734,9794,9967,9969,10138,10140,10463,10472,10499,10521,10771,10891,11278,23013,23175,23389,23414,23429,23543,23613,25937,26039,29883,29915,51132,51460,54880,55534,55578,55749,55758,56956,57178,57492,57496,57649,57708,58508,64855,79365,79718,79733,84159,92140,116931,121536,124359,144455,166968,221895 | ACTN1,ATF3,BTG1,RUNX1T1,CBFB,CREB1,CREBBP,CTBP2,CTNNB1,DR1,EP300,EZH1,FOXC1,GTF2A1,NRG1,HNRNPU,MXD1,MNT,NAB1,NEUROD1,NFIL3,CNOT2,PPARG,PTPN14,RARB,RFXAP,SRSF2,SKI,SMARCA2,SMARCB1,SMARCD1,SMARCD2,SP4,SS18,TAF4,TAF12,TCF3,ZEB1,TCF20,TDG,TFAP2A,TFDP2,TGIF1,THRB,TLE1,TP53BP1,NR2C2,UBE2L3,UBE3A,UBP1,YY1,ZFX,KAT6A,NCOA3,NRIP1,ARID1A,YBX3,BHLHE40,PIAS1,KLF7,URI1,SAP30,KAT2B,LDB2,COPS2,MED7,TBPL1,CIR1,HDAC9,MAML1,THRAP3,MED13,YAF2,TOB1,SLC30A9,ZBTB18,NCOA2,DDX17,ZMYND11,PPARGC1A,KLF12,SPEN,LPIN1,MED13L,ZFPM2,RYBP,RBFOX2,ZMYND8,WWTR1,SS18L1,CNOT7,HCFC2,RLIM,SFMBT1,BCOR,MAML3,SUPT20H,CCAR1,RCOR3,LHX9,ZMIZ1,ARID1B,MRTFB,PHF12,MIER1,KMT2C,NIBAN2,BHLHE41,TBL1XR1,E2F8,ARID5B,MTDH,MED12L,AEBP2,CDYL2,E2F7,MIER3,JAZF1 |
| 16_Member | GO Molecular Functions | GO:0003714 | transcription corepressor activity | -11.12862605 | -9.050 | 54/238 | 467,862,1387,1488,1810,2145,3192,4084,4335,4783,6427,6497,6935,7050,7068,7088,7342,7528,8204,8531,8553,8554,8725,8819,9318,9541,9734,10138,10140,10771,11278,23013,23414,23429,23543,23613,25937,29883,51132,51460,54880,55749,55758,56956,57649,57708,79365,79718,79733,121536,124359,144455,166968,221895 | ATF3,RUNX1T1,CREBBP,CTBP2,DR1,EZH1,HNRNPU,MXD1,MNT,NFIL3,SRSF2,SKI,ZEB1,TGIF1,THRB,TLE1,UBP1,YY1,NRIP1,YBX3,BHLHE40,PIAS1,URI1,SAP30,COPS2,CIR1,HDAC9,YAF2,TOB1,ZMYND11,KLF12,SPEN,ZFPM2,RYBP,RBFOX2,ZMYND8,WWTR1,CNOT7,RLIM,SFMBT1,BCOR,CCAR1,RCOR3,LHX9,PHF12,MIER1,BHLHE41,TBL1XR1,E2F8,AEBP2,CDYL2,E2F7,MIER3,JAZF1 |
| 16_Member | GO Molecular Functions | GO:0003713 | transcription coactivator activity | -10.95237659 | -8.885 | 65/320 | 87,865,1387,1488,1499,1810,2033,2296,2957,4335,4760,5468,5915,5994,6595,6598,6602,6603,6671,6760,6874,6883,6929,6935,6942,7020,7068,7182,7332,7337,7528,7543,7994,8202,8204,8289,8554,8609,8850,9443,9519,9794,9967,9969,10138,10463,10472,10499,10521,10891,23175,23414,25937,26039,29915,55534,55749,57178,57492,57496,58508,64855,84159,92140,116931 | ACTN1,CBFB,CREBBP,CTBP2,CTNNB1,DR1,EP300,FOXC1,GTF2A1,MNT,NEUROD1,PPARG,RARB,RFXAP,SMARCA2,SMARCB1,SMARCD1,SMARCD2,SP4,SS18,TAF4,TAF12,TCF3,ZEB1,TCF20,TFAP2A,THRB,NR2C2,UBE2L3,UBE3A,YY1,ZFX,KAT6A,NCOA3,NRIP1,ARID1A,PIAS1,KLF7,KAT2B,MED7,TBPL1,MAML1,THRAP3,MED13,YAF2,SLC30A9,ZBTB18,NCOA2,DDX17,PPARGC1A,LPIN1,ZFPM2,WWTR1,SS18L1,HCFC2,MAML3,CCAR1,ZMIZ1,ARID1B,MRTFB,KMT2C,NIBAN2,ARID5B,MTDH,MED12L |
| 17_Summary | GO Biological Processes | GO:0099536 | synaptic signaling | -20.2239571 | -17.475 | 138/720 | 18,320,321,375,488,590,627,774,776,783,793,1129,1268,1385,1488,1499,1607,1739,1740,1741,1759,1837,1906,1977,2043,2048,2259,2332,2554,2571,2572,2668,2742,2743,2770,2891,2895,2898,2900,2903,2911,2913,2915,3084,3688,3717,3745,3786,3799,3815,3845,4040,4131,4208,4842,4857,5028,5048,5270,5332,5376,5530,5577,5590,5594,5728,5764,5789,5868,5908,6505,6506,6507,6529,6547,6571,6616,6620,6622,6780,6812,6857,6860,7416,7419,7804,8500,8573,8618,8867,9101,9211,9342,9456,9465,9568,9590,9627,9693,9699,9873,10142,10814,11252,22849,22871,22883,22941,22987,22999,23191,23228,23373,23394,23504,23613,25769,26045,27020,27115,27185,27253,27445,54476,54899,55638,57282,57492,57616,63908,79772,81832,84961,89848,116372,149111,152330,341359,27,372,596,1602,1826,1958,2033,2115,2258,2334,2628,2764,3064,3251,4097,4211,4929,5090,6310,6323,6446,6801,7057,7060,7068,7080,7111,7432,8013,8301,8554,9698,10396,10499,10716,10752,23230,53616,54207,54806,55084,55342,55636,55737,55777,57561,57626,64856,65009,84189,84251,89797,93986,130507,153222,221935,285671,338811,342371 | ABAT,APBA1,APBA2,ARF1,ATP2A2,BCHE,BDNF,CACNA1B,CACNA1D,CACNB2,CALB1,CHRM2,CNR1,CREB1,CTBP2,CTNNB1,DGKB,DLG1,DLG2,DLG3,DNM1,DTNA,EDN1,EIF4E,EPHA4,EPHB2,FGF14,FMR1,GABRA1,GAD1,GAD2,GDNF,GLRA2,GLRB,GNAI1,GRIA2,GRID2,GRIK2,GRIK4,GRIN2A,GRM1,GRM3,GRM5,NRG1,ITGB1,JAK2,KCNB1,KCNQ3,KIF5B,KIT,KRAS,LRP6,MAP1B,MEF2C,NOS1,NOVA1,P2RY1,PAFAH1B1,SERPINE2,PLCB4,PMP22,PPP3CA,PRKAR2B,PRKCZ,MAPK1,PTEN,PTN,PTPRD,RAB5A,RAP1B,SLC1A1,SLC1A2,SLC1A3,SLC6A1,SLC8A3,SLC18A2,SNAP25,SNCB,SNCA,STAU1,STXBP1,SYT1,SYT4,VDAC1,VDAC3,LRP8,PPFIA1,CASK,CADPS,SYNJ1,USP8,LGI1,SNAP29,HOMER1,AKAP7,GABBR2,AKAP12,SNCAIP,RAPGEF2,RIMS2,FCHSD2,AKAP9,CPLX2,PACSIN2,CPEB3,NLGN1,CLSTN1,SHANK2,SV2C,RIMS1,CYFIP1,PLCL2,CRTC1,ADNP,RIMBP2,ZMYND8,SLC24A2,LRRTM2,NPTN,PDE7B,DISC1,PCDH17,PCLO,RNF216,PXK,SYBU,SLC4A10,ARID1B,TSHZ3,NAPB,MCTP1,NETO1,FBXL20,FCHSD1,LYPD1,CNIH3,CNTN4,SYT10,ABL2,ARCN1,BCL2,DACH1,DSCAM,EGR1,EP300,ETV1,FGF13,AFF2,GATM,GMFB,HTT,HPRT1,MAFG,MEIS1,NR4A2,PBX3,ATXN1,SCN1A,SGK1,STRN,THBS1,THBS4,THRB,NKX2-1,TMOD1,VIP,NR4A3,PICALM,PIAS1,PUM1,ATP8A1,NCOA2,TBR1,CHL1,VPS13A,ADAM22,KCNK10,AHI1,SOBP,STRBP,CHD7,VPS35,MBD5,ARRDC3,KLHL1,VWA1,NDRG4,SLITRK6,SGIP1,NAV2,FOXP2,UBR3,CREBRF,SDK1,RNF180,TAFA2,ATXN1L |
| 17_Member | GO Biological Processes | GO:0099536 | synaptic signaling | -20.2239571 | -17.475 | 138/720 | 18,320,321,375,488,590,627,774,776,783,793,1129,1268,1385,1488,1499,1607,1739,1740,1741,1759,1837,1906,1977,2043,2048,2259,2332,2554,2571,2572,2668,2742,2743,2770,2891,2895,2898,2900,2903,2911,2913,2915,3084,3688,3717,3745,3786,3799,3815,3845,4040,4131,4208,4842,4857,5028,5048,5270,5332,5376,5530,5577,5590,5594,5728,5764,5789,5868,5908,6505,6506,6507,6529,6547,6571,6616,6620,6622,6780,6812,6857,6860,7416,7419,7804,8500,8573,8618,8867,9101,9211,9342,9456,9465,9568,9590,9627,9693,9699,9873,10142,10814,11252,22849,22871,22883,22941,22987,22999,23191,23228,23373,23394,23504,23613,25769,26045,27020,27115,27185,27253,27445,54476,54899,55638,57282,57492,57616,63908,79772,81832,84961,89848,116372,149111,152330,341359 | ABAT,APBA1,APBA2,ARF1,ATP2A2,BCHE,BDNF,CACNA1B,CACNA1D,CACNB2,CALB1,CHRM2,CNR1,CREB1,CTBP2,CTNNB1,DGKB,DLG1,DLG2,DLG3,DNM1,DTNA,EDN1,EIF4E,EPHA4,EPHB2,FGF14,FMR1,GABRA1,GAD1,GAD2,GDNF,GLRA2,GLRB,GNAI1,GRIA2,GRID2,GRIK2,GRIK4,GRIN2A,GRM1,GRM3,GRM5,NRG1,ITGB1,JAK2,KCNB1,KCNQ3,KIF5B,KIT,KRAS,LRP6,MAP1B,MEF2C,NOS1,NOVA1,P2RY1,PAFAH1B1,SERPINE2,PLCB4,PMP22,PPP3CA,PRKAR2B,PRKCZ,MAPK1,PTEN,PTN,PTPRD,RAB5A,RAP1B,SLC1A1,SLC1A2,SLC1A3,SLC6A1,SLC8A3,SLC18A2,SNAP25,SNCB,SNCA,STAU1,STXBP1,SYT1,SYT4,VDAC1,VDAC3,LRP8,PPFIA1,CASK,CADPS,SYNJ1,USP8,LGI1,SNAP29,HOMER1,AKAP7,GABBR2,AKAP12,SNCAIP,RAPGEF2,RIMS2,FCHSD2,AKAP9,CPLX2,PACSIN2,CPEB3,NLGN1,CLSTN1,SHANK2,SV2C,RIMS1,CYFIP1,PLCL2,CRTC1,ADNP,RIMBP2,ZMYND8,SLC24A2,LRRTM2,NPTN,PDE7B,DISC1,PCDH17,PCLO,RNF216,PXK,SYBU,SLC4A10,ARID1B,TSHZ3,NAPB,MCTP1,NETO1,FBXL20,FCHSD1,LYPD1,CNIH3,CNTN4,SYT10 |
| 17_Member | GO Biological Processes | GO:0099537 | trans-synaptic signaling | -20.12342675 | -17.406 | 137/714 | 18,320,321,375,488,590,627,774,776,783,793,1129,1268,1385,1488,1499,1607,1739,1740,1741,1759,1837,1906,1977,2043,2048,2259,2332,2554,2571,2572,2668,2742,2743,2770,2891,2895,2898,2900,2903,2911,2913,2915,3688,3717,3745,3786,3799,3815,3845,4040,4131,4208,4842,4857,5028,5048,5270,5332,5376,5530,5577,5590,5594,5728,5764,5789,5868,5908,6505,6506,6507,6529,6547,6571,6616,6620,6622,6780,6812,6857,6860,7416,7419,7804,8500,8573,8618,8867,9101,9211,9342,9456,9465,9568,9590,9627,9693,9699,9873,10142,10814,11252,22849,22871,22883,22941,22987,22999,23191,23228,23373,23394,23504,23613,25769,26045,27020,27115,27185,27253,27445,54476,54899,55638,57282,57492,57616,63908,79772,81832,84961,89848,116372,149111,152330,341359 | ABAT,APBA1,APBA2,ARF1,ATP2A2,BCHE,BDNF,CACNA1B,CACNA1D,CACNB2,CALB1,CHRM2,CNR1,CREB1,CTBP2,CTNNB1,DGKB,DLG1,DLG2,DLG3,DNM1,DTNA,EDN1,EIF4E,EPHA4,EPHB2,FGF14,FMR1,GABRA1,GAD1,GAD2,GDNF,GLRA2,GLRB,GNAI1,GRIA2,GRID2,GRIK2,GRIK4,GRIN2A,GRM1,GRM3,GRM5,ITGB1,JAK2,KCNB1,KCNQ3,KIF5B,KIT,KRAS,LRP6,MAP1B,MEF2C,NOS1,NOVA1,P2RY1,PAFAH1B1,SERPINE2,PLCB4,PMP22,PPP3CA,PRKAR2B,PRKCZ,MAPK1,PTEN,PTN,PTPRD,RAB5A,RAP1B,SLC1A1,SLC1A2,SLC1A3,SLC6A1,SLC8A3,SLC18A2,SNAP25,SNCB,SNCA,STAU1,STXBP1,SYT1,SYT4,VDAC1,VDAC3,LRP8,PPFIA1,CASK,CADPS,SYNJ1,USP8,LGI1,SNAP29,HOMER1,AKAP7,GABBR2,AKAP12,SNCAIP,RAPGEF2,RIMS2,FCHSD2,AKAP9,CPLX2,PACSIN2,CPEB3,NLGN1,CLSTN1,SHANK2,SV2C,RIMS1,CYFIP1,PLCL2,CRTC1,ADNP,RIMBP2,ZMYND8,SLC24A2,LRRTM2,NPTN,PDE7B,DISC1,PCDH17,PCLO,RNF216,PXK,SYBU,SLC4A10,ARID1B,TSHZ3,NAPB,MCTP1,NETO1,FBXL20,FCHSD1,LYPD1,CNIH3,CNTN4,SYT10 |
| 17_Member | GO Biological Processes | GO:0007268 | chemical synaptic transmission | -19.69595164 | -17.015 | 135/706 | 18,320,321,375,488,590,627,774,776,783,793,1129,1268,1385,1488,1499,1607,1739,1740,1741,1759,1837,1906,1977,2043,2048,2259,2332,2554,2571,2572,2668,2742,2743,2770,2891,2895,2898,2900,2903,2911,2913,2915,3688,3717,3745,3786,3799,3815,3845,4040,4131,4208,4857,5028,5048,5270,5332,5376,5530,5577,5590,5594,5728,5764,5868,5908,6505,6506,6507,6529,6547,6571,6616,6620,6622,6780,6812,6857,6860,7416,7419,7804,8500,8573,8618,8867,9101,9211,9342,9456,9465,9568,9590,9627,9693,9699,9873,10142,10814,11252,22849,22871,22883,22941,22987,22999,23191,23228,23373,23394,23504,23613,25769,26045,27020,27115,27185,27253,27445,54476,54899,55638,57282,57492,57616,63908,79772,81832,84961,89848,116372,149111,152330,341359 | ABAT,APBA1,APBA2,ARF1,ATP2A2,BCHE,BDNF,CACNA1B,CACNA1D,CACNB2,CALB1,CHRM2,CNR1,CREB1,CTBP2,CTNNB1,DGKB,DLG1,DLG2,DLG3,DNM1,DTNA,EDN1,EIF4E,EPHA4,EPHB2,FGF14,FMR1,GABRA1,GAD1,GAD2,GDNF,GLRA2,GLRB,GNAI1,GRIA2,GRID2,GRIK2,GRIK4,GRIN2A,GRM1,GRM3,GRM5,ITGB1,JAK2,KCNB1,KCNQ3,KIF5B,KIT,KRAS,LRP6,MAP1B,MEF2C,NOVA1,P2RY1,PAFAH1B1,SERPINE2,PLCB4,PMP22,PPP3CA,PRKAR2B,PRKCZ,MAPK1,PTEN,PTN,RAB5A,RAP1B,SLC1A1,SLC1A2,SLC1A3,SLC6A1,SLC8A3,SLC18A2,SNAP25,SNCB,SNCA,STAU1,STXBP1,SYT1,SYT4,VDAC1,VDAC3,LRP8,PPFIA1,CASK,CADPS,SYNJ1,USP8,LGI1,SNAP29,HOMER1,AKAP7,GABBR2,AKAP12,SNCAIP,RAPGEF2,RIMS2,FCHSD2,AKAP9,CPLX2,PACSIN2,CPEB3,NLGN1,CLSTN1,SHANK2,SV2C,RIMS1,CYFIP1,PLCL2,CRTC1,ADNP,RIMBP2,ZMYND8,SLC24A2,LRRTM2,NPTN,PDE7B,DISC1,PCDH17,PCLO,RNF216,PXK,SYBU,SLC4A10,ARID1B,TSHZ3,NAPB,MCTP1,NETO1,FBXL20,FCHSD1,LYPD1,CNIH3,CNTN4,SYT10 |
| 17_Member | GO Biological Processes | GO:0098916 | anterograde trans-synaptic signaling | -19.69595164 | -17.015 | 135/706 | 18,320,321,375,488,590,627,774,776,783,793,1129,1268,1385,1488,1499,1607,1739,1740,1741,1759,1837,1906,1977,2043,2048,2259,2332,2554,2571,2572,2668,2742,2743,2770,2891,2895,2898,2900,2903,2911,2913,2915,3688,3717,3745,3786,3799,3815,3845,4040,4131,4208,4857,5028,5048,5270,5332,5376,5530,5577,5590,5594,5728,5764,5868,5908,6505,6506,6507,6529,6547,6571,6616,6620,6622,6780,6812,6857,6860,7416,7419,7804,8500,8573,8618,8867,9101,9211,9342,9456,9465,9568,9590,9627,9693,9699,9873,10142,10814,11252,22849,22871,22883,22941,22987,22999,23191,23228,23373,23394,23504,23613,25769,26045,27020,27115,27185,27253,27445,54476,54899,55638,57282,57492,57616,63908,79772,81832,84961,89848,116372,149111,152330,341359 | ABAT,APBA1,APBA2,ARF1,ATP2A2,BCHE,BDNF,CACNA1B,CACNA1D,CACNB2,CALB1,CHRM2,CNR1,CREB1,CTBP2,CTNNB1,DGKB,DLG1,DLG2,DLG3,DNM1,DTNA,EDN1,EIF4E,EPHA4,EPHB2,FGF14,FMR1,GABRA1,GAD1,GAD2,GDNF,GLRA2,GLRB,GNAI1,GRIA2,GRID2,GRIK2,GRIK4,GRIN2A,GRM1,GRM3,GRM5,ITGB1,JAK2,KCNB1,KCNQ3,KIF5B,KIT,KRAS,LRP6,MAP1B,MEF2C,NOVA1,P2RY1,PAFAH1B1,SERPINE2,PLCB4,PMP22,PPP3CA,PRKAR2B,PRKCZ,MAPK1,PTEN,PTN,RAB5A,RAP1B,SLC1A1,SLC1A2,SLC1A3,SLC6A1,SLC8A3,SLC18A2,SNAP25,SNCB,SNCA,STAU1,STXBP1,SYT1,SYT4,VDAC1,VDAC3,LRP8,PPFIA1,CASK,CADPS,SYNJ1,USP8,LGI1,SNAP29,HOMER1,AKAP7,GABBR2,AKAP12,SNCAIP,RAPGEF2,RIMS2,FCHSD2,AKAP9,CPLX2,PACSIN2,CPEB3,NLGN1,CLSTN1,SHANK2,SV2C,RIMS1,CYFIP1,PLCL2,CRTC1,ADNP,RIMBP2,ZMYND8,SLC24A2,LRRTM2,NPTN,PDE7B,DISC1,PCDH17,PCLO,RNF216,PXK,SYBU,SLC4A10,ARID1B,TSHZ3,NAPB,MCTP1,NETO1,FBXL20,FCHSD1,LYPD1,CNIH3,CNTN4,SYT10 |
| 17_Member | GO Biological Processes | GO:0050804 | modulation of chemical synaptic transmission | -18.84942561 | -16.223 | 97/436 | 18,320,321,375,488,590,627,774,776,783,793,1129,1268,1385,1607,1759,1906,1977,2043,2048,2259,2332,2668,2770,2895,2898,2900,2903,2911,2913,2915,3688,3717,3745,3799,3815,3845,4131,4208,5028,5270,5332,5530,5577,5590,5594,5728,5764,5868,5908,6507,6529,6547,6616,6622,6780,6812,6857,6860,7804,8573,9101,9211,9456,9465,9590,9627,9693,9699,10142,10814,11252,22849,22871,22883,22941,22999,23191,23228,23373,23394,23613,25769,26045,27020,27185,27253,54476,54899,55638,57282,57616,63908,79772,81832,84961,152330 | ABAT,APBA1,APBA2,ARF1,ATP2A2,BCHE,BDNF,CACNA1B,CACNA1D,CACNB2,CALB1,CHRM2,CNR1,CREB1,DGKB,DNM1,EDN1,EIF4E,EPHA4,EPHB2,FGF14,FMR1,GDNF,GNAI1,GRID2,GRIK2,GRIK4,GRIN2A,GRM1,GRM3,GRM5,ITGB1,JAK2,KCNB1,KIF5B,KIT,KRAS,MAP1B,MEF2C,P2RY1,SERPINE2,PLCB4,PPP3CA,PRKAR2B,PRKCZ,MAPK1,PTEN,PTN,RAB5A,RAP1B,SLC1A3,SLC6A1,SLC8A3,SNAP25,SNCA,STAU1,STXBP1,SYT1,SYT4,LRP8,CASK,USP8,LGI1,HOMER1,AKAP7,AKAP12,SNCAIP,RAPGEF2,RIMS2,AKAP9,CPLX2,PACSIN2,CPEB3,NLGN1,CLSTN1,SHANK2,RIMS1,CYFIP1,PLCL2,CRTC1,ADNP,ZMYND8,SLC24A2,LRRTM2,NPTN,DISC1,PCDH17,RNF216,PXK,SYBU,SLC4A10,TSHZ3,NAPB,MCTP1,NETO1,FBXL20,CNTN4 |
| 17_Member | GO Biological Processes | GO:0099177 | regulation of trans-synaptic signaling | -18.77630699 | -16.158 | 97/437 | 18,320,321,375,488,590,627,774,776,783,793,1129,1268,1385,1607,1759,1906,1977,2043,2048,2259,2332,2668,2770,2895,2898,2900,2903,2911,2913,2915,3688,3717,3745,3799,3815,3845,4131,4208,5028,5270,5332,5530,5577,5590,5594,5728,5764,5868,5908,6507,6529,6547,6616,6622,6780,6812,6857,6860,7804,8573,9101,9211,9456,9465,9590,9627,9693,9699,10142,10814,11252,22849,22871,22883,22941,22999,23191,23228,23373,23394,23613,25769,26045,27020,27185,27253,54476,54899,55638,57282,57616,63908,79772,81832,84961,152330 | ABAT,APBA1,APBA2,ARF1,ATP2A2,BCHE,BDNF,CACNA1B,CACNA1D,CACNB2,CALB1,CHRM2,CNR1,CREB1,DGKB,DNM1,EDN1,EIF4E,EPHA4,EPHB2,FGF14,FMR1,GDNF,GNAI1,GRID2,GRIK2,GRIK4,GRIN2A,GRM1,GRM3,GRM5,ITGB1,JAK2,KCNB1,KIF5B,KIT,KRAS,MAP1B,MEF2C,P2RY1,SERPINE2,PLCB4,PPP3CA,PRKAR2B,PRKCZ,MAPK1,PTEN,PTN,RAB5A,RAP1B,SLC1A3,SLC6A1,SLC8A3,SNAP25,SNCA,STAU1,STXBP1,SYT1,SYT4,LRP8,CASK,USP8,LGI1,HOMER1,AKAP7,AKAP12,SNCAIP,RAPGEF2,RIMS2,AKAP9,CPLX2,PACSIN2,CPEB3,NLGN1,CLSTN1,SHANK2,RIMS1,CYFIP1,PLCL2,CRTC1,ADNP,ZMYND8,SLC24A2,LRRTM2,NPTN,DISC1,PCDH17,RNF216,PXK,SYBU,SLC4A10,TSHZ3,NAPB,MCTP1,NETO1,FBXL20,CNTN4 |
| 17_Member | GO Biological Processes | GO:0007610 | behavior | -17.58041531 | -15.022 | 116/596 | 18,27,320,321,372,590,596,627,774,793,1268,1385,1602,1826,1958,1977,2033,2043,2048,2115,2258,2334,2571,2628,2668,2743,2764,2898,2903,2911,2915,3064,3084,3251,3688,3815,3845,4097,4208,4211,4857,4929,5028,5048,5090,5270,5577,5590,5594,5728,5764,6310,6323,6446,6506,6507,6529,6547,6571,6616,6622,6801,6860,7057,7060,7068,7080,7111,7416,7419,7432,8013,8301,8554,8867,9456,9698,10396,10499,10716,10752,22849,22871,22941,23230,23373,23394,25769,27020,27253,53616,54207,54806,55084,55342,55636,55737,55777,57282,57561,57626,64856,65009,81832,84189,84251,84961,89797,93986,116372,130507,153222,221935,285671,338811,342371 | ABAT,ABL2,APBA1,APBA2,ARCN1,BCHE,BCL2,BDNF,CACNA1B,CALB1,CNR1,CREB1,DACH1,DSCAM,EGR1,EIF4E,EP300,EPHA4,EPHB2,ETV1,FGF13,AFF2,GAD1,GATM,GDNF,GLRB,GMFB,GRIK2,GRIN2A,GRM1,GRM5,HTT,NRG1,HPRT1,ITGB1,KIT,KRAS,MAFG,MEF2C,MEIS1,NOVA1,NR4A2,P2RY1,PAFAH1B1,PBX3,SERPINE2,PRKAR2B,PRKCZ,MAPK1,PTEN,PTN,ATXN1,SCN1A,SGK1,SLC1A2,SLC1A3,SLC6A1,SLC8A3,SLC18A2,SNAP25,SNCA,STRN,SYT4,THBS1,THBS4,THRB,NKX2-1,TMOD1,VDAC1,VDAC3,VIP,NR4A3,PICALM,PIAS1,SYNJ1,HOMER1,PUM1,ATP8A1,NCOA2,TBR1,CHL1,CPEB3,NLGN1,SHANK2,VPS13A,CRTC1,ADNP,SLC24A2,NPTN,PCDH17,ADAM22,KCNK10,AHI1,SOBP,STRBP,CHD7,VPS35,MBD5,SLC4A10,ARRDC3,KLHL1,VWA1,NDRG4,NETO1,SLITRK6,SGIP1,FBXL20,NAV2,FOXP2,LYPD1,UBR3,CREBRF,SDK1,RNF180,TAFA2,ATXN1L |
| 17_Member | GO Biological Processes | GO:0048167 | regulation of synaptic plasticity | -9.73768939 | -7.792 | 44/187 | 375,793,1385,2043,2048,2259,2332,2895,2898,2903,2915,3745,3815,3845,4131,4208,5270,5590,5594,5728,5764,5868,6547,6616,6622,6780,6812,6860,9693,9699,10814,22849,22871,22941,22999,23373,25769,26045,27020,57282,57616,79772,81832,152330 | ARF1,CALB1,CREB1,EPHA4,EPHB2,FGF14,FMR1,GRID2,GRIK2,GRIN2A,GRM5,KCNB1,KIT,KRAS,MAP1B,MEF2C,SERPINE2,PRKCZ,MAPK1,PTEN,PTN,RAB5A,SLC8A3,SNAP25,SNCA,STAU1,STXBP1,SYT4,RAPGEF2,RIMS2,CPLX2,CPEB3,NLGN1,SHANK2,RIMS1,CRTC1,SLC24A2,LRRTM2,NPTN,SLC4A10,TSHZ3,MCTP1,NETO1,CNTN4 |
| 17_Member | GO Biological Processes | GO:0007611 | learning or memory | -7.991285666 | -6.191 | 50/256 | 590,627,793,1268,1385,2033,2048,2258,2334,2628,2764,2903,2915,3064,3688,3815,3845,4208,5048,5577,5590,5594,5728,5764,6310,6446,6529,6547,6616,6860,7416,7419,7432,8301,8554,8867,10396,10716,22849,22941,23373,23394,25769,27020,54207,65009,81832,93986,338811,342371 | BCHE,BDNF,CALB1,CNR1,CREB1,EP300,EPHB2,FGF13,AFF2,GATM,GMFB,GRIN2A,GRM5,HTT,ITGB1,KIT,KRAS,MEF2C,PAFAH1B1,PRKAR2B,PRKCZ,MAPK1,PTEN,PTN,ATXN1,SGK1,SLC6A1,SLC8A3,SNAP25,SYT4,VDAC1,VDAC3,VIP,PICALM,PIAS1,SYNJ1,ATP8A1,TBR1,CPEB3,SHANK2,CRTC1,ADNP,SLC24A2,NPTN,KCNK10,NDRG4,NETO1,FOXP2,TAFA2,ATXN1L |
| 17_Member | GO Biological Processes | GO:0050890 | cognition | -7.506930778 | -5.755 | 54/296 | 590,627,793,1268,1385,2033,2048,2258,2334,2628,2764,2903,2915,3064,3688,3815,3845,4208,5048,5577,5590,5594,5728,5764,6310,6446,6529,6547,6616,6860,7416,7419,7432,8301,8554,8867,10396,10716,10752,22849,22941,23191,23373,23394,25769,27020,54207,55084,55636,65009,81832,93986,338811,342371 | BCHE,BDNF,CALB1,CNR1,CREB1,EP300,EPHB2,FGF13,AFF2,GATM,GMFB,GRIN2A,GRM5,HTT,ITGB1,KIT,KRAS,MEF2C,PAFAH1B1,PRKAR2B,PRKCZ,MAPK1,PTEN,PTN,ATXN1,SGK1,SLC6A1,SLC8A3,SNAP25,SYT4,VDAC1,VDAC3,VIP,PICALM,PIAS1,SYNJ1,ATP8A1,TBR1,CHL1,CPEB3,SHANK2,CYFIP1,CRTC1,ADNP,SLC24A2,NPTN,KCNK10,SOBP,CHD7,NDRG4,NETO1,FOXP2,TAFA2,ATXN1L |
| 17_Member | GO Biological Processes | GO:0050806 | positive regulation of synaptic transmission | -6.981496056 | -5.277 | 36/168 | 18,776,783,793,1268,1385,2043,2048,2332,2898,2903,3799,5270,5590,5594,5728,5764,6507,6547,6616,6622,6780,6812,6857,9211,9699,22871,22883,22941,22999,23373,25769,26045,27020,57616,81832 | ABAT,CACNA1D,CACNB2,CALB1,CNR1,CREB1,EPHA4,EPHB2,FMR1,GRIK2,GRIN2A,KIF5B,SERPINE2,PRKCZ,MAPK1,PTEN,PTN,SLC1A3,SLC8A3,SNAP25,SNCA,STAU1,STXBP1,SYT1,LGI1,RIMS2,NLGN1,CLSTN1,SHANK2,RIMS1,CRTC1,SLC24A2,LRRTM2,NPTN,TSHZ3,NETO1 |
| 17_Member | GO Biological Processes | GO:0007612 | learning | -6.084226999 | -4.479 | 31/145 | 590,1385,2048,2258,2764,2903,2915,3064,3688,3815,3845,5577,5764,6310,6529,6547,6616,7416,7419,8554,8867,10396,10716,22941,25769,27020,65009,81832,93986,338811,342371 | BCHE,CREB1,EPHB2,FGF13,GMFB,GRIN2A,GRM5,HTT,ITGB1,KIT,KRAS,PRKAR2B,PTN,ATXN1,SLC6A1,SLC8A3,SNAP25,VDAC1,VDAC3,PIAS1,SYNJ1,ATP8A1,TBR1,SHANK2,SLC24A2,NPTN,NDRG4,NETO1,FOXP2,TAFA2,ATXN1L |
| 17_Member | GO Biological Processes | GO:0060291 | long-term synaptic potentiation | -5.27169513 | -3.777 | 21/86 | 793,1385,2043,2048,2903,5270,5590,5594,5728,5764,6547,6616,6622,6780,22871,22941,23373,25769,26045,27020,57616 | CALB1,CREB1,EPHA4,EPHB2,GRIN2A,SERPINE2,PRKCZ,MAPK1,PTEN,PTN,SLC8A3,SNAP25,SNCA,STAU1,NLGN1,SHANK2,CRTC1,SLC24A2,LRRTM2,NPTN,TSHZ3 |
| 17_Member | GO Biological Processes | GO:0007613 | memory | -3.219587869 | -2.028 | 21/117 | 627,793,1268,1385,2258,2903,5590,5728,5764,6310,6446,6547,6860,22849,23373,23394,25769,54207,81832,338811,342371 | BDNF,CALB1,CNR1,CREB1,FGF13,GRIN2A,PRKCZ,PTEN,PTN,ATXN1,SGK1,SLC8A3,SYT4,CPEB3,CRTC1,ADNP,SLC24A2,KCNK10,NETO1,TAFA2,ATXN1L |
| 18_Summary | GO Molecular Functions | GO:0019901 | protein kinase binding | -19.69352048 | -17.015 | 128/652 | 59,60,90,102,324,390,636,659,858,868,894,998,1027,1213,1488,1499,1540,1641,1739,1759,1826,1832,1994,2043,2181,2886,2908,2915,3084,3717,3784,3799,4082,4214,4659,4745,4753,5062,5066,5080,5170,5295,5573,5576,5577,5580,5590,5594,5597,5728,5764,5770,5784,5796,5801,5879,6009,6331,6416,6427,6430,6497,6558,6812,6885,6929,6934,6996,7048,7186,7187,7402,7416,7430,7534,7855,8013,8618,8621,8660,8717,8812,8829,8850,8874,9043,9465,9541,9555,9655,9732,9734,9794,9821,9892,10019,10140,10221,10716,10818,10982,11124,11221,11235,22841,22919,23162,23328,26060,26960,27347,28951,51422,54206,54619,55704,57381,57605,58480,79616,79866,143384,150094,153090,161742,219771,253260,285489,694,1740,1741,3064,4613,5151,5525,29979,55841,57630 | ACTA2,ACTB,ACVR1,ADAM10,APC,RND3,BICD1,BMPR2,CAV2,CBLB,CCND2,CDC42,CDKN1B,CLTC,CTBP2,CTNNB1,CYLD,DCX,DLG1,DNM1,DSCAM,DSP,ELAVL1,EPHA4,ACSL3,GRB7,NR3C1,GRM5,NRG1,JAK2,KCNQ1,KIF5B,MARCKS,MAP3K1,PPP1R12A,NELL1,NELL2,PAK2,PAM,PAX6,PDPK1,PIK3R1,PRKAR1A,PRKAR2A,PRKAR2B,PRKCD,PRKCZ,MAPK1,MAPK6,PTEN,PTN,PTPN1,PTPN14,PTPRK,PTPRR,RAC1,RHEB,SCN5A,MAP2K4,SRSF2,SRSF5,SKI,SLC12A2,STXBP1,MAP3K7,TCF3,TCF7L2,TDG,TGFBR2,TRAF2,TRAF3,UTRN,VDAC1,EZR,YWHAZ,FZD5,NR4A3,CADPS,CDK13,IRS2,TRADD,CCNK,NRP1,KAT2B,ARHGEF7,SPAG9,AKAP7,CIR1,MACROH2A1,SOCS5,DOCK4,HDAC9,MAML1,RB1CC1,SNAP91,SH2B3,TOB1,TRIB1,TBR1,FRS2,MAPRE2,FAF1,DUSP10,PDCD10,RAB11FIP2,MAPRE1,MAPK8IP3,SASH1,APPL1,NBEA,STK39,TRIB2,PRKAG2,ERRFI1,CCNJ,CCDC88A,RHOJ,PITPNM2,RHOU,CCNJL,BORA,CACUL1,SIK1,DAB2IP,SPRED1,CCNY,RICTOR,DOK7,BTG1,DLG2,DLG3,HTT,MYCN,PDE8A,PPP2R5A,UBQLN1,WWC3,SH3RF1 |
| 18_Member | GO Molecular Functions | GO:0019901 | protein kinase binding | -19.69352048 | -17.015 | 128/652 | 59,60,90,102,324,390,636,659,858,868,894,998,1027,1213,1488,1499,1540,1641,1739,1759,1826,1832,1994,2043,2181,2886,2908,2915,3084,3717,3784,3799,4082,4214,4659,4745,4753,5062,5066,5080,5170,5295,5573,5576,5577,5580,5590,5594,5597,5728,5764,5770,5784,5796,5801,5879,6009,6331,6416,6427,6430,6497,6558,6812,6885,6929,6934,6996,7048,7186,7187,7402,7416,7430,7534,7855,8013,8618,8621,8660,8717,8812,8829,8850,8874,9043,9465,9541,9555,9655,9732,9734,9794,9821,9892,10019,10140,10221,10716,10818,10982,11124,11221,11235,22841,22919,23162,23328,26060,26960,27347,28951,51422,54206,54619,55704,57381,57605,58480,79616,79866,143384,150094,153090,161742,219771,253260,285489 | ACTA2,ACTB,ACVR1,ADAM10,APC,RND3,BICD1,BMPR2,CAV2,CBLB,CCND2,CDC42,CDKN1B,CLTC,CTBP2,CTNNB1,CYLD,DCX,DLG1,DNM1,DSCAM,DSP,ELAVL1,EPHA4,ACSL3,GRB7,NR3C1,GRM5,NRG1,JAK2,KCNQ1,KIF5B,MARCKS,MAP3K1,PPP1R12A,NELL1,NELL2,PAK2,PAM,PAX6,PDPK1,PIK3R1,PRKAR1A,PRKAR2A,PRKAR2B,PRKCD,PRKCZ,MAPK1,MAPK6,PTEN,PTN,PTPN1,PTPN14,PTPRK,PTPRR,RAC1,RHEB,SCN5A,MAP2K4,SRSF2,SRSF5,SKI,SLC12A2,STXBP1,MAP3K7,TCF3,TCF7L2,TDG,TGFBR2,TRAF2,TRAF3,UTRN,VDAC1,EZR,YWHAZ,FZD5,NR4A3,CADPS,CDK13,IRS2,TRADD,CCNK,NRP1,KAT2B,ARHGEF7,SPAG9,AKAP7,CIR1,MACROH2A1,SOCS5,DOCK4,HDAC9,MAML1,RB1CC1,SNAP91,SH2B3,TOB1,TRIB1,TBR1,FRS2,MAPRE2,FAF1,DUSP10,PDCD10,RAB11FIP2,MAPRE1,MAPK8IP3,SASH1,APPL1,NBEA,STK39,TRIB2,PRKAG2,ERRFI1,CCNJ,CCDC88A,RHOJ,PITPNM2,RHOU,CCNJL,BORA,CACUL1,SIK1,DAB2IP,SPRED1,CCNY,RICTOR,DOK7 |
| 18_Member | GO Molecular Functions | GO:0019900 | kinase binding | -19.60894629 | -16.940 | 138/731 | 59,60,90,102,324,390,636,659,694,858,868,894,998,1027,1213,1488,1499,1540,1641,1739,1740,1741,1759,1826,1832,1994,2043,2181,2886,2908,2915,3064,3084,3717,3784,3799,4082,4214,4613,4659,4745,4753,5062,5066,5080,5151,5170,5295,5525,5573,5576,5577,5580,5590,5594,5597,5728,5764,5770,5784,5796,5801,5879,6009,6331,6416,6427,6430,6497,6558,6812,6885,6929,6934,6996,7048,7186,7187,7402,7416,7430,7534,7855,8013,8618,8621,8660,8717,8812,8829,8850,8874,9043,9465,9541,9555,9655,9732,9734,9794,9821,9892,10019,10140,10221,10716,10818,10982,11124,11221,11235,22841,22919,23162,23328,26060,26960,27347,28951,29979,51422,54206,54619,55704,55841,57381,57605,57630,58480,79616,79866,143384,150094,153090,161742,219771,253260,285489 | ACTA2,ACTB,ACVR1,ADAM10,APC,RND3,BICD1,BMPR2,BTG1,CAV2,CBLB,CCND2,CDC42,CDKN1B,CLTC,CTBP2,CTNNB1,CYLD,DCX,DLG1,DLG2,DLG3,DNM1,DSCAM,DSP,ELAVL1,EPHA4,ACSL3,GRB7,NR3C1,GRM5,HTT,NRG1,JAK2,KCNQ1,KIF5B,MARCKS,MAP3K1,MYCN,PPP1R12A,NELL1,NELL2,PAK2,PAM,PAX6,PDE8A,PDPK1,PIK3R1,PPP2R5A,PRKAR1A,PRKAR2A,PRKAR2B,PRKCD,PRKCZ,MAPK1,MAPK6,PTEN,PTN,PTPN1,PTPN14,PTPRK,PTPRR,RAC1,RHEB,SCN5A,MAP2K4,SRSF2,SRSF5,SKI,SLC12A2,STXBP1,MAP3K7,TCF3,TCF7L2,TDG,TGFBR2,TRAF2,TRAF3,UTRN,VDAC1,EZR,YWHAZ,FZD5,NR4A3,CADPS,CDK13,IRS2,TRADD,CCNK,NRP1,KAT2B,ARHGEF7,SPAG9,AKAP7,CIR1,MACROH2A1,SOCS5,DOCK4,HDAC9,MAML1,RB1CC1,SNAP91,SH2B3,TOB1,TRIB1,TBR1,FRS2,MAPRE2,FAF1,DUSP10,PDCD10,RAB11FIP2,MAPRE1,MAPK8IP3,SASH1,APPL1,NBEA,STK39,TRIB2,UBQLN1,PRKAG2,ERRFI1,CCNJ,CCDC88A,WWC3,RHOJ,PITPNM2,SH3RF1,RHOU,CCNJL,BORA,CACUL1,SIK1,DAB2IP,SPRED1,CCNY,RICTOR,DOK7 |
| 19_Summary | GO Biological Processes | GO:0048598 | embryonic morphogenesis | -19.55954822 | -16.899 | 119/586 | 90,92,133,367,657,659,775,1028,1290,1301,1303,1387,1499,1739,1749,1750,1906,2048,2138,2201,2255,2263,2294,2295,2296,2487,2624,2668,2736,2737,2768,3202,3204,3205,3217,3239,3624,3638,3688,3714,3899,4036,4040,4154,4208,4487,4760,5080,5156,5567,5573,5594,5629,5727,5898,5915,6045,6093,6299,6497,6670,6913,6935,7020,7042,7046,7048,7358,7403,7855,8013,8289,8324,8434,8452,8543,8854,9314,9355,9475,9839,10194,10265,10395,10804,10818,10959,11019,23122,23168,23213,23314,23767,26018,26146,28514,29072,51141,51441,51804,54806,54928,55084,55578,55636,57453,57534,60436,65009,79577,84189,84678,121227,125488,130497,145258,157506,204851,100131390,1046,2186,3516,3815,5125,5887,10019,23414,55773,56034,57448,57599,79733,144455,102,357,596,650,780,793,1027,2043,3784,4094,4916,4919,5048,8549,9474,9497,9547,79633 | ACVR1,ACVR2A,ADM,AR,BMPR1A,BMPR2,CACNA1C,CDKN1C,COL5A2,COL11A1,COL12A1,CREBBP,CTNNB1,DLG1,DLX5,DLX6,EDN1,EPHB2,EYA1,FBN2,FGF10,FGFR2,FOXF1,FOXF2,FOXC1,FRZB,GATA2,GDNF,GLI2,GLI3,GNA12,HOXA5,HOXA7,HOXA9,HOXB7,HOXD13,INHBA,INSIG1,ITGB1,JAG2,AFF3,LRP2,LRP6,MBNL1,MEF2C,MSX1,NEUROD1,PAX6,PDGFRA,PRKACB,PRKAR1A,MAPK1,PROX1,PTCH1,RALA,RARB,RNF2,ROCK1,SALL1,SKI,SP3,TBX15,ZEB1,TFAP2A,TGFB2,TGFBR1,TGFBR2,UGDH,KDM6A,FZD5,NR4A3,ARID1A,FZD7,RECK,CUL3,LMO4,ALDH1A2,KLF4,LHX2,ROCK2,ZEB2,TSHZ1,IRX5,DLC1,GJB6,FRS2,TMED2,LIAS,CLASP2,RTF1,SULF1,SATB2,FLRT3,LRIG1,TRAF3IP1,DLL1,SETD2,INSIG2,YTHDF2,SIX4,AHI1,IMPAD1,SOBP,SUPT20H,CHD7,DSCAML1,MIB1,TGIF2,NDRG4,CDC73,SLITRK6,KDM2B,LRIG3,TTC39C,OSR1,GSC,RDH10,HIPK1,SP9,CDX4,BPTF,RBPJ,KIT,PCSK5,RAD23B,SH2B3,ZFPM2,TBC1D23,PDGFC,BIRC6,WDR48,E2F8,E2F7,ADAM10,SHROOM2,BCL2,BMP2,DDR1,CALB1,CDKN1B,EPHA4,KCNQ1,MAF,NTRK3,ROR1,PAFAH1B1,LGR5,ATG5,SLC4A7,CXCL14,FAT4 |
| 19_Member | GO Biological Processes | GO:0048598 | embryonic morphogenesis | -19.55954822 | -16.899 | 119/586 | 90,92,133,367,657,659,775,1028,1290,1301,1303,1387,1499,1739,1749,1750,1906,2048,2138,2201,2255,2263,2294,2295,2296,2487,2624,2668,2736,2737,2768,3202,3204,3205,3217,3239,3624,3638,3688,3714,3899,4036,4040,4154,4208,4487,4760,5080,5156,5567,5573,5594,5629,5727,5898,5915,6045,6093,6299,6497,6670,6913,6935,7020,7042,7046,7048,7358,7403,7855,8013,8289,8324,8434,8452,8543,8854,9314,9355,9475,9839,10194,10265,10395,10804,10818,10959,11019,23122,23168,23213,23314,23767,26018,26146,28514,29072,51141,51441,51804,54806,54928,55084,55578,55636,57453,57534,60436,65009,79577,84189,84678,121227,125488,130497,145258,157506,204851,100131390 | ACVR1,ACVR2A,ADM,AR,BMPR1A,BMPR2,CACNA1C,CDKN1C,COL5A2,COL11A1,COL12A1,CREBBP,CTNNB1,DLG1,DLX5,DLX6,EDN1,EPHB2,EYA1,FBN2,FGF10,FGFR2,FOXF1,FOXF2,FOXC1,FRZB,GATA2,GDNF,GLI2,GLI3,GNA12,HOXA5,HOXA7,HOXA9,HOXB7,HOXD13,INHBA,INSIG1,ITGB1,JAG2,AFF3,LRP2,LRP6,MBNL1,MEF2C,MSX1,NEUROD1,PAX6,PDGFRA,PRKACB,PRKAR1A,MAPK1,PROX1,PTCH1,RALA,RARB,RNF2,ROCK1,SALL1,SKI,SP3,TBX15,ZEB1,TFAP2A,TGFB2,TGFBR1,TGFBR2,UGDH,KDM6A,FZD5,NR4A3,ARID1A,FZD7,RECK,CUL3,LMO4,ALDH1A2,KLF4,LHX2,ROCK2,ZEB2,TSHZ1,IRX5,DLC1,GJB6,FRS2,TMED2,LIAS,CLASP2,RTF1,SULF1,SATB2,FLRT3,LRIG1,TRAF3IP1,DLL1,SETD2,INSIG2,YTHDF2,SIX4,AHI1,IMPAD1,SOBP,SUPT20H,CHD7,DSCAML1,MIB1,TGIF2,NDRG4,CDC73,SLITRK6,KDM2B,LRIG3,TTC39C,OSR1,GSC,RDH10,HIPK1,SP9 |
| 19_Member | GO Biological Processes | GO:0048568 | embryonic organ development | -16.31231046 | -13.848 | 91/429 | 90,133,657,1028,1046,1290,1301,1499,1739,1749,1750,1906,2048,2138,2186,2201,2255,2263,2294,2295,2296,2487,2624,2668,2736,2737,3202,3204,3217,3516,3638,3815,4040,4208,4487,4760,5080,5125,5156,5594,5629,5727,5887,5915,6299,6670,6913,6935,7020,7042,7046,7048,7403,7855,8013,8289,8854,10019,10194,10265,10804,10818,10959,23314,23414,26018,26146,28514,29072,51141,51804,54806,55084,55636,55773,56034,57448,57453,57534,57599,65009,79733,84189,84678,121227,125488,130497,144455,145258,157506,204851 | ACVR1,ADM,BMPR1A,CDKN1C,CDX4,COL5A2,COL11A1,CTNNB1,DLG1,DLX5,DLX6,EDN1,EPHB2,EYA1,BPTF,FBN2,FGF10,FGFR2,FOXF1,FOXF2,FOXC1,FRZB,GATA2,GDNF,GLI2,GLI3,HOXA5,HOXA7,HOXB7,RBPJ,INSIG1,KIT,LRP6,MEF2C,MSX1,NEUROD1,PAX6,PCSK5,PDGFRA,MAPK1,PROX1,PTCH1,RAD23B,RARB,SALL1,SP3,TBX15,ZEB1,TFAP2A,TGFB2,TGFBR1,TGFBR2,KDM6A,FZD5,NR4A3,ARID1A,ALDH1A2,SH2B3,TSHZ1,IRX5,GJB6,FRS2,TMED2,SATB2,ZFPM2,LRIG1,TRAF3IP1,DLL1,SETD2,INSIG2,SIX4,AHI1,SOBP,CHD7,TBC1D23,PDGFC,BIRC6,DSCAML1,MIB1,WDR48,NDRG4,E2F8,SLITRK6,KDM2B,LRIG3,TTC39C,OSR1,E2F7,GSC,RDH10,HIPK1 |
| 19_Member | GO Biological Processes | GO:0043583 | ear development | -14.43180245 | -12.134 | 57/219 | 102,357,596,650,780,793,1027,1301,1749,1750,1906,2043,2048,2138,2255,2263,2487,2624,2736,2737,3516,3638,3714,3784,4094,4487,4760,4916,4919,5048,5594,5629,6299,6935,7020,7042,8013,8549,9474,9497,9547,10194,10804,26018,28514,51141,51804,54806,55084,55636,79633,84189,121227,125488,130497,145258,157506 | ADAM10,SHROOM2,BCL2,BMP2,DDR1,CALB1,CDKN1B,COL11A1,DLX5,DLX6,EDN1,EPHA4,EPHB2,EYA1,FGF10,FGFR2,FRZB,GATA2,GLI2,GLI3,RBPJ,INSIG1,JAG2,KCNQ1,MAF,MSX1,NEUROD1,NTRK3,ROR1,PAFAH1B1,MAPK1,PROX1,SALL1,ZEB1,TFAP2A,TGFB2,NR4A3,LGR5,ATG5,SLC4A7,CXCL14,TSHZ1,GJB6,LRIG1,DLL1,INSIG2,SIX4,AHI1,SOBP,CHD7,FAT4,SLITRK6,LRIG3,TTC39C,OSR1,GSC,RDH10 |
| 19_Member | GO Biological Processes | GO:0048562 | embryonic organ morphogenesis | -14.20495383 | -11.926 | 67/288 | 90,1301,1499,1739,1749,1750,1906,2048,2138,2201,2255,2263,2294,2295,2487,2624,2736,2737,3202,3204,3217,3638,4040,4208,4487,4760,5080,5156,5594,5629,5915,6299,6670,6913,6935,7020,7046,7048,7403,7855,8013,8289,10194,10265,10804,10818,10959,23314,26018,28514,29072,51141,51804,54806,55084,55636,57453,57534,65009,84189,84678,121227,125488,130497,145258,157506,204851 | ACVR1,COL11A1,CTNNB1,DLG1,DLX5,DLX6,EDN1,EPHB2,EYA1,FBN2,FGF10,FGFR2,FOXF1,FOXF2,FRZB,GATA2,GLI2,GLI3,HOXA5,HOXA7,HOXB7,INSIG1,LRP6,MEF2C,MSX1,NEUROD1,PAX6,PDGFRA,MAPK1,PROX1,RARB,SALL1,SP3,TBX15,ZEB1,TFAP2A,TGFBR1,TGFBR2,KDM6A,FZD5,NR4A3,ARID1A,TSHZ1,IRX5,GJB6,FRS2,TMED2,SATB2,LRIG1,DLL1,SETD2,INSIG2,SIX4,AHI1,SOBP,CHD7,DSCAML1,MIB1,NDRG4,SLITRK6,KDM2B,LRIG3,TTC39C,OSR1,GSC,RDH10,HIPK1 |
| 19_Member | GO Biological Processes | GO:0048839 | inner ear development | -10.43308244 | -8.423 | 46/192 | 102,650,793,1027,1301,1749,1750,2043,2048,2138,2255,2263,2487,2624,2736,2737,3516,3638,3714,3784,4094,4760,4916,4919,5048,5629,6935,7020,7042,8013,8549,9474,9497,9547,10804,26018,28514,51141,51804,54806,55084,55636,79633,84189,121227,125488 | ADAM10,BMP2,CALB1,CDKN1B,COL11A1,DLX5,DLX6,EPHA4,EPHB2,EYA1,FGF10,FGFR2,FRZB,GATA2,GLI2,GLI3,RBPJ,INSIG1,JAG2,KCNQ1,MAF,NEUROD1,NTRK3,ROR1,PAFAH1B1,PROX1,ZEB1,TFAP2A,TGFB2,NR4A3,LGR5,ATG5,SLC4A7,CXCL14,GJB6,LRIG1,DLL1,INSIG2,SIX4,AHI1,SOBP,CHD7,FAT4,SLITRK6,LRIG3,TTC39C |
| 19_Member | GO Biological Processes | GO:0042471 | ear morphogenesis | -8.275932182 | -6.457 | 31/118 | 1301,1749,1750,1906,2048,2138,2255,2263,2487,2624,2736,3638,4487,5594,5629,6299,6935,7020,8013,10194,10804,26018,51141,51804,55084,55636,84189,121227,125488,130497,145258 | COL11A1,DLX5,DLX6,EDN1,EPHB2,EYA1,FGF10,FGFR2,FRZB,GATA2,GLI2,INSIG1,MSX1,MAPK1,PROX1,SALL1,ZEB1,TFAP2A,NR4A3,TSHZ1,GJB6,LRIG1,INSIG2,SIX4,SOBP,CHD7,SLITRK6,LRIG3,TTC39C,OSR1,GSC |
| 19_Member | GO Biological Processes | GO:0042472 | inner ear morphogenesis | -5.396287741 | -3.888 | 23/98 | 1301,1749,1750,2048,2138,2255,2263,2487,2624,2736,3638,5629,6935,7020,8013,26018,51141,51804,55084,55636,84189,121227,125488 | COL11A1,DLX5,DLX6,EPHB2,EYA1,FGF10,FGFR2,FRZB,GATA2,GLI2,INSIG1,PROX1,ZEB1,TFAP2A,NR4A3,LRIG1,INSIG2,SIX4,SOBP,CHD7,SLITRK6,LRIG3,TTC39C |
| 20_Summary | GO Cellular Components | GO:1990234 | transferase complex | -19.15523263 | -16.512 | 143/779 | 18,60,90,92,894,1027,1387,1810,2033,2145,2957,2959,3720,4008,5218,5286,5291,5295,5431,5563,5573,5576,5929,5978,5980,6045,6400,6500,6873,6874,6875,6883,6885,6908,7046,7186,7320,7321,7322,7323,7329,7332,7334,7335,7336,7403,7994,8065,8110,8139,8411,8445,8452,8503,8507,8621,8726,8812,8816,8850,9306,9443,9474,9519,9655,9734,9781,9821,10048,10238,10336,10393,10444,10580,11065,11143,22976,23001,23030,23032,23085,23142,23168,23269,23291,23304,23512,23774,25820,25879,25962,26009,26122,26224,27252,27430,29915,51125,51230,51422,54165,54467,54619,54880,55031,55167,55209,55294,55304,55578,55689,55832,55958,57332,57688,58490,58508,64750,64839,79269,79577,79616,80012,80018,80155,80218,80311,80314,84289,84333,84661,84961,114907,121536,130507,150094,152485,171546,197131,201595,219771,339745,554251,102,1462,2266,3895,3915,4052,5034,5711,5868,6383,7184,8533,8614,9318,11320,23198,26043,51138,55175,56929,56975,64708,64856,140460 | ABAT,ACTB,ACVR1,ACVR2A,CCND2,CDKN1B,CREBBP,DR1,EP300,EZH1,GTF2A1,GTF2B,JARID2,LMO7,CDK14,PIK3C2A,PIK3CB,PIK3R1,POLR2B,PRKAA2,PRKAR1A,PRKAR2A,RBBP5,REST,REV3L,RNF2,SEL1L,SKP1,TAF2,TAF4,TAF4B,TAF12,MAP3K7,TBP,TGFBR1,TRAF2,UBE2B,UBE2D1,UBE2D2,UBE2D3,UBE2I,UBE2L3,UBE2N,UBE2V1,UBE2V2,KDM6A,KAT6A,CUL5,DPF3,GAN,EEA1,DYRK2,CUL3,PIK3R3,ENC1,CDK13,EED,CCNK,DCAF5,KAT2B,SOCS6,MED7,ATG5,TBPL1,SOCS5,HDAC9,RNF144A,RB1CC1,RANBP9,DCAF7,PCGF3,ANAPC10,ZER1,SORBS1,UBE2C,KAT7,PAXIP1,WDFY3,KDM4B,USP33,ERC1,DCUN1D4,RTF1,MGA,FBXW11,UBR2,SUZ12,BRD1,ARIH1,DCAF13,VIRMA,ZZZ3,EPC2,FBXL3,KLHL20,MAT2B,HCFC2,GOLGA7,PHF20,PRKAG2,DCUN1D1,ANKIB1,CCNJ,BCOR,USP47,MSL2,SETD5,FBXW7,SPTLC3,SUPT20H,YEATS2,CAND1,KLHL9,CBX8,ZSWIM6,RPRD1B,KMT2C,SMURF2,FBXL17,DCAF10,CDC73,CCNJL,PHC3,NAA25,NAA15,NAA50,KLHL15,EPC1,ING5,PCGF5,DPY30,FBXL20,FBXO32,AEBP2,UBR3,SIK1,ZNF827,SPTSSA,UBR1,STT3B,CCNY,SPOPL,FBXO48,ADAM10,VCAN,FGG,KTN1,LAMC1,LTBP1,P4HB,PSMD5,RAB5A,SDC2,HSP90B1,COPS3,STC2,COPS2,MGAT4A,PSME4,UBXN7,COPS4,KLHL11,FEM1C,FAM20C,COPS7B,VWA1,ASB7 |
| 20_Member | GO Cellular Components | GO:1990234 | transferase complex | -19.15523263 | -16.512 | 143/779 | 18,60,90,92,894,1027,1387,1810,2033,2145,2957,2959,3720,4008,5218,5286,5291,5295,5431,5563,5573,5576,5929,5978,5980,6045,6400,6500,6873,6874,6875,6883,6885,6908,7046,7186,7320,7321,7322,7323,7329,7332,7334,7335,7336,7403,7994,8065,8110,8139,8411,8445,8452,8503,8507,8621,8726,8812,8816,8850,9306,9443,9474,9519,9655,9734,9781,9821,10048,10238,10336,10393,10444,10580,11065,11143,22976,23001,23030,23032,23085,23142,23168,23269,23291,23304,23512,23774,25820,25879,25962,26009,26122,26224,27252,27430,29915,51125,51230,51422,54165,54467,54619,54880,55031,55167,55209,55294,55304,55578,55689,55832,55958,57332,57688,58490,58508,64750,64839,79269,79577,79616,80012,80018,80155,80218,80311,80314,84289,84333,84661,84961,114907,121536,130507,150094,152485,171546,197131,201595,219771,339745,554251 | ABAT,ACTB,ACVR1,ACVR2A,CCND2,CDKN1B,CREBBP,DR1,EP300,EZH1,GTF2A1,GTF2B,JARID2,LMO7,CDK14,PIK3C2A,PIK3CB,PIK3R1,POLR2B,PRKAA2,PRKAR1A,PRKAR2A,RBBP5,REST,REV3L,RNF2,SEL1L,SKP1,TAF2,TAF4,TAF4B,TAF12,MAP3K7,TBP,TGFBR1,TRAF2,UBE2B,UBE2D1,UBE2D2,UBE2D3,UBE2I,UBE2L3,UBE2N,UBE2V1,UBE2V2,KDM6A,KAT6A,CUL5,DPF3,GAN,EEA1,DYRK2,CUL3,PIK3R3,ENC1,CDK13,EED,CCNK,DCAF5,KAT2B,SOCS6,MED7,ATG5,TBPL1,SOCS5,HDAC9,RNF144A,RB1CC1,RANBP9,DCAF7,PCGF3,ANAPC10,ZER1,SORBS1,UBE2C,KAT7,PAXIP1,WDFY3,KDM4B,USP33,ERC1,DCUN1D4,RTF1,MGA,FBXW11,UBR2,SUZ12,BRD1,ARIH1,DCAF13,VIRMA,ZZZ3,EPC2,FBXL3,KLHL20,MAT2B,HCFC2,GOLGA7,PHF20,PRKAG2,DCUN1D1,ANKIB1,CCNJ,BCOR,USP47,MSL2,SETD5,FBXW7,SPTLC3,SUPT20H,YEATS2,CAND1,KLHL9,CBX8,ZSWIM6,RPRD1B,KMT2C,SMURF2,FBXL17,DCAF10,CDC73,CCNJL,PHC3,NAA25,NAA15,NAA50,KLHL15,EPC1,ING5,PCGF5,DPY30,FBXL20,FBXO32,AEBP2,UBR3,SIK1,ZNF827,SPTSSA,UBR1,STT3B,CCNY,SPOPL,FBXO48 |
| 20_Member | GO Cellular Components | GO:0000151 | ubiquitin ligase complex | -9.145445972 | -7.247 | 56/282 | 1027,4008,6045,6400,6500,7186,7320,7321,7322,7323,7332,7334,7335,8065,8139,8445,8452,8507,8816,9443,9781,10048,10238,10336,10393,10444,11065,23032,23142,23291,23304,25820,25879,26224,27252,54165,54467,54880,55031,55294,55832,55958,57332,57688,64750,64839,79269,80012,80311,84333,84961,114907,130507,197131,339745,554251 | CDKN1B,LMO7,RNF2,SEL1L,SKP1,TRAF2,UBE2B,UBE2D1,UBE2D2,UBE2D3,UBE2L3,UBE2N,UBE2V1,CUL5,GAN,DYRK2,CUL3,ENC1,DCAF5,MED7,RNF144A,RANBP9,DCAF7,PCGF3,ANAPC10,ZER1,UBE2C,USP33,DCUN1D4,FBXW11,UBR2,ARIH1,DCAF13,FBXL3,KLHL20,DCUN1D1,ANKIB1,BCOR,USP47,FBXW7,CAND1,KLHL9,CBX8,ZSWIM6,SMURF2,FBXL17,DCAF10,PHC3,KLHL15,PCGF5,FBXL20,FBXO32,UBR3,UBR1,SPOPL,FBXO48 |
| 20_Member | GO Cellular Components | GO:0031461 | cullin-RING ubiquitin ligase complex | -3.938707865 | -2.626 | 28/159 | 1027,6500,8065,8139,8452,8507,8816,10238,10393,10444,11065,23291,25820,25879,26224,27252,55031,55294,55832,55958,57688,64839,79269,80311,84961,114907,339745,554251 | CDKN1B,SKP1,CUL5,GAN,CUL3,ENC1,DCAF5,DCAF7,ANAPC10,ZER1,UBE2C,FBXW11,ARIH1,DCAF13,FBXL3,KLHL20,USP47,FBXW7,CAND1,KLHL9,ZSWIM6,FBXL17,DCAF10,KLHL15,FBXL20,FBXO32,SPOPL,FBXO48 |
| 20_Member | GO Biological Processes | GO:0043687 | post-translational protein modification | -2.823298442 | -1.706 | 47/362 | 102,1462,2266,3895,3915,4008,4052,5034,5711,5868,5929,6383,6500,7184,8065,8139,8452,8533,8614,8816,9306,9318,9474,9655,10238,11320,23198,23291,25879,26043,26224,27252,51138,54165,55175,55294,55832,55958,56929,56975,64708,64856,79269,84961,114907,140460,201595 | ADAM10,VCAN,FGG,KTN1,LAMC1,LMO7,LTBP1,P4HB,PSMD5,RAB5A,RBBP5,SDC2,SKP1,HSP90B1,CUL5,GAN,CUL3,COPS3,STC2,DCAF5,SOCS6,COPS2,ATG5,SOCS5,DCAF7,MGAT4A,PSME4,FBXW11,DCAF13,UBXN7,FBXL3,KLHL20,COPS4,DCUN1D1,KLHL11,FBXW7,CAND1,KLHL9,FEM1C,FAM20C,COPS7B,VWA1,DCAF10,FBXL20,FBXO32,ASB7,STT3B |
