# Supplementary material for: MiRNA Profiling in Pectoral Muscle Throughout Pre- to Post-Natal Stages of Chicken Development
Source: Front Genet. 2020 Jun 23;11:570. doi: 10.3389/fgene.2020.00570 (PMC7324647; doi:10.3389/fgene.2020.00570)
Supplement: Supplementary file 1 [file Data_Sheet_1.ZIP › Supplementary Material/table S4 KEGG metascape_result.docx]

| **GroupID** | **Category** | **Term** | **Description** | **LogP** | **Log(q-value)** | **InTerm_InList** | **Genes** | **Symbols** |
| --- | --- | --- | --- | --- | --- | --- | --- | --- |
| 1_Summary | KEGG Pathway | hsa04360 | Axon guidance | -16.51921138 | -13.825 | 53/175 | 659,817,998,1073,1808,2042,2043,2044,2045,2046,2048,2049,2770,3688,3845,3983,3985,4233,4773,4775,5062,5291,5295,5361,5530,5534,5578,5590,5594,5727,5879,5921,6091,6092,6093,7220,7225,8503,8829,9475,9723,9901,10371,10501,10507,10512,55740,56920,57522,80031,81029,137970,285220 | BMPR2,CAMK2D,CDC42,CFL2,DPYSL2,EPHA3,EPHA4,EPHA5,EPHA7,EPHA8,EPHB2,EPHB3,GNAI1,ITGB1,KRAS,ABLIM1,LIMK2,MET,NFATC2,NFATC3,PAK2,PIK3CB,PIK3R1,PLXNA1,PPP3CA,PPP3R1,PRKCA,PRKCZ,MAPK1,PTCH1,RAC1,RASA1,ROBO1,ROBO2,ROCK1,TRPC1,TRPC6,PIK3R3,NRP1,ROCK2,SEMA3E,SRGAP3,SEMA3A,SEMA6B,SEMA4D,SEMA3C,ENAH,SEMA3G,SRGAP1,SEMA6D,WNT5B,UNC5D,EPHA6 |
| 1_Member | KEGG Pathway | hsa04360 | Axon guidance | -16.51921138 | -13.825 | 53/175 | 659,817,998,1073,1808,2042,2043,2044,2045,2046,2048,2049,2770,3688,3845,3983,3985,4233,4773,4775,5062,5291,5295,5361,5530,5534,5578,5590,5594,5727,5879,5921,6091,6092,6093,7220,7225,8503,8829,9475,9723,9901,10371,10501,10507,10512,55740,56920,57522,80031,81029,137970,285220 | BMPR2,CAMK2D,CDC42,CFL2,DPYSL2,EPHA3,EPHA4,EPHA5,EPHA7,EPHA8,EPHB2,EPHB3,GNAI1,ITGB1,KRAS,ABLIM1,LIMK2,MET,NFATC2,NFATC3,PAK2,PIK3CB,PIK3R1,PLXNA1,PPP3CA,PPP3R1,PRKCA,PRKCZ,MAPK1,PTCH1,RAC1,RASA1,ROBO1,ROBO2,ROCK1,TRPC1,TRPC6,PIK3R3,NRP1,ROCK2,SEMA3E,SRGAP3,SEMA3A,SEMA6B,SEMA4D,SEMA3C,ENAH,SEMA3G,SRGAP1,SEMA6D,WNT5B,UNC5D,EPHA6 |
| 2_Summary | KEGG Pathway | hsa04144 | Endocytosis | -13.71819269 | -11.325 | 62/260 | 375,858,868,998,1173,1211,1212,1213,1759,2060,2066,2261,2263,3799,3800,3815,4233,5156,5590,5867,5868,6457,6642,6643,7042,7046,7048,7879,8027,8411,8724,8976,9101,9135,9372,9559,9727,9829,9922,10015,10552,10564,10565,10890,22841,23327,23362,26052,27131,51100,51324,51699,55737,57154,60682,64744,64750,81873,83737,116987,128866,137492 | ARF1,CAV2,CBLB,CDC42,AP2M1,CLTA,CLTB,CLTC,DNM1,EPS15,ERBB4,FGFR3,FGFR2,KIF5B,KIF5C,KIT,MET,PDGFRA,PRKCZ,RAB4A,RAB5A,SH3GL3,SNX1,SNX2,TGFB2,TGFBR1,TGFBR2,RAB7A,STAM,EEA1,SNX3,WASL,USP8,RABEP1,ZFYVE9,VPS26A,RAB11FIP3,DNAJC6,IQSEC1,PDCD6IP,ARPC1A,ARFGEF2,ARFGEF1,RAB10,RAB11FIP2,NEDD4L,PSD3,DNM3,SNX5,SH3GLB1,SPG21,VPS29,VPS35,SMURF1,SMAP1,SMAP2,SMURF2,ARPC5L,ITCH,AGAP1,CHMP4B,VPS37A |
| 2_Member | KEGG Pathway | hsa04144 | Endocytosis | -13.71819269 | -11.325 | 62/260 | 375,858,868,998,1173,1211,1212,1213,1759,2060,2066,2261,2263,3799,3800,3815,4233,5156,5590,5867,5868,6457,6642,6643,7042,7046,7048,7879,8027,8411,8724,8976,9101,9135,9372,9559,9727,9829,9922,10015,10552,10564,10565,10890,22841,23327,23362,26052,27131,51100,51324,51699,55737,57154,60682,64744,64750,81873,83737,116987,128866,137492 | ARF1,CAV2,CBLB,CDC42,AP2M1,CLTA,CLTB,CLTC,DNM1,EPS15,ERBB4,FGFR3,FGFR2,KIF5B,KIF5C,KIT,MET,PDGFRA,PRKCZ,RAB4A,RAB5A,SH3GL3,SNX1,SNX2,TGFB2,TGFBR1,TGFBR2,RAB7A,STAM,EEA1,SNX3,WASL,USP8,RABEP1,ZFYVE9,VPS26A,RAB11FIP3,DNAJC6,IQSEC1,PDCD6IP,ARPC1A,ARFGEF2,ARFGEF1,RAB10,RAB11FIP2,NEDD4L,PSD3,DNM3,SNX5,SH3GLB1,SPG21,VPS29,VPS35,SMURF1,SMAP1,SMAP2,SMURF2,ARPC5L,ITCH,AGAP1,CHMP4B,VPS37A |
| 3_Summary | KEGG Pathway | hsa05200 | Pathways in cancer | -11.45315198 | -9.335 | 76/395 | 108,115,324,367,596,650,862,868,998,1027,1387,1399,1488,1499,1871,2033,2113,2122,2252,2255,2258,2259,2261,2263,2308,2736,2737,2768,2770,2776,2782,3655,3688,3815,3845,3915,4233,4286,5156,5291,5295,5332,5468,5567,5578,5594,5599,5727,5728,5879,5888,5898,5915,6093,6655,6934,7042,7046,7048,7170,7184,7186,7187,7855,8030,8324,8503,9475,10000,10342,10672,10928,26060,54583,64399,81029,60,1268,2903,5028,5587,5590,5908,7010,7057,9223,9693,9732,9771,9863,11069,56034,260425,894,1129,1293,1297,1385,1977,3680,3717,5105,5170,5515,5516,5520,5525,5527,5529,5563,6009,6198,6446,7058,7060,7529,7531,7534,9586,10971,23678,55844,27,5062,5868,5921,5924,8036,9462,23179,29110 | ADCY2,ADCY9,APC,AR,BCL2,BMP2,RUNX1T1,CBLB,CDC42,CDKN1B,CREBBP,CRKL,CTBP2,CTNNB1,E2F3,EP300,ETS1,MECOM,FGF7,FGF10,FGF13,FGF14,FGFR3,FGFR2,FOXO1,GLI2,GLI3,GNA12,GNAI1,GNAQ,GNB1,ITGA6,ITGB1,KIT,KRAS,LAMC1,MET,MITF,PDGFRA,PIK3CB,PIK3R1,PLCB4,PPARG,PRKACB,PRKCA,MAPK1,MAPK8,PTCH1,PTEN,RAC1,RAD51,RALA,RARB,ROCK1,SOS2,TCF7L2,TGFB2,TGFBR1,TGFBR2,TPM3,HSP90B1,TRAF2,TRAF3,FZD5,CCDC6,FZD7,PIK3R3,ROCK2,AKT3,TFG,GNA13,RALBP1,APPL1,EGLN1,HHIP,WNT5B,ACTB,CNR1,GRIN2A,P2RY1,PRKD1,PRKCZ,RAP1B,TEK,THBS1,MAGI1,RAPGEF2,DOCK4,RAPGEF5,MAGI2,RAPGEF4,PDGFC,MAGI3,CCND2,CHRM2,COL6A3,COL9A1,CREB1,EIF4E,ITGA9,JAK2,PCK1,PDPK1,PPP2CA,PPP2CB,PPP2R2A,PPP2R5A,PPP2R5C,PPP2R5E,PRKAA2,RHEB,RPS6KB1,SGK1,THBS2,THBS4,YWHAB,YWHAE,YWHAZ,CREB5,YWHAQ,SGK3,PPP2R2D,ABL2,PAK2,RAB5A,RASA1,RASGRF2,SHOC2,RASAL2,RGL1,TBK1 |
| 3_Member | KEGG Pathway | hsa05200 | Pathways in cancer | -11.45315198 | -9.335 | 76/395 | 108,115,324,367,596,650,862,868,998,1027,1387,1399,1488,1499,1871,2033,2113,2122,2252,2255,2258,2259,2261,2263,2308,2736,2737,2768,2770,2776,2782,3655,3688,3815,3845,3915,4233,4286,5156,5291,5295,5332,5468,5567,5578,5594,5599,5727,5728,5879,5888,5898,5915,6093,6655,6934,7042,7046,7048,7170,7184,7186,7187,7855,8030,8324,8503,9475,10000,10342,10672,10928,26060,54583,64399,81029 | ADCY2,ADCY9,APC,AR,BCL2,BMP2,RUNX1T1,CBLB,CDC42,CDKN1B,CREBBP,CRKL,CTBP2,CTNNB1,E2F3,EP300,ETS1,MECOM,FGF7,FGF10,FGF13,FGF14,FGFR3,FGFR2,FOXO1,GLI2,GLI3,GNA12,GNAI1,GNAQ,GNB1,ITGA6,ITGB1,KIT,KRAS,LAMC1,MET,MITF,PDGFRA,PIK3CB,PIK3R1,PLCB4,PPARG,PRKACB,PRKCA,MAPK1,MAPK8,PTCH1,PTEN,RAC1,RAD51,RALA,RARB,ROCK1,SOS2,TCF7L2,TGFB2,TGFBR1,TGFBR2,TPM3,HSP90B1,TRAF2,TRAF3,FZD5,CCDC6,FZD7,PIK3R3,ROCK2,AKT3,TFG,GNA13,RALBP1,APPL1,EGLN1,HHIP,WNT5B |
| 3_Member | KEGG Pathway | hsa04015 | Rap1 signaling pathway | -8.063724365 | -6.484 | 44/210 | 60,108,115,998,1268,1399,1499,2252,2255,2258,2259,2261,2263,2770,2776,2903,3688,3815,3845,4233,5028,5156,5291,5295,5332,5578,5587,5590,5594,5879,5898,5908,7010,7057,8503,9223,9693,9732,9771,9863,10000,11069,56034,260425 | ACTB,ADCY2,ADCY9,CDC42,CNR1,CRKL,CTNNB1,FGF7,FGF10,FGF13,FGF14,FGFR3,FGFR2,GNAI1,GNAQ,GRIN2A,ITGB1,KIT,KRAS,MET,P2RY1,PDGFRA,PIK3CB,PIK3R1,PLCB4,PRKCA,PRKD1,PRKCZ,MAPK1,RAC1,RALA,RAP1B,TEK,THBS1,PIK3R3,MAGI1,RAPGEF2,DOCK4,RAPGEF5,MAGI2,AKT3,RAPGEF4,PDGFC,MAGI3 |
| 3_Member | KEGG Pathway | hsa04151 | PI3K-Akt signaling pathway | -6.858804309 | -5.545 | 58/342 | 596,894,1027,1129,1293,1297,1385,1977,2252,2255,2258,2259,2261,2263,2782,3655,3680,3688,3717,3815,3845,3915,4233,5105,5156,5170,5291,5295,5515,5516,5520,5525,5527,5529,5563,5578,5594,5728,5879,6009,6198,6446,6655,7010,7057,7058,7060,7184,7529,7531,7534,8503,9586,10000,10971,23678,55844,56034 | BCL2,CCND2,CDKN1B,CHRM2,COL6A3,COL9A1,CREB1,EIF4E,FGF7,FGF10,FGF13,FGF14,FGFR3,FGFR2,GNB1,ITGA6,ITGA9,ITGB1,JAK2,KIT,KRAS,LAMC1,MET,PCK1,PDGFRA,PDPK1,PIK3CB,PIK3R1,PPP2CA,PPP2CB,PPP2R2A,PPP2R5A,PPP2R5C,PPP2R5E,PRKAA2,PRKCA,MAPK1,PTEN,RAC1,RHEB,RPS6KB1,SGK1,SOS2,TEK,THBS1,THBS2,THBS4,HSP90B1,YWHAB,YWHAE,YWHAZ,PIK3R3,CREB5,AKT3,YWHAQ,SGK3,PPP2R2D,PDGFC |
| 3_Member | KEGG Pathway | hsa04014 | Ras signaling pathway | -4.968509845 | -3.938 | 39/227 | 27,998,2113,2252,2255,2258,2259,2261,2263,2782,2903,3815,3845,4233,5062,5156,5291,5295,5567,5578,5594,5599,5868,5879,5898,5908,5921,5924,6655,7010,8036,8503,9462,9771,10000,10928,23179,29110,56034 | ABL2,CDC42,ETS1,FGF7,FGF10,FGF13,FGF14,FGFR3,FGFR2,GNB1,GRIN2A,KIT,KRAS,MET,PAK2,PDGFRA,PIK3CB,PIK3R1,PRKACB,PRKCA,MAPK1,MAPK8,RAB5A,RAC1,RALA,RAP1B,RASA1,RASGRF2,SOS2,TEK,SHOC2,PIK3R3,RASAL2,RAPGEF5,AKT3,RALBP1,RGL1,TBK1,PDGFC |
| 4_Summary | KEGG Pathway | hsa04010 | MAPK signaling pathway | -11.42640799 | -9.335 | 57/255 | 627,774,775,776,781,783,998,1399,1849,1850,2122,2252,2255,2258,2259,2261,2263,2768,3845,3925,4208,4214,4775,5062,5156,5494,5495,5530,5534,5567,5578,5594,5599,5801,5879,5908,5921,5924,6416,6655,6885,7042,7046,7048,7186,8491,9252,9448,9693,10000,11221,23162,27330,51347,51701,57551,80824 | BDNF,CACNA1B,CACNA1C,CACNA1D,CACNA2D1,CACNB2,CDC42,CRKL,DUSP7,DUSP8,MECOM,FGF7,FGF10,FGF13,FGF14,FGFR3,FGFR2,GNA12,KRAS,STMN1,MEF2C,MAP3K1,NFATC3,PAK2,PDGFRA,PPM1A,PPM1B,PPP3CA,PPP3R1,PRKACB,PRKCA,MAPK1,MAPK8,PTPRR,RAC1,RAP1B,RASA1,RASGRF2,MAP2K4,SOS2,MAP3K7,TGFB2,TGFBR1,TGFBR2,TRAF2,MAP4K3,RPS6KA5,MAP4K4,RAPGEF2,AKT3,DUSP10,MAPK8IP3,RPS6KA6,TAOK3,NLK,TAOK1,DUSP16 |
| 4_Member | KEGG Pathway | hsa04010 | MAPK signaling pathway | -11.42640799 | -9.335 | 57/255 | 627,774,775,776,781,783,998,1399,1849,1850,2122,2252,2255,2258,2259,2261,2263,2768,3845,3925,4208,4214,4775,5062,5156,5494,5495,5530,5534,5567,5578,5594,5599,5801,5879,5908,5921,5924,6416,6655,6885,7042,7046,7048,7186,8491,9252,9448,9693,10000,11221,23162,27330,51347,51701,57551,80824 | BDNF,CACNA1B,CACNA1C,CACNA1D,CACNA2D1,CACNB2,CDC42,CRKL,DUSP7,DUSP8,MECOM,FGF7,FGF10,FGF13,FGF14,FGFR3,FGFR2,GNA12,KRAS,STMN1,MEF2C,MAP3K1,NFATC3,PAK2,PDGFRA,PPM1A,PPM1B,PPP3CA,PPP3R1,PRKACB,PRKCA,MAPK1,MAPK8,PTPRR,RAC1,RAP1B,RASA1,RASGRF2,MAP2K4,SOS2,MAP3K7,TGFB2,TGFBR1,TGFBR2,TRAF2,MAP4K3,RPS6KA5,MAP4K4,RAPGEF2,AKT3,DUSP10,MAPK8IP3,RPS6KA6,TAOK3,NLK,TAOK1,DUSP16 |
| 5_Summary | KEGG Pathway | hsa04350 | TGF-beta signaling pathway | -10.9180618 | -8.923 | 29/84 | 90,92,650,657,659,1387,1875,2033,3624,3625,4052,4091,5308,5515,5516,5594,6093,6198,6500,7042,7046,7048,7050,7057,9372,10468,57154,60436,64750 | ACVR1,ACVR2A,BMP2,BMPR1A,BMPR2,CREBBP,E2F5,EP300,INHBA,INHBB,LTBP1,SMAD6,PITX2,PPP2CA,PPP2CB,MAPK1,ROCK1,RPS6KB1,SKP1,TGFB2,TGFBR1,TGFBR2,TGIF1,THBS1,ZFYVE9,FST,SMURF1,TGIF2,SMURF2 |
| 5_Member | KEGG Pathway | hsa04350 | TGF-beta signaling pathway | -10.9180618 | -8.923 | 29/84 | 90,92,650,657,659,1387,1875,2033,3624,3625,4052,4091,5308,5515,5516,5594,6093,6198,6500,7042,7046,7048,7050,7057,9372,10468,57154,60436,64750 | ACVR1,ACVR2A,BMP2,BMPR1A,BMPR2,CREBBP,E2F5,EP300,INHBA,INHBB,LTBP1,SMAD6,PITX2,PPP2CA,PPP2CB,MAPK1,ROCK1,RPS6KB1,SKP1,TGFB2,TGFBR1,TGFBR2,TGIF1,THBS1,ZFYVE9,FST,SMURF1,TGIF2,SMURF2 |
| 6_Summary | KEGG Pathway | hsa04810 | Regulation of actin cytoskeleton | -10.46730599 | -8.552 | 49/212 | 60,87,324,998,1073,1129,1399,1730,2252,2255,2258,2259,2261,2263,2768,3655,3680,3688,3845,3985,4627,4628,4638,4659,5062,5156,5291,5295,5305,5594,5879,6093,6655,7114,7414,7430,8503,8874,8976,9475,10552,10672,10787,10788,23191,50649,55740,56034,81873,858,868,1211,1212,1213,1499,1759,4233,23607,26052,26084,394,596,894,1293,1297,3915,5170,5578,5599,5728,5908,7057,7058,7060,10000,288,817,1655,2066,3708,5567,5727,6198,6383,7042,7855,8324,10818,27250,81029,4691,7100,7534,10971,84617,7322,9474,23291,2770,7412,9080,11069,4082,5580,1781,1783,7879 | ACTB,ACTN1,APC,CDC42,CFL2,CHRM2,CRKL,DIAPH2,FGF7,FGF10,FGF13,FGF14,FGFR3,FGFR2,GNA12,ITGA6,ITGA9,ITGB1,KRAS,LIMK2,MYH9,MYH10,MYLK,PPP1R12A,PAK2,PDGFRA,PIK3CB,PIK3R1,PIP4K2A,MAPK1,RAC1,ROCK1,SOS2,TMSB4X,VCL,EZR,PIK3R3,ARHGEF7,WASL,ROCK2,ARPC1A,GNA13,NCKAP1,IQGAP2,CYFIP1,ARHGEF4,ENAH,PDGFC,ARPC5L,CAV2,CBLB,CLTA,CLTB,CLTC,CTNNB1,DNM1,MET,CD2AP,DNM3,ARHGEF26,ARHGAP5,BCL2,CCND2,COL6A3,COL9A1,LAMC1,PDPK1,PRKCA,MAPK8,PTEN,RAP1B,THBS1,THBS2,THBS4,AKT3,ANK3,CAMK2D,DDX5,ERBB4,ITPR1,PRKACB,PTCH1,RPS6KB1,SDC2,TGFB2,FZD5,FZD7,FRS2,PDCD4,WNT5B,NCL,TLR5,YWHAZ,YWHAQ,TUBB6,UBE2D2,ATG5,FBXW11,GNAI1,VCAM1,CLDN9,RAPGEF4,MARCKS,PRKCD,DYNC1I2,DYNC1LI2,RAB7A |
| 6_Member | KEGG Pathway | hsa04810 | Regulation of actin cytoskeleton | -10.46730599 | -8.552 | 49/212 | 60,87,324,998,1073,1129,1399,1730,2252,2255,2258,2259,2261,2263,2768,3655,3680,3688,3845,3985,4627,4628,4638,4659,5062,5156,5291,5295,5305,5594,5879,6093,6655,7114,7414,7430,8503,8874,8976,9475,10552,10672,10787,10788,23191,50649,55740,56034,81873 | ACTB,ACTN1,APC,CDC42,CFL2,CHRM2,CRKL,DIAPH2,FGF7,FGF10,FGF13,FGF14,FGFR3,FGFR2,GNA12,ITGA6,ITGA9,ITGB1,KRAS,LIMK2,MYH9,MYH10,MYLK,PPP1R12A,PAK2,PDGFRA,PIK3CB,PIK3R1,PIP4K2A,MAPK1,RAC1,ROCK1,SOS2,TMSB4X,VCL,EZR,PIK3R3,ARHGEF7,WASL,ROCK2,ARPC1A,GNA13,NCKAP1,IQGAP2,CYFIP1,ARHGEF4,ENAH,PDGFC,ARPC5L |
| 6_Member | KEGG Pathway | hsa05100 | Bacterial invasion of epithelial cells | -7.555429067 | -6.140 | 23/76 | 60,858,868,998,1211,1212,1213,1399,1499,1759,3688,4233,5291,5295,5879,7414,8503,8976,10552,23607,26052,26084,81873 | ACTB,CAV2,CBLB,CDC42,CLTA,CLTB,CLTC,CRKL,CTNNB1,DNM1,ITGB1,MET,PIK3CB,PIK3R1,RAC1,VCL,PIK3R3,WASL,ARPC1A,CD2AP,DNM3,ARHGEF26,ARPC5L |
| 6_Member | KEGG Pathway | hsa04510 | Focal adhesion | -6.425849072 | -5.163 | 39/199 | 60,87,394,596,858,894,998,1293,1297,1399,1499,3655,3680,3688,3915,4233,4638,4659,5062,5156,5170,5291,5295,5578,5594,5599,5728,5879,5908,6093,6655,7057,7058,7060,7414,8503,9475,10000,56034 | ACTB,ACTN1,ARHGAP5,BCL2,CAV2,CCND2,CDC42,COL6A3,COL9A1,CRKL,CTNNB1,ITGA6,ITGA9,ITGB1,LAMC1,MET,MYLK,PPP1R12A,PAK2,PDGFRA,PDPK1,PIK3CB,PIK3R1,PRKCA,MAPK1,MAPK8,PTEN,RAC1,RAP1B,ROCK1,SOS2,THBS1,THBS2,THBS4,VCL,PIK3R3,ROCK2,AKT3,PDGFC |
| 6_Member | KEGG Pathway | hsa05205 | Proteoglycans in cancer | -5.349898012 | -4.203 | 37/203 | 60,288,817,858,868,998,1499,1655,2066,3688,3708,3845,4233,4659,5170,5291,5295,5567,5578,5594,5727,5879,6093,6198,6383,6655,7042,7057,7430,7855,8324,8503,9475,10000,10818,27250,81029 | ACTB,ANK3,CAMK2D,CAV2,CBLB,CDC42,CTNNB1,DDX5,ERBB4,ITGB1,ITPR1,KRAS,MET,PPP1R12A,PDPK1,PIK3CB,PIK3R1,PRKACB,PRKCA,MAPK1,PTCH1,RAC1,ROCK1,RPS6KB1,SDC2,SOS2,TGFB2,THBS1,EZR,FZD5,FZD7,PIK3R3,ROCK2,AKT3,FRS2,PDCD4,WNT5B |
| 6_Member | KEGG Pathway | hsa05130 | Pathogenic Escherichia coli infection | -5.201426167 | -4.110 | 16/55 | 60,998,1499,3688,4691,5578,6093,7100,7430,7534,8976,9475,10552,10971,81873,84617 | ACTB,CDC42,CTNNB1,ITGB1,NCL,PRKCA,ROCK1,TLR5,EZR,YWHAZ,WASL,ROCK2,ARPC1A,YWHAQ,ARPC5L,TUBB6 |
| 6_Member | KEGG Pathway | hsa05131 | Shigellosis | -4.209050087 | -3.286 | 16/65 | 60,998,1399,3688,5594,5599,5879,6093,7322,7414,8976,9474,9475,10552,23291,81873 | ACTB,CDC42,CRKL,ITGB1,MAPK1,MAPK8,RAC1,ROCK1,UBE2D2,VCL,WASL,ATG5,ROCK2,ARPC1A,FBXW11,ARPC5L |
| 6_Member | KEGG Pathway | hsa04670 | Leukocyte transendothelial migration | -2.966468187 | -2.202 | 20/114 | 60,87,394,998,1499,2770,3688,5291,5295,5578,5879,5908,6093,7412,7414,7430,8503,9080,9475,11069 | ACTB,ACTN1,ARHGAP5,CDC42,CTNNB1,GNAI1,ITGB1,PIK3CB,PIK3R1,PRKCA,RAC1,RAP1B,ROCK1,VCAM1,VCL,EZR,PIK3R3,CLDN9,ROCK2,RAPGEF4 |
| 6_Member | KEGG Pathway | hsa04666 | Fc gamma R-mediated phagocytosis | -2.913819214 | -2.161 | 17/91 | 998,1073,1399,3985,4082,5291,5295,5578,5580,5594,5879,6198,8503,8976,10000,10552,81873 | CDC42,CFL2,CRKL,LIMK2,MARCKS,PIK3CB,PIK3R1,PRKCA,PRKCD,MAPK1,RAC1,RPS6KB1,PIK3R3,WASL,AKT3,ARPC1A,ARPC5L |
| 6_Member | KEGG Pathway | hsa05132 | Salmonella infection | -2.7564063 | -2.031 | 16/86 | 60,998,1781,1783,4627,4628,5594,5599,5879,6093,7100,7879,8976,9475,10552,81873 | ACTB,CDC42,DYNC1I2,DYNC1LI2,MYH9,MYH10,MAPK1,MAPK8,RAC1,ROCK1,TLR5,RAB7A,WASL,ROCK2,ARPC1A,ARPC5L |
| 7_Summary | KEGG Pathway | hsa04120 | Ubiquitin mediated proteolysis | -10.11849767 | -8.270 | 37/137 | 868,3093,4214,4281,6477,6500,7320,7321,7322,7323,7327,7328,7329,7332,7334,7337,8065,8452,8554,8924,8925,9320,9690,10054,10393,10477,11065,23291,23327,55284,55294,55958,57154,57448,64750,83737,92912 | CBLB,UBE2K,MAP3K1,MID1,SIAH1,SKP1,UBE2B,UBE2D1,UBE2D2,UBE2D3,UBE2G2,UBE2H,UBE2I,UBE2L3,UBE2N,UBE3A,CUL5,CUL3,PIAS1,HERC2,HERC1,TRIP12,UBE3C,UBA2,ANAPC10,UBE2E3,UBE2C,FBXW11,NEDD4L,UBE2W,FBXW7,KLHL9,SMURF1,BIRC6,SMURF2,ITCH,UBE2Q2 |
| 7_Member | KEGG Pathway | hsa04120 | Ubiquitin mediated proteolysis | -10.11849767 | -8.270 | 37/137 | 868,3093,4214,4281,6477,6500,7320,7321,7322,7323,7327,7328,7329,7332,7334,7337,8065,8452,8554,8924,8925,9320,9690,10054,10393,10477,11065,23291,23327,55284,55294,55958,57154,57448,64750,83737,92912 | CBLB,UBE2K,MAP3K1,MID1,SIAH1,SKP1,UBE2B,UBE2D1,UBE2D2,UBE2D3,UBE2G2,UBE2H,UBE2I,UBE2L3,UBE2N,UBE3A,CUL5,CUL3,PIAS1,HERC2,HERC1,TRIP12,UBE3C,UBA2,ANAPC10,UBE2E3,UBE2C,FBXW11,NEDD4L,UBE2W,FBXW7,KLHL9,SMURF1,BIRC6,SMURF2,ITCH,UBE2Q2 |
| 8_Summary | KEGG Pathway | hsa04140 | Autophagy - animal | -9.7713084 | -7.981 | 35/128 | 596,664,3708,3845,5170,5291,5295,5515,5516,5563,5567,5580,5594,5599,5728,6009,6198,6885,7879,8503,8660,8897,9342,9474,9706,9821,10000,51100,55054,55578,55626,58476,58528,81671,84938,1385,1994,2308,5105,5209,5468,5520,5525,5527,5529,6319,9586,10890,10891,51422,51552,55844,79966,81617,604,894,1027,1387,2033,2911,6446,6655,7042,7046,7048,7874,9456,23678,51701,80854,114907,108,115,1977,26060,27244,79813,143686,868,1399,5573,5576,5577,5590,5770,5792,10580,27330 | BCL2,BNIP3,ITPR1,KRAS,PDPK1,PIK3CB,PIK3R1,PPP2CA,PPP2CB,PRKAA2,PRKACB,PRKCD,MAPK1,MAPK8,PTEN,RHEB,RPS6KB1,MAP3K7,RAB7A,PIK3R3,IRS2,MTMR3,SNAP29,ATG5,ULK2,RB1CC1,AKT3,SH3GLB1,ATG16L1,SUPT20H,AMBRA1,TP53INP2,RRAGD,VMP1,ATG4C,CREB1,ELAVL1,FOXO1,PCK1,PFKFB3,PPARG,PPP2R2A,PPP2R5A,PPP2R5C,PPP2R5E,SCD,CREB5,RAB10,PPARGC1A,PRKAG2,RAB14,PPP2R2D,SCD5,CAB39L,BCL6,CCND2,CDKN1B,CREBBP,EP300,GRM1,SGK1,SOS2,TGFB2,TGFBR1,TGFBR2,USP7,HOMER1,SGK3,NLK,SETD7,FBXO32,ADCY2,ADCY9,EIF4E,APPL1,SESN1,EHMT1,SESN3,CBLB,CRKL,PRKAR1A,PRKAR2A,PRKAR2B,PRKCZ,PTPN1,PTPRF,SORBS1,RPS6KA6 |
| 8_Member | KEGG Pathway | hsa04140 | Autophagy - animal | -9.7713084 | -7.981 | 35/128 | 596,664,3708,3845,5170,5291,5295,5515,5516,5563,5567,5580,5594,5599,5728,6009,6198,6885,7879,8503,8660,8897,9342,9474,9706,9821,10000,51100,55054,55578,55626,58476,58528,81671,84938 | BCL2,BNIP3,ITPR1,KRAS,PDPK1,PIK3CB,PIK3R1,PPP2CA,PPP2CB,PRKAA2,PRKACB,PRKCD,MAPK1,MAPK8,PTEN,RHEB,RPS6KB1,MAP3K7,RAB7A,PIK3R3,IRS2,MTMR3,SNAP29,ATG5,ULK2,RB1CC1,AKT3,SH3GLB1,ATG16L1,SUPT20H,AMBRA1,TP53INP2,RRAGD,VMP1,ATG4C |
| 8_Member | KEGG Pathway | hsa04152 | AMPK signaling pathway | -8.08641006 | -6.484 | 31/120 | 1385,1994,2308,5105,5170,5209,5291,5295,5468,5515,5516,5520,5525,5527,5529,5563,6009,6198,6319,6885,8503,8660,9586,10000,10890,10891,51422,51552,55844,79966,81617 | CREB1,ELAVL1,FOXO1,PCK1,PDPK1,PFKFB3,PIK3CB,PIK3R1,PPARG,PPP2CA,PPP2CB,PPP2R2A,PPP2R5A,PPP2R5C,PPP2R5E,PRKAA2,RHEB,RPS6KB1,SCD,MAP3K7,PIK3R3,IRS2,CREB5,AKT3,RAB10,PPARGC1A,PRKAG2,RAB14,PPP2R2D,SCD5,CAB39L |
| 8_Member | KEGG Pathway | hsa04068 | FoxO signaling pathway | -7.602792541 | -6.164 | 32/132 | 604,664,894,1027,1387,2033,2308,2911,3845,5105,5170,5291,5295,5563,5594,5599,5728,6446,6655,7042,7046,7048,7874,8503,8660,9456,10000,23678,51422,51701,80854,114907 | BCL6,BNIP3,CCND2,CDKN1B,CREBBP,EP300,FOXO1,GRM1,KRAS,PCK1,PDPK1,PIK3CB,PIK3R1,PRKAA2,MAPK1,MAPK8,PTEN,SGK1,SOS2,TGFB2,TGFBR1,TGFBR2,USP7,PIK3R3,IRS2,HOMER1,AKT3,SGK3,PRKAG2,NLK,SETD7,FBXO32 |
| 8_Member | KEGG Pathway | hsa04211 | Longevity regulating pathway | -7.430738896 | -6.059 | 25/89 | 108,115,1385,1977,2308,3845,5291,5295,5468,5563,5567,6009,6198,8503,8660,9474,9586,9821,10000,10891,26060,27244,51422,79813,143686 | ADCY2,ADCY9,CREB1,EIF4E,FOXO1,KRAS,PIK3CB,PIK3R1,PPARG,PRKAA2,PRKACB,RHEB,RPS6KB1,PIK3R3,IRS2,ATG5,CREB5,RB1CC1,AKT3,PPARGC1A,APPL1,SESN1,PRKAG2,EHMT1,SESN3 |
| 8_Member | KEGG Pathway | hsa04910 | Insulin signaling pathway | -5.099282963 | -4.049 | 28/138 | 868,1399,1977,2308,3845,5105,5170,5291,5295,5563,5567,5573,5576,5577,5590,5594,5599,5770,5792,6009,6198,6655,8503,8660,10000,10580,10891,51422 | CBLB,CRKL,EIF4E,FOXO1,KRAS,PCK1,PDPK1,PIK3CB,PIK3R1,PRKAA2,PRKACB,PRKAR1A,PRKAR2A,PRKAR2B,PRKCZ,MAPK1,MAPK8,PTPN1,PTPRF,RHEB,RPS6KB1,SOS2,PIK3R3,IRS2,AKT3,SORBS1,PPARGC1A,PRKAG2 |
| 8_Member | KEGG Pathway | hsa04931 | Insulin resistance | -3.76960487 | -2.913 | 21/107 | 1385,2308,5105,5170,5291,5295,5563,5580,5590,5599,5728,5770,5792,6198,8503,8660,9586,10000,10891,27330,51422 | CREB1,FOXO1,PCK1,PDPK1,PIK3CB,PIK3R1,PRKAA2,PRKCD,PRKCZ,MAPK8,PTEN,PTPN1,PTPRF,RPS6KB1,PIK3R3,IRS2,CREB5,AKT3,PPARGC1A,RPS6KA6,PRKAG2 |
| 8_Member | KEGG Pathway | hsa04213 | Longevity regulating pathway - multiple species | -3.338509479 | -2.520 | 14/62 | 108,115,2308,3845,5291,5295,5563,5567,6198,8503,8660,9474,10000,51422 | ADCY2,ADCY9,FOXO1,KRAS,PIK3CB,PIK3R1,PRKAA2,PRKACB,RPS6KB1,PIK3R3,IRS2,ATG5,AKT3,PRKAG2 |
| 9_Summary | KEGG Pathway | hsa04310 | Wnt signaling pathway | -8.944077295 | -7.205 | 36/143 | 324,817,894,1387,1457,1488,1499,2033,2239,4040,4773,4775,5332,5530,5534,5567,5578,5599,5879,6477,6500,6885,6934,7855,8324,9475,23002,23291,27123,51701,79718,81029,81839,85407,122011,166336 | APC,CAMK2D,CCND2,CREBBP,CSNK2A1,CTBP2,CTNNB1,EP300,GPC4,LRP6,NFATC2,NFATC3,PLCB4,PPP3CA,PPP3R1,PRKACB,PRKCA,MAPK8,RAC1,SIAH1,SKP1,MAP3K7,TCF7L2,FZD5,FZD7,ROCK2,DAAM1,FBXW11,DKK2,NLK,TBL1XR1,WNT5B,VANGL1,NKD1,CSNK1A1L,PRICKLE2 |
| 9_Member | KEGG Pathway | hsa04310 | Wnt signaling pathway | -8.944077295 | -7.205 | 36/143 | 324,817,894,1387,1457,1488,1499,2033,2239,4040,4773,4775,5332,5530,5534,5567,5578,5599,5879,6477,6500,6885,6934,7855,8324,9475,23002,23291,27123,51701,79718,81029,81839,85407,122011,166336 | APC,CAMK2D,CCND2,CREBBP,CSNK2A1,CTBP2,CTNNB1,EP300,GPC4,LRP6,NFATC2,NFATC3,PLCB4,PPP3CA,PPP3R1,PRKACB,PRKCA,MAPK8,RAC1,SIAH1,SKP1,MAP3K7,TCF7L2,FZD5,FZD7,ROCK2,DAAM1,FBXW11,DKK2,NLK,TBL1XR1,WNT5B,VANGL1,NKD1,CSNK1A1L,PRICKLE2 |
| 10_Summary | KEGG Pathway | hsa04071 | Sphingolipid signaling pathway | -8.895272501 | -7.202 | 32/118 | 596,2768,2770,2776,3845,5170,5291,5295,5332,5515,5516,5520,5525,5527,5529,5578,5590,5594,5599,5728,5879,6093,7186,8503,8717,9475,10000,10672,55304,55844,166929,259230,774,775,776,817,1385,2774,2782,2891,2903,3708,3763,3799,3800,5530,5567,6323,6571,9575,9586,108,115,488,781,783,1390,3784,6331,6546,7170,9252,11069,6416,7042,7046,7048,9630 | BCL2,GNA12,GNAI1,GNAQ,KRAS,PDPK1,PIK3CB,PIK3R1,PLCB4,PPP2CA,PPP2CB,PPP2R2A,PPP2R5A,PPP2R5C,PPP2R5E,PRKCA,PRKCZ,MAPK1,MAPK8,PTEN,RAC1,ROCK1,TRAF2,PIK3R3,TRADD,ROCK2,AKT3,GNA13,SPTLC3,PPP2R2D,SGMS2,SGMS1,CACNA1B,CACNA1C,CACNA1D,CAMK2D,CREB1,GNAL,GNB1,GRIA2,GRIN2A,ITPR1,KCNJ6,KIF5B,KIF5C,PPP3CA,PRKACB,SCN1A,SLC18A2,CLOCK,CREB5,ADCY2,ADCY9,ATP2A2,CACNA2D1,CACNB2,CREM,KCNQ1,SCN5A,SLC8A1,TPM3,RPS6KA5,RAPGEF4,MAP2K4,TGFB2,TGFBR1,TGFBR2,GNA14 |
| 10_Member | KEGG Pathway | hsa04071 | Sphingolipid signaling pathway | -8.895272501 | -7.202 | 32/118 | 596,2768,2770,2776,3845,5170,5291,5295,5332,5515,5516,5520,5525,5527,5529,5578,5590,5594,5599,5728,5879,6093,7186,8503,8717,9475,10000,10672,55304,55844,166929,259230 | BCL2,GNA12,GNAI1,GNAQ,KRAS,PDPK1,PIK3CB,PIK3R1,PLCB4,PPP2CA,PPP2CB,PPP2R2A,PPP2R5A,PPP2R5C,PPP2R5E,PRKCA,PRKCZ,MAPK1,MAPK8,PTEN,RAC1,ROCK1,TRAF2,PIK3R3,TRADD,ROCK2,AKT3,GNA13,SPTLC3,PPP2R2D,SGMS2,SGMS1 |
| 10_Member | KEGG Pathway | hsa04728 | Dopaminergic synapse | -7.773659317 | -6.284 | 32/130 | 774,775,776,817,1385,2770,2774,2776,2782,2891,2903,3708,3763,3799,3800,5332,5515,5516,5520,5525,5527,5529,5530,5567,5578,5599,6323,6571,9575,9586,10000,55844 | CACNA1B,CACNA1C,CACNA1D,CAMK2D,CREB1,GNAI1,GNAL,GNAQ,GNB1,GRIA2,GRIN2A,ITPR1,KCNJ6,KIF5B,KIF5C,PLCB4,PPP2CA,PPP2CB,PPP2R2A,PPP2R5A,PPP2R5C,PPP2R5E,PPP3CA,PRKACB,PRKCA,MAPK8,SCN1A,SLC18A2,CLOCK,CREB5,AKT3,PPP2R2D |
| 10_Member | KEGG Pathway | hsa04261 | Adrenergic signaling in cardiomyocytes | -6.660219801 | -5.381 | 32/144 | 108,115,488,596,775,776,781,783,817,1385,1390,2770,2776,3784,5332,5515,5516,5520,5525,5527,5529,5567,5578,5594,6331,6546,7170,9252,9586,10000,11069,55844 | ADCY2,ADCY9,ATP2A2,BCL2,CACNA1C,CACNA1D,CACNA2D1,CACNB2,CAMK2D,CREB1,CREM,GNAI1,GNAQ,KCNQ1,PLCB4,PPP2CA,PPP2CB,PPP2R2A,PPP2R5A,PPP2R5C,PPP2R5E,PRKACB,PRKCA,MAPK1,SCN5A,SLC8A1,TPM3,RPS6KA5,CREB5,AKT3,RAPGEF4,PPP2R2D |
| 10_Member | KEGG Pathway | hsa05142 | Chagas disease (American trypanosomiasis) | -3.172271313 | -2.382 | 19/102 | 2770,2774,2776,5291,5295,5332,5515,5516,5520,5594,5599,6416,7042,7046,7048,8503,9630,10000,55844 | GNAI1,GNAL,GNAQ,PIK3CB,PIK3R1,PLCB4,PPP2CA,PPP2CB,PPP2R2A,MAPK1,MAPK8,MAP2K4,TGFB2,TGFBR1,TGFBR2,PIK3R3,GNA14,AKT3,PPP2R2D |
| 11_Summary | KEGG Pathway | hsa04520 | Adherens junction | -8.79830578 | -7.146 | 24/72 | 60,87,998,1387,1457,1499,2033,2241,4008,4233,5594,5770,5792,5797,5879,6591,6885,6934,7046,7048,7414,8976,10580,51701 | ACTB,ACTN1,CDC42,CREBBP,CSNK2A1,CTNNB1,EP300,FER,LMO7,MET,MAPK1,PTPN1,PTPRF,PTPRM,RAC1,SNAI2,MAP3K7,TCF7L2,TGFBR1,TGFBR2,VCL,WASL,SORBS1,NLK |
| 11_Member | KEGG Pathway | hsa04520 | Adherens junction | -8.79830578 | -7.146 | 24/72 | 60,87,998,1387,1457,1499,2033,2241,4008,4233,5594,5770,5792,5797,5879,6591,6885,6934,7046,7048,7414,8976,10580,51701 | ACTB,ACTN1,CDC42,CREBBP,CSNK2A1,CTNNB1,EP300,FER,LMO7,MET,MAPK1,PTPN1,PTPRF,PTPRM,RAC1,SNAI2,MAP3K7,TCF7L2,TGFBR1,TGFBR2,VCL,WASL,SORBS1,NLK |
| 12_Summary | KEGG Pathway | hsa04390 | Hippo signaling pathway | -8.015688998 | -6.474 | 36/154 | 60,324,650,657,659,894,1490,1499,1739,1740,1741,2736,5515,5516,5520,5590,6591,6934,7003,7004,7042,7046,7048,7529,7531,7534,7855,8324,10971,23291,25937,26524,55844,64398,81029,85407,2737,5727,64399 | ACTB,APC,BMP2,BMPR1A,BMPR2,CCND2,CCN2,CTNNB1,DLG1,DLG2,DLG3,GLI2,PPP2CA,PPP2CB,PPP2R2A,PRKCZ,SNAI2,TCF7L2,TEAD1,TEAD4,TGFB2,TGFBR1,TGFBR2,YWHAB,YWHAE,YWHAZ,FZD5,FZD7,YWHAQ,FBXW11,WWTR1,LATS2,PPP2R2D,MPP5,WNT5B,NKD1,GLI3,PTCH1,HHIP |
| 12_Member | KEGG Pathway | hsa04390 | Hippo signaling pathway | -8.015688998 | -6.474 | 36/154 | 60,324,650,657,659,894,1490,1499,1739,1740,1741,2736,5515,5516,5520,5590,6591,6934,7003,7004,7042,7046,7048,7529,7531,7534,7855,8324,10971,23291,25937,26524,55844,64398,81029,85407 | ACTB,APC,BMP2,BMPR1A,BMPR2,CCND2,CCN2,CTNNB1,DLG1,DLG2,DLG3,GLI2,PPP2CA,PPP2CB,PPP2R2A,PRKCZ,SNAI2,TCF7L2,TEAD1,TEAD4,TGFB2,TGFBR1,TGFBR2,YWHAB,YWHAE,YWHAZ,FZD5,FZD7,YWHAQ,FBXW11,WWTR1,LATS2,PPP2R2D,MPP5,WNT5B,NKD1 |
| 12_Member | KEGG Pathway | hsa05217 | Basal cell carcinoma | -2.304243029 | -1.671 | 11/55 | 324,650,1499,2736,2737,5727,6934,7855,8324,64399,81029 | APC,BMP2,CTNNB1,GLI2,GLI3,PTCH1,TCF7L2,FZD5,FZD7,HHIP,WNT5B |
| 13_Summary | KEGG Pathway | hsa05166 | HTLV-I infection | -7.991285666 | -6.474 | 50/256 | 108,115,291,324,467,894,1385,1387,1390,1499,1739,1871,1958,2033,2113,3845,4214,4487,4603,4773,4775,5156,5291,5295,5530,5534,5567,5599,5901,6416,6908,6929,7042,7046,7048,7412,7416,7419,7514,7855,8324,8503,8829,8850,9519,10000,10393,23373,81029,94241 | ADCY2,ADCY9,SLC25A4,APC,ATF3,CCND2,CREB1,CREBBP,CREM,CTNNB1,DLG1,E2F3,EGR1,EP300,ETS1,KRAS,MAP3K1,MSX1,MYBL1,NFATC2,NFATC3,PDGFRA,PIK3CB,PIK3R1,PPP3CA,PPP3R1,PRKACB,MAPK8,RAN,MAP2K4,TBP,TCF3,TGFB2,TGFBR1,TGFBR2,VCAM1,VDAC1,VDAC3,XPO1,FZD5,FZD7,PIK3R3,NRP1,KAT2B,TBPL1,AKT3,ANAPC10,CRTC1,WNT5B,TP53INP1 |
| 13_Member | KEGG Pathway | hsa05166 | HTLV-I infection | -7.991285666 | -6.474 | 50/256 | 108,115,291,324,467,894,1385,1387,1390,1499,1739,1871,1958,2033,2113,3845,4214,4487,4603,4773,4775,5156,5291,5295,5530,5534,5567,5599,5901,6416,6908,6929,7042,7046,7048,7412,7416,7419,7514,7855,8324,8503,8829,8850,9519,10000,10393,23373,81029,94241 | ADCY2,ADCY9,SLC25A4,APC,ATF3,CCND2,CREB1,CREBBP,CREM,CTNNB1,DLG1,E2F3,EGR1,EP300,ETS1,KRAS,MAP3K1,MSX1,MYBL1,NFATC2,NFATC3,PDGFRA,PIK3CB,PIK3R1,PPP3CA,PPP3R1,PRKACB,MAPK8,RAN,MAP2K4,TBP,TCF3,TGFB2,TGFBR1,TGFBR2,VCAM1,VDAC1,VDAC3,XPO1,FZD5,FZD7,PIK3R3,NRP1,KAT2B,TBPL1,AKT3,ANAPC10,CRTC1,WNT5B,TP53INP1 |
| 14_Summary | KEGG Pathway | hsa04725 | Cholinergic synapse | -7.633452076 | -6.170 | 29/112 | 108,115,596,774,775,776,817,1129,1385,2770,2776,2782,3708,3717,3759,3763,3784,3786,3845,5291,5295,5332,5567,5578,5594,8503,9586,10000,56479,60,2244,2266,2977,3688,4638,4659,5028,5590,5592,5908,6093,9475,10672,2911,5580,6655,7184,9568,488,627,1387,2033,2737,2891,2903,5599,5727,5879,11069,64399,8660,10393,22849,27330,64506,80315,132864,1081,998,1237,1399,8976,57580 | ADCY2,ADCY9,BCL2,CACNA1B,CACNA1C,CACNA1D,CAMK2D,CHRM2,CREB1,GNAI1,GNAQ,GNB1,ITPR1,JAK2,KCNJ2,KCNJ6,KCNQ1,KCNQ3,KRAS,PIK3CB,PIK3R1,PLCB4,PRKACB,PRKCA,MAPK1,PIK3R3,CREB5,AKT3,KCNQ5,ACTB,FGB,FGG,GUCY1A2,ITGB1,MYLK,PPP1R12A,P2RY1,PRKCZ,PRKG1,RAP1B,ROCK1,ROCK2,GNA13,GRM1,PRKCD,SOS2,HSP90B1,GABBR2,ATP2A2,BDNF,CREBBP,EP300,GLI3,GRIA2,GRIN2A,MAPK8,PTCH1,RAC1,RAPGEF4,HHIP,IRS2,ANAPC10,CPEB3,RPS6KA6,CPEB1,CPEB4,CPEB2,CGA,CDC42,CCR8,CRKL,WASL,PREX1 |
| 14_Member | KEGG Pathway | hsa04725 | Cholinergic synapse | -7.633452076 | -6.170 | 29/112 | 108,115,596,774,775,776,817,1129,1385,2770,2776,2782,3708,3717,3759,3763,3784,3786,3845,5291,5295,5332,5567,5578,5594,8503,9586,10000,56479 | ADCY2,ADCY9,BCL2,CACNA1B,CACNA1C,CACNA1D,CAMK2D,CHRM2,CREB1,GNAI1,GNAQ,GNB1,ITPR1,JAK2,KCNJ2,KCNJ6,KCNQ1,KCNQ3,KRAS,PIK3CB,PIK3R1,PLCB4,PRKACB,PRKCA,MAPK1,PIK3R3,CREB5,AKT3,KCNQ5 |
| 14_Member | KEGG Pathway | hsa04611 | Platelet activation | -5.116215978 | -4.049 | 26/123 | 60,108,115,2244,2266,2770,2776,2977,3688,3708,4638,4659,5028,5291,5295,5332,5567,5590,5592,5594,5908,6093,8503,9475,10000,10672 | ACTB,ADCY2,ADCY9,FGB,FGG,GNAI1,GNAQ,GUCY1A2,ITGB1,ITPR1,MYLK,PPP1R12A,P2RY1,PIK3CB,PIK3R1,PLCB4,PRKACB,PRKCZ,PRKG1,MAPK1,RAP1B,ROCK1,PIK3R3,ROCK2,AKT3,GNA13 |
| 14_Member | KEGG Pathway | hsa04915 | Estrogen signaling pathway | -4.347361831 | -3.394 | 21/98 | 108,115,1385,2770,2776,2911,3708,3763,3845,5291,5295,5332,5567,5580,5594,6655,7184,8503,9568,9586,10000 | ADCY2,ADCY9,CREB1,GNAI1,GNAQ,GRM1,ITPR1,KCNJ6,KRAS,PIK3CB,PIK3R1,PLCB4,PRKACB,PRKCD,MAPK1,SOS2,HSP90B1,PIK3R3,GABBR2,CREB5,AKT3 |
| 14_Member | KEGG Pathway | hsa04024 | cAMP signaling pathway | -3.683356983 | -2.847 | 32/198 | 108,115,488,627,775,776,817,1129,1385,1387,2033,2737,2770,2891,2903,4659,5291,5295,5567,5594,5599,5727,5879,5908,6093,8503,9475,9568,9586,10000,11069,64399 | ADCY2,ADCY9,ATP2A2,BDNF,CACNA1C,CACNA1D,CAMK2D,CHRM2,CREB1,CREBBP,EP300,GLI3,GNAI1,GRIA2,GRIN2A,PPP1R12A,PIK3CB,PIK3R1,PRKACB,MAPK1,MAPK8,PTCH1,RAC1,RAP1B,ROCK1,PIK3R3,ROCK2,GABBR2,CREB5,AKT3,RAPGEF4,HHIP |
| 14_Member | KEGG Pathway | hsa04930 | Type II diabetes mellitus | -2.957533268 | -2.198 | 11/46 | 774,775,776,5291,5295,5580,5590,5594,5599,8503,8660 | CACNA1B,CACNA1C,CACNA1D,PIK3CB,PIK3R1,PRKCD,PRKCZ,MAPK1,MAPK8,PIK3R3,IRS2 |
| 14_Member | KEGG Pathway | hsa04914 | Progesterone-mediated oocyte maturation | -2.65187916 | -1.949 | 17/96 | 108,115,2770,3845,5291,5295,5567,5594,5599,8503,10000,10393,22849,27330,64506,80315,132864 | ADCY2,ADCY9,GNAI1,KRAS,PIK3CB,PIK3R1,PRKACB,MAPK1,MAPK8,PIK3R3,AKT3,ANAPC10,CPEB3,RPS6KA6,CPEB1,CPEB4,CPEB2 |
| 14_Member | KEGG Pathway | hsa04923 | Regulation of lipolysis in adipocytes | -2.368001335 | -1.723 | 11/54 | 108,115,1081,2770,5291,5295,5567,5592,8503,8660,10000 | ADCY2,ADCY9,CGA,GNAI1,PIK3CB,PIK3R1,PRKACB,PRKG1,PIK3R3,IRS2,AKT3 |
| 14_Member | KEGG Pathway | hsa04062 | Chemokine signaling pathway | -2.056691478 | -1.442 | 25/182 | 108,115,998,1237,1399,2770,2782,3717,3845,5291,5295,5332,5567,5580,5590,5594,5879,5908,6093,6655,8503,8976,9475,10000,57580 | ADCY2,ADCY9,CDC42,CCR8,CRKL,GNAI1,GNB1,JAK2,KRAS,PIK3CB,PIK3R1,PLCB4,PRKACB,PRKCD,PRKCZ,MAPK1,RAC1,RAP1B,ROCK1,SOS2,PIK3R3,WASL,ROCK2,AKT3,PREX1 |
| 15_Summary | KEGG Pathway | hsa04724 | Glutamatergic synapse | -7.448336403 | -6.059 | 29/114 | 108,115,775,776,2770,2776,2782,2891,2898,2900,2903,2911,2913,2915,3708,5332,5530,5534,5567,5578,5594,6505,6506,6507,7220,9456,22941,54407,81539,291,488,1385,2768,2969,2977,4208,4638,4659,4773,4775,5592,6093,6546,6547,7225,7416,7419,8660,9475,9586,10000,10242,10672,2781,2895,3845,4842,5515,5516,817,1387,2033,5908,27330,60,781,783,3759,3763,5563,51422,9252,59,1490,1958,3714,6198,7046,10891,774,1268,2554,5599,22999,1499,1906,3815,4286,6934,7855,8324,81029,1129,2066,2774,5156,9630,57620,90550,998,1081,4214,5580,6416,6655,56034,84617,3772,3784,7430,54207,3781,6616,9699,11069,27445,2308,5105,150094,3309,4036,7184,493869,4929,5587,6571 | ADCY2,ADCY9,CACNA1C,CACNA1D,GNAI1,GNAQ,GNB1,GRIA2,GRIK2,GRIK4,GRIN2A,GRM1,GRM3,GRM5,ITPR1,PLCB4,PPP3CA,PPP3R1,PRKACB,PRKCA,MAPK1,SLC1A1,SLC1A2,SLC1A3,TRPC1,HOMER1,SHANK2,SLC38A2,SLC38A1,SLC25A4,ATP2A2,CREB1,GNA12,GTF2I,GUCY1A2,MEF2C,MYLK,PPP1R12A,NFATC2,NFATC3,PRKG1,ROCK1,SLC8A1,SLC8A3,TRPC6,VDAC1,VDAC3,IRS2,ROCK2,CREB5,AKT3,KCNMB2,GNA13,GNAZ,GRID2,KRAS,NOS1,PPP2CA,PPP2CB,CAMK2D,CREBBP,EP300,RAP1B,RPS6KA6,ACTB,CACNA2D1,CACNB2,KCNJ2,KCNJ6,PRKAA2,PRKAG2,RPS6KA5,ACTA2,CCN2,EGR1,JAG2,RPS6KB1,TGFBR1,PPARGC1A,CACNA1B,CNR1,GABRA1,MAPK8,RIMS1,CTNNB1,EDN1,KIT,MITF,TCF7L2,FZD5,FZD7,WNT5B,CHRM2,ERBB4,GNAL,PDGFRA,GNA14,STIM2,MCU,CDC42,CGA,MAP3K1,PRKCD,MAP2K4,SOS2,PDGFC,TUBB6,KCNJ15,KCNQ1,EZR,KCNK10,KCNN2,SNAP25,RIMS2,RAPGEF4,PCLO,FOXO1,PCK1,SIK1,HSPA5,LRP2,HSP90B1,GPX8,NR4A2,PRKD1,SLC18A2 |
| 15_Member | KEGG Pathway | hsa04724 | Glutamatergic synapse | -7.448336403 | -6.059 | 29/114 | 108,115,775,776,2770,2776,2782,2891,2898,2900,2903,2911,2913,2915,3708,5332,5530,5534,5567,5578,5594,6505,6506,6507,7220,9456,22941,54407,81539 | ADCY2,ADCY9,CACNA1C,CACNA1D,GNAI1,GNAQ,GNB1,GRIA2,GRIK2,GRIK4,GRIN2A,GRM1,GRM3,GRM5,ITPR1,PLCB4,PPP3CA,PPP3R1,PRKACB,PRKCA,MAPK1,SLC1A1,SLC1A2,SLC1A3,TRPC1,HOMER1,SHANK2,SLC38A2,SLC38A1 |
| 15_Member | KEGG Pathway | hsa04022 | cGMP-PKG signaling pathway | -6.829712549 | -5.534 | 35/163 | 108,115,291,488,775,776,1385,2768,2770,2776,2969,2977,3708,4208,4638,4659,4773,4775,5332,5530,5534,5592,5594,6093,6546,6547,7225,7416,7419,8660,9475,9586,10000,10242,10672 | ADCY2,ADCY9,SLC25A4,ATP2A2,CACNA1C,CACNA1D,CREB1,GNA12,GNAI1,GNAQ,GTF2I,GUCY1A2,ITPR1,MEF2C,MYLK,PPP1R12A,NFATC2,NFATC3,PLCB4,PPP3CA,PPP3R1,PRKG1,MAPK1,ROCK1,SLC8A1,SLC8A3,TRPC6,VDAC1,VDAC3,IRS2,ROCK2,CREB5,AKT3,KCNMB2,GNA13 |
| 15_Member | KEGG Pathway | hsa04730 | Long-term depression | -5.99128859 | -4.803 | 18/60 | 2768,2770,2776,2781,2891,2895,2911,2977,3708,3845,4842,5332,5515,5516,5578,5592,5594,10672 | GNA12,GNAI1,GNAQ,GNAZ,GRIA2,GRID2,GRM1,GUCY1A2,ITPR1,KRAS,NOS1,PLCB4,PPP2CA,PPP2CB,PRKCA,PRKG1,MAPK1,GNA13 |
| 15_Member | KEGG Pathway | hsa04720 | Long-term potentiation | -5.873255982 | -4.698 | 19/67 | 775,817,1387,2033,2776,2891,2903,2911,2915,3708,3845,5332,5530,5534,5567,5578,5594,5908,27330 | CACNA1C,CAMK2D,CREBBP,EP300,GNAQ,GRIA2,GRIN2A,GRM1,GRM5,ITPR1,KRAS,PLCB4,PPP3CA,PPP3R1,PRKACB,PRKCA,MAPK1,RAP1B,RPS6KA6 |
| 15_Member | KEGG Pathway | hsa04921 | Oxytocin signaling pathway | -5.162856754 | -4.082 | 30/152 | 60,108,115,775,776,781,783,817,2770,2776,2977,3708,3759,3763,3845,4208,4638,4659,4773,4775,5332,5530,5534,5563,5567,5578,5594,6093,9475,51422 | ACTB,ADCY2,ADCY9,CACNA1C,CACNA1D,CACNA2D1,CACNB2,CAMK2D,GNAI1,GNAQ,GUCY1A2,ITPR1,KCNJ2,KCNJ6,KRAS,MEF2C,MYLK,PPP1R12A,NFATC2,NFATC3,PLCB4,PPP3CA,PPP3R1,PRKAA2,PRKACB,PRKCA,MAPK1,ROCK1,ROCK2,PRKAG2 |
| 15_Member | KEGG Pathway | hsa04713 | Circadian entrainment | -4.488127455 | -3.519 | 21/96 | 108,115,775,776,817,1385,2770,2776,2782,2891,2903,2977,3708,3763,4842,5332,5567,5578,5592,5594,9252 | ADCY2,ADCY9,CACNA1C,CACNA1D,CAMK2D,CREB1,GNAI1,GNAQ,GNB1,GRIA2,GRIN2A,GUCY1A2,ITPR1,KCNJ6,NOS1,PLCB4,PRKACB,PRKCA,PRKG1,MAPK1,RPS6KA5 |
| 15_Member | KEGG Pathway | hsa04371 | Apelin signaling pathway | -4.201896889 | -3.286 | 26/138 | 59,108,115,1490,1958,2770,2776,2782,3708,3714,3845,4208,4638,4842,5332,5563,5567,5594,6198,6546,6547,7046,10000,10672,10891,51422 | ACTA2,ADCY2,ADCY9,CCN2,EGR1,GNAI1,GNAQ,GNB1,ITPR1,JAG2,KRAS,MEF2C,MYLK,NOS1,PLCB4,PRKAA2,PRKACB,MAPK1,RPS6KB1,SLC8A1,SLC8A3,TGFBR1,AKT3,GNA13,PPARGC1A,PRKAG2 |
| 15_Member | KEGG Pathway | hsa04723 | Retrograde endocannabinoid signaling | -4.145032705 | -3.251 | 21/101 | 108,115,774,775,776,1268,2554,2770,2776,2782,2891,2911,2915,3708,3763,5332,5567,5578,5594,5599,22999 | ADCY2,ADCY9,CACNA1B,CACNA1C,CACNA1D,CNR1,GABRA1,GNAI1,GNAQ,GNB1,GRIA2,GRM1,GRM5,ITPR1,KCNJ6,PLCB4,PRKACB,PRKCA,MAPK1,MAPK8,RIMS1 |
| 15_Member | KEGG Pathway | hsa04916 | Melanogenesis | -4.145032705 | -3.251 | 21/101 | 108,115,817,1385,1387,1499,1906,2033,2770,2776,3815,3845,4286,5332,5567,5578,5594,6934,7855,8324,81029 | ADCY2,ADCY9,CAMK2D,CREB1,CREBBP,CTNNB1,EDN1,EP300,GNAI1,GNAQ,KIT,KRAS,MITF,PLCB4,PRKACB,PRKCA,MAPK1,TCF7L2,FZD5,FZD7,WNT5B |
| 15_Member | KEGG Pathway | hsa04020 | Calcium signaling pathway | -4.011995443 | -3.138 | 31/182 | 108,115,291,488,774,775,776,817,1129,2066,2774,2776,2903,2911,2915,3708,4638,4842,5156,5332,5530,5534,5567,5578,6546,6547,7416,7419,9630,57620,90550 | ADCY2,ADCY9,SLC25A4,ATP2A2,CACNA1B,CACNA1C,CACNA1D,CAMK2D,CHRM2,ERBB4,GNAL,GNAQ,GRIN2A,GRM1,GRM5,ITPR1,MYLK,NOS1,PDGFRA,PLCB4,PPP3CA,PPP3R1,PRKACB,PRKCA,SLC8A1,SLC8A3,VDAC1,VDAC3,GNA14,STIM2,MCU |
| 15_Member | KEGG Pathway | hsa04912 | GnRH signaling pathway | -3.76820509 | -2.913 | 19/92 | 108,115,775,776,817,998,1081,2776,3708,3845,4214,5332,5567,5578,5580,5594,5599,6416,6655 | ADCY2,ADCY9,CACNA1C,CACNA1D,CAMK2D,CDC42,CGA,GNAQ,ITPR1,KRAS,MAP3K1,PLCB4,PRKACB,PRKCA,PRKCD,MAPK1,MAPK8,MAP2K4,SOS2 |
| 15_Member | KEGG Pathway | hsa04540 | Gap junction | -3.547040625 | -2.717 | 18/88 | 108,115,2770,2776,2911,2915,2977,3708,3845,5156,5332,5567,5578,5592,5594,6655,56034,84617 | ADCY2,ADCY9,GNAI1,GNAQ,GRM1,GRM5,GUCY1A2,ITPR1,KRAS,PDGFRA,PLCB4,PRKACB,PRKCA,PRKG1,MAPK1,SOS2,PDGFC,TUBB6 |
| 15_Member | KEGG Pathway | hsa04971 | Gastric acid secretion | -3.432947261 | -2.608 | 16/75 | 60,108,115,817,2770,2776,3708,3759,3772,3784,4638,5332,5567,5578,7430,54207 | ACTB,ADCY2,ADCY9,CAMK2D,GNAI1,GNAQ,ITPR1,KCNJ2,KCNJ15,KCNQ1,MYLK,PLCB4,PRKACB,PRKCA,EZR,KCNK10 |
| 15_Member | KEGG Pathway | hsa04911 | Insulin secretion | -3.263712158 | -2.456 | 17/85 | 108,115,775,776,817,1385,2776,3781,5332,5567,5578,6616,9586,9699,10242,11069,27445 | ADCY2,ADCY9,CACNA1C,CACNA1D,CAMK2D,CREB1,GNAQ,KCNN2,PLCB4,PRKACB,PRKCA,SNAP25,CREB5,RIMS2,KCNMB2,RAPGEF4,PCLO |
| 15_Member | KEGG Pathway | hsa04922 | Glucagon signaling pathway | -3.118283397 | -2.333 | 19/103 | 108,817,1385,1387,2033,2308,2776,3708,5105,5332,5530,5534,5563,5567,9586,10000,10891,51422,150094 | ADCY2,CAMK2D,CREB1,CREBBP,EP300,FOXO1,GNAQ,ITPR1,PCK1,PLCB4,PPP3CA,PPP3R1,PRKAA2,PRKACB,CREB5,AKT3,PPARGC1A,PRKAG2,SIK1 |
| 15_Member | KEGG Pathway | hsa04270 | Vascular smooth muscle contraction | -3.022702306 | -2.248 | 21/121 | 59,108,115,775,776,2768,2776,2977,3708,4638,4659,5332,5567,5578,5580,5592,5594,6093,9475,10242,10672 | ACTA2,ADCY2,ADCY9,CACNA1C,CACNA1D,GNA12,GNAQ,GUCY1A2,ITPR1,MYLK,PPP1R12A,PLCB4,PRKACB,PRKCA,PRKCD,PRKG1,MAPK1,ROCK1,ROCK2,KCNMB2,GNA13 |
| 15_Member | KEGG Pathway | hsa04918 | Thyroid hormone synthesis | -2.550397085 | -1.870 | 14/74 | 108,115,1081,1385,2776,3309,3708,4036,5332,5567,5578,7184,9586,493869 | ADCY2,ADCY9,CGA,CREB1,GNAQ,HSPA5,ITPR1,LRP2,PLCB4,PRKACB,PRKCA,HSP90B1,CREB5,GPX8 |
| 15_Member | KEGG Pathway | hsa04924 | Renin secretion | -2.174168357 | -1.552 | 12/65 | 775,776,1385,2770,2776,2977,3708,3759,5332,5530,5534,5567 | CACNA1C,CACNA1D,CREB1,GNAI1,GNAQ,GUCY1A2,ITPR1,KCNJ2,PLCB4,PPP3CA,PPP3R1,PRKACB |
| 15_Member | KEGG Pathway | hsa04925 | Aldosterone synthesis and secretion | -2.136934237 | -1.519 | 14/82 | 108,115,775,776,817,1385,2776,3708,4929,5332,5567,5578,5587,9586 | ADCY2,ADCY9,CACNA1C,CACNA1D,CAMK2D,CREB1,GNAQ,ITPR1,NR4A2,PLCB4,PRKACB,PRKCA,PRKD1,CREB5 |
| 15_Member | KEGG Pathway | hsa05031 | Amphetamine addiction | -2.016708357 | -1.413 | 12/68 | 775,776,817,1385,2891,2903,5530,5534,5567,5578,6571,9586 | CACNA1C,CACNA1D,CAMK2D,CREB1,GRIA2,GRIN2A,PPP3CA,PPP3R1,PRKACB,PRKCA,SLC18A2,CREB5 |
| 16_Summary | KEGG Pathway | hsa04550 | Signaling pathways regulating pluripotency of stem cells | -7.036606944 | -5.685 | 32/139 | 90,92,324,657,659,1499,1749,2261,2263,3175,3624,3625,3717,3720,3845,3977,4211,5080,5291,5295,5594,5978,6929,7855,7994,8324,8503,9314,10000,10336,81029,84333 | ACVR1,ACVR2A,APC,BMPR1A,BMPR2,CTNNB1,DLX5,FGFR3,FGFR2,ONECUT1,INHBA,INHBB,JAK2,JARID2,KRAS,LIFR,MEIS1,PAX6,PIK3CB,PIK3R1,MAPK1,REST,TCF3,FZD5,KAT6A,FZD7,PIK3R3,KLF4,AKT3,PCGF3,WNT5B,PCGF5 |
| 16_Member | KEGG Pathway | hsa04550 | Signaling pathways regulating pluripotency of stem cells | -7.036606944 | -5.685 | 32/139 | 90,92,324,657,659,1499,1749,2261,2263,3175,3624,3625,3717,3720,3845,3977,4211,5080,5291,5295,5594,5978,6929,7855,7994,8324,8503,9314,10000,10336,81029,84333 | ACVR1,ACVR2A,APC,BMPR1A,BMPR2,CTNNB1,DLX5,FGFR3,FGFR2,ONECUT1,INHBA,INHBB,JAK2,JARID2,KRAS,LIFR,MEIS1,PAX6,PIK3CB,PIK3R1,MAPK1,REST,TCF3,FZD5,KAT6A,FZD7,PIK3R3,KLF4,AKT3,PCGF3,WNT5B,PCGF5 |
| 17_Summary | KEGG Pathway | hsa05215 | Prostate cancer | -6.996020501 | -5.664 | 24/87 | 367,596,1027,1385,1387,1499,1871,2033,2263,2308,3845,5156,5170,5291,5295,5594,5728,6655,6934,7184,8503,9586,10000,56034,523,526,528,1977,2887,4040,5563,5578,6009,6198,6249,6446,7855,8324,9706,23175,27330,57600,58528,81029,81617,220441,253260,998,1399,2113,4233,5062,5879,5908,7042,54583,1906,1958,3717,5332,5580,5590,5599,7046,7048,7412,1119,1607,8976,10434,23446,56261,160851,204962,60,488,1734,5567,7068,8202,8850,9969,10499,23389,116931,397,627,817,4214,4916,7531,9252,10019,10818,1654,4773,4775,6416,7419,7529,7534,10971,29110,2261,3084,324,26060,108,115,375,1759,2768,2911,2913,2915,3815,5898,10672,11069,26052,56895,2252,2255,2258,2259,3714,8600,28514,122011,5888,10928,27,868,2066,4286,1488,2122,4306,23327,894,1081,9306,9655,1540,4982,5468,5530,5534,6885,7186,5915,27436,5209,7010,1739,862,3655,3688,3915,7187 | AR,BCL2,CDKN1B,CREB1,CREBBP,CTNNB1,E2F3,EP300,FGFR2,FOXO1,KRAS,PDGFRA,PDPK1,PIK3CB,PIK3R1,MAPK1,PTEN,SOS2,TCF7L2,HSP90B1,PIK3R3,CREB5,AKT3,PDGFC,ATP6V1A,ATP6V1B2,ATP6V1C1,EIF4E,GRB10,LRP6,PRKAA2,PRKCA,RHEB,RPS6KB1,CLIP1,SGK1,FZD5,FZD7,ULK2,LPIN1,RPS6KA6,FNIP2,RRAGD,WNT5B,CAB39L,RNF152,RICTOR,CDC42,CRKL,ETS1,MET,PAK2,RAC1,RAP1B,TGFB2,EGLN1,EDN1,EGR1,JAK2,PLCB4,PRKCD,PRKCZ,MAPK8,TGFBR1,TGFBR2,VCAM1,CHKA,DGKB,WASL,LYPLA1,SLC44A1,GPCPD1,DGKH,SLC44A5,ACTB,ATP2A2,DIO2,PRKACB,THRB,NCOA3,KAT2B,MED13,NCOA2,MED13L,MED12L,ARHGDIB,BDNF,CAMK2D,MAP3K1,NTRK3,YWHAE,RPS6KA5,SH2B3,FRS2,DDX3X,NFATC2,NFATC3,MAP2K4,VDAC3,YWHAB,YWHAZ,YWHAQ,TBK1,FGFR3,NRG1,APC,APPL1,ADCY2,ADCY9,ARF1,DNM1,GNA12,GRM1,GRM3,GRM5,KIT,RALA,GNA13,RAPGEF4,DNM3,AGPAT4,FGF7,FGF10,FGF13,FGF14,JAG2,TNFSF11,DLL1,CSNK1A1L,RAD51,RALBP1,ABL2,CBLB,ERBB4,MITF,CTBP2,MECOM,NR3C2,NEDD4L,CCND2,CGA,SOCS6,SOCS5,CYLD,TNFRSF11B,PPARG,PPP3CA,PPP3R1,MAP3K7,TRAF2,RARB,EML4,PFKFB3,TEK,DLG1,RUNX1T1,ITGA6,ITGB1,LAMC1,TRAF3 |
| 17_Member | KEGG Pathway | hsa05215 | Prostate cancer | -6.996020501 | -5.664 | 24/87 | 367,596,1027,1385,1387,1499,1871,2033,2263,2308,3845,5156,5170,5291,5295,5594,5728,6655,6934,7184,8503,9586,10000,56034 | AR,BCL2,CDKN1B,CREB1,CREBBP,CTNNB1,E2F3,EP300,FGFR2,FOXO1,KRAS,PDGFRA,PDPK1,PIK3CB,PIK3R1,MAPK1,PTEN,SOS2,TCF7L2,HSP90B1,PIK3R3,CREB5,AKT3,PDGFC |
| 17_Member | KEGG Pathway | hsa04150 | mTOR signaling pathway | -6.168679946 | -4.952 | 32/151 | 523,526,528,1977,2887,3845,4040,5170,5291,5295,5563,5578,5594,5728,6009,6198,6249,6446,6655,7855,8324,8503,9706,10000,23175,27330,57600,58528,81029,81617,220441,253260 | ATP6V1A,ATP6V1B2,ATP6V1C1,EIF4E,GRB10,KRAS,LRP6,PDPK1,PIK3CB,PIK3R1,PRKAA2,PRKCA,MAPK1,PTEN,RHEB,RPS6KB1,CLIP1,SGK1,SOS2,FZD5,FZD7,PIK3R3,ULK2,AKT3,LPIN1,RPS6KA6,FNIP2,RRAGD,WNT5B,CAB39L,RNF152,RICTOR |
| 17_Member | KEGG Pathway | hsa05211 | Renal cell carcinoma | -5.434580389 | -4.272 | 18/65 | 998,1387,1399,2033,2113,3845,4233,5062,5291,5295,5594,5879,5908,6655,7042,8503,10000,54583 | CDC42,CREBBP,CRKL,EP300,ETS1,KRAS,MET,PAK2,PIK3CB,PIK3R1,MAPK1,RAC1,RAP1B,SOS2,TGFB2,PIK3R3,AKT3,EGLN1 |
| 17_Member | KEGG Pathway | hsa04933 | AGE-RAGE signaling pathway in diabetic complications | -5.316659804 | -4.203 | 23/99 | 596,998,1027,1906,1958,2308,3717,3845,5291,5295,5332,5578,5580,5590,5594,5599,5879,7042,7046,7048,7412,8503,10000 | BCL2,CDC42,CDKN1B,EDN1,EGR1,FOXO1,JAK2,KRAS,PIK3CB,PIK3R1,PLCB4,PRKCA,PRKCD,PRKCZ,MAPK1,MAPK8,RAC1,TGFB2,TGFBR1,TGFBR2,VCAM1,PIK3R3,AKT3 |
| 17_Member | KEGG Pathway | hsa05231 | Choline metabolism in cancer | -5.316659804 | -4.203 | 23/99 | 1119,1607,3845,5156,5170,5291,5295,5578,5594,5599,5879,6009,6198,6655,8503,8976,10000,10434,23446,56034,56261,160851,204962 | CHKA,DGKB,KRAS,PDGFRA,PDPK1,PIK3CB,PIK3R1,PRKCA,MAPK1,MAPK8,RAC1,RHEB,RPS6KB1,SOS2,PIK3R3,WASL,AKT3,LYPLA1,SLC44A1,PDGFC,GPCPD1,DGKH,SLC44A5 |
| 17_Member | KEGG Pathway | hsa04919 | Thyroid hormone signaling pathway | -5.099987725 | -4.049 | 25/116 | 60,488,1387,1499,1734,2033,2308,3845,5170,5291,5295,5332,5567,5578,5594,6009,7068,8202,8503,8850,9969,10000,10499,23389,116931 | ACTB,ATP2A2,CREBBP,CTNNB1,DIO2,EP300,FOXO1,KRAS,PDPK1,PIK3CB,PIK3R1,PLCB4,PRKACB,PRKCA,MAPK1,RHEB,THRB,NCOA3,PIK3R3,KAT2B,MED13,AKT3,NCOA2,MED13L,MED12L |
| 17_Member | KEGG Pathway | hsa04722 | Neurotrophin signaling pathway | -4.895538058 | -3.874 | 25/119 | 397,596,627,817,998,1399,3845,4214,4916,5170,5291,5295,5580,5594,5599,5879,5908,6655,7531,8503,9252,10000,10019,10818,27330 | ARHGDIB,BCL2,BDNF,CAMK2D,CDC42,CRKL,KRAS,MAP3K1,NTRK3,PDPK1,PIK3CB,PIK3R1,PRKCD,MAPK1,MAPK8,RAC1,RAP1B,SOS2,YWHAE,PIK3R3,RPS6KA5,AKT3,SH2B3,FRS2,RPS6KA6 |
| 17_Member | KEGG Pathway | hsa05161 | Hepatitis B | -4.736834021 | -3.725 | 28/144 | 596,1027,1385,1387,1654,1871,2033,3845,4214,4773,4775,5291,5295,5578,5594,5599,5728,6416,7042,7046,7419,7529,7534,8503,9586,10000,10971,29110 | BCL2,CDKN1B,CREB1,CREBBP,DDX3X,E2F3,EP300,KRAS,MAP3K1,NFATC2,NFATC3,PIK3CB,PIK3R1,PRKCA,MAPK1,MAPK8,PTEN,MAP2K4,TGFB2,TGFBR1,VDAC3,YWHAB,YWHAZ,PIK3R3,CREB5,AKT3,YWHAQ,TBK1 |
| 17_Member | KEGG Pathway | hsa01521 | EGFR tyrosine kinase inhibitor resistance | -4.728952668 | -3.725 | 19/79 | 596,1977,2261,2263,3084,3717,3845,4233,5156,5291,5295,5578,5594,5728,6198,6655,8503,10000,56034 | BCL2,EIF4E,FGFR3,FGFR2,NRG1,JAK2,KRAS,MET,PDGFRA,PIK3CB,PIK3R1,PRKCA,MAPK1,PTEN,RPS6KB1,SOS2,PIK3R3,AKT3,PDGFC |
| 17_Member | KEGG Pathway | hsa05210 | Colorectal cancer | -4.673000443 | -3.678 | 16/60 | 324,596,1499,3845,5291,5295,5594,5599,5879,6934,7042,7046,7048,8503,10000,26060 | APC,BCL2,CTNNB1,KRAS,PIK3CB,PIK3R1,MAPK1,MAPK8,RAC1,TCF7L2,TGFB2,TGFBR1,TGFBR2,PIK3R3,AKT3,APPL1 |
| 17_Member | KEGG Pathway | hsa04072 | Phospholipase D signaling pathway | -4.621870973 | -3.644 | 28/146 | 108,115,375,1607,1759,2768,2911,2913,2915,3815,3845,5156,5291,5295,5332,5578,5594,5898,6009,6655,8503,10000,10672,11069,26052,56034,56895,160851 | ADCY2,ADCY9,ARF1,DGKB,DNM1,GNA12,GRM1,GRM3,GRM5,KIT,KRAS,PDGFRA,PIK3CB,PIK3R1,PLCB4,PRKCA,MAPK1,RALA,RHEB,SOS2,PIK3R3,AKT3,GNA13,RAPGEF4,DNM3,PDGFC,AGPAT4,DGKH |
| 17_Member | KEGG Pathway | hsa05224 | Breast cancer | -4.300953323 | -3.359 | 27/144 | 324,1499,1871,2252,2255,2258,2259,3714,3815,3845,4040,5291,5295,5594,5728,6198,6655,6934,7855,8202,8324,8503,8600,10000,28514,81029,122011 | APC,CTNNB1,E2F3,FGF7,FGF10,FGF13,FGF14,JAG2,KIT,KRAS,LRP6,PIK3CB,PIK3R1,MAPK1,PTEN,RPS6KB1,SOS2,TCF7L2,FZD5,NCOA3,FZD7,PIK3R3,TNFSF11,AKT3,DLL1,WNT5B,CSNK1A1L |
| 17_Member | KEGG Pathway | hsa05212 | Pancreatic cancer | -4.297256703 | -3.359 | 16/64 | 998,1871,3845,5291,5295,5594,5599,5879,5888,5898,7042,7046,7048,8503,10000,10928 | CDC42,E2F3,KRAS,PIK3CB,PIK3R1,MAPK1,MAPK8,RAC1,RAD51,RALA,TGFB2,TGFBR1,TGFBR2,PIK3R3,AKT3,RALBP1 |
| 17_Member | KEGG Pathway | hsa04012 | ErbB signaling pathway | -4.181823169 | -3.273 | 19/86 | 27,817,868,1027,1399,2066,3084,3845,5062,5291,5295,5578,5594,5599,6198,6416,6655,8503,10000 | ABL2,CAMK2D,CBLB,CDKN1B,CRKL,ERBB4,NRG1,KRAS,PAK2,PIK3CB,PIK3R1,PRKCA,MAPK1,MAPK8,RPS6KB1,MAP2K4,SOS2,PIK3R3,AKT3 |
| 17_Member | KEGG Pathway | hsa05218 | Melanoma | -3.876784602 | -3.009 | 16/69 | 1871,2252,2255,2258,2259,3845,4233,4286,5156,5291,5295,5594,5728,8503,10000,56034 | E2F3,FGF7,FGF10,FGF13,FGF14,KRAS,MET,MITF,PDGFRA,PIK3CB,PIK3R1,MAPK1,PTEN,PIK3R3,AKT3,PDGFC |
| 17_Member | KEGG Pathway | hsa05220 | Chronic myeloid leukemia | -3.722037936 | -2.873 | 16/71 | 868,1027,1399,1488,1871,2122,3845,5291,5295,5594,6655,7042,7046,7048,8503,10000 | CBLB,CDKN1B,CRKL,CTBP2,E2F3,MECOM,KRAS,PIK3CB,PIK3R1,MAPK1,SOS2,TGFB2,TGFBR1,TGFBR2,PIK3R3,AKT3 |
| 17_Member | KEGG Pathway | hsa05213 | Endometrial cancer | -3.19263337 | -2.391 | 12/50 | 324,1499,3845,5170,5291,5295,5594,5728,6655,6934,8503,10000 | APC,CTNNB1,KRAS,PDPK1,PIK3CB,PIK3R1,MAPK1,PTEN,SOS2,TCF7L2,PIK3R3,AKT3 |
| 17_Member | KEGG Pathway | hsa04960 | Aldosterone-regulated sodium reabsorption | -3.181041279 | -2.385 | 10/37 | 3845,4306,5170,5291,5295,5578,5594,6446,8503,23327 | KRAS,NR3C2,PDPK1,PIK3CB,PIK3R1,PRKCA,MAPK1,SGK1,PIK3R3,NEDD4L |
| 17_Member | KEGG Pathway | hsa01522 | Endocrine resistance | -3.070735045 | -2.291 | 18/96 | 108,115,596,1027,1871,3714,3845,5291,5295,5567,5594,5599,6198,6655,8202,8503,10000,28514 | ADCY2,ADCY9,BCL2,CDKN1B,E2F3,JAG2,KRAS,PIK3CB,PIK3R1,PRKACB,MAPK1,MAPK8,RPS6KB1,SOS2,NCOA3,PIK3R3,AKT3,DLL1 |
| 17_Member | KEGG Pathway | hsa04917 | Prolactin signaling pathway | -2.787819804 | -2.058 | 14/70 | 894,1081,3717,3845,5291,5295,5594,5599,6655,8503,8600,9306,9655,10000 | CCND2,CGA,JAK2,KRAS,PIK3CB,PIK3R1,MAPK1,MAPK8,SOS2,PIK3R3,TNFSF11,SOCS6,SOCS5,AKT3 |
| 17_Member | KEGG Pathway | hsa05230 | Central carbon metabolism in cancer | -2.627658379 | -1.930 | 13/65 | 2261,2263,3815,3845,4233,4916,5156,5291,5295,5594,5728,8503,10000 | FGFR3,FGFR2,KIT,KRAS,MET,NTRK3,PDGFRA,PIK3CB,PIK3R1,MAPK1,PTEN,PIK3R3,AKT3 |
| 17_Member | KEGG Pathway | hsa04380 | Osteoclast differentiation | -2.621437151 | -1.928 | 21/130 | 1385,1540,4286,4773,4982,5291,5295,5468,5530,5534,5594,5599,5879,6885,7042,7046,7048,7186,8503,8600,10000 | CREB1,CYLD,MITF,NFATC2,TNFRSF11B,PIK3CB,PIK3R1,PPARG,PPP3CA,PPP3R1,MAPK1,MAPK8,RAC1,MAP3K7,TGFB2,TGFBR1,TGFBR2,TRAF2,PIK3R3,TNFSF11,AKT3 |
| 17_Member | KEGG Pathway | hsa05223 | Non-small cell lung cancer | -2.595277876 | -1.906 | 12/58 | 1871,3845,5170,5291,5295,5578,5594,5915,6655,8503,10000,27436 | E2F3,KRAS,PDPK1,PIK3CB,PIK3R1,PRKCA,MAPK1,RARB,SOS2,PIK3R3,AKT3,EML4 |
| 17_Member | KEGG Pathway | hsa04370 | VEGF signaling pathway | -2.529992065 | -1.857 | 12/59 | 998,3845,4773,5291,5295,5530,5534,5578,5594,5879,8503,10000 | CDC42,KRAS,NFATC2,PIK3CB,PIK3R1,PPP3CA,PPP3R1,PRKCA,MAPK1,RAC1,PIK3R3,AKT3 |
| 17_Member | KEGG Pathway | hsa04066 | HIF-1 signaling pathway | -2.413597745 | -1.757 | 17/101 | 596,817,1027,1387,1906,1977,2033,5209,5291,5295,5578,5594,6198,7010,8503,10000,54583 | BCL2,CAMK2D,CDKN1B,CREBBP,EDN1,EIF4E,EP300,PFKFB3,PIK3CB,PIK3R1,PRKCA,MAPK1,RPS6KB1,TEK,PIK3R3,AKT3,EGLN1 |
| 17_Member | KEGG Pathway | hsa04660 | T cell receptor signaling pathway | -2.324320271 | -1.684 | 17/103 | 868,998,1739,3845,4773,4775,5062,5170,5291,5295,5530,5534,5594,6655,6885,8503,10000 | CBLB,CDC42,DLG1,KRAS,NFATC2,NFATC3,PAK2,PDPK1,PIK3CB,PIK3R1,PPP3CA,PPP3R1,MAPK1,SOS2,MAP3K7,PIK3R3,AKT3 |
| 17_Member | KEGG Pathway | hsa05221 | Acute myeloid leukemia | -2.304243029 | -1.671 | 11/55 | 862,3815,3845,5291,5295,5594,6198,6655,6934,8503,10000 | RUNX1T1,KIT,KRAS,PIK3CB,PIK3R1,MAPK1,RPS6KB1,SOS2,TCF7L2,PIK3R3,AKT3 |
| 17_Member | KEGG Pathway | hsa05214 | Glioma | -2.229505914 | -1.604 | 12/64 | 817,1871,3845,5156,5291,5295,5578,5594,5728,6655,8503,10000 | CAMK2D,E2F3,KRAS,PDGFRA,PIK3CB,PIK3R1,PRKCA,MAPK1,PTEN,SOS2,PIK3R3,AKT3 |
| 17_Member | KEGG Pathway | hsa05222 | Small cell lung cancer | -2.044714807 | -1.434 | 14/84 | 596,1027,1871,3655,3688,3915,5291,5295,5728,5915,7186,7187,8503,10000 | BCL2,CDKN1B,E2F3,ITGA6,ITGB1,LAMC1,PIK3CB,PIK3R1,PTEN,RARB,TRAF2,TRAF3,PIK3R3,AKT3 |
| 17_Member | KEGG Pathway | hsa04664 | Fc epsilon RI signaling pathway | -2.016708357 | -1.413 | 12/68 | 3845,5170,5291,5295,5578,5594,5599,5879,6416,6655,8503,10000 | KRAS,PDPK1,PIK3CB,PIK3R1,PRKCA,MAPK1,MAPK8,RAC1,MAP2K4,SOS2,PIK3R3,AKT3 |
| 18_Summary | KEGG Pathway | hsa04530 | Tight junction | -6.364396897 | -5.118 | 35/170 | 60,87,776,998,1739,1740,1741,3688,4214,4627,4628,5515,5516,5520,5563,5567,5590,5599,5879,6093,7430,8531,8976,9080,9223,9414,9475,9693,23327,23370,51422,51762,55844,57180,64398 | ACTB,ACTN1,CACNA1D,CDC42,DLG1,DLG2,DLG3,ITGB1,MAP3K1,MYH9,MYH10,PPP2CA,PPP2CB,PPP2R2A,PRKAA2,PRKACB,PRKCZ,MAPK8,RAC1,ROCK1,EZR,YBX3,WASL,CLDN9,MAGI1,TJP2,ROCK2,RAPGEF2,NEDD4L,ARHGEF18,PRKAG2,RAB8B,PPP2R2D,ACTR3B,MPP5 |
| 18_Member | KEGG Pathway | hsa04530 | Tight junction | -6.364396897 | -5.118 | 35/170 | 60,87,776,998,1739,1740,1741,3688,4214,4627,4628,5515,5516,5520,5563,5567,5590,5599,5879,6093,7430,8531,8976,9080,9223,9414,9475,9693,23327,23370,51422,51762,55844,57180,64398 | ACTB,ACTN1,CACNA1D,CDC42,DLG1,DLG2,DLG3,ITGB1,MAP3K1,MYH9,MYH10,PPP2CA,PPP2CB,PPP2R2A,PRKAA2,PRKACB,PRKCZ,MAPK8,RAC1,ROCK1,EZR,YBX3,WASL,CLDN9,MAGI1,TJP2,ROCK2,RAPGEF2,NEDD4L,ARHGEF18,PRKAG2,RAB8B,PPP2R2D,ACTR3B,MPP5 |
| 19_Summary | KEGG Pathway | hsa04340 | Hedgehog signaling pathway | -6.220295231 | -4.989 | 16/47 | 596,894,2736,2737,4036,5567,5727,8452,11127,23291,53944,57154,64399,64750,122011,339745 | BCL2,CCND2,GLI2,GLI3,LRP2,PRKACB,PTCH1,CUL3,KIF3A,FBXW11,CSNK1G1,SMURF1,HHIP,SMURF2,CSNK1A1L,SPOPL |
| 19_Member | KEGG Pathway | hsa04340 | Hedgehog signaling pathway | -6.220295231 | -4.989 | 16/47 | 596,894,2736,2737,4036,5567,5727,8452,11127,23291,53944,57154,64399,64750,122011,339745 | BCL2,CCND2,GLI2,GLI3,LRP2,PRKACB,PTCH1,CUL3,KIF3A,FBXW11,CSNK1G1,SMURF1,HHIP,SMURF2,CSNK1A1L,SPOPL |
| 20_Summary | KEGG Pathway | hsa04114 | Oocyte meiosis | -6.06083618 | -4.858 | 28/124 | 108,115,367,817,3708,5515,5516,5525,5527,5529,5530,5534,5567,5594,6500,7529,7531,7534,9126,9748,10393,10971,22849,23291,27330,64506,80315,132864 | ADCY2,ADCY9,AR,CAMK2D,ITPR1,PPP2CA,PPP2CB,PPP2R5A,PPP2R5C,PPP2R5E,PPP3CA,PPP3R1,PRKACB,MAPK1,SKP1,YWHAB,YWHAE,YWHAZ,SMC3,SLK,ANAPC10,YWHAQ,CPEB3,FBXW11,RPS6KA6,CPEB1,CPEB4,CPEB2 |
| 20_Member | KEGG Pathway | hsa04114 | Oocyte meiosis | -6.06083618 | -4.858 | 28/124 | 108,115,367,817,3708,5515,5516,5525,5527,5529,5530,5534,5567,5594,6500,7529,7531,7534,9126,9748,10393,10971,22849,23291,27330,64506,80315,132864 | ADCY2,ADCY9,AR,CAMK2D,ITPR1,PPP2CA,PPP2CB,PPP2R5A,PPP2R5C,PPP2R5E,PPP3CA,PPP3R1,PRKACB,MAPK1,SKP1,YWHAB,YWHAE,YWHAZ,SMC3,SLK,ANAPC10,YWHAQ,CPEB3,FBXW11,RPS6KA6,CPEB1,CPEB4,CPEB2 |
