# Supplementary material for: MiRNA Profiling in Pectoral Muscle Throughout Pre- to Post-Natal Stages of Chicken Development
Source: Front Genet. 2020 Jun 23;11:570. doi: 10.3389/fgene.2020.00570 (PMC7324647; doi:10.3389/fgene.2020.00570)
Supplement: Supplementary file 1 [file Data_Sheet_1.ZIP › Supplementary Material/table S5 Gene Ontology metascape_result.docx]

| **GroupID** | **Category** | **Term** | **Description** | **LogP** | **Log(q-value)** | **InTerm_InList** | **Genes** | **Symbols** |
| --- | --- | --- | --- | --- | --- | --- | --- | --- |
| 1_Summary | GO Biological Processes | GO:0032990 | cell part morphogenesis | -47.51309675 | -43.236 | 209/685 | 25,60,102,288,323,351,387,473,655,659,960,998,1009,1123,1385,1400,1499,1501,1600,1808,1826,1857,1946,1956,2017,2043,2044,2045,2048,2115,2263,2297,2332,2335,2534,2549,2625,2668,2736,2737,2823,2885,2887,3688,3800,3984,4038,4076,4131,4133,4205,4534,4628,4684,4734,4756,4781,4803,4851,4897,4915,4929,5048,5063,5168,5291,5295,5306,5361,5362,5530,5578,5588,5594,5604,5649,5727,5728,5789,5797,5803,5911,6091,6092,6093,6239,6383,6477,6642,6654,6683,6709,6711,6722,6857,6860,7074,7101,7155,7204,7224,7337,7430,7436,7465,7533,7852,8013,8239,8301,8408,8609,8633,8650,8660,8766,8828,8829,8851,8936,8976,9037,9201,9211,9252,9331,9334,9353,9369,9378,9448,9693,9699,9706,9723,9798,9839,10006,10048,10087,10152,10160,10458,10479,10507,10631,10752,10787,10818,10890,10939,11127,11141,11252,22854,22871,22903,22941,22999,23011,23072,23077,23098,23114,23189,23242,23327,23394,23543,23603,23767,23768,25942,26039,26053,26999,27020,27023,27255,30011,50618,51761,55607,55740,56947,57142,57154,57520,57556,57688,57689,59277,64919,65981,80025,80031,81565,84189,84612,84631,84665,114798,139065,140578,152330,220164,284217,375790,389549,3398,4208,10427,367,1303,1398,1399,1955,2033,2195,3037,3915,4627,5629,5829,5861,6781,8452,10109,29071,55435,57493,64856,79834,79977,81578,27,182,375,411,463,604,650,654,1268,1272,1482,1656,1977,2119,2138,2185,2281,2475,2895,3082,3169,4139,4760,4762,5274,5469,5584,5597,5756,5792,5793,5978,6453,6616,6657,6664,6695,6929,6935,6938,7068,7161,7248,7532,8321,9043,9231,9314,9745,9860,9863,22849,22882,23064,23081,23108,23251,23258,23271,23373,23493,23531,23613,25793,26281,27063,28514,29116,30812,50807,55704,55714,55754,55906,56478,57118,57534,64764,91752,114788,221074,221935,285016,3717,3725,4685,5354,23162,27242,324,688,1540,2059,2904,3064,3815,4134,4651,5420,5868,5898,5962,7046,7813,9456,9474,10178,10253,11138,22873,25940,51762,54443,54874,55633,55737,57489,57533,64422,64786,89795,125058,1869,4124,5270,5501,6009,7424,8604,10636,10810,22883,23013,26045,57633,84504,118738,158038,347731,118,5139,5991,9472,10221,27302,284,1130,1843,1880,1901,2252,2255,2277,3084,3146,3491,3680,4856,5142,5144,5155,5156,5771,6558,7042,7057,7114,7412,8817,9547,9732,10928,64094,65125,151647,158747 | ABL1,ACTB,ADAM10,ANK3,APBB2,APP,RHOA,RERE,BMP7,BMPR2,CD44,CDC42,CDH11,CHN1,CREB1,CRMP1,CTNNB1,CTNND2,DAB1,DPYSL2,DSCAM,DVL3,EFNA5,EGFR,CTTN,EPHA4,EPHA5,EPHA7,EPHB2,ETV1,FGFR2,FOXD1,FMR1,FN1,FYN,GAB1,GATA3,GDNF,GLI2,GLI3,GPM6A,GRB2,GRB10,ITGB1,KIF5C,LIMK1,LRP4,CAPRIN1,MAP1B,MAP2,MEF2A,MTM1,MYH10,NCAM1,NEDD4,NEO1,NFIB,NGF,NOTCH1,NRCAM,NTRK2,NR4A2,PAFAH1B1,PAK3,ENPP2,PIK3CB,PIK3R1,PITPNA,PLXNA1,PLXNA2,PPP3CA,PRKCA,PRKCQ,MAPK1,MAP2K1,RELN,PTCH1,PTEN,PTPRD,PTPRM,PTPRZ1,RAP2A,ROBO1,ROBO2,ROCK1,RREB1,SDC2,SIAH1,SNX1,SOS1,SPAST,SPTAN1,SPTBN1,SRF,SYT1,SYT4,TIAM1,NR2E1,TOP2B,TRIO,TRPC5,UBE3A,EZR,VLDLR,WEE1,YWHAH,CXCR4,NR4A3,USP9X,PICALM,ULK1,KLF7,UNC5C,NUMB,IRS2,RAB11A,NRP2,NRP1,CDK5R1,WASF1,WASL,SEMA5A,DCLK1,LGI1,RPS6KA5,B4GALT6,B4GALT5,SLIT2,NRXN3,NRXN1,MAP4K4,RAPGEF2,RIMS2,ULK2,SEMA3E,IST1,ZEB2,ABI1,RANBP9,CERT1,ABI2,FARP1,BAIAP2,SLC9A6,SEMA4D,POSTN,CHL1,NCKAP1,FRS2,RAB10,AFG3L2,KIF3A,IL1RAPL1,PACSIN2,NTNG1,NLGN1,BTBD3,SHANK2,RIMS1,RAB21,HECW1,MYCBP2,SARM1,NFASC,KANK1,COBL,NEDD4L,ADNP,RBFOX2,CORO1C,FLRT3,FLRT2,SIN3A,SS18L1,AUTS2,CYFIP2,NPTN,FOXB1,CNTN6,SH3KBP1,ITSN2,ATP8A2,PPP1R9A,ENAH,MFF,RTN4,SMURF1,HECW2,SEMA6A,ZSWIM6,LRRC4C,NTN4,BCL11B,CAPRIN2,PANK2,SEMA6D,NDEL1,SLITRK6,PARD6B,SLITRK2,MYPN,SLITRK1,SLITRK4,CHODL,CNTN4,DOK6,LAMA1,AGRN,FEZF1,ID2,MEF2C,SEC24B,AR,COL12A1,CRK,CRKL,MEGF9,EP300,FAT1,HAS2,LAMC1,MYH9,PROX1,PXN,RAB1A,STC1,CUL3,ARPC2,C1GALT1C1,AP1AR,HEG1,VWA1,PEAK1,GRHL2,COL21A1,ABL2,JAG1,ARF1,ARSB,ZFHX3,BCL6,BMP2,BMP6,CNR1,CNTN1,NKX2-5,DDX6,EIF4E,ETV5,EYA1,PTK2B,FKBP1B,MTOR,GRID2,HGF,FOXA1,MARK1,NEUROD1,NEUROG1,SERPINI1,MED1,PRKCI,MAPK6,TWF1,PTPRF,PTPRG,REST,ITSN1,SNAP25,SOX2,SOX11,SPOCK1,TCF3,ZEB1,TCF12,THRB,TP73,TSC1,YWHAG,FZD1,SPAG9,DLG5,KLF4,ZNF536,LRIG2,MAGI2,CPEB3,ZHX2,SETX,KDM4C,RAP1GAP2,MINAR1,DENND5A,CAMSAP2,CRTC1,HEY2,MMD,ZMYND8,FBXO7,FGF20,ANKRD1,DLL1,MYLIP,SOX8,ASAP1,CCDC88A,TENM3,TMEM30A,ZC4H2,EIF4ENIF1,CAMK1D,MIB1,CREB3L2,ZNF804A,CSMD3,SLC39A12,SDK1,ALKAL2,JAK2,JUN,NCAM2,PLP1,MAPK8IP3,TNFRSF21,APC,KLF5,CYLD,EPS8,GRIN2B,HTT,KIT,MAP4,MYO10,PODXL,RAB5A,RALA,RDX,TGFBR1,EVI5,HOMER1,ATG5,TENM1,SPRY2,TBC1D8,DZIP1,FAM98A,RAB8B,ANLN,FNBP1L,TBC1D22B,VPS35,ODF2L,TBC1D14,ATG3,TBC1D15,NAV3,TBC1D16,E2F1,MAN2A1,SERPINE2,PPP1CC,RHEB,VEGFC,SLC25A12,RGS14,WASF3,CLSTN1,SPEN,LRRTM2,LRRN1,NKX6-2,ZNF488,LINGO2,LRRTM3,ADD1,PDE3A,RFX3,AKAP6,TRIB1,BMP10,ANGPT1,LYST,DUSP1,GPR183,S1PR1,FGF7,FGF10,VEGFD,NRG1,HMGB1,CCN1,ITGA9,CCN3,PDE4B,PDE4D,PDGFB,PDGFRA,PTPN2,SLC12A2,TGFB2,THBS1,TMSB4X,VCAM1,FGF18,CXCL14,DOCK4,RALBP1,SMOC2,WNK1,TAFA4,MOSPD2 |
| 1_Member | GO Biological Processes | GO:0032990 | cell part morphogenesis | -47.51309675 | -43.236 | 209/685 | 25,60,102,288,323,351,387,473,655,659,960,998,1009,1123,1385,1400,1499,1501,1600,1808,1826,1857,1946,1956,2017,2043,2044,2045,2048,2115,2263,2297,2332,2335,2534,2549,2625,2668,2736,2737,2823,2885,2887,3688,3800,3984,4038,4076,4131,4133,4205,4534,4628,4684,4734,4756,4781,4803,4851,4897,4915,4929,5048,5063,5168,5291,5295,5306,5361,5362,5530,5578,5588,5594,5604,5649,5727,5728,5789,5797,5803,5911,6091,6092,6093,6239,6383,6477,6642,6654,6683,6709,6711,6722,6857,6860,7074,7101,7155,7204,7224,7337,7430,7436,7465,7533,7852,8013,8239,8301,8408,8609,8633,8650,8660,8766,8828,8829,8851,8936,8976,9037,9201,9211,9252,9331,9334,9353,9369,9378,9448,9693,9699,9706,9723,9798,9839,10006,10048,10087,10152,10160,10458,10479,10507,10631,10752,10787,10818,10890,10939,11127,11141,11252,22854,22871,22903,22941,22999,23011,23072,23077,23098,23114,23189,23242,23327,23394,23543,23603,23767,23768,25942,26039,26053,26999,27020,27023,27255,30011,50618,51761,55607,55740,56947,57142,57154,57520,57556,57688,57689,59277,64919,65981,80025,80031,81565,84189,84612,84631,84665,114798,139065,140578,152330,220164,284217,375790,389549 | ABL1,ACTB,ADAM10,ANK3,APBB2,APP,RHOA,RERE,BMP7,BMPR2,CD44,CDC42,CDH11,CHN1,CREB1,CRMP1,CTNNB1,CTNND2,DAB1,DPYSL2,DSCAM,DVL3,EFNA5,EGFR,CTTN,EPHA4,EPHA5,EPHA7,EPHB2,ETV1,FGFR2,FOXD1,FMR1,FN1,FYN,GAB1,GATA3,GDNF,GLI2,GLI3,GPM6A,GRB2,GRB10,ITGB1,KIF5C,LIMK1,LRP4,CAPRIN1,MAP1B,MAP2,MEF2A,MTM1,MYH10,NCAM1,NEDD4,NEO1,NFIB,NGF,NOTCH1,NRCAM,NTRK2,NR4A2,PAFAH1B1,PAK3,ENPP2,PIK3CB,PIK3R1,PITPNA,PLXNA1,PLXNA2,PPP3CA,PRKCA,PRKCQ,MAPK1,MAP2K1,RELN,PTCH1,PTEN,PTPRD,PTPRM,PTPRZ1,RAP2A,ROBO1,ROBO2,ROCK1,RREB1,SDC2,SIAH1,SNX1,SOS1,SPAST,SPTAN1,SPTBN1,SRF,SYT1,SYT4,TIAM1,NR2E1,TOP2B,TRIO,TRPC5,UBE3A,EZR,VLDLR,WEE1,YWHAH,CXCR4,NR4A3,USP9X,PICALM,ULK1,KLF7,UNC5C,NUMB,IRS2,RAB11A,NRP2,NRP1,CDK5R1,WASF1,WASL,SEMA5A,DCLK1,LGI1,RPS6KA5,B4GALT6,B4GALT5,SLIT2,NRXN3,NRXN1,MAP4K4,RAPGEF2,RIMS2,ULK2,SEMA3E,IST1,ZEB2,ABI1,RANBP9,CERT1,ABI2,FARP1,BAIAP2,SLC9A6,SEMA4D,POSTN,CHL1,NCKAP1,FRS2,RAB10,AFG3L2,KIF3A,IL1RAPL1,PACSIN2,NTNG1,NLGN1,BTBD3,SHANK2,RIMS1,RAB21,HECW1,MYCBP2,SARM1,NFASC,KANK1,COBL,NEDD4L,ADNP,RBFOX2,CORO1C,FLRT3,FLRT2,SIN3A,SS18L1,AUTS2,CYFIP2,NPTN,FOXB1,CNTN6,SH3KBP1,ITSN2,ATP8A2,PPP1R9A,ENAH,MFF,RTN4,SMURF1,HECW2,SEMA6A,ZSWIM6,LRRC4C,NTN4,BCL11B,CAPRIN2,PANK2,SEMA6D,NDEL1,SLITRK6,PARD6B,SLITRK2,MYPN,SLITRK1,SLITRK4,CHODL,CNTN4,DOK6,LAMA1,AGRN,FEZF1 |
| 1_Member | GO Biological Processes | GO:0048858 | cell projection morphogenesis | -47.25738463 | -43.236 | 205/666 | 25,60,102,288,323,351,387,473,655,659,960,998,1009,1123,1385,1400,1499,1501,1600,1808,1826,1857,1946,1956,2017,2043,2044,2045,2048,2115,2263,2297,2332,2335,2534,2549,2625,2668,2736,2737,2823,2885,2887,3688,3800,3984,4038,4076,4131,4133,4205,4628,4684,4734,4756,4781,4803,4851,4897,4915,4929,5048,5063,5168,5291,5295,5306,5361,5362,5530,5578,5588,5594,5604,5649,5727,5728,5789,5797,5803,5911,6091,6092,6093,6239,6383,6477,6642,6654,6683,6709,6711,6722,6857,6860,7074,7101,7155,7204,7224,7337,7430,7436,7465,7533,7852,8013,8239,8301,8408,8609,8633,8650,8660,8766,8828,8829,8851,8936,8976,9037,9201,9211,9252,9331,9334,9353,9369,9378,9448,9693,9699,9706,9723,9798,9839,10006,10048,10152,10160,10458,10479,10507,10631,10752,10787,10818,10890,10939,11127,11141,11252,22854,22871,22903,22941,22999,23011,23072,23077,23098,23114,23189,23242,23327,23394,23543,23603,23767,23768,25942,26039,26053,26999,27020,27023,27255,30011,50618,51761,55607,55740,57142,57154,57520,57556,57688,57689,59277,64919,65981,80031,81565,84189,84612,84631,84665,114798,139065,140578,152330,220164,284217,375790,389549 | ABL1,ACTB,ADAM10,ANK3,APBB2,APP,RHOA,RERE,BMP7,BMPR2,CD44,CDC42,CDH11,CHN1,CREB1,CRMP1,CTNNB1,CTNND2,DAB1,DPYSL2,DSCAM,DVL3,EFNA5,EGFR,CTTN,EPHA4,EPHA5,EPHA7,EPHB2,ETV1,FGFR2,FOXD1,FMR1,FN1,FYN,GAB1,GATA3,GDNF,GLI2,GLI3,GPM6A,GRB2,GRB10,ITGB1,KIF5C,LIMK1,LRP4,CAPRIN1,MAP1B,MAP2,MEF2A,MYH10,NCAM1,NEDD4,NEO1,NFIB,NGF,NOTCH1,NRCAM,NTRK2,NR4A2,PAFAH1B1,PAK3,ENPP2,PIK3CB,PIK3R1,PITPNA,PLXNA1,PLXNA2,PPP3CA,PRKCA,PRKCQ,MAPK1,MAP2K1,RELN,PTCH1,PTEN,PTPRD,PTPRM,PTPRZ1,RAP2A,ROBO1,ROBO2,ROCK1,RREB1,SDC2,SIAH1,SNX1,SOS1,SPAST,SPTAN1,SPTBN1,SRF,SYT1,SYT4,TIAM1,NR2E1,TOP2B,TRIO,TRPC5,UBE3A,EZR,VLDLR,WEE1,YWHAH,CXCR4,NR4A3,USP9X,PICALM,ULK1,KLF7,UNC5C,NUMB,IRS2,RAB11A,NRP2,NRP1,CDK5R1,WASF1,WASL,SEMA5A,DCLK1,LGI1,RPS6KA5,B4GALT6,B4GALT5,SLIT2,NRXN3,NRXN1,MAP4K4,RAPGEF2,RIMS2,ULK2,SEMA3E,IST1,ZEB2,ABI1,RANBP9,ABI2,FARP1,BAIAP2,SLC9A6,SEMA4D,POSTN,CHL1,NCKAP1,FRS2,RAB10,AFG3L2,KIF3A,IL1RAPL1,PACSIN2,NTNG1,NLGN1,BTBD3,SHANK2,RIMS1,RAB21,HECW1,MYCBP2,SARM1,NFASC,KANK1,COBL,NEDD4L,ADNP,RBFOX2,CORO1C,FLRT3,FLRT2,SIN3A,SS18L1,AUTS2,CYFIP2,NPTN,FOXB1,CNTN6,SH3KBP1,ITSN2,ATP8A2,PPP1R9A,ENAH,RTN4,SMURF1,HECW2,SEMA6A,ZSWIM6,LRRC4C,NTN4,BCL11B,CAPRIN2,SEMA6D,NDEL1,SLITRK6,PARD6B,SLITRK2,MYPN,SLITRK1,SLITRK4,CHODL,CNTN4,DOK6,LAMA1,AGRN,FEZF1 |
| 1_Member | GO Biological Processes | GO:0120039 | plasma membrane bounded cell projection morphogenesis | -47.10916093 | -43.236 | 204/662 | 25,60,102,288,323,351,387,473,655,659,960,998,1009,1123,1385,1400,1499,1501,1600,1808,1826,1857,1946,1956,2017,2043,2044,2045,2048,2115,2263,2297,2332,2335,2534,2549,2625,2668,2736,2737,2823,2885,2887,3688,3800,3984,4038,4076,4131,4133,4205,4628,4684,4734,4756,4781,4803,4851,4897,4915,4929,5048,5063,5168,5291,5295,5306,5361,5362,5530,5578,5588,5594,5604,5649,5727,5728,5789,5797,5803,5911,6091,6092,6093,6239,6383,6477,6642,6654,6683,6709,6711,6722,6857,6860,7074,7101,7155,7204,7224,7337,7430,7436,7465,7533,7852,8013,8239,8301,8408,8609,8633,8650,8660,8766,8828,8829,8851,8936,8976,9037,9201,9211,9252,9331,9334,9353,9369,9378,9448,9693,9699,9706,9723,9798,9839,10006,10048,10152,10160,10458,10479,10507,10631,10752,10787,10818,10890,10939,11127,11141,22854,22871,22903,22941,22999,23011,23072,23077,23098,23114,23189,23242,23327,23394,23543,23603,23767,23768,25942,26039,26053,26999,27020,27023,27255,30011,50618,51761,55607,55740,57142,57154,57520,57556,57688,57689,59277,64919,65981,80031,81565,84189,84612,84631,84665,114798,139065,140578,152330,220164,284217,375790,389549 | ABL1,ACTB,ADAM10,ANK3,APBB2,APP,RHOA,RERE,BMP7,BMPR2,CD44,CDC42,CDH11,CHN1,CREB1,CRMP1,CTNNB1,CTNND2,DAB1,DPYSL2,DSCAM,DVL3,EFNA5,EGFR,CTTN,EPHA4,EPHA5,EPHA7,EPHB2,ETV1,FGFR2,FOXD1,FMR1,FN1,FYN,GAB1,GATA3,GDNF,GLI2,GLI3,GPM6A,GRB2,GRB10,ITGB1,KIF5C,LIMK1,LRP4,CAPRIN1,MAP1B,MAP2,MEF2A,MYH10,NCAM1,NEDD4,NEO1,NFIB,NGF,NOTCH1,NRCAM,NTRK2,NR4A2,PAFAH1B1,PAK3,ENPP2,PIK3CB,PIK3R1,PITPNA,PLXNA1,PLXNA2,PPP3CA,PRKCA,PRKCQ,MAPK1,MAP2K1,RELN,PTCH1,PTEN,PTPRD,PTPRM,PTPRZ1,RAP2A,ROBO1,ROBO2,ROCK1,RREB1,SDC2,SIAH1,SNX1,SOS1,SPAST,SPTAN1,SPTBN1,SRF,SYT1,SYT4,TIAM1,NR2E1,TOP2B,TRIO,TRPC5,UBE3A,EZR,VLDLR,WEE1,YWHAH,CXCR4,NR4A3,USP9X,PICALM,ULK1,KLF7,UNC5C,NUMB,IRS2,RAB11A,NRP2,NRP1,CDK5R1,WASF1,WASL,SEMA5A,DCLK1,LGI1,RPS6KA5,B4GALT6,B4GALT5,SLIT2,NRXN3,NRXN1,MAP4K4,RAPGEF2,RIMS2,ULK2,SEMA3E,IST1,ZEB2,ABI1,RANBP9,ABI2,FARP1,BAIAP2,SLC9A6,SEMA4D,POSTN,CHL1,NCKAP1,FRS2,RAB10,AFG3L2,KIF3A,IL1RAPL1,NTNG1,NLGN1,BTBD3,SHANK2,RIMS1,RAB21,HECW1,MYCBP2,SARM1,NFASC,KANK1,COBL,NEDD4L,ADNP,RBFOX2,CORO1C,FLRT3,FLRT2,SIN3A,SS18L1,AUTS2,CYFIP2,NPTN,FOXB1,CNTN6,SH3KBP1,ITSN2,ATP8A2,PPP1R9A,ENAH,RTN4,SMURF1,HECW2,SEMA6A,ZSWIM6,LRRC4C,NTN4,BCL11B,CAPRIN2,SEMA6D,NDEL1,SLITRK6,PARD6B,SLITRK2,MYPN,SLITRK1,SLITRK4,CHODL,CNTN4,DOK6,LAMA1,AGRN,FEZF1 |
| 1_Member | GO Biological Processes | GO:0048812 | neuron projection morphogenesis | -45.08107335 | -41.333 | 198/648 | 25,60,102,288,323,351,387,473,655,659,998,1009,1123,1385,1400,1499,1501,1600,1808,1826,1857,1946,1956,2017,2043,2044,2045,2048,2115,2263,2297,2332,2335,2534,2549,2625,2668,2736,2737,2823,2885,2887,3688,3800,3984,4038,4076,4131,4133,4205,4628,4684,4734,4756,4781,4803,4851,4897,4915,4929,5048,5063,5291,5295,5306,5361,5362,5530,5578,5588,5594,5604,5649,5727,5728,5789,5797,5803,5911,6091,6092,6093,6383,6477,6654,6683,6709,6711,6722,6857,6860,7074,7101,7155,7204,7224,7337,7430,7436,7465,7533,7852,8013,8239,8301,8408,8609,8633,8650,8660,8766,8828,8829,8851,8936,8976,9037,9201,9211,9252,9331,9334,9353,9369,9378,9448,9693,9699,9706,9723,9798,9839,10006,10048,10152,10160,10458,10479,10507,10631,10752,10787,10818,10890,10939,11127,11141,22854,22871,22903,22941,22999,23011,23072,23077,23098,23114,23242,23327,23394,23543,23767,23768,25942,26039,26053,26999,27020,27023,27255,30011,50618,51761,55607,55740,57142,57154,57520,57556,57688,57689,59277,64919,65981,80031,81565,84189,84612,84631,84665,114798,139065,140578,152330,220164,284217,375790,389549 | ABL1,ACTB,ADAM10,ANK3,APBB2,APP,RHOA,RERE,BMP7,BMPR2,CDC42,CDH11,CHN1,CREB1,CRMP1,CTNNB1,CTNND2,DAB1,DPYSL2,DSCAM,DVL3,EFNA5,EGFR,CTTN,EPHA4,EPHA5,EPHA7,EPHB2,ETV1,FGFR2,FOXD1,FMR1,FN1,FYN,GAB1,GATA3,GDNF,GLI2,GLI3,GPM6A,GRB2,GRB10,ITGB1,KIF5C,LIMK1,LRP4,CAPRIN1,MAP1B,MAP2,MEF2A,MYH10,NCAM1,NEDD4,NEO1,NFIB,NGF,NOTCH1,NRCAM,NTRK2,NR4A2,PAFAH1B1,PAK3,PIK3CB,PIK3R1,PITPNA,PLXNA1,PLXNA2,PPP3CA,PRKCA,PRKCQ,MAPK1,MAP2K1,RELN,PTCH1,PTEN,PTPRD,PTPRM,PTPRZ1,RAP2A,ROBO1,ROBO2,ROCK1,SDC2,SIAH1,SOS1,SPAST,SPTAN1,SPTBN1,SRF,SYT1,SYT4,TIAM1,NR2E1,TOP2B,TRIO,TRPC5,UBE3A,EZR,VLDLR,WEE1,YWHAH,CXCR4,NR4A3,USP9X,PICALM,ULK1,KLF7,UNC5C,NUMB,IRS2,RAB11A,NRP2,NRP1,CDK5R1,WASF1,WASL,SEMA5A,DCLK1,LGI1,RPS6KA5,B4GALT6,B4GALT5,SLIT2,NRXN3,NRXN1,MAP4K4,RAPGEF2,RIMS2,ULK2,SEMA3E,IST1,ZEB2,ABI1,RANBP9,ABI2,FARP1,BAIAP2,SLC9A6,SEMA4D,POSTN,CHL1,NCKAP1,FRS2,RAB10,AFG3L2,KIF3A,IL1RAPL1,NTNG1,NLGN1,BTBD3,SHANK2,RIMS1,RAB21,HECW1,MYCBP2,SARM1,NFASC,COBL,NEDD4L,ADNP,RBFOX2,FLRT3,FLRT2,SIN3A,SS18L1,AUTS2,CYFIP2,NPTN,FOXB1,CNTN6,SH3KBP1,ITSN2,ATP8A2,PPP1R9A,ENAH,RTN4,SMURF1,HECW2,SEMA6A,ZSWIM6,LRRC4C,NTN4,BCL11B,CAPRIN2,SEMA6D,NDEL1,SLITRK6,PARD6B,SLITRK2,MYPN,SLITRK1,SLITRK4,CHODL,CNTN4,DOK6,LAMA1,AGRN,FEZF1 |
| 1_Member | GO Biological Processes | GO:0048667 | cell morphogenesis involved in neuron differentiation | -44.70047075 | -41.049 | 185/583 | 25,60,102,288,323,351,387,473,655,659,998,1009,1123,1385,1400,1501,1600,1808,1826,1946,2017,2043,2044,2045,2048,2115,2263,2297,2332,2335,2534,2549,2625,2668,2736,2737,2885,2887,3398,3688,3800,3984,4038,4076,4131,4133,4205,4208,4628,4684,4734,4756,4781,4803,4851,4897,4915,4929,5048,5063,5291,5295,5306,5361,5362,5530,5578,5588,5594,5604,5649,5727,5728,5789,5797,5803,5911,6091,6092,6383,6477,6654,6683,6709,6711,6722,7074,7101,7155,7204,7224,7337,7430,7436,7533,7852,8013,8239,8301,8408,8609,8633,8650,8660,8766,8828,8829,8851,8976,9037,9201,9211,9252,9331,9334,9353,9369,9378,9693,9706,9723,9798,9839,10006,10048,10152,10160,10427,10458,10479,10507,10752,10818,10890,10939,11127,11141,22854,22871,22903,22941,23011,23072,23077,23098,23114,23242,23327,23394,23543,23767,23768,25942,26039,26053,26999,27020,27023,27255,30011,51761,55607,55740,57142,57520,57556,57688,57689,59277,64919,65981,80031,81565,84189,84612,84631,84665,114798,139065,140578,152330,220164,284217,375790,389549 | ABL1,ACTB,ADAM10,ANK3,APBB2,APP,RHOA,RERE,BMP7,BMPR2,CDC42,CDH11,CHN1,CREB1,CRMP1,CTNND2,DAB1,DPYSL2,DSCAM,EFNA5,CTTN,EPHA4,EPHA5,EPHA7,EPHB2,ETV1,FGFR2,FOXD1,FMR1,FN1,FYN,GAB1,GATA3,GDNF,GLI2,GLI3,GRB2,GRB10,ID2,ITGB1,KIF5C,LIMK1,LRP4,CAPRIN1,MAP1B,MAP2,MEF2A,MEF2C,MYH10,NCAM1,NEDD4,NEO1,NFIB,NGF,NOTCH1,NRCAM,NTRK2,NR4A2,PAFAH1B1,PAK3,PIK3CB,PIK3R1,PITPNA,PLXNA1,PLXNA2,PPP3CA,PRKCA,PRKCQ,MAPK1,MAP2K1,RELN,PTCH1,PTEN,PTPRD,PTPRM,PTPRZ1,RAP2A,ROBO1,ROBO2,SDC2,SIAH1,SOS1,SPAST,SPTAN1,SPTBN1,SRF,TIAM1,NR2E1,TOP2B,TRIO,TRPC5,UBE3A,EZR,VLDLR,YWHAH,CXCR4,NR4A3,USP9X,PICALM,ULK1,KLF7,UNC5C,NUMB,IRS2,RAB11A,NRP2,NRP1,CDK5R1,WASL,SEMA5A,DCLK1,LGI1,RPS6KA5,B4GALT6,B4GALT5,SLIT2,NRXN3,NRXN1,RAPGEF2,ULK2,SEMA3E,IST1,ZEB2,ABI1,RANBP9,ABI2,FARP1,SEC24B,BAIAP2,SLC9A6,SEMA4D,CHL1,FRS2,RAB10,AFG3L2,KIF3A,IL1RAPL1,NTNG1,NLGN1,BTBD3,SHANK2,RAB21,HECW1,MYCBP2,SARM1,NFASC,COBL,NEDD4L,ADNP,RBFOX2,FLRT3,FLRT2,SIN3A,SS18L1,AUTS2,CYFIP2,NPTN,FOXB1,CNTN6,SH3KBP1,ATP8A2,PPP1R9A,ENAH,RTN4,HECW2,SEMA6A,ZSWIM6,LRRC4C,NTN4,BCL11B,CAPRIN2,SEMA6D,NDEL1,SLITRK6,PARD6B,SLITRK2,MYPN,SLITRK1,SLITRK4,CHODL,CNTN4,DOK6,LAMA1,AGRN,FEZF1 |
| 1_Member | GO Biological Processes | GO:0000904 | cell morphogenesis involved in differentiation | -43.99825432 | -40.426 | 214/745 | 25,60,102,288,323,351,367,387,473,655,659,998,1009,1123,1303,1385,1398,1399,1400,1499,1501,1600,1808,1826,1946,1955,2017,2033,2043,2044,2045,2048,2115,2195,2263,2297,2332,2335,2534,2549,2625,2668,2736,2737,2885,2887,3037,3398,3688,3800,3915,3984,4038,4076,4131,4133,4205,4208,4627,4628,4684,4734,4756,4781,4803,4851,4897,4915,4929,5048,5063,5291,5295,5306,5361,5362,5530,5578,5588,5594,5604,5629,5649,5727,5728,5789,5797,5803,5829,5861,5911,6091,6092,6093,6239,6383,6477,6654,6683,6709,6711,6722,6781,7074,7101,7155,7204,7224,7337,7430,7436,7533,7852,8013,8239,8301,8408,8452,8609,8633,8650,8660,8766,8828,8829,8851,8976,9037,9201,9211,9252,9331,9334,9353,9369,9378,9693,9706,9723,9798,9839,10006,10048,10109,10152,10160,10427,10458,10479,10507,10631,10752,10818,10890,10939,11127,11141,22854,22871,22903,22941,23011,23072,23077,23098,23114,23189,23242,23327,23394,23543,23603,23767,23768,25942,26039,26053,26999,27020,27023,27255,29071,30011,51761,55435,55607,55740,57142,57493,57520,57556,57688,57689,59277,64856,64919,65981,79834,79977,80031,81565,81578,84189,84612,84631,84665,114798,139065,140578,152330,220164,284217,375790,389549 | ABL1,ACTB,ADAM10,ANK3,APBB2,APP,AR,RHOA,RERE,BMP7,BMPR2,CDC42,CDH11,CHN1,COL12A1,CREB1,CRK,CRKL,CRMP1,CTNNB1,CTNND2,DAB1,DPYSL2,DSCAM,EFNA5,MEGF9,CTTN,EP300,EPHA4,EPHA5,EPHA7,EPHB2,ETV1,FAT1,FGFR2,FOXD1,FMR1,FN1,FYN,GAB1,GATA3,GDNF,GLI2,GLI3,GRB2,GRB10,HAS2,ID2,ITGB1,KIF5C,LAMC1,LIMK1,LRP4,CAPRIN1,MAP1B,MAP2,MEF2A,MEF2C,MYH9,MYH10,NCAM1,NEDD4,NEO1,NFIB,NGF,NOTCH1,NRCAM,NTRK2,NR4A2,PAFAH1B1,PAK3,PIK3CB,PIK3R1,PITPNA,PLXNA1,PLXNA2,PPP3CA,PRKCA,PRKCQ,MAPK1,MAP2K1,PROX1,RELN,PTCH1,PTEN,PTPRD,PTPRM,PTPRZ1,PXN,RAB1A,RAP2A,ROBO1,ROBO2,ROCK1,RREB1,SDC2,SIAH1,SOS1,SPAST,SPTAN1,SPTBN1,SRF,STC1,TIAM1,NR2E1,TOP2B,TRIO,TRPC5,UBE3A,EZR,VLDLR,YWHAH,CXCR4,NR4A3,USP9X,PICALM,ULK1,CUL3,KLF7,UNC5C,NUMB,IRS2,RAB11A,NRP2,NRP1,CDK5R1,WASL,SEMA5A,DCLK1,LGI1,RPS6KA5,B4GALT6,B4GALT5,SLIT2,NRXN3,NRXN1,RAPGEF2,ULK2,SEMA3E,IST1,ZEB2,ABI1,RANBP9,ARPC2,ABI2,FARP1,SEC24B,BAIAP2,SLC9A6,SEMA4D,POSTN,CHL1,FRS2,RAB10,AFG3L2,KIF3A,IL1RAPL1,NTNG1,NLGN1,BTBD3,SHANK2,RAB21,HECW1,MYCBP2,SARM1,NFASC,KANK1,COBL,NEDD4L,ADNP,RBFOX2,CORO1C,FLRT3,FLRT2,SIN3A,SS18L1,AUTS2,CYFIP2,NPTN,FOXB1,CNTN6,C1GALT1C1,SH3KBP1,ATP8A2,AP1AR,PPP1R9A,ENAH,RTN4,HEG1,HECW2,SEMA6A,ZSWIM6,LRRC4C,NTN4,VWA1,BCL11B,CAPRIN2,PEAK1,GRHL2,SEMA6D,NDEL1,COL21A1,SLITRK6,PARD6B,SLITRK2,MYPN,SLITRK1,SLITRK4,CHODL,CNTN4,DOK6,LAMA1,AGRN,FEZF1 |
| 1_Member | GO Biological Processes | GO:0045664 | regulation of neuron differentiation | -41.26656265 | -37.819 | 193/656 | 25,27,102,182,351,375,387,411,463,604,650,654,655,659,1123,1268,1272,1398,1399,1400,1482,1600,1656,1808,1826,1857,1946,1977,2017,2033,2043,2045,2048,2119,2138,2185,2281,2332,2335,2475,2534,2625,2736,2737,2895,3082,3169,3398,3984,4038,4076,4131,4133,4139,4208,4734,4760,4762,4803,4851,4897,4915,5048,5063,5274,5361,5362,5469,5530,5584,5597,5604,5629,5649,5728,5756,5789,5792,5793,5911,5978,6091,6092,6093,6383,6453,6616,6657,6664,6695,6722,6857,6860,6929,6935,6938,7068,7074,7101,7161,7224,7248,7337,7436,7532,7533,7852,8321,8766,8829,8851,9037,9043,9231,9314,9353,9448,9693,9699,9706,9723,9745,9798,9839,9860,9863,10152,10458,10507,11141,22849,22871,22882,22941,22999,23011,23064,23072,23077,23081,23098,23108,23189,23242,23251,23258,23271,23327,23373,23394,23493,23531,23613,25793,25942,26039,26281,27020,27063,28514,29116,30812,50618,50807,51761,55607,55704,55714,55754,55906,56478,57118,57142,57154,57520,57534,57556,57688,57689,64764,64919,65981,80031,81565,91752,114788,114798,140578,152330,221074,221935,285016,389549 | ABL1,ABL2,ADAM10,JAG1,APP,ARF1,RHOA,ARSB,ZFHX3,BCL6,BMP2,BMP6,BMP7,BMPR2,CHN1,CNR1,CNTN1,CRK,CRKL,CRMP1,NKX2-5,DAB1,DDX6,DPYSL2,DSCAM,DVL3,EFNA5,EIF4E,CTTN,EP300,EPHA4,EPHA7,EPHB2,ETV5,EYA1,PTK2B,FKBP1B,FMR1,FN1,MTOR,FYN,GATA3,GLI2,GLI3,GRID2,HGF,FOXA1,ID2,LIMK1,LRP4,CAPRIN1,MAP1B,MAP2,MARK1,MEF2C,NEDD4,NEUROD1,NEUROG1,NGF,NOTCH1,NRCAM,NTRK2,PAFAH1B1,PAK3,SERPINI1,PLXNA1,PLXNA2,MED1,PPP3CA,PRKCI,MAPK6,MAP2K1,PROX1,RELN,PTEN,TWF1,PTPRD,PTPRF,PTPRG,RAP2A,REST,ROBO1,ROBO2,ROCK1,SDC2,ITSN1,SNAP25,SOX2,SOX11,SPOCK1,SRF,SYT1,SYT4,TCF3,ZEB1,TCF12,THRB,TIAM1,NR2E1,TP73,TRPC5,TSC1,UBE3A,VLDLR,YWHAG,YWHAH,CXCR4,FZD1,RAB11A,NRP1,CDK5R1,SEMA5A,SPAG9,DLG5,KLF4,SLIT2,MAP4K4,RAPGEF2,RIMS2,ULK2,SEMA3E,ZNF536,IST1,ZEB2,LRIG2,MAGI2,ABI2,BAIAP2,SEMA4D,IL1RAPL1,CPEB3,NLGN1,ZHX2,SHANK2,RIMS1,RAB21,SETX,HECW1,MYCBP2,KDM4C,SARM1,RAP1GAP2,KANK1,COBL,MINAR1,DENND5A,CAMSAP2,NEDD4L,CRTC1,ADNP,HEY2,MMD,ZMYND8,FBXO7,SIN3A,SS18L1,FGF20,NPTN,ANKRD1,DLL1,MYLIP,SOX8,ITSN2,ASAP1,ATP8A2,PPP1R9A,CCDC88A,TENM3,TMEM30A,ZC4H2,EIF4ENIF1,CAMK1D,RTN4,SMURF1,HECW2,MIB1,SEMA6A,ZSWIM6,LRRC4C,CREB3L2,BCL11B,CAPRIN2,SEMA6D,NDEL1,ZNF804A,CSMD3,SLITRK1,CHODL,CNTN4,SLC39A12,SDK1,ALKAL2,FEZF1 |
| 1_Member | GO Biological Processes | GO:0061564 | axon development | -39.31101606 | -35.915 | 163/514 | 25,60,288,323,351,387,655,659,1009,1123,1268,1385,1400,1600,1808,1826,1946,2017,2043,2044,2045,2048,2115,2263,2281,2297,2335,2534,2549,2625,2668,2736,2737,2885,2887,3688,3717,3725,3800,3984,4038,4131,4133,4628,4684,4685,4756,4781,4803,4851,4897,4915,4929,5048,5063,5291,5295,5306,5354,5361,5362,5578,5588,5594,5604,5649,5727,5728,5792,5797,5803,6091,6092,6477,6654,6683,6709,6711,6722,7074,7101,7155,7204,7224,7430,7436,7852,8013,8239,8301,8408,8609,8633,8650,8660,8766,8828,8829,8851,9037,9201,9211,9252,9314,9331,9334,9353,9369,9378,9706,9723,9798,9839,9860,10048,10458,10479,10507,10752,10818,10890,10939,11127,22854,23011,23077,23114,23162,23242,23271,23394,23767,23768,25942,26053,26999,27020,27023,27242,27255,30011,51761,55740,57142,57556,57688,57689,59277,64919,80031,81565,84189,84612,84631,84665,114798,139065,140578,152330,220164,284217,375790,389549 | ABL1,ACTB,ANK3,APBB2,APP,RHOA,BMP7,BMPR2,CDH11,CHN1,CNR1,CREB1,CRMP1,DAB1,DPYSL2,DSCAM,EFNA5,CTTN,EPHA4,EPHA5,EPHA7,EPHB2,ETV1,FGFR2,FKBP1B,FOXD1,FN1,FYN,GAB1,GATA3,GDNF,GLI2,GLI3,GRB2,GRB10,ITGB1,JAK2,JUN,KIF5C,LIMK1,LRP4,MAP1B,MAP2,MYH10,NCAM1,NCAM2,NEO1,NFIB,NGF,NOTCH1,NRCAM,NTRK2,NR4A2,PAFAH1B1,PAK3,PIK3CB,PIK3R1,PITPNA,PLP1,PLXNA1,PLXNA2,PRKCA,PRKCQ,MAPK1,MAP2K1,RELN,PTCH1,PTEN,PTPRF,PTPRM,PTPRZ1,ROBO1,ROBO2,SIAH1,SOS1,SPAST,SPTAN1,SPTBN1,SRF,TIAM1,NR2E1,TOP2B,TRIO,TRPC5,EZR,VLDLR,CXCR4,NR4A3,USP9X,PICALM,ULK1,KLF7,UNC5C,NUMB,IRS2,RAB11A,NRP2,NRP1,CDK5R1,SEMA5A,DCLK1,LGI1,RPS6KA5,KLF4,B4GALT6,B4GALT5,SLIT2,NRXN3,NRXN1,ULK2,SEMA3E,IST1,ZEB2,LRIG2,RANBP9,BAIAP2,SLC9A6,SEMA4D,CHL1,FRS2,RAB10,AFG3L2,KIF3A,NTNG1,RAB21,MYCBP2,NFASC,MAPK8IP3,COBL,CAMSAP2,ADNP,FLRT3,FLRT2,SIN3A,AUTS2,CYFIP2,NPTN,FOXB1,TNFRSF21,CNTN6,SH3KBP1,ATP8A2,ENAH,RTN4,SEMA6A,ZSWIM6,LRRC4C,NTN4,BCL11B,SEMA6D,NDEL1,SLITRK6,PARD6B,SLITRK2,MYPN,SLITRK1,SLITRK4,CHODL,CNTN4,DOK6,LAMA1,AGRN,FEZF1 |
| 1_Member | GO Biological Processes | GO:0007409 | axonogenesis | -37.40375811 | -34.132 | 151/468 | 25,60,288,323,351,387,655,659,1009,1123,1385,1400,1600,1808,1826,1946,2017,2043,2044,2045,2048,2115,2263,2297,2335,2534,2549,2625,2668,2736,2737,2885,2887,3688,3800,3984,4038,4131,4133,4628,4684,4756,4781,4803,4851,4897,4915,4929,5048,5063,5291,5295,5306,5361,5362,5578,5588,5594,5604,5649,5727,5728,5797,5803,6091,6092,6477,6654,6683,6709,6711,6722,7074,7101,7155,7204,7224,7430,7436,7852,8013,8239,8301,8408,8609,8633,8650,8660,8766,8828,8829,8851,9037,9201,9211,9252,9331,9334,9353,9369,9378,9706,9723,9798,9839,10048,10458,10479,10507,10752,10818,10890,10939,11127,22854,23011,23077,23114,23242,23394,23767,23768,25942,26053,26999,27020,27023,27255,30011,51761,55740,57142,57556,57688,57689,59277,64919,80031,81565,84189,84612,84631,84665,114798,139065,140578,152330,220164,284217,375790,389549 | ABL1,ACTB,ANK3,APBB2,APP,RHOA,BMP7,BMPR2,CDH11,CHN1,CREB1,CRMP1,DAB1,DPYSL2,DSCAM,EFNA5,CTTN,EPHA4,EPHA5,EPHA7,EPHB2,ETV1,FGFR2,FOXD1,FN1,FYN,GAB1,GATA3,GDNF,GLI2,GLI3,GRB2,GRB10,ITGB1,KIF5C,LIMK1,LRP4,MAP1B,MAP2,MYH10,NCAM1,NEO1,NFIB,NGF,NOTCH1,NRCAM,NTRK2,NR4A2,PAFAH1B1,PAK3,PIK3CB,PIK3R1,PITPNA,PLXNA1,PLXNA2,PRKCA,PRKCQ,MAPK1,MAP2K1,RELN,PTCH1,PTEN,PTPRM,PTPRZ1,ROBO1,ROBO2,SIAH1,SOS1,SPAST,SPTAN1,SPTBN1,SRF,TIAM1,NR2E1,TOP2B,TRIO,TRPC5,EZR,VLDLR,CXCR4,NR4A3,USP9X,PICALM,ULK1,KLF7,UNC5C,NUMB,IRS2,RAB11A,NRP2,NRP1,CDK5R1,SEMA5A,DCLK1,LGI1,RPS6KA5,B4GALT6,B4GALT5,SLIT2,NRXN3,NRXN1,ULK2,SEMA3E,IST1,ZEB2,RANBP9,BAIAP2,SLC9A6,SEMA4D,CHL1,FRS2,RAB10,AFG3L2,KIF3A,NTNG1,RAB21,MYCBP2,NFASC,COBL,ADNP,FLRT3,FLRT2,SIN3A,AUTS2,CYFIP2,NPTN,FOXB1,CNTN6,SH3KBP1,ATP8A2,ENAH,RTN4,SEMA6A,ZSWIM6,LRRC4C,NTN4,BCL11B,SEMA6D,NDEL1,SLITRK6,PARD6B,SLITRK2,MYPN,SLITRK1,SLITRK4,CHODL,CNTN4,DOK6,LAMA1,AGRN,FEZF1 |
| 1_Member | GO Biological Processes | GO:0031344 | regulation of cell projection organization | -36.12020309 | -32.884 | 193/708 | 25,27,102,324,375,387,411,655,659,688,960,998,1123,1268,1272,1398,1399,1400,1540,1600,1808,1826,1857,1946,2017,2033,2043,2045,2048,2059,2185,2281,2332,2335,2475,2534,2625,2823,2895,2904,3064,3082,3815,3984,4038,4076,4131,4133,4134,4139,4208,4651,4734,4803,4897,4915,5048,5063,5168,5274,5361,5362,5420,5530,5584,5597,5604,5649,5728,5756,5789,5792,5793,5868,5898,5911,5962,6091,6092,6239,6383,6453,6616,6695,6722,6857,6860,7046,7074,7101,7224,7248,7337,7430,7436,7533,7813,7852,8321,8766,8829,8851,8976,9037,9231,9314,9353,9448,9456,9474,9693,9699,9706,9723,9798,9839,9860,9863,10109,10152,10178,10253,10458,10507,10787,11138,11141,22849,22871,22873,22941,22999,23011,23064,23072,23077,23098,23108,23189,23242,23251,23258,23271,23327,23373,23394,23603,23613,25793,25940,25942,26039,26053,27020,27063,29116,50618,50807,51761,51762,54443,54874,55607,55633,55704,55714,55737,55754,57118,57142,57154,57489,57520,57533,57556,57688,57689,64422,64764,64786,65981,80031,81565,89795,91752,114788,114798,125058,140578,221074,221935,285016,375790 | ABL1,ABL2,ADAM10,APC,ARF1,RHOA,ARSB,BMP7,BMPR2,KLF5,CD44,CDC42,CHN1,CNR1,CNTN1,CRK,CRKL,CRMP1,CYLD,DAB1,DPYSL2,DSCAM,DVL3,EFNA5,CTTN,EP300,EPHA4,EPHA7,EPHB2,EPS8,PTK2B,FKBP1B,FMR1,FN1,MTOR,FYN,GATA3,GPM6A,GRID2,GRIN2B,HTT,HGF,KIT,LIMK1,LRP4,CAPRIN1,MAP1B,MAP2,MAP4,MARK1,MEF2C,MYO10,NEDD4,NGF,NRCAM,NTRK2,PAFAH1B1,PAK3,ENPP2,SERPINI1,PLXNA1,PLXNA2,PODXL,PPP3CA,PRKCI,MAPK6,MAP2K1,RELN,PTEN,TWF1,PTPRD,PTPRF,PTPRG,RAB5A,RALA,RAP2A,RDX,ROBO1,ROBO2,RREB1,SDC2,ITSN1,SNAP25,SPOCK1,SRF,SYT1,SYT4,TGFBR1,TIAM1,NR2E1,TRPC5,TSC1,UBE3A,EZR,VLDLR,YWHAH,EVI5,CXCR4,FZD1,RAB11A,NRP1,CDK5R1,WASL,SEMA5A,DLG5,KLF4,SLIT2,MAP4K4,HOMER1,ATG5,RAPGEF2,RIMS2,ULK2,SEMA3E,IST1,ZEB2,LRIG2,MAGI2,ARPC2,ABI2,TENM1,SPRY2,BAIAP2,SEMA4D,NCKAP1,TBC1D8,IL1RAPL1,CPEB3,NLGN1,DZIP1,SHANK2,RIMS1,RAB21,SETX,HECW1,MYCBP2,SARM1,RAP1GAP2,KANK1,COBL,MINAR1,DENND5A,CAMSAP2,NEDD4L,CRTC1,ADNP,CORO1C,ZMYND8,FBXO7,FAM98A,SIN3A,SS18L1,AUTS2,NPTN,ANKRD1,MYLIP,ITSN2,ASAP1,ATP8A2,RAB8B,ANLN,FNBP1L,PPP1R9A,TBC1D22B,CCDC88A,TENM3,VPS35,TMEM30A,CAMK1D,RTN4,SMURF1,ODF2L,HECW2,TBC1D14,SEMA6A,ZSWIM6,LRRC4C,ATG3,CREB3L2,TBC1D15,CAPRIN2,SEMA6D,NDEL1,NAV3,ZNF804A,CSMD3,SLITRK1,TBC1D16,CHODL,SLC39A12,SDK1,ALKAL2,AGRN |
| 1_Member | GO Biological Processes | GO:0120035 | regulation of plasma membrane bounded cell projection organization | -35.98960411 | -32.785 | 191/698 | 25,27,102,324,375,387,411,655,659,688,960,998,1123,1268,1272,1398,1399,1400,1540,1600,1808,1826,1857,1946,2017,2033,2043,2045,2048,2059,2185,2281,2332,2335,2475,2534,2625,2823,2895,2904,3064,3082,3815,3984,4038,4076,4131,4133,4134,4139,4208,4651,4734,4803,4897,4915,5048,5063,5168,5274,5361,5362,5420,5530,5584,5597,5604,5649,5728,5756,5789,5792,5793,5868,5898,5911,5962,6091,6092,6239,6383,6453,6616,6695,6722,6857,6860,7046,7074,7101,7224,7248,7337,7430,7436,7533,7813,7852,8321,8766,8829,8851,8976,9037,9231,9314,9353,9448,9456,9474,9693,9699,9706,9723,9798,9839,9860,9863,10109,10152,10178,10458,10507,10787,11138,11141,22849,22871,22873,22941,22999,23011,23064,23072,23077,23098,23108,23189,23242,23251,23258,23271,23327,23373,23394,23603,23613,25793,25940,25942,26039,26053,27020,27063,29116,50618,50807,51761,54443,54874,55607,55633,55704,55714,55737,55754,57118,57142,57154,57489,57520,57533,57556,57688,57689,64422,64764,64786,65981,80031,81565,89795,91752,114788,114798,125058,140578,221074,221935,285016,375790 | ABL1,ABL2,ADAM10,APC,ARF1,RHOA,ARSB,BMP7,BMPR2,KLF5,CD44,CDC42,CHN1,CNR1,CNTN1,CRK,CRKL,CRMP1,CYLD,DAB1,DPYSL2,DSCAM,DVL3,EFNA5,CTTN,EP300,EPHA4,EPHA7,EPHB2,EPS8,PTK2B,FKBP1B,FMR1,FN1,MTOR,FYN,GATA3,GPM6A,GRID2,GRIN2B,HTT,HGF,KIT,LIMK1,LRP4,CAPRIN1,MAP1B,MAP2,MAP4,MARK1,MEF2C,MYO10,NEDD4,NGF,NRCAM,NTRK2,PAFAH1B1,PAK3,ENPP2,SERPINI1,PLXNA1,PLXNA2,PODXL,PPP3CA,PRKCI,MAPK6,MAP2K1,RELN,PTEN,TWF1,PTPRD,PTPRF,PTPRG,RAB5A,RALA,RAP2A,RDX,ROBO1,ROBO2,RREB1,SDC2,ITSN1,SNAP25,SPOCK1,SRF,SYT1,SYT4,TGFBR1,TIAM1,NR2E1,TRPC5,TSC1,UBE3A,EZR,VLDLR,YWHAH,EVI5,CXCR4,FZD1,RAB11A,NRP1,CDK5R1,WASL,SEMA5A,DLG5,KLF4,SLIT2,MAP4K4,HOMER1,ATG5,RAPGEF2,RIMS2,ULK2,SEMA3E,IST1,ZEB2,LRIG2,MAGI2,ARPC2,ABI2,TENM1,BAIAP2,SEMA4D,NCKAP1,TBC1D8,IL1RAPL1,CPEB3,NLGN1,DZIP1,SHANK2,RIMS1,RAB21,SETX,HECW1,MYCBP2,SARM1,RAP1GAP2,KANK1,COBL,MINAR1,DENND5A,CAMSAP2,NEDD4L,CRTC1,ADNP,CORO1C,ZMYND8,FBXO7,FAM98A,SIN3A,SS18L1,AUTS2,NPTN,ANKRD1,MYLIP,ITSN2,ASAP1,ATP8A2,ANLN,FNBP1L,PPP1R9A,TBC1D22B,CCDC88A,TENM3,VPS35,TMEM30A,CAMK1D,RTN4,SMURF1,ODF2L,HECW2,TBC1D14,SEMA6A,ZSWIM6,LRRC4C,ATG3,CREB3L2,TBC1D15,CAPRIN2,SEMA6D,NDEL1,NAV3,ZNF804A,CSMD3,SLITRK1,TBC1D16,CHODL,SLC39A12,SDK1,ALKAL2,AGRN |
| 1_Member | GO Biological Processes | GO:0010975 | regulation of neuron projection development | -32.04273979 | -29.014 | 148/499 | 25,27,102,375,387,411,655,659,1123,1268,1272,1398,1399,1400,1600,1808,1826,1857,1946,2017,2033,2043,2045,2048,2185,2281,2332,2335,2475,2534,2625,2895,3082,3984,4038,4076,4131,4133,4139,4208,4734,4803,4897,4915,5048,5063,5274,5361,5362,5530,5584,5597,5604,5649,5728,5756,5789,5792,5793,5911,6091,6092,6383,6453,6616,6695,6722,6857,6860,7074,7101,7224,7248,7337,7436,7533,7852,8321,8766,8829,8851,9037,9231,9314,9353,9448,9693,9699,9706,9723,9798,9839,9860,9863,10152,10458,10507,11141,22849,22871,22941,22999,23011,23064,23072,23077,23098,23108,23189,23242,23251,23258,23271,23327,23373,23394,23613,25793,25942,26039,27020,27063,29116,50618,50807,51761,55607,55704,55714,55754,57118,57142,57154,57520,57556,57688,57689,64764,65981,80031,81565,91752,114788,114798,140578,221074,221935,285016 | ABL1,ABL2,ADAM10,ARF1,RHOA,ARSB,BMP7,BMPR2,CHN1,CNR1,CNTN1,CRK,CRKL,CRMP1,DAB1,DPYSL2,DSCAM,DVL3,EFNA5,CTTN,EP300,EPHA4,EPHA7,EPHB2,PTK2B,FKBP1B,FMR1,FN1,MTOR,FYN,GATA3,GRID2,HGF,LIMK1,LRP4,CAPRIN1,MAP1B,MAP2,MARK1,MEF2C,NEDD4,NGF,NRCAM,NTRK2,PAFAH1B1,PAK3,SERPINI1,PLXNA1,PLXNA2,PPP3CA,PRKCI,MAPK6,MAP2K1,RELN,PTEN,TWF1,PTPRD,PTPRF,PTPRG,RAP2A,ROBO1,ROBO2,SDC2,ITSN1,SNAP25,SPOCK1,SRF,SYT1,SYT4,TIAM1,NR2E1,TRPC5,TSC1,UBE3A,VLDLR,YWHAH,CXCR4,FZD1,RAB11A,NRP1,CDK5R1,SEMA5A,DLG5,KLF4,SLIT2,MAP4K4,RAPGEF2,RIMS2,ULK2,SEMA3E,IST1,ZEB2,LRIG2,MAGI2,ABI2,BAIAP2,SEMA4D,IL1RAPL1,CPEB3,NLGN1,SHANK2,RIMS1,RAB21,SETX,HECW1,MYCBP2,SARM1,RAP1GAP2,KANK1,COBL,MINAR1,DENND5A,CAMSAP2,NEDD4L,CRTC1,ADNP,ZMYND8,FBXO7,SIN3A,SS18L1,NPTN,ANKRD1,MYLIP,ITSN2,ASAP1,ATP8A2,PPP1R9A,CCDC88A,TENM3,TMEM30A,CAMK1D,RTN4,SMURF1,HECW2,SEMA6A,ZSWIM6,LRRC4C,CREB3L2,CAPRIN2,SEMA6D,NDEL1,ZNF804A,CSMD3,SLITRK1,CHODL,SLC39A12,SDK1,ALKAL2 |
| 1_Member | GO Biological Processes | GO:0051962 | positive regulation of nervous system development | -29.46182277 | -26.509 | 151/541 | 27,375,387,411,604,650,654,655,659,1268,1272,1399,1482,1499,1600,1826,1857,1869,1946,2033,2043,2048,2119,2185,2281,2332,2335,2475,2534,2736,2737,2895,3082,3169,3398,3815,3984,4076,4124,4131,4208,4760,4762,4803,4851,4897,4915,5048,5063,5270,5274,5361,5362,5501,5584,5597,5604,5629,5649,5728,5756,5789,5803,5978,6009,6091,6092,6453,6664,6722,6857,6860,6929,6935,6938,7074,7161,7224,7424,7436,7852,8321,8604,8650,8766,8829,9037,9043,9231,9353,9378,9693,9699,9798,9839,9863,10458,10507,10636,10810,11141,22849,22871,22883,22941,22999,23011,23013,23064,23081,23242,23327,23373,23394,23531,23613,23767,23768,25942,26039,26045,26281,27020,27063,30812,50618,51761,55607,55714,55754,55906,57118,57154,57556,57633,64764,65981,81565,84189,84504,84631,91752,114798,118738,139065,140578,158038,285016,347731,375790,389549 | ABL2,ARF1,RHOA,ARSB,BCL6,BMP2,BMP6,BMP7,BMPR2,CNR1,CNTN1,CRKL,NKX2-5,CTNNB1,DAB1,DSCAM,DVL3,E2F1,EFNA5,EP300,EPHA4,EPHB2,ETV5,PTK2B,FKBP1B,FMR1,FN1,MTOR,FYN,GLI2,GLI3,GRID2,HGF,FOXA1,ID2,KIT,LIMK1,CAPRIN1,MAN2A1,MAP1B,MEF2C,NEUROD1,NEUROG1,NGF,NOTCH1,NRCAM,NTRK2,PAFAH1B1,PAK3,SERPINE2,SERPINI1,PLXNA1,PLXNA2,PPP1CC,PRKCI,MAPK6,MAP2K1,PROX1,RELN,PTEN,TWF1,PTPRD,PTPRZ1,REST,RHEB,ROBO1,ROBO2,ITSN1,SOX11,SRF,SYT1,SYT4,TCF3,ZEB1,TCF12,TIAM1,TP73,TRPC5,VEGFC,VLDLR,CXCR4,FZD1,SLC25A12,NUMB,RAB11A,NRP1,SEMA5A,SPAG9,DLG5,SLIT2,NRXN1,RAPGEF2,RIMS2,IST1,ZEB2,MAGI2,BAIAP2,SEMA4D,RGS14,WASF3,IL1RAPL1,CPEB3,NLGN1,CLSTN1,SHANK2,RIMS1,RAB21,SPEN,SETX,KDM4C,COBL,NEDD4L,CRTC1,ADNP,MMD,ZMYND8,FLRT3,FLRT2,SIN3A,SS18L1,LRRTM2,FGF20,NPTN,ANKRD1,SOX8,ITSN2,ATP8A2,PPP1R9A,TENM3,TMEM30A,ZC4H2,CAMK1D,SMURF1,SEMA6A,LRRN1,CREB3L2,CAPRIN2,NDEL1,SLITRK6,NKX6-2,SLITRK2,ZNF804A,SLITRK1,ZNF488,SLITRK4,CHODL,LINGO2,ALKAL2,LRRTM3,AGRN,FEZF1 |
| 1_Member | GO Biological Processes | GO:0050769 | positive regulation of neurogenesis | -27.85974738 | -25.014 | 136/474 | 27,375,387,411,604,650,654,655,659,1268,1272,1399,1482,1499,1600,1826,1857,1869,1946,2033,2043,2048,2119,2185,2281,2332,2335,2475,2534,2736,2737,3082,3169,3398,3815,3984,4076,4124,4131,4208,4760,4762,4803,4851,4897,4915,5048,5063,5270,5274,5361,5362,5501,5584,5597,5604,5629,5649,5728,5756,5789,5803,5978,6009,6091,6092,6453,6664,6722,6857,6860,6929,6935,6938,7074,7161,7224,7424,7436,7852,8321,8650,8766,8829,9037,9043,9231,9353,9693,9699,9798,9839,9863,10458,10507,10636,11141,22849,22871,22941,22999,23011,23013,23064,23081,23242,23327,23373,23394,23531,23613,25942,26039,26281,27020,27063,30812,50618,51761,55607,55714,55754,55906,57118,57154,57556,64764,65981,81565,84504,91752,114798,118738,140578,285016,389549 | ABL2,ARF1,RHOA,ARSB,BCL6,BMP2,BMP6,BMP7,BMPR2,CNR1,CNTN1,CRKL,NKX2-5,CTNNB1,DAB1,DSCAM,DVL3,E2F1,EFNA5,EP300,EPHA4,EPHB2,ETV5,PTK2B,FKBP1B,FMR1,FN1,MTOR,FYN,GLI2,GLI3,HGF,FOXA1,ID2,KIT,LIMK1,CAPRIN1,MAN2A1,MAP1B,MEF2C,NEUROD1,NEUROG1,NGF,NOTCH1,NRCAM,NTRK2,PAFAH1B1,PAK3,SERPINE2,SERPINI1,PLXNA1,PLXNA2,PPP1CC,PRKCI,MAPK6,MAP2K1,PROX1,RELN,PTEN,TWF1,PTPRD,PTPRZ1,REST,RHEB,ROBO1,ROBO2,ITSN1,SOX11,SRF,SYT1,SYT4,TCF3,ZEB1,TCF12,TIAM1,TP73,TRPC5,VEGFC,VLDLR,CXCR4,FZD1,NUMB,RAB11A,NRP1,SEMA5A,SPAG9,DLG5,SLIT2,RAPGEF2,RIMS2,IST1,ZEB2,MAGI2,BAIAP2,SEMA4D,RGS14,IL1RAPL1,CPEB3,NLGN1,SHANK2,RIMS1,RAB21,SPEN,SETX,KDM4C,COBL,NEDD4L,CRTC1,ADNP,MMD,ZMYND8,SIN3A,SS18L1,FGF20,NPTN,ANKRD1,SOX8,ITSN2,ATP8A2,PPP1R9A,TENM3,TMEM30A,ZC4H2,CAMK1D,SMURF1,SEMA6A,CREB3L2,CAPRIN2,NDEL1,NKX6-2,ZNF804A,SLITRK1,ZNF488,CHODL,ALKAL2,FEZF1 |
| 1_Member | GO Biological Processes | GO:0010720 | positive regulation of cell development | -26.77544756 | -23.981 | 148/553 | 25,27,118,375,387,411,604,650,654,655,659,998,1268,1272,1398,1399,1482,1499,1600,1826,1857,1869,1946,2033,2043,2048,2119,2185,2281,2332,2335,2475,2534,2736,2737,3037,3082,3169,3398,3815,3984,4076,4124,4131,4208,4760,4762,4803,4851,4897,4915,5048,5063,5139,5270,5274,5361,5362,5501,5584,5597,5604,5629,5649,5728,5756,5789,5803,5978,5991,6009,6091,6092,6239,6453,6664,6722,6857,6860,6929,6935,6938,7074,7161,7224,7424,7436,7852,8321,8650,8766,8829,9037,9043,9231,9353,9472,9693,9699,9798,9839,9863,10109,10221,10458,10507,10636,11141,22849,22871,22941,22999,23011,23013,23064,23081,23242,23327,23373,23394,23531,23613,25942,26039,26281,27020,27063,27302,30812,50618,51761,55607,55714,55754,55906,57118,57154,57556,64764,65981,81565,84504,91752,114798,118738,140578,285016,389549 | ABL1,ABL2,ADD1,ARF1,RHOA,ARSB,BCL6,BMP2,BMP6,BMP7,BMPR2,CDC42,CNR1,CNTN1,CRK,CRKL,NKX2-5,CTNNB1,DAB1,DSCAM,DVL3,E2F1,EFNA5,EP300,EPHA4,EPHB2,ETV5,PTK2B,FKBP1B,FMR1,FN1,MTOR,FYN,GLI2,GLI3,HAS2,HGF,FOXA1,ID2,KIT,LIMK1,CAPRIN1,MAN2A1,MAP1B,MEF2C,NEUROD1,NEUROG1,NGF,NOTCH1,NRCAM,NTRK2,PAFAH1B1,PAK3,PDE3A,SERPINE2,SERPINI1,PLXNA1,PLXNA2,PPP1CC,PRKCI,MAPK6,MAP2K1,PROX1,RELN,PTEN,TWF1,PTPRD,PTPRZ1,REST,RFX3,RHEB,ROBO1,ROBO2,RREB1,ITSN1,SOX11,SRF,SYT1,SYT4,TCF3,ZEB1,TCF12,TIAM1,TP73,TRPC5,VEGFC,VLDLR,CXCR4,FZD1,NUMB,RAB11A,NRP1,SEMA5A,SPAG9,DLG5,SLIT2,AKAP6,RAPGEF2,RIMS2,IST1,ZEB2,MAGI2,ARPC2,TRIB1,BAIAP2,SEMA4D,RGS14,IL1RAPL1,CPEB3,NLGN1,SHANK2,RIMS1,RAB21,SPEN,SETX,KDM4C,COBL,NEDD4L,CRTC1,ADNP,MMD,ZMYND8,SIN3A,SS18L1,FGF20,NPTN,ANKRD1,BMP10,SOX8,ITSN2,ATP8A2,PPP1R9A,TENM3,TMEM30A,ZC4H2,CAMK1D,SMURF1,SEMA6A,CREB3L2,CAPRIN2,NDEL1,NKX6-2,ZNF804A,SLITRK1,ZNF488,CHODL,ALKAL2,FEZF1 |
| 1_Member | GO Biological Processes | GO:0045666 | positive regulation of neuron differentiation | -26.21847721 | -23.491 | 114/371 | 27,375,387,411,604,650,654,655,659,1268,1272,1482,1600,1826,1857,1946,2033,2043,2119,2185,2281,2332,2335,2475,2534,2736,3082,3169,3984,4076,4131,4208,4760,4762,4803,4897,4915,5048,5063,5274,5361,5362,5584,5597,5604,5629,5649,5728,5756,5789,5978,6091,6092,6453,6664,6722,6857,6860,6929,6935,6938,7074,7224,7436,7852,8321,8766,8829,9037,9043,9231,9353,9693,9699,9798,9839,9863,10458,10507,11141,22849,22871,22941,22999,23011,23064,23081,23242,23327,23373,23394,23531,23613,25942,26039,26281,27020,27063,50618,51761,55607,55714,55754,55906,57118,57154,64764,65981,81565,91752,114798,140578,285016,389549 | ABL2,ARF1,RHOA,ARSB,BCL6,BMP2,BMP6,BMP7,BMPR2,CNR1,CNTN1,NKX2-5,DAB1,DSCAM,DVL3,EFNA5,EP300,EPHA4,ETV5,PTK2B,FKBP1B,FMR1,FN1,MTOR,FYN,GLI2,HGF,FOXA1,LIMK1,CAPRIN1,MAP1B,MEF2C,NEUROD1,NEUROG1,NGF,NRCAM,NTRK2,PAFAH1B1,PAK3,SERPINI1,PLXNA1,PLXNA2,PRKCI,MAPK6,MAP2K1,PROX1,RELN,PTEN,TWF1,PTPRD,REST,ROBO1,ROBO2,ITSN1,SOX11,SRF,SYT1,SYT4,TCF3,ZEB1,TCF12,TIAM1,TRPC5,VLDLR,CXCR4,FZD1,RAB11A,NRP1,SEMA5A,SPAG9,DLG5,SLIT2,RAPGEF2,RIMS2,IST1,ZEB2,MAGI2,BAIAP2,SEMA4D,IL1RAPL1,CPEB3,NLGN1,SHANK2,RIMS1,RAB21,SETX,KDM4C,COBL,NEDD4L,CRTC1,ADNP,MMD,ZMYND8,SIN3A,SS18L1,FGF20,NPTN,ANKRD1,ITSN2,ATP8A2,PPP1R9A,TENM3,TMEM30A,ZC4H2,CAMK1D,SMURF1,CREB3L2,CAPRIN2,NDEL1,ZNF804A,SLITRK1,CHODL,ALKAL2,FEZF1 |
| 1_Member | GO Biological Processes | GO:0031346 | positive regulation of cell projection organization | -23.7629888 | -21.191 | 112/383 | 27,324,375,387,411,655,659,998,1268,1272,1826,1857,1946,2033,2043,2059,2185,2281,2332,2335,2475,2534,2823,3064,3082,3815,3984,4076,4131,4803,4915,5048,5063,5168,5274,5361,5362,5584,5597,5604,5649,5756,5789,5898,6091,6092,6239,6453,6722,6857,6860,7046,7074,7224,7436,7852,8321,8766,8829,8976,9037,9231,9353,9693,9699,9798,9839,9863,10109,10178,10458,10507,10787,11141,22849,22871,22873,22941,22999,23011,23064,23242,23327,23373,23394,23603,23613,25940,26039,26053,27020,27063,50618,51761,51762,54443,54874,55607,55704,55714,55754,57118,57154,64764,65981,81565,89795,91752,114798,140578,285016,375790 | ABL2,APC,ARF1,RHOA,ARSB,BMP7,BMPR2,CDC42,CNR1,CNTN1,DSCAM,DVL3,EFNA5,EP300,EPHA4,EPS8,PTK2B,FKBP1B,FMR1,FN1,MTOR,FYN,GPM6A,HTT,HGF,KIT,LIMK1,CAPRIN1,MAP1B,NGF,NTRK2,PAFAH1B1,PAK3,ENPP2,SERPINI1,PLXNA1,PLXNA2,PRKCI,MAPK6,MAP2K1,RELN,TWF1,PTPRD,RALA,ROBO1,ROBO2,RREB1,ITSN1,SRF,SYT1,SYT4,TGFBR1,TIAM1,TRPC5,VLDLR,CXCR4,FZD1,RAB11A,NRP1,WASL,SEMA5A,DLG5,SLIT2,RAPGEF2,RIMS2,IST1,ZEB2,MAGI2,ARPC2,TENM1,BAIAP2,SEMA4D,NCKAP1,IL1RAPL1,CPEB3,NLGN1,DZIP1,SHANK2,RIMS1,RAB21,SETX,COBL,NEDD4L,CRTC1,ADNP,CORO1C,ZMYND8,FAM98A,SS18L1,AUTS2,NPTN,ANKRD1,ITSN2,ATP8A2,RAB8B,ANLN,FNBP1L,PPP1R9A,CCDC88A,TENM3,TMEM30A,CAMK1D,SMURF1,CREB3L2,CAPRIN2,NDEL1,NAV3,ZNF804A,SLITRK1,CHODL,ALKAL2,AGRN |
| 1_Member | GO Biological Processes | GO:0007411 | axon guidance | -22.81370034 | -20.302 | 90/276 | 323,351,655,659,1123,1400,1600,1808,1826,1946,2043,2044,2045,2048,2115,2297,2534,2549,2625,2668,2736,2737,2885,2887,3800,4628,4684,4756,4781,4897,5291,5295,5361,5362,5578,5588,5594,5649,5727,5797,6091,6092,6477,6654,6709,6711,7204,7430,7436,7852,8013,8609,8633,8660,8828,8829,8851,9037,9211,9252,9353,9369,9378,9723,10048,10507,10752,10818,11127,22854,23077,23114,23767,23768,26999,27020,27255,30011,55740,57556,57688,59277,64919,80031,84665,152330,220164,284217,375790,389549 | APBB2,APP,BMP7,BMPR2,CHN1,CRMP1,DAB1,DPYSL2,DSCAM,EFNA5,EPHA4,EPHA5,EPHA7,EPHB2,ETV1,FOXD1,FYN,GAB1,GATA3,GDNF,GLI2,GLI3,GRB2,GRB10,KIF5C,MYH10,NCAM1,NEO1,NFIB,NRCAM,PIK3CB,PIK3R1,PLXNA1,PLXNA2,PRKCA,PRKCQ,MAPK1,RELN,PTCH1,PTPRM,ROBO1,ROBO2,SIAH1,SOS1,SPTAN1,SPTBN1,TRIO,EZR,VLDLR,CXCR4,NR4A3,KLF7,UNC5C,IRS2,NRP2,NRP1,CDK5R1,SEMA5A,LGI1,RPS6KA5,SLIT2,NRXN3,NRXN1,SEMA3E,RANBP9,SEMA4D,CHL1,FRS2,KIF3A,NTNG1,MYCBP2,NFASC,FLRT3,FLRT2,CYFIP2,NPTN,CNTN6,SH3KBP1,ENAH,SEMA6A,ZSWIM6,NTN4,BCL11B,SEMA6D,MYPN,CNTN4,DOK6,LAMA1,AGRN,FEZF1 |
| 1_Member | GO Biological Processes | GO:0097485 | neuron projection guidance | -22.68944515 | -20.202 | 90/277 | 323,351,655,659,1123,1400,1600,1808,1826,1946,2043,2044,2045,2048,2115,2297,2534,2549,2625,2668,2736,2737,2885,2887,3800,4628,4684,4756,4781,4897,5291,5295,5361,5362,5578,5588,5594,5649,5727,5797,6091,6092,6477,6654,6709,6711,7204,7430,7436,7852,8013,8609,8633,8660,8828,8829,8851,9037,9211,9252,9353,9369,9378,9723,10048,10507,10752,10818,11127,22854,23077,23114,23767,23768,26999,27020,27255,30011,55740,57556,57688,59277,64919,80031,84665,152330,220164,284217,375790,389549 | APBB2,APP,BMP7,BMPR2,CHN1,CRMP1,DAB1,DPYSL2,DSCAM,EFNA5,EPHA4,EPHA5,EPHA7,EPHB2,ETV1,FOXD1,FYN,GAB1,GATA3,GDNF,GLI2,GLI3,GRB2,GRB10,KIF5C,MYH10,NCAM1,NEO1,NFIB,NRCAM,PIK3CB,PIK3R1,PLXNA1,PLXNA2,PRKCA,PRKCQ,MAPK1,RELN,PTCH1,PTPRM,ROBO1,ROBO2,SIAH1,SOS1,SPTAN1,SPTBN1,TRIO,EZR,VLDLR,CXCR4,NR4A3,KLF7,UNC5C,IRS2,NRP2,NRP1,CDK5R1,SEMA5A,LGI1,RPS6KA5,SLIT2,NRXN3,NRXN1,SEMA3E,RANBP9,SEMA4D,CHL1,FRS2,KIF3A,NTNG1,MYCBP2,NFASC,FLRT3,FLRT2,CYFIP2,NPTN,CNTN6,SH3KBP1,ENAH,SEMA6A,ZSWIM6,NTN4,BCL11B,SEMA6D,MYPN,CNTN4,DOK6,LAMA1,AGRN,FEZF1 |
| 1_Member | GO Biological Processes | GO:0010976 | positive regulation of neuron projection development | -20.97912324 | -18.548 | 88/281 | 27,375,387,411,655,659,1268,1272,1826,1857,1946,2033,2043,2185,2281,2332,2335,2475,2534,3082,3984,4076,4131,4803,4915,5048,5063,5274,5361,5362,5584,5597,5604,5649,5756,5789,6091,6092,6453,6722,6857,6860,7074,7224,7436,7852,8321,8766,8829,9037,9231,9353,9693,9699,9798,9839,9863,10458,10507,11141,22849,22871,22941,22999,23011,23064,23242,23327,23373,23394,23613,26039,27020,27063,50618,51761,55607,55714,55754,57118,57154,64764,65981,81565,91752,114798,140578,285016 | ABL2,ARF1,RHOA,ARSB,BMP7,BMPR2,CNR1,CNTN1,DSCAM,DVL3,EFNA5,EP300,EPHA4,PTK2B,FKBP1B,FMR1,FN1,MTOR,FYN,HGF,LIMK1,CAPRIN1,MAP1B,NGF,NTRK2,PAFAH1B1,PAK3,SERPINI1,PLXNA1,PLXNA2,PRKCI,MAPK6,MAP2K1,RELN,TWF1,PTPRD,ROBO1,ROBO2,ITSN1,SRF,SYT1,SYT4,TIAM1,TRPC5,VLDLR,CXCR4,FZD1,RAB11A,NRP1,SEMA5A,DLG5,SLIT2,RAPGEF2,RIMS2,IST1,ZEB2,MAGI2,BAIAP2,SEMA4D,IL1RAPL1,CPEB3,NLGN1,SHANK2,RIMS1,RAB21,SETX,COBL,NEDD4L,CRTC1,ADNP,ZMYND8,SS18L1,NPTN,ANKRD1,ITSN2,ATP8A2,PPP1R9A,TENM3,TMEM30A,CAMK1D,SMURF1,CREB3L2,CAPRIN2,NDEL1,ZNF804A,SLITRK1,CHODL,ALKAL2 |
| 1_Member | GO Biological Processes | GO:0042330 | taxis | -15.5178962 | -13.346 | 138/647 | 102,284,323,351,387,655,659,1123,1130,1385,1398,1399,1400,1600,1808,1826,1843,1880,1901,1946,2033,2043,2044,2045,2048,2115,2185,2252,2255,2277,2297,2534,2549,2625,2668,2736,2737,2885,2887,3082,3084,3146,3398,3491,3680,3800,3815,4628,4684,4756,4781,4851,4856,4897,5142,5144,5155,5156,5168,5291,5295,5361,5362,5578,5588,5594,5604,5649,5727,5771,5797,5898,6091,6092,6477,6558,6654,6709,6711,7042,7057,7074,7114,7204,7412,7424,7430,7436,7852,8013,8609,8633,8660,8817,8828,8829,8851,9037,9211,9252,9353,9369,9378,9547,9723,9732,10048,10507,10752,10818,10928,11127,22854,23077,23114,23767,23768,26999,27020,27255,30011,55740,57118,57556,57688,59277,64094,64919,65125,80031,84665,151647,152330,158747,220164,284217,375790,389549 | ADAM10,ANGPT1,APBB2,APP,RHOA,BMP7,BMPR2,CHN1,LYST,CREB1,CRK,CRKL,CRMP1,DAB1,DPYSL2,DSCAM,DUSP1,GPR183,S1PR1,EFNA5,EP300,EPHA4,EPHA5,EPHA7,EPHB2,ETV1,PTK2B,FGF7,FGF10,VEGFD,FOXD1,FYN,GAB1,GATA3,GDNF,GLI2,GLI3,GRB2,GRB10,HGF,NRG1,HMGB1,ID2,CCN1,ITGA9,KIF5C,KIT,MYH10,NCAM1,NEO1,NFIB,NOTCH1,CCN3,NRCAM,PDE4B,PDE4D,PDGFB,PDGFRA,ENPP2,PIK3CB,PIK3R1,PLXNA1,PLXNA2,PRKCA,PRKCQ,MAPK1,MAP2K1,RELN,PTCH1,PTPN2,PTPRM,RALA,ROBO1,ROBO2,SIAH1,SLC12A2,SOS1,SPTAN1,SPTBN1,TGFB2,THBS1,TIAM1,TMSB4X,TRIO,VCAM1,VEGFC,EZR,VLDLR,CXCR4,NR4A3,KLF7,UNC5C,IRS2,FGF18,NRP2,NRP1,CDK5R1,SEMA5A,LGI1,RPS6KA5,SLIT2,NRXN3,NRXN1,CXCL14,SEMA3E,DOCK4,RANBP9,SEMA4D,CHL1,FRS2,RALBP1,KIF3A,NTNG1,MYCBP2,NFASC,FLRT3,FLRT2,CYFIP2,NPTN,CNTN6,SH3KBP1,ENAH,CAMK1D,SEMA6A,ZSWIM6,NTN4,SMOC2,BCL11B,WNK1,SEMA6D,MYPN,TAFA4,CNTN4,MOSPD2,DOK6,LAMA1,AGRN,FEZF1 |
| 1_Member | GO Biological Processes | GO:0006935 | chemotaxis | -14.87837744 | -12.771 | 136/645 | 102,284,323,351,387,655,659,1123,1130,1385,1398,1399,1400,1600,1808,1826,1843,1880,1901,1946,2043,2044,2045,2048,2115,2185,2252,2255,2277,2297,2534,2549,2625,2668,2736,2737,2885,2887,3082,3084,3146,3491,3680,3800,3815,4628,4684,4756,4781,4851,4856,4897,5142,5144,5155,5156,5168,5291,5295,5361,5362,5578,5588,5594,5604,5649,5727,5771,5797,5898,6091,6092,6477,6558,6654,6709,6711,7042,7057,7074,7114,7204,7412,7424,7430,7436,7852,8013,8609,8633,8660,8817,8828,8829,8851,9037,9211,9252,9353,9369,9378,9547,9723,9732,10048,10507,10752,10818,10928,11127,22854,23077,23114,23767,23768,26999,27020,27255,30011,55740,57118,57556,57688,59277,64094,64919,65125,80031,84665,151647,152330,158747,220164,284217,375790,389549 | ADAM10,ANGPT1,APBB2,APP,RHOA,BMP7,BMPR2,CHN1,LYST,CREB1,CRK,CRKL,CRMP1,DAB1,DPYSL2,DSCAM,DUSP1,GPR183,S1PR1,EFNA5,EPHA4,EPHA5,EPHA7,EPHB2,ETV1,PTK2B,FGF7,FGF10,VEGFD,FOXD1,FYN,GAB1,GATA3,GDNF,GLI2,GLI3,GRB2,GRB10,HGF,NRG1,HMGB1,CCN1,ITGA9,KIF5C,KIT,MYH10,NCAM1,NEO1,NFIB,NOTCH1,CCN3,NRCAM,PDE4B,PDE4D,PDGFB,PDGFRA,ENPP2,PIK3CB,PIK3R1,PLXNA1,PLXNA2,PRKCA,PRKCQ,MAPK1,MAP2K1,RELN,PTCH1,PTPN2,PTPRM,RALA,ROBO1,ROBO2,SIAH1,SLC12A2,SOS1,SPTAN1,SPTBN1,TGFB2,THBS1,TIAM1,TMSB4X,TRIO,VCAM1,VEGFC,EZR,VLDLR,CXCR4,NR4A3,KLF7,UNC5C,IRS2,FGF18,NRP2,NRP1,CDK5R1,SEMA5A,LGI1,RPS6KA5,SLIT2,NRXN3,NRXN1,CXCL14,SEMA3E,DOCK4,RANBP9,SEMA4D,CHL1,FRS2,RALBP1,KIF3A,NTNG1,MYCBP2,NFASC,FLRT3,FLRT2,CYFIP2,NPTN,CNTN6,SH3KBP1,ENAH,CAMK1D,SEMA6A,ZSWIM6,NTN4,SMOC2,BCL11B,WNK1,SEMA6D,MYPN,TAFA4,CNTN4,MOSPD2,DOK6,LAMA1,AGRN,FEZF1 |
| 2_Summary | GO Cellular Components | GO:0098794 | postsynapse | -43.63693215 | -40.131 | 189/613 | 25,60,102,118,120,163,287,288,320,321,351,375,387,490,547,659,664,747,775,793,998,1007,1272,1305,1399,1499,1501,1600,1739,1740,1756,1812,1977,1994,2017,2035,2043,2045,2048,2059,2066,2185,2332,2475,2534,2554,2557,2558,2565,2742,2743,2823,2891,2895,2898,2903,2904,2911,2915,2917,3064,3188,3688,3708,3717,3736,3739,3745,3746,3747,3756,3759,3781,4038,4131,4133,4134,4208,4628,4734,4897,4905,4915,4986,5062,5063,5100,5142,5170,5332,5501,5530,5577,5594,5727,5728,5813,5868,6009,6453,6529,6538,6546,6616,6654,6695,6711,7074,7248,7402,8001,8087,8301,8440,8502,8661,8766,8828,8829,8851,8898,8936,9201,9228,9231,9455,9456,9568,9743,9829,9863,10006,10152,10160,10243,10458,10564,10636,10810,11141,22849,22866,22871,22883,22941,22986,23208,23229,23236,23345,23362,23373,23613,23673,23767,25897,26045,26115,27020,27253,27445,29102,50807,53616,54477,55327,55607,55737,55906,55914,56899,57120,57142,57534,57689,58489,64506,80059,81831,91752,93664,94056,114798,347731,388336,399694,401190,642938,729993,493,776,1006,1268,1363,2054,2572,3737,5664,6326,6857,8573,8867,9378,9627,9699,9892,10497,22854,22999,23085,23390,27255,53942,115548,134957,223082,440279,7534,9465,1009,1607,2982,3084,5520,5573,5584,5789,6860,7531,8499,8650,9211,10082,10109,10397,11252,64854,81873,84631,139065 | ABL1,ACTB,ADAM10,ADD1,ADD3,AP2B1,ANK2,ANK3,APBA1,APBA2,APP,ARF1,RHOA,ATP2B1,KIF1A,BMPR2,BNIP3,DAGLA,CACNA1C,CALB1,CDC42,CDH9,CNTN1,COL13A1,CRKL,CTNNB1,CTNND2,DAB1,DLG1,DLG2,DMD,DRD1,EIF4E,ELAVL1,CTTN,EPB41,EPHA4,EPHA7,EPHB2,EPS8,ERBB4,PTK2B,FMR1,MTOR,FYN,GABRA1,GABRA4,GABRA5,GABRG1,GLRA2,GLRB,GPM6A,GRIA2,GRID2,GRIK2,GRIN2A,GRIN2B,GRM1,GRM5,GRM7,HTT,HNRNPH2,ITGB1,ITPR1,JAK2,KCNA1,KCNA4,KCNB1,KCNC1,KCNC2,KCNH1,KCNJ2,KCNN2,LRP4,MAP1B,MAP2,MAP4,MEF2C,MYH10,NEDD4,NRCAM,NSF,NTRK2,OPRK1,PAK2,PAK3,PCDH8,PDE4B,PDPK1,PLCB4,PPP1CC,PPP3CA,PRKAR2B,MAPK1,PTCH1,PTEN,PURA,RAB5A,RHEB,ITSN1,SLC6A1,SLC6A11,SLC8A1,SNAP25,SOS1,SPOCK1,SPTBN1,TIAM1,TSC1,UTRN,GLRA3,FXR1,PICALM,NCK2,PKP4,EIF3A,RAB11A,NRP2,NRP1,CDK5R1,MTMR2,WASF1,DCLK1,DLGAP2,DLG5,HOMER2,HOMER1,GABBR2,ARHGAP32,DNAJC6,MAGI2,ABI1,ABI2,FARP1,GPHN,BAIAP2,ARFGEF2,RGS14,WASF3,IL1RAPL1,CPEB3,CNKSR2,NLGN1,CLSTN1,SHANK2,SORCS3,SYT11,ARHGEF9,PLCB1,SYNE1,PSD3,CRTC1,ZMYND8,STX12,FLRT3,RNF19A,LRRTM2,TANC2,NPTN,PCDH17,PCLO,DROSHA,ASAP1,ADAM22,PLEKHA5,LIN7C,PPP1R9A,VPS35,ZC4H2,ERBIN,ANKS1B,GOPC,RTN4,MIB1,LRRC4C,ABHD17C,CPEB1,LRRTM4,NETO2,ZNF804A,CADPS2,SYAP1,SLITRK1,LRRTM3,SHISA6,SHC4,RGS7BP,INSYN2A,SHISA9,ATP2B4,CACNA1D,CDH8,CNR1,CPE,STX2,GAD2,KCNA2,PSEN2,SCN2A,SYT1,CASK,SYNJ1,NRXN1,SNCAIP,RIMS2,SNAP91,UNC13B,NTNG1,RIMS1,ERC1,ZDHHC17,CNTN6,CNTN5,FCHO2,STXBP5,ZNRF2,UNC13C,YWHAZ,AKAP7,CDH11,DGKB,GUCY1A1,NRG1,PPP2R2A,PRKAR1A,PRKCI,PTPRD,SYT4,YWHAE,PPFIA2,NUMB,LGI1,GPC6,ARPC2,NDRG1,PACSIN2,USP46,ARPC5L,SLITRK2,SLITRK4 |
| 2_Member | GO Cellular Components | GO:0098794 | postsynapse | -43.63693215 | -40.131 | 189/613 | 25,60,102,118,120,163,287,288,320,321,351,375,387,490,547,659,664,747,775,793,998,1007,1272,1305,1399,1499,1501,1600,1739,1740,1756,1812,1977,1994,2017,2035,2043,2045,2048,2059,2066,2185,2332,2475,2534,2554,2557,2558,2565,2742,2743,2823,2891,2895,2898,2903,2904,2911,2915,2917,3064,3188,3688,3708,3717,3736,3739,3745,3746,3747,3756,3759,3781,4038,4131,4133,4134,4208,4628,4734,4897,4905,4915,4986,5062,5063,5100,5142,5170,5332,5501,5530,5577,5594,5727,5728,5813,5868,6009,6453,6529,6538,6546,6616,6654,6695,6711,7074,7248,7402,8001,8087,8301,8440,8502,8661,8766,8828,8829,8851,8898,8936,9201,9228,9231,9455,9456,9568,9743,9829,9863,10006,10152,10160,10243,10458,10564,10636,10810,11141,22849,22866,22871,22883,22941,22986,23208,23229,23236,23345,23362,23373,23613,23673,23767,25897,26045,26115,27020,27253,27445,29102,50807,53616,54477,55327,55607,55737,55906,55914,56899,57120,57142,57534,57689,58489,64506,80059,81831,91752,93664,94056,114798,347731,388336,399694,401190,642938,729993 | ABL1,ACTB,ADAM10,ADD1,ADD3,AP2B1,ANK2,ANK3,APBA1,APBA2,APP,ARF1,RHOA,ATP2B1,KIF1A,BMPR2,BNIP3,DAGLA,CACNA1C,CALB1,CDC42,CDH9,CNTN1,COL13A1,CRKL,CTNNB1,CTNND2,DAB1,DLG1,DLG2,DMD,DRD1,EIF4E,ELAVL1,CTTN,EPB41,EPHA4,EPHA7,EPHB2,EPS8,ERBB4,PTK2B,FMR1,MTOR,FYN,GABRA1,GABRA4,GABRA5,GABRG1,GLRA2,GLRB,GPM6A,GRIA2,GRID2,GRIK2,GRIN2A,GRIN2B,GRM1,GRM5,GRM7,HTT,HNRNPH2,ITGB1,ITPR1,JAK2,KCNA1,KCNA4,KCNB1,KCNC1,KCNC2,KCNH1,KCNJ2,KCNN2,LRP4,MAP1B,MAP2,MAP4,MEF2C,MYH10,NEDD4,NRCAM,NSF,NTRK2,OPRK1,PAK2,PAK3,PCDH8,PDE4B,PDPK1,PLCB4,PPP1CC,PPP3CA,PRKAR2B,MAPK1,PTCH1,PTEN,PURA,RAB5A,RHEB,ITSN1,SLC6A1,SLC6A11,SLC8A1,SNAP25,SOS1,SPOCK1,SPTBN1,TIAM1,TSC1,UTRN,GLRA3,FXR1,PICALM,NCK2,PKP4,EIF3A,RAB11A,NRP2,NRP1,CDK5R1,MTMR2,WASF1,DCLK1,DLGAP2,DLG5,HOMER2,HOMER1,GABBR2,ARHGAP32,DNAJC6,MAGI2,ABI1,ABI2,FARP1,GPHN,BAIAP2,ARFGEF2,RGS14,WASF3,IL1RAPL1,CPEB3,CNKSR2,NLGN1,CLSTN1,SHANK2,SORCS3,SYT11,ARHGEF9,PLCB1,SYNE1,PSD3,CRTC1,ZMYND8,STX12,FLRT3,RNF19A,LRRTM2,TANC2,NPTN,PCDH17,PCLO,DROSHA,ASAP1,ADAM22,PLEKHA5,LIN7C,PPP1R9A,VPS35,ZC4H2,ERBIN,ANKS1B,GOPC,RTN4,MIB1,LRRC4C,ABHD17C,CPEB1,LRRTM4,NETO2,ZNF804A,CADPS2,SYAP1,SLITRK1,LRRTM3,SHISA6,SHC4,RGS7BP,INSYN2A,SHISA9 |
| 2_Member | GO Cellular Components | GO:0097060 | synaptic membrane | -28.85370363 | -25.940 | 130/432 | 102,287,288,320,375,490,493,747,775,776,1006,1007,1268,1272,1305,1363,1399,1499,1739,1740,1756,1812,2043,2045,2048,2054,2066,2332,2554,2557,2558,2565,2572,2742,2743,2823,2891,2895,2898,2903,2904,2911,2915,2917,3688,3736,3737,3745,3746,3747,3756,4038,4897,4986,5100,5664,5728,6326,6529,6538,6546,6616,6857,7074,7402,8001,8301,8573,8828,8829,8867,8898,9228,9231,9378,9455,9456,9568,9627,9699,9743,9892,10006,10243,10458,10497,10636,11141,22849,22854,22866,22871,22883,22941,22986,22999,23085,23208,23229,23345,23362,23390,23767,26045,27020,27253,27255,53616,53942,55327,55906,56899,57120,57689,58489,64506,80059,93664,94056,114798,115548,134957,223082,347731,388336,399694,401190,440279,642938,729993 | ADAM10,ANK2,ANK3,APBA1,ARF1,ATP2B1,ATP2B4,DAGLA,CACNA1C,CACNA1D,CDH8,CDH9,CNR1,CNTN1,COL13A1,CPE,CRKL,CTNNB1,DLG1,DLG2,DMD,DRD1,EPHA4,EPHA7,EPHB2,STX2,ERBB4,FMR1,GABRA1,GABRA4,GABRA5,GABRG1,GAD2,GLRA2,GLRB,GPM6A,GRIA2,GRID2,GRIK2,GRIN2A,GRIN2B,GRM1,GRM5,GRM7,ITGB1,KCNA1,KCNA2,KCNB1,KCNC1,KCNC2,KCNH1,LRP4,NRCAM,OPRK1,PCDH8,PSEN2,PTEN,SCN2A,SLC6A1,SLC6A11,SLC8A1,SNAP25,SYT1,TIAM1,UTRN,GLRA3,PICALM,CASK,NRP2,NRP1,SYNJ1,MTMR2,DLGAP2,DLG5,NRXN1,HOMER2,HOMER1,GABBR2,SNCAIP,RIMS2,ARHGAP32,SNAP91,ABI1,GPHN,BAIAP2,UNC13B,RGS14,IL1RAPL1,CPEB3,NTNG1,CNKSR2,NLGN1,CLSTN1,SHANK2,SORCS3,RIMS1,ERC1,SYT11,ARHGEF9,SYNE1,PSD3,ZDHHC17,FLRT3,LRRTM2,NPTN,PCDH17,CNTN6,ADAM22,CNTN5,LIN7C,ZC4H2,ANKS1B,GOPC,LRRC4C,ABHD17C,CPEB1,LRRTM4,CADPS2,SYAP1,SLITRK1,FCHO2,STXBP5,ZNRF2,LRRTM3,SHISA6,SHC4,RGS7BP,UNC13C,INSYN2A,SHISA9 |
| 2_Member | GO Cellular Components | GO:0098984 | neuron to neuron synapse | -26.23327036 | -23.495 | 110/350 | 102,118,120,375,659,664,775,793,1499,1501,1600,1739,1740,2035,2043,2045,2059,2066,2185,2332,2534,2891,2895,2898,2903,2904,2911,2915,2917,3188,3708,3756,4038,4131,4133,4134,4897,4905,4915,5062,5063,5142,5170,5332,5594,5727,6009,6654,6695,6711,6857,7074,7248,7534,8087,8440,8502,8661,8851,8898,9201,9228,9231,9455,9456,9465,9743,9829,9863,10006,10243,10458,10497,10564,10636,22849,22866,22871,22883,22941,22986,23208,23229,23362,23373,25897,26045,26115,27020,27445,29102,53616,54477,55327,55607,55737,56899,57120,57142,57534,57689,58489,64506,81831,114798,347731,388336,401190,642938,729993 | ADAM10,ADD1,ADD3,ARF1,BMPR2,BNIP3,CACNA1C,CALB1,CTNNB1,CTNND2,DAB1,DLG1,DLG2,EPB41,EPHA4,EPHA7,EPS8,ERBB4,PTK2B,FMR1,FYN,GRIA2,GRID2,GRIK2,GRIN2A,GRIN2B,GRM1,GRM5,GRM7,HNRNPH2,ITPR1,KCNH1,LRP4,MAP1B,MAP2,MAP4,NRCAM,NSF,NTRK2,PAK2,PAK3,PDE4B,PDPK1,PLCB4,MAPK1,PTCH1,RHEB,SOS1,SPOCK1,SPTBN1,SYT1,TIAM1,TSC1,YWHAZ,FXR1,NCK2,PKP4,EIF3A,CDK5R1,MTMR2,DCLK1,DLGAP2,DLG5,HOMER2,HOMER1,AKAP7,ARHGAP32,DNAJC6,MAGI2,ABI1,GPHN,BAIAP2,UNC13B,ARFGEF2,RGS14,CPEB3,CNKSR2,NLGN1,CLSTN1,SHANK2,SORCS3,SYT11,ARHGEF9,PSD3,CRTC1,RNF19A,LRRTM2,TANC2,NPTN,PCLO,DROSHA,ADAM22,PLEKHA5,LIN7C,PPP1R9A,VPS35,ANKS1B,GOPC,RTN4,MIB1,LRRC4C,ABHD17C,CPEB1,NETO2,SLITRK1,LRRTM3,SHISA6,RGS7BP,INSYN2A,SHISA9 |
| 2_Member | GO Cellular Components | GO:0098978 | glutamatergic synapse | -25.74442668 | -23.057 | 109/349 | 60,102,163,320,375,387,490,493,793,1006,1009,1268,1607,1739,1740,1812,1977,1994,2043,2045,2048,2059,2066,2185,2475,2534,2823,2895,2898,2903,2911,2982,3084,3688,3717,3736,4628,4897,5062,5063,5100,5332,5501,5520,5530,5573,5577,5584,5789,5813,6326,6453,6616,6711,6857,6860,7074,7531,7534,8087,8499,8650,8766,8828,8829,9211,9378,9455,9456,10082,10109,10397,10458,10497,10636,10810,11141,11252,22854,22866,22871,22883,22986,23236,23373,23767,25897,26045,26115,27020,27253,53616,54477,55327,55607,55737,55914,57689,58489,64854,81873,84631,93664,114798,139065,347731,388336,401190,729993 | ACTB,ADAM10,AP2B1,APBA1,ARF1,RHOA,ATP2B1,ATP2B4,CALB1,CDH8,CDH11,CNR1,DGKB,DLG1,DLG2,DRD1,EIF4E,ELAVL1,EPHA4,EPHA7,EPHB2,EPS8,ERBB4,PTK2B,MTOR,FYN,GPM6A,GRID2,GRIK2,GRIN2A,GRM1,GUCY1A1,NRG1,ITGB1,JAK2,KCNA1,MYH10,NRCAM,PAK2,PAK3,PCDH8,PLCB4,PPP1CC,PPP2R2A,PPP3CA,PRKAR1A,PRKAR2B,PRKCI,PTPRD,PURA,SCN2A,ITSN1,SNAP25,SPTBN1,SYT1,SYT4,TIAM1,YWHAE,YWHAZ,FXR1,PPFIA2,NUMB,RAB11A,NRP2,NRP1,LGI1,NRXN1,HOMER2,HOMER1,GPC6,ARPC2,NDRG1,BAIAP2,UNC13B,RGS14,WASF3,IL1RAPL1,PACSIN2,NTNG1,CNKSR2,NLGN1,CLSTN1,SORCS3,PLCB1,CRTC1,FLRT3,RNF19A,LRRTM2,TANC2,NPTN,PCDH17,ADAM22,PLEKHA5,LIN7C,PPP1R9A,VPS35,ERBIN,LRRC4C,ABHD17C,USP46,ARPC5L,SLITRK2,CADPS2,SLITRK1,SLITRK4,LRRTM3,SHISA6,RGS7BP,SHISA9 |
| 2_Member | GO Cellular Components | GO:0032279 | asymmetric synapse | -25.17253157 | -22.512 | 104/328 | 102,118,120,375,659,664,775,1499,1501,1600,1739,1740,2035,2043,2045,2059,2066,2185,2332,2534,2891,2895,2898,2903,2904,2911,2915,2917,3188,3708,3756,4038,4131,4133,4134,4897,4905,4915,5062,5063,5142,5170,5332,5594,5727,6009,6654,6695,6711,7074,7248,8087,8440,8502,8661,8851,8898,9201,9228,9231,9455,9456,9743,9829,9863,10006,10243,10458,10564,10636,22849,22866,22871,22883,22941,22986,23208,23229,23362,23373,26045,26115,27020,27445,29102,53616,54477,55327,55607,55737,56899,57120,57142,57534,57689,58489,64506,81831,114798,347731,388336,401190,642938,729993 | ADAM10,ADD1,ADD3,ARF1,BMPR2,BNIP3,CACNA1C,CTNNB1,CTNND2,DAB1,DLG1,DLG2,EPB41,EPHA4,EPHA7,EPS8,ERBB4,PTK2B,FMR1,FYN,GRIA2,GRID2,GRIK2,GRIN2A,GRIN2B,GRM1,GRM5,GRM7,HNRNPH2,ITPR1,KCNH1,LRP4,MAP1B,MAP2,MAP4,NRCAM,NSF,NTRK2,PAK2,PAK3,PDE4B,PDPK1,PLCB4,MAPK1,PTCH1,RHEB,SOS1,SPOCK1,SPTBN1,TIAM1,TSC1,FXR1,NCK2,PKP4,EIF3A,CDK5R1,MTMR2,DCLK1,DLGAP2,DLG5,HOMER2,HOMER1,ARHGAP32,DNAJC6,MAGI2,ABI1,GPHN,BAIAP2,ARFGEF2,RGS14,CPEB3,CNKSR2,NLGN1,CLSTN1,SHANK2,SORCS3,SYT11,ARHGEF9,PSD3,CRTC1,LRRTM2,TANC2,NPTN,PCLO,DROSHA,ADAM22,PLEKHA5,LIN7C,PPP1R9A,VPS35,ANKS1B,GOPC,RTN4,MIB1,LRRC4C,ABHD17C,CPEB1,NETO2,SLITRK1,LRRTM3,SHISA6,RGS7BP,INSYN2A,SHISA9 |
| 2_Member | GO Cellular Components | GO:0014069 | postsynaptic density | -24.42532702 | -21.807 | 102/324 | 102,118,120,375,659,664,775,1499,1501,1600,1739,1740,2035,2043,2045,2059,2066,2185,2332,2534,2891,2895,2898,2903,2904,2911,2915,3188,3708,3756,4038,4131,4133,4134,4897,4905,4915,5062,5063,5142,5170,5332,5594,5727,6009,6654,6695,6711,7074,7248,8087,8440,8502,8661,8851,8898,9201,9228,9231,9455,9456,9743,9829,9863,10006,10243,10458,10636,22849,22866,22871,22883,22941,22986,23208,23229,23362,23373,26045,26115,27020,27445,29102,53616,54477,55327,55607,55737,56899,57120,57142,57534,57689,58489,64506,81831,114798,347731,388336,401190,642938,729993 | ADAM10,ADD1,ADD3,ARF1,BMPR2,BNIP3,CACNA1C,CTNNB1,CTNND2,DAB1,DLG1,DLG2,EPB41,EPHA4,EPHA7,EPS8,ERBB4,PTK2B,FMR1,FYN,GRIA2,GRID2,GRIK2,GRIN2A,GRIN2B,GRM1,GRM5,HNRNPH2,ITPR1,KCNH1,LRP4,MAP1B,MAP2,MAP4,NRCAM,NSF,NTRK2,PAK2,PAK3,PDE4B,PDPK1,PLCB4,MAPK1,PTCH1,RHEB,SOS1,SPOCK1,SPTBN1,TIAM1,TSC1,FXR1,NCK2,PKP4,EIF3A,CDK5R1,MTMR2,DCLK1,DLGAP2,DLG5,HOMER2,HOMER1,ARHGAP32,DNAJC6,MAGI2,ABI1,GPHN,BAIAP2,RGS14,CPEB3,CNKSR2,NLGN1,CLSTN1,SHANK2,SORCS3,SYT11,ARHGEF9,PSD3,CRTC1,LRRTM2,TANC2,NPTN,PCLO,DROSHA,ADAM22,PLEKHA5,LIN7C,PPP1R9A,VPS35,ANKS1B,GOPC,RTN4,MIB1,LRRC4C,ABHD17C,CPEB1,NETO2,SLITRK1,LRRTM3,SHISA6,RGS7BP,INSYN2A,SHISA9 |
| 2_Member | GO Cellular Components | GO:0099572 | postsynaptic specialization | -23.48729721 | -20.956 | 105/348 | 102,118,120,375,659,664,775,1499,1501,1600,1739,1740,2035,2043,2045,2059,2066,2185,2332,2534,2554,2557,2558,2891,2895,2898,2903,2904,2911,2915,3188,3708,3756,4038,4131,4133,4134,4897,4905,4915,5062,5063,5142,5170,5332,5594,5727,6009,6654,6695,6711,7074,7248,8087,8440,8502,8661,8851,8898,9201,9228,9231,9455,9456,9743,9829,9863,10006,10243,10458,10636,22849,22866,22871,22883,22941,22986,23208,23229,23362,23373,26045,26115,27020,27445,29102,53616,54477,55327,55607,55737,56899,57120,57142,57534,57689,58489,64506,81831,114798,347731,388336,401190,642938,729993 | ADAM10,ADD1,ADD3,ARF1,BMPR2,BNIP3,CACNA1C,CTNNB1,CTNND2,DAB1,DLG1,DLG2,EPB41,EPHA4,EPHA7,EPS8,ERBB4,PTK2B,FMR1,FYN,GABRA1,GABRA4,GABRA5,GRIA2,GRID2,GRIK2,GRIN2A,GRIN2B,GRM1,GRM5,HNRNPH2,ITPR1,KCNH1,LRP4,MAP1B,MAP2,MAP4,NRCAM,NSF,NTRK2,PAK2,PAK3,PDE4B,PDPK1,PLCB4,MAPK1,PTCH1,RHEB,SOS1,SPOCK1,SPTBN1,TIAM1,TSC1,FXR1,NCK2,PKP4,EIF3A,CDK5R1,MTMR2,DCLK1,DLGAP2,DLG5,HOMER2,HOMER1,ARHGAP32,DNAJC6,MAGI2,ABI1,GPHN,BAIAP2,RGS14,CPEB3,CNKSR2,NLGN1,CLSTN1,SHANK2,SORCS3,SYT11,ARHGEF9,PSD3,CRTC1,LRRTM2,TANC2,NPTN,PCLO,DROSHA,ADAM22,PLEKHA5,LIN7C,PPP1R9A,VPS35,ANKS1B,GOPC,RTN4,MIB1,LRRC4C,ABHD17C,CPEB1,NETO2,SLITRK1,LRRTM3,SHISA6,RGS7BP,INSYN2A,SHISA9 |
| 2_Member | GO Cellular Components | GO:0045211 | postsynaptic membrane | -19.3161863 | -16.958 | 93/323 | 102,287,288,375,747,775,1007,1272,1305,1399,1499,1739,1740,1756,1812,2043,2045,2048,2066,2332,2554,2557,2558,2565,2742,2743,2891,2895,2898,2903,2904,2911,2915,2917,3736,3745,3746,3747,3756,4897,4986,5100,5728,6529,6538,6546,7074,7402,8001,8301,8828,8829,9228,9231,9455,9456,9568,9743,10006,10243,10636,11141,22849,22866,22871,22883,22941,22986,23229,23345,23362,23767,26045,27020,27253,53616,55327,55906,56899,57120,57689,58489,64506,80059,93664,94056,114798,347731,388336,399694,401190,642938,729993 | ADAM10,ANK2,ANK3,ARF1,DAGLA,CACNA1C,CDH9,CNTN1,COL13A1,CRKL,CTNNB1,DLG1,DLG2,DMD,DRD1,EPHA4,EPHA7,EPHB2,ERBB4,FMR1,GABRA1,GABRA4,GABRA5,GABRG1,GLRA2,GLRB,GRIA2,GRID2,GRIK2,GRIN2A,GRIN2B,GRM1,GRM5,GRM7,KCNA1,KCNB1,KCNC1,KCNC2,KCNH1,NRCAM,OPRK1,PCDH8,PTEN,SLC6A1,SLC6A11,SLC8A1,TIAM1,UTRN,GLRA3,PICALM,NRP2,NRP1,DLGAP2,DLG5,HOMER2,HOMER1,GABBR2,ARHGAP32,ABI1,GPHN,RGS14,IL1RAPL1,CPEB3,CNKSR2,NLGN1,CLSTN1,SHANK2,SORCS3,ARHGEF9,SYNE1,PSD3,FLRT3,LRRTM2,NPTN,PCDH17,ADAM22,LIN7C,ZC4H2,ANKS1B,GOPC,LRRC4C,ABHD17C,CPEB1,LRRTM4,CADPS2,SYAP1,SLITRK1,LRRTM3,SHISA6,SHC4,RGS7BP,INSYN2A,SHISA9 |
| 3_Summary | GO Molecular Functions | GO:0001012 | RNA polymerase II regulatory region DNA binding | -38.76054538 | -35.410 | 213/794 | 60,196,367,406,463,571,604,639,688,860,988,1106,1316,1385,1386,1482,1602,1871,1874,1958,2004,2005,2023,2033,2078,2113,2115,2117,2119,2120,2294,2295,2297,2313,2353,2494,2625,2626,2649,2736,2737,2908,3015,3096,3131,3170,3190,3192,3200,3202,3206,3212,3217,3234,3660,3720,3725,4005,4010,4086,4091,4094,4097,4205,4208,4212,4223,4286,4335,4520,4601,4603,4613,4661,4760,4762,4773,4774,4775,4779,4781,4783,4851,4862,4929,5079,5090,5241,5308,5451,5469,5629,5813,5978,5991,6095,6239,6256,6299,6304,6421,6656,6664,6670,6722,6909,6929,6934,6935,6938,7020,7021,7022,7027,7050,7068,7071,7101,7150,7161,7182,7227,7291,7403,7421,7494,7528,7546,7547,7552,7690,7702,7707,7716,7764,7975,8013,8204,8609,8726,8939,9189,9314,9480,9496,9519,9555,9575,9611,9612,9705,9745,9925,9960,10336,10499,10725,11278,22823,23040,23119,23126,23269,23314,23411,23493,23512,23598,23635,23648,26468,26523,27324,28999,29841,29942,30812,51274,51804,55553,55636,55749,55810,56938,57621,58499,60468,63973,63974,64641,64764,64864,64919,79733,79776,84159,84333,84504,84678,84969,93166,93986,121536,130497,132660,152485,153222,253738,359948,376940,389058,389549,431707,1387,1869,6928,7003,9658,3169,3198,5316,5326,6657,9839,10274,23036,23435,57822,79977,473,546,905,1164,1499,1655,1956,2308,2332,2965,3184,4300,4676,5563,5901,5927,5976,6602,7155,8019,8028,8289,8467,8535,8725,8841,9126,9425,10194,10463,10735,10891,10951,11051,23054,23081,23272,23394,25842,25942,26053,26057,50809,51317,51322,51780,54454,54737,55777,56916,57616,80816,80854,83990,84289,84733,128553,221037 | ACTB,AHR,AR,ARNTL,ZFHX3,BACH1,BCL6,PRDM1,KLF5,RUNX2,CDC5L,CHD2,KLF6,CREB1,ATF2,NKX2-5,DACH1,E2F3,E2F4,EGR1,ELK3,ELK4,ENO1,EP300,ERG,ETS1,ETV1,ETV3,ETV5,ETV6,FOXF1,FOXF2,FOXD1,FLI1,FOS,NR5A2,GATA3,GATA4,NR6A1,GLI2,GLI3,NR3C1,H2AZ1,HIVEP1,HLF,FOXA2,HNRNPK,HNRNPU,HOXA3,HOXA5,HOXA10,HOXB2,HOXB7,HOXD8,IRF2,JARID2,JUN,LMO2,LMX1B,SMAD1,SMAD6,MAF,MAFG,MEF2A,MEF2C,MEIS2,MEOX2,MITF,MNT,MTF1,MXI1,MYBL1,MYCN,MYT1,NEUROD1,NEUROG1,NFATC2,NFIA,NFATC3,NFE2L1,NFIB,NFIL3,NOTCH1,NPAS2,NR4A2,PAX5,PBX3,PGR,PITX2,POU2F1,MED1,PROX1,PURA,REST,RFX3,RORA,RREB1,RXRA,SALL1,SATB1,SFPQ,SOX1,SOX11,SP3,SRF,TBX2,TCF3,TCF7L2,ZEB1,TCF12,TFAP2A,TFAP2B,TFAP2C,TFDP1,TGIF1,THRB,KLF10,NR2E1,TOP1,TP73,NR2C2,TRPS1,TWIST1,KDM6A,VDR,XBP1,YY1,ZIC2,ZIC3,ZNF711,ZNF131,ZNF143,ZNF148,VEZF1,ZNF217,MAFK,NR4A3,NRIP1,KLF7,EED,FUBP3,ZBED1,KLF4,ONECUT2,TBX4,TBPL1,MACROH2A1,CLOCK,NCOR1,NCOR2,ST18,ZNF536,ZBTB5,USP3,PCGF3,NCOA2,NFAT5,KLF12,MTF2,MYT1L,HIC2,POGZ,MGA,SATB2,SIRT1,HEY2,SUZ12,PATZ1,SSBP2,SSBP3,LHX6,AGO1,TOX3,KLF15,GRHL1,PURG,SOX8,KLF3,SIX4,SOX6,CHD7,CCAR1,FOXJ2,ARNTL2,ZBTB2,ZNF462,BACH2,NEUROG2,NEUROD6,EBF2,CREB3L2,RFX7,BCL11B,E2F8,ZFHX4,ARID5B,PCGF5,NKX6-2,KDM2B,TOX2,PRDM6,FOXP2,AEBP2,OSR1,LIN54,ZNF827,CREBRF,EBF3,IRF2BP2,ZC3H6,SP5,FEZF1,LHX8,CREBBP,E2F1,HNF1B,TEAD1,ZNF516,FOXA1,HOXA1,PKNOX1,PLAGL2,SOX2,ZEB2,STAG1,ZNF292,TARDBP,GRHL3,GRHL2,RERE,ATRX,CCNT2,CKS2,CTNNB1,DDX5,EGFR,FOXO1,FMR1,GTF2H1,HNRNPD,MLLT3,NAP1L4,PRKAA2,RAN,KDM5A,UPF1,SMARCD1,TOP2B,BRD3,MLLT10,ARID1A,SMARCA5,CBX4,URI1,HDAC3,SMC3,CDYL,TSHZ1,SLC30A9,STAG2,PPARGC1A,CBX1,NUDT21,NCOA6,KDM4C,TASOR,ADNP,ASF1A,SIN3A,AUTS2,ANKRD17,HP1BP3,PHF21A,WAC,KDM3B,ATAD2B,MPHOSPH8,MBD5,SMARCAD1,TSHZ3,ASXL3,SETD7,BRIP1,ING5,CBX2,TSHZ2,JMJD1C |
| 3_Member | GO Molecular Functions | GO:0001012 | RNA polymerase II regulatory region DNA binding | -38.76054538 | -35.410 | 213/794 | 60,196,367,406,463,571,604,639,688,860,988,1106,1316,1385,1386,1482,1602,1871,1874,1958,2004,2005,2023,2033,2078,2113,2115,2117,2119,2120,2294,2295,2297,2313,2353,2494,2625,2626,2649,2736,2737,2908,3015,3096,3131,3170,3190,3192,3200,3202,3206,3212,3217,3234,3660,3720,3725,4005,4010,4086,4091,4094,4097,4205,4208,4212,4223,4286,4335,4520,4601,4603,4613,4661,4760,4762,4773,4774,4775,4779,4781,4783,4851,4862,4929,5079,5090,5241,5308,5451,5469,5629,5813,5978,5991,6095,6239,6256,6299,6304,6421,6656,6664,6670,6722,6909,6929,6934,6935,6938,7020,7021,7022,7027,7050,7068,7071,7101,7150,7161,7182,7227,7291,7403,7421,7494,7528,7546,7547,7552,7690,7702,7707,7716,7764,7975,8013,8204,8609,8726,8939,9189,9314,9480,9496,9519,9555,9575,9611,9612,9705,9745,9925,9960,10336,10499,10725,11278,22823,23040,23119,23126,23269,23314,23411,23493,23512,23598,23635,23648,26468,26523,27324,28999,29841,29942,30812,51274,51804,55553,55636,55749,55810,56938,57621,58499,60468,63973,63974,64641,64764,64864,64919,79733,79776,84159,84333,84504,84678,84969,93166,93986,121536,130497,132660,152485,153222,253738,359948,376940,389058,389549,431707 | ACTB,AHR,AR,ARNTL,ZFHX3,BACH1,BCL6,PRDM1,KLF5,RUNX2,CDC5L,CHD2,KLF6,CREB1,ATF2,NKX2-5,DACH1,E2F3,E2F4,EGR1,ELK3,ELK4,ENO1,EP300,ERG,ETS1,ETV1,ETV3,ETV5,ETV6,FOXF1,FOXF2,FOXD1,FLI1,FOS,NR5A2,GATA3,GATA4,NR6A1,GLI2,GLI3,NR3C1,H2AZ1,HIVEP1,HLF,FOXA2,HNRNPK,HNRNPU,HOXA3,HOXA5,HOXA10,HOXB2,HOXB7,HOXD8,IRF2,JARID2,JUN,LMO2,LMX1B,SMAD1,SMAD6,MAF,MAFG,MEF2A,MEF2C,MEIS2,MEOX2,MITF,MNT,MTF1,MXI1,MYBL1,MYCN,MYT1,NEUROD1,NEUROG1,NFATC2,NFIA,NFATC3,NFE2L1,NFIB,NFIL3,NOTCH1,NPAS2,NR4A2,PAX5,PBX3,PGR,PITX2,POU2F1,MED1,PROX1,PURA,REST,RFX3,RORA,RREB1,RXRA,SALL1,SATB1,SFPQ,SOX1,SOX11,SP3,SRF,TBX2,TCF3,TCF7L2,ZEB1,TCF12,TFAP2A,TFAP2B,TFAP2C,TFDP1,TGIF1,THRB,KLF10,NR2E1,TOP1,TP73,NR2C2,TRPS1,TWIST1,KDM6A,VDR,XBP1,YY1,ZIC2,ZIC3,ZNF711,ZNF131,ZNF143,ZNF148,VEZF1,ZNF217,MAFK,NR4A3,NRIP1,KLF7,EED,FUBP3,ZBED1,KLF4,ONECUT2,TBX4,TBPL1,MACROH2A1,CLOCK,NCOR1,NCOR2,ST18,ZNF536,ZBTB5,USP3,PCGF3,NCOA2,NFAT5,KLF12,MTF2,MYT1L,HIC2,POGZ,MGA,SATB2,SIRT1,HEY2,SUZ12,PATZ1,SSBP2,SSBP3,LHX6,AGO1,TOX3,KLF15,GRHL1,PURG,SOX8,KLF3,SIX4,SOX6,CHD7,CCAR1,FOXJ2,ARNTL2,ZBTB2,ZNF462,BACH2,NEUROG2,NEUROD6,EBF2,CREB3L2,RFX7,BCL11B,E2F8,ZFHX4,ARID5B,PCGF5,NKX6-2,KDM2B,TOX2,PRDM6,FOXP2,AEBP2,OSR1,LIN54,ZNF827,CREBRF,EBF3,IRF2BP2,ZC3H6,SP5,FEZF1,LHX8 |
| 3_Member | GO Molecular Functions | GO:0000977 | RNA polymerase II regulatory region sequence-specific DNA binding | -38.25960606 | -34.950 | 211/788 | 60,196,367,406,463,571,604,639,688,860,988,1106,1316,1385,1386,1482,1602,1871,1874,1958,2004,2005,2023,2033,2078,2113,2115,2117,2119,2120,2294,2295,2297,2313,2353,2494,2625,2626,2649,2736,2737,2908,3015,3096,3131,3170,3190,3192,3200,3202,3206,3212,3217,3234,3660,3720,3725,4005,4010,4086,4091,4094,4097,4205,4208,4212,4223,4286,4335,4520,4601,4603,4613,4661,4760,4762,4773,4774,4775,4779,4781,4783,4851,4862,4929,5079,5090,5241,5308,5451,5469,5629,5813,5978,5991,6095,6239,6256,6299,6304,6421,6656,6664,6670,6722,6909,6929,6934,6935,6938,7020,7021,7022,7027,7050,7068,7071,7101,7150,7161,7182,7227,7291,7403,7421,7494,7528,7546,7547,7552,7690,7702,7707,7716,7764,7975,8013,8204,8609,8726,8939,9189,9314,9480,9496,9519,9555,9575,9705,9745,9925,9960,10336,10499,10725,11278,22823,23040,23119,23126,23269,23314,23411,23493,23512,23598,23635,23648,26468,26523,27324,28999,29841,29942,30812,51274,51804,55553,55636,55749,55810,56938,57621,58499,60468,63973,63974,64641,64764,64864,64919,79733,79776,84159,84333,84504,84678,84969,93166,93986,121536,130497,132660,152485,153222,253738,359948,376940,389058,389549,431707 | ACTB,AHR,AR,ARNTL,ZFHX3,BACH1,BCL6,PRDM1,KLF5,RUNX2,CDC5L,CHD2,KLF6,CREB1,ATF2,NKX2-5,DACH1,E2F3,E2F4,EGR1,ELK3,ELK4,ENO1,EP300,ERG,ETS1,ETV1,ETV3,ETV5,ETV6,FOXF1,FOXF2,FOXD1,FLI1,FOS,NR5A2,GATA3,GATA4,NR6A1,GLI2,GLI3,NR3C1,H2AZ1,HIVEP1,HLF,FOXA2,HNRNPK,HNRNPU,HOXA3,HOXA5,HOXA10,HOXB2,HOXB7,HOXD8,IRF2,JARID2,JUN,LMO2,LMX1B,SMAD1,SMAD6,MAF,MAFG,MEF2A,MEF2C,MEIS2,MEOX2,MITF,MNT,MTF1,MXI1,MYBL1,MYCN,MYT1,NEUROD1,NEUROG1,NFATC2,NFIA,NFATC3,NFE2L1,NFIB,NFIL3,NOTCH1,NPAS2,NR4A2,PAX5,PBX3,PGR,PITX2,POU2F1,MED1,PROX1,PURA,REST,RFX3,RORA,RREB1,RXRA,SALL1,SATB1,SFPQ,SOX1,SOX11,SP3,SRF,TBX2,TCF3,TCF7L2,ZEB1,TCF12,TFAP2A,TFAP2B,TFAP2C,TFDP1,TGIF1,THRB,KLF10,NR2E1,TOP1,TP73,NR2C2,TRPS1,TWIST1,KDM6A,VDR,XBP1,YY1,ZIC2,ZIC3,ZNF711,ZNF131,ZNF143,ZNF148,VEZF1,ZNF217,MAFK,NR4A3,NRIP1,KLF7,EED,FUBP3,ZBED1,KLF4,ONECUT2,TBX4,TBPL1,MACROH2A1,CLOCK,ST18,ZNF536,ZBTB5,USP3,PCGF3,NCOA2,NFAT5,KLF12,MTF2,MYT1L,HIC2,POGZ,MGA,SATB2,SIRT1,HEY2,SUZ12,PATZ1,SSBP2,SSBP3,LHX6,AGO1,TOX3,KLF15,GRHL1,PURG,SOX8,KLF3,SIX4,SOX6,CHD7,CCAR1,FOXJ2,ARNTL2,ZBTB2,ZNF462,BACH2,NEUROG2,NEUROD6,EBF2,CREB3L2,RFX7,BCL11B,E2F8,ZFHX4,ARID5B,PCGF5,NKX6-2,KDM2B,TOX2,PRDM6,FOXP2,AEBP2,OSR1,LIN54,ZNF827,CREBRF,EBF3,IRF2BP2,ZC3H6,SP5,FEZF1,LHX8 |
| 3_Member | GO Molecular Functions | GO:0000987 | proximal promoter sequence-specific DNA binding | -29.79373409 | -26.820 | 155/560 | 60,196,367,406,463,639,688,860,1106,1316,1385,1386,1387,1482,1602,1869,1871,1874,2004,2005,2033,2078,2113,2115,2120,2297,2313,2353,2494,2625,2626,2649,2736,2737,2908,3015,3170,3190,3192,3200,3202,3206,3212,3217,3725,4005,4086,4091,4094,4097,4205,4208,4212,4223,4286,4520,4603,4613,4760,4762,4773,4774,4775,4781,4783,4851,4929,5079,5241,5308,5451,5469,5629,5978,5991,6095,6239,6256,6299,6421,6656,6664,6670,6722,6909,6928,6929,6934,6935,6938,7003,7020,7021,7022,7050,7071,7150,7161,7182,7291,7403,7421,7494,7528,7547,7702,7707,7764,7975,8013,8204,8609,8726,8939,9189,9314,9480,9519,9575,9658,9705,9745,9960,10499,10725,23314,23512,23598,23635,23648,26523,27324,28999,29841,51804,55553,55636,55810,56938,57621,63973,63974,64641,64764,64864,64919,79733,79776,84504,84678,84969,93986,121536,253738,389549 | ACTB,AHR,AR,ARNTL,ZFHX3,PRDM1,KLF5,RUNX2,CHD2,KLF6,CREB1,ATF2,CREBBP,NKX2-5,DACH1,E2F1,E2F3,E2F4,ELK3,ELK4,EP300,ERG,ETS1,ETV1,ETV6,FOXD1,FLI1,FOS,NR5A2,GATA3,GATA4,NR6A1,GLI2,GLI3,NR3C1,H2AZ1,FOXA2,HNRNPK,HNRNPU,HOXA3,HOXA5,HOXA10,HOXB2,HOXB7,JUN,LMO2,SMAD1,SMAD6,MAF,MAFG,MEF2A,MEF2C,MEIS2,MEOX2,MITF,MTF1,MYBL1,MYCN,NEUROD1,NEUROG1,NFATC2,NFIA,NFATC3,NFIB,NFIL3,NOTCH1,NR4A2,PAX5,PGR,PITX2,POU2F1,MED1,PROX1,REST,RFX3,RORA,RREB1,RXRA,SALL1,SFPQ,SOX1,SOX11,SP3,SRF,TBX2,HNF1B,TCF3,TCF7L2,ZEB1,TCF12,TEAD1,TFAP2A,TFAP2B,TFAP2C,TGIF1,KLF10,TOP1,TP73,NR2C2,TWIST1,KDM6A,VDR,XBP1,YY1,ZIC3,ZNF143,ZNF148,ZNF217,MAFK,NR4A3,NRIP1,KLF7,EED,FUBP3,ZBED1,KLF4,ONECUT2,TBPL1,CLOCK,ZNF516,ST18,ZNF536,USP3,NCOA2,NFAT5,SATB2,SUZ12,PATZ1,SSBP2,SSBP3,AGO1,TOX3,KLF15,GRHL1,SIX4,SOX6,CHD7,FOXJ2,ARNTL2,ZBTB2,NEUROG2,NEUROD6,EBF2,CREB3L2,RFX7,BCL11B,E2F8,ZFHX4,NKX6-2,KDM2B,TOX2,FOXP2,AEBP2,EBF3,FEZF1 |
| 3_Member | GO Molecular Functions | GO:0000978 | RNA polymerase II proximal promoter sequence-specific DNA binding | -28.11374503 | -25.255 | 149/544 | 60,196,367,406,463,639,688,860,1106,1316,1385,1386,1482,1602,1874,2004,2005,2033,2078,2113,2115,2120,2297,2313,2353,2494,2625,2626,2649,2736,2737,2908,3015,3170,3190,3192,3200,3202,3206,3212,3217,3725,4005,4086,4091,4094,4097,4205,4208,4212,4223,4286,4520,4603,4613,4760,4762,4773,4774,4775,4781,4783,4851,4929,5079,5241,5308,5451,5469,5629,5978,5991,6095,6239,6256,6299,6421,6656,6664,6670,6722,6909,6929,6934,6935,6938,7020,7021,7022,7050,7071,7150,7161,7182,7291,7403,7421,7494,7528,7547,7702,7707,7764,7975,8013,8204,8609,8726,8939,9189,9314,9480,9519,9575,9705,9745,9960,10499,10725,23314,23512,23598,23635,23648,26523,27324,28999,29841,51804,55553,55636,55810,56938,57621,63973,63974,64641,64764,64864,64919,79733,79776,84504,84678,84969,93986,121536,253738,389549 | ACTB,AHR,AR,ARNTL,ZFHX3,PRDM1,KLF5,RUNX2,CHD2,KLF6,CREB1,ATF2,NKX2-5,DACH1,E2F4,ELK3,ELK4,EP300,ERG,ETS1,ETV1,ETV6,FOXD1,FLI1,FOS,NR5A2,GATA3,GATA4,NR6A1,GLI2,GLI3,NR3C1,H2AZ1,FOXA2,HNRNPK,HNRNPU,HOXA3,HOXA5,HOXA10,HOXB2,HOXB7,JUN,LMO2,SMAD1,SMAD6,MAF,MAFG,MEF2A,MEF2C,MEIS2,MEOX2,MITF,MTF1,MYBL1,MYCN,NEUROD1,NEUROG1,NFATC2,NFIA,NFATC3,NFIB,NFIL3,NOTCH1,NR4A2,PAX5,PGR,PITX2,POU2F1,MED1,PROX1,REST,RFX3,RORA,RREB1,RXRA,SALL1,SFPQ,SOX1,SOX11,SP3,SRF,TBX2,TCF3,TCF7L2,ZEB1,TCF12,TFAP2A,TFAP2B,TFAP2C,TGIF1,KLF10,TOP1,TP73,NR2C2,TWIST1,KDM6A,VDR,XBP1,YY1,ZIC3,ZNF143,ZNF148,ZNF217,MAFK,NR4A3,NRIP1,KLF7,EED,FUBP3,ZBED1,KLF4,ONECUT2,TBPL1,CLOCK,ST18,ZNF536,USP3,NCOA2,NFAT5,SATB2,SUZ12,PATZ1,SSBP2,SSBP3,AGO1,TOX3,KLF15,GRHL1,SIX4,SOX6,CHD7,FOXJ2,ARNTL2,ZBTB2,NEUROG2,NEUROD6,EBF2,CREB3L2,RFX7,BCL11B,E2F8,ZFHX4,NKX6-2,KDM2B,TOX2,FOXP2,AEBP2,EBF3,FEZF1 |
| 3_Member | GO Molecular Functions | GO:0001228 | DNA-binding transcription activator activity, RNA polymerase II-specific | -25.85009893 | -23.143 | 126/439 | 367,406,571,688,860,988,1316,1385,1386,1482,1871,1874,1958,2004,2005,2033,2078,2113,2115,2119,2120,2294,2295,2297,2313,2353,2494,2625,2626,2649,2736,2737,2908,3131,3169,3190,3198,3202,3206,3212,3217,3234,3660,3725,4005,4086,4094,4097,4205,4208,4212,4223,4286,4520,4603,4613,4661,4760,4773,4774,4775,4779,4781,4851,4929,5079,5090,5241,5316,5326,5978,6095,6239,6656,6657,6664,6722,6909,6938,7020,7021,7022,7027,7071,7101,7161,7182,7547,7690,7702,7716,8013,8609,8939,9189,9314,9480,9496,9519,9575,9705,9839,10274,10725,23036,23040,23269,23314,23411,23435,23598,28999,29841,30812,51804,55810,57822,63974,64641,64764,64919,79977,84969,153222,253738,359948 | AR,ARNTL,BACH1,KLF5,RUNX2,CDC5L,KLF6,CREB1,ATF2,NKX2-5,E2F3,E2F4,EGR1,ELK3,ELK4,EP300,ERG,ETS1,ETV1,ETV5,ETV6,FOXF1,FOXF2,FOXD1,FLI1,FOS,NR5A2,GATA3,GATA4,NR6A1,GLI2,GLI3,NR3C1,HLF,FOXA1,HNRNPK,HOXA1,HOXA5,HOXA10,HOXB2,HOXB7,HOXD8,IRF2,JUN,LMO2,SMAD1,MAF,MAFG,MEF2A,MEF2C,MEIS2,MEOX2,MITF,MTF1,MYBL1,MYCN,MYT1,NEUROD1,NFATC2,NFIA,NFATC3,NFE2L1,NFIB,NOTCH1,NR4A2,PAX5,PBX3,PGR,PKNOX1,PLAGL2,REST,RORA,RREB1,SOX1,SOX2,SOX11,SRF,TBX2,TCF12,TFAP2A,TFAP2B,TFAP2C,TFDP1,KLF10,NR2E1,TP73,NR2C2,ZIC3,ZNF131,ZNF143,VEZF1,NR4A3,KLF7,FUBP3,ZBED1,KLF4,ONECUT2,TBX4,TBPL1,CLOCK,ST18,ZEB2,STAG1,NFAT5,ZNF292,MYT1L,MGA,SATB2,SIRT1,TARDBP,PATZ1,KLF15,GRHL1,SOX8,SIX4,FOXJ2,GRHL3,NEUROD6,EBF2,CREB3L2,BCL11B,GRHL2,TOX2,CREBRF,EBF3,IRF2BP2 |
| 3_Member | GO Molecular Functions | GO:0003682 | chromatin binding | -18.29907053 | -15.970 | 129/546 | 60,367,473,546,604,639,860,905,1164,1386,1387,1482,1499,1655,1874,1956,1958,2005,2033,2078,2308,2313,2332,2353,2494,2736,2737,2965,3015,3184,3192,3720,3725,4091,4205,4208,4286,4300,4335,4676,4760,4762,4773,4774,4775,4779,4851,5469,5563,5901,5927,5976,5978,6256,6304,6421,6602,6670,6722,6934,6935,7020,7021,7068,7150,7155,7403,7546,7552,8019,8028,8289,8467,8535,8725,8726,8841,9126,9314,9425,9555,9575,9611,9612,9960,10194,10274,10463,10499,10725,10735,10891,10951,11051,22823,23054,23081,23272,23314,23394,23512,23598,25842,25942,26053,26057,27324,29841,50809,51317,51322,51780,54454,54737,55636,55777,56916,57616,57822,64641,79977,80816,80854,83990,84289,84733,93166,128553,221037 | ACTB,AR,RERE,ATRX,BCL6,PRDM1,RUNX2,CCNT2,CKS2,ATF2,CREBBP,NKX2-5,CTNNB1,DDX5,E2F4,EGFR,EGR1,ELK4,EP300,ERG,FOXO1,FLI1,FMR1,FOS,NR5A2,GLI2,GLI3,GTF2H1,H2AZ1,HNRNPD,HNRNPU,JARID2,JUN,SMAD6,MEF2A,MEF2C,MITF,MLLT3,MNT,NAP1L4,NEUROD1,NEUROG1,NFATC2,NFIA,NFATC3,NFE2L1,NOTCH1,MED1,PRKAA2,RAN,KDM5A,UPF1,REST,RXRA,SATB1,SFPQ,SMARCD1,SP3,SRF,TCF7L2,ZEB1,TFAP2A,TFAP2B,THRB,TOP1,TOP2B,KDM6A,ZIC2,ZNF711,BRD3,MLLT10,ARID1A,SMARCA5,CBX4,URI1,EED,HDAC3,SMC3,KLF4,CDYL,MACROH2A1,CLOCK,NCOR1,NCOR2,USP3,TSHZ1,STAG1,SLC30A9,NCOA2,NFAT5,STAG2,PPARGC1A,CBX1,NUDT21,MTF2,NCOA6,KDM4C,TASOR,SATB2,ADNP,SUZ12,PATZ1,ASF1A,SIN3A,AUTS2,ANKRD17,TOX3,GRHL1,HP1BP3,PHF21A,WAC,KDM3B,ATAD2B,MPHOSPH8,CHD7,MBD5,SMARCAD1,TSHZ3,GRHL3,EBF2,GRHL2,ASXL3,SETD7,BRIP1,ING5,CBX2,PRDM6,TSHZ2,JMJD1C |
| 4_Summary | GO Biological Processes | GO:0007420 | brain development | -35.84217337 | -32.668 | 197/734 | 18,25,60,351,357,372,387,463,473,490,493,546,547,650,655,657,659,664,801,805,998,1021,1272,1281,1282,1385,1398,1399,1499,1600,1808,1812,1869,1901,1956,2009,2044,2045,2048,2066,2113,2186,2255,2263,2334,2475,2534,2558,2736,2737,2895,2903,2904,3084,3157,3176,3184,3212,3398,3688,3736,3746,3747,3799,3845,4040,4086,4142,4628,4724,4760,4763,4781,4851,4915,4929,5048,5049,5079,5270,5308,5354,5362,5455,5469,5530,5592,5604,5629,5649,5727,5728,5793,5999,6091,6092,6095,6299,6310,6453,6491,6497,6538,6546,6654,6656,6657,6722,6857,6860,6928,7048,7101,7155,7248,7337,7531,7533,7546,7707,7832,7852,7881,8013,8289,8321,8455,8633,8650,8660,8828,8829,8851,8867,8925,9037,9201,9231,9353,9693,9839,9919,10000,10092,10395,10427,10458,10579,10818,10891,10971,22903,22931,22941,23054,23236,23266,23314,23353,23543,23544,23648,25942,26038,26468,27023,28514,29072,54407,54806,55636,56934,57142,57282,57526,57688,58155,59345,63974,64919,79977,80031,80055,80853,81565,84002,84678,84879,93986,136319,139411,152330,167153,317762,342371,389549,431707,2033,5156,5594,23767,59338,83690,84159 | ABAT,ABL1,ACTB,APP,SHROOM2,ARCN1,RHOA,ZFHX3,RERE,ATP2B1,ATP2B4,ATRX,KIF1A,BMP2,BMP7,BMPR1A,BMPR2,BNIP3,CALM1,CALM2,CDC42,CDK6,CNTN1,COL3A1,COL4A1,CREB1,CRK,CRKL,CTNNB1,DAB1,DPYSL2,DRD1,E2F1,S1PR1,EGFR,EML1,EPHA5,EPHA7,EPHB2,ERBB4,ETS1,BPTF,FGF10,FGFR2,AFF2,MTOR,FYN,GABRA5,GLI2,GLI3,GRID2,GRIN2A,GRIN2B,NRG1,HMGCS1,HNMT,HNRNPD,HOXB2,ID2,ITGB1,KCNA1,KCNC1,KCNC2,KIF5B,KRAS,LRP6,SMAD1,MAS1,MYH10,NDUFS4,NEUROD1,NF1,NFIB,NOTCH1,NTRK2,NR4A2,PAFAH1B1,PAFAH1B2,PAX5,SERPINE2,PITX2,PLP1,PLXNA2,POU3F3,MED1,PPP3CA,PRKG1,MAP2K1,PROX1,RELN,PTCH1,PTEN,PTPRG,RGS4,ROBO1,ROBO2,RORA,SALL1,ATXN1,ITSN1,STIL,SKI,SLC6A11,SLC8A1,SOS1,SOX1,SOX2,SRF,SYT1,SYT4,HNF1B,TGFBR2,NR2E1,TOP2B,TSC1,UBE3A,YWHAE,YWHAH,ZIC2,ZNF148,BTG2,CXCR4,KCNAB1,NR4A3,ARID1A,FZD1,ATRN,UNC5C,NUMB,IRS2,NRP2,NRP1,CDK5R1,SYNJ1,HERC1,SEMA5A,DCLK1,DLG5,SLIT2,RAPGEF2,ZEB2,SEC16A,AKT3,ARPC5,DLC1,SEC24B,BAIAP2,TACC2,FRS2,PPARGC1A,YWHAQ,BTBD3,RAB18,SHANK2,NCOA6,PLCB1,ADGRL2,SATB2,SUN1,RBFOX2,SEZ6L,SSBP3,SIN3A,CHD5,LHX6,FOXB1,DLL1,SETD2,SLC38A2,AHI1,CHD7,CA10,RTN4,SLC4A10,PCDH19,ZSWIM6,PTBP2,GNB4,NEUROD6,BCL11B,GRHL2,SEMA6D,PGAP1,KDM7A,NDEL1,B3GNT5,KDM2B,MFSD2A,FOXP2,MTPN,PTCHD1,CNTN4,TENT2,CCDC85C,ATXN1L,FEZF1,LHX8,EP300,PDGFRA,MAPK1,FLRT3,PLEKHA1,CRISPLD1,ARID5B |
| 4_Member | GO Biological Processes | GO:0007420 | brain development | -35.84217337 | -32.668 | 197/734 | 18,25,60,351,357,372,387,463,473,490,493,546,547,650,655,657,659,664,801,805,998,1021,1272,1281,1282,1385,1398,1399,1499,1600,1808,1812,1869,1901,1956,2009,2044,2045,2048,2066,2113,2186,2255,2263,2334,2475,2534,2558,2736,2737,2895,2903,2904,3084,3157,3176,3184,3212,3398,3688,3736,3746,3747,3799,3845,4040,4086,4142,4628,4724,4760,4763,4781,4851,4915,4929,5048,5049,5079,5270,5308,5354,5362,5455,5469,5530,5592,5604,5629,5649,5727,5728,5793,5999,6091,6092,6095,6299,6310,6453,6491,6497,6538,6546,6654,6656,6657,6722,6857,6860,6928,7048,7101,7155,7248,7337,7531,7533,7546,7707,7832,7852,7881,8013,8289,8321,8455,8633,8650,8660,8828,8829,8851,8867,8925,9037,9201,9231,9353,9693,9839,9919,10000,10092,10395,10427,10458,10579,10818,10891,10971,22903,22931,22941,23054,23236,23266,23314,23353,23543,23544,23648,25942,26038,26468,27023,28514,29072,54407,54806,55636,56934,57142,57282,57526,57688,58155,59345,63974,64919,79977,80031,80055,80853,81565,84002,84678,84879,93986,136319,139411,152330,167153,317762,342371,389549,431707 | ABAT,ABL1,ACTB,APP,SHROOM2,ARCN1,RHOA,ZFHX3,RERE,ATP2B1,ATP2B4,ATRX,KIF1A,BMP2,BMP7,BMPR1A,BMPR2,BNIP3,CALM1,CALM2,CDC42,CDK6,CNTN1,COL3A1,COL4A1,CREB1,CRK,CRKL,CTNNB1,DAB1,DPYSL2,DRD1,E2F1,S1PR1,EGFR,EML1,EPHA5,EPHA7,EPHB2,ERBB4,ETS1,BPTF,FGF10,FGFR2,AFF2,MTOR,FYN,GABRA5,GLI2,GLI3,GRID2,GRIN2A,GRIN2B,NRG1,HMGCS1,HNMT,HNRNPD,HOXB2,ID2,ITGB1,KCNA1,KCNC1,KCNC2,KIF5B,KRAS,LRP6,SMAD1,MAS1,MYH10,NDUFS4,NEUROD1,NF1,NFIB,NOTCH1,NTRK2,NR4A2,PAFAH1B1,PAFAH1B2,PAX5,SERPINE2,PITX2,PLP1,PLXNA2,POU3F3,MED1,PPP3CA,PRKG1,MAP2K1,PROX1,RELN,PTCH1,PTEN,PTPRG,RGS4,ROBO1,ROBO2,RORA,SALL1,ATXN1,ITSN1,STIL,SKI,SLC6A11,SLC8A1,SOS1,SOX1,SOX2,SRF,SYT1,SYT4,HNF1B,TGFBR2,NR2E1,TOP2B,TSC1,UBE3A,YWHAE,YWHAH,ZIC2,ZNF148,BTG2,CXCR4,KCNAB1,NR4A3,ARID1A,FZD1,ATRN,UNC5C,NUMB,IRS2,NRP2,NRP1,CDK5R1,SYNJ1,HERC1,SEMA5A,DCLK1,DLG5,SLIT2,RAPGEF2,ZEB2,SEC16A,AKT3,ARPC5,DLC1,SEC24B,BAIAP2,TACC2,FRS2,PPARGC1A,YWHAQ,BTBD3,RAB18,SHANK2,NCOA6,PLCB1,ADGRL2,SATB2,SUN1,RBFOX2,SEZ6L,SSBP3,SIN3A,CHD5,LHX6,FOXB1,DLL1,SETD2,SLC38A2,AHI1,CHD7,CA10,RTN4,SLC4A10,PCDH19,ZSWIM6,PTBP2,GNB4,NEUROD6,BCL11B,GRHL2,SEMA6D,PGAP1,KDM7A,NDEL1,B3GNT5,KDM2B,MFSD2A,FOXP2,MTPN,PTCHD1,CNTN4,TENT2,CCDC85C,ATXN1L,FEZF1,LHX8 |
| 4_Member | GO Biological Processes | GO:0060322 | head development | -35.63552929 | -32.489 | 204/777 | 18,25,60,351,357,372,387,463,473,490,493,546,547,650,655,657,659,664,801,805,998,1021,1272,1281,1282,1385,1398,1399,1499,1600,1808,1812,1869,1901,1956,2009,2033,2044,2045,2048,2066,2113,2186,2255,2263,2334,2475,2534,2558,2736,2737,2895,2903,2904,3084,3157,3176,3184,3212,3398,3688,3736,3746,3747,3799,3845,4040,4086,4142,4628,4724,4760,4763,4781,4851,4915,4929,5048,5049,5079,5156,5270,5308,5354,5362,5455,5469,5530,5592,5594,5604,5629,5649,5727,5728,5793,5999,6091,6092,6095,6299,6310,6453,6491,6497,6538,6546,6654,6656,6657,6722,6857,6860,6928,7048,7101,7155,7248,7337,7531,7533,7546,7707,7832,7852,7881,8013,8289,8321,8455,8633,8650,8660,8828,8829,8851,8867,8925,9037,9201,9231,9353,9693,9839,9919,10000,10092,10395,10427,10458,10579,10818,10891,10971,22903,22931,22941,23054,23236,23266,23314,23353,23543,23544,23648,23767,25942,26038,26468,27023,28514,29072,54407,54806,55636,56934,57142,57282,57526,57688,58155,59338,59345,63974,64919,79977,80031,80055,80853,81565,83690,84002,84159,84678,84879,93986,136319,139411,152330,167153,317762,342371,389549,431707 | ABAT,ABL1,ACTB,APP,SHROOM2,ARCN1,RHOA,ZFHX3,RERE,ATP2B1,ATP2B4,ATRX,KIF1A,BMP2,BMP7,BMPR1A,BMPR2,BNIP3,CALM1,CALM2,CDC42,CDK6,CNTN1,COL3A1,COL4A1,CREB1,CRK,CRKL,CTNNB1,DAB1,DPYSL2,DRD1,E2F1,S1PR1,EGFR,EML1,EP300,EPHA5,EPHA7,EPHB2,ERBB4,ETS1,BPTF,FGF10,FGFR2,AFF2,MTOR,FYN,GABRA5,GLI2,GLI3,GRID2,GRIN2A,GRIN2B,NRG1,HMGCS1,HNMT,HNRNPD,HOXB2,ID2,ITGB1,KCNA1,KCNC1,KCNC2,KIF5B,KRAS,LRP6,SMAD1,MAS1,MYH10,NDUFS4,NEUROD1,NF1,NFIB,NOTCH1,NTRK2,NR4A2,PAFAH1B1,PAFAH1B2,PAX5,PDGFRA,SERPINE2,PITX2,PLP1,PLXNA2,POU3F3,MED1,PPP3CA,PRKG1,MAPK1,MAP2K1,PROX1,RELN,PTCH1,PTEN,PTPRG,RGS4,ROBO1,ROBO2,RORA,SALL1,ATXN1,ITSN1,STIL,SKI,SLC6A11,SLC8A1,SOS1,SOX1,SOX2,SRF,SYT1,SYT4,HNF1B,TGFBR2,NR2E1,TOP2B,TSC1,UBE3A,YWHAE,YWHAH,ZIC2,ZNF148,BTG2,CXCR4,KCNAB1,NR4A3,ARID1A,FZD1,ATRN,UNC5C,NUMB,IRS2,NRP2,NRP1,CDK5R1,SYNJ1,HERC1,SEMA5A,DCLK1,DLG5,SLIT2,RAPGEF2,ZEB2,SEC16A,AKT3,ARPC5,DLC1,SEC24B,BAIAP2,TACC2,FRS2,PPARGC1A,YWHAQ,BTBD3,RAB18,SHANK2,NCOA6,PLCB1,ADGRL2,SATB2,SUN1,RBFOX2,SEZ6L,SSBP3,FLRT3,SIN3A,CHD5,LHX6,FOXB1,DLL1,SETD2,SLC38A2,AHI1,CHD7,CA10,RTN4,SLC4A10,PCDH19,ZSWIM6,PTBP2,PLEKHA1,GNB4,NEUROD6,BCL11B,GRHL2,SEMA6D,PGAP1,KDM7A,NDEL1,CRISPLD1,B3GNT5,ARID5B,KDM2B,MFSD2A,FOXP2,MTPN,PTCHD1,CNTN4,TENT2,CCDC85C,ATXN1L,FEZF1,LHX8 |
| 4_Member | GO Biological Processes | GO:0030900 | forebrain development | -21.65522015 | -19.202 | 108/382 | 351,387,493,546,547,650,657,664,1021,1281,1385,1398,1399,1499,1600,1812,1869,1956,2044,2048,2066,2113,2255,2263,2534,2736,2737,3084,3398,3736,3746,3747,3799,3845,4040,4142,4628,4760,4763,4781,4851,4915,4929,5048,5079,5308,5455,5592,5629,5649,5728,6091,6092,6299,6491,6497,6546,6656,6657,6722,7101,7155,7248,7531,7832,7852,8013,8289,8650,8828,8829,8851,9037,9201,9353,9693,9839,10092,10395,10579,10818,10891,22903,23236,23314,23353,25942,26038,26468,27023,29072,54407,55636,57142,57282,57688,63974,64919,80055,81565,84678,84879,93986,139411,167153,317762,389549,431707 | APP,RHOA,ATP2B4,ATRX,KIF1A,BMP2,BMPR1A,BNIP3,CDK6,COL3A1,CREB1,CRK,CRKL,CTNNB1,DAB1,DRD1,E2F1,EGFR,EPHA5,EPHB2,ERBB4,ETS1,FGF10,FGFR2,FYN,GLI2,GLI3,NRG1,ID2,KCNA1,KCNC1,KCNC2,KIF5B,KRAS,LRP6,MAS1,MYH10,NEUROD1,NF1,NFIB,NOTCH1,NTRK2,NR4A2,PAFAH1B1,PAX5,PITX2,POU3F3,PRKG1,PROX1,RELN,PTEN,ROBO1,ROBO2,SALL1,STIL,SKI,SLC8A1,SOX1,SOX2,SRF,NR2E1,TOP2B,TSC1,YWHAE,BTG2,CXCR4,NR4A3,ARID1A,NUMB,NRP2,NRP1,CDK5R1,SEMA5A,DCLK1,SLIT2,RAPGEF2,ZEB2,ARPC5,DLC1,TACC2,FRS2,PPARGC1A,BTBD3,PLCB1,SATB2,SUN1,SIN3A,CHD5,LHX6,FOXB1,SETD2,SLC38A2,CHD7,RTN4,SLC4A10,ZSWIM6,NEUROD6,BCL11B,PGAP1,NDEL1,KDM2B,MFSD2A,FOXP2,PTCHD1,TENT2,CCDC85C,FEZF1,LHX8 |
| 4_Member | GO Biological Processes | GO:0021537 | telencephalon development | -13.74149231 | -11.703 | 70/253 | 387,493,650,664,1021,1281,1398,1399,1499,1600,1812,1956,2044,2048,2066,2737,3084,3398,3736,3799,4040,4142,4628,4760,4763,4781,4915,5048,5079,5455,5629,5649,5728,6091,6092,6299,6497,6546,6722,7101,7248,7531,7832,7852,8013,8650,8828,8829,8851,9353,9839,10579,22903,23236,23353,26468,27023,54407,55636,57142,57688,63974,64919,81565,84678,84879,93986,167153,317762,389549 | RHOA,ATP2B4,BMP2,BNIP3,CDK6,COL3A1,CRK,CRKL,CTNNB1,DAB1,DRD1,EGFR,EPHA5,EPHB2,ERBB4,GLI3,NRG1,ID2,KCNA1,KIF5B,LRP6,MAS1,MYH10,NEUROD1,NF1,NFIB,NTRK2,PAFAH1B1,PAX5,POU3F3,PROX1,RELN,PTEN,ROBO1,ROBO2,SALL1,SKI,SLC8A1,SRF,NR2E1,TSC1,YWHAE,BTG2,CXCR4,NR4A3,NUMB,NRP2,NRP1,CDK5R1,SLIT2,ZEB2,TACC2,BTBD3,PLCB1,SUN1,LHX6,FOXB1,SLC38A2,CHD7,RTN4,ZSWIM6,NEUROD6,BCL11B,NDEL1,KDM2B,MFSD2A,FOXP2,TENT2,CCDC85C,FEZF1 |
| 4_Member | GO Biological Processes | GO:0021543 | pallium development | -11.43487465 | -9.545 | 51/172 | 387,493,664,1021,1281,1398,1399,1499,1600,1812,1956,2044,2737,3736,3799,4040,4142,4760,4763,4915,5048,5079,5455,5629,5649,5728,6091,6722,7101,7248,7531,7832,8013,8828,8829,8851,9353,9839,10579,22903,23236,23353,26468,54407,57142,63974,81565,84879,93986,167153,317762 | RHOA,ATP2B4,BNIP3,CDK6,COL3A1,CRK,CRKL,CTNNB1,DAB1,DRD1,EGFR,EPHA5,GLI3,KCNA1,KIF5B,LRP6,MAS1,NEUROD1,NF1,NTRK2,PAFAH1B1,PAX5,POU3F3,PROX1,RELN,PTEN,ROBO1,SRF,NR2E1,TSC1,YWHAE,BTG2,NR4A3,NRP2,NRP1,CDK5R1,SLIT2,ZEB2,TACC2,BTBD3,PLCB1,SUN1,LHX6,SLC38A2,RTN4,NEUROD6,NDEL1,MFSD2A,FOXP2,TENT2,CCDC85C |
| 4_Member | GO Biological Processes | GO:0021761 | limbic system development | -8.487953925 | -6.817 | 34/109 | 493,1021,1398,1399,1600,1812,2044,2113,2263,2737,3736,3799,4142,4760,4763,5048,5629,5649,5728,6722,7101,7248,7531,7832,8013,8828,8829,8851,9839,10092,27023,63974,84879,167153 | ATP2B4,CDK6,CRK,CRKL,DAB1,DRD1,EPHA5,ETS1,FGFR2,GLI3,KCNA1,KIF5B,MAS1,NEUROD1,NF1,PAFAH1B1,PROX1,RELN,PTEN,SRF,NR2E1,TSC1,YWHAE,BTG2,NR4A3,NRP2,NRP1,CDK5R1,ZEB2,ARPC5,FOXB1,NEUROD6,MFSD2A,TENT2 |
| 4_Member | GO Biological Processes | GO:0021987 | cerebral cortex development | -7.721161235 | -6.121 | 34/116 | 387,664,1281,1398,1399,1499,1600,1956,2737,4040,4763,4915,5048,5079,5455,5649,6091,7101,7248,7531,8828,8829,8851,9353,10579,22903,23236,23353,26468,54407,57142,81565,93986,317762 | RHOA,BNIP3,COL3A1,CRK,CRKL,CTNNB1,DAB1,EGFR,GLI3,LRP6,NF1,NTRK2,PAFAH1B1,PAX5,POU3F3,RELN,ROBO1,NR2E1,TSC1,YWHAE,NRP2,NRP1,CDK5R1,SLIT2,TACC2,BTBD3,PLCB1,SUN1,LHX6,SLC38A2,RTN4,NDEL1,FOXP2,CCDC85C |
| 4_Member | GO Biological Processes | GO:0021766 | hippocampus development | -7.56088299 | -5.977 | 27/81 | 493,1021,1398,1399,1600,1812,2044,2737,3736,3799,4142,4760,5048,5629,5649,5728,6722,7101,7248,7531,7832,8013,8851,9839,63974,84879,167153 | ATP2B4,CDK6,CRK,CRKL,DAB1,DRD1,EPHA5,GLI3,KCNA1,KIF5B,MAS1,NEUROD1,PAFAH1B1,PROX1,RELN,PTEN,SRF,NR2E1,TSC1,YWHAE,BTG2,NR4A3,CDK5R1,ZEB2,NEUROD6,MFSD2A,TENT2 |
| 5_Summary | GO Biological Processes | GO:0070848 | response to growth factor | -34.88031935 | -31.760 | 196/739 | 25,90,92,351,387,463,607,650,654,655,657,659,677,753,858,860,890,960,988,1028,1278,1281,1385,1387,1398,1399,1482,1490,1499,1634,1655,1783,1848,1869,1915,1956,1958,2033,2066,2081,2200,2201,2252,2255,2261,2263,2277,2297,2353,2534,2549,2615,2619,2625,2626,2885,2887,2908,3037,3082,3096,3491,3643,3688,3725,3746,3747,4052,4086,4091,4131,4208,4212,4734,4756,4803,4851,4915,5139,5151,5155,5156,5170,5469,5494,5594,5725,5728,5782,5796,5829,5962,6091,6093,6198,6497,6653,6654,6657,6660,6664,6885,6935,7021,7042,7046,7048,7050,7057,7073,7291,7321,7323,7337,7412,7424,8036,8239,8321,8573,8678,8725,8817,8828,8829,8936,9110,9197,9314,9353,9475,9480,9508,9693,9863,10140,10252,10253,10458,10479,10631,10818,10891,11021,11171,23064,23090,23213,23411,23543,23592,23767,23768,25928,26060,26281,26999,27020,27063,27250,27302,28514,28996,51496,51552,51592,51701,54206,54567,54778,55512,55553,55614,55959,56975,57154,57556,57608,63976,64094,64388,64399,64750,79801,80004,83891,91851,94031,94056,130399,161742,168667,200734,222008,285704,348093,60,163,284,324,367,526,528,868,998,1123,1173,1176,1282,1287,1946,2043,2044,2045,2048,2185,2308,2904,3084,3482,3717,3815,4643,4919,5062,5063,5291,5295,5578,5588,5618,5664,5771,5774,5791,5793,5801,5921,6383,6453,6760,7074,7248,7879,8013,8027,8065,8440,8660,8851,8976,9021,9252,9451,9655,9770,9788,9860,10006,10092,10109,10135,10580,10636,10787,11122,23189,23236,23294,26058,27131,30011,54806,55023,55294,55777,55914,56034,57142,59338,81565,84159,84959,89853,143686,222584 | ABL1,ACVR1,ACVR2A,APP,RHOA,ZFHX3,BCL9,BMP2,BMP6,BMP7,BMPR1A,BMPR2,ZFP36L1,LDLRAD4,CAV2,RUNX2,CCNA2,CD44,CDC5L,CDKN1C,COL1A2,COL3A1,CREB1,CREBBP,CRK,CRKL,NKX2-5,CCN2,CTNNB1,DCN,DDX5,DYNC1LI2,DUSP6,E2F1,EEF1A1,EGFR,EGR1,EP300,ERBB4,ERN1,FBN1,FBN2,FGF7,FGF10,FGFR3,FGFR2,VEGFD,FOXD1,FOS,FYN,GAB1,LRRC32,GAS1,GATA3,GATA4,GRB2,GRB10,NR3C1,HAS2,HGF,HIVEP1,CCN1,INSR,ITGB1,JUN,KCNC1,KCNC2,LTBP1,SMAD1,SMAD6,MAP1B,MEF2C,MEIS2,NEDD4,NEO1,NGF,NOTCH1,NTRK2,PDE3A,PDE8A,PDGFB,PDGFRA,PDPK1,MED1,PPM1A,MAPK1,PTBP1,PTEN,PTPN12,PTPRK,PXN,RDX,ROBO1,ROCK1,RPS6KB1,SKI,SORL1,SOS1,SOX2,SOX5,SOX11,MAP3K7,ZEB1,TFAP2B,TGFB2,TGFBR1,TGFBR2,TGIF1,THBS1,TIAL1,TWIST1,UBE2D1,UBE2D3,UBE3A,VCAM1,VEGFC,SHOC2,USP9X,FZD1,CASK,BECN1,URI1,FGF18,NRP2,NRP1,WASF1,MTMR4,SLC33A1,KLF4,SLIT2,ROCK2,ONECUT2,ADAMTS3,RAPGEF2,MAGI2,TOB1,SPRY1,SPRY2,BAIAP2,SLC9A6,POSTN,FRS2,PPARGC1A,RAB35,STRAP,SETX,ZNF423,SULF1,SIRT1,RBFOX2,LEMD3,FLRT3,FLRT2,SOSTDC1,APPL1,FGF20,CYFIP2,NPTN,ANKRD1,PDCD4,BMP10,DLL1,HIPK2,CTDSPL2,RAB14,TRIM33,NLK,ERRFI1,DLL4,RNF111,SMPD3,SOX6,KIF16B,SULF2,FAM20C,SMURF1,SEMA6A,JCAD,PRDM16,SMOC2,GREM2,HHIP,SMURF2,SHCBP1,ESRP2,SNX25,CHRDL1,HTRA3,SYAP1,ACVR1C,SPRED1,BMPER,SPRED2,VSTM2A,RGMB,RBPMS2,ACTB,AP2B1,ANGPT1,APC,AR,ATP6V1B2,ATP6V1C1,CBLB,CDC42,CHN1,AP2M1,AP3S1,COL4A1,COL4A5,EFNA5,EPHA4,EPHA5,EPHA7,EPHB2,PTK2B,FOXO1,GRIN2B,NRG1,IGF2R,JAK2,KIT,MYO1E,ROR1,PAK2,PAK3,PIK3CB,PIK3R1,PRKCA,PRKCQ,PRLR,PSEN2,PTPN2,PTPN3,PTPRE,PTPRG,PTPRR,RASA1,SDC2,ITSN1,SS18,TIAM1,TSC1,RAB7A,NR4A3,STAM,CUL5,NCK2,IRS2,CDK5R1,WASL,SOCS3,RPS6KA5,EIF2AK3,SOCS5,RASSF2,MTSS1,LRIG2,ABI1,ARPC5,ARPC2,NAMPT,SORBS1,RGS14,NCKAP1,PTPRT,KANK1,PLCB1,ANKS1A,GIGYF2,SNX5,SH3KBP1,AHI1,PHIP,FBXW7,MBD5,ERBIN,PDGFC,RTN4,PLEKHA1,NDEL1,ARID5B,UBASH3B,MVB12B,SESN3,FAM83B |
| 5_Member | GO Biological Processes | GO:0070848 | response to growth factor | -34.88031935 | -31.760 | 196/739 | 25,90,92,351,387,463,607,650,654,655,657,659,677,753,858,860,890,960,988,1028,1278,1281,1385,1387,1398,1399,1482,1490,1499,1634,1655,1783,1848,1869,1915,1956,1958,2033,2066,2081,2200,2201,2252,2255,2261,2263,2277,2297,2353,2534,2549,2615,2619,2625,2626,2885,2887,2908,3037,3082,3096,3491,3643,3688,3725,3746,3747,4052,4086,4091,4131,4208,4212,4734,4756,4803,4851,4915,5139,5151,5155,5156,5170,5469,5494,5594,5725,5728,5782,5796,5829,5962,6091,6093,6198,6497,6653,6654,6657,6660,6664,6885,6935,7021,7042,7046,7048,7050,7057,7073,7291,7321,7323,7337,7412,7424,8036,8239,8321,8573,8678,8725,8817,8828,8829,8936,9110,9197,9314,9353,9475,9480,9508,9693,9863,10140,10252,10253,10458,10479,10631,10818,10891,11021,11171,23064,23090,23213,23411,23543,23592,23767,23768,25928,26060,26281,26999,27020,27063,27250,27302,28514,28996,51496,51552,51592,51701,54206,54567,54778,55512,55553,55614,55959,56975,57154,57556,57608,63976,64094,64388,64399,64750,79801,80004,83891,91851,94031,94056,130399,161742,168667,200734,222008,285704,348093 | ABL1,ACVR1,ACVR2A,APP,RHOA,ZFHX3,BCL9,BMP2,BMP6,BMP7,BMPR1A,BMPR2,ZFP36L1,LDLRAD4,CAV2,RUNX2,CCNA2,CD44,CDC5L,CDKN1C,COL1A2,COL3A1,CREB1,CREBBP,CRK,CRKL,NKX2-5,CCN2,CTNNB1,DCN,DDX5,DYNC1LI2,DUSP6,E2F1,EEF1A1,EGFR,EGR1,EP300,ERBB4,ERN1,FBN1,FBN2,FGF7,FGF10,FGFR3,FGFR2,VEGFD,FOXD1,FOS,FYN,GAB1,LRRC32,GAS1,GATA3,GATA4,GRB2,GRB10,NR3C1,HAS2,HGF,HIVEP1,CCN1,INSR,ITGB1,JUN,KCNC1,KCNC2,LTBP1,SMAD1,SMAD6,MAP1B,MEF2C,MEIS2,NEDD4,NEO1,NGF,NOTCH1,NTRK2,PDE3A,PDE8A,PDGFB,PDGFRA,PDPK1,MED1,PPM1A,MAPK1,PTBP1,PTEN,PTPN12,PTPRK,PXN,RDX,ROBO1,ROCK1,RPS6KB1,SKI,SORL1,SOS1,SOX2,SOX5,SOX11,MAP3K7,ZEB1,TFAP2B,TGFB2,TGFBR1,TGFBR2,TGIF1,THBS1,TIAL1,TWIST1,UBE2D1,UBE2D3,UBE3A,VCAM1,VEGFC,SHOC2,USP9X,FZD1,CASK,BECN1,URI1,FGF18,NRP2,NRP1,WASF1,MTMR4,SLC33A1,KLF4,SLIT2,ROCK2,ONECUT2,ADAMTS3,RAPGEF2,MAGI2,TOB1,SPRY1,SPRY2,BAIAP2,SLC9A6,POSTN,FRS2,PPARGC1A,RAB35,STRAP,SETX,ZNF423,SULF1,SIRT1,RBFOX2,LEMD3,FLRT3,FLRT2,SOSTDC1,APPL1,FGF20,CYFIP2,NPTN,ANKRD1,PDCD4,BMP10,DLL1,HIPK2,CTDSPL2,RAB14,TRIM33,NLK,ERRFI1,DLL4,RNF111,SMPD3,SOX6,KIF16B,SULF2,FAM20C,SMURF1,SEMA6A,JCAD,PRDM16,SMOC2,GREM2,HHIP,SMURF2,SHCBP1,ESRP2,SNX25,CHRDL1,HTRA3,SYAP1,ACVR1C,SPRED1,BMPER,SPRED2,VSTM2A,RGMB,RBPMS2 |
| 5_Member | GO Biological Processes | GO:0071363 | cellular response to growth factor stimulus | -33.42970282 | -30.358 | 188/709 | 25,90,92,351,387,607,650,654,655,657,659,677,753,858,860,890,960,988,1028,1278,1281,1385,1387,1398,1399,1482,1490,1499,1634,1655,1783,1869,1915,1956,1958,2033,2066,2081,2200,2201,2252,2255,2261,2263,2277,2297,2353,2534,2549,2615,2619,2625,2626,2885,2887,2908,3037,3082,3096,3491,3643,3688,3725,4052,4086,4091,4131,4208,4734,4756,4803,4851,4915,5139,5151,5155,5156,5170,5469,5494,5594,5725,5728,5782,5796,5829,5962,6091,6198,6497,6653,6654,6660,6664,6885,6935,7021,7042,7046,7048,7050,7057,7073,7291,7321,7323,7337,7412,7424,8036,8239,8321,8573,8678,8725,8817,8828,8829,8936,9110,9197,9314,9353,9480,9508,9693,9863,10140,10252,10253,10458,10479,10631,10818,10891,11021,11171,23064,23090,23213,23411,23543,23592,23767,23768,25928,26060,26281,26999,27020,27063,27250,27302,28514,28996,51496,51552,51592,51701,54206,54567,54778,55512,55553,55614,55959,56975,57154,57556,57608,63976,64094,64388,64399,64750,79801,80004,83891,91851,94031,94056,130399,161742,168667,200734,222008,285704,348093 | ABL1,ACVR1,ACVR2A,APP,RHOA,BCL9,BMP2,BMP6,BMP7,BMPR1A,BMPR2,ZFP36L1,LDLRAD4,CAV2,RUNX2,CCNA2,CD44,CDC5L,CDKN1C,COL1A2,COL3A1,CREB1,CREBBP,CRK,CRKL,NKX2-5,CCN2,CTNNB1,DCN,DDX5,DYNC1LI2,E2F1,EEF1A1,EGFR,EGR1,EP300,ERBB4,ERN1,FBN1,FBN2,FGF7,FGF10,FGFR3,FGFR2,VEGFD,FOXD1,FOS,FYN,GAB1,LRRC32,GAS1,GATA3,GATA4,GRB2,GRB10,NR3C1,HAS2,HGF,HIVEP1,CCN1,INSR,ITGB1,JUN,LTBP1,SMAD1,SMAD6,MAP1B,MEF2C,NEDD4,NEO1,NGF,NOTCH1,NTRK2,PDE3A,PDE8A,PDGFB,PDGFRA,PDPK1,MED1,PPM1A,MAPK1,PTBP1,PTEN,PTPN12,PTPRK,PXN,RDX,ROBO1,RPS6KB1,SKI,SORL1,SOS1,SOX5,SOX11,MAP3K7,ZEB1,TFAP2B,TGFB2,TGFBR1,TGFBR2,TGIF1,THBS1,TIAL1,TWIST1,UBE2D1,UBE2D3,UBE3A,VCAM1,VEGFC,SHOC2,USP9X,FZD1,CASK,BECN1,URI1,FGF18,NRP2,NRP1,WASF1,MTMR4,SLC33A1,KLF4,SLIT2,ONECUT2,ADAMTS3,RAPGEF2,MAGI2,TOB1,SPRY1,SPRY2,BAIAP2,SLC9A6,POSTN,FRS2,PPARGC1A,RAB35,STRAP,SETX,ZNF423,SULF1,SIRT1,RBFOX2,LEMD3,FLRT3,FLRT2,SOSTDC1,APPL1,FGF20,CYFIP2,NPTN,ANKRD1,PDCD4,BMP10,DLL1,HIPK2,CTDSPL2,RAB14,TRIM33,NLK,ERRFI1,DLL4,RNF111,SMPD3,SOX6,KIF16B,SULF2,FAM20C,SMURF1,SEMA6A,JCAD,PRDM16,SMOC2,GREM2,HHIP,SMURF2,SHCBP1,ESRP2,SNX25,CHRDL1,HTRA3,SYAP1,ACVR1C,SPRED1,BMPER,SPRED2,VSTM2A,RGMB,RBPMS2 |
| 5_Member | GO Biological Processes | GO:0007169 | transmembrane receptor protein tyrosine kinase signaling pathway | -24.72596773 | -22.083 | 172/722 | 25,60,163,284,324,351,367,387,526,528,650,858,860,868,998,1123,1173,1176,1282,1287,1398,1399,1490,1499,1634,1946,1956,2043,2044,2045,2048,2066,2185,2252,2255,2261,2263,2277,2308,2534,2549,2625,2885,2887,2904,3082,3084,3482,3643,3717,3815,4643,4734,4803,4915,4919,5062,5063,5155,5156,5170,5291,5295,5578,5588,5594,5618,5664,5725,5728,5771,5774,5782,5791,5793,5801,5829,5921,6091,6093,6198,6383,6453,6653,6654,6760,7057,7073,7074,7248,7424,7879,8013,8027,8036,8065,8440,8660,8817,8828,8829,8851,8936,8976,9021,9252,9451,9475,9508,9655,9693,9770,9788,9860,10006,10092,10109,10135,10252,10253,10458,10479,10580,10636,10787,10818,11122,23064,23189,23213,23236,23294,23411,23543,23767,23768,26058,26060,26281,26999,27020,27131,28514,30011,51552,54206,54806,55023,55294,55512,55614,55777,55914,55959,56034,56975,57142,57556,57608,59338,64094,64399,79801,80004,81565,84159,84959,89853,143686,161742,200734,222584 | ABL1,ACTB,AP2B1,ANGPT1,APC,APP,AR,RHOA,ATP6V1B2,ATP6V1C1,BMP2,CAV2,RUNX2,CBLB,CDC42,CHN1,AP2M1,AP3S1,COL4A1,COL4A5,CRK,CRKL,CCN2,CTNNB1,DCN,EFNA5,EGFR,EPHA4,EPHA5,EPHA7,EPHB2,ERBB4,PTK2B,FGF7,FGF10,FGFR3,FGFR2,VEGFD,FOXO1,FYN,GAB1,GATA3,GRB2,GRB10,GRIN2B,HGF,NRG1,IGF2R,INSR,JAK2,KIT,MYO1E,NEDD4,NGF,NTRK2,ROR1,PAK2,PAK3,PDGFB,PDGFRA,PDPK1,PIK3CB,PIK3R1,PRKCA,PRKCQ,MAPK1,PRLR,PSEN2,PTBP1,PTEN,PTPN2,PTPN3,PTPN12,PTPRE,PTPRG,PTPRR,PXN,RASA1,ROBO1,ROCK1,RPS6KB1,SDC2,ITSN1,SORL1,SOS1,SS18,THBS1,TIAL1,TIAM1,TSC1,VEGFC,RAB7A,NR4A3,STAM,SHOC2,CUL5,NCK2,IRS2,FGF18,NRP2,NRP1,CDK5R1,WASF1,WASL,SOCS3,RPS6KA5,EIF2AK3,ROCK2,ADAMTS3,SOCS5,RAPGEF2,RASSF2,MTSS1,LRIG2,ABI1,ARPC5,ARPC2,NAMPT,SPRY1,SPRY2,BAIAP2,SLC9A6,SORBS1,RGS14,NCKAP1,FRS2,PTPRT,SETX,KANK1,SULF1,PLCB1,ANKS1A,SIRT1,RBFOX2,FLRT3,FLRT2,GIGYF2,APPL1,FGF20,CYFIP2,NPTN,SNX5,DLL1,SH3KBP1,RAB14,ERRFI1,AHI1,PHIP,FBXW7,SMPD3,KIF16B,MBD5,ERBIN,SULF2,PDGFC,FAM20C,RTN4,SEMA6A,JCAD,PLEKHA1,SMOC2,HHIP,SHCBP1,ESRP2,NDEL1,ARID5B,UBASH3B,MVB12B,SESN3,SPRED1,SPRED2,FAM83B |
| 6_Summary | GO Biological Processes | GO:0048729 | tissue morphogenesis | -34.51301633 | -31.418 | 184/674 | 25,90,163,182,367,387,650,655,657,659,960,998,1173,1213,1281,1282,1289,1303,1482,1499,1739,1857,1901,1956,2043,2045,2119,2138,2252,2255,2263,2294,2295,2297,2475,2625,2626,2668,2736,2737,2885,3082,3084,3169,3202,3212,3217,3491,3688,3714,3845,4040,4041,4086,4208,4300,4478,4638,4851,4919,5048,5100,5241,5361,5420,5469,5567,5573,5613,5629,5727,5728,5898,5911,6091,6092,6239,6256,6299,6491,6497,6654,6664,6722,6781,6788,6909,6928,7020,7042,7046,7048,7074,7168,7248,7291,7403,7421,7424,7430,7547,7852,8013,8289,8321,8828,8829,9021,9231,9314,9353,9496,9620,9723,9788,9839,9863,10082,10116,10252,10253,10277,10395,10427,10672,10818,10890,10959,11146,23002,23122,23213,23242,23332,23414,23493,23767,25928,26249,27063,27302,28514,29072,30812,51804,54567,54806,55083,55327,55366,55636,55727,55966,56998,57154,57167,57493,57534,57619,57822,59277,64398,64399,64750,64856,65979,79977,80000,80004,81578,81839,84133,84678,85407,90627,94134,115908,129446,130497,144165,153572,157506,166336,284217,92,102,118,284,320,321,639,677,860,1028,1889,2033,2182,2186,2494,3198,3200,4010,4097,4124,4154,4223,4325,4627,4628,4643,4763,4848,4863,5079,5125,5155,5156,5362,5594,5604,5801,5930,6670,6935,8178,8313,8452,8533,8851,9318,9508,9541,9644,9871,9927,10006,10087,10265,10436,10658,10742,23081,23168,23250,23314,23648,25942,26115,27023,54799,55122,55553,55746,55959,56243,56853,56977,57142,57178,57448,60482,60626,64131,79718,79733,79805,80312,81565,84295,84541,130507,253260,775,1387,2048,2200,2201,2335,3170,3206,3398,3638,3899,4038,4760,4762,6093,6657,8434,9475,10194,10586,26018,28996,51761,54928,55084,55614,64388,65125,79577,84189,121227,123169,125488,204851,221833,348093,100131390,473,1305,3815,56034,57599,196528,4781 | ABL1,ACVR1,AP2B1,JAG1,AR,RHOA,BMP2,BMP7,BMPR1A,BMPR2,CD44,CDC42,AP2M1,CLTC,COL3A1,COL4A1,COL5A1,COL12A1,NKX2-5,CTNNB1,DLG1,DVL3,S1PR1,EGFR,EPHA4,EPHA7,ETV5,EYA1,FGF7,FGF10,FGFR2,FOXF1,FOXF2,FOXD1,MTOR,GATA3,GATA4,GDNF,GLI2,GLI3,GRB2,HGF,NRG1,FOXA1,HOXA5,HOXB2,HOXB7,CCN1,ITGB1,JAG2,KRAS,LRP6,LRP5,SMAD1,MEF2C,MLLT3,MSN,MYLK,NOTCH1,ROR1,PAFAH1B1,PCDH8,PGR,PLXNA1,PODXL,MED1,PRKACB,PRKAR1A,PRKX,PROX1,PTCH1,PTEN,RALA,RAP2A,ROBO1,ROBO2,RREB1,RXRA,SALL1,STIL,SKI,SOS1,SOX11,SRF,STC1,STK3,TBX2,HNF1B,TFAP2A,TGFB2,TGFBR1,TGFBR2,TIAM1,TPM1,TSC1,TWIST1,KDM6A,VDR,VEGFC,EZR,ZIC3,CXCR4,NR4A3,ARID1A,FZD1,NRP2,NRP1,SOCS3,DLG5,KLF4,SLIT2,TBX4,CELSR1,SEMA3E,MTSS1,ZEB2,MAGI2,GPC6,FEM1B,SPRY1,SPRY2,UBE4B,DLC1,SEC24B,GNA13,FRS2,RAB10,TMED2,GLMN,DAAM1,CLASP2,SULF1,COBL,CLASP1,ZFPM2,HEY2,FLRT3,SOSTDC1,KLHL3,ANKRD1,BMP10,DLL1,SETD2,SOX8,SIX4,DLL4,AHI1,KIF26B,LIN7C,LGR4,CHD7,BTBD7,AJAP1,CTNNBIP1,SMURF1,SALL4,HEG1,MIB1,SHROOM3,GRHL3,NTN4,MPP5,HHIP,SMURF2,VWA1,PHACTR4,GRHL2,GREB1L,ESRP2,COL21A1,VANGL1,ZNRF3,KDM2B,NKD1,STARD13,ARHGAP12,CTHRC1,XIRP2,OSR1,PRICKLE1,IRX2,RDH10,PRICKLE2,LAMA1,ACVR2A,ADAM10,ADD1,ANGPT1,APBA1,APBA2,PRDM1,ZFP36L1,RUNX2,CDKN1C,ECE1,EP300,ACSL4,BPTF,NR5A2,HOXA1,HOXA3,LMX1B,MAFG,MAN2A1,MBNL1,MEOX2,MMP16,MYH9,MYH10,MYO1E,NF1,CNOT2,NPAT,PAX5,PCSK5,PDGFB,PDGFRA,PLXNA2,MAPK1,MAP2K1,PTPRR,RBBP6,SP3,ZEB1,ELL,AXIN2,CUL3,COPS3,CDK5R1,COPS2,ADAMTS3,CIR1,SH3PXD2A,SEC24D,MFN2,ABI1,CERT1,IRX5,EMG1,CELF1,RAI2,KDM4C,RTF1,ATP11A,SATB2,SSBP3,SIN3A,TANC2,FOXB1,MBTD1,AKIRIN2,SOX6,NUP133,SULF2,KIAA1217,CELF4,STOX2,RTN4,ZMIZ1,BIRC6,SLC5A7,RIC8A,XYLT1,TBL1XR1,E2F8,VASH2,TET1,NDEL1,PHF6,KBTBD8,UBR3,RICTOR,CACNA1C,CREBBP,EPHB2,FBN1,FBN2,FN1,FOXA2,HOXA10,ID2,INSIG1,AFF3,LRP4,NEUROD1,NEUROG1,ROCK1,SOX2,RECK,ROCK2,TSHZ1,MAB21L2,LRIG1,HIPK2,ATP8A2,IMPAD1,SOBP,KIF16B,GREM2,WNK1,CDC73,SLITRK6,LRIG3,LEO1,TTC39C,HIPK1,SP8,RBPMS2,SP9,RERE,COL13A1,KIT,PDGFC,WDR48,ARID2,NFIB |
| 6_Member | GO Biological Processes | GO:0048729 | tissue morphogenesis | -34.51301633 | -31.418 | 184/674 | 25,90,163,182,367,387,650,655,657,659,960,998,1173,1213,1281,1282,1289,1303,1482,1499,1739,1857,1901,1956,2043,2045,2119,2138,2252,2255,2263,2294,2295,2297,2475,2625,2626,2668,2736,2737,2885,3082,3084,3169,3202,3212,3217,3491,3688,3714,3845,4040,4041,4086,4208,4300,4478,4638,4851,4919,5048,5100,5241,5361,5420,5469,5567,5573,5613,5629,5727,5728,5898,5911,6091,6092,6239,6256,6299,6491,6497,6654,6664,6722,6781,6788,6909,6928,7020,7042,7046,7048,7074,7168,7248,7291,7403,7421,7424,7430,7547,7852,8013,8289,8321,8828,8829,9021,9231,9314,9353,9496,9620,9723,9788,9839,9863,10082,10116,10252,10253,10277,10395,10427,10672,10818,10890,10959,11146,23002,23122,23213,23242,23332,23414,23493,23767,25928,26249,27063,27302,28514,29072,30812,51804,54567,54806,55083,55327,55366,55636,55727,55966,56998,57154,57167,57493,57534,57619,57822,59277,64398,64399,64750,64856,65979,79977,80000,80004,81578,81839,84133,84678,85407,90627,94134,115908,129446,130497,144165,153572,157506,166336,284217 | ABL1,ACVR1,AP2B1,JAG1,AR,RHOA,BMP2,BMP7,BMPR1A,BMPR2,CD44,CDC42,AP2M1,CLTC,COL3A1,COL4A1,COL5A1,COL12A1,NKX2-5,CTNNB1,DLG1,DVL3,S1PR1,EGFR,EPHA4,EPHA7,ETV5,EYA1,FGF7,FGF10,FGFR2,FOXF1,FOXF2,FOXD1,MTOR,GATA3,GATA4,GDNF,GLI2,GLI3,GRB2,HGF,NRG1,FOXA1,HOXA5,HOXB2,HOXB7,CCN1,ITGB1,JAG2,KRAS,LRP6,LRP5,SMAD1,MEF2C,MLLT3,MSN,MYLK,NOTCH1,ROR1,PAFAH1B1,PCDH8,PGR,PLXNA1,PODXL,MED1,PRKACB,PRKAR1A,PRKX,PROX1,PTCH1,PTEN,RALA,RAP2A,ROBO1,ROBO2,RREB1,RXRA,SALL1,STIL,SKI,SOS1,SOX11,SRF,STC1,STK3,TBX2,HNF1B,TFAP2A,TGFB2,TGFBR1,TGFBR2,TIAM1,TPM1,TSC1,TWIST1,KDM6A,VDR,VEGFC,EZR,ZIC3,CXCR4,NR4A3,ARID1A,FZD1,NRP2,NRP1,SOCS3,DLG5,KLF4,SLIT2,TBX4,CELSR1,SEMA3E,MTSS1,ZEB2,MAGI2,GPC6,FEM1B,SPRY1,SPRY2,UBE4B,DLC1,SEC24B,GNA13,FRS2,RAB10,TMED2,GLMN,DAAM1,CLASP2,SULF1,COBL,CLASP1,ZFPM2,HEY2,FLRT3,SOSTDC1,KLHL3,ANKRD1,BMP10,DLL1,SETD2,SOX8,SIX4,DLL4,AHI1,KIF26B,LIN7C,LGR4,CHD7,BTBD7,AJAP1,CTNNBIP1,SMURF1,SALL4,HEG1,MIB1,SHROOM3,GRHL3,NTN4,MPP5,HHIP,SMURF2,VWA1,PHACTR4,GRHL2,GREB1L,ESRP2,COL21A1,VANGL1,ZNRF3,KDM2B,NKD1,STARD13,ARHGAP12,CTHRC1,XIRP2,OSR1,PRICKLE1,IRX2,RDH10,PRICKLE2,LAMA1 |
| 6_Member | GO Biological Processes | GO:0002009 | morphogenesis of an epithelium | -32.86831732 | -29.819 | 157/540 | 25,90,163,182,367,387,650,655,960,998,1173,1213,1282,1289,1482,1499,1739,1857,1956,2043,2045,2119,2138,2252,2255,2263,2294,2295,2297,2475,2625,2626,2668,2736,2737,2885,3082,3169,3202,3212,3217,3491,3714,3845,4040,4041,4208,4300,4478,4851,4919,5048,5100,5241,5361,5420,5469,5567,5613,5629,5727,5728,5898,5911,6239,6256,6299,6491,6497,6654,6664,6722,6788,6909,6928,7020,7042,7048,7074,7248,7291,7403,7421,7424,7430,7547,7852,8289,8321,8829,9021,9231,9314,9353,9496,9620,9723,9788,9839,9863,10082,10116,10252,10253,10395,10427,10672,10818,10890,10959,11146,23002,23122,23213,23242,23332,23767,25928,26249,28514,29072,30812,51804,54567,54806,55083,55327,55366,55727,55966,56998,57154,57167,57534,57619,57822,59277,64398,64399,64750,65979,79977,80000,80004,81839,84133,84678,85407,90627,94134,115908,130497,144165,153572,157506,166336,284217 | ABL1,ACVR1,AP2B1,JAG1,AR,RHOA,BMP2,BMP7,CD44,CDC42,AP2M1,CLTC,COL4A1,COL5A1,NKX2-5,CTNNB1,DLG1,DVL3,EGFR,EPHA4,EPHA7,ETV5,EYA1,FGF7,FGF10,FGFR2,FOXF1,FOXF2,FOXD1,MTOR,GATA3,GATA4,GDNF,GLI2,GLI3,GRB2,HGF,FOXA1,HOXA5,HOXB2,HOXB7,CCN1,JAG2,KRAS,LRP6,LRP5,MEF2C,MLLT3,MSN,NOTCH1,ROR1,PAFAH1B1,PCDH8,PGR,PLXNA1,PODXL,MED1,PRKACB,PRKX,PROX1,PTCH1,PTEN,RALA,RAP2A,RREB1,RXRA,SALL1,STIL,SKI,SOS1,SOX11,SRF,STK3,TBX2,HNF1B,TFAP2A,TGFB2,TGFBR2,TIAM1,TSC1,TWIST1,KDM6A,VDR,VEGFC,EZR,ZIC3,CXCR4,ARID1A,FZD1,NRP1,SOCS3,DLG5,KLF4,SLIT2,TBX4,CELSR1,SEMA3E,MTSS1,ZEB2,MAGI2,GPC6,FEM1B,SPRY1,SPRY2,DLC1,SEC24B,GNA13,FRS2,RAB10,TMED2,GLMN,DAAM1,CLASP2,SULF1,COBL,CLASP1,FLRT3,SOSTDC1,KLHL3,DLL1,SETD2,SOX8,SIX4,DLL4,AHI1,KIF26B,LIN7C,LGR4,BTBD7,AJAP1,CTNNBIP1,SMURF1,SALL4,MIB1,SHROOM3,GRHL3,NTN4,MPP5,HHIP,SMURF2,PHACTR4,GRHL2,GREB1L,ESRP2,VANGL1,ZNRF3,KDM2B,NKD1,STARD13,ARHGAP12,CTHRC1,OSR1,PRICKLE1,IRX2,RDH10,PRICKLE2,LAMA1 |
| 6_Member | GO Biological Processes | GO:0009792 | embryo development ending in birth or egg hatching | -29.79034613 | -26.820 | 170/646 | 25,90,92,102,118,284,320,321,367,639,650,655,657,659,677,860,1028,1482,1499,1739,1857,1889,1956,2033,2138,2182,2186,2263,2294,2494,2625,2736,2737,2885,3169,3198,3200,3202,3212,3217,3491,3688,3714,4010,4040,4097,4124,4154,4208,4223,4325,4627,4628,4643,4763,4848,4851,4863,5079,5100,5125,5155,5156,5362,5469,5567,5594,5604,5629,5727,5801,5898,5930,6256,6491,6497,6664,6670,6722,6788,6928,6935,7020,7042,7046,7048,7168,7248,7291,7403,8178,8289,8313,8321,8452,8533,8851,9021,9318,9508,9541,9620,9644,9839,9871,9927,10006,10087,10265,10395,10427,10436,10658,10672,10742,10959,11146,23081,23168,23213,23242,23250,23314,23414,23493,23648,25942,26115,27023,28514,29072,30812,51804,54799,55122,55553,55636,55746,55959,56243,56853,56977,57142,57167,57178,57448,57493,57534,57619,57822,60482,60626,64131,65979,79718,79733,79805,79977,80312,81565,84295,84541,84678,85407,115908,130497,130507,144165,157506,253260 | ABL1,ACVR1,ACVR2A,ADAM10,ADD1,ANGPT1,APBA1,APBA2,AR,PRDM1,BMP2,BMP7,BMPR1A,BMPR2,ZFP36L1,RUNX2,CDKN1C,NKX2-5,CTNNB1,DLG1,DVL3,ECE1,EGFR,EP300,EYA1,ACSL4,BPTF,FGFR2,FOXF1,NR5A2,GATA3,GLI2,GLI3,GRB2,FOXA1,HOXA1,HOXA3,HOXA5,HOXB2,HOXB7,CCN1,ITGB1,JAG2,LMX1B,LRP6,MAFG,MAN2A1,MBNL1,MEF2C,MEOX2,MMP16,MYH9,MYH10,MYO1E,NF1,CNOT2,NOTCH1,NPAT,PAX5,PCDH8,PCSK5,PDGFB,PDGFRA,PLXNA2,MED1,PRKACB,MAPK1,MAP2K1,PROX1,PTCH1,PTPRR,RALA,RBBP6,RXRA,STIL,SKI,SOX11,SP3,SRF,STK3,HNF1B,ZEB1,TFAP2A,TGFB2,TGFBR1,TGFBR2,TPM1,TSC1,TWIST1,KDM6A,ELL,ARID1A,AXIN2,FZD1,CUL3,COPS3,CDK5R1,SOCS3,COPS2,ADAMTS3,CIR1,CELSR1,SH3PXD2A,ZEB2,SEC24D,MFN2,ABI1,CERT1,IRX5,DLC1,SEC24B,EMG1,CELF1,GNA13,RAI2,TMED2,GLMN,KDM4C,RTF1,SULF1,COBL,ATP11A,SATB2,ZFPM2,HEY2,SSBP3,SIN3A,TANC2,FOXB1,DLL1,SETD2,SOX8,SIX4,MBTD1,AKIRIN2,SOX6,CHD7,NUP133,SULF2,KIAA1217,CELF4,STOX2,RTN4,SALL4,ZMIZ1,BIRC6,HEG1,MIB1,SHROOM3,GRHL3,SLC5A7,RIC8A,XYLT1,PHACTR4,TBL1XR1,E2F8,VASH2,GRHL2,TET1,NDEL1,PHF6,KBTBD8,KDM2B,NKD1,CTHRC1,OSR1,UBR3,PRICKLE1,RDH10,RICTOR |
| 6_Member | GO Biological Processes | GO:0043009 | chordate embryonic development | -27.25191674 | -24.433 | 162/629 | 25,90,92,102,118,284,320,321,367,639,650,655,657,659,677,860,1028,1482,1499,1739,1857,1889,1956,2033,2138,2186,2263,2294,2625,2736,2737,2885,3169,3198,3200,3202,3212,3217,3491,3688,3714,4010,4040,4097,4124,4154,4208,4223,4325,4627,4628,4643,4763,4848,4851,4863,5079,5100,5125,5155,5156,5362,5469,5567,5594,5604,5629,5727,5801,5898,5930,6256,6491,6497,6664,6670,6722,6788,6928,6935,7020,7042,7046,7048,7168,7248,7291,7403,8178,8289,8313,8321,8452,8533,9021,9318,9508,9541,9620,9644,9839,9871,9927,10006,10087,10265,10395,10427,10436,10672,10959,11146,23081,23168,23213,23242,23250,23314,23414,23493,23648,25942,26115,27023,28514,29072,30812,51804,54799,55553,55636,55746,55959,56243,56853,57142,57167,57178,57448,57493,57534,57619,57822,60482,60626,64131,65979,79718,79733,79805,79977,80312,81565,84295,84541,84678,85407,115908,130497,130507,144165,157506 | ABL1,ACVR1,ACVR2A,ADAM10,ADD1,ANGPT1,APBA1,APBA2,AR,PRDM1,BMP2,BMP7,BMPR1A,BMPR2,ZFP36L1,RUNX2,CDKN1C,NKX2-5,CTNNB1,DLG1,DVL3,ECE1,EGFR,EP300,EYA1,BPTF,FGFR2,FOXF1,GATA3,GLI2,GLI3,GRB2,FOXA1,HOXA1,HOXA3,HOXA5,HOXB2,HOXB7,CCN1,ITGB1,JAG2,LMX1B,LRP6,MAFG,MAN2A1,MBNL1,MEF2C,MEOX2,MMP16,MYH9,MYH10,MYO1E,NF1,CNOT2,NOTCH1,NPAT,PAX5,PCDH8,PCSK5,PDGFB,PDGFRA,PLXNA2,MED1,PRKACB,MAPK1,MAP2K1,PROX1,PTCH1,PTPRR,RALA,RBBP6,RXRA,STIL,SKI,SOX11,SP3,SRF,STK3,HNF1B,ZEB1,TFAP2A,TGFB2,TGFBR1,TGFBR2,TPM1,TSC1,TWIST1,KDM6A,ELL,ARID1A,AXIN2,FZD1,CUL3,COPS3,SOCS3,COPS2,ADAMTS3,CIR1,CELSR1,SH3PXD2A,ZEB2,SEC24D,MFN2,ABI1,CERT1,IRX5,DLC1,SEC24B,EMG1,GNA13,TMED2,GLMN,KDM4C,RTF1,SULF1,COBL,ATP11A,SATB2,ZFPM2,HEY2,SSBP3,SIN3A,TANC2,FOXB1,DLL1,SETD2,SOX8,SIX4,MBTD1,SOX6,CHD7,NUP133,SULF2,KIAA1217,CELF4,RTN4,SALL4,ZMIZ1,BIRC6,HEG1,MIB1,SHROOM3,GRHL3,SLC5A7,RIC8A,XYLT1,PHACTR4,TBL1XR1,E2F8,VASH2,GRHL2,TET1,NDEL1,PHF6,KBTBD8,KDM2B,NKD1,CTHRC1,OSR1,UBR3,PRICKLE1,RDH10 |
| 6_Member | GO Biological Processes | GO:0048598 | embryonic morphogenesis | -26.42500113 | -23.665 | 153/586 | 25,90,92,367,655,657,659,677,775,860,1028,1289,1303,1387,1482,1499,1739,1857,1889,2048,2138,2200,2201,2255,2263,2294,2295,2335,2625,2626,2668,2736,2737,2885,3170,3198,3200,3202,3206,3212,3217,3398,3491,3638,3688,3714,3899,4038,4040,4041,4086,4154,4208,4325,4760,4762,4851,5079,5100,5156,5469,5567,5573,5594,5629,5727,5898,6093,6299,6491,6497,6654,6657,6664,6670,6722,6788,6909,6928,6935,7020,7042,7046,7048,7248,7291,7403,7424,7547,8013,8289,8321,8434,8452,9021,9314,9475,9496,9620,9839,10194,10253,10265,10395,10427,10586,10818,10959,11146,23122,23168,23213,23242,23314,23332,23767,26018,28514,28996,29072,30812,51761,51804,54567,54806,54928,55084,55614,55636,57167,57534,57619,57822,60626,64388,65125,65979,79577,79977,84189,84678,115908,121227,123169,125488,130497,144165,153572,157506,204851,221833,348093,100131390 | ABL1,ACVR1,ACVR2A,AR,BMP7,BMPR1A,BMPR2,ZFP36L1,CACNA1C,RUNX2,CDKN1C,COL5A1,COL12A1,CREBBP,NKX2-5,CTNNB1,DLG1,DVL3,ECE1,EPHB2,EYA1,FBN1,FBN2,FGF10,FGFR2,FOXF1,FOXF2,FN1,GATA3,GATA4,GDNF,GLI2,GLI3,GRB2,FOXA2,HOXA1,HOXA3,HOXA5,HOXA10,HOXB2,HOXB7,ID2,CCN1,INSIG1,ITGB1,JAG2,AFF3,LRP4,LRP6,LRP5,SMAD1,MBNL1,MEF2C,MMP16,NEUROD1,NEUROG1,NOTCH1,PAX5,PCDH8,PDGFRA,MED1,PRKACB,PRKAR1A,MAPK1,PROX1,PTCH1,RALA,ROCK1,SALL1,STIL,SKI,SOS1,SOX2,SOX11,SP3,SRF,STK3,TBX2,HNF1B,ZEB1,TFAP2A,TGFB2,TGFBR1,TGFBR2,TSC1,TWIST1,KDM6A,VEGFC,ZIC3,NR4A3,ARID1A,FZD1,RECK,CUL3,SOCS3,KLF4,ROCK2,TBX4,CELSR1,ZEB2,TSHZ1,SPRY2,IRX5,DLC1,SEC24B,MAB21L2,FRS2,TMED2,GLMN,CLASP2,RTF1,SULF1,COBL,SATB2,CLASP1,FLRT3,LRIG1,DLL1,HIPK2,SETD2,SOX8,ATP8A2,SIX4,DLL4,AHI1,IMPAD1,SOBP,KIF16B,CHD7,SALL4,MIB1,SHROOM3,GRHL3,RIC8A,GREM2,WNK1,PHACTR4,CDC73,GRHL2,SLITRK6,KDM2B,CTHRC1,LRIG3,LEO1,TTC39C,OSR1,PRICKLE1,IRX2,RDH10,HIPK1,SP8,RBPMS2,SP9 |
| 6_Member | GO Biological Processes | GO:0060562 | epithelial tube morphogenesis | -24.05313401 | -21.451 | 101/322 | 25,90,367,387,650,655,1282,1482,1499,1739,1857,2043,2045,2119,2138,2255,2263,2294,2297,2625,2626,2668,2736,2737,3169,3202,3217,3845,4040,4041,4208,4851,5241,5420,5469,5567,5613,5629,5727,5898,6299,6491,6497,6664,6722,6788,6909,6928,7042,7048,7248,7291,7403,7421,7547,7852,8289,8321,8829,9231,9353,9620,9723,9788,9839,10252,10253,10395,10427,10672,10959,11146,23242,25928,26249,28514,29072,30812,51804,54567,54806,55083,55366,56998,57167,57534,57619,57822,64399,65979,79977,80000,80004,84678,90627,115908,130497,144165,153572,157506,284217 | ABL1,ACVR1,AR,RHOA,BMP2,BMP7,COL4A1,NKX2-5,CTNNB1,DLG1,DVL3,EPHA4,EPHA7,ETV5,EYA1,FGF10,FGFR2,FOXF1,FOXD1,GATA3,GATA4,GDNF,GLI2,GLI3,FOXA1,HOXA5,HOXB7,KRAS,LRP6,LRP5,MEF2C,NOTCH1,PGR,PODXL,MED1,PRKACB,PRKX,PROX1,PTCH1,RALA,SALL1,STIL,SKI,SOX11,SRF,STK3,TBX2,HNF1B,TGFB2,TGFBR2,TSC1,TWIST1,KDM6A,VDR,ZIC3,CXCR4,ARID1A,FZD1,NRP1,DLG5,SLIT2,CELSR1,SEMA3E,MTSS1,ZEB2,SPRY1,SPRY2,DLC1,SEC24B,GNA13,TMED2,GLMN,COBL,SOSTDC1,KLHL3,DLL1,SETD2,SOX8,SIX4,DLL4,AHI1,KIF26B,LGR4,CTNNBIP1,SALL4,MIB1,SHROOM3,GRHL3,HHIP,PHACTR4,GRHL2,GREB1L,ESRP2,KDM2B,STARD13,CTHRC1,OSR1,PRICKLE1,IRX2,RDH10,LAMA1 |
| 6_Member | GO Biological Processes | GO:0001763 | morphogenesis of a branching structure | -19.71093372 | -17.319 | 69/196 | 25,90,367,473,639,650,655,1282,1305,1499,1739,2045,2119,2138,2252,2255,2263,2294,2297,2668,2736,2737,2885,3082,3169,3202,3217,3845,4040,4041,4851,5241,5361,5469,5629,5727,6256,6299,6722,6928,7048,7421,8829,9021,9231,9353,9620,9723,10116,10252,10253,10672,10818,23213,29072,30812,51804,54567,55366,55727,56998,57142,59277,64399,79977,80000,80004,157506,284217 | ABL1,ACVR1,AR,RERE,PRDM1,BMP2,BMP7,COL4A1,COL13A1,CTNNB1,DLG1,EPHA7,ETV5,EYA1,FGF7,FGF10,FGFR2,FOXF1,FOXD1,GDNF,GLI2,GLI3,GRB2,HGF,FOXA1,HOXA5,HOXB7,KRAS,LRP6,LRP5,NOTCH1,PGR,PLXNA1,MED1,PROX1,PTCH1,RXRA,SALL1,SRF,HNF1B,TGFBR2,VDR,NRP1,SOCS3,DLG5,SLIT2,CELSR1,SEMA3E,FEM1B,SPRY1,SPRY2,GNA13,FRS2,SULF1,SETD2,SOX8,SIX4,DLL4,LGR4,BTBD7,CTNNBIP1,RTN4,NTN4,HHIP,GRHL2,GREB1L,ESRP2,RDH10,LAMA1 |
| 6_Member | GO Biological Processes | GO:0048568 | embryonic organ development | -17.98589373 | -15.669 | 109/429 | 90,639,655,657,677,860,1028,1482,1499,1739,1956,2048,2138,2186,2200,2201,2255,2263,2294,2295,2625,2626,2668,2736,2737,2885,3198,3200,3202,3212,3217,3398,3491,3638,3815,4040,4208,4325,4760,4762,4851,5079,5125,5155,5156,5469,5594,5604,5629,5727,5930,6299,6491,6664,6670,6722,6788,6909,6928,6935,7020,7042,7046,7048,7291,7403,7547,8013,8289,9021,9620,10194,10253,10265,10427,10818,10959,23242,23314,23414,23493,26018,28514,28996,29072,51761,51804,54806,55084,55636,56034,57448,57534,57599,57822,65979,79733,79805,79977,84189,84678,115908,121227,125488,130497,157506,196528,204851,348093 | ACVR1,PRDM1,BMP7,BMPR1A,ZFP36L1,RUNX2,CDKN1C,NKX2-5,CTNNB1,DLG1,EGFR,EPHB2,EYA1,BPTF,FBN1,FBN2,FGF10,FGFR2,FOXF1,FOXF2,GATA3,GATA4,GDNF,GLI2,GLI3,GRB2,HOXA1,HOXA3,HOXA5,HOXB2,HOXB7,ID2,CCN1,INSIG1,KIT,LRP6,MEF2C,MMP16,NEUROD1,NEUROG1,NOTCH1,PAX5,PCSK5,PDGFB,PDGFRA,MED1,MAPK1,MAP2K1,PROX1,PTCH1,RBBP6,SALL1,STIL,SOX11,SP3,SRF,STK3,TBX2,HNF1B,ZEB1,TFAP2A,TGFB2,TGFBR1,TGFBR2,TWIST1,KDM6A,ZIC3,NR4A3,ARID1A,SOCS3,CELSR1,TSHZ1,SPRY2,IRX5,SEC24B,FRS2,TMED2,COBL,SATB2,ZFPM2,HEY2,LRIG1,DLL1,HIPK2,SETD2,ATP8A2,SIX4,AHI1,SOBP,CHD7,PDGFC,BIRC6,MIB1,WDR48,GRHL3,PHACTR4,E2F8,VASH2,GRHL2,SLITRK6,KDM2B,CTHRC1,LRIG3,TTC39C,OSR1,RDH10,ARID2,HIPK1,RBPMS2 |
| 6_Member | GO Biological Processes | GO:0061138 | morphogenesis of a branching epithelium | -17.63259342 | -15.329 | 63/182 | 25,90,367,650,655,1282,1499,1739,2119,2138,2252,2255,2263,2294,2297,2668,2736,2737,2885,3082,3169,3202,3217,3845,4040,4041,4851,5241,5361,5469,5629,5727,6256,6299,6722,6928,7048,7421,8829,9021,9231,9353,9620,9723,10116,10252,10253,10672,10818,23213,30812,51804,54567,55366,55727,56998,59277,64399,79977,80000,80004,157506,284217 | ABL1,ACVR1,AR,BMP2,BMP7,COL4A1,CTNNB1,DLG1,ETV5,EYA1,FGF7,FGF10,FGFR2,FOXF1,FOXD1,GDNF,GLI2,GLI3,GRB2,HGF,FOXA1,HOXA5,HOXB7,KRAS,LRP6,LRP5,NOTCH1,PGR,PLXNA1,MED1,PROX1,PTCH1,RXRA,SALL1,SRF,HNF1B,TGFBR2,VDR,NRP1,SOCS3,DLG5,SLIT2,CELSR1,SEMA3E,FEM1B,SPRY1,SPRY2,GNA13,FRS2,SULF1,SOX8,SIX4,DLL4,LGR4,BTBD7,CTNNBIP1,NTN4,HHIP,GRHL2,GREB1L,ESRP2,RDH10,LAMA1 |
| 6_Member | GO Biological Processes | GO:0001701 | in utero embryonic development | -16.62558901 | -14.392 | 97/375 | 90,102,118,284,320,321,367,639,650,655,657,677,1028,1499,1956,2186,2263,2294,2625,2736,2737,2885,3491,3688,3714,4010,4097,4124,4154,4627,4628,4643,4848,4851,4863,5155,5156,5469,5594,5604,5727,5801,5930,6256,6491,6670,6722,6788,6928,7046,7048,7168,7291,7403,8178,8452,8533,9021,9318,9508,9541,9644,9871,9927,10087,10436,10672,10959,23081,23168,23250,23414,23493,25942,26115,29072,30812,55553,55636,56853,57142,57167,57178,57448,57493,57534,60482,60626,79718,79733,79805,79977,80312,81565,84295,130507,157506 | ACVR1,ADAM10,ADD1,ANGPT1,APBA1,APBA2,AR,PRDM1,BMP2,BMP7,BMPR1A,ZFP36L1,CDKN1C,CTNNB1,EGFR,BPTF,FGFR2,FOXF1,GATA3,GLI2,GLI3,GRB2,CCN1,ITGB1,JAG2,LMX1B,MAFG,MAN2A1,MBNL1,MYH9,MYH10,MYO1E,CNOT2,NOTCH1,NPAT,PDGFB,PDGFRA,MED1,MAPK1,MAP2K1,PTCH1,PTPRR,RBBP6,RXRA,STIL,SP3,SRF,STK3,HNF1B,TGFBR1,TGFBR2,TPM1,TWIST1,KDM6A,ELL,CUL3,COPS3,SOCS3,COPS2,ADAMTS3,CIR1,SH3PXD2A,SEC24D,MFN2,CERT1,EMG1,GNA13,TMED2,KDM4C,RTF1,ATP11A,ZFPM2,HEY2,SIN3A,TANC2,SETD2,SOX8,SOX6,CHD7,CELF4,RTN4,SALL4,ZMIZ1,BIRC6,HEG1,MIB1,SLC5A7,RIC8A,TBL1XR1,E2F8,VASH2,GRHL2,TET1,NDEL1,PHF6,UBR3,RDH10 |
| 6_Member | GO Biological Processes | GO:0048562 | embryonic organ morphogenesis | -16.24671839 | -14.036 | 81/288 | 90,655,860,1482,1499,1739,2048,2138,2200,2201,2255,2263,2294,2295,2625,2626,2736,2737,3198,3200,3202,3212,3217,3398,3638,4040,4208,4325,4760,4762,4851,5079,5156,5594,5629,6299,6491,6664,6670,6722,6909,6928,6935,7020,7046,7048,7291,7403,7547,8013,8289,9620,10194,10253,10265,10427,10818,10959,23314,26018,28514,28996,29072,51761,51804,54806,55084,55636,57534,57822,65979,79977,84189,84678,115908,121227,125488,130497,157506,204851,348093 | ACVR1,BMP7,RUNX2,NKX2-5,CTNNB1,DLG1,EPHB2,EYA1,FBN1,FBN2,FGF10,FGFR2,FOXF1,FOXF2,GATA3,GATA4,GLI2,GLI3,HOXA1,HOXA3,HOXA5,HOXB2,HOXB7,ID2,INSIG1,LRP6,MEF2C,MMP16,NEUROD1,NEUROG1,NOTCH1,PAX5,PDGFRA,MAPK1,PROX1,SALL1,STIL,SOX11,SP3,SRF,TBX2,HNF1B,ZEB1,TFAP2A,TGFBR1,TGFBR2,TWIST1,KDM6A,ZIC3,NR4A3,ARID1A,CELSR1,TSHZ1,SPRY2,IRX5,SEC24B,FRS2,TMED2,SATB2,LRIG1,DLL1,HIPK2,SETD2,ATP8A2,SIX4,AHI1,SOBP,CHD7,MIB1,GRHL3,PHACTR4,GRHL2,SLITRK6,KDM2B,CTHRC1,LRIG3,TTC39C,OSR1,RDH10,HIPK1,RBPMS2 |
| 6_Member | GO Biological Processes | GO:0016331 | morphogenesis of embryonic epithelium | -14.70861692 | -12.608 | 52/150 | 25,367,655,1499,1857,2255,2263,2625,2668,2736,3714,4040,5100,5567,5727,5898,6491,6497,6664,6788,6928,7020,7042,7248,7291,7403,7424,8289,8321,9620,9839,10395,10427,10959,11146,23213,23242,29072,30812,51804,57167,57534,57619,57822,65979,79977,84678,115908,130497,144165,153572,157506 | ABL1,AR,BMP7,CTNNB1,DVL3,FGF10,FGFR2,GATA3,GDNF,GLI2,JAG2,LRP6,PCDH8,PRKACB,PTCH1,RALA,STIL,SKI,SOX11,STK3,HNF1B,TFAP2A,TGFB2,TSC1,TWIST1,KDM6A,VEGFC,ARID1A,FZD1,CELSR1,ZEB2,DLC1,SEC24B,TMED2,GLMN,SULF1,COBL,SETD2,SOX8,SIX4,SALL4,MIB1,SHROOM3,GRHL3,PHACTR4,GRHL2,KDM2B,CTHRC1,OSR1,PRICKLE1,IRX2,RDH10 |
| 6_Member | GO Biological Processes | GO:0048754 | branching morphogenesis of an epithelial tube | -13.40175393 | -11.373 | 50/150 | 25,90,367,650,655,1282,1499,1739,2119,2138,2255,2263,2294,2297,2668,2736,2737,3169,3202,3217,3845,4040,4041,4851,5241,5469,5727,6299,6722,6928,7048,7421,8829,9231,9353,9620,9723,10252,10253,10672,30812,51804,54567,55366,56998,64399,80000,80004,157506,284217 | ABL1,ACVR1,AR,BMP2,BMP7,COL4A1,CTNNB1,DLG1,ETV5,EYA1,FGF10,FGFR2,FOXF1,FOXD1,GDNF,GLI2,GLI3,FOXA1,HOXA5,HOXB7,KRAS,LRP6,LRP5,NOTCH1,PGR,MED1,PTCH1,SALL1,SRF,HNF1B,TGFBR2,VDR,NRP1,DLG5,SLIT2,CELSR1,SEMA3E,SPRY1,SPRY2,GNA13,SOX8,SIX4,DLL4,LGR4,CTNNBIP1,HHIP,GREB1L,ESRP2,RDH10,LAMA1 |
| 6_Member | GO Biological Processes | GO:0072175 | epithelial tube formation | -13.19033914 | -11.174 | 46/132 | 25,655,1499,1857,2255,2263,2625,2668,4040,5420,5567,5629,5727,5898,6491,6497,6664,6788,6928,7042,7248,7291,7403,8289,8321,9620,9839,10395,10427,10959,11146,23242,29072,30812,51804,57167,57534,57619,57822,65979,79977,84678,115908,130497,144165,153572 | ABL1,BMP7,CTNNB1,DVL3,FGF10,FGFR2,GATA3,GDNF,LRP6,PODXL,PRKACB,PROX1,PTCH1,RALA,STIL,SKI,SOX11,STK3,HNF1B,TGFB2,TSC1,TWIST1,KDM6A,ARID1A,FZD1,CELSR1,ZEB2,DLC1,SEC24B,TMED2,GLMN,COBL,SETD2,SOX8,SIX4,SALL4,MIB1,SHROOM3,GRHL3,PHACTR4,GRHL2,KDM2B,CTHRC1,OSR1,PRICKLE1,IRX2 |
| 6_Member | GO Biological Processes | GO:0035148 | tube formation | -13.01990017 | -11.016 | 49/148 | 25,655,1499,1857,2255,2263,2625,2668,3198,4040,4781,4851,5420,5567,5629,5727,5898,6491,6497,6664,6788,6928,7042,7248,7291,7403,8289,8321,9620,9839,10395,10427,10959,11146,23242,29072,30812,51804,57167,57534,57619,57822,65979,79977,84678,115908,130497,144165,153572 | ABL1,BMP7,CTNNB1,DVL3,FGF10,FGFR2,GATA3,GDNF,HOXA1,LRP6,NFIB,NOTCH1,PODXL,PRKACB,PROX1,PTCH1,RALA,STIL,SKI,SOX11,STK3,HNF1B,TGFB2,TSC1,TWIST1,KDM6A,ARID1A,FZD1,CELSR1,ZEB2,DLC1,SEC24B,TMED2,GLMN,COBL,SETD2,SOX8,SIX4,SALL4,MIB1,SHROOM3,GRHL3,PHACTR4,GRHL2,KDM2B,CTHRC1,OSR1,PRICKLE1,IRX2 |
| 6_Member | GO Biological Processes | GO:0001838 | embryonic epithelial tube formation | -11.63430853 | -9.731 | 42/124 | 25,655,1499,1857,2625,2668,4040,5567,5727,5898,6491,6497,6664,6788,6928,7042,7248,7291,7403,8289,8321,9620,9839,10395,10427,10959,11146,23242,29072,30812,51804,57167,57534,57619,57822,65979,79977,84678,115908,130497,144165,153572 | ABL1,BMP7,CTNNB1,DVL3,GATA3,GDNF,LRP6,PRKACB,PTCH1,RALA,STIL,SKI,SOX11,STK3,HNF1B,TGFB2,TSC1,TWIST1,KDM6A,ARID1A,FZD1,CELSR1,ZEB2,DLC1,SEC24B,TMED2,GLMN,COBL,SETD2,SOX8,SIX4,SALL4,MIB1,SHROOM3,GRHL3,PHACTR4,GRHL2,KDM2B,CTHRC1,OSR1,PRICKLE1,IRX2 |
| 6_Member | GO Biological Processes | GO:0021915 | neural tube development | -9.341473423 | -7.591 | 45/160 | 25,655,677,1857,2736,2737,3169,4040,4763,4851,5362,5567,5629,5727,5898,6491,6497,6664,6788,7042,7248,7291,7403,8289,8321,9620,9839,10395,10427,10959,11146,23242,23648,27023,29072,55746,57167,57534,57619,57822,65979,79977,84678,115908,144165 | ABL1,BMP7,ZFP36L1,DVL3,GLI2,GLI3,FOXA1,LRP6,NF1,NOTCH1,PLXNA2,PRKACB,PROX1,PTCH1,RALA,STIL,SKI,SOX11,STK3,TGFB2,TSC1,TWIST1,KDM6A,ARID1A,FZD1,CELSR1,ZEB2,DLC1,SEC24B,TMED2,GLMN,COBL,SSBP3,FOXB1,SETD2,NUP133,SALL4,MIB1,SHROOM3,GRHL3,PHACTR4,GRHL2,KDM2B,CTHRC1,PRICKLE1 |
| 6_Member | GO Biological Processes | GO:0001841 | neural tube formation | -8.964503142 | -7.249 | 34/105 | 25,655,1857,4040,5567,5727,5898,6491,6497,6664,6788,7042,7248,7291,7403,8289,8321,9620,9839,10395,10427,10959,11146,23242,29072,57167,57534,57619,57822,65979,79977,84678,115908,144165 | ABL1,BMP7,DVL3,LRP6,PRKACB,PTCH1,RALA,STIL,SKI,SOX11,STK3,TGFB2,TSC1,TWIST1,KDM6A,ARID1A,FZD1,CELSR1,ZEB2,DLC1,SEC24B,TMED2,GLMN,COBL,SETD2,SALL4,MIB1,SHROOM3,GRHL3,PHACTR4,GRHL2,KDM2B,CTHRC1,PRICKLE1 |
| 6_Member | GO Biological Processes | GO:0060606 | tube closure | -8.842300466 | -7.138 | 31/91 | 25,1857,2255,4040,5567,5727,5898,6491,6497,7042,7248,7291,7403,8289,8321,9620,9839,10395,10427,10959,11146,23242,29072,57167,57619,57822,65979,79977,84678,115908,144165 | ABL1,DVL3,FGF10,LRP6,PRKACB,PTCH1,RALA,STIL,SKI,TGFB2,TSC1,TWIST1,KDM6A,ARID1A,FZD1,CELSR1,ZEB2,DLC1,SEC24B,TMED2,GLMN,COBL,SETD2,SALL4,SHROOM3,GRHL3,PHACTR4,GRHL2,KDM2B,CTHRC1,PRICKLE1 |
| 6_Member | GO Biological Processes | GO:0001843 | neural tube closure | -8.325129605 | -6.673 | 30/90 | 25,1857,4040,5567,5727,5898,6491,6497,7042,7248,7291,7403,8289,8321,9620,9839,10395,10427,10959,11146,23242,29072,57167,57619,57822,65979,79977,84678,115908,144165 | ABL1,DVL3,LRP6,PRKACB,PTCH1,RALA,STIL,SKI,TGFB2,TSC1,TWIST1,KDM6A,ARID1A,FZD1,CELSR1,ZEB2,DLC1,SEC24B,TMED2,GLMN,COBL,SETD2,SALL4,SHROOM3,GRHL3,PHACTR4,GRHL2,KDM2B,CTHRC1,PRICKLE1 |
| 6_Member | GO Biological Processes | GO:0014020 | primary neural tube formation | -8.081260938 | -6.451 | 31/97 | 25,655,1857,4040,5567,5727,5898,6491,6497,7042,7248,7291,7403,8289,8321,9620,9839,10395,10427,10959,11146,23242,29072,57167,57619,57822,65979,79977,84678,115908,144165 | ABL1,BMP7,DVL3,LRP6,PRKACB,PTCH1,RALA,STIL,SKI,TGFB2,TSC1,TWIST1,KDM6A,ARID1A,FZD1,CELSR1,ZEB2,DLC1,SEC24B,TMED2,GLMN,COBL,SETD2,SALL4,SHROOM3,GRHL3,PHACTR4,GRHL2,KDM2B,CTHRC1,PRICKLE1 |
| 7_Summary | GO Biological Processes | GO:0048589 | developmental growth | -31.40397094 | -28.396 | 178/674 | 25,118,320,321,351,367,546,607,657,659,677,688,1027,1028,1303,1385,1482,1499,1808,1826,1848,1901,1946,2017,2033,2045,2066,2252,2255,2261,2263,2335,2475,2619,2625,2626,2736,2737,3202,3643,3688,3720,3776,3984,4038,4040,4086,4131,4133,4208,4534,4803,4851,4856,4897,5048,5144,5292,5361,5469,5530,5573,5594,5629,5727,5728,5782,5930,5999,6256,6299,6416,6491,6654,6657,6722,6781,6788,6857,6860,6909,6928,7042,7046,7048,7074,7161,7224,7337,7403,7430,7528,7852,8239,8408,8455,8470,8766,8828,8829,8851,8936,9037,9201,9254,9318,9353,9472,9699,9706,9723,9798,9839,9863,10252,10253,10479,10507,10631,10939,10959,11221,22941,22999,23011,23168,23236,23242,23286,23327,23394,23414,23493,23767,25937,25942,26053,26058,26281,26524,26999,27302,28514,50618,51761,51804,54796,55083,55512,55636,55777,55841,57142,57154,57167,57178,57282,57468,57493,57556,57578,57599,59338,64856,79718,79977,80014,80031,81565,81578,84159,84189,85407,114798,136319,157506,196528,375790,102,387,390,960,998,1123,1398,1399,1600,1739,1857,2035,2043,2048,2059,2185,2332,2534,3037,3815,4076,4478,4627,4628,4651,4734,4915,5063,5168,5362,5604,5649,5789,5911,5921,5962,6091,6092,6239,6383,7101,7168,7533,7750,9202,9693,10109,10152,10395,10458,10672,10810,11141,22871,23072,23077,23098,23189,23603,26039,30011,55023,55435,55607,55660,55785,56990,57194,57381,57520,57619,57688,57689,58480,65981,93663,113251,118987,121512,140578,152503,157922,192670,254065,323,604,694,817,1654,1956,1982,2023,3490,4853,5062,5270,5515,5588,6195,6197,6695,8089,8725,9021,9211,9306,9538,9655,9792,10276,10477,22883,23251,23411,23677,26153,28985,29967,51009,51542,54617,55004,55031,55602,57521,79577,79602,80314,83452,83737,84162,84289,84312,84678,123879,131566,361,1182,1874,6558,7248,9821,10000,23108 | ABL1,ADD1,APBA1,APBA2,APP,AR,ATRX,BCL9,BMPR1A,BMPR2,ZFP36L1,KLF5,CDKN1B,CDKN1C,COL12A1,CREB1,NKX2-5,CTNNB1,DPYSL2,DSCAM,DUSP6,S1PR1,EFNA5,CTTN,EP300,EPHA7,ERBB4,FGF7,FGF10,FGFR3,FGFR2,FN1,MTOR,GAS1,GATA3,GATA4,GLI2,GLI3,HOXA5,INSR,ITGB1,JARID2,KCNK2,LIMK1,LRP4,LRP6,SMAD1,MAP1B,MAP2,MEF2C,MTM1,NGF,NOTCH1,CCN3,NRCAM,PAFAH1B1,PDE4D,PIM1,PLXNA1,MED1,PPP3CA,PRKAR1A,MAPK1,PROX1,PTCH1,PTEN,PTPN12,RBBP6,RGS4,RXRA,SALL1,MAP2K4,STIL,SOS1,SOX2,SRF,STC1,STK3,SYT1,SYT4,TBX2,HNF1B,TGFB2,TGFBR1,TGFBR2,TIAM1,TP73,TRPC5,UBE3A,KDM6A,EZR,YY1,CXCR4,USP9X,ULK1,ATRN,SORBS2,RAB11A,NRP2,NRP1,CDK5R1,WASF1,SEMA5A,DCLK1,CACNA2D2,COPS2,SLIT2,AKAP6,RIMS2,ULK2,SEMA3E,IST1,ZEB2,MAGI2,SPRY1,SPRY2,SLC9A6,SEMA4D,POSTN,AFG3L2,TMED2,DUSP10,SHANK2,RIMS1,RAB21,RTF1,PLCB1,COBL,WWC1,NEDD4L,ADNP,ZFPM2,HEY2,FLRT3,WWTR1,SIN3A,AUTS2,GIGYF2,FGF20,LATS2,CYFIP2,BMP10,DLL1,ITSN2,ATP8A2,SIX4,BNC2,KIF26B,SMPD3,CHD7,MBD5,WWC3,RTN4,SMURF1,SALL4,ZMIZ1,SLC4A10,SLC12A5,HEG1,SEMA6A,UNC79,WDR48,PLEKHA1,VWA1,TBL1XR1,GRHL2,WWC2,SEMA6D,NDEL1,COL21A1,ARID5B,SLITRK6,NKD1,SLITRK1,MTPN,RDH10,ARID2,AGRN,ADAM10,RHOA,RND3,CD44,CDC42,CHN1,CRK,CRKL,DAB1,DLG1,DVL3,EPB41,EPHA4,EPHB2,EPS8,PTK2B,FMR1,FYN,HAS2,KIT,CAPRIN1,MSN,MYH9,MYH10,MYO10,NEDD4,NTRK2,PAK3,ENPP2,PLXNA2,MAP2K1,RELN,PTPRD,RAP2A,RASA1,RDX,ROBO1,ROBO2,RREB1,SDC2,NR2E1,TPM1,YWHAH,ZMYM2,ZMYM4,RAPGEF2,ARPC2,ABI2,DLC1,BAIAP2,GNA13,WASF3,IL1RAPL1,NLGN1,HECW1,MYCBP2,SARM1,KANK1,CORO1C,SS18L1,SH3KBP1,PHIP,AP1AR,PPP1R9A,PRPF40A,FGD6,CDC42SE2,ATP10A,RHOJ,HECW2,SHROOM3,ZSWIM6,LRRC4C,RHOU,CAPRIN2,ARHGAP18,LARP4,PDZD8,FGD4,CHODL,SH3D19,CAMSAP1,AGO4,BRWD3,APBB2,BCL6,BTG1,CAMK2D,DDX3X,EGFR,EIF4G2,ENO1,IGFBP7,NOTCH2,PAK2,SERPINE2,PPP2CA,PRKCQ,RPS6KA1,RPS6KA3,SPOCK1,YEATS4,URI1,SOCS3,LGI1,SOCS6,EI24,SOCS5,SERTAD2,NET1,UBE2E3,CLSTN1,MINAR1,SIRT1,SH3BP4,KIF26A,MCTS1,LRP12,DERL2,VPS54,INO80,LAMTOR1,USP47,CDKN2AIP,RPTOR,CDC73,ADIPOR2,EPC1,RAB33B,ITCH,KIAA1109,ING5,BRMS1L,KDM2B,DCUN1D3,DCBLD2,AQP4,CLCN3,E2F4,SLC12A2,TSC1,RB1CC1,AKT3,RAP1GAP2 |
| 7_Member | GO Biological Processes | GO:0048589 | developmental growth | -31.40397094 | -28.396 | 178/674 | 25,118,320,321,351,367,546,607,657,659,677,688,1027,1028,1303,1385,1482,1499,1808,1826,1848,1901,1946,2017,2033,2045,2066,2252,2255,2261,2263,2335,2475,2619,2625,2626,2736,2737,3202,3643,3688,3720,3776,3984,4038,4040,4086,4131,4133,4208,4534,4803,4851,4856,4897,5048,5144,5292,5361,5469,5530,5573,5594,5629,5727,5728,5782,5930,5999,6256,6299,6416,6491,6654,6657,6722,6781,6788,6857,6860,6909,6928,7042,7046,7048,7074,7161,7224,7337,7403,7430,7528,7852,8239,8408,8455,8470,8766,8828,8829,8851,8936,9037,9201,9254,9318,9353,9472,9699,9706,9723,9798,9839,9863,10252,10253,10479,10507,10631,10939,10959,11221,22941,22999,23011,23168,23236,23242,23286,23327,23394,23414,23493,23767,25937,25942,26053,26058,26281,26524,26999,27302,28514,50618,51761,51804,54796,55083,55512,55636,55777,55841,57142,57154,57167,57178,57282,57468,57493,57556,57578,57599,59338,64856,79718,79977,80014,80031,81565,81578,84159,84189,85407,114798,136319,157506,196528,375790 | ABL1,ADD1,APBA1,APBA2,APP,AR,ATRX,BCL9,BMPR1A,BMPR2,ZFP36L1,KLF5,CDKN1B,CDKN1C,COL12A1,CREB1,NKX2-5,CTNNB1,DPYSL2,DSCAM,DUSP6,S1PR1,EFNA5,CTTN,EP300,EPHA7,ERBB4,FGF7,FGF10,FGFR3,FGFR2,FN1,MTOR,GAS1,GATA3,GATA4,GLI2,GLI3,HOXA5,INSR,ITGB1,JARID2,KCNK2,LIMK1,LRP4,LRP6,SMAD1,MAP1B,MAP2,MEF2C,MTM1,NGF,NOTCH1,CCN3,NRCAM,PAFAH1B1,PDE4D,PIM1,PLXNA1,MED1,PPP3CA,PRKAR1A,MAPK1,PROX1,PTCH1,PTEN,PTPN12,RBBP6,RGS4,RXRA,SALL1,MAP2K4,STIL,SOS1,SOX2,SRF,STC1,STK3,SYT1,SYT4,TBX2,HNF1B,TGFB2,TGFBR1,TGFBR2,TIAM1,TP73,TRPC5,UBE3A,KDM6A,EZR,YY1,CXCR4,USP9X,ULK1,ATRN,SORBS2,RAB11A,NRP2,NRP1,CDK5R1,WASF1,SEMA5A,DCLK1,CACNA2D2,COPS2,SLIT2,AKAP6,RIMS2,ULK2,SEMA3E,IST1,ZEB2,MAGI2,SPRY1,SPRY2,SLC9A6,SEMA4D,POSTN,AFG3L2,TMED2,DUSP10,SHANK2,RIMS1,RAB21,RTF1,PLCB1,COBL,WWC1,NEDD4L,ADNP,ZFPM2,HEY2,FLRT3,WWTR1,SIN3A,AUTS2,GIGYF2,FGF20,LATS2,CYFIP2,BMP10,DLL1,ITSN2,ATP8A2,SIX4,BNC2,KIF26B,SMPD3,CHD7,MBD5,WWC3,RTN4,SMURF1,SALL4,ZMIZ1,SLC4A10,SLC12A5,HEG1,SEMA6A,UNC79,WDR48,PLEKHA1,VWA1,TBL1XR1,GRHL2,WWC2,SEMA6D,NDEL1,COL21A1,ARID5B,SLITRK6,NKD1,SLITRK1,MTPN,RDH10,ARID2,AGRN |
| 7_Member | GO Biological Processes | GO:0022604 | regulation of cell morphogenesis | -25.79971835 | -23.102 | 134/484 | 25,102,387,390,659,960,998,1123,1398,1399,1600,1739,1808,1826,1857,1946,2017,2035,2043,2045,2048,2059,2185,2332,2335,2534,3037,3815,3984,4038,4076,4131,4133,4478,4627,4628,4651,4734,4803,4897,4915,5048,5063,5168,5361,5362,5530,5604,5649,5728,5789,5911,5921,5962,6091,6092,6239,6383,6722,6857,6860,7074,7101,7168,7224,7337,7430,7533,7750,7852,8766,8829,8851,9037,9202,9353,9693,9699,9706,9723,9798,9839,10109,10152,10395,10458,10507,10631,10672,10810,11141,22871,22999,23011,23072,23077,23098,23189,23327,23394,23603,25942,26039,30011,50618,55023,55435,55607,55660,55785,56990,57142,57154,57194,57381,57520,57556,57619,57688,57689,58480,65981,80031,81565,93663,113251,114798,118987,121512,140578,152503,157922,192670,254065 | ABL1,ADAM10,RHOA,RND3,BMPR2,CD44,CDC42,CHN1,CRK,CRKL,DAB1,DLG1,DPYSL2,DSCAM,DVL3,EFNA5,CTTN,EPB41,EPHA4,EPHA7,EPHB2,EPS8,PTK2B,FMR1,FN1,FYN,HAS2,KIT,LIMK1,LRP4,CAPRIN1,MAP1B,MAP2,MSN,MYH9,MYH10,MYO10,NEDD4,NGF,NRCAM,NTRK2,PAFAH1B1,PAK3,ENPP2,PLXNA1,PLXNA2,PPP3CA,MAP2K1,RELN,PTEN,PTPRD,RAP2A,RASA1,RDX,ROBO1,ROBO2,RREB1,SDC2,SRF,SYT1,SYT4,TIAM1,NR2E1,TPM1,TRPC5,UBE3A,EZR,YWHAH,ZMYM2,CXCR4,RAB11A,NRP1,CDK5R1,SEMA5A,ZMYM4,SLIT2,RAPGEF2,RIMS2,ULK2,SEMA3E,IST1,ZEB2,ARPC2,ABI2,DLC1,BAIAP2,SEMA4D,POSTN,GNA13,WASF3,IL1RAPL1,NLGN1,RIMS1,RAB21,HECW1,MYCBP2,SARM1,KANK1,NEDD4L,ADNP,CORO1C,SIN3A,SS18L1,SH3KBP1,ITSN2,PHIP,AP1AR,PPP1R9A,PRPF40A,FGD6,CDC42SE2,RTN4,SMURF1,ATP10A,RHOJ,HECW2,SEMA6A,SHROOM3,ZSWIM6,LRRC4C,RHOU,CAPRIN2,SEMA6D,NDEL1,ARHGAP18,LARP4,SLITRK1,PDZD8,FGD4,CHODL,SH3D19,CAMSAP1,AGO4,BRWD3 |
| 7_Member | GO Biological Processes | GO:0040008 | regulation of growth | -19.31284828 | -16.958 | 153/682 | 25,102,323,351,367,387,604,657,659,694,817,998,1027,1385,1399,1482,1654,1808,1826,1848,1946,1956,1982,2017,2023,2045,2066,2185,2261,2263,2335,2475,3490,3643,3720,3776,3984,4131,4133,4208,4534,4803,4851,4853,4856,4897,5048,5062,5270,5292,5515,5588,5594,5629,5727,5728,5999,6195,6197,6695,6722,6788,6857,6860,6909,6928,7042,7046,7048,7161,7224,7430,7528,7852,8089,8455,8725,8766,8829,8851,9021,9037,9211,9254,9306,9353,9472,9538,9655,9699,9706,9723,9792,9798,10276,10477,10507,10939,11221,22883,22999,23011,23236,23251,23286,23327,23394,23411,23414,23493,23677,25942,26153,26281,26524,27302,28514,28985,29967,50618,51009,51542,51761,51804,54617,55004,55031,55602,55636,55777,55841,57142,57154,57521,57556,65981,79577,79602,80014,80031,80314,81565,83452,83737,84162,84289,84312,84678,85407,123879,131566,136319,375790 | ABL1,ADAM10,APBB2,APP,AR,RHOA,BCL6,BMPR1A,BMPR2,BTG1,CAMK2D,CDC42,CDKN1B,CREB1,CRKL,NKX2-5,DDX3X,DPYSL2,DSCAM,DUSP6,EFNA5,EGFR,EIF4G2,CTTN,ENO1,EPHA7,ERBB4,PTK2B,FGFR3,FGFR2,FN1,MTOR,IGFBP7,INSR,JARID2,KCNK2,LIMK1,MAP1B,MAP2,MEF2C,MTM1,NGF,NOTCH1,NOTCH2,CCN3,NRCAM,PAFAH1B1,PAK2,SERPINE2,PIM1,PPP2CA,PRKCQ,MAPK1,PROX1,PTCH1,PTEN,RGS4,RPS6KA1,RPS6KA3,SPOCK1,SRF,STK3,SYT1,SYT4,TBX2,HNF1B,TGFB2,TGFBR1,TGFBR2,TP73,TRPC5,EZR,YY1,CXCR4,YEATS4,ATRN,URI1,RAB11A,NRP1,CDK5R1,SOCS3,SEMA5A,LGI1,CACNA2D2,SOCS6,SLIT2,AKAP6,EI24,SOCS5,RIMS2,ULK2,SEMA3E,SERTAD2,IST1,NET1,UBE2E3,SEMA4D,AFG3L2,DUSP10,CLSTN1,RIMS1,RAB21,PLCB1,MINAR1,WWC1,NEDD4L,ADNP,SIRT1,ZFPM2,HEY2,SH3BP4,SIN3A,KIF26A,FGF20,LATS2,BMP10,DLL1,MCTS1,LRP12,ITSN2,DERL2,VPS54,ATP8A2,SIX4,INO80,LAMTOR1,USP47,CDKN2AIP,CHD7,MBD5,WWC3,RTN4,SMURF1,RPTOR,SEMA6A,CAPRIN2,CDC73,ADIPOR2,WWC2,SEMA6D,EPC1,NDEL1,RAB33B,ITCH,KIAA1109,ING5,BRMS1L,KDM2B,NKD1,DCUN1D3,DCBLD2,MTPN,AGRN |
| 7_Member | GO Biological Processes | GO:0010769 | regulation of cell morphogenesis involved in differentiation | -18.22735159 | -15.906 | 87/301 | 25,102,387,659,998,1123,1398,1399,1600,1808,1826,1946,2017,2043,2045,2048,2332,2335,3037,3984,4038,4076,4131,4133,4734,4803,4897,4915,5048,5063,5361,5362,5530,5604,5649,5728,5789,5911,6091,6092,6239,6383,6722,7074,7101,7224,7337,7533,8766,8829,8851,9037,9353,9693,9706,9723,9798,9839,10109,10152,10458,10507,10631,11141,22871,23011,23072,23077,23098,23189,23327,23394,23603,25942,26039,55435,55607,57142,57520,57556,57688,57689,65981,80031,81565,114798,140578 | ABL1,ADAM10,RHOA,BMPR2,CDC42,CHN1,CRK,CRKL,DAB1,DPYSL2,DSCAM,EFNA5,CTTN,EPHA4,EPHA7,EPHB2,FMR1,FN1,HAS2,LIMK1,LRP4,CAPRIN1,MAP1B,MAP2,NEDD4,NGF,NRCAM,NTRK2,PAFAH1B1,PAK3,PLXNA1,PLXNA2,PPP3CA,MAP2K1,RELN,PTEN,PTPRD,RAP2A,ROBO1,ROBO2,RREB1,SDC2,SRF,TIAM1,NR2E1,TRPC5,UBE3A,YWHAH,RAB11A,NRP1,CDK5R1,SEMA5A,SLIT2,RAPGEF2,ULK2,SEMA3E,IST1,ZEB2,ARPC2,ABI2,BAIAP2,SEMA4D,POSTN,IL1RAPL1,NLGN1,RAB21,HECW1,MYCBP2,SARM1,KANK1,NEDD4L,ADNP,CORO1C,SIN3A,SS18L1,AP1AR,PPP1R9A,RTN4,HECW2,SEMA6A,ZSWIM6,LRRC4C,CAPRIN2,SEMA6D,NDEL1,SLITRK1,CHODL |
| 7_Member | GO Biological Processes | GO:0060560 | developmental growth involved in morphogenesis | -17.89576573 | -15.587 | 74/235 | 25,351,659,1499,1808,1826,1901,1946,2017,2045,2255,2263,2335,3688,3984,4040,4131,4133,4803,4851,4897,5048,5361,5469,6299,6722,6857,6860,6909,6928,7048,7074,7224,7852,8239,8408,8766,8828,8829,8851,8936,9037,9201,9353,9699,9706,9723,9798,9839,9863,10252,10253,10479,10507,10631,22999,23011,23242,23327,23394,23767,25942,26053,26999,50618,51804,55083,57142,57154,57556,80031,81565,85407,157506 | ABL1,APP,BMPR2,CTNNB1,DPYSL2,DSCAM,S1PR1,EFNA5,CTTN,EPHA7,FGF10,FGFR2,FN1,ITGB1,LIMK1,LRP6,MAP1B,MAP2,NGF,NOTCH1,NRCAM,PAFAH1B1,PLXNA1,MED1,SALL1,SRF,SYT1,SYT4,TBX2,HNF1B,TGFBR2,TIAM1,TRPC5,CXCR4,USP9X,ULK1,RAB11A,NRP2,NRP1,CDK5R1,WASF1,SEMA5A,DCLK1,SLIT2,RIMS2,ULK2,SEMA3E,IST1,ZEB2,MAGI2,SPRY1,SPRY2,SLC9A6,SEMA4D,POSTN,RIMS1,RAB21,COBL,NEDD4L,ADNP,FLRT3,SIN3A,AUTS2,CYFIP2,ITSN2,SIX4,KIF26B,RTN4,SMURF1,SEMA6A,SEMA6D,NDEL1,NKD1,RDH10 |
| 7_Member | GO Biological Processes | GO:0016049 | cell growth | -16.78836577 | -14.545 | 116/487 | 25,102,323,351,387,604,659,694,817,998,1027,1399,1499,1654,1808,1826,1946,1956,1982,2017,2023,2045,2185,2335,2475,2626,3490,3688,3984,4131,4133,4803,4856,4897,5048,5270,5361,5515,5588,5999,6195,6197,6416,6695,6722,6857,6860,7042,7046,7048,7074,7224,7528,7852,8239,8408,8470,8725,8766,8828,8829,8851,8936,9037,9201,9211,9353,9472,9538,9699,9706,9723,9792,9798,9839,10276,10479,10507,10631,22883,22999,23011,23242,23251,23327,23394,23411,23677,23767,25942,26053,26153,26999,27302,50618,51009,54617,55004,55031,55602,57142,57154,57521,57556,65981,79577,79602,80031,81565,83452,83737,84162,84678,123879,131566,136319 | ABL1,ADAM10,APBB2,APP,RHOA,BCL6,BMPR2,BTG1,CAMK2D,CDC42,CDKN1B,CRKL,CTNNB1,DDX3X,DPYSL2,DSCAM,EFNA5,EGFR,EIF4G2,CTTN,ENO1,EPHA7,PTK2B,FN1,MTOR,GATA4,IGFBP7,ITGB1,LIMK1,MAP1B,MAP2,NGF,CCN3,NRCAM,PAFAH1B1,SERPINE2,PLXNA1,PPP2CA,PRKCQ,RGS4,RPS6KA1,RPS6KA3,MAP2K4,SPOCK1,SRF,SYT1,SYT4,TGFB2,TGFBR1,TGFBR2,TIAM1,TRPC5,YY1,CXCR4,USP9X,ULK1,SORBS2,URI1,RAB11A,NRP2,NRP1,CDK5R1,WASF1,SEMA5A,DCLK1,LGI1,SLIT2,AKAP6,EI24,RIMS2,ULK2,SEMA3E,SERTAD2,IST1,ZEB2,NET1,SLC9A6,SEMA4D,POSTN,CLSTN1,RIMS1,RAB21,COBL,MINAR1,NEDD4L,ADNP,SIRT1,SH3BP4,FLRT3,SIN3A,AUTS2,KIF26A,CYFIP2,BMP10,ITSN2,DERL2,INO80,LAMTOR1,USP47,CDKN2AIP,RTN4,SMURF1,RPTOR,SEMA6A,CAPRIN2,CDC73,ADIPOR2,SEMA6D,NDEL1,RAB33B,ITCH,KIAA1109,KDM2B,DCUN1D3,DCBLD2,MTPN |
| 7_Member | GO Biological Processes | GO:0048638 | regulation of developmental growth | -16.04116521 | -13.846 | 91/347 | 25,351,367,657,659,1027,1385,1482,1808,1826,1848,1946,2017,2045,2066,2261,2263,2335,2475,3643,3720,3776,3984,4131,4133,4208,4534,4803,4851,4897,5048,5292,5594,5629,5727,5728,5999,6722,6788,6857,6860,6909,6928,7046,7048,7161,7224,7430,7528,7852,8455,8766,8829,8851,9037,9254,9472,9699,9706,9723,9798,10507,10939,11221,22999,23011,23236,23286,23327,23394,23414,23493,25942,26281,26524,27302,28514,50618,51761,51804,55636,55777,55841,57142,57154,57556,80014,80031,81565,85407,375790 | ABL1,APP,AR,BMPR1A,BMPR2,CDKN1B,CREB1,NKX2-5,DPYSL2,DSCAM,DUSP6,EFNA5,CTTN,EPHA7,ERBB4,FGFR3,FGFR2,FN1,MTOR,INSR,JARID2,KCNK2,LIMK1,MAP1B,MAP2,MEF2C,MTM1,NGF,NOTCH1,NRCAM,PAFAH1B1,PIM1,MAPK1,PROX1,PTCH1,PTEN,RGS4,SRF,STK3,SYT1,SYT4,TBX2,HNF1B,TGFBR1,TGFBR2,TP73,TRPC5,EZR,YY1,CXCR4,ATRN,RAB11A,NRP1,CDK5R1,SEMA5A,CACNA2D2,AKAP6,RIMS2,ULK2,SEMA3E,IST1,SEMA4D,AFG3L2,DUSP10,RIMS1,RAB21,PLCB1,WWC1,NEDD4L,ADNP,ZFPM2,HEY2,SIN3A,FGF20,LATS2,BMP10,DLL1,ITSN2,ATP8A2,SIX4,CHD7,MBD5,WWC3,RTN4,SMURF1,SEMA6A,WWC2,SEMA6D,NDEL1,NKD1,AGRN |
| 7_Member | GO Biological Processes | GO:0048588 | developmental cell growth | -12.89773994 | -10.905 | 65/234 | 25,351,659,1499,1808,1826,1946,2017,2045,2335,2475,2626,3688,3984,4131,4133,4803,4897,5048,5361,5999,6416,6722,6857,6860,7048,7074,7224,7528,7852,8239,8408,8470,8766,8828,8829,8851,8936,9037,9201,9353,9472,9699,9706,9723,9798,9839,10479,10507,10631,22999,23011,23242,23327,23394,23767,25942,26053,26999,50618,57142,57154,57556,80031,81565 | ABL1,APP,BMPR2,CTNNB1,DPYSL2,DSCAM,EFNA5,CTTN,EPHA7,FN1,MTOR,GATA4,ITGB1,LIMK1,MAP1B,MAP2,NGF,NRCAM,PAFAH1B1,PLXNA1,RGS4,MAP2K4,SRF,SYT1,SYT4,TGFBR2,TIAM1,TRPC5,YY1,CXCR4,USP9X,ULK1,SORBS2,RAB11A,NRP2,NRP1,CDK5R1,WASF1,SEMA5A,DCLK1,SLIT2,AKAP6,RIMS2,ULK2,SEMA3E,IST1,ZEB2,SLC9A6,SEMA4D,POSTN,RIMS1,RAB21,COBL,NEDD4L,ADNP,FLRT3,SIN3A,AUTS2,CYFIP2,ITSN2,RTN4,SMURF1,SEMA6A,SEMA6D,NDEL1 |
| 7_Member | GO Biological Processes | GO:0001558 | regulation of cell growth | -12.48025156 | -10.519 | 95/419 | 25,102,323,387,604,659,694,817,998,1027,1399,1654,1808,1826,1946,1956,1982,2017,2023,2045,2185,2335,2475,3490,3984,4131,4133,4803,4856,4897,5048,5270,5515,5588,5999,6195,6197,6695,6722,6857,6860,7042,7046,7224,7528,7852,8725,8766,8829,8851,9037,9211,9353,9472,9538,9699,9706,9723,9792,9798,10276,10507,22883,22999,23011,23251,23327,23394,23411,23677,25942,26153,27302,50618,51009,54617,55004,55031,55602,57142,57154,57521,57556,65981,79577,79602,80031,81565,83452,83737,84162,84678,123879,131566,136319 | ABL1,ADAM10,APBB2,RHOA,BCL6,BMPR2,BTG1,CAMK2D,CDC42,CDKN1B,CRKL,DDX3X,DPYSL2,DSCAM,EFNA5,EGFR,EIF4G2,CTTN,ENO1,EPHA7,PTK2B,FN1,MTOR,IGFBP7,LIMK1,MAP1B,MAP2,NGF,CCN3,NRCAM,PAFAH1B1,SERPINE2,PPP2CA,PRKCQ,RGS4,RPS6KA1,RPS6KA3,SPOCK1,SRF,SYT1,SYT4,TGFB2,TGFBR1,TRPC5,YY1,CXCR4,URI1,RAB11A,NRP1,CDK5R1,SEMA5A,LGI1,SLIT2,AKAP6,EI24,RIMS2,ULK2,SEMA3E,SERTAD2,IST1,NET1,SEMA4D,CLSTN1,RIMS1,RAB21,MINAR1,NEDD4L,ADNP,SIRT1,SH3BP4,SIN3A,KIF26A,BMP10,ITSN2,DERL2,INO80,LAMTOR1,USP47,CDKN2AIP,RTN4,SMURF1,RPTOR,SEMA6A,CAPRIN2,CDC73,ADIPOR2,SEMA6D,NDEL1,RAB33B,ITCH,KIAA1109,KDM2B,DCUN1D3,DCBLD2,MTPN |
| 7_Member | GO Biological Processes | GO:0045927 | positive regulation of growth | -11.75213102 | -9.844 | 69/270 | 102,387,657,659,998,1385,1654,1826,1946,1956,1982,2066,2185,2263,2335,2475,3643,3984,4131,4208,4534,4803,4851,5048,5292,5594,5629,6195,6197,6722,6857,6860,6909,7042,7046,7048,7224,7430,7852,8766,8829,9037,9211,9254,9472,9699,9798,10507,22999,23236,23327,23394,23414,23493,27302,28514,50618,51009,51761,54617,55031,55602,55636,57154,57521,81565,84678,136319,375790 | ADAM10,RHOA,BMPR1A,BMPR2,CDC42,CREB1,DDX3X,DSCAM,EFNA5,EGFR,EIF4G2,ERBB4,PTK2B,FGFR2,FN1,MTOR,INSR,LIMK1,MAP1B,MEF2C,MTM1,NGF,NOTCH1,PAFAH1B1,PIM1,MAPK1,PROX1,RPS6KA1,RPS6KA3,SRF,SYT1,SYT4,TBX2,TGFB2,TGFBR1,TGFBR2,TRPC5,EZR,CXCR4,RAB11A,NRP1,SEMA5A,LGI1,CACNA2D2,AKAP6,RIMS2,IST1,SEMA4D,RIMS1,PLCB1,NEDD4L,ADNP,ZFPM2,HEY2,BMP10,DLL1,ITSN2,DERL2,ATP8A2,INO80,USP47,CDKN2AIP,CHD7,SMURF1,RPTOR,NDEL1,KDM2B,MTPN,AGRN |
| 7_Member | GO Biological Processes | GO:0050770 | regulation of axonogenesis | -11.41362201 | -9.525 | 53/183 | 25,387,659,1123,1600,1808,1826,1946,2017,2043,2045,2048,2335,3984,4038,4131,4133,4803,4897,4915,5048,5063,5361,5362,5604,5728,6091,6092,6722,7074,7224,8766,8829,8851,9037,9353,9706,9723,9798,9839,10507,23011,23077,23394,25942,57142,57556,57688,57689,80031,81565,114798,140578 | ABL1,RHOA,BMPR2,CHN1,DAB1,DPYSL2,DSCAM,EFNA5,CTTN,EPHA4,EPHA7,EPHB2,FN1,LIMK1,LRP4,MAP1B,MAP2,NGF,NRCAM,NTRK2,PAFAH1B1,PAK3,PLXNA1,PLXNA2,MAP2K1,PTEN,ROBO1,ROBO2,SRF,TIAM1,TRPC5,RAB11A,NRP1,CDK5R1,SEMA5A,SLIT2,ULK2,SEMA3E,IST1,ZEB2,SEMA4D,RAB21,MYCBP2,ADNP,SIN3A,RTN4,SEMA6A,ZSWIM6,LRRC4C,SEMA6D,NDEL1,SLITRK1,CHODL |
| 7_Member | GO Biological Processes | GO:1990138 | neuron projection extension | -11.29145908 | -9.411 | 50/168 | 25,659,1499,1808,1826,2017,2335,3688,3984,4131,4133,4897,5048,5361,6722,6857,6860,7074,7224,7852,8239,8408,8766,8828,8829,8851,8936,9037,9201,9353,9699,9706,9723,10479,10507,10631,22999,23011,23327,23394,23767,25942,26053,26999,50618,57142,57154,57556,80031,81565 | ABL1,BMPR2,CTNNB1,DPYSL2,DSCAM,CTTN,FN1,ITGB1,LIMK1,MAP1B,MAP2,NRCAM,PAFAH1B1,PLXNA1,SRF,SYT1,SYT4,TIAM1,TRPC5,CXCR4,USP9X,ULK1,RAB11A,NRP2,NRP1,CDK5R1,WASF1,SEMA5A,DCLK1,SLIT2,RIMS2,ULK2,SEMA3E,SLC9A6,SEMA4D,POSTN,RIMS1,RAB21,NEDD4L,ADNP,FLRT3,SIN3A,AUTS2,CYFIP2,ITSN2,RTN4,SMURF1,SEMA6A,SEMA6D,NDEL1 |
| 7_Member | GO Biological Processes | GO:0010770 | positive regulation of cell morphogenesis involved in differentiation | -10.50576286 | -8.674 | 46/154 | 25,387,659,998,1398,1399,1826,1946,2043,2335,3037,3984,4076,4131,4803,4915,5048,5063,5361,5362,5604,5649,5789,6091,6092,6239,6722,7074,7224,8766,8829,9037,9353,9798,9839,10109,10458,10507,11141,23011,23394,26039,65981,81565,114798,140578 | ABL1,RHOA,BMPR2,CDC42,CRK,CRKL,DSCAM,EFNA5,EPHA4,FN1,HAS2,LIMK1,CAPRIN1,MAP1B,NGF,NTRK2,PAFAH1B1,PAK3,PLXNA1,PLXNA2,MAP2K1,RELN,PTPRD,ROBO1,ROBO2,RREB1,SRF,TIAM1,TRPC5,RAB11A,NRP1,SEMA5A,SLIT2,IST1,ZEB2,ARPC2,BAIAP2,SEMA4D,IL1RAPL1,RAB21,ADNP,SS18L1,CAPRIN2,NDEL1,SLITRK1,CHODL |
| 7_Member | GO Biological Processes | GO:0008361 | regulation of cell size | -10.08704724 | -8.281 | 50/180 | 25,118,361,387,659,1182,1385,1808,1826,1874,1946,2017,2045,2335,2475,3984,4131,4133,4478,4803,4897,5048,5728,5962,6558,6722,7224,7248,7430,8766,8829,8851,9037,9706,9723,9798,9821,10000,10507,23011,23108,23394,25942,57142,57468,57521,57556,80031,81565,136319 | ABL1,ADD1,AQP4,RHOA,BMPR2,CLCN3,CREB1,DPYSL2,DSCAM,E2F4,EFNA5,CTTN,EPHA7,FN1,MTOR,LIMK1,MAP1B,MAP2,MSN,NGF,NRCAM,PAFAH1B1,PTEN,RDX,SLC12A2,SRF,TRPC5,TSC1,EZR,RAB11A,NRP1,CDK5R1,SEMA5A,ULK2,SEMA3E,IST1,RB1CC1,AKT3,SEMA4D,RAB21,RAP1GAP2,ADNP,SIN3A,RTN4,SLC12A5,RPTOR,SEMA6A,SEMA6D,NDEL1,MTPN |
| 7_Member | GO Biological Processes | GO:0048639 | positive regulation of developmental growth | -9.716128931 | -7.941 | 50/184 | 657,659,1385,1826,1946,2066,2263,2335,2475,3643,3984,4131,4208,4534,4803,4851,5048,5292,5594,5629,6722,6857,6860,6909,7048,7224,7430,7852,8766,8829,9037,9254,9472,9699,9798,10507,22999,23236,23327,23394,23414,23493,27302,28514,50618,51761,55636,57154,81565,375790 | BMPR1A,BMPR2,CREB1,DSCAM,EFNA5,ERBB4,FGFR2,FN1,MTOR,INSR,LIMK1,MAP1B,MEF2C,MTM1,NGF,NOTCH1,PAFAH1B1,PIM1,MAPK1,PROX1,SRF,SYT1,SYT4,TBX2,TGFBR2,TRPC5,EZR,CXCR4,RAB11A,NRP1,SEMA5A,CACNA2D2,AKAP6,RIMS2,IST1,SEMA4D,RIMS1,PLCB1,NEDD4L,ADNP,ZFPM2,HEY2,BMP10,DLL1,ITSN2,ATP8A2,CHD7,SMURF1,NDEL1,AGRN |
| 7_Member | GO Biological Processes | GO:0030307 | positive regulation of cell growth | -9.021835214 | -7.304 | 46/169 | 102,387,659,998,1654,1826,1946,1956,1982,2185,2335,2475,3984,4131,4803,5048,6195,6197,6722,6857,6860,7042,7046,7224,7852,8766,8829,9037,9211,9472,9699,9798,10507,22999,23327,23394,50618,51009,54617,55031,55602,57154,57521,81565,84678,136319 | ADAM10,RHOA,BMPR2,CDC42,DDX3X,DSCAM,EFNA5,EGFR,EIF4G2,PTK2B,FN1,MTOR,LIMK1,MAP1B,NGF,PAFAH1B1,RPS6KA1,RPS6KA3,SRF,SYT1,SYT4,TGFB2,TGFBR1,TRPC5,CXCR4,RAB11A,NRP1,SEMA5A,LGI1,AKAP6,RIMS2,IST1,SEMA4D,RIMS1,NEDD4L,ADNP,ITSN2,DERL2,INO80,USP47,CDKN2AIP,SMURF1,RPTOR,NDEL1,KDM2B,MTPN |
| 7_Member | GO Biological Processes | GO:0050772 | positive regulation of axonogenesis | -8.333619868 | -6.680 | 29/85 | 387,659,1826,1946,2335,3984,4131,4803,4915,5048,5361,5362,5604,6091,6092,6722,7074,7224,8766,8829,9037,9353,9798,9839,10507,23394,81565,114798,140578 | RHOA,BMPR2,DSCAM,EFNA5,FN1,LIMK1,MAP1B,NGF,NTRK2,PAFAH1B1,PLXNA1,PLXNA2,MAP2K1,ROBO1,ROBO2,SRF,TIAM1,TRPC5,RAB11A,NRP1,SEMA5A,SLIT2,IST1,ZEB2,SEMA4D,ADNP,NDEL1,SLITRK1,CHODL |
| 7_Member | GO Biological Processes | GO:0048675 | axon extension | -7.764540484 | -6.160 | 35/121 | 25,659,1808,1826,2017,2335,3688,3984,4131,4133,4897,5048,6722,7224,8239,8408,8766,8828,8829,8851,9037,9201,9353,9706,9723,10479,10507,23011,23394,25942,26053,57142,57556,80031,81565 | ABL1,BMPR2,DPYSL2,DSCAM,CTTN,FN1,ITGB1,LIMK1,MAP1B,MAP2,NRCAM,PAFAH1B1,SRF,TRPC5,USP9X,ULK1,RAB11A,NRP2,NRP1,CDK5R1,SEMA5A,DCLK1,SLIT2,ULK2,SEMA3E,SLC9A6,SEMA4D,RAB21,ADNP,SIN3A,AUTS2,RTN4,SEMA6A,SEMA6D,NDEL1 |
| 7_Member | GO Biological Processes | GO:0061387 | regulation of extent of cell growth | -6.675178836 | -5.173 | 31/110 | 25,659,1808,1826,1946,2017,2045,2335,3984,4131,4133,4803,4897,5048,6722,7224,8766,8829,8851,9037,9706,9723,9798,10507,23011,23394,25942,57142,57556,80031,81565 | ABL1,BMPR2,DPYSL2,DSCAM,EFNA5,CTTN,EPHA7,FN1,LIMK1,MAP1B,MAP2,NGF,NRCAM,PAFAH1B1,SRF,TRPC5,RAB11A,NRP1,CDK5R1,SEMA5A,ULK2,SEMA3E,IST1,SEMA4D,RAB21,ADNP,SIN3A,RTN4,SEMA6A,SEMA6D,NDEL1 |
| 7_Member | GO Biological Processes | GO:0030516 | regulation of axon extension | -5.354769055 | -4.005 | 26/96 | 25,659,1808,1826,2017,2335,3984,4131,4133,4897,5048,6722,7224,8766,8829,8851,9037,9723,10507,23011,23394,25942,57142,57556,80031,81565 | ABL1,BMPR2,DPYSL2,DSCAM,CTTN,FN1,LIMK1,MAP1B,MAP2,NRCAM,PAFAH1B1,SRF,TRPC5,RAB11A,NRP1,CDK5R1,SEMA5A,SEMA3E,SEMA4D,RAB21,ADNP,SIN3A,RTN4,SEMA6A,SEMA6D,NDEL1 |
| 7_Member | GO Biological Processes | GO:0045773 | positive regulation of axon extension | -3.478559029 | -2.352 | 13/43 | 659,1826,2335,3984,4131,5048,6722,7224,8766,8829,9037,23394,81565 | BMPR2,DSCAM,FN1,LIMK1,MAP1B,PAFAH1B1,SRF,TRPC5,RAB11A,NRP1,SEMA5A,ADNP,NDEL1 |
| 8_Summary | GO Cellular Components | GO:0030425 | dendrite | -29.01827674 | -26.083 | 164/620 | 25,102,108,288,320,321,351,387,490,547,657,659,664,775,793,998,1393,1501,1812,1826,2017,2043,2044,2045,2048,2185,2332,2475,2534,2554,2557,2558,2565,2743,2781,2782,2823,2891,2895,2898,2903,2911,2917,3064,3192,3358,3643,3688,3736,3737,3739,3745,3746,3747,3756,3759,3781,3800,4038,4076,4131,4133,4139,4140,4628,4734,4763,4803,4856,4905,4915,4986,5100,5126,5142,5173,5332,5368,5501,5530,5563,5577,5594,5599,5649,5727,5728,5796,5813,5868,6416,6421,6453,6546,6860,7074,7224,7881,8001,8087,8633,8678,8725,8851,8898,8910,8936,9455,9456,9743,9863,10152,10160,10236,10243,10458,10479,10564,10636,10752,10891,11141,22849,22871,22883,22941,23034,23098,23114,23162,23208,23242,23373,23394,23613,23774,25828,26058,26090,26278,27020,29966,50807,51218,54407,54464,55607,56899,57120,57282,57521,58489,60481,60482,64506,79739,84945,91752,94056,342371,348980,388336,392617,729993,1363,1515,1600,2182,3267,3776,4040,4651,4762,5048,5152,5170,5274,5664,5792,6323,6654,7042,7150,7432,8301,8829,9378,9590,9627,9693,9860,10146,10846,22866,23001,23544,55737,57142,57154,57565,58155,81565,140767,143187,146713,5783,7430,83394,135138,8411 | ABL1,ADAM10,ADCY2,ANK3,APBA1,APBA2,APP,RHOA,ATP2B1,KIF1A,BMPR1A,BMPR2,BNIP3,CACNA1C,CALB1,CDC42,CRHBP,CTNND2,DRD1,DSCAM,CTTN,EPHA4,EPHA5,EPHA7,EPHB2,PTK2B,FMR1,MTOR,FYN,GABRA1,GABRA4,GABRA5,GABRG1,GLRB,GNAZ,GNB1,GPM6A,GRIA2,GRID2,GRIK2,GRIN2A,GRM1,GRM7,HTT,HNRNPU,HTR2C,INSR,ITGB1,KCNA1,KCNA2,KCNA4,KCNB1,KCNC1,KCNC2,KCNH1,KCNJ2,KCNN2,KIF5C,LRP4,CAPRIN1,MAP1B,MAP2,MARK1,MARK3,MYH10,NEDD4,NF1,NGF,CCN3,NSF,NTRK2,OPRK1,PCDH8,PCSK2,PDE4B,PDYN,PLCB4,PNOC,PPP1CC,PPP3CA,PRKAA2,PRKAR2B,MAPK1,MAPK8,RELN,PTCH1,PTEN,PTPRK,PURA,RAB5A,MAP2K4,SFPQ,ITSN1,SLC8A1,SYT4,TIAM1,TRPC5,KCNAB1,GLRA3,FXR1,UNC5C,BECN1,URI1,CDK5R1,MTMR2,SGCE,WASF1,HOMER2,HOMER1,ARHGAP32,MAGI2,ABI2,FARP1,HNRNPR,GPHN,BAIAP2,SLC9A6,ARFGEF2,RGS14,CHL1,PPARGC1A,IL1RAPL1,CPEB3,NLGN1,CLSTN1,SHANK2,SAMD4A,SARM1,NFASC,MAPK8IP3,SYT11,COBL,CRTC1,ADNP,ZMYND8,BRD1,TXN2,GIGYF2,ABHD12,SACS,NPTN,STRN3,ASAP1,GLRX5,SLC38A2,XRN1,PPP1R9A,ANKS1B,GOPC,SLC4A10,RPTOR,ABHD17C,ELOVL5,SLC5A7,CPEB1,TTLL7,ABHD13,ZNF804A,SYAP1,ATXN1L,HCN1,SHISA6,ELFN1,SHISA9,CPE,CTSV,DAB1,ACSL4,AGFG1,KCNK2,LRP6,MYO10,NEUROG1,PAFAH1B1,PDE9A,PDPK1,SERPINI1,PSEN2,PTPRF,SCN1A,SOS1,TGFB2,TOP1,VIP,PICALM,NRP1,NRXN1,AKAP12,SNCAIP,RAPGEF2,LRIG2,G3BP1,PDE10A,CNKSR2,WDFY3,SEZ6L,VPS35,RTN4,SMURF1,KLHL14,PTBP2,NDEL1,NRSN1,VTI1A,RBFOX3,PTPN13,EZR,PITPNM3,PACRG,EEA1 |
| 8_Member | GO Cellular Components | GO:0030425 | dendrite | -29.01827674 | -26.083 | 164/620 | 25,102,108,288,320,321,351,387,490,547,657,659,664,775,793,998,1393,1501,1812,1826,2017,2043,2044,2045,2048,2185,2332,2475,2534,2554,2557,2558,2565,2743,2781,2782,2823,2891,2895,2898,2903,2911,2917,3064,3192,3358,3643,3688,3736,3737,3739,3745,3746,3747,3756,3759,3781,3800,4038,4076,4131,4133,4139,4140,4628,4734,4763,4803,4856,4905,4915,4986,5100,5126,5142,5173,5332,5368,5501,5530,5563,5577,5594,5599,5649,5727,5728,5796,5813,5868,6416,6421,6453,6546,6860,7074,7224,7881,8001,8087,8633,8678,8725,8851,8898,8910,8936,9455,9456,9743,9863,10152,10160,10236,10243,10458,10479,10564,10636,10752,10891,11141,22849,22871,22883,22941,23034,23098,23114,23162,23208,23242,23373,23394,23613,23774,25828,26058,26090,26278,27020,29966,50807,51218,54407,54464,55607,56899,57120,57282,57521,58489,60481,60482,64506,79739,84945,91752,94056,342371,348980,388336,392617,729993 | ABL1,ADAM10,ADCY2,ANK3,APBA1,APBA2,APP,RHOA,ATP2B1,KIF1A,BMPR1A,BMPR2,BNIP3,CACNA1C,CALB1,CDC42,CRHBP,CTNND2,DRD1,DSCAM,CTTN,EPHA4,EPHA5,EPHA7,EPHB2,PTK2B,FMR1,MTOR,FYN,GABRA1,GABRA4,GABRA5,GABRG1,GLRB,GNAZ,GNB1,GPM6A,GRIA2,GRID2,GRIK2,GRIN2A,GRM1,GRM7,HTT,HNRNPU,HTR2C,INSR,ITGB1,KCNA1,KCNA2,KCNA4,KCNB1,KCNC1,KCNC2,KCNH1,KCNJ2,KCNN2,KIF5C,LRP4,CAPRIN1,MAP1B,MAP2,MARK1,MARK3,MYH10,NEDD4,NF1,NGF,CCN3,NSF,NTRK2,OPRK1,PCDH8,PCSK2,PDE4B,PDYN,PLCB4,PNOC,PPP1CC,PPP3CA,PRKAA2,PRKAR2B,MAPK1,MAPK8,RELN,PTCH1,PTEN,PTPRK,PURA,RAB5A,MAP2K4,SFPQ,ITSN1,SLC8A1,SYT4,TIAM1,TRPC5,KCNAB1,GLRA3,FXR1,UNC5C,BECN1,URI1,CDK5R1,MTMR2,SGCE,WASF1,HOMER2,HOMER1,ARHGAP32,MAGI2,ABI2,FARP1,HNRNPR,GPHN,BAIAP2,SLC9A6,ARFGEF2,RGS14,CHL1,PPARGC1A,IL1RAPL1,CPEB3,NLGN1,CLSTN1,SHANK2,SAMD4A,SARM1,NFASC,MAPK8IP3,SYT11,COBL,CRTC1,ADNP,ZMYND8,BRD1,TXN2,GIGYF2,ABHD12,SACS,NPTN,STRN3,ASAP1,GLRX5,SLC38A2,XRN1,PPP1R9A,ANKS1B,GOPC,SLC4A10,RPTOR,ABHD17C,ELOVL5,SLC5A7,CPEB1,TTLL7,ABHD13,ZNF804A,SYAP1,ATXN1L,HCN1,SHISA6,ELFN1,SHISA9 |
| 8_Member | GO Cellular Components | GO:0097447 | dendritic tree | -28.84391088 | -25.940 | 164/622 | 25,102,108,288,320,321,351,387,490,547,657,659,664,775,793,998,1393,1501,1812,1826,2017,2043,2044,2045,2048,2185,2332,2475,2534,2554,2557,2558,2565,2743,2781,2782,2823,2891,2895,2898,2903,2911,2917,3064,3192,3358,3643,3688,3736,3737,3739,3745,3746,3747,3756,3759,3781,3800,4038,4076,4131,4133,4139,4140,4628,4734,4763,4803,4856,4905,4915,4986,5100,5126,5142,5173,5332,5368,5501,5530,5563,5577,5594,5599,5649,5727,5728,5796,5813,5868,6416,6421,6453,6546,6860,7074,7224,7881,8001,8087,8633,8678,8725,8851,8898,8910,8936,9455,9456,9743,9863,10152,10160,10236,10243,10458,10479,10564,10636,10752,10891,11141,22849,22871,22883,22941,23034,23098,23114,23162,23208,23242,23373,23394,23613,23774,25828,26058,26090,26278,27020,29966,50807,51218,54407,54464,55607,56899,57120,57282,57521,58489,60481,60482,64506,79739,84945,91752,94056,342371,348980,388336,392617,729993 | ABL1,ADAM10,ADCY2,ANK3,APBA1,APBA2,APP,RHOA,ATP2B1,KIF1A,BMPR1A,BMPR2,BNIP3,CACNA1C,CALB1,CDC42,CRHBP,CTNND2,DRD1,DSCAM,CTTN,EPHA4,EPHA5,EPHA7,EPHB2,PTK2B,FMR1,MTOR,FYN,GABRA1,GABRA4,GABRA5,GABRG1,GLRB,GNAZ,GNB1,GPM6A,GRIA2,GRID2,GRIK2,GRIN2A,GRM1,GRM7,HTT,HNRNPU,HTR2C,INSR,ITGB1,KCNA1,KCNA2,KCNA4,KCNB1,KCNC1,KCNC2,KCNH1,KCNJ2,KCNN2,KIF5C,LRP4,CAPRIN1,MAP1B,MAP2,MARK1,MARK3,MYH10,NEDD4,NF1,NGF,CCN3,NSF,NTRK2,OPRK1,PCDH8,PCSK2,PDE4B,PDYN,PLCB4,PNOC,PPP1CC,PPP3CA,PRKAA2,PRKAR2B,MAPK1,MAPK8,RELN,PTCH1,PTEN,PTPRK,PURA,RAB5A,MAP2K4,SFPQ,ITSN1,SLC8A1,SYT4,TIAM1,TRPC5,KCNAB1,GLRA3,FXR1,UNC5C,BECN1,URI1,CDK5R1,MTMR2,SGCE,WASF1,HOMER2,HOMER1,ARHGAP32,MAGI2,ABI2,FARP1,HNRNPR,GPHN,BAIAP2,SLC9A6,ARFGEF2,RGS14,CHL1,PPARGC1A,IL1RAPL1,CPEB3,NLGN1,CLSTN1,SHANK2,SAMD4A,SARM1,NFASC,MAPK8IP3,SYT11,COBL,CRTC1,ADNP,ZMYND8,BRD1,TXN2,GIGYF2,ABHD12,SACS,NPTN,STRN3,ASAP1,GLRX5,SLC38A2,XRN1,PPP1R9A,ANKS1B,GOPC,SLC4A10,RPTOR,ABHD17C,ELOVL5,SLC5A7,CPEB1,TTLL7,ABHD13,ZNF804A,SYAP1,ATXN1L,HCN1,SHISA6,ELFN1,SHISA9 |
| 8_Member | GO Cellular Components | GO:0043025 | neuronal cell body | -18.29418834 | -15.969 | 121/497 | 25,102,351,490,547,657,659,775,793,998,1363,1393,1501,1515,1600,1826,2043,2044,2045,2048,2182,2185,2332,2475,2558,2823,2898,3267,3643,3736,3737,3745,3746,3747,3756,3759,3776,3781,3800,4038,4040,4131,4133,4628,4651,4762,4856,4986,5048,5126,5152,5170,5173,5274,5368,5563,5577,5594,5664,5792,5796,5813,5868,6323,6416,6453,6654,6860,7042,7074,7150,7224,7432,7881,8001,8087,8301,8633,8829,8851,9378,9455,9590,9627,9693,9860,10146,10458,10846,10891,22866,22941,23001,23242,23373,23394,23544,23774,25828,26058,29966,51218,54407,54464,55607,55737,57142,57154,57282,57521,57565,58155,60481,60482,79739,81565,91752,94056,140767,143187,146713 | ABL1,ADAM10,APP,ATP2B1,KIF1A,BMPR1A,BMPR2,CACNA1C,CALB1,CDC42,CPE,CRHBP,CTNND2,CTSV,DAB1,DSCAM,EPHA4,EPHA5,EPHA7,EPHB2,ACSL4,PTK2B,FMR1,MTOR,GABRA5,GPM6A,GRIK2,AGFG1,INSR,KCNA1,KCNA2,KCNB1,KCNC1,KCNC2,KCNH1,KCNJ2,KCNK2,KCNN2,KIF5C,LRP4,LRP6,MAP1B,MAP2,MYH10,MYO10,NEUROG1,CCN3,OPRK1,PAFAH1B1,PCSK2,PDE9A,PDPK1,PDYN,SERPINI1,PNOC,PRKAA2,PRKAR2B,MAPK1,PSEN2,PTPRF,PTPRK,PURA,RAB5A,SCN1A,MAP2K4,ITSN1,SOS1,SYT4,TGFB2,TIAM1,TOP1,TRPC5,VIP,KCNAB1,GLRA3,FXR1,PICALM,UNC5C,NRP1,CDK5R1,NRXN1,HOMER2,AKAP12,SNCAIP,RAPGEF2,LRIG2,G3BP1,BAIAP2,PDE10A,PPARGC1A,CNKSR2,SHANK2,WDFY3,COBL,CRTC1,ADNP,SEZ6L,BRD1,TXN2,GIGYF2,STRN3,GLRX5,SLC38A2,XRN1,PPP1R9A,VPS35,RTN4,SMURF1,SLC4A10,RPTOR,KLHL14,PTBP2,ELOVL5,SLC5A7,TTLL7,NDEL1,ZNF804A,SYAP1,NRSN1,VTI1A,RBFOX3 |
| 8_Member | GO Cellular Components | GO:0044297 | cell body | -17.4870917 | -15.197 | 130/564 | 25,102,351,490,547,657,659,775,793,998,1363,1393,1501,1515,1600,1826,2043,2044,2045,2048,2182,2185,2332,2475,2534,2558,2781,2782,2823,2898,3267,3643,3736,3737,3745,3746,3747,3756,3759,3776,3781,3800,4038,4040,4131,4133,4628,4651,4762,4856,4986,5048,5126,5152,5170,5173,5274,5368,5563,5577,5594,5664,5783,5792,5796,5813,5868,6323,6416,6453,6654,6860,7042,7074,7150,7224,7430,7432,7881,8001,8087,8301,8633,8829,8851,9378,9455,9590,9627,9693,9860,10146,10458,10846,10891,22866,22941,23001,23162,23208,23242,23373,23394,23544,23774,25828,26058,29966,51218,54407,54464,55607,55737,57142,57154,57282,57521,57565,58155,60481,60482,79739,81565,83394,91752,94056,135138,140767,143187,146713 | ABL1,ADAM10,APP,ATP2B1,KIF1A,BMPR1A,BMPR2,CACNA1C,CALB1,CDC42,CPE,CRHBP,CTNND2,CTSV,DAB1,DSCAM,EPHA4,EPHA5,EPHA7,EPHB2,ACSL4,PTK2B,FMR1,MTOR,FYN,GABRA5,GNAZ,GNB1,GPM6A,GRIK2,AGFG1,INSR,KCNA1,KCNA2,KCNB1,KCNC1,KCNC2,KCNH1,KCNJ2,KCNK2,KCNN2,KIF5C,LRP4,LRP6,MAP1B,MAP2,MYH10,MYO10,NEUROG1,CCN3,OPRK1,PAFAH1B1,PCSK2,PDE9A,PDPK1,PDYN,SERPINI1,PNOC,PRKAA2,PRKAR2B,MAPK1,PSEN2,PTPN13,PTPRF,PTPRK,PURA,RAB5A,SCN1A,MAP2K4,ITSN1,SOS1,SYT4,TGFB2,TIAM1,TOP1,TRPC5,EZR,VIP,KCNAB1,GLRA3,FXR1,PICALM,UNC5C,NRP1,CDK5R1,NRXN1,HOMER2,AKAP12,SNCAIP,RAPGEF2,LRIG2,G3BP1,BAIAP2,PDE10A,PPARGC1A,CNKSR2,SHANK2,WDFY3,MAPK8IP3,SYT11,COBL,CRTC1,ADNP,SEZ6L,BRD1,TXN2,GIGYF2,STRN3,GLRX5,SLC38A2,XRN1,PPP1R9A,VPS35,RTN4,SMURF1,SLC4A10,RPTOR,KLHL14,PTBP2,ELOVL5,SLC5A7,TTLL7,NDEL1,PITPNM3,ZNF804A,SYAP1,PACRG,NRSN1,VTI1A,RBFOX3 |
| 8_Member | GO Cellular Components | GO:0044309 | neuron spine | -15.17733196 | -13.042 | 57/171 | 102,320,321,351,387,490,793,998,1812,2017,2043,2185,2332,2823,2891,2895,2903,3688,3739,3759,3781,4131,4628,4734,4915,5142,5501,5530,5577,5728,6453,6546,7074,8087,8411,8851,8898,9456,9743,10152,10160,10458,10479,10564,10636,22871,22883,22941,23208,23613,50807,55607,56899,58489,91752,388336,729993 | ADAM10,APBA1,APBA2,APP,RHOA,ATP2B1,CALB1,CDC42,DRD1,CTTN,EPHA4,PTK2B,FMR1,GPM6A,GRIA2,GRID2,GRIN2A,ITGB1,KCNA4,KCNJ2,KCNN2,MAP1B,MYH10,NEDD4,NTRK2,PDE4B,PPP1CC,PPP3CA,PRKAR2B,PTEN,ITSN1,SLC8A1,TIAM1,FXR1,EEA1,CDK5R1,MTMR2,HOMER1,ARHGAP32,ABI2,FARP1,BAIAP2,SLC9A6,ARFGEF2,RGS14,NLGN1,CLSTN1,SHANK2,SYT11,ZMYND8,ASAP1,PPP1R9A,ANKS1B,ABHD17C,ZNF804A,SHISA6,SHISA9 |
| 8_Member | GO Cellular Components | GO:0043197 | dendritic spine | -14.16410943 | -12.097 | 55/169 | 102,320,321,351,387,490,793,998,1812,2017,2043,2185,2332,2823,2891,2895,2903,3688,3739,3759,3781,4131,4628,4734,4915,5142,5501,5530,5577,5728,6453,6546,7074,8087,8851,8898,9456,9743,10152,10160,10458,10564,10636,22871,22883,22941,23208,23613,50807,55607,56899,58489,91752,388336,729993 | ADAM10,APBA1,APBA2,APP,RHOA,ATP2B1,CALB1,CDC42,DRD1,CTTN,EPHA4,PTK2B,FMR1,GPM6A,GRIA2,GRID2,GRIN2A,ITGB1,KCNA4,KCNJ2,KCNN2,MAP1B,MYH10,NEDD4,NTRK2,PDE4B,PPP1CC,PPP3CA,PRKAR2B,PTEN,ITSN1,SLC8A1,TIAM1,FXR1,CDK5R1,MTMR2,HOMER1,ARHGAP32,ABI2,FARP1,BAIAP2,ARFGEF2,RGS14,NLGN1,CLSTN1,SHANK2,SYT11,ZMYND8,ASAP1,PPP1R9A,ANKS1B,ABHD17C,ZNF804A,SHISA6,SHISA9 |
| 9_Summary | GO Biological Processes | GO:0050808 | synapse organization | -28.57485006 | -25.687 | 125/408 | 25,60,102,288,351,375,387,783,998,1006,1007,1282,1287,1399,1499,1501,1607,1739,1740,1812,1826,1946,2017,2043,2045,2048,2066,2119,2332,2534,2554,2668,2743,2823,2895,2904,2915,3084,3643,4038,4076,4131,4208,4628,4734,4774,4897,4915,5048,5063,5100,5649,5728,5789,5792,5978,6092,6453,7074,7248,7337,7402,7534,8321,8499,8828,8829,8851,8898,8936,8976,9231,9254,9378,9456,9723,9863,10152,10160,10243,10397,10458,10479,10497,10507,10810,10939,11141,22854,22866,22871,22883,22941,23114,23394,23544,23613,23767,23768,26045,27020,27253,27445,51804,53942,55607,55638,55737,55906,57633,57689,58489,65981,84189,84631,91752,114798,139065,158038,221935,347731,375790,388336,392617,440279,57084 | ABL1,ACTB,ADAM10,ANK3,APP,ARF1,RHOA,CACNB2,CDC42,CDH8,CDH9,COL4A1,COL4A5,CRKL,CTNNB1,CTNND2,DGKB,DLG1,DLG2,DRD1,DSCAM,EFNA5,CTTN,EPHA4,EPHA7,EPHB2,ERBB4,ETV5,FMR1,FYN,GABRA1,GDNF,GLRB,GPM6A,GRID2,GRIN2B,GRM5,NRG1,INSR,LRP4,CAPRIN1,MAP1B,MEF2C,MYH10,NEDD4,NFIA,NRCAM,NTRK2,PAFAH1B1,PAK3,PCDH8,RELN,PTEN,PTPRD,PTPRF,REST,ROBO2,ITSN1,TIAM1,TSC1,UBE3A,UTRN,YWHAZ,FZD1,PPFIA2,NRP2,NRP1,CDK5R1,MTMR2,WASF1,WASL,DLG5,CACNA2D2,NRXN1,HOMER1,SEMA3E,MAGI2,ABI2,FARP1,GPHN,NDRG1,BAIAP2,SLC9A6,UNC13B,SEMA4D,WASF3,AFG3L2,IL1RAPL1,NTNG1,CNKSR2,NLGN1,CLSTN1,SHANK2,NFASC,ADNP,SEZ6L,ZMYND8,FLRT3,FLRT2,LRRTM2,NPTN,PCDH17,PCLO,SIX4,CNTN5,PPP1R9A,SYBU,VPS35,ZC4H2,LRRN1,LRRC4C,ABHD17C,CAPRIN2,SLITRK6,SLITRK2,ZNF804A,SLITRK1,SLITRK4,LINGO2,SDK1,LRRTM3,AGRN,SHISA6,ELFN1,UNC13C,SLC17A6 |
| 9_Member | GO Biological Processes | GO:0050808 | synapse organization | -28.57485006 | -25.687 | 125/408 | 25,60,102,288,351,375,387,783,998,1006,1007,1282,1287,1399,1499,1501,1607,1739,1740,1812,1826,1946,2017,2043,2045,2048,2066,2119,2332,2534,2554,2668,2743,2823,2895,2904,2915,3084,3643,4038,4076,4131,4208,4628,4734,4774,4897,4915,5048,5063,5100,5649,5728,5789,5792,5978,6092,6453,7074,7248,7337,7402,7534,8321,8499,8828,8829,8851,8898,8936,8976,9231,9254,9378,9456,9723,9863,10152,10160,10243,10397,10458,10479,10497,10507,10810,10939,11141,22854,22866,22871,22883,22941,23114,23394,23544,23613,23767,23768,26045,27020,27253,27445,51804,53942,55607,55638,55737,55906,57633,57689,58489,65981,84189,84631,91752,114798,139065,158038,221935,347731,375790,388336,392617,440279 | ABL1,ACTB,ADAM10,ANK3,APP,ARF1,RHOA,CACNB2,CDC42,CDH8,CDH9,COL4A1,COL4A5,CRKL,CTNNB1,CTNND2,DGKB,DLG1,DLG2,DRD1,DSCAM,EFNA5,CTTN,EPHA4,EPHA7,EPHB2,ERBB4,ETV5,FMR1,FYN,GABRA1,GDNF,GLRB,GPM6A,GRID2,GRIN2B,GRM5,NRG1,INSR,LRP4,CAPRIN1,MAP1B,MEF2C,MYH10,NEDD4,NFIA,NRCAM,NTRK2,PAFAH1B1,PAK3,PCDH8,RELN,PTEN,PTPRD,PTPRF,REST,ROBO2,ITSN1,TIAM1,TSC1,UBE3A,UTRN,YWHAZ,FZD1,PPFIA2,NRP2,NRP1,CDK5R1,MTMR2,WASF1,WASL,DLG5,CACNA2D2,NRXN1,HOMER1,SEMA3E,MAGI2,ABI2,FARP1,GPHN,NDRG1,BAIAP2,SLC9A6,UNC13B,SEMA4D,WASF3,AFG3L2,IL1RAPL1,NTNG1,CNKSR2,NLGN1,CLSTN1,SHANK2,NFASC,ADNP,SEZ6L,ZMYND8,FLRT3,FLRT2,LRRTM2,NPTN,PCDH17,PCLO,SIX4,CNTN5,PPP1R9A,SYBU,VPS35,ZC4H2,LRRN1,LRRC4C,ABHD17C,CAPRIN2,SLITRK6,SLITRK2,ZNF804A,SLITRK1,SLITRK4,LINGO2,SDK1,LRRTM3,AGRN,SHISA6,ELFN1,UNC13C |
| 9_Member | GO Biological Processes | GO:0050803 | regulation of synapse structure or activity | -15.25729581 | -13.105 | 68/227 | 25,102,351,387,1006,1399,1607,1946,2043,2045,2048,2119,2332,2534,2823,2895,2904,4038,4076,4208,4734,4897,4915,5048,5063,5100,5649,5728,5789,6092,6453,7074,7337,7534,8321,8828,8851,9231,9378,9456,10152,10458,10507,11141,22871,22883,23394,23613,23767,23768,26045,51804,55607,55638,55737,57084,57633,58489,65981,84189,84631,91752,114798,139065,158038,347731,375790,388336 | ABL1,ADAM10,APP,RHOA,CDH8,CRKL,DGKB,EFNA5,EPHA4,EPHA7,EPHB2,ETV5,FMR1,FYN,GPM6A,GRID2,GRIN2B,LRP4,CAPRIN1,MEF2C,NEDD4,NRCAM,NTRK2,PAFAH1B1,PAK3,PCDH8,RELN,PTEN,PTPRD,ROBO2,ITSN1,TIAM1,UBE3A,YWHAZ,FZD1,NRP2,CDK5R1,DLG5,NRXN1,HOMER1,ABI2,BAIAP2,SEMA4D,IL1RAPL1,NLGN1,CLSTN1,ADNP,ZMYND8,FLRT3,FLRT2,LRRTM2,SIX4,PPP1R9A,SYBU,VPS35,SLC17A6,LRRN1,ABHD17C,CAPRIN2,SLITRK6,SLITRK2,ZNF804A,SLITRK1,SLITRK4,LINGO2,LRRTM3,AGRN,SHISA6 |
| 9_Member | GO Biological Processes | GO:0050807 | regulation of synapse organization | -15.08181254 | -12.951 | 66/218 | 25,102,351,387,1006,1399,1607,1946,2043,2045,2048,2119,2332,2534,2823,2895,2904,4038,4076,4208,4734,4897,4915,5048,5063,5100,5649,5728,5789,6092,6453,7074,7337,7534,8321,8828,8851,9231,9378,9456,10152,10458,10507,11141,22871,22883,23394,23613,23767,23768,26045,51804,55607,55737,57633,58489,65981,84189,84631,91752,114798,139065,158038,347731,375790,388336 | ABL1,ADAM10,APP,RHOA,CDH8,CRKL,DGKB,EFNA5,EPHA4,EPHA7,EPHB2,ETV5,FMR1,FYN,GPM6A,GRID2,GRIN2B,LRP4,CAPRIN1,MEF2C,NEDD4,NRCAM,NTRK2,PAFAH1B1,PAK3,PCDH8,RELN,PTEN,PTPRD,ROBO2,ITSN1,TIAM1,UBE3A,YWHAZ,FZD1,NRP2,CDK5R1,DLG5,NRXN1,HOMER1,ABI2,BAIAP2,SEMA4D,IL1RAPL1,NLGN1,CLSTN1,ADNP,ZMYND8,FLRT3,FLRT2,LRRTM2,SIX4,PPP1R9A,VPS35,LRRN1,ABHD17C,CAPRIN2,SLITRK6,SLITRK2,ZNF804A,SLITRK1,SLITRK4,LINGO2,LRRTM3,AGRN,SHISA6 |
| 9_Member | GO Biological Processes | GO:0007416 | synapse assembly | -10.91851431 | -9.052 | 51/177 | 351,1007,1399,1812,1826,1946,2045,2048,2066,2554,2823,2895,3084,4038,4131,4208,4897,4915,5728,5789,6092,8321,9231,9378,9863,10160,10507,11141,22871,22883,22941,23394,23767,23768,26045,27020,27253,27445,51804,53942,55607,55737,57633,84189,84631,114798,139065,158038,221935,347731,375790 | APP,CDH9,CRKL,DRD1,DSCAM,EFNA5,EPHA7,EPHB2,ERBB4,GABRA1,GPM6A,GRID2,NRG1,LRP4,MAP1B,MEF2C,NRCAM,NTRK2,PTEN,PTPRD,ROBO2,FZD1,DLG5,NRXN1,MAGI2,FARP1,SEMA4D,IL1RAPL1,NLGN1,CLSTN1,SHANK2,ADNP,FLRT3,FLRT2,LRRTM2,NPTN,PCDH17,PCLO,SIX4,CNTN5,PPP1R9A,VPS35,LRRN1,SLITRK6,SLITRK2,SLITRK1,SLITRK4,LINGO2,SDK1,LRRTM3,AGRN |
| 9_Member | GO Biological Processes | GO:0051963 | regulation of synapse assembly | -7.076543327 | -5.531 | 31/106 | 351,1946,2045,2048,2895,4208,4915,5789,6092,8321,9231,9378,10507,11141,22871,22883,23394,23767,23768,26045,51804,55607,55737,57633,84189,84631,114798,139065,158038,347731,375790 | APP,EFNA5,EPHA7,EPHB2,GRID2,MEF2C,NTRK2,PTPRD,ROBO2,FZD1,DLG5,NRXN1,SEMA4D,IL1RAPL1,NLGN1,CLSTN1,ADNP,FLRT3,FLRT2,LRRTM2,SIX4,PPP1R9A,VPS35,LRRN1,SLITRK6,SLITRK2,SLITRK1,SLITRK4,LINGO2,LRRTM3,AGRN |
| 9_Member | GO Biological Processes | GO:0051965 | positive regulation of synapse assembly | -6.669837776 | -5.170 | 23/68 | 1946,2048,2895,4915,5789,9231,9378,10507,11141,22871,22883,23394,23767,23768,26045,57633,84189,84631,114798,139065,158038,347731,375790 | EFNA5,EPHB2,GRID2,NTRK2,PTPRD,DLG5,NRXN1,SEMA4D,IL1RAPL1,NLGN1,CLSTN1,ADNP,FLRT3,FLRT2,LRRTM2,LRRN1,SLITRK6,SLITRK2,SLITRK1,SLITRK4,LINGO2,LRRTM3,AGRN |
| 9_Member | GO Biological Processes | GO:0099560 | synaptic membrane adhesion | -6.27851441 | -4.826 | 13/26 | 1007,1946,5100,5789,5792,9378,22854,22871,23767,27253,57689,84631,114798 | CDH9,EFNA5,PCDH8,PTPRD,PTPRF,NRXN1,NTNG1,NLGN1,FLRT3,PCDH17,LRRC4C,SLITRK2,SLITRK1 |
| 9_Member | GO Biological Processes | GO:0099172 | presynapse organization | -4.351503478 | -3.106 | 15/46 | 351,4038,5728,5789,8321,8499,9378,11141,22871,27253,53942,55737,84631,114798,347731 | APP,LRP4,PTEN,PTPRD,FZD1,PPFIA2,NRXN1,IL1RAPL1,NLGN1,PCDH17,CNTN5,VPS35,SLITRK2,SLITRK1,LRRTM3 |
| 9_Member | GO Biological Processes | GO:0099054 | presynapse assembly | -4.091388576 | -2.880 | 14/43 | 351,4038,5728,5789,8321,9378,11141,22871,27253,53942,55737,84631,114798,347731 | APP,LRP4,PTEN,PTPRD,FZD1,NRXN1,IL1RAPL1,NLGN1,PCDH17,CNTN5,VPS35,SLITRK2,SLITRK1,LRRTM3 |
| 10_Summary | GO Cellular Components | GO:0030424 | axon | -28.12749121 | -25.255 | 160/607 | 60,287,288,323,351,387,547,667,747,793,1006,1176,1268,1385,1393,1739,1740,1826,1837,2017,2043,2044,2048,2059,2185,2332,2571,2572,2823,2898,2917,3064,3084,3643,3736,3737,3739,3745,3746,3747,3756,3776,3799,3800,4131,4133,4134,4628,4685,4763,4781,4803,4856,4897,4915,4919,4986,5048,5101,5173,5368,5563,5594,5599,5727,5796,5799,5868,6091,6092,6323,6326,6328,6334,6416,6453,6529,6571,6616,6683,6695,6711,6857,6860,7042,7074,7224,7248,7402,7531,7881,8087,8239,8408,8411,8546,8633,8766,8828,8829,8851,8867,8898,9053,9456,9860,10006,10092,10236,10479,10497,10533,11127,11141,22848,22941,23001,23011,23064,23077,23098,23114,23162,23208,23242,23394,23767,26053,26230,26278,26985,27020,27242,27255,53616,54407,55207,55607,55638,55714,57154,57282,57556,57616,58155,60482,63915,81565,81846,84665,91752,94056,127829,130074,136319,140767,152330,253559,348980,440279 | ACTB,ANK2,ANK3,APBB2,APP,RHOA,KIF1A,DST,DAGLA,CALB1,CDH8,AP3S1,CNR1,CREB1,CRHBP,DLG1,DLG2,DSCAM,DTNA,CTTN,EPHA4,EPHA5,EPHB2,EPS8,PTK2B,FMR1,GAD1,GAD2,GPM6A,GRIK2,GRM7,HTT,NRG1,INSR,KCNA1,KCNA2,KCNA4,KCNB1,KCNC1,KCNC2,KCNH1,KCNK2,KIF5B,KIF5C,MAP1B,MAP2,MAP4,MYH10,NCAM2,NF1,NFIB,NGF,CCN3,NRCAM,NTRK2,ROR1,OPRK1,PAFAH1B1,PCDH9,PDYN,PNOC,PRKAA2,MAPK1,MAPK8,PTCH1,PTPRK,PTPRN2,RAB5A,ROBO1,ROBO2,SCN1A,SCN2A,SCN3A,SCN8A,MAP2K4,ITSN1,SLC6A1,SLC18A2,SNAP25,SPAST,SPOCK1,SPTBN1,SYT1,SYT4,TGFB2,TIAM1,TRPC5,TSC1,UTRN,YWHAE,KCNAB1,FXR1,USP9X,ULK1,EEA1,AP3B1,UNC5C,RAB11A,NRP2,NRP1,CDK5R1,SYNJ1,MTMR2,MAP7,HOMER1,LRIG2,ABI1,ARPC5,HNRNPR,SLC9A6,UNC13B,ATG7,KIF3A,IL1RAPL1,AAK1,SHANK2,WDFY3,RAB21,SETX,MYCBP2,SARM1,NFASC,MAPK8IP3,SYT11,COBL,ADNP,FLRT3,AUTS2,TIAM2,SACS,AP3M1,NPTN,TNFRSF21,CNTN6,ADAM22,SLC38A2,ARL8B,PPP1R9A,SYBU,TENM3,SMURF1,SLC4A10,SEMA6A,TSHZ3,PTBP2,SLC5A7,BLOC1S5,NDEL1,SBF2,MYPN,ZNF804A,SYAP1,ARL8A,FAM168B,MTPN,NRSN1,CNTN4,CADM2,HCN1,UNC13C |
| 10_Member | GO Cellular Components | GO:0030424 | axon | -28.12749121 | -25.255 | 160/607 | 60,287,288,323,351,387,547,667,747,793,1006,1176,1268,1385,1393,1739,1740,1826,1837,2017,2043,2044,2048,2059,2185,2332,2571,2572,2823,2898,2917,3064,3084,3643,3736,3737,3739,3745,3746,3747,3756,3776,3799,3800,4131,4133,4134,4628,4685,4763,4781,4803,4856,4897,4915,4919,4986,5048,5101,5173,5368,5563,5594,5599,5727,5796,5799,5868,6091,6092,6323,6326,6328,6334,6416,6453,6529,6571,6616,6683,6695,6711,6857,6860,7042,7074,7224,7248,7402,7531,7881,8087,8239,8408,8411,8546,8633,8766,8828,8829,8851,8867,8898,9053,9456,9860,10006,10092,10236,10479,10497,10533,11127,11141,22848,22941,23001,23011,23064,23077,23098,23114,23162,23208,23242,23394,23767,26053,26230,26278,26985,27020,27242,27255,53616,54407,55207,55607,55638,55714,57154,57282,57556,57616,58155,60482,63915,81565,81846,84665,91752,94056,127829,130074,136319,140767,152330,253559,348980,440279 | ACTB,ANK2,ANK3,APBB2,APP,RHOA,KIF1A,DST,DAGLA,CALB1,CDH8,AP3S1,CNR1,CREB1,CRHBP,DLG1,DLG2,DSCAM,DTNA,CTTN,EPHA4,EPHA5,EPHB2,EPS8,PTK2B,FMR1,GAD1,GAD2,GPM6A,GRIK2,GRM7,HTT,NRG1,INSR,KCNA1,KCNA2,KCNA4,KCNB1,KCNC1,KCNC2,KCNH1,KCNK2,KIF5B,KIF5C,MAP1B,MAP2,MAP4,MYH10,NCAM2,NF1,NFIB,NGF,CCN3,NRCAM,NTRK2,ROR1,OPRK1,PAFAH1B1,PCDH9,PDYN,PNOC,PRKAA2,MAPK1,MAPK8,PTCH1,PTPRK,PTPRN2,RAB5A,ROBO1,ROBO2,SCN1A,SCN2A,SCN3A,SCN8A,MAP2K4,ITSN1,SLC6A1,SLC18A2,SNAP25,SPAST,SPOCK1,SPTBN1,SYT1,SYT4,TGFB2,TIAM1,TRPC5,TSC1,UTRN,YWHAE,KCNAB1,FXR1,USP9X,ULK1,EEA1,AP3B1,UNC5C,RAB11A,NRP2,NRP1,CDK5R1,SYNJ1,MTMR2,MAP7,HOMER1,LRIG2,ABI1,ARPC5,HNRNPR,SLC9A6,UNC13B,ATG7,KIF3A,IL1RAPL1,AAK1,SHANK2,WDFY3,RAB21,SETX,MYCBP2,SARM1,NFASC,MAPK8IP3,SYT11,COBL,ADNP,FLRT3,AUTS2,TIAM2,SACS,AP3M1,NPTN,TNFRSF21,CNTN6,ADAM22,SLC38A2,ARL8B,PPP1R9A,SYBU,TENM3,SMURF1,SLC4A10,SEMA6A,TSHZ3,PTBP2,SLC5A7,BLOC1S5,NDEL1,SBF2,MYPN,ZNF804A,SYAP1,ARL8A,FAM168B,MTPN,NRSN1,CNTN4,CADM2,HCN1,UNC13C |
| 10_Member | GO Cellular Components | GO:0033267 | axon part | -17.50878012 | -15.215 | 100/382 | 60,287,288,323,351,547,667,747,793,1006,1176,1268,1393,1739,1740,1826,2017,2043,2059,2185,2332,2571,2823,2898,3736,3737,3746,3747,3776,3799,3800,4131,4133,4628,4897,4915,4919,4986,5048,5101,5173,5368,5727,5799,5868,6092,6323,6326,6334,6453,6571,6616,6683,6695,6711,7074,7224,7248,7402,7531,7881,8239,8411,8546,8633,8829,8851,8867,9860,10006,10092,10236,10479,10497,11127,22848,22941,23011,23064,23114,23162,23208,23242,23767,26053,26230,26985,55207,55607,55638,57282,57616,58155,63915,81565,91752,94056,127829,140767,440279 | ACTB,ANK2,ANK3,APBB2,APP,KIF1A,DST,DAGLA,CALB1,CDH8,AP3S1,CNR1,CRHBP,DLG1,DLG2,DSCAM,CTTN,EPHA4,EPS8,PTK2B,FMR1,GAD1,GPM6A,GRIK2,KCNA1,KCNA2,KCNC1,KCNC2,KCNK2,KIF5B,KIF5C,MAP1B,MAP2,MYH10,NRCAM,NTRK2,ROR1,OPRK1,PAFAH1B1,PCDH9,PDYN,PNOC,PTCH1,PTPRN2,RAB5A,ROBO2,SCN1A,SCN2A,SCN8A,ITSN1,SLC18A2,SNAP25,SPAST,SPOCK1,SPTBN1,TIAM1,TRPC5,TSC1,UTRN,YWHAE,KCNAB1,USP9X,EEA1,AP3B1,UNC5C,NRP1,CDK5R1,SYNJ1,LRIG2,ABI1,ARPC5,HNRNPR,SLC9A6,UNC13B,KIF3A,AAK1,SHANK2,RAB21,SETX,NFASC,MAPK8IP3,SYT11,COBL,FLRT3,AUTS2,TIAM2,AP3M1,ARL8B,PPP1R9A,SYBU,SLC4A10,TSHZ3,PTBP2,BLOC1S5,NDEL1,ZNF804A,SYAP1,ARL8A,NRSN1,UNC13C |
| 10_Member | GO Cellular Components | GO:0150034 | distal axon | -12.44604055 | -10.488 | 73/285 | 60,323,351,793,1006,1268,1393,1826,2017,2043,2059,2185,2332,2571,2823,2898,3736,3737,3746,3747,3776,3799,3800,4131,4133,4628,4915,4919,4986,5048,5101,5173,5368,5727,5799,5868,6453,6571,6616,7074,7224,7248,7402,7531,8239,8633,8829,8851,8867,9860,10006,10092,10236,10479,10497,22848,22941,23064,23162,23208,23242,23767,26053,26230,55607,57282,57616,58155,81565,91752,94056,140767,440279 | ACTB,APBB2,APP,CALB1,CDH8,CNR1,CRHBP,DSCAM,CTTN,EPHA4,EPS8,PTK2B,FMR1,GAD1,GPM6A,GRIK2,KCNA1,KCNA2,KCNC1,KCNC2,KCNK2,KIF5B,KIF5C,MAP1B,MAP2,MYH10,NTRK2,ROR1,OPRK1,PAFAH1B1,PCDH9,PDYN,PNOC,PTCH1,PTPRN2,RAB5A,ITSN1,SLC18A2,SNAP25,TIAM1,TRPC5,TSC1,UTRN,YWHAE,USP9X,UNC5C,NRP1,CDK5R1,SYNJ1,LRIG2,ABI1,ARPC5,HNRNPR,SLC9A6,UNC13B,AAK1,SHANK2,SETX,MAPK8IP3,SYT11,COBL,FLRT3,AUTS2,TIAM2,PPP1R9A,SLC4A10,TSHZ3,PTBP2,NDEL1,ZNF804A,SYAP1,NRSN1,UNC13C |
| 10_Member | GO Cellular Components | GO:0030426 | growth cone | -8.841227527 | -7.138 | 46/171 | 323,351,1268,1826,2017,2043,2059,2185,2332,2823,3799,3800,4131,4133,4628,5048,5101,5727,6616,7074,7224,7248,7402,7531,8239,8633,8829,8851,9860,10006,10092,10236,22941,23064,23162,23242,23767,26053,26230,55607,57616,58155,81565,91752,94056,140767 | APBB2,APP,CNR1,DSCAM,CTTN,EPHA4,EPS8,PTK2B,FMR1,GPM6A,KIF5B,KIF5C,MAP1B,MAP2,MYH10,PAFAH1B1,PCDH9,PTCH1,SNAP25,TIAM1,TRPC5,TSC1,UTRN,YWHAE,USP9X,UNC5C,NRP1,CDK5R1,LRIG2,ABI1,ARPC5,HNRNPR,SHANK2,SETX,MAPK8IP3,COBL,FLRT3,AUTS2,TIAM2,PPP1R9A,TSHZ3,PTBP2,NDEL1,ZNF804A,SYAP1,NRSN1 |
| 10_Member | GO Cellular Components | GO:0030427 | site of polarized growth | -8.405683937 | -6.744 | 46/176 | 323,351,1268,1826,2017,2043,2059,2185,2332,2823,3799,3800,4131,4133,4628,5048,5101,5727,6616,7074,7224,7248,7402,7531,8239,8633,8829,8851,9860,10006,10092,10236,22941,23064,23162,23242,23767,26053,26230,55607,57616,58155,81565,91752,94056,140767 | APBB2,APP,CNR1,DSCAM,CTTN,EPHA4,EPS8,PTK2B,FMR1,GPM6A,KIF5B,KIF5C,MAP1B,MAP2,MYH10,PAFAH1B1,PCDH9,PTCH1,SNAP25,TIAM1,TRPC5,TSC1,UTRN,YWHAE,USP9X,UNC5C,NRP1,CDK5R1,LRIG2,ABI1,ARPC5,HNRNPR,SHANK2,SETX,MAPK8IP3,COBL,FLRT3,AUTS2,TIAM2,PPP1R9A,TSHZ3,PTBP2,NDEL1,ZNF804A,SYAP1,NRSN1 |
| 11_Summary | GO Biological Processes | GO:0007423 | sensory organ development | -27.74466579 | -24.913 | 149/548 | 102,182,357,490,493,639,650,654,655,659,775,793,890,1027,1028,1282,1289,1296,1499,1641,1739,1826,1889,1956,2043,2048,2138,2195,2200,2201,2255,2263,2295,2558,2625,2736,2737,2782,2823,3198,3638,3714,3725,3776,3815,4040,4041,4094,4124,4212,4286,4628,4760,4762,4763,4810,4851,4915,4919,5048,5156,5308,5316,5469,5584,5594,5629,5797,6256,6299,6497,6654,6656,6657,6664,6670,6722,6909,6935,7020,7021,7042,7046,7048,7068,7101,7291,7528,8013,8289,8829,9314,9474,9497,9547,9620,9839,9927,10152,10194,10210,10253,10265,10427,10586,10818,22882,22931,23047,23418,23493,26018,26281,28514,28996,30812,51761,51804,54567,54796,54806,55084,55636,55714,56853,57381,57822,63976,64093,64919,65979,79977,84189,84465,84678,85407,91851,93986,115908,121227,125488,130497,157506,167153,168667,204851,221935,284217,348980 | ADAM10,JAG1,SHROOM2,ATP2B1,ATP2B4,PRDM1,BMP2,BMP6,BMP7,BMPR2,CACNA1C,CALB1,CCNA2,CDKN1B,CDKN1C,COL4A1,COL5A1,COL8A2,CTNNB1,DCX,DLG1,DSCAM,ECE1,EGFR,EPHA4,EPHB2,EYA1,FAT1,FBN1,FBN2,FGF10,FGFR2,FOXF2,GABRA5,GATA3,GLI2,GLI3,GNB1,GPM6A,HOXA1,INSIG1,JAG2,JUN,KCNK2,KIT,LRP6,LRP5,MAF,MAN2A1,MEIS2,MITF,MYH10,NEUROD1,NEUROG1,NF1,NHS,NOTCH1,NTRK2,ROR1,PAFAH1B1,PDGFRA,PITX2,PKNOX1,MED1,PRKCI,MAPK1,PROX1,PTPRM,RXRA,SALL1,SKI,SOS1,SOX1,SOX2,SOX11,SP3,SRF,TBX2,ZEB1,TFAP2A,TFAP2B,TGFB2,TGFBR1,TGFBR2,THRB,NR2E1,TWIST1,YY1,NR4A3,ARID1A,NRP1,KLF4,ATG5,SLC4A7,CXCL14,CELSR1,ZEB2,MFN2,ABI2,TSHZ1,TOPORS,SPRY2,IRX5,SEC24B,MAB21L2,FRS2,ZHX2,RAB18,PDS5B,CRB1,HEY2,LRIG1,FGF20,DLL1,HIPK2,SOX8,ATP8A2,SIX4,DLL4,BNC2,AHI1,SOBP,CHD7,TENM3,CELF4,RHOJ,GRHL3,PRDM16,SMOC1,BCL11B,PHACTR4,GRHL2,SLITRK6,MEGF11,KDM2B,NKD1,CHRDL1,FOXP2,CTHRC1,LRIG3,TTC39C,OSR1,RDH10,TENT2,BMPER,HIPK1,SDK1,LAMA1,HCN1 |
| 11_Member | GO Biological Processes | GO:0007423 | sensory organ development | -27.74466579 | -24.913 | 149/548 | 102,182,357,490,493,639,650,654,655,659,775,793,890,1027,1028,1282,1289,1296,1499,1641,1739,1826,1889,1956,2043,2048,2138,2195,2200,2201,2255,2263,2295,2558,2625,2736,2737,2782,2823,3198,3638,3714,3725,3776,3815,4040,4041,4094,4124,4212,4286,4628,4760,4762,4763,4810,4851,4915,4919,5048,5156,5308,5316,5469,5584,5594,5629,5797,6256,6299,6497,6654,6656,6657,6664,6670,6722,6909,6935,7020,7021,7042,7046,7048,7068,7101,7291,7528,8013,8289,8829,9314,9474,9497,9547,9620,9839,9927,10152,10194,10210,10253,10265,10427,10586,10818,22882,22931,23047,23418,23493,26018,26281,28514,28996,30812,51761,51804,54567,54796,54806,55084,55636,55714,56853,57381,57822,63976,64093,64919,65979,79977,84189,84465,84678,85407,91851,93986,115908,121227,125488,130497,157506,167153,168667,204851,221935,284217,348980 | ADAM10,JAG1,SHROOM2,ATP2B1,ATP2B4,PRDM1,BMP2,BMP6,BMP7,BMPR2,CACNA1C,CALB1,CCNA2,CDKN1B,CDKN1C,COL4A1,COL5A1,COL8A2,CTNNB1,DCX,DLG1,DSCAM,ECE1,EGFR,EPHA4,EPHB2,EYA1,FAT1,FBN1,FBN2,FGF10,FGFR2,FOXF2,GABRA5,GATA3,GLI2,GLI3,GNB1,GPM6A,HOXA1,INSIG1,JAG2,JUN,KCNK2,KIT,LRP6,LRP5,MAF,MAN2A1,MEIS2,MITF,MYH10,NEUROD1,NEUROG1,NF1,NHS,NOTCH1,NTRK2,ROR1,PAFAH1B1,PDGFRA,PITX2,PKNOX1,MED1,PRKCI,MAPK1,PROX1,PTPRM,RXRA,SALL1,SKI,SOS1,SOX1,SOX2,SOX11,SP3,SRF,TBX2,ZEB1,TFAP2A,TFAP2B,TGFB2,TGFBR1,TGFBR2,THRB,NR2E1,TWIST1,YY1,NR4A3,ARID1A,NRP1,KLF4,ATG5,SLC4A7,CXCL14,CELSR1,ZEB2,MFN2,ABI2,TSHZ1,TOPORS,SPRY2,IRX5,SEC24B,MAB21L2,FRS2,ZHX2,RAB18,PDS5B,CRB1,HEY2,LRIG1,FGF20,DLL1,HIPK2,SOX8,ATP8A2,SIX4,DLL4,BNC2,AHI1,SOBP,CHD7,TENM3,CELF4,RHOJ,GRHL3,PRDM16,SMOC1,BCL11B,PHACTR4,GRHL2,SLITRK6,MEGF11,KDM2B,NKD1,CHRDL1,FOXP2,CTHRC1,LRIG3,TTC39C,OSR1,RDH10,TENT2,BMPER,HIPK1,SDK1,LAMA1,HCN1 |
| 11_Member | GO Biological Processes | GO:0001654 | eye development | -24.28180241 | -21.672 | 109/362 | 182,357,490,493,639,654,655,659,775,793,1027,1028,1282,1289,1296,1499,1641,1739,1826,1956,2048,2195,2200,2201,2255,2295,2625,2737,2782,2823,3725,4040,4041,4094,4124,4212,4286,4628,4760,4763,4810,4915,5156,5308,5316,5469,5584,5629,5797,6256,6497,6654,6656,6657,6664,6670,6722,6909,6935,7020,7021,7042,7046,7048,7068,7101,7291,7528,8289,8829,9314,9839,9927,10152,10210,10265,10586,10818,22882,22931,23047,23418,28514,28996,30812,51761,54567,54806,55636,55714,56853,57381,57822,64093,64919,65979,79977,84189,84465,84678,85407,91851,93986,157506,167153,204851,221935,284217,348980 | JAG1,SHROOM2,ATP2B1,ATP2B4,PRDM1,BMP6,BMP7,BMPR2,CACNA1C,CALB1,CDKN1B,CDKN1C,COL4A1,COL5A1,COL8A2,CTNNB1,DCX,DLG1,DSCAM,EGFR,EPHB2,FAT1,FBN1,FBN2,FGF10,FOXF2,GATA3,GLI3,GNB1,GPM6A,JUN,LRP6,LRP5,MAF,MAN2A1,MEIS2,MITF,MYH10,NEUROD1,NF1,NHS,NTRK2,PDGFRA,PITX2,PKNOX1,MED1,PRKCI,PROX1,PTPRM,RXRA,SKI,SOS1,SOX1,SOX2,SOX11,SP3,SRF,TBX2,ZEB1,TFAP2A,TFAP2B,TGFB2,TGFBR1,TGFBR2,THRB,NR2E1,TWIST1,YY1,ARID1A,NRP1,KLF4,ZEB2,MFN2,ABI2,TOPORS,IRX5,MAB21L2,FRS2,ZHX2,RAB18,PDS5B,CRB1,DLL1,HIPK2,SOX8,ATP8A2,DLL4,AHI1,CHD7,TENM3,CELF4,RHOJ,GRHL3,SMOC1,BCL11B,PHACTR4,GRHL2,SLITRK6,MEGF11,KDM2B,NKD1,CHRDL1,FOXP2,RDH10,TENT2,HIPK1,SDK1,LAMA1,HCN1 |
| 11_Member | GO Biological Processes | GO:0150063 | visual system development | -23.84968572 | -21.255 | 109/366 | 182,357,490,493,639,654,655,659,775,793,1027,1028,1282,1289,1296,1499,1641,1739,1826,1956,2048,2195,2200,2201,2255,2295,2625,2737,2782,2823,3725,4040,4041,4094,4124,4212,4286,4628,4760,4763,4810,4915,5156,5308,5316,5469,5584,5629,5797,6256,6497,6654,6656,6657,6664,6670,6722,6909,6935,7020,7021,7042,7046,7048,7068,7101,7291,7528,8289,8829,9314,9839,9927,10152,10210,10265,10586,10818,22882,22931,23047,23418,28514,28996,30812,51761,54567,54806,55636,55714,56853,57381,57822,64093,64919,65979,79977,84189,84465,84678,85407,91851,93986,157506,167153,204851,221935,284217,348980 | JAG1,SHROOM2,ATP2B1,ATP2B4,PRDM1,BMP6,BMP7,BMPR2,CACNA1C,CALB1,CDKN1B,CDKN1C,COL4A1,COL5A1,COL8A2,CTNNB1,DCX,DLG1,DSCAM,EGFR,EPHB2,FAT1,FBN1,FBN2,FGF10,FOXF2,GATA3,GLI3,GNB1,GPM6A,JUN,LRP6,LRP5,MAF,MAN2A1,MEIS2,MITF,MYH10,NEUROD1,NF1,NHS,NTRK2,PDGFRA,PITX2,PKNOX1,MED1,PRKCI,PROX1,PTPRM,RXRA,SKI,SOS1,SOX1,SOX2,SOX11,SP3,SRF,TBX2,ZEB1,TFAP2A,TFAP2B,TGFB2,TGFBR1,TGFBR2,THRB,NR2E1,TWIST1,YY1,ARID1A,NRP1,KLF4,ZEB2,MFN2,ABI2,TOPORS,IRX5,MAB21L2,FRS2,ZHX2,RAB18,PDS5B,CRB1,DLL1,HIPK2,SOX8,ATP8A2,DLL4,AHI1,CHD7,TENM3,CELF4,RHOJ,GRHL3,SMOC1,BCL11B,PHACTR4,GRHL2,SLITRK6,MEGF11,KDM2B,NKD1,CHRDL1,FOXP2,RDH10,TENT2,HIPK1,SDK1,LAMA1,HCN1 |
| 11_Member | GO Biological Processes | GO:0048880 | sensory system development | -23.32082903 | -20.796 | 109/371 | 182,357,490,493,639,654,655,659,775,793,1027,1028,1282,1289,1296,1499,1641,1739,1826,1956,2048,2195,2200,2201,2255,2295,2625,2737,2782,2823,3725,4040,4041,4094,4124,4212,4286,4628,4760,4763,4810,4915,5156,5308,5316,5469,5584,5629,5797,6256,6497,6654,6656,6657,6664,6670,6722,6909,6935,7020,7021,7042,7046,7048,7068,7101,7291,7528,8289,8829,9314,9839,9927,10152,10210,10265,10586,10818,22882,22931,23047,23418,28514,28996,30812,51761,54567,54806,55636,55714,56853,57381,57822,64093,64919,65979,79977,84189,84465,84678,85407,91851,93986,157506,167153,204851,221935,284217,348980 | JAG1,SHROOM2,ATP2B1,ATP2B4,PRDM1,BMP6,BMP7,BMPR2,CACNA1C,CALB1,CDKN1B,CDKN1C,COL4A1,COL5A1,COL8A2,CTNNB1,DCX,DLG1,DSCAM,EGFR,EPHB2,FAT1,FBN1,FBN2,FGF10,FOXF2,GATA3,GLI3,GNB1,GPM6A,JUN,LRP6,LRP5,MAF,MAN2A1,MEIS2,MITF,MYH10,NEUROD1,NF1,NHS,NTRK2,PDGFRA,PITX2,PKNOX1,MED1,PRKCI,PROX1,PTPRM,RXRA,SKI,SOS1,SOX1,SOX2,SOX11,SP3,SRF,TBX2,ZEB1,TFAP2A,TFAP2B,TGFB2,TGFBR1,TGFBR2,THRB,NR2E1,TWIST1,YY1,ARID1A,NRP1,KLF4,ZEB2,MFN2,ABI2,TOPORS,IRX5,MAB21L2,FRS2,ZHX2,RAB18,PDS5B,CRB1,DLL1,HIPK2,SOX8,ATP8A2,DLL4,AHI1,CHD7,TENM3,CELF4,RHOJ,GRHL3,SMOC1,BCL11B,PHACTR4,GRHL2,SLITRK6,MEGF11,KDM2B,NKD1,CHRDL1,FOXP2,RDH10,TENT2,HIPK1,SDK1,LAMA1,HCN1 |
| 11_Member | GO Biological Processes | GO:0043010 | camera-type eye development | -23.17278639 | -20.655 | 98/314 | 182,357,490,493,655,659,775,793,1027,1028,1282,1296,1499,1641,1739,1826,1956,2048,2195,2200,2201,2255,2295,2625,2737,2782,2823,3725,4040,4041,4094,4124,4286,4628,4760,4763,4810,4915,5156,5308,5316,5469,5629,5797,6256,6497,6654,6656,6664,6670,6722,6909,6935,7020,7021,7042,7046,7048,7068,7101,7291,7528,8289,8829,9314,9839,9927,10152,10210,10265,10586,10818,22882,23047,28514,28996,30812,51761,54567,54806,55636,55714,56853,57381,57822,64919,65979,79977,84189,84465,84678,93986,157506,167153,204851,221935,284217,348980 | JAG1,SHROOM2,ATP2B1,ATP2B4,BMP7,BMPR2,CACNA1C,CALB1,CDKN1B,CDKN1C,COL4A1,COL8A2,CTNNB1,DCX,DLG1,DSCAM,EGFR,EPHB2,FAT1,FBN1,FBN2,FGF10,FOXF2,GATA3,GLI3,GNB1,GPM6A,JUN,LRP6,LRP5,MAF,MAN2A1,MITF,MYH10,NEUROD1,NF1,NHS,NTRK2,PDGFRA,PITX2,PKNOX1,MED1,PROX1,PTPRM,RXRA,SKI,SOS1,SOX1,SOX11,SP3,SRF,TBX2,ZEB1,TFAP2A,TFAP2B,TGFB2,TGFBR1,TGFBR2,THRB,NR2E1,TWIST1,YY1,ARID1A,NRP1,KLF4,ZEB2,MFN2,ABI2,TOPORS,IRX5,MAB21L2,FRS2,ZHX2,PDS5B,DLL1,HIPK2,SOX8,ATP8A2,DLL4,AHI1,CHD7,TENM3,CELF4,RHOJ,GRHL3,BCL11B,PHACTR4,GRHL2,SLITRK6,MEGF11,KDM2B,FOXP2,RDH10,TENT2,HIPK1,SDK1,LAMA1,HCN1 |
| 11_Member | GO Biological Processes | GO:0090596 | sensory organ morphogenesis | -19.67111294 | -17.284 | 81/256 | 182,357,639,655,793,1289,1296,1499,1826,2048,2138,2195,2200,2201,2255,2263,2295,2625,2736,2737,3198,3638,4040,4041,4124,4762,4763,4915,5308,5584,5594,5629,5797,6299,6497,6656,6664,6670,6909,6935,7020,7021,7068,7291,7528,8013,8289,9620,9927,10152,10194,10210,10253,10265,10427,10818,22882,23418,26018,28514,28996,30812,51761,51804,54806,55084,55636,55714,57822,65979,84189,84465,84678,85407,115908,121227,125488,130497,204851,221935,348980 | JAG1,SHROOM2,PRDM1,BMP7,CALB1,COL5A1,COL8A2,CTNNB1,DSCAM,EPHB2,EYA1,FAT1,FBN1,FBN2,FGF10,FGFR2,FOXF2,GATA3,GLI2,GLI3,HOXA1,INSIG1,LRP6,LRP5,MAN2A1,NEUROG1,NF1,NTRK2,PITX2,PRKCI,MAPK1,PROX1,PTPRM,SALL1,SKI,SOX1,SOX11,SP3,TBX2,ZEB1,TFAP2A,TFAP2B,THRB,TWIST1,YY1,NR4A3,ARID1A,CELSR1,MFN2,ABI2,TSHZ1,TOPORS,SPRY2,IRX5,SEC24B,FRS2,ZHX2,CRB1,LRIG1,DLL1,HIPK2,SOX8,ATP8A2,SIX4,AHI1,SOBP,CHD7,TENM3,GRHL3,PHACTR4,SLITRK6,MEGF11,KDM2B,NKD1,CTHRC1,LRIG3,TTC39C,OSR1,HIPK1,SDK1,HCN1 |
| 11_Member | GO Biological Processes | GO:0048593 | camera-type eye morphogenesis | -17.96627807 | -15.653 | 48/112 | 182,357,655,793,1296,1499,1826,2048,2195,2295,2737,4040,4041,4124,4763,5308,5629,5797,6497,6656,6664,6670,6909,6935,7020,7021,7068,7291,7528,8289,9927,10152,10210,10265,10818,22882,28514,28996,30812,51761,54806,55714,65979,84465,84678,204851,221935,348980 | JAG1,SHROOM2,BMP7,CALB1,COL8A2,CTNNB1,DSCAM,EPHB2,FAT1,FOXF2,GLI3,LRP6,LRP5,MAN2A1,NF1,PITX2,PROX1,PTPRM,SKI,SOX1,SOX11,SP3,TBX2,ZEB1,TFAP2A,TFAP2B,THRB,TWIST1,YY1,ARID1A,MFN2,ABI2,TOPORS,IRX5,FRS2,ZHX2,DLL1,HIPK2,SOX8,ATP8A2,AHI1,TENM3,PHACTR4,MEGF11,KDM2B,HIPK1,SDK1,HCN1 |
| 11_Member | GO Biological Processes | GO:0048592 | eye morphogenesis | -17.63077947 | -15.329 | 56/149 | 182,357,639,655,793,1289,1296,1499,1826,2048,2195,2200,2201,2295,2737,4040,4041,4124,4763,4915,5308,5584,5629,5797,6497,6656,6664,6670,6909,6935,7020,7021,7068,7291,7528,8289,9927,10152,10210,10265,10818,22882,23418,28514,28996,30812,51761,54806,55714,65979,84465,84678,85407,204851,221935,348980 | JAG1,SHROOM2,PRDM1,BMP7,CALB1,COL5A1,COL8A2,CTNNB1,DSCAM,EPHB2,FAT1,FBN1,FBN2,FOXF2,GLI3,LRP6,LRP5,MAN2A1,NF1,NTRK2,PITX2,PRKCI,PROX1,PTPRM,SKI,SOX1,SOX11,SP3,TBX2,ZEB1,TFAP2A,TFAP2B,THRB,TWIST1,YY1,ARID1A,MFN2,ABI2,TOPORS,IRX5,FRS2,ZHX2,CRB1,DLL1,HIPK2,SOX8,ATP8A2,AHI1,TENM3,PHACTR4,MEGF11,KDM2B,NKD1,HIPK1,SDK1,HCN1 |
| 11_Member | GO Biological Processes | GO:0060041 | retina development in camera-type eye | -10.44149437 | -8.616 | 44/144 | 490,493,659,793,1282,1641,1826,2782,2823,4040,4041,4124,4628,4760,4915,5156,5469,5629,5797,6497,7020,7021,7042,7068,7101,8829,10210,10265,22882,28514,28996,30812,51761,54567,54806,55636,56853,57381,84465,167153,204851,221935,284217,348980 | ATP2B1,ATP2B4,BMPR2,CALB1,COL4A1,DCX,DSCAM,GNB1,GPM6A,LRP6,LRP5,MAN2A1,MYH10,NEUROD1,NTRK2,PDGFRA,MED1,PROX1,PTPRM,SKI,TFAP2A,TFAP2B,TGFB2,THRB,NR2E1,NRP1,TOPORS,IRX5,ZHX2,DLL1,HIPK2,SOX8,ATP8A2,DLL4,AHI1,CHD7,CELF4,RHOJ,MEGF11,TENT2,HIPK1,SDK1,LAMA1,HCN1 |
| 11_Member | GO Biological Processes | GO:0060042 | retina morphogenesis in camera-type eye | -9.075585231 | -7.351 | 22/49 | 793,1826,4040,4041,4124,5629,5797,7020,7021,7068,10210,10265,22882,28514,28996,30812,51761,54806,84465,204851,221935,348980 | CALB1,DSCAM,LRP6,LRP5,MAN2A1,PROX1,PTPRM,TFAP2A,TFAP2B,THRB,TOPORS,IRX5,ZHX2,DLL1,HIPK2,SOX8,ATP8A2,AHI1,MEGF11,HIPK1,SDK1,HCN1 |
| 11_Member | GO Biological Processes | GO:0003407 | neural retina development | -7.525609808 | -5.944 | 23/62 | 490,493,793,1826,2823,4760,5797,7020,7021,7042,7068,10210,10265,22882,28996,30812,51761,54567,54806,84465,204851,221935,348980 | ATP2B1,ATP2B4,CALB1,DSCAM,GPM6A,NEUROD1,PTPRM,TFAP2A,TFAP2B,TGFB2,THRB,TOPORS,IRX5,ZHX2,HIPK2,SOX8,ATP8A2,DLL4,AHI1,MEGF11,HIPK1,SDK1,HCN1 |
| 11_Member | GO Biological Processes | GO:0010842 | retina layer formation | -6.370203443 | -4.904 | 12/22 | 793,1826,5797,7020,7021,10210,28996,51761,54806,84465,204851,221935 | CALB1,DSCAM,PTPRM,TFAP2A,TFAP2B,TOPORS,HIPK2,ATP8A2,AHI1,MEGF11,HIPK1,SDK1 |
| 12_Summary | GO Biological Processes | GO:0007610 | behavior | -27.06726233 | -24.261 | 156/596 | 18,27,320,321,351,372,463,793,1268,1271,1374,1385,1393,1600,1602,1812,1816,1826,1956,1958,1977,2033,2043,2048,2059,2115,2119,2334,2353,2475,2534,2558,2571,2668,2743,2764,2898,2903,2904,2911,2915,2917,3064,3084,3156,3170,3198,3358,3398,3643,3688,3725,3737,3776,3815,3845,4097,4208,4212,4762,4763,4803,4857,4862,4864,4915,4929,4986,5048,5079,5090,5270,5577,5581,5594,5649,5728,5803,6310,6323,6326,6529,6571,6616,6722,6860,7057,7068,7101,7248,7432,7436,7832,7881,8013,8301,8867,9369,9378,9455,9456,9611,9620,9698,10396,10499,10636,10752,11245,22849,22871,22941,22986,23230,23236,23373,23394,23544,25769,26058,26090,27020,27023,27253,28996,51009,51761,53616,54207,54806,55084,55342,55636,55737,55777,57282,57338,57468,57561,57578,60626,64854,64856,81627,84189,84251,89797,93986,114798,130507,139411,153222,221935,338811,342371,431707,4248,9993,801,1387,1843,1874,1965,2332,2776,2782,3157,3746,3747,4216,4734,5295,5501,5599,5796,6785,7528,8491,8533,9397,9575,10277,10714,23411,23596,51455,54617,55031,57646,94241,123879,150094,167153,1281,2138,2625,2885,7412,8493,10135,10276,64326 | ABAT,ABL2,APBA1,APBA2,APP,ARCN1,ZFHX3,CALB1,CNR1,CNTFR,CPT1A,CREB1,CRHBP,DAB1,DACH1,DRD1,DRD5,DSCAM,EGFR,EGR1,EIF4E,EP300,EPHA4,EPHB2,EPS8,ETV1,ETV5,AFF2,FOS,MTOR,FYN,GABRA5,GAD1,GDNF,GLRB,GMFB,GRIK2,GRIN2A,GRIN2B,GRM1,GRM5,GRM7,HTT,NRG1,HMGCR,FOXA2,HOXA1,HTR2C,ID2,INSR,ITGB1,JUN,KCNA2,KCNK2,KIT,KRAS,MAFG,MEF2C,MEIS2,NEUROG1,NF1,NGF,NOVA1,NPAS2,NPC1,NTRK2,NR4A2,OPRK1,PAFAH1B1,PAX5,PBX3,SERPINE2,PRKAR2B,PRKCE,MAPK1,RELN,PTEN,PTPRZ1,ATXN1,SCN1A,SCN2A,SLC6A1,SLC18A2,SNAP25,SRF,SYT4,THBS1,THRB,NR2E1,TSC1,VIP,VLDLR,BTG2,KCNAB1,NR4A3,PICALM,SYNJ1,NRXN3,NRXN1,HOMER2,HOMER1,NCOR1,CELSR1,PUM1,ATP8A1,NCOA2,RGS14,CHL1,GPR176,CPEB3,NLGN1,SHANK2,SORCS3,VPS13A,PLCB1,CRTC1,ADNP,SEZ6L,SLC24A2,GIGYF2,ABHD12,NPTN,FOXB1,PCDH17,HIPK2,DERL2,ATP8A2,ADAM22,KCNK10,AHI1,SOBP,STRBP,CHD7,VPS35,MBD5,SLC4A10,JPH3,SLC12A5,ARRDC3,UNC79,RIC8A,USP46,VWA1,TRMT1L,SLITRK6,SGIP1,NAV2,FOXP2,SLITRK1,UBR3,PTCHD1,CREBRF,SDK1,TAFA2,ATXN1L,LHX8,MGAT3,DGCR2,CALM1,CREBBP,DUSP1,E2F4,EIF2S1,FMR1,GNAQ,GNB1,HMGCS1,KCNC1,KCNC2,MAP3K4,NEDD4,PIK3R1,PPP1CC,MAPK8,PTPRK,ELOVL4,YY1,MAP4K3,COPS3,NMT2,CLOCK,UBE4B,POLD3,SIRT1,OPN3,REV1,INO80,USP47,USP28,TP53INP1,DCUN1D3,SIK1,TENT2,COL3A1,EYA1,GATA3,GRB2,VCAM1,PPM1D,NAMPT,NET1,COP1 |
| 12_Member | GO Biological Processes | GO:0007610 | behavior | -27.06726233 | -24.261 | 156/596 | 18,27,320,321,351,372,463,793,1268,1271,1374,1385,1393,1600,1602,1812,1816,1826,1956,1958,1977,2033,2043,2048,2059,2115,2119,2334,2353,2475,2534,2558,2571,2668,2743,2764,2898,2903,2904,2911,2915,2917,3064,3084,3156,3170,3198,3358,3398,3643,3688,3725,3737,3776,3815,3845,4097,4208,4212,4762,4763,4803,4857,4862,4864,4915,4929,4986,5048,5079,5090,5270,5577,5581,5594,5649,5728,5803,6310,6323,6326,6529,6571,6616,6722,6860,7057,7068,7101,7248,7432,7436,7832,7881,8013,8301,8867,9369,9378,9455,9456,9611,9620,9698,10396,10499,10636,10752,11245,22849,22871,22941,22986,23230,23236,23373,23394,23544,25769,26058,26090,27020,27023,27253,28996,51009,51761,53616,54207,54806,55084,55342,55636,55737,55777,57282,57338,57468,57561,57578,60626,64854,64856,81627,84189,84251,89797,93986,114798,130507,139411,153222,221935,338811,342371,431707 | ABAT,ABL2,APBA1,APBA2,APP,ARCN1,ZFHX3,CALB1,CNR1,CNTFR,CPT1A,CREB1,CRHBP,DAB1,DACH1,DRD1,DRD5,DSCAM,EGFR,EGR1,EIF4E,EP300,EPHA4,EPHB2,EPS8,ETV1,ETV5,AFF2,FOS,MTOR,FYN,GABRA5,GAD1,GDNF,GLRB,GMFB,GRIK2,GRIN2A,GRIN2B,GRM1,GRM5,GRM7,HTT,NRG1,HMGCR,FOXA2,HOXA1,HTR2C,ID2,INSR,ITGB1,JUN,KCNA2,KCNK2,KIT,KRAS,MAFG,MEF2C,MEIS2,NEUROG1,NF1,NGF,NOVA1,NPAS2,NPC1,NTRK2,NR4A2,OPRK1,PAFAH1B1,PAX5,PBX3,SERPINE2,PRKAR2B,PRKCE,MAPK1,RELN,PTEN,PTPRZ1,ATXN1,SCN1A,SCN2A,SLC6A1,SLC18A2,SNAP25,SRF,SYT4,THBS1,THRB,NR2E1,TSC1,VIP,VLDLR,BTG2,KCNAB1,NR4A3,PICALM,SYNJ1,NRXN3,NRXN1,HOMER2,HOMER1,NCOR1,CELSR1,PUM1,ATP8A1,NCOA2,RGS14,CHL1,GPR176,CPEB3,NLGN1,SHANK2,SORCS3,VPS13A,PLCB1,CRTC1,ADNP,SEZ6L,SLC24A2,GIGYF2,ABHD12,NPTN,FOXB1,PCDH17,HIPK2,DERL2,ATP8A2,ADAM22,KCNK10,AHI1,SOBP,STRBP,CHD7,VPS35,MBD5,SLC4A10,JPH3,SLC12A5,ARRDC3,UNC79,RIC8A,USP46,VWA1,TRMT1L,SLITRK6,SGIP1,NAV2,FOXP2,SLITRK1,UBR3,PTCHD1,CREBRF,SDK1,TAFA2,ATXN1L,LHX8 |
| 12_Member | GO Biological Processes | GO:0007611 | learning or memory | -15.05677334 | -12.931 | 73/256 | 351,793,1268,1385,1393,1812,1816,1956,2033,2048,2334,2353,2475,2534,2558,2764,2903,2904,2915,3064,3156,3643,3688,3725,3776,3815,3845,4208,4212,4762,4763,4803,4915,4986,5048,5577,5594,5649,5728,5803,6310,6326,6529,6616,6722,6860,7432,7436,7832,7881,8301,8867,9369,9378,10396,10636,22849,22941,22986,23236,23373,23394,25769,27020,27023,54207,57338,57468,60626,93986,338811,342371,431707 | APP,CALB1,CNR1,CREB1,CRHBP,DRD1,DRD5,EGFR,EP300,EPHB2,AFF2,FOS,MTOR,FYN,GABRA5,GMFB,GRIN2A,GRIN2B,GRM5,HTT,HMGCR,INSR,ITGB1,JUN,KCNK2,KIT,KRAS,MEF2C,MEIS2,NEUROG1,NF1,NGF,NTRK2,OPRK1,PAFAH1B1,PRKAR2B,MAPK1,RELN,PTEN,PTPRZ1,ATXN1,SCN2A,SLC6A1,SNAP25,SRF,SYT4,VIP,VLDLR,BTG2,KCNAB1,PICALM,SYNJ1,NRXN3,NRXN1,ATP8A1,RGS14,CPEB3,SHANK2,SORCS3,PLCB1,CRTC1,ADNP,SLC24A2,NPTN,FOXB1,KCNK10,JPH3,SLC12A5,RIC8A,FOXP2,TAFA2,ATXN1L,LHX8 |
| 12_Member | GO Biological Processes | GO:0050890 | cognition | -14.97575981 | -12.856 | 80/296 | 351,793,1268,1385,1393,1812,1816,1956,2033,2048,2334,2353,2475,2534,2558,2764,2903,2904,2915,3064,3156,3198,3643,3688,3725,3776,3815,3845,4208,4212,4248,4762,4763,4803,4915,4986,5048,5577,5594,5649,5728,5803,6310,6326,6529,6616,6722,6860,7432,7436,7832,7881,8301,8867,9369,9378,9993,10396,10636,10752,22849,22941,22986,23236,23373,23394,25769,27020,27023,54207,55084,55636,57338,57468,60626,93986,139411,338811,342371,431707 | APP,CALB1,CNR1,CREB1,CRHBP,DRD1,DRD5,EGFR,EP300,EPHB2,AFF2,FOS,MTOR,FYN,GABRA5,GMFB,GRIN2A,GRIN2B,GRM5,HTT,HMGCR,HOXA1,INSR,ITGB1,JUN,KCNK2,KIT,KRAS,MEF2C,MEIS2,MGAT3,NEUROG1,NF1,NGF,NTRK2,OPRK1,PAFAH1B1,PRKAR2B,MAPK1,RELN,PTEN,PTPRZ1,ATXN1,SCN2A,SLC6A1,SNAP25,SRF,SYT4,VIP,VLDLR,BTG2,KCNAB1,PICALM,SYNJ1,NRXN3,NRXN1,DGCR2,ATP8A1,RGS14,CHL1,CPEB3,SHANK2,SORCS3,PLCB1,CRTC1,ADNP,SLC24A2,NPTN,FOXB1,KCNK10,SOBP,CHD7,JPH3,SLC12A5,RIC8A,FOXP2,PTCHD1,TAFA2,ATXN1L,LHX8 |
| 12_Member | GO Biological Processes | GO:0007612 | learning | -11.5211659 | -9.625 | 46/145 | 351,1385,1812,1816,2048,2353,2475,2534,2558,2764,2903,2915,3064,3156,3643,3688,3725,3815,3845,4212,4763,4915,4986,5577,5649,6310,6529,6616,6722,7832,8867,9369,9378,10396,10636,22941,22986,25769,27020,27023,57338,57468,60626,93986,338811,342371 | APP,CREB1,DRD1,DRD5,EPHB2,FOS,MTOR,FYN,GABRA5,GMFB,GRIN2A,GRM5,HTT,HMGCR,INSR,ITGB1,JUN,KIT,KRAS,MEIS2,NF1,NTRK2,OPRK1,PRKAR2B,RELN,ATXN1,SLC6A1,SNAP25,SRF,BTG2,SYNJ1,NRXN3,NRXN1,ATP8A1,RGS14,SHANK2,SORCS3,SLC24A2,NPTN,FOXB1,JPH3,SLC12A5,RIC8A,FOXP2,TAFA2,ATXN1L |
| 12_Member | GO Biological Processes | GO:0008306 | associative learning | -6.069934373 | -4.636 | 24/78 | 351,1385,1812,1816,2353,2475,2558,2903,3156,3688,3815,3845,4212,4763,4986,5649,6616,6722,7832,10636,27020,27023,60626,338811 | APP,CREB1,DRD1,DRD5,FOS,MTOR,GABRA5,GRIN2A,HMGCR,ITGB1,KIT,KRAS,MEIS2,NF1,OPRK1,RELN,SNAP25,SRF,BTG2,RGS14,NPTN,FOXB1,RIC8A,TAFA2 |
| 12_Member | GO Biological Processes | GO:0009416 | response to light stimulus | -5.076638389 | -3.752 | 59/315 | 351,801,1385,1387,1812,1843,1874,1956,1965,2033,2332,2353,2475,2776,2782,2903,3156,3157,3198,3398,3688,3746,3747,3815,3845,4212,4216,4734,4763,5295,5501,5599,5796,6785,7528,8491,8533,9397,9575,10277,10636,10714,23373,23411,23596,27020,27023,51455,51761,54617,55031,57282,57646,60626,94241,123879,150094,167153,338811 | APP,CALM1,CREB1,CREBBP,DRD1,DUSP1,E2F4,EGFR,EIF2S1,EP300,FMR1,FOS,MTOR,GNAQ,GNB1,GRIN2A,HMGCR,HMGCS1,HOXA1,ID2,ITGB1,KCNC1,KCNC2,KIT,KRAS,MEIS2,MAP3K4,NEDD4,NF1,PIK3R1,PPP1CC,MAPK8,PTPRK,ELOVL4,YY1,MAP4K3,COPS3,NMT2,CLOCK,UBE4B,RGS14,POLD3,CRTC1,SIRT1,OPN3,NPTN,FOXB1,REV1,ATP8A2,INO80,USP47,SLC4A10,USP28,RIC8A,TP53INP1,DCUN1D3,SIK1,TENT2,TAFA2 |
| 12_Member | GO Biological Processes | GO:0007632 | visual behavior | -4.746159542 | -3.455 | 17/53 | 351,1385,1812,2475,2903,3156,3198,3688,3815,3845,4212,4763,10636,27020,27023,60626,338811 | APP,CREB1,DRD1,MTOR,GRIN2A,HMGCR,HOXA1,ITGB1,KIT,KRAS,MEIS2,NF1,RGS14,NPTN,FOXB1,RIC8A,TAFA2 |
| 12_Member | GO Biological Processes | GO:0008542 | visual learning | -4.738100297 | -3.448 | 16/48 | 351,1385,1812,2475,2903,3156,3688,3815,3845,4212,4763,10636,27020,27023,60626,338811 | APP,CREB1,DRD1,MTOR,GRIN2A,HMGCR,ITGB1,KIT,KRAS,MEIS2,NF1,RGS14,NPTN,FOXB1,RIC8A,TAFA2 |
| 12_Member | GO Biological Processes | GO:0007613 | memory | -4.172426098 | -2.947 | 27/117 | 793,1268,1385,1812,2475,2903,3643,3776,4803,5649,5728,6310,6326,6722,6860,7436,10636,22849,22986,23236,23373,23394,25769,54207,57338,338811,342371 | CALB1,CNR1,CREB1,DRD1,MTOR,GRIN2A,INSR,KCNK2,NGF,RELN,PTEN,ATXN1,SCN2A,SRF,SYT4,VLDLR,RGS14,CPEB3,SORCS3,PLCB1,CRTC1,ADNP,SLC24A2,KCNK10,JPH3,TAFA2,ATXN1L |
| 12_Member | GO Biological Processes | GO:0009314 | response to radiation | -3.48554703 | -2.359 | 71/449 | 351,801,1281,1385,1387,1812,1843,1874,1956,1958,1965,2033,2138,2332,2353,2475,2625,2776,2782,2885,2903,3156,3157,3198,3398,3688,3725,3746,3747,3815,3845,4212,4216,4734,4763,4986,5295,5501,5599,5796,6785,7412,7528,8491,8493,8533,9397,9575,10135,10276,10277,10636,10714,23373,23411,23596,27020,27023,51455,51761,54617,55031,57282,57646,60626,64326,94241,123879,150094,167153,338811 | APP,CALM1,COL3A1,CREB1,CREBBP,DRD1,DUSP1,E2F4,EGFR,EGR1,EIF2S1,EP300,EYA1,FMR1,FOS,MTOR,GATA3,GNAQ,GNB1,GRB2,GRIN2A,HMGCR,HMGCS1,HOXA1,ID2,ITGB1,JUN,KCNC1,KCNC2,KIT,KRAS,MEIS2,MAP3K4,NEDD4,NF1,OPRK1,PIK3R1,PPP1CC,MAPK8,PTPRK,ELOVL4,VCAM1,YY1,MAP4K3,PPM1D,COPS3,NMT2,CLOCK,NAMPT,NET1,UBE4B,RGS14,POLD3,CRTC1,SIRT1,OPN3,NPTN,FOXB1,REV1,ATP8A2,INO80,USP47,SLC4A10,USP28,RIC8A,COP1,TP53INP1,DCUN1D3,SIK1,TENT2,TAFA2 |
| 13_Summary | GO Cellular Components | GO:0005912 | adherens junction | -26.61949685 | -23.837 | 147/549 | 60,102,118,182,324,357,375,387,390,488,659,667,858,960,998,1006,1007,1009,1213,1499,1501,1656,1739,1946,1956,1982,2017,2043,2185,2195,2261,2274,2923,3190,3482,3660,3688,3696,3717,3845,3984,4008,4026,4082,4301,4478,4627,4643,4659,4810,4851,5034,5170,5239,5501,5576,5594,5604,5756,5782,5796,5797,5829,5898,5962,6730,6840,7082,7430,7529,7531,7532,7534,8321,8470,8500,8502,8573,8650,8675,8829,8936,9124,9223,9231,9267,9414,9448,9590,9815,10092,10109,10146,10152,10395,10397,10458,10580,10672,10787,10818,10890,10971,10982,11252,22800,22823,22919,23011,23114,23122,23150,23164,23332,23418,23603,23767,23768,26986,28514,29091,29119,29766,29843,30011,30845,54806,55327,55691,55740,55827,55966,57608,57619,58480,60312,64398,79038,79834,81873,83478,83660,83872,84978,117583,129446,171024,2683,1756,55914 | ACTB,ADAM10,ADD1,JAG1,APC,SHROOM2,ARF1,RHOA,RND3,ATP2A2,BMPR2,DST,CAV2,CD44,CDC42,CDH8,CDH9,CDH11,CLTC,CTNNB1,CTNND2,DDX6,DLG1,EFNA5,EGFR,EIF4G2,CTTN,EPHA4,PTK2B,FAT1,FGFR3,FHL2,PDIA3,HNRNPK,IGF2R,IRF2,ITGB1,ITGB8,JAK2,KRAS,LIMK1,LMO7,LPP,MARCKS,AFDN,MSN,MYH9,MYO1E,PPP1R12A,NHS,NOTCH1,P4HB,PDPK1,PGM5,PPP1CC,PRKAR2A,MAPK1,MAP2K1,TWF1,PTPN12,PTPRK,PTPRM,PXN,RALA,RDX,SRP68,SVIL,TJP1,EZR,YWHAB,YWHAE,YWHAG,YWHAZ,FZD1,SORBS2,PPFIA1,PKP4,CASK,NUMB,STX16,NRP1,WASF1,PDLIM1,MAGI1,DLG5,CYTH1,TJP2,MAP4K4,AKAP12,GIT2,ARPC5,ARPC2,G3BP1,ABI2,DLC1,NDRG1,BAIAP2,SORBS1,GNA13,NCKAP1,FRS2,RAB10,YWHAQ,MAPRE2,PACSIN2,RRAS2,MTF2,MAPRE1,RAB21,NFASC,CLASP2,FRMD4B,MPRIP,CLASP1,CRB1,CORO1C,FLRT3,FLRT2,PABPC1,DLL1,STXBP6,CTNNA3,TMOD3,SENP1,SH3KBP1,EHD3,AHI1,LIN7C,FRMD4A,ENAH,DCAF6,AJAP1,JCAD,SHROOM3,RHOU,AFAP1,MPP5,ZFYVE21,PEAK1,ARPC5L,ARHGAP24,TLN2,HMCN1,FRMD5,PARD3B,XIRP2,SYNPO2,B4GALT1,DMD,ERBIN |
| 13_Member | GO Cellular Components | GO:0005912 | adherens junction | -26.61949685 | -23.837 | 147/549 | 60,102,118,182,324,357,375,387,390,488,659,667,858,960,998,1006,1007,1009,1213,1499,1501,1656,1739,1946,1956,1982,2017,2043,2185,2195,2261,2274,2923,3190,3482,3660,3688,3696,3717,3845,3984,4008,4026,4082,4301,4478,4627,4643,4659,4810,4851,5034,5170,5239,5501,5576,5594,5604,5756,5782,5796,5797,5829,5898,5962,6730,6840,7082,7430,7529,7531,7532,7534,8321,8470,8500,8502,8573,8650,8675,8829,8936,9124,9223,9231,9267,9414,9448,9590,9815,10092,10109,10146,10152,10395,10397,10458,10580,10672,10787,10818,10890,10971,10982,11252,22800,22823,22919,23011,23114,23122,23150,23164,23332,23418,23603,23767,23768,26986,28514,29091,29119,29766,29843,30011,30845,54806,55327,55691,55740,55827,55966,57608,57619,58480,60312,64398,79038,79834,81873,83478,83660,83872,84978,117583,129446,171024 | ACTB,ADAM10,ADD1,JAG1,APC,SHROOM2,ARF1,RHOA,RND3,ATP2A2,BMPR2,DST,CAV2,CD44,CDC42,CDH8,CDH9,CDH11,CLTC,CTNNB1,CTNND2,DDX6,DLG1,EFNA5,EGFR,EIF4G2,CTTN,EPHA4,PTK2B,FAT1,FGFR3,FHL2,PDIA3,HNRNPK,IGF2R,IRF2,ITGB1,ITGB8,JAK2,KRAS,LIMK1,LMO7,LPP,MARCKS,AFDN,MSN,MYH9,MYO1E,PPP1R12A,NHS,NOTCH1,P4HB,PDPK1,PGM5,PPP1CC,PRKAR2A,MAPK1,MAP2K1,TWF1,PTPN12,PTPRK,PTPRM,PXN,RALA,RDX,SRP68,SVIL,TJP1,EZR,YWHAB,YWHAE,YWHAG,YWHAZ,FZD1,SORBS2,PPFIA1,PKP4,CASK,NUMB,STX16,NRP1,WASF1,PDLIM1,MAGI1,DLG5,CYTH1,TJP2,MAP4K4,AKAP12,GIT2,ARPC5,ARPC2,G3BP1,ABI2,DLC1,NDRG1,BAIAP2,SORBS1,GNA13,NCKAP1,FRS2,RAB10,YWHAQ,MAPRE2,PACSIN2,RRAS2,MTF2,MAPRE1,RAB21,NFASC,CLASP2,FRMD4B,MPRIP,CLASP1,CRB1,CORO1C,FLRT3,FLRT2,PABPC1,DLL1,STXBP6,CTNNA3,TMOD3,SENP1,SH3KBP1,EHD3,AHI1,LIN7C,FRMD4A,ENAH,DCAF6,AJAP1,JCAD,SHROOM3,RHOU,AFAP1,MPP5,ZFYVE21,PEAK1,ARPC5L,ARHGAP24,TLN2,HMCN1,FRMD5,PARD3B,XIRP2,SYNPO2 |
| 13_Member | GO Cellular Components | GO:0070161 | anchoring junction | -25.7264288 | -23.048 | 148/565 | 60,102,118,182,324,357,375,387,390,488,659,667,858,960,998,1006,1007,1009,1213,1499,1501,1656,1739,1946,1956,1982,2017,2043,2185,2195,2261,2274,2683,2923,3190,3482,3660,3688,3696,3717,3845,3984,4008,4026,4082,4301,4478,4627,4643,4659,4810,4851,5034,5170,5239,5501,5576,5594,5604,5756,5782,5796,5797,5829,5898,5962,6730,6840,7082,7430,7529,7531,7532,7534,8321,8470,8500,8502,8573,8650,8675,8829,8936,9124,9223,9231,9267,9414,9448,9590,9815,10092,10109,10146,10152,10395,10397,10458,10580,10672,10787,10818,10890,10971,10982,11252,22800,22823,22919,23011,23114,23122,23150,23164,23332,23418,23603,23767,23768,26986,28514,29091,29119,29766,29843,30011,30845,54806,55327,55691,55740,55827,55966,57608,57619,58480,60312,64398,79038,79834,81873,83478,83660,83872,84978,117583,129446,171024 | ACTB,ADAM10,ADD1,JAG1,APC,SHROOM2,ARF1,RHOA,RND3,ATP2A2,BMPR2,DST,CAV2,CD44,CDC42,CDH8,CDH9,CDH11,CLTC,CTNNB1,CTNND2,DDX6,DLG1,EFNA5,EGFR,EIF4G2,CTTN,EPHA4,PTK2B,FAT1,FGFR3,FHL2,B4GALT1,PDIA3,HNRNPK,IGF2R,IRF2,ITGB1,ITGB8,JAK2,KRAS,LIMK1,LMO7,LPP,MARCKS,AFDN,MSN,MYH9,MYO1E,PPP1R12A,NHS,NOTCH1,P4HB,PDPK1,PGM5,PPP1CC,PRKAR2A,MAPK1,MAP2K1,TWF1,PTPN12,PTPRK,PTPRM,PXN,RALA,RDX,SRP68,SVIL,TJP1,EZR,YWHAB,YWHAE,YWHAG,YWHAZ,FZD1,SORBS2,PPFIA1,PKP4,CASK,NUMB,STX16,NRP1,WASF1,PDLIM1,MAGI1,DLG5,CYTH1,TJP2,MAP4K4,AKAP12,GIT2,ARPC5,ARPC2,G3BP1,ABI2,DLC1,NDRG1,BAIAP2,SORBS1,GNA13,NCKAP1,FRS2,RAB10,YWHAQ,MAPRE2,PACSIN2,RRAS2,MTF2,MAPRE1,RAB21,NFASC,CLASP2,FRMD4B,MPRIP,CLASP1,CRB1,CORO1C,FLRT3,FLRT2,PABPC1,DLL1,STXBP6,CTNNA3,TMOD3,SENP1,SH3KBP1,EHD3,AHI1,LIN7C,FRMD4A,ENAH,DCAF6,AJAP1,JCAD,SHROOM3,RHOU,AFAP1,MPP5,ZFYVE21,PEAK1,ARPC5L,ARHGAP24,TLN2,HMCN1,FRMD5,PARD3B,XIRP2,SYNPO2 |
| 13_Member | GO Cellular Components | GO:0030055 | cell-substrate junction | -16.73401649 | -14.497 | 104/415 | 60,102,118,375,387,390,667,858,960,998,1213,1499,1756,1956,2017,2185,2195,2261,2274,2923,3190,3482,3660,3688,3696,3717,3845,3984,4008,4026,4082,4478,4627,4659,4810,5034,5170,5239,5501,5576,5594,5604,5756,5782,5829,5898,5962,6730,6840,7430,7529,7531,7532,7534,8321,8470,8500,8573,8650,8675,8829,8936,9124,9448,9590,9815,10092,10109,10146,10395,10580,10672,10787,10890,10971,10982,11252,22800,22823,22919,23011,23114,23122,23164,23332,23603,23767,23768,26986,29843,30011,30845,55740,55827,55914,58480,60312,79038,79834,81873,83478,83660,129446,171024 | ACTB,ADAM10,ADD1,ARF1,RHOA,RND3,DST,CAV2,CD44,CDC42,CLTC,CTNNB1,DMD,EGFR,CTTN,PTK2B,FAT1,FGFR3,FHL2,PDIA3,HNRNPK,IGF2R,IRF2,ITGB1,ITGB8,JAK2,KRAS,LIMK1,LMO7,LPP,MARCKS,MSN,MYH9,PPP1R12A,NHS,P4HB,PDPK1,PGM5,PPP1CC,PRKAR2A,MAPK1,MAP2K1,TWF1,PTPN12,PXN,RALA,RDX,SRP68,SVIL,EZR,YWHAB,YWHAE,YWHAG,YWHAZ,FZD1,SORBS2,PPFIA1,CASK,NUMB,STX16,NRP1,WASF1,PDLIM1,MAP4K4,AKAP12,GIT2,ARPC5,ARPC2,G3BP1,DLC1,SORBS1,GNA13,NCKAP1,RAB10,YWHAQ,MAPRE2,PACSIN2,RRAS2,MTF2,MAPRE1,RAB21,NFASC,CLASP2,MPRIP,CLASP1,CORO1C,FLRT3,FLRT2,PABPC1,SENP1,SH3KBP1,EHD3,ENAH,DCAF6,ERBIN,RHOU,AFAP1,ZFYVE21,PEAK1,ARPC5L,ARHGAP24,TLN2,XIRP2,SYNPO2 |
| 13_Member | GO Cellular Components | GO:0005925 | focal adhesion | -16.34649516 | -14.129 | 102/408 | 60,102,118,375,387,390,667,858,960,998,1213,1499,1956,2017,2185,2195,2261,2274,2923,3190,3482,3660,3688,3696,3717,3845,3984,4008,4026,4082,4478,4627,4659,4810,5034,5170,5239,5501,5576,5594,5604,5756,5782,5829,5898,5962,6730,6840,7430,7529,7531,7532,7534,8321,8470,8500,8573,8650,8675,8829,8936,9124,9448,9590,9815,10092,10109,10146,10395,10580,10672,10787,10890,10971,10982,11252,22800,22823,22919,23011,23114,23122,23164,23332,23603,23767,23768,26986,29843,30011,30845,55740,55827,58480,60312,79038,79834,81873,83478,83660,129446,171024 | ACTB,ADAM10,ADD1,ARF1,RHOA,RND3,DST,CAV2,CD44,CDC42,CLTC,CTNNB1,EGFR,CTTN,PTK2B,FAT1,FGFR3,FHL2,PDIA3,HNRNPK,IGF2R,IRF2,ITGB1,ITGB8,JAK2,KRAS,LIMK1,LMO7,LPP,MARCKS,MSN,MYH9,PPP1R12A,NHS,P4HB,PDPK1,PGM5,PPP1CC,PRKAR2A,MAPK1,MAP2K1,TWF1,PTPN12,PXN,RALA,RDX,SRP68,SVIL,EZR,YWHAB,YWHAE,YWHAG,YWHAZ,FZD1,SORBS2,PPFIA1,CASK,NUMB,STX16,NRP1,WASF1,PDLIM1,MAP4K4,AKAP12,GIT2,ARPC5,ARPC2,G3BP1,DLC1,SORBS1,GNA13,NCKAP1,RAB10,YWHAQ,MAPRE2,PACSIN2,RRAS2,MTF2,MAPRE1,RAB21,NFASC,CLASP2,MPRIP,CLASP1,CORO1C,FLRT3,FLRT2,PABPC1,SENP1,SH3KBP1,EHD3,ENAH,DCAF6,RHOU,AFAP1,ZFYVE21,PEAK1,ARPC5L,ARHGAP24,TLN2,XIRP2,SYNPO2 |
| 13_Member | GO Cellular Components | GO:0005924 | cell-substrate adherens junction | -16.11298682 | -13.912 | 102/411 | 60,102,118,375,387,390,667,858,960,998,1213,1499,1956,2017,2185,2195,2261,2274,2923,3190,3482,3660,3688,3696,3717,3845,3984,4008,4026,4082,4478,4627,4659,4810,5034,5170,5239,5501,5576,5594,5604,5756,5782,5829,5898,5962,6730,6840,7430,7529,7531,7532,7534,8321,8470,8500,8573,8650,8675,8829,8936,9124,9448,9590,9815,10092,10109,10146,10395,10580,10672,10787,10890,10971,10982,11252,22800,22823,22919,23011,23114,23122,23164,23332,23603,23767,23768,26986,29843,30011,30845,55740,55827,58480,60312,79038,79834,81873,83478,83660,129446,171024 | ACTB,ADAM10,ADD1,ARF1,RHOA,RND3,DST,CAV2,CD44,CDC42,CLTC,CTNNB1,EGFR,CTTN,PTK2B,FAT1,FGFR3,FHL2,PDIA3,HNRNPK,IGF2R,IRF2,ITGB1,ITGB8,JAK2,KRAS,LIMK1,LMO7,LPP,MARCKS,MSN,MYH9,PPP1R12A,NHS,P4HB,PDPK1,PGM5,PPP1CC,PRKAR2A,MAPK1,MAP2K1,TWF1,PTPN12,PXN,RALA,RDX,SRP68,SVIL,EZR,YWHAB,YWHAE,YWHAG,YWHAZ,FZD1,SORBS2,PPFIA1,CASK,NUMB,STX16,NRP1,WASF1,PDLIM1,MAP4K4,AKAP12,GIT2,ARPC5,ARPC2,G3BP1,DLC1,SORBS1,GNA13,NCKAP1,RAB10,YWHAQ,MAPRE2,PACSIN2,RRAS2,MTF2,MAPRE1,RAB21,NFASC,CLASP2,MPRIP,CLASP1,CORO1C,FLRT3,FLRT2,PABPC1,SENP1,SH3KBP1,EHD3,ENAH,DCAF6,RHOU,AFAP1,ZFYVE21,PEAK1,ARPC5L,ARHGAP24,TLN2,XIRP2,SYNPO2 |
| 14_Summary | GO Biological Processes | GO:0061061 | muscle structure development | -26.50260013 | -23.732 | 168/674 | 25,90,182,287,387,406,463,488,607,650,657,677,688,694,858,905,998,1073,1106,1271,1281,1385,1482,1499,1634,1655,1756,1901,1958,2033,2066,2115,2255,2263,2294,2353,2475,2626,3084,3131,3156,3192,3344,3688,3720,3756,3776,3815,3845,4154,4205,4208,4223,4534,4627,4628,4638,4762,4763,4773,4775,4851,4856,5079,5155,5156,5239,5292,5469,5530,5573,5594,5629,5725,5728,5978,5999,6095,6256,6416,6444,6497,6546,6664,6722,6840,6909,6929,6934,6935,6938,7042,7046,7048,7161,7168,7248,7291,7402,7494,7528,7832,7881,8087,8289,8321,8470,8841,8910,9043,9124,9254,9318,9444,9456,9472,9474,9547,9734,9794,10277,10521,10529,10818,10939,11146,23236,23345,23411,23414,23493,25842,25928,26057,26281,27063,27250,27302,28514,29766,30812,51460,51804,54567,55544,55553,55636,55796,56261,56704,57493,57496,64091,80314,84159,84665,85407,93166,93986,129446,136319,137868,140578,144165,150094,221662,283078,348093,163,639,655,659,775,1027,1289,1363,1386,1399,1848,1889,1956,2138,2200,2625,2736,2737,3037,3398,3491,3643,3725,4040,4052,4086,4091,4853,5125,5308,5604,5727,6091,6092,6093,6299,6491,6654,6788,7403,7412,7547,8313,8828,8829,9353,9475,9821,10052,10087,10252,10395,10427,10959,23054,23118,23767,23768,29072,54806,57142,57167,57178,57534,63917,79602,79977,80000,80114,130497,161742,196528,1739,10891,367,1303,2252,2261,6781,7337,10253,23286,26524,54796,55512,55841,64856,80014,81578,284,3717 | ABL1,ACVR1,JAG1,ANK2,RHOA,ARNTL,ZFHX3,ATP2A2,BCL9,BMP2,BMPR1A,ZFP36L1,KLF5,BTG1,CAV2,CCNT2,CDC42,CFL2,CHD2,CNTFR,COL3A1,CREB1,NKX2-5,CTNNB1,DCN,DDX5,DMD,S1PR1,EGR1,EP300,ERBB4,ETV1,FGF10,FGFR2,FOXF1,FOS,MTOR,GATA4,NRG1,HLF,HMGCR,HNRNPU,FOXN2,ITGB1,JARID2,KCNH1,KCNK2,KIT,KRAS,MBNL1,MEF2A,MEF2C,MEOX2,MTM1,MYH9,MYH10,MYLK,NEUROG1,NF1,NFATC2,NFATC3,NOTCH1,CCN3,PAX5,PDGFB,PDGFRA,PGM5,PIM1,MED1,PPP3CA,PRKAR1A,MAPK1,PROX1,PTBP1,PTEN,REST,RGS4,RORA,RXRA,MAP2K4,SGCD,SKI,SLC8A1,SOX11,SRF,SVIL,TBX2,TCF3,TCF7L2,ZEB1,TCF12,TGFB2,TGFBR1,TGFBR2,TP73,TPM1,TSC1,TWIST1,UTRN,XBP1,YY1,BTG2,KCNAB1,FXR1,ARID1A,FZD1,SORBS2,HDAC3,SGCE,SPAG9,PDLIM1,CACNA2D2,COPS2,QKI,HOMER1,AKAP6,ATG5,CXCL14,HDAC9,MAML1,UBE4B,DDX17,NEBL,FRS2,AFG3L2,GLMN,PLCB1,SYNE1,SIRT1,ZFPM2,HEY2,ASF1A,SOSTDC1,ANKRD17,FGF20,ANKRD1,PDCD4,BMP10,DLL1,TMOD3,SOX8,SFMBT1,SIX4,DLL4,RBM38,SOX6,CHD7,MBNL3,GPCPD1,JPH1,HEG1,MRTFB,POPDC2,EPC1,ARID5B,MYPN,NKD1,PRDM6,FOXP2,XIRP2,MTPN,SGCZ,CHODL,PRICKLE1,SIK1,RBM24,MKX,RBPMS2,AP2B1,PRDM1,BMP7,BMPR2,CACNA1C,CDKN1B,COL5A1,CPE,ATF2,CRKL,DUSP6,ECE1,EGFR,EYA1,FBN1,GATA3,GLI2,GLI3,HAS2,ID2,CCN1,INSR,JUN,LRP6,LTBP1,SMAD1,SMAD6,NOTCH2,PCSK5,PITX2,MAP2K1,PTCH1,ROBO1,ROBO2,ROCK1,SALL1,STIL,SOS1,STK3,KDM6A,VCAM1,ZIC3,AXIN2,NRP2,NRP1,SLIT2,ROCK2,RB1CC1,GJC1,CERT1,SPRY1,DLC1,SEC24B,TMED2,NCOA6,TAB2,FLRT3,FLRT2,SETD2,AHI1,RTN4,SALL4,ZMIZ1,MIB1,GALNT11,ADIPOR2,GRHL2,GREB1L,BICC1,OSR1,SPRED1,ARID2,DLG1,PPARGC1A,AR,COL12A1,FGF7,FGFR3,STC1,UBE3A,SPRY2,WWC1,LATS2,BNC2,SMPD3,WWC3,VWA1,WWC2,COL21A1,ANGPT1,JAK2 |
| 14_Member | GO Biological Processes | GO:0061061 | muscle structure development | -26.50260013 | -23.732 | 168/674 | 25,90,182,287,387,406,463,488,607,650,657,677,688,694,858,905,998,1073,1106,1271,1281,1385,1482,1499,1634,1655,1756,1901,1958,2033,2066,2115,2255,2263,2294,2353,2475,2626,3084,3131,3156,3192,3344,3688,3720,3756,3776,3815,3845,4154,4205,4208,4223,4534,4627,4628,4638,4762,4763,4773,4775,4851,4856,5079,5155,5156,5239,5292,5469,5530,5573,5594,5629,5725,5728,5978,5999,6095,6256,6416,6444,6497,6546,6664,6722,6840,6909,6929,6934,6935,6938,7042,7046,7048,7161,7168,7248,7291,7402,7494,7528,7832,7881,8087,8289,8321,8470,8841,8910,9043,9124,9254,9318,9444,9456,9472,9474,9547,9734,9794,10277,10521,10529,10818,10939,11146,23236,23345,23411,23414,23493,25842,25928,26057,26281,27063,27250,27302,28514,29766,30812,51460,51804,54567,55544,55553,55636,55796,56261,56704,57493,57496,64091,80314,84159,84665,85407,93166,93986,129446,136319,137868,140578,144165,150094,221662,283078,348093 | ABL1,ACVR1,JAG1,ANK2,RHOA,ARNTL,ZFHX3,ATP2A2,BCL9,BMP2,BMPR1A,ZFP36L1,KLF5,BTG1,CAV2,CCNT2,CDC42,CFL2,CHD2,CNTFR,COL3A1,CREB1,NKX2-5,CTNNB1,DCN,DDX5,DMD,S1PR1,EGR1,EP300,ERBB4,ETV1,FGF10,FGFR2,FOXF1,FOS,MTOR,GATA4,NRG1,HLF,HMGCR,HNRNPU,FOXN2,ITGB1,JARID2,KCNH1,KCNK2,KIT,KRAS,MBNL1,MEF2A,MEF2C,MEOX2,MTM1,MYH9,MYH10,MYLK,NEUROG1,NF1,NFATC2,NFATC3,NOTCH1,CCN3,PAX5,PDGFB,PDGFRA,PGM5,PIM1,MED1,PPP3CA,PRKAR1A,MAPK1,PROX1,PTBP1,PTEN,REST,RGS4,RORA,RXRA,MAP2K4,SGCD,SKI,SLC8A1,SOX11,SRF,SVIL,TBX2,TCF3,TCF7L2,ZEB1,TCF12,TGFB2,TGFBR1,TGFBR2,TP73,TPM1,TSC1,TWIST1,UTRN,XBP1,YY1,BTG2,KCNAB1,FXR1,ARID1A,FZD1,SORBS2,HDAC3,SGCE,SPAG9,PDLIM1,CACNA2D2,COPS2,QKI,HOMER1,AKAP6,ATG5,CXCL14,HDAC9,MAML1,UBE4B,DDX17,NEBL,FRS2,AFG3L2,GLMN,PLCB1,SYNE1,SIRT1,ZFPM2,HEY2,ASF1A,SOSTDC1,ANKRD17,FGF20,ANKRD1,PDCD4,BMP10,DLL1,TMOD3,SOX8,SFMBT1,SIX4,DLL4,RBM38,SOX6,CHD7,MBNL3,GPCPD1,JPH1,HEG1,MRTFB,POPDC2,EPC1,ARID5B,MYPN,NKD1,PRDM6,FOXP2,XIRP2,MTPN,SGCZ,CHODL,PRICKLE1,SIK1,RBM24,MKX,RBPMS2 |
| 14_Member | GO Biological Processes | GO:0007507 | heart development | -26.2549368 | -23.506 | 153/588 | 90,163,182,287,639,650,655,657,659,677,775,1027,1281,1289,1363,1385,1386,1399,1482,1499,1848,1889,1901,1956,2033,2066,2138,2200,2263,2294,2475,2625,2626,2736,2737,3037,3084,3192,3398,3491,3643,3688,3720,3725,3776,4040,4052,4086,4091,4205,4208,4628,4763,4851,4853,5125,5155,5156,5292,5308,5469,5573,5594,5604,5629,5727,5728,5978,5999,6091,6092,6093,6256,6299,6416,6444,6491,6546,6654,6664,6722,6788,6909,7042,7046,7048,7161,7168,7248,7291,7403,7412,7528,7547,7881,8289,8313,8321,8470,8828,8829,9124,9353,9472,9474,9475,9734,9794,9821,10052,10087,10252,10277,10395,10427,10529,10818,10959,23054,23118,23414,23493,23767,23768,26281,27063,27250,27302,28514,29072,54567,54806,55553,55636,57142,57167,57178,57493,57534,63917,64091,79602,79977,80000,80114,129446,130497,137868,144165,150094,161742,196528,221662 | ACVR1,AP2B1,JAG1,ANK2,PRDM1,BMP2,BMP7,BMPR1A,BMPR2,ZFP36L1,CACNA1C,CDKN1B,COL3A1,COL5A1,CPE,CREB1,ATF2,CRKL,NKX2-5,CTNNB1,DUSP6,ECE1,S1PR1,EGFR,EP300,ERBB4,EYA1,FBN1,FGFR2,FOXF1,MTOR,GATA3,GATA4,GLI2,GLI3,HAS2,NRG1,HNRNPU,ID2,CCN1,INSR,ITGB1,JARID2,JUN,KCNK2,LRP6,LTBP1,SMAD1,SMAD6,MEF2A,MEF2C,MYH10,NF1,NOTCH1,NOTCH2,PCSK5,PDGFB,PDGFRA,PIM1,PITX2,MED1,PRKAR1A,MAPK1,MAP2K1,PROX1,PTCH1,PTEN,REST,RGS4,ROBO1,ROBO2,ROCK1,RXRA,SALL1,MAP2K4,SGCD,STIL,SLC8A1,SOS1,SOX11,SRF,STK3,TBX2,TGFB2,TGFBR1,TGFBR2,TP73,TPM1,TSC1,TWIST1,KDM6A,VCAM1,YY1,ZIC3,KCNAB1,ARID1A,AXIN2,FZD1,SORBS2,NRP2,NRP1,PDLIM1,SLIT2,AKAP6,ATG5,ROCK2,HDAC9,MAML1,RB1CC1,GJC1,CERT1,SPRY1,UBE4B,DLC1,SEC24B,NEBL,FRS2,TMED2,NCOA6,TAB2,ZFPM2,HEY2,FLRT3,FLRT2,FGF20,ANKRD1,PDCD4,BMP10,DLL1,SETD2,DLL4,AHI1,SOX6,CHD7,RTN4,SALL4,ZMIZ1,HEG1,MIB1,GALNT11,POPDC2,ADIPOR2,GRHL2,GREB1L,BICC1,XIRP2,OSR1,SGCZ,PRICKLE1,SIK1,SPRED1,ARID2,RBM24 |
| 14_Member | GO Biological Processes | GO:0060537 | muscle tissue development | -21.74158224 | -19.283 | 113/409 | 90,387,406,607,650,655,657,659,688,858,905,1073,1281,1385,1482,1499,1634,1655,1739,1901,1958,2033,2066,2138,2263,2353,2475,2626,3084,3131,3156,3192,3344,3398,3688,3720,3776,4086,4205,4208,4223,4534,4628,4638,4763,4851,5079,5156,5239,5292,5469,5530,5573,5594,5629,5728,5999,6256,6416,6444,6497,6546,6664,6722,6840,6909,7042,7046,7048,7161,7168,7248,7291,7528,7832,7881,8289,8470,9318,9456,9472,9474,9734,9794,10052,10277,10521,10529,10818,10891,23414,23493,26281,27063,27302,28514,30812,51804,54567,55553,55636,56261,57493,57496,64091,93986,129446,130497,136319,137868,150094,196528,221662 | ACVR1,RHOA,ARNTL,BCL9,BMP2,BMP7,BMPR1A,BMPR2,KLF5,CAV2,CCNT2,CFL2,COL3A1,CREB1,NKX2-5,CTNNB1,DCN,DDX5,DLG1,S1PR1,EGR1,EP300,ERBB4,EYA1,FGFR2,FOS,MTOR,GATA4,NRG1,HLF,HMGCR,HNRNPU,FOXN2,ID2,ITGB1,JARID2,KCNK2,SMAD1,MEF2A,MEF2C,MEOX2,MTM1,MYH10,MYLK,NF1,NOTCH1,PAX5,PDGFRA,PGM5,PIM1,MED1,PPP3CA,PRKAR1A,MAPK1,PROX1,PTEN,RGS4,RXRA,MAP2K4,SGCD,SKI,SLC8A1,SOX11,SRF,SVIL,TBX2,TGFB2,TGFBR1,TGFBR2,TP73,TPM1,TSC1,TWIST1,YY1,BTG2,KCNAB1,ARID1A,SORBS2,COPS2,HOMER1,AKAP6,ATG5,HDAC9,MAML1,GJC1,UBE4B,DDX17,NEBL,FRS2,PPARGC1A,ZFPM2,HEY2,FGF20,ANKRD1,BMP10,DLL1,SOX8,SIX4,DLL4,SOX6,CHD7,GPCPD1,HEG1,MRTFB,POPDC2,FOXP2,XIRP2,OSR1,MTPN,SGCZ,SIK1,ARID2,RBM24 |
| 14_Member | GO Biological Processes | GO:0014706 | striated muscle tissue development | -20.79303065 | -18.372 | 108/391 | 90,387,406,607,650,655,657,659,688,858,905,1073,1385,1482,1499,1634,1655,1901,1958,2033,2066,2138,2263,2353,2475,2626,3084,3131,3156,3192,3344,3398,3688,3720,3776,4086,4205,4208,4223,4534,4628,4763,4851,5079,5156,5239,5292,5469,5530,5573,5594,5629,5728,5999,6256,6416,6444,6497,6546,6664,6722,6840,6909,7042,7046,7048,7161,7168,7248,7291,7528,7832,7881,8289,8470,9318,9456,9472,9474,9734,9794,10052,10277,10521,10529,10818,23414,23493,26281,27063,27302,28514,30812,51804,54567,55553,55636,56261,57493,57496,64091,93986,129446,136319,137868,150094,196528,221662 | ACVR1,RHOA,ARNTL,BCL9,BMP2,BMP7,BMPR1A,BMPR2,KLF5,CAV2,CCNT2,CFL2,CREB1,NKX2-5,CTNNB1,DCN,DDX5,S1PR1,EGR1,EP300,ERBB4,EYA1,FGFR2,FOS,MTOR,GATA4,NRG1,HLF,HMGCR,HNRNPU,FOXN2,ID2,ITGB1,JARID2,KCNK2,SMAD1,MEF2A,MEF2C,MEOX2,MTM1,MYH10,NF1,NOTCH1,PAX5,PDGFRA,PGM5,PIM1,MED1,PPP3CA,PRKAR1A,MAPK1,PROX1,PTEN,RGS4,RXRA,MAP2K4,SGCD,SKI,SLC8A1,SOX11,SRF,SVIL,TBX2,TGFB2,TGFBR1,TGFBR2,TP73,TPM1,TSC1,TWIST1,YY1,BTG2,KCNAB1,ARID1A,SORBS2,COPS2,HOMER1,AKAP6,ATG5,HDAC9,MAML1,GJC1,UBE4B,DDX17,NEBL,FRS2,ZFPM2,HEY2,FGF20,ANKRD1,BMP10,DLL1,SOX8,SIX4,DLL4,SOX6,CHD7,GPCPD1,HEG1,MRTFB,POPDC2,FOXP2,XIRP2,MTPN,SGCZ,SIK1,ARID2,RBM24 |
| 14_Member | GO Biological Processes | GO:0003007 | heart morphogenesis | -16.87036058 | -14.624 | 77/260 | 90,182,650,655,657,659,1289,1363,1386,1399,1482,1499,1901,2138,2263,2294,2475,2625,2626,3037,3084,3398,3491,3643,3725,4091,4208,4851,4853,5292,5308,5469,5629,5727,6091,6092,6256,6491,6654,6664,6722,6909,7042,7046,7048,7168,7291,7403,7547,8313,8321,8828,8829,9353,10087,10252,10277,10395,10427,10959,23414,23493,23768,27063,27250,27302,28514,54567,54806,55636,57142,57178,57493,57534,79977,129446,196528 | ACVR1,JAG1,BMP2,BMP7,BMPR1A,BMPR2,COL5A1,CPE,ATF2,CRKL,NKX2-5,CTNNB1,S1PR1,EYA1,FGFR2,FOXF1,MTOR,GATA3,GATA4,HAS2,NRG1,ID2,CCN1,INSR,JUN,SMAD6,MEF2C,NOTCH1,NOTCH2,PIM1,PITX2,MED1,PROX1,PTCH1,ROBO1,ROBO2,RXRA,STIL,SOS1,SOX11,SRF,TBX2,TGFB2,TGFBR1,TGFBR2,TPM1,TWIST1,KDM6A,ZIC3,AXIN2,FZD1,NRP2,NRP1,SLIT2,CERT1,SPRY1,UBE4B,DLC1,SEC24B,TMED2,ZFPM2,HEY2,FLRT2,ANKRD1,PDCD4,BMP10,DLL1,DLL4,AHI1,CHD7,RTN4,ZMIZ1,HEG1,MIB1,GRHL2,XIRP2,ARID2 |
| 14_Member | GO Biological Processes | GO:0007517 | muscle organ development | -16.19047162 | -13.986 | 102/410 | 387,406,463,607,650,657,688,858,905,1073,1106,1271,1281,1385,1482,1499,1634,1655,1756,1901,1958,2033,2066,2115,2263,2353,2475,3084,3131,3156,3344,3720,3776,4205,4208,4223,4534,4638,4762,4763,4851,5079,5292,5469,5530,5594,5629,5728,5999,6256,6444,6497,6664,6840,6909,6938,7042,7046,7048,7161,7168,7291,7402,7494,7528,7832,7881,8087,8321,8910,9318,9456,9472,9734,10277,10521,10818,23411,23414,23493,26281,27063,27302,28514,30812,51460,51804,54567,55553,55636,56261,56704,57493,57496,64091,84159,93986,129446,136319,140578,221662,283078 | RHOA,ARNTL,ZFHX3,BCL9,BMP2,BMPR1A,KLF5,CAV2,CCNT2,CFL2,CHD2,CNTFR,COL3A1,CREB1,NKX2-5,CTNNB1,DCN,DDX5,DMD,S1PR1,EGR1,EP300,ERBB4,ETV1,FGFR2,FOS,MTOR,NRG1,HLF,HMGCR,FOXN2,JARID2,KCNK2,MEF2A,MEF2C,MEOX2,MTM1,MYLK,NEUROG1,NF1,NOTCH1,PAX5,PIM1,MED1,PPP3CA,MAPK1,PROX1,PTEN,RGS4,RXRA,SGCD,SKI,SOX11,SVIL,TBX2,TCF12,TGFB2,TGFBR1,TGFBR2,TP73,TPM1,TWIST1,UTRN,XBP1,YY1,BTG2,KCNAB1,FXR1,FZD1,SGCE,COPS2,HOMER1,AKAP6,HDAC9,UBE4B,DDX17,FRS2,SIRT1,ZFPM2,HEY2,FGF20,ANKRD1,BMP10,DLL1,SOX8,SFMBT1,SIX4,DLL4,SOX6,CHD7,GPCPD1,JPH1,HEG1,MRTFB,POPDC2,ARID5B,FOXP2,XIRP2,MTPN,CHODL,RBM24,MKX |
| 14_Member | GO Biological Processes | GO:0048738 | cardiac muscle tissue development | -13.97174007 | -11.916 | 67/234 | 90,650,655,657,659,1385,1482,1901,2066,2263,2475,2626,3084,3192,3398,3688,3720,3776,4086,4205,4208,4628,4851,5156,5292,5469,5573,5594,5629,5728,5999,6256,6416,6444,6546,6722,6909,7042,7046,7048,7161,7168,7248,7528,8289,8470,9472,9474,9794,10052,10277,10529,10818,23414,23493,26281,27063,27302,28514,54567,55553,55636,57493,129446,137868,150094,196528 | ACVR1,BMP2,BMP7,BMPR1A,BMPR2,CREB1,NKX2-5,S1PR1,ERBB4,FGFR2,MTOR,GATA4,NRG1,HNRNPU,ID2,ITGB1,JARID2,KCNK2,SMAD1,MEF2A,MEF2C,MYH10,NOTCH1,PDGFRA,PIM1,MED1,PRKAR1A,MAPK1,PROX1,PTEN,RGS4,RXRA,MAP2K4,SGCD,SLC8A1,SRF,TBX2,TGFB2,TGFBR1,TGFBR2,TP73,TPM1,TSC1,YY1,ARID1A,SORBS2,AKAP6,ATG5,MAML1,GJC1,UBE4B,NEBL,FRS2,ZFPM2,HEY2,FGF20,ANKRD1,BMP10,DLL1,DLL4,SOX6,CHD7,HEG1,XIRP2,SGCZ,SIK1,ARID2 |
| 14_Member | GO Biological Processes | GO:0035265 | organ growth | -9.966706177 | -8.175 | 54/204 | 367,657,1303,1482,1848,1901,2066,2252,2255,2261,2263,2475,2626,3720,3776,4086,4208,4851,5292,5573,5594,5629,5728,5999,6256,6416,6781,6788,6909,7042,7046,7048,7161,7337,7528,8470,9254,9472,10253,23286,23414,23493,26281,26524,27302,28514,54796,55512,55841,57493,64856,80014,81578,196528 | AR,BMPR1A,COL12A1,NKX2-5,DUSP6,S1PR1,ERBB4,FGF7,FGF10,FGFR3,FGFR2,MTOR,GATA4,JARID2,KCNK2,SMAD1,MEF2C,NOTCH1,PIM1,PRKAR1A,MAPK1,PROX1,PTEN,RGS4,RXRA,MAP2K4,STC1,STK3,TBX2,TGFB2,TGFBR1,TGFBR2,TP73,UBE3A,YY1,SORBS2,CACNA2D2,AKAP6,SPRY2,WWC1,ZFPM2,HEY2,FGF20,LATS2,BMP10,DLL1,BNC2,SMPD3,WWC3,HEG1,VWA1,WWC2,COL21A1,ARID2 |
| 14_Member | GO Biological Processes | GO:0060419 | heart growth | -8.740737934 | -7.045 | 35/112 | 657,1482,1848,1901,2066,2263,2475,2626,3720,3776,4086,4208,4851,5292,5573,5594,5629,5728,5999,6256,6416,6909,7042,7046,7048,7161,7528,8470,9472,23414,23493,26281,27302,57493,196528 | BMPR1A,NKX2-5,DUSP6,S1PR1,ERBB4,FGFR2,MTOR,GATA4,JARID2,KCNK2,SMAD1,MEF2C,NOTCH1,PIM1,PRKAR1A,MAPK1,PROX1,PTEN,RGS4,RXRA,MAP2K4,TBX2,TGFB2,TGFBR1,TGFBR2,TP73,YY1,SORBS2,AKAP6,ZFPM2,HEY2,FGF20,BMP10,HEG1,ARID2 |
| 14_Member | GO Biological Processes | GO:0048634 | regulation of muscle organ development | -8.66267659 | -6.974 | 43/156 | 406,650,657,1385,1482,1499,1655,2066,2263,2475,3084,3156,3720,3776,4208,4534,4762,4851,5292,5594,5728,5999,6909,7046,7048,7161,7291,7528,9472,9734,10521,10818,23414,23493,26281,27302,28514,51460,51804,55553,57496,136319,221662 | ARNTL,BMP2,BMPR1A,CREB1,NKX2-5,CTNNB1,DDX5,ERBB4,FGFR2,MTOR,NRG1,HMGCR,JARID2,KCNK2,MEF2C,MTM1,NEUROG1,NOTCH1,PIM1,MAPK1,PTEN,RGS4,TBX2,TGFBR1,TGFBR2,TP73,TWIST1,YY1,AKAP6,HDAC9,DDX17,FRS2,ZFPM2,HEY2,FGF20,BMP10,DLL1,SFMBT1,SIX4,SOX6,MRTFB,MTPN,RBM24 |
| 14_Member | GO Biological Processes | GO:0055017 | cardiac muscle tissue growth | -8.351170916 | -6.696 | 33/105 | 657,1482,1901,2066,2263,2475,2626,3720,3776,4086,4208,4851,5292,5573,5594,5728,5999,6256,6416,6909,7042,7046,7048,7161,7528,8470,9472,23414,23493,26281,27302,57493,196528 | BMPR1A,NKX2-5,S1PR1,ERBB4,FGFR2,MTOR,GATA4,JARID2,KCNK2,SMAD1,MEF2C,NOTCH1,PIM1,PRKAR1A,MAPK1,PTEN,RGS4,RXRA,MAP2K4,TBX2,TGFB2,TGFBR1,TGFBR2,TP73,YY1,SORBS2,AKAP6,ZFPM2,HEY2,FGF20,BMP10,HEG1,ARID2 |
| 14_Member | GO Biological Processes | GO:1901861 | regulation of muscle tissue development | -8.236201898 | -6.590 | 42/155 | 406,650,657,1385,1482,1499,1655,2066,2263,2475,3084,3156,3720,3776,4208,4534,4851,5292,5594,5728,5999,6909,7046,7048,7161,7291,7528,9472,9734,10521,10818,10891,23414,23493,26281,27302,28514,51804,55553,57496,136319,221662 | ARNTL,BMP2,BMPR1A,CREB1,NKX2-5,CTNNB1,DDX5,ERBB4,FGFR2,MTOR,NRG1,HMGCR,JARID2,KCNK2,MEF2C,MTM1,NOTCH1,PIM1,MAPK1,PTEN,RGS4,TBX2,TGFBR1,TGFBR2,TP73,TWIST1,YY1,AKAP6,HDAC9,DDX17,FRS2,PPARGC1A,ZFPM2,HEY2,FGF20,BMP10,DLL1,SIX4,SOX6,MRTFB,MTPN,RBM24 |
| 14_Member | GO Biological Processes | GO:0016202 | regulation of striated muscle tissue development | -7.994720608 | -6.372 | 41/152 | 406,650,657,1385,1482,1499,1655,2066,2263,2475,3084,3156,3720,3776,4208,4534,4851,5292,5594,5728,5999,6909,7046,7048,7161,7291,7528,9472,9734,10521,10818,23414,23493,26281,27302,28514,51804,55553,57496,136319,221662 | ARNTL,BMP2,BMPR1A,CREB1,NKX2-5,CTNNB1,DDX5,ERBB4,FGFR2,MTOR,NRG1,HMGCR,JARID2,KCNK2,MEF2C,MTM1,NOTCH1,PIM1,MAPK1,PTEN,RGS4,TBX2,TGFBR1,TGFBR2,TP73,TWIST1,YY1,AKAP6,HDAC9,DDX17,FRS2,ZFPM2,HEY2,FGF20,BMP10,DLL1,SIX4,SOX6,MRTFB,MTPN,RBM24 |
| 14_Member | GO Biological Processes | GO:0060038 | cardiac muscle cell proliferation | -7.479742132 | -5.901 | 24/67 | 657,1482,2066,2263,3720,3776,4086,4208,4851,5292,5573,5594,5728,6256,6909,7042,7046,7048,7161,23414,23493,26281,27302,196528 | BMPR1A,NKX2-5,ERBB4,FGFR2,JARID2,KCNK2,SMAD1,MEF2C,NOTCH1,PIM1,PRKAR1A,MAPK1,PTEN,RXRA,TBX2,TGFB2,TGFBR1,TGFBR2,TP73,ZFPM2,HEY2,FGF20,BMP10,ARID2 |
| 14_Member | GO Biological Processes | GO:0014855 | striated muscle cell proliferation | -6.930437735 | -5.401 | 26/81 | 284,657,1482,2066,2263,3717,3720,3776,4086,4208,4851,5292,5573,5594,5728,6256,6909,7042,7046,7048,7161,23414,23493,26281,27302,196528 | ANGPT1,BMPR1A,NKX2-5,ERBB4,FGFR2,JAK2,JARID2,KCNK2,SMAD1,MEF2C,NOTCH1,PIM1,PRKAR1A,MAPK1,PTEN,RXRA,TBX2,TGFB2,TGFBR1,TGFBR2,TP73,ZFPM2,HEY2,FGF20,BMP10,ARID2 |
| 14_Member | GO Biological Processes | GO:0055024 | regulation of cardiac muscle tissue development | -6.577718317 | -5.087 | 29/100 | 650,657,1385,1482,2066,2263,2475,3084,3720,3776,4208,4851,5292,5594,5728,5999,6909,7046,7048,7161,7528,9472,10818,23414,23493,26281,27302,28514,55553 | BMP2,BMPR1A,CREB1,NKX2-5,ERBB4,FGFR2,MTOR,NRG1,JARID2,KCNK2,MEF2C,NOTCH1,PIM1,MAPK1,PTEN,RGS4,TBX2,TGFBR1,TGFBR2,TP73,YY1,AKAP6,FRS2,ZFPM2,HEY2,FGF20,BMP10,DLL1,SOX6 |
| 14_Member | GO Biological Processes | GO:0046620 | regulation of organ growth | -6.39029743 | -4.921 | 31/113 | 657,1482,1848,2066,2263,2475,3720,3776,4208,4851,5292,5594,5629,5728,5999,6788,6909,7046,7048,7161,7528,9254,9472,23286,23414,23493,26281,26524,27302,55841,80014 | BMPR1A,NKX2-5,DUSP6,ERBB4,FGFR2,MTOR,JARID2,KCNK2,MEF2C,NOTCH1,PIM1,MAPK1,PROX1,PTEN,RGS4,STK3,TBX2,TGFBR1,TGFBR2,TP73,YY1,CACNA2D2,AKAP6,WWC1,ZFPM2,HEY2,FGF20,LATS2,BMP10,WWC3,WWC2 |
| 14_Member | GO Biological Processes | GO:0060420 | regulation of heart growth | -5.993164633 | -4.567 | 25/84 | 657,1482,1848,2066,2263,2475,3720,3776,4208,4851,5292,5594,5629,5728,5999,6909,7046,7048,7161,7528,9472,23414,23493,26281,27302 | BMPR1A,NKX2-5,DUSP6,ERBB4,FGFR2,MTOR,JARID2,KCNK2,MEF2C,NOTCH1,PIM1,MAPK1,PROX1,PTEN,RGS4,TBX2,TGFBR1,TGFBR2,TP73,YY1,AKAP6,ZFPM2,HEY2,FGF20,BMP10 |
| 14_Member | GO Biological Processes | GO:0060043 | regulation of cardiac muscle cell proliferation | -5.512666035 | -4.146 | 19/57 | 657,1482,2066,2263,3720,3776,4208,4851,5292,5594,5728,6909,7046,7048,7161,23414,23493,26281,27302 | BMPR1A,NKX2-5,ERBB4,FGFR2,JARID2,KCNK2,MEF2C,NOTCH1,PIM1,MAPK1,PTEN,TBX2,TGFBR1,TGFBR2,TP73,ZFPM2,HEY2,FGF20,BMP10 |
| 14_Member | GO Biological Processes | GO:0055021 | regulation of cardiac muscle tissue growth | -5.489459607 | -4.126 | 23/78 | 657,1482,2066,2263,2475,3720,3776,4208,4851,5292,5594,5728,5999,6909,7046,7048,7161,7528,9472,23414,23493,26281,27302 | BMPR1A,NKX2-5,ERBB4,FGFR2,MTOR,JARID2,KCNK2,MEF2C,NOTCH1,PIM1,MAPK1,PTEN,RGS4,TBX2,TGFBR1,TGFBR2,TP73,YY1,AKAP6,ZFPM2,HEY2,FGF20,BMP10 |
| 14_Member | GO Biological Processes | GO:1901863 | positive regulation of muscle tissue development | -4.539794314 | -3.268 | 23/88 | 406,657,1385,1499,2066,2263,2475,3084,3156,4208,4534,4851,5292,5594,6909,9472,10891,23414,23493,27302,28514,57496,221662 | ARNTL,BMPR1A,CREB1,CTNNB1,ERBB4,FGFR2,MTOR,NRG1,HMGCR,MEF2C,MTM1,NOTCH1,PIM1,MAPK1,TBX2,AKAP6,PPARGC1A,ZFPM2,HEY2,BMP10,DLL1,MRTFB,RBM24 |
| 14_Member | GO Biological Processes | GO:0048636 | positive regulation of muscle organ development | -4.134652593 | -2.918 | 22/87 | 406,657,1385,1499,2066,2263,2475,3084,3156,4208,4534,4851,5292,5594,6909,9472,23414,23493,27302,28514,57496,221662 | ARNTL,BMPR1A,CREB1,CTNNB1,ERBB4,FGFR2,MTOR,NRG1,HMGCR,MEF2C,MTM1,NOTCH1,PIM1,MAPK1,TBX2,AKAP6,ZFPM2,HEY2,BMP10,DLL1,MRTFB,RBM24 |
| 14_Member | GO Biological Processes | GO:0045844 | positive regulation of striated muscle tissue development | -4.134652593 | -2.918 | 22/87 | 406,657,1385,1499,2066,2263,2475,3084,3156,4208,4534,4851,5292,5594,6909,9472,23414,23493,27302,28514,57496,221662 | ARNTL,BMPR1A,CREB1,CTNNB1,ERBB4,FGFR2,MTOR,NRG1,HMGCR,MEF2C,MTM1,NOTCH1,PIM1,MAPK1,TBX2,AKAP6,ZFPM2,HEY2,BMP10,DLL1,MRTFB,RBM24 |
| 14_Member | GO Biological Processes | GO:0060421 | positive regulation of heart growth | -3.132444526 | -2.062 | 14/52 | 657,2066,2263,2475,4208,4851,5292,5594,5629,6909,9472,23414,23493,27302 | BMPR1A,ERBB4,FGFR2,MTOR,MEF2C,NOTCH1,PIM1,MAPK1,PROX1,TBX2,AKAP6,ZFPM2,HEY2,BMP10 |
| 14_Member | GO Biological Processes | GO:0055023 | positive regulation of cardiac muscle tissue growth | -2.973968561 | -1.922 | 13/48 | 657,2066,2263,2475,4208,4851,5292,5594,6909,9472,23414,23493,27302 | BMPR1A,ERBB4,FGFR2,MTOR,MEF2C,NOTCH1,PIM1,MAPK1,TBX2,AKAP6,ZFPM2,HEY2,BMP10 |
| 15_Summary | GO Molecular Functions | GO:0008134 | transcription factor binding | -26.10674622 | -23.390 | 165/661 | 60,118,196,323,367,406,473,688,860,905,988,1385,1386,1387,1482,1499,1654,1655,1810,1869,1871,1874,1875,1977,2033,2113,2186,2274,2295,2308,2353,2475,2625,2626,2736,2957,3146,3149,3169,3170,3184,3192,3398,3720,3725,4005,4205,4208,4212,4760,4773,4774,4775,4848,4929,5168,5292,5295,5308,5469,5594,5629,5757,5813,5901,5978,6095,6256,6497,6613,6722,6874,6875,6883,6907,6909,6929,6934,6935,6938,6996,7003,7027,7068,7090,7159,7161,7291,7329,7421,7533,7534,7874,7975,7994,8013,8202,8204,8289,8621,8841,9039,9314,9320,9496,9519,9611,9612,9658,9686,9734,9969,10054,10221,10458,10463,10499,10533,10579,10725,10766,10783,10891,11124,23013,23054,23081,23186,23269,23368,23373,23411,23414,23493,23522,23543,23613,25897,25942,27063,28514,28951,28996,29883,30812,51523,51701,55062,55689,55758,55809,55832,57167,57178,57521,57649,63976,79733,84181,92140,93986,150094,166968,221037,284252,463,607,865,1602,2294,2494,2649,2965,3206,4086,4091,4601,4862,5090,5316,5451,5991,6421,6656,6657,6664,6917,6928,7528,8295,8464,9124,9441,9575,10625,23314,23648,25937,51003,51804,55729,56938,80155,83860,117154 | ACTB,ADD1,AHR,APBB2,AR,ARNTL,RERE,KLF5,RUNX2,CCNT2,CDC5L,CREB1,ATF2,CREBBP,NKX2-5,CTNNB1,DDX3X,DDX5,DR1,E2F1,E2F3,E2F4,E2F5,EIF4E,EP300,ETS1,BPTF,FHL2,FOXF2,FOXO1,FOS,MTOR,GATA3,GATA4,GLI2,GTF2A1,HMGB1,HMGB3,FOXA1,FOXA2,HNRNPD,HNRNPU,ID2,JARID2,JUN,LMO2,MEF2A,MEF2C,MEIS2,NEUROD1,NFATC2,NFIA,NFATC3,CNOT2,NR4A2,ENPP2,PIM1,PIK3R1,PITX2,MED1,MAPK1,PROX1,PTMA,PURA,RAN,REST,RORA,RXRA,SKI,SUMO2,SRF,TAF4,TAF4B,TAF12,TBL1X,TBX2,TCF3,TCF7L2,ZEB1,TCF12,TDG,TEAD1,TFDP1,THRB,TLE3,TP53BP2,TP73,TWIST1,UBE2I,VDR,YWHAH,YWHAZ,USP7,MAFK,KAT6A,NR4A3,NCOA3,NRIP1,ARID1A,CDK13,HDAC3,UBA3,KLF4,TRIP12,TBX4,TBPL1,NCOR1,NCOR2,ZNF516,VGLL4,HDAC9,MED13,UBA2,TRIB1,BAIAP2,SLC30A9,NCOA2,ATG7,TACC2,NFAT5,TOB2,NEK6,PPARGC1A,FAF1,SPEN,NCOA6,KDM4C,RCOR1,MGA,PPP1R13B,CRTC1,SIRT1,ZFPM2,HEY2,KAT6B,RBFOX2,ZMYND8,RNF19A,SIN3A,ANKRD1,DLL1,TRIB2,HIPK2,CNOT7,SOX8,CXXC5,NLK,WIPI1,YEATS2,RCOR3,TRERF1,CAND1,SALL4,ZMIZ1,RPTOR,PHF12,PRDM16,E2F8,CHD6,MTDH,FOXP2,SIK1,MIER3,JMJD1C,KCTD1,ZFHX3,BCL9,CBFB,DACH1,FOXF1,NR5A2,NR6A1,GTF2H1,HOXA10,SMAD1,SMAD6,MXI1,NPAS2,PBX3,PKNOX1,POU2F1,RFX3,SFPQ,SOX1,SOX2,SOX11,TCEA1,HNF1B,YY1,TRRAP,SUPT3H,PDLIM1,MED26,CLOCK,IVNS1ABP,SATB2,SSBP3,WWTR1,MED31,SIX4,ATF7IP,ARNTL2,NAA15,TAF3,DACH2 |
| 15_Member | GO Molecular Functions | GO:0008134 | transcription factor binding | -26.10674622 | -23.390 | 165/661 | 60,118,196,323,367,406,473,688,860,905,988,1385,1386,1387,1482,1499,1654,1655,1810,1869,1871,1874,1875,1977,2033,2113,2186,2274,2295,2308,2353,2475,2625,2626,2736,2957,3146,3149,3169,3170,3184,3192,3398,3720,3725,4005,4205,4208,4212,4760,4773,4774,4775,4848,4929,5168,5292,5295,5308,5469,5594,5629,5757,5813,5901,5978,6095,6256,6497,6613,6722,6874,6875,6883,6907,6909,6929,6934,6935,6938,6996,7003,7027,7068,7090,7159,7161,7291,7329,7421,7533,7534,7874,7975,7994,8013,8202,8204,8289,8621,8841,9039,9314,9320,9496,9519,9611,9612,9658,9686,9734,9969,10054,10221,10458,10463,10499,10533,10579,10725,10766,10783,10891,11124,23013,23054,23081,23186,23269,23368,23373,23411,23414,23493,23522,23543,23613,25897,25942,27063,28514,28951,28996,29883,30812,51523,51701,55062,55689,55758,55809,55832,57167,57178,57521,57649,63976,79733,84181,92140,93986,150094,166968,221037,284252 | ACTB,ADD1,AHR,APBB2,AR,ARNTL,RERE,KLF5,RUNX2,CCNT2,CDC5L,CREB1,ATF2,CREBBP,NKX2-5,CTNNB1,DDX3X,DDX5,DR1,E2F1,E2F3,E2F4,E2F5,EIF4E,EP300,ETS1,BPTF,FHL2,FOXF2,FOXO1,FOS,MTOR,GATA3,GATA4,GLI2,GTF2A1,HMGB1,HMGB3,FOXA1,FOXA2,HNRNPD,HNRNPU,ID2,JARID2,JUN,LMO2,MEF2A,MEF2C,MEIS2,NEUROD1,NFATC2,NFIA,NFATC3,CNOT2,NR4A2,ENPP2,PIM1,PIK3R1,PITX2,MED1,MAPK1,PROX1,PTMA,PURA,RAN,REST,RORA,RXRA,SKI,SUMO2,SRF,TAF4,TAF4B,TAF12,TBL1X,TBX2,TCF3,TCF7L2,ZEB1,TCF12,TDG,TEAD1,TFDP1,THRB,TLE3,TP53BP2,TP73,TWIST1,UBE2I,VDR,YWHAH,YWHAZ,USP7,MAFK,KAT6A,NR4A3,NCOA3,NRIP1,ARID1A,CDK13,HDAC3,UBA3,KLF4,TRIP12,TBX4,TBPL1,NCOR1,NCOR2,ZNF516,VGLL4,HDAC9,MED13,UBA2,TRIB1,BAIAP2,SLC30A9,NCOA2,ATG7,TACC2,NFAT5,TOB2,NEK6,PPARGC1A,FAF1,SPEN,NCOA6,KDM4C,RCOR1,MGA,PPP1R13B,CRTC1,SIRT1,ZFPM2,HEY2,KAT6B,RBFOX2,ZMYND8,RNF19A,SIN3A,ANKRD1,DLL1,TRIB2,HIPK2,CNOT7,SOX8,CXXC5,NLK,WIPI1,YEATS2,RCOR3,TRERF1,CAND1,SALL4,ZMIZ1,RPTOR,PHF12,PRDM16,E2F8,CHD6,MTDH,FOXP2,SIK1,MIER3,JMJD1C,KCTD1 |
| 15_Member | GO Cellular Components | GO:0005667 | transcription factor complex | -15.99780322 | -13.806 | 94/365 | 196,406,463,607,688,860,865,1385,1482,1499,1602,1869,1871,1874,1875,2033,2113,2294,2295,2353,2494,2626,2649,2957,2965,3192,3206,3725,4005,4086,4091,4205,4601,4760,4773,4775,4862,4929,5090,5308,5316,5451,5991,6256,6421,6497,6656,6657,6664,6874,6875,6883,6909,6917,6928,6929,6934,6935,6938,7003,7027,7068,7090,7421,7528,8013,8295,8464,9124,9314,9441,9519,9575,9734,10625,10725,23054,23186,23314,23648,25937,25942,28996,30812,51003,51804,55729,55758,55809,56938,79733,80155,83860,117154 | AHR,ARNTL,ZFHX3,BCL9,KLF5,RUNX2,CBFB,CREB1,NKX2-5,CTNNB1,DACH1,E2F1,E2F3,E2F4,E2F5,EP300,ETS1,FOXF1,FOXF2,FOS,NR5A2,GATA4,NR6A1,GTF2A1,GTF2H1,HNRNPU,HOXA10,JUN,LMO2,SMAD1,SMAD6,MEF2A,MXI1,NEUROD1,NFATC2,NFATC3,NPAS2,NR4A2,PBX3,PITX2,PKNOX1,POU2F1,RFX3,RXRA,SFPQ,SKI,SOX1,SOX2,SOX11,TAF4,TAF4B,TAF12,TBX2,TCEA1,HNF1B,TCF3,TCF7L2,ZEB1,TCF12,TEAD1,TFDP1,THRB,TLE3,VDR,YY1,NR4A3,TRRAP,SUPT3H,PDLIM1,KLF4,MED26,TBPL1,CLOCK,HDAC9,IVNS1ABP,NFAT5,NCOA6,RCOR1,SATB2,SSBP3,WWTR1,SIN3A,HIPK2,SOX8,MED31,SIX4,ATF7IP,RCOR3,TRERF1,ARNTL2,E2F8,NAA15,TAF3,DACH2 |
| 15_Member | GO Cellular Components | GO:0044798 | nuclear transcription factor complex | -7.829884696 | -6.220 | 49/201 | 607,865,1385,1482,1499,1869,1871,1874,1875,2353,2494,2626,2957,2965,3192,3725,4601,4760,4773,4775,5451,6256,6421,6656,6657,6664,6874,6875,6883,6917,6929,6934,6938,7003,7027,7068,7090,7421,8295,8464,9314,9441,9519,10725,28996,30812,51003,79733,83860 | BCL9,CBFB,CREB1,NKX2-5,CTNNB1,E2F1,E2F3,E2F4,E2F5,FOS,NR5A2,GATA4,GTF2A1,GTF2H1,HNRNPU,JUN,MXI1,NEUROD1,NFATC2,NFATC3,POU2F1,RXRA,SFPQ,SOX1,SOX2,SOX11,TAF4,TAF4B,TAF12,TCEA1,TCF3,TCF7L2,TCF12,TEAD1,TFDP1,THRB,TLE3,VDR,TRRAP,SUPT3H,KLF4,MED26,TBPL1,NFAT5,HIPK2,SOX8,MED31,E2F8,TAF3 |
| 15_Member | GO Cellular Components | GO:0090575 | RNA polymerase II transcription factor complex | -7.066022108 | -5.523 | 41/163 | 607,865,1385,1482,1499,1869,1871,1874,1875,2353,2494,2626,2957,2965,3192,3725,4601,4760,5451,6256,6421,6874,6875,6883,6917,6929,6934,6938,7003,7027,7068,7090,7421,8295,8464,9441,9519,28996,51003,79733,83860 | BCL9,CBFB,CREB1,NKX2-5,CTNNB1,E2F1,E2F3,E2F4,E2F5,FOS,NR5A2,GATA4,GTF2A1,GTF2H1,HNRNPU,JUN,MXI1,NEUROD1,POU2F1,RXRA,SFPQ,TAF4,TAF4B,TAF12,TCEA1,TCF3,TCF7L2,TCF12,TEAD1,TFDP1,THRB,TLE3,VDR,TRRAP,SUPT3H,MED26,TBPL1,HIPK2,MED31,E2F8,TAF3 |
| 16_Summary | GO Cellular Components | GO:1990234 | transferase complex | -25.5332276 | -22.864 | 183/779 | 18,60,90,92,890,894,905,996,1021,1027,1164,1387,1810,2033,2081,2957,2965,3643,3720,4008,4734,5214,5229,5291,5295,5469,5563,5573,5576,5631,5927,5978,5980,6400,6502,6632,6874,6875,6883,6885,6917,7046,7186,7321,7322,7323,7329,7334,7335,7336,7403,7994,8065,8089,8110,8295,8405,8408,8411,8445,8452,8454,8464,8507,8535,8621,8678,8726,8812,8816,8851,9021,9306,9474,9519,9589,9655,9734,9781,9821,10048,10210,10238,10277,10336,10393,10444,10580,10622,10714,11146,11275,22823,23001,23054,23081,23085,23126,23142,23168,23269,23291,23338,23411,23492,23512,23522,23774,25793,25820,25852,25897,25962,26009,26122,26234,26249,26269,26994,27154,29915,29982,51003,51009,51230,51646,51719,54165,54467,54619,55031,55167,55197,55208,55209,55294,55689,55827,55832,57610,57688,58499,58508,60560,64326,64422,64750,64839,79139,79577,80012,80018,80155,80176,80204,80232,80311,80314,83860,84289,84333,84541,84661,84733,90293,114907,121536,122830,123169,123879,130399,130507,150094,152485,165679,167153,171546,197131,201595,219771,254170,339745,554251 | ABAT,ACTB,ACVR1,ACVR2A,CCNA2,CCND2,CCNT2,CDC27,CDK6,CDKN1B,CKS2,CREBBP,DR1,EP300,ERN1,GTF2A1,GTF2H1,INSR,JARID2,LMO7,NEDD4,PFKP,PGGT1B,PIK3CB,PIK3R1,MED1,PRKAA2,PRKAR1A,PRKAR2A,PRPS1,KDM5A,REST,REV3L,SEL1L,SKP2,SNRPD1,TAF4,TAF4B,TAF12,MAP3K7,TCEA1,TGFBR1,TRAF2,UBE2D1,UBE2D2,UBE2D3,UBE2I,UBE2N,UBE2V1,UBE2V2,KDM6A,KAT6A,CUL5,YEATS4,DPF3,TRRAP,SPOP,ULK1,EEA1,DYRK2,CUL3,CUL1,SUPT3H,ENC1,CBX4,CDK13,BECN1,EED,CCNK,DCAF5,CDK5R1,SOCS3,SOCS6,ATG5,TBPL1,WTAP,SOCS5,HDAC9,RNF144A,RB1CC1,RANBP9,TOPORS,DCAF7,UBE4B,PCGF3,ANAPC10,ZER1,SORBS1,POLR3G,POLD3,GLMN,KLHL2,MTF2,WDFY3,NCOA6,KDM4C,ERC1,POGZ,DCUN1D4,RTF1,MGA,FBXW11,JADE2,SIRT1,CBX7,SUZ12,KAT6B,BRD1,FBXO7,ARIH1,ARMC8,RNF19A,VIRMA,ZZZ3,EPC2,FBXL5,KLHL3,FBXO8,RNF11,BRPF3,HCFC2,NRBF2,MED31,DERL2,PHF20,YPEL5,CAB39,DCUN1D1,ANKIB1,CCNJ,USP47,MSL2,RPRD1A,DCUN1D2,SETD5,FBXW7,YEATS2,DCAF6,CAND1,RANBP10,ZSWIM6,ZNF462,KMT2C,NAA35,COP1,ATG3,SMURF2,FBXL17,DERL1,CDC73,PHC3,NAA25,NAA15,SPSB1,FBXO11,WDR26,KLHL15,EPC1,TAF3,ING5,PCGF5,KBTBD8,DPY30,CBX2,KLHL13,FBXO32,AEBP2,NAA30,LEO1,DCUN1D3,ACVR1C,UBR3,SIK1,ZNF827,SPTSSB,TENT2,SPTSSA,UBR1,STT3B,CCNY,FBXO33,SPOPL,FBXO48 |
| 16_Member | GO Cellular Components | GO:1990234 | transferase complex | -25.5332276 | -22.864 | 183/779 | 18,60,90,92,890,894,905,996,1021,1027,1164,1387,1810,2033,2081,2957,2965,3643,3720,4008,4734,5214,5229,5291,5295,5469,5563,5573,5576,5631,5927,5978,5980,6400,6502,6632,6874,6875,6883,6885,6917,7046,7186,7321,7322,7323,7329,7334,7335,7336,7403,7994,8065,8089,8110,8295,8405,8408,8411,8445,8452,8454,8464,8507,8535,8621,8678,8726,8812,8816,8851,9021,9306,9474,9519,9589,9655,9734,9781,9821,10048,10210,10238,10277,10336,10393,10444,10580,10622,10714,11146,11275,22823,23001,23054,23081,23085,23126,23142,23168,23269,23291,23338,23411,23492,23512,23522,23774,25793,25820,25852,25897,25962,26009,26122,26234,26249,26269,26994,27154,29915,29982,51003,51009,51230,51646,51719,54165,54467,54619,55031,55167,55197,55208,55209,55294,55689,55827,55832,57610,57688,58499,58508,60560,64326,64422,64750,64839,79139,79577,80012,80018,80155,80176,80204,80232,80311,80314,83860,84289,84333,84541,84661,84733,90293,114907,121536,122830,123169,123879,130399,130507,150094,152485,165679,167153,171546,197131,201595,219771,254170,339745,554251 | ABAT,ACTB,ACVR1,ACVR2A,CCNA2,CCND2,CCNT2,CDC27,CDK6,CDKN1B,CKS2,CREBBP,DR1,EP300,ERN1,GTF2A1,GTF2H1,INSR,JARID2,LMO7,NEDD4,PFKP,PGGT1B,PIK3CB,PIK3R1,MED1,PRKAA2,PRKAR1A,PRKAR2A,PRPS1,KDM5A,REST,REV3L,SEL1L,SKP2,SNRPD1,TAF4,TAF4B,TAF12,MAP3K7,TCEA1,TGFBR1,TRAF2,UBE2D1,UBE2D2,UBE2D3,UBE2I,UBE2N,UBE2V1,UBE2V2,KDM6A,KAT6A,CUL5,YEATS4,DPF3,TRRAP,SPOP,ULK1,EEA1,DYRK2,CUL3,CUL1,SUPT3H,ENC1,CBX4,CDK13,BECN1,EED,CCNK,DCAF5,CDK5R1,SOCS3,SOCS6,ATG5,TBPL1,WTAP,SOCS5,HDAC9,RNF144A,RB1CC1,RANBP9,TOPORS,DCAF7,UBE4B,PCGF3,ANAPC10,ZER1,SORBS1,POLR3G,POLD3,GLMN,KLHL2,MTF2,WDFY3,NCOA6,KDM4C,ERC1,POGZ,DCUN1D4,RTF1,MGA,FBXW11,JADE2,SIRT1,CBX7,SUZ12,KAT6B,BRD1,FBXO7,ARIH1,ARMC8,RNF19A,VIRMA,ZZZ3,EPC2,FBXL5,KLHL3,FBXO8,RNF11,BRPF3,HCFC2,NRBF2,MED31,DERL2,PHF20,YPEL5,CAB39,DCUN1D1,ANKIB1,CCNJ,USP47,MSL2,RPRD1A,DCUN1D2,SETD5,FBXW7,YEATS2,DCAF6,CAND1,RANBP10,ZSWIM6,ZNF462,KMT2C,NAA35,COP1,ATG3,SMURF2,FBXL17,DERL1,CDC73,PHC3,NAA25,NAA15,SPSB1,FBXO11,WDR26,KLHL15,EPC1,TAF3,ING5,PCGF5,KBTBD8,DPY30,CBX2,KLHL13,FBXO32,AEBP2,NAA30,LEO1,DCUN1D3,ACVR1C,UBR3,SIK1,ZNF827,SPTSSB,TENT2,SPTSSA,UBR1,STT3B,CCNY,FBXO33,SPOPL,FBXO48 |
| 16_Member | GO Cellular Components | GO:0000151 | ubiquitin ligase complex | -14.18900297 | -12.119 | 76/282 | 996,1027,1164,4008,4734,5469,6400,6502,7186,7321,7322,7323,7334,7335,8065,8405,8445,8452,8454,8507,8535,8816,9781,10048,10210,10238,10277,10336,10393,10444,11146,11275,23142,23291,23492,25793,25820,25852,25897,26234,26249,26269,26994,51003,51009,51646,54165,54467,55031,55208,55294,55827,55832,57610,57688,64326,64422,64750,64839,79139,80012,80176,80204,80232,80311,84333,84541,84733,90293,114907,123879,130507,197131,254170,339745,554251 | CDC27,CDKN1B,CKS2,LMO7,NEDD4,MED1,SEL1L,SKP2,TRAF2,UBE2D1,UBE2D2,UBE2D3,UBE2N,UBE2V1,CUL5,SPOP,DYRK2,CUL3,CUL1,ENC1,CBX4,DCAF5,RNF144A,RANBP9,TOPORS,DCAF7,UBE4B,PCGF3,ANAPC10,ZER1,GLMN,KLHL2,DCUN1D4,FBXW11,CBX7,FBXO7,ARIH1,ARMC8,RNF19A,FBXL5,KLHL3,FBXO8,RNF11,MED31,DERL2,YPEL5,DCUN1D1,ANKIB1,USP47,DCUN1D2,FBXW7,DCAF6,CAND1,RANBP10,ZSWIM6,COP1,ATG3,SMURF2,FBXL17,DERL1,PHC3,SPSB1,FBXO11,WDR26,KLHL15,PCGF5,KBTBD8,CBX2,KLHL13,FBXO32,DCUN1D3,UBR3,UBR1,FBXO33,SPOPL,FBXO48 |
| 16_Member | GO Cellular Components | GO:0031461 | cullin-RING ubiquitin ligase complex | -4.740153119 | -3.449 | 35/159 | 996,1027,1164,6502,8065,8405,8452,8454,8507,8816,10238,10393,10444,11146,11275,23291,25793,25820,26234,26249,55031,55294,55827,55832,57688,64326,64839,80176,80311,84541,90293,114907,254170,339745,554251 | CDC27,CDKN1B,CKS2,SKP2,CUL5,SPOP,CUL3,CUL1,ENC1,DCAF5,DCAF7,ANAPC10,ZER1,GLMN,KLHL2,FBXW11,FBXO7,ARIH1,FBXL5,KLHL3,USP47,FBXW7,DCAF6,CAND1,ZSWIM6,COP1,FBXL17,SPSB1,KLHL15,KBTBD8,KLHL13,FBXO32,FBXO33,SPOPL,FBXO48 |
| 16_Member | GO Cellular Components | GO:0019005 | SCF ubiquitin ligase complex | -3.031519231 | -1.976 | 16/65 | 1164,6502,8065,8452,8454,23291,25793,25820,26234,55031,55294,64839,80176,114907,254170,554251 | CKS2,SKP2,CUL5,CUL3,CUL1,FBXW11,FBXO7,ARIH1,FBXL5,USP47,FBXW7,FBXL17,SPSB1,FBXO32,FBXO33,FBXO48 |
| 17_Summary | GO Biological Processes | GO:0007264 | small GTPase mediated signal transduction | -25.13283584 | -22.481 | 148/572 | 25,27,387,390,394,395,604,890,998,1123,1124,1278,1281,1398,1399,1600,2048,2059,2255,2782,2885,3084,3688,3717,3725,3845,3984,4763,4803,4851,4853,5516,5649,5861,5868,5870,5878,5898,5900,5908,5911,5912,5921,5922,5924,5962,6009,6091,6093,6236,6239,6453,6654,6655,7042,7074,7204,7879,8036,8452,8660,8766,8829,8936,9267,9353,9367,9411,9448,9475,9620,9649,9693,9732,9743,9771,9824,9886,9908,9922,9927,10146,10152,10160,10252,10253,10276,10395,10564,10565,10672,10787,10890,10928,10966,10971,10982,11021,22800,22931,23011,23108,23179,23189,23229,23348,23362,23607,25894,26053,26230,26269,27314,50618,50650,51552,51715,51762,54453,54848,55103,55114,55288,55785,55789,56990,57381,57403,57493,57522,57568,57569,57584,57646,58480,65979,83452,83478,84144,84253,90627,93663,94134,115827,117289,121512,201475,253959 | ABL1,ABL2,RHOA,RND3,ARHGAP5,ARHGAP6,BCL6,CCNA2,CDC42,CHN1,CHN2,COL1A2,COL3A1,CRK,CRKL,DAB1,EPHB2,EPS8,FGF10,GNB1,GRB2,NRG1,ITGB1,JAK2,JUN,KRAS,LIMK1,NF1,NGF,NOTCH1,NOTCH2,PPP2CB,RELN,RAB1A,RAB5A,RAB6A,RAB5C,RALA,RALGDS,RAP1B,RAP2A,RAP2B,RASA1,RASA2,RASGRF2,RDX,RHEB,ROBO1,ROCK1,RRAD,RREB1,ITSN1,SOS1,SOS2,TGFB2,TIAM1,TRIO,RAB7A,SHOC2,CUL3,IRS2,RAB11A,NRP1,WASF1,CYTH1,SLIT2,RAB9A,ARHGAP29,MAP4K4,ROCK2,CELSR1,RALGPS1,RAPGEF2,DOCK4,ARHGAP32,RAPGEF5,ARHGAP11A,RHOBTB1,G3BP2,IQSEC1,MFN2,G3BP1,ABI2,FARP1,SPRY1,SPRY2,NET1,DLC1,ARFGEF2,ARFGEF1,GNA13,NCKAP1,RAB10,RALBP1,RAB40B,YWHAQ,MAPRE2,RAB35,RRAS2,RAB18,RAB21,RAP1GAP2,RGL1,KANK1,ARHGEF9,DOCK9,PSD3,CD2AP,PLEKHG4,AUTS2,TIAM2,FBXO8,RAB30,ITSN2,ARHGEF3,RAB14,RAB23,RAB8B,RIN2,ARHGEF38,RALGPS2,ARHGAP17,RHOT1,FGD6,DEPDC1B,CDC42SE2,RHOJ,RAB22A,HEG1,SRGAP1,SIPA1L2,ARHGAP20,ARHGAP21,USP28,RHOU,PHACTR4,RAB33B,ARHGAP24,SYDE2,GARNL3,STARD13,ARHGAP18,ARHGAP12,RAB3C,TAGAP,FGD4,RAB12,RALGAPA1 |
| 17_Member | GO Biological Processes | GO:0007264 | small GTPase mediated signal transduction | -25.13283584 | -22.481 | 148/572 | 25,27,387,390,394,395,604,890,998,1123,1124,1278,1281,1398,1399,1600,2048,2059,2255,2782,2885,3084,3688,3717,3725,3845,3984,4763,4803,4851,4853,5516,5649,5861,5868,5870,5878,5898,5900,5908,5911,5912,5921,5922,5924,5962,6009,6091,6093,6236,6239,6453,6654,6655,7042,7074,7204,7879,8036,8452,8660,8766,8829,8936,9267,9353,9367,9411,9448,9475,9620,9649,9693,9732,9743,9771,9824,9886,9908,9922,9927,10146,10152,10160,10252,10253,10276,10395,10564,10565,10672,10787,10890,10928,10966,10971,10982,11021,22800,22931,23011,23108,23179,23189,23229,23348,23362,23607,25894,26053,26230,26269,27314,50618,50650,51552,51715,51762,54453,54848,55103,55114,55288,55785,55789,56990,57381,57403,57493,57522,57568,57569,57584,57646,58480,65979,83452,83478,84144,84253,90627,93663,94134,115827,117289,121512,201475,253959 | ABL1,ABL2,RHOA,RND3,ARHGAP5,ARHGAP6,BCL6,CCNA2,CDC42,CHN1,CHN2,COL1A2,COL3A1,CRK,CRKL,DAB1,EPHB2,EPS8,FGF10,GNB1,GRB2,NRG1,ITGB1,JAK2,JUN,KRAS,LIMK1,NF1,NGF,NOTCH1,NOTCH2,PPP2CB,RELN,RAB1A,RAB5A,RAB6A,RAB5C,RALA,RALGDS,RAP1B,RAP2A,RAP2B,RASA1,RASA2,RASGRF2,RDX,RHEB,ROBO1,ROCK1,RRAD,RREB1,ITSN1,SOS1,SOS2,TGFB2,TIAM1,TRIO,RAB7A,SHOC2,CUL3,IRS2,RAB11A,NRP1,WASF1,CYTH1,SLIT2,RAB9A,ARHGAP29,MAP4K4,ROCK2,CELSR1,RALGPS1,RAPGEF2,DOCK4,ARHGAP32,RAPGEF5,ARHGAP11A,RHOBTB1,G3BP2,IQSEC1,MFN2,G3BP1,ABI2,FARP1,SPRY1,SPRY2,NET1,DLC1,ARFGEF2,ARFGEF1,GNA13,NCKAP1,RAB10,RALBP1,RAB40B,YWHAQ,MAPRE2,RAB35,RRAS2,RAB18,RAB21,RAP1GAP2,RGL1,KANK1,ARHGEF9,DOCK9,PSD3,CD2AP,PLEKHG4,AUTS2,TIAM2,FBXO8,RAB30,ITSN2,ARHGEF3,RAB14,RAB23,RAB8B,RIN2,ARHGEF38,RALGPS2,ARHGAP17,RHOT1,FGD6,DEPDC1B,CDC42SE2,RHOJ,RAB22A,HEG1,SRGAP1,SIPA1L2,ARHGAP20,ARHGAP21,USP28,RHOU,PHACTR4,RAB33B,ARHGAP24,SYDE2,GARNL3,STARD13,ARHGAP18,ARHGAP12,RAB3C,TAGAP,FGD4,RAB12,RALGAPA1 |
| 17_Member | GO Biological Processes | GO:0007265 | Ras protein signal transduction | -19.72657974 | -17.330 | 116/449 | 25,27,387,390,394,395,604,890,998,1278,1281,1398,1399,2048,2059,2255,2782,2885,3084,3688,3717,3725,3845,3984,4763,4803,4851,4853,5516,5861,5868,5870,5878,5898,5900,5908,5911,5912,5921,5922,5924,5962,6091,6093,6239,6453,6654,6655,7042,7074,7204,7879,8036,8452,8660,8766,8829,8936,9267,9367,9411,9448,9475,9620,9649,9693,9886,9908,9922,9927,10146,10152,10160,10252,10253,10276,10395,10564,10565,10672,10787,10890,10966,10982,11021,22800,22931,23011,23189,23229,23362,25894,26053,26230,26269,27314,50618,50650,51552,51715,51762,54848,55103,55288,55785,56990,57381,57403,57493,57646,58480,65979,83452,115827,121512,201475 | ABL1,ABL2,RHOA,RND3,ARHGAP5,ARHGAP6,BCL6,CCNA2,CDC42,COL1A2,COL3A1,CRK,CRKL,EPHB2,EPS8,FGF10,GNB1,GRB2,NRG1,ITGB1,JAK2,JUN,KRAS,LIMK1,NF1,NGF,NOTCH1,NOTCH2,PPP2CB,RAB1A,RAB5A,RAB6A,RAB5C,RALA,RALGDS,RAP1B,RAP2A,RAP2B,RASA1,RASA2,RASGRF2,RDX,ROBO1,ROCK1,RREB1,ITSN1,SOS1,SOS2,TGFB2,TIAM1,TRIO,RAB7A,SHOC2,CUL3,IRS2,RAB11A,NRP1,WASF1,CYTH1,RAB9A,ARHGAP29,MAP4K4,ROCK2,CELSR1,RALGPS1,RAPGEF2,RHOBTB1,G3BP2,IQSEC1,MFN2,G3BP1,ABI2,FARP1,SPRY1,SPRY2,NET1,DLC1,ARFGEF2,ARFGEF1,GNA13,NCKAP1,RAB10,RAB40B,MAPRE2,RAB35,RRAS2,RAB18,RAB21,KANK1,ARHGEF9,PSD3,PLEKHG4,AUTS2,TIAM2,FBXO8,RAB30,ITSN2,ARHGEF3,RAB14,RAB23,RAB8B,ARHGEF38,RALGPS2,RHOT1,FGD6,CDC42SE2,RHOJ,RAB22A,HEG1,USP28,RHOU,PHACTR4,RAB33B,RAB3C,FGD4,RAB12 |
| 17_Member | GO Biological Processes | GO:0051056 | regulation of small GTPase mediated signal transduction | -18.82720713 | -16.489 | 95/339 | 25,27,387,394,395,604,998,1123,1124,1281,1398,1399,2048,2059,2255,2885,3084,3688,3717,3845,4763,4803,4851,4853,5516,5649,5921,5922,5924,5962,6091,6453,6654,6655,7042,7074,7204,8036,8452,8660,8829,9267,9353,9411,9448,9649,9743,9824,9886,9922,9927,10160,10252,10253,10276,10395,10564,10565,10672,10928,10982,23108,23189,23229,23362,23607,25894,26053,26230,26269,50618,50650,54848,55103,55114,55288,55785,55789,56990,57381,57493,57522,57568,57569,57584,58480,83478,84144,84253,90627,93663,94134,117289,121512,253959 | ABL1,ABL2,RHOA,ARHGAP5,ARHGAP6,BCL6,CDC42,CHN1,CHN2,COL3A1,CRK,CRKL,EPHB2,EPS8,FGF10,GRB2,NRG1,ITGB1,JAK2,KRAS,NF1,NGF,NOTCH1,NOTCH2,PPP2CB,RELN,RASA1,RASA2,RASGRF2,RDX,ROBO1,ITSN1,SOS1,SOS2,TGFB2,TIAM1,TRIO,SHOC2,CUL3,IRS2,NRP1,CYTH1,SLIT2,ARHGAP29,MAP4K4,RALGPS1,ARHGAP32,ARHGAP11A,RHOBTB1,IQSEC1,MFN2,FARP1,SPRY1,SPRY2,NET1,DLC1,ARFGEF2,ARFGEF1,GNA13,RALBP1,MAPRE2,RAP1GAP2,KANK1,ARHGEF9,PSD3,CD2AP,PLEKHG4,AUTS2,TIAM2,FBXO8,ITSN2,ARHGEF3,ARHGEF38,RALGPS2,ARHGAP17,RHOT1,FGD6,DEPDC1B,CDC42SE2,RHOJ,HEG1,SRGAP1,SIPA1L2,ARHGAP20,ARHGAP21,RHOU,ARHGAP24,SYDE2,GARNL3,STARD13,ARHGAP18,ARHGAP12,TAGAP,FGD4,RALGAPA1 |
| 17_Member | GO Biological Processes | GO:0046578 | regulation of Ras protein signal transduction | -10.92979796 | -9.062 | 62/239 | 25,27,604,1281,1398,1399,2048,2059,2255,2885,3084,3688,3717,3845,4763,4803,4851,4853,5516,5921,5922,5924,5962,6091,6453,6654,6655,7042,7074,7204,8036,8452,8660,8829,9267,9448,9649,9922,9927,10160,10252,10253,10276,10395,10564,10565,10982,23189,23229,23362,25894,26053,26230,26269,50618,50650,54848,55103,55785,56990,57493,121512 | ABL1,ABL2,BCL6,COL3A1,CRK,CRKL,EPHB2,EPS8,FGF10,GRB2,NRG1,ITGB1,JAK2,KRAS,NF1,NGF,NOTCH1,NOTCH2,PPP2CB,RASA1,RASA2,RASGRF2,RDX,ROBO1,ITSN1,SOS1,SOS2,TGFB2,TIAM1,TRIO,SHOC2,CUL3,IRS2,NRP1,CYTH1,MAP4K4,RALGPS1,IQSEC1,MFN2,FARP1,SPRY1,SPRY2,NET1,DLC1,ARFGEF2,ARFGEF1,MAPRE2,KANK1,ARHGEF9,PSD3,PLEKHG4,AUTS2,TIAM2,FBXO8,ITSN2,ARHGEF3,ARHGEF38,RALGPS2,FGD6,CDC42SE2,HEG1,FGD4 |
| 17_Member | GO Biological Processes | GO:0007266 | Rho protein signal transduction | -6.33011374 | -4.869 | 46/204 | 25,27,387,390,394,395,604,998,1278,1281,2059,3688,3984,5924,6091,6093,6453,6654,6655,7074,7204,8452,8829,9411,9475,9620,9886,10160,10276,10395,10672,23189,23229,25894,26230,50618,50650,54848,55288,55785,56990,57381,57493,58480,65979,121512 | ABL1,ABL2,RHOA,RND3,ARHGAP5,ARHGAP6,BCL6,CDC42,COL1A2,COL3A1,EPS8,ITGB1,LIMK1,RASGRF2,ROBO1,ROCK1,ITSN1,SOS1,SOS2,TIAM1,TRIO,CUL3,NRP1,ARHGAP29,ROCK2,CELSR1,RHOBTB1,FARP1,NET1,DLC1,GNA13,KANK1,ARHGEF9,PLEKHG4,TIAM2,ITSN2,ARHGEF3,ARHGEF38,RHOT1,FGD6,CDC42SE2,RHOJ,HEG1,RHOU,PHACTR4,FGD4 |
| 17_Member | GO Biological Processes | GO:0035023 | regulation of Rho protein signal transduction | -3.501049209 | -2.372 | 29/141 | 25,27,604,1281,2059,3688,5924,6091,6453,6654,6655,7074,7204,8452,8829,10160,10276,10395,23189,23229,25894,26230,50618,50650,54848,55785,56990,57493,121512 | ABL1,ABL2,BCL6,COL3A1,EPS8,ITGB1,RASGRF2,ROBO1,ITSN1,SOS1,SOS2,TIAM1,TRIO,CUL3,NRP1,FARP1,NET1,DLC1,KANK1,ARHGEF9,PLEKHG4,TIAM2,ITSN2,ARHGEF3,ARHGEF38,FGD6,CDC42SE2,HEG1,FGD4 |
| 18_Summary | GO Biological Processes | GO:0016358 | dendrite development | -24.66499207 | -22.030 | 84/233 | 102,351,375,387,473,655,998,1398,1399,1501,1600,1826,2043,2048,2182,2332,2475,2534,3688,4038,4076,4131,4133,4139,4205,4208,4734,5048,5062,5063,5530,5592,5597,5649,5728,5789,5911,6383,6453,7074,7101,7224,7337,7436,7533,8301,8440,8609,8829,8851,8976,9201,9231,9693,10006,10152,10160,10458,10479,10507,11141,22849,22871,22903,22941,23011,23072,23098,23242,23271,23327,23373,23543,23613,26039,50807,55607,57118,57468,57492,57520,65981,114788,221935,60,1607,1739,1740,2017,2045,2119,2668,2743,2895,2904,3643,4628,4897,8828,8898,9378,9456,9863,10243,10397,22866,26045,55737,58489,91752,388336 | ADAM10,APP,ARF1,RHOA,RERE,BMP7,CDC42,CRK,CRKL,CTNND2,DAB1,DSCAM,EPHA4,EPHB2,ACSL4,FMR1,MTOR,FYN,ITGB1,LRP4,CAPRIN1,MAP1B,MAP2,MARK1,MEF2A,MEF2C,NEDD4,PAFAH1B1,PAK2,PAK3,PPP3CA,PRKG1,MAPK6,RELN,PTEN,PTPRD,RAP2A,SDC2,ITSN1,TIAM1,NR2E1,TRPC5,UBE3A,VLDLR,YWHAH,PICALM,NCK2,KLF7,NRP1,CDK5R1,WASL,DCLK1,DLG5,RAPGEF2,ABI1,ABI2,FARP1,BAIAP2,SLC9A6,SEMA4D,IL1RAPL1,CPEB3,NLGN1,BTBD3,SHANK2,RAB21,HECW1,SARM1,COBL,CAMSAP2,NEDD4L,CRTC1,RBFOX2,ZMYND8,SS18L1,ASAP1,PPP1R9A,CAMK1D,SLC12A5,ARID1B,HECW2,CAPRIN2,CSMD3,SDK1,ACTB,DGKB,DLG1,DLG2,CTTN,EPHA7,ETV5,GDNF,GLRB,GRID2,GRIN2B,INSR,MYH10,NRCAM,NRP2,MTMR2,NRXN1,HOMER1,MAGI2,GPHN,NDRG1,CNKSR2,LRRTM2,VPS35,ABHD17C,ZNF804A,SHISA6 |
| 18_Member | GO Biological Processes | GO:0016358 | dendrite development | -24.66499207 | -22.030 | 84/233 | 102,351,375,387,473,655,998,1398,1399,1501,1600,1826,2043,2048,2182,2332,2475,2534,3688,4038,4076,4131,4133,4139,4205,4208,4734,5048,5062,5063,5530,5592,5597,5649,5728,5789,5911,6383,6453,7074,7101,7224,7337,7436,7533,8301,8440,8609,8829,8851,8976,9201,9231,9693,10006,10152,10160,10458,10479,10507,11141,22849,22871,22903,22941,23011,23072,23098,23242,23271,23327,23373,23543,23613,26039,50807,55607,57118,57468,57492,57520,65981,114788,221935 | ADAM10,APP,ARF1,RHOA,RERE,BMP7,CDC42,CRK,CRKL,CTNND2,DAB1,DSCAM,EPHA4,EPHB2,ACSL4,FMR1,MTOR,FYN,ITGB1,LRP4,CAPRIN1,MAP1B,MAP2,MARK1,MEF2A,MEF2C,NEDD4,PAFAH1B1,PAK2,PAK3,PPP3CA,PRKG1,MAPK6,RELN,PTEN,PTPRD,RAP2A,SDC2,ITSN1,TIAM1,NR2E1,TRPC5,UBE3A,VLDLR,YWHAH,PICALM,NCK2,KLF7,NRP1,CDK5R1,WASL,DCLK1,DLG5,RAPGEF2,ABI1,ABI2,FARP1,BAIAP2,SLC9A6,SEMA4D,IL1RAPL1,CPEB3,NLGN1,BTBD3,SHANK2,RAB21,HECW1,SARM1,COBL,CAMSAP2,NEDD4L,CRTC1,RBFOX2,ZMYND8,SS18L1,ASAP1,PPP1R9A,CAMK1D,SLC12A5,ARID1B,HECW2,CAPRIN2,CSMD3,SDK1 |
| 18_Member | GO Biological Processes | GO:0048813 | dendrite morphogenesis | -17.1336362 | -14.862 | 54/143 | 102,473,998,1501,1826,2043,2048,2332,2534,3688,4038,4076,4133,4205,4734,5048,5063,5530,5649,5728,5789,5911,6383,7074,7101,7224,7337,7436,7533,8301,8609,8829,8851,8976,9201,9693,10006,10152,10160,10458,10507,11141,22871,22903,22941,23011,23072,23098,23327,23543,26039,55607,57520,65981 | ADAM10,RERE,CDC42,CTNND2,DSCAM,EPHA4,EPHB2,FMR1,FYN,ITGB1,LRP4,CAPRIN1,MAP2,MEF2A,NEDD4,PAFAH1B1,PAK3,PPP3CA,RELN,PTEN,PTPRD,RAP2A,SDC2,TIAM1,NR2E1,TRPC5,UBE3A,VLDLR,YWHAH,PICALM,KLF7,NRP1,CDK5R1,WASL,DCLK1,RAPGEF2,ABI1,ABI2,FARP1,BAIAP2,SEMA4D,IL1RAPL1,NLGN1,BTBD3,SHANK2,RAB21,HECW1,SARM1,NEDD4L,RBFOX2,SS18L1,PPP1R9A,HECW2,CAPRIN2 |
| 18_Member | GO Biological Processes | GO:0050773 | regulation of dendrite development | -16.916029 | -14.662 | 55/149 | 102,375,387,655,1398,1399,2043,2332,2475,4076,4139,4208,4734,5048,5063,5530,5597,5649,5728,5789,5911,6383,6453,7074,7101,7224,7337,7436,7533,8851,9231,9693,10152,10458,10507,11141,22849,22871,22941,23011,23072,23098,23242,23271,23327,23373,23613,26039,50807,55607,57118,57520,65981,114788,221935 | ADAM10,ARF1,RHOA,BMP7,CRK,CRKL,EPHA4,FMR1,MTOR,CAPRIN1,MARK1,MEF2C,NEDD4,PAFAH1B1,PAK3,PPP3CA,MAPK6,RELN,PTEN,PTPRD,RAP2A,SDC2,ITSN1,TIAM1,NR2E1,TRPC5,UBE3A,VLDLR,YWHAH,CDK5R1,DLG5,RAPGEF2,ABI2,BAIAP2,SEMA4D,IL1RAPL1,CPEB3,NLGN1,SHANK2,RAB21,HECW1,SARM1,COBL,CAMSAP2,NEDD4L,CRTC1,ZMYND8,SS18L1,ASAP1,PPP1R9A,CAMK1D,HECW2,CAPRIN2,CSMD3,SDK1 |
| 18_Member | GO Biological Processes | GO:0099173 | postsynapse organization | -14.93058398 | -12.816 | 55/163 | 60,102,375,998,1399,1501,1607,1739,1740,2017,2043,2045,2048,2119,2534,2668,2743,2895,2904,3643,4038,4076,4628,4897,5048,5063,5649,5728,5789,7074,7337,8828,8829,8851,8898,8976,9378,9456,9863,10152,10243,10397,10458,11141,22866,22871,22941,23613,26045,55607,55737,58489,65981,91752,388336 | ACTB,ADAM10,ARF1,CDC42,CRKL,CTNND2,DGKB,DLG1,DLG2,CTTN,EPHA4,EPHA7,EPHB2,ETV5,FYN,GDNF,GLRB,GRID2,GRIN2B,INSR,LRP4,CAPRIN1,MYH10,NRCAM,PAFAH1B1,PAK3,RELN,PTEN,PTPRD,TIAM1,UBE3A,NRP2,NRP1,CDK5R1,MTMR2,WASL,NRXN1,HOMER1,MAGI2,ABI2,GPHN,NDRG1,BAIAP2,IL1RAPL1,CNKSR2,NLGN1,SHANK2,ZMYND8,LRRTM2,PPP1R9A,VPS35,ABHD17C,CAPRIN2,ZNF804A,SHISA6 |
| 18_Member | GO Biological Processes | GO:0060996 | dendritic spine development | -12.12161461 | -10.194 | 37/97 | 102,375,998,1501,2043,2048,2182,2332,2475,4076,4208,5048,5062,5063,5597,5649,5728,6453,7074,7337,8440,8851,8976,9231,10152,10458,10479,22849,22871,22941,23613,50807,55607,57468,57492,65981,221935 | ADAM10,ARF1,CDC42,CTNND2,EPHA4,EPHB2,ACSL4,FMR1,MTOR,CAPRIN1,MEF2C,PAFAH1B1,PAK2,PAK3,MAPK6,RELN,PTEN,ITSN1,TIAM1,UBE3A,NCK2,CDK5R1,WASL,DLG5,ABI2,BAIAP2,SLC9A6,CPEB3,NLGN1,SHANK2,ZMYND8,ASAP1,PPP1R9A,SLC12A5,ARID1B,CAPRIN2,SDK1 |
| 18_Member | GO Biological Processes | GO:0048814 | regulation of dendrite morphogenesis | -10.19431831 | -8.380 | 33/91 | 102,2043,2332,4076,4734,5048,5063,5530,5649,5728,5789,5911,6383,7074,7101,7224,7337,7533,8851,9693,10152,10458,10507,11141,22871,23011,23072,23098,23327,26039,55607,57520,65981 | ADAM10,EPHA4,FMR1,CAPRIN1,NEDD4,PAFAH1B1,PAK3,PPP3CA,RELN,PTEN,PTPRD,RAP2A,SDC2,TIAM1,NR2E1,TRPC5,UBE3A,YWHAH,CDK5R1,RAPGEF2,ABI2,BAIAP2,SEMA4D,IL1RAPL1,NLGN1,RAB21,HECW1,SARM1,NEDD4L,SS18L1,PPP1R9A,HECW2,CAPRIN2 |
| 18_Member | GO Biological Processes | GO:0099175 | regulation of postsynapse organization | -9.875602367 | -8.087 | 34/98 | 102,1399,1607,2043,2045,2534,2895,2904,4038,4076,4897,5048,5063,5649,5728,5789,7074,7337,8828,8851,9378,9456,10152,10458,11141,22871,23613,26045,55607,55737,58489,65981,91752,388336 | ADAM10,CRKL,DGKB,EPHA4,EPHA7,FYN,GRID2,GRIN2B,LRP4,CAPRIN1,NRCAM,PAFAH1B1,PAK3,RELN,PTEN,PTPRD,TIAM1,UBE3A,NRP2,CDK5R1,NRXN1,HOMER1,ABI2,BAIAP2,IL1RAPL1,NLGN1,ZMYND8,LRRTM2,PPP1R9A,VPS35,ABHD17C,CAPRIN2,ZNF804A,SHISA6 |
| 18_Member | GO Biological Processes | GO:0097061 | dendritic spine organization | -9.746289432 | -7.970 | 30/80 | 102,375,998,1501,2017,2043,2048,2534,2904,3643,4076,5048,5063,5649,5728,7074,7337,8851,8898,8976,9456,10152,10458,22871,22941,23613,55607,55737,65981,91752 | ADAM10,ARF1,CDC42,CTNND2,CTTN,EPHA4,EPHB2,FYN,GRIN2B,INSR,CAPRIN1,PAFAH1B1,PAK3,RELN,PTEN,TIAM1,UBE3A,CDK5R1,MTMR2,WASL,HOMER1,ABI2,BAIAP2,NLGN1,SHANK2,ZMYND8,PPP1R9A,VPS35,CAPRIN2,ZNF804A |
| 18_Member | GO Biological Processes | GO:0106027 | neuron projection organization | -9.254745958 | -7.512 | 31/88 | 102,351,375,998,1501,2017,2043,2048,2534,2904,3643,4076,5048,5063,5649,5728,7074,7337,8851,8898,8976,9456,10152,10458,22871,22941,23613,55607,55737,65981,91752 | ADAM10,APP,ARF1,CDC42,CTNND2,CTTN,EPHA4,EPHB2,FYN,GRIN2B,INSR,CAPRIN1,PAFAH1B1,PAK3,RELN,PTEN,TIAM1,UBE3A,CDK5R1,MTMR2,WASL,HOMER1,ABI2,BAIAP2,NLGN1,SHANK2,ZMYND8,PPP1R9A,VPS35,CAPRIN2,ZNF804A |
| 18_Member | GO Biological Processes | GO:0060998 | regulation of dendritic spine development | -8.533860956 | -6.860 | 27/74 | 102,375,2043,2332,2475,4076,4208,5048,5063,5597,5649,5728,6453,7074,7337,8851,9231,10152,10458,22849,22871,22941,23613,50807,55607,65981,221935 | ADAM10,ARF1,EPHA4,FMR1,MTOR,CAPRIN1,MEF2C,PAFAH1B1,PAK3,MAPK6,RELN,PTEN,ITSN1,TIAM1,UBE3A,CDK5R1,DLG5,ABI2,BAIAP2,CPEB3,NLGN1,SHANK2,ZMYND8,ASAP1,PPP1R9A,CAPRIN2,SDK1 |
| 18_Member | GO Biological Processes | GO:1900006 | positive regulation of dendrite development | -8.533860956 | -6.860 | 27/74 | 375,655,2043,2332,2475,4076,5048,5063,5597,5649,5789,6453,7074,7436,9231,10458,11141,22849,22871,22941,23011,23242,23373,23613,26039,55607,65981 | ARF1,BMP7,EPHA4,FMR1,MTOR,CAPRIN1,PAFAH1B1,PAK3,MAPK6,RELN,PTPRD,ITSN1,TIAM1,VLDLR,DLG5,BAIAP2,IL1RAPL1,CPEB3,NLGN1,SHANK2,RAB21,COBL,CRTC1,ZMYND8,SS18L1,PPP1R9A,CAPRIN2 |
| 18_Member | GO Biological Processes | GO:0060999 | positive regulation of dendritic spine development | -6.780021439 | -5.268 | 18/44 | 375,2332,2475,4076,5048,5063,5597,5649,6453,7074,9231,10458,22849,22871,22941,23613,55607,65981 | ARF1,FMR1,MTOR,CAPRIN1,PAFAH1B1,PAK3,MAPK6,RELN,ITSN1,TIAM1,DLG5,BAIAP2,CPEB3,NLGN1,SHANK2,ZMYND8,PPP1R9A,CAPRIN2 |
| 18_Member | GO Biological Processes | GO:0060997 | dendritic spine morphogenesis | -6.035576308 | -4.606 | 20/58 | 102,998,1501,2043,2048,4076,5048,5063,5649,5728,7074,7337,8851,8976,10152,10458,22871,22941,55607,65981 | ADAM10,CDC42,CTNND2,EPHA4,EPHB2,CAPRIN1,PAFAH1B1,PAK3,RELN,PTEN,TIAM1,UBE3A,CDK5R1,WASL,ABI2,BAIAP2,NLGN1,SHANK2,PPP1R9A,CAPRIN2 |
| 18_Member | GO Biological Processes | GO:0061001 | regulation of dendritic spine morphogenesis | -4.610501926 | -3.333 | 15/44 | 102,2043,4076,5048,5063,5649,5728,7074,7337,8851,10152,10458,22871,55607,65981 | ADAM10,EPHA4,CAPRIN1,PAFAH1B1,PAK3,RELN,PTEN,TIAM1,UBE3A,CDK5R1,ABI2,BAIAP2,NLGN1,PPP1R9A,CAPRIN2 |
| 18_Member | GO Biological Processes | GO:0050775 | positive regulation of dendrite morphogenesis | -3.330586947 | -2.225 | 12/39 | 2043,4076,5048,5063,5649,5789,7074,10458,11141,23011,26039,65981 | EPHA4,CAPRIN1,PAFAH1B1,PAK3,RELN,PTPRD,TIAM1,BAIAP2,IL1RAPL1,RAB21,SS18L1,CAPRIN2 |
| 19_Summary | GO Biological Processes | GO:0001944 | vasculature development | -24.56446112 | -21.938 | 183/793 | 25,90,102,163,182,196,284,387,639,655,657,659,677,688,694,1278,1281,1282,1289,1296,1386,1399,1482,1490,1499,1634,1901,1958,2004,2048,2113,2138,2185,2255,2263,2277,2294,2308,2335,2549,2626,2683,2737,3037,3082,3198,3200,3202,3491,3688,3696,3725,3815,3964,4041,4052,4086,4091,4208,4215,4223,4627,4628,4638,4643,4763,4773,4775,4851,4856,4897,4915,5125,5140,5155,5156,5168,5170,5291,5316,5469,5578,5594,5604,5613,5629,5728,5784,5797,5921,6091,6092,6093,6095,6303,6444,6654,6722,6733,6909,6934,7021,7042,7046,7048,7057,7101,7291,7424,7494,7716,7852,8289,8325,8434,8817,8828,8829,9021,9037,9314,9353,9444,9451,9475,9496,9693,9723,9734,10000,10052,10253,10427,10672,10959,11146,23213,23251,23328,23411,23414,23493,23576,23592,23670,26057,26523,27250,28514,28996,29072,54206,54567,54583,55109,55294,55636,55810,57178,57381,57493,57534,57556,57608,60626,64094,79602,79733,79805,80155,80314,83478,90627,92140,130497,144165,154810,161742,168667,196528,204851,221981,284217,92,367,650,654,687,858,1021,1027,1028,1739,1956,2081,2252,2475,2625,3146,3398,3915,4163,4781,4853,5241,5727,5796,5813,6195,6239,6431,6657,6664,6928,7421,7432,8013,8573,9788,10818,11171,11221,51804,55366,55959,57142,57521,57599,64919,79577,80004,83452,93986,253260,411,2066,2668,5048,5063,5308,5581,5586,5793,5801,5829,6546,6781,7114,8766,9448,9590,9839,9922,10507,10982,23042,23122,23189,23332,23603,26060,27302,30812,54443,64750,65979,80031,84159,130399,79977,324,351,1398,1812,1826,3643,3717,4986,5295,5420,5530,5649,5962,7074,7803,8650,8660,9043,9126,9480,9547,9732,10396,10631,22800,22919,55737,55749,56034,57118,65125,128866,158747,604,4133 | ABL1,ACVR1,ADAM10,AP2B1,JAG1,AHR,ANGPT1,RHOA,PRDM1,BMP7,BMPR1A,BMPR2,ZFP36L1,KLF5,BTG1,COL1A2,COL3A1,COL4A1,COL5A1,COL8A2,ATF2,CRKL,NKX2-5,CCN2,CTNNB1,DCN,S1PR1,EGR1,ELK3,EPHB2,ETS1,EYA1,PTK2B,FGF10,FGFR2,VEGFD,FOXF1,FOXO1,FN1,GAB1,GATA4,B4GALT1,GLI3,HAS2,HGF,HOXA1,HOXA3,HOXA5,CCN1,ITGB1,ITGB8,JUN,KIT,LGALS8,LRP5,LTBP1,SMAD1,SMAD6,MEF2C,MAP3K3,MEOX2,MYH9,MYH10,MYLK,MYO1E,NF1,NFATC2,NFATC3,NOTCH1,CCN3,NRCAM,NTRK2,PCSK5,PDE3B,PDGFB,PDGFRA,ENPP2,PDPK1,PIK3CB,PKNOX1,MED1,PRKCA,MAPK1,MAP2K1,PRKX,PROX1,PTEN,PTPN14,PTPRM,RASA1,ROBO1,ROBO2,ROCK1,RORA,SAT1,SGCD,SOS1,SRF,SRPK2,TBX2,TCF7L2,TFAP2B,TGFB2,TGFBR1,TGFBR2,THBS1,NR2E1,TWIST1,VEGFC,XBP1,VEZF1,CXCR4,ARID1A,FZD8,RECK,FGF18,NRP2,NRP1,SOCS3,SEMA5A,KLF4,SLIT2,QKI,EIF2AK3,ROCK2,TBX4,RAPGEF2,SEMA3E,HDAC9,AKT3,GJC1,SPRY2,SEC24B,GNA13,TMED2,GLMN,SULF1,MINAR1,SASH1,SIRT1,ZFPM2,HEY2,DDAH1,LEMD3,CEMIP2,ANKRD17,AGO1,PDCD4,DLL1,HIPK2,SETD2,ERRFI1,DLL4,EGLN1,AGGF1,FBXW7,CHD7,FOXJ2,ZMIZ1,RHOJ,HEG1,MIB1,SEMA6A,JCAD,RIC8A,SMOC2,ADIPOR2,E2F8,VASH2,NAA15,EPC1,ARHGAP24,STARD13,MTDH,OSR1,PRICKLE1,AMOTL1,SPRED1,BMPER,ARID2,HIPK1,THSD7A,LAMA1,ACVR2A,AR,BMP2,BMP6,KLF9,CAV2,CDK6,CDKN1B,CDKN1C,DLG1,EGFR,ERN1,FGF7,MTOR,GATA3,HMGB1,ID2,LAMC1,MCC,NFIB,NOTCH2,PGR,PTCH1,PTPRK,PURA,RPS6KA1,RREB1,SRSF6,SOX2,SOX11,HNF1B,VDR,VIP,NR4A3,CASK,MTSS1,FRS2,STRAP,DUSP10,SIX4,LGR4,SULF2,RTN4,RPTOR,WDR48,BCL11B,CDC73,ESRP2,RAB33B,FOXP2,RICTOR,ARSB,ERBB4,GDNF,PAFAH1B1,PAK3,PITX2,PRKCE,PKN2,PTPRG,PTPRR,PXN,SLC8A1,STC1,TMSB4X,RAB11A,MAP4K4,AKAP12,ZEB2,IQSEC1,SEMA4D,MAPRE2,PDXDC1,CLASP2,KANK1,CLASP1,CORO1C,APPL1,BMP10,SOX8,ANLN,SMURF2,PHACTR4,SEMA6D,ARID5B,ACVR1C,GRHL2,APC,APP,CRK,DRD1,DSCAM,INSR,JAK2,OPRK1,PIK3R1,PODXL,PPP3CA,RELN,RDX,TIAM1,PTP4A1,NUMB,IRS2,SPAG9,SMC3,ONECUT2,CXCL14,DOCK4,ATP8A1,POSTN,RRAS2,MAPRE1,VPS35,CCAR1,PDGFC,CAMK1D,WNK1,CHMP4B,MOSPD2,BCL6,MAP2 |
| 19_Member | GO Biological Processes | GO:0001944 | vasculature development | -24.56446112 | -21.938 | 183/793 | 25,90,102,163,182,196,284,387,639,655,657,659,677,688,694,1278,1281,1282,1289,1296,1386,1399,1482,1490,1499,1634,1901,1958,2004,2048,2113,2138,2185,2255,2263,2277,2294,2308,2335,2549,2626,2683,2737,3037,3082,3198,3200,3202,3491,3688,3696,3725,3815,3964,4041,4052,4086,4091,4208,4215,4223,4627,4628,4638,4643,4763,4773,4775,4851,4856,4897,4915,5125,5140,5155,5156,5168,5170,5291,5316,5469,5578,5594,5604,5613,5629,5728,5784,5797,5921,6091,6092,6093,6095,6303,6444,6654,6722,6733,6909,6934,7021,7042,7046,7048,7057,7101,7291,7424,7494,7716,7852,8289,8325,8434,8817,8828,8829,9021,9037,9314,9353,9444,9451,9475,9496,9693,9723,9734,10000,10052,10253,10427,10672,10959,11146,23213,23251,23328,23411,23414,23493,23576,23592,23670,26057,26523,27250,28514,28996,29072,54206,54567,54583,55109,55294,55636,55810,57178,57381,57493,57534,57556,57608,60626,64094,79602,79733,79805,80155,80314,83478,90627,92140,130497,144165,154810,161742,168667,196528,204851,221981,284217 | ABL1,ACVR1,ADAM10,AP2B1,JAG1,AHR,ANGPT1,RHOA,PRDM1,BMP7,BMPR1A,BMPR2,ZFP36L1,KLF5,BTG1,COL1A2,COL3A1,COL4A1,COL5A1,COL8A2,ATF2,CRKL,NKX2-5,CCN2,CTNNB1,DCN,S1PR1,EGR1,ELK3,EPHB2,ETS1,EYA1,PTK2B,FGF10,FGFR2,VEGFD,FOXF1,FOXO1,FN1,GAB1,GATA4,B4GALT1,GLI3,HAS2,HGF,HOXA1,HOXA3,HOXA5,CCN1,ITGB1,ITGB8,JUN,KIT,LGALS8,LRP5,LTBP1,SMAD1,SMAD6,MEF2C,MAP3K3,MEOX2,MYH9,MYH10,MYLK,MYO1E,NF1,NFATC2,NFATC3,NOTCH1,CCN3,NRCAM,NTRK2,PCSK5,PDE3B,PDGFB,PDGFRA,ENPP2,PDPK1,PIK3CB,PKNOX1,MED1,PRKCA,MAPK1,MAP2K1,PRKX,PROX1,PTEN,PTPN14,PTPRM,RASA1,ROBO1,ROBO2,ROCK1,RORA,SAT1,SGCD,SOS1,SRF,SRPK2,TBX2,TCF7L2,TFAP2B,TGFB2,TGFBR1,TGFBR2,THBS1,NR2E1,TWIST1,VEGFC,XBP1,VEZF1,CXCR4,ARID1A,FZD8,RECK,FGF18,NRP2,NRP1,SOCS3,SEMA5A,KLF4,SLIT2,QKI,EIF2AK3,ROCK2,TBX4,RAPGEF2,SEMA3E,HDAC9,AKT3,GJC1,SPRY2,SEC24B,GNA13,TMED2,GLMN,SULF1,MINAR1,SASH1,SIRT1,ZFPM2,HEY2,DDAH1,LEMD3,CEMIP2,ANKRD17,AGO1,PDCD4,DLL1,HIPK2,SETD2,ERRFI1,DLL4,EGLN1,AGGF1,FBXW7,CHD7,FOXJ2,ZMIZ1,RHOJ,HEG1,MIB1,SEMA6A,JCAD,RIC8A,SMOC2,ADIPOR2,E2F8,VASH2,NAA15,EPC1,ARHGAP24,STARD13,MTDH,OSR1,PRICKLE1,AMOTL1,SPRED1,BMPER,ARID2,HIPK1,THSD7A,LAMA1 |
| 19_Member | GO Biological Processes | GO:0001568 | blood vessel development | -22.40107074 | -19.926 | 173/762 | 25,90,163,182,196,284,387,639,655,657,659,677,688,694,1278,1281,1282,1289,1296,1386,1399,1482,1490,1499,1634,1901,1958,2004,2048,2113,2138,2185,2255,2263,2277,2294,2308,2335,2549,2626,2683,2737,3037,3082,3198,3200,3202,3491,3688,3696,3725,4041,4052,4086,4091,4208,4215,4223,4627,4628,4638,4643,4763,4851,4856,4897,4915,5125,5140,5155,5156,5168,5170,5291,5316,5469,5578,5594,5604,5613,5629,5728,5797,5921,6091,6092,6093,6095,6303,6444,6654,6722,6733,6909,6934,7021,7042,7046,7048,7057,7101,7291,7424,7494,7716,7852,8289,8325,8434,8817,8828,8829,9021,9037,9314,9353,9444,9451,9475,9496,9693,9723,9734,10000,10052,10253,10427,10672,10959,11146,23213,23251,23328,23411,23414,23493,23576,23592,23670,26057,26523,28514,28996,29072,54567,54583,55109,55294,55636,55810,57178,57381,57493,57534,57556,57608,64094,79602,79733,79805,80155,83478,90627,92140,130497,144165,154810,161742,168667,196528,204851,221981,284217 | ABL1,ACVR1,AP2B1,JAG1,AHR,ANGPT1,RHOA,PRDM1,BMP7,BMPR1A,BMPR2,ZFP36L1,KLF5,BTG1,COL1A2,COL3A1,COL4A1,COL5A1,COL8A2,ATF2,CRKL,NKX2-5,CCN2,CTNNB1,DCN,S1PR1,EGR1,ELK3,EPHB2,ETS1,EYA1,PTK2B,FGF10,FGFR2,VEGFD,FOXF1,FOXO1,FN1,GAB1,GATA4,B4GALT1,GLI3,HAS2,HGF,HOXA1,HOXA3,HOXA5,CCN1,ITGB1,ITGB8,JUN,LRP5,LTBP1,SMAD1,SMAD6,MEF2C,MAP3K3,MEOX2,MYH9,MYH10,MYLK,MYO1E,NF1,NOTCH1,CCN3,NRCAM,NTRK2,PCSK5,PDE3B,PDGFB,PDGFRA,ENPP2,PDPK1,PIK3CB,PKNOX1,MED1,PRKCA,MAPK1,MAP2K1,PRKX,PROX1,PTEN,PTPRM,RASA1,ROBO1,ROBO2,ROCK1,RORA,SAT1,SGCD,SOS1,SRF,SRPK2,TBX2,TCF7L2,TFAP2B,TGFB2,TGFBR1,TGFBR2,THBS1,NR2E1,TWIST1,VEGFC,XBP1,VEZF1,CXCR4,ARID1A,FZD8,RECK,FGF18,NRP2,NRP1,SOCS3,SEMA5A,KLF4,SLIT2,QKI,EIF2AK3,ROCK2,TBX4,RAPGEF2,SEMA3E,HDAC9,AKT3,GJC1,SPRY2,SEC24B,GNA13,TMED2,GLMN,SULF1,MINAR1,SASH1,SIRT1,ZFPM2,HEY2,DDAH1,LEMD3,CEMIP2,ANKRD17,AGO1,DLL1,HIPK2,SETD2,DLL4,EGLN1,AGGF1,FBXW7,CHD7,FOXJ2,ZMIZ1,RHOJ,HEG1,MIB1,SEMA6A,JCAD,SMOC2,ADIPOR2,E2F8,VASH2,NAA15,ARHGAP24,STARD13,MTDH,OSR1,PRICKLE1,AMOTL1,SPRED1,BMPER,ARID2,HIPK1,THSD7A,LAMA1 |
| 19_Member | GO Biological Processes | GO:0048514 | blood vessel morphogenesis | -16.92256717 | -14.665 | 147/682 | 25,90,182,284,387,639,657,659,677,688,694,1281,1282,1296,1386,1482,1490,1499,1634,1901,2004,2048,2113,2138,2185,2255,2263,2277,2294,2335,2549,2626,2683,3037,3082,3198,3200,3202,3491,3688,3696,3725,4041,4086,4215,4223,4627,4638,4643,4763,4851,4856,4897,4915,5140,5156,5168,5170,5291,5316,5469,5578,5613,5629,5728,5797,5921,6091,6093,6095,6303,6444,6654,6722,6733,6909,7021,7042,7046,7048,7057,7101,7291,7424,7494,7716,7852,8325,8434,8817,8828,8829,9037,9314,9353,9444,9451,9475,9496,9693,9723,9734,10000,10052,10253,10427,10672,11146,23213,23251,23328,23411,23414,23493,23576,23592,23670,26523,28514,28996,29072,54567,54583,55109,55294,55636,55810,57178,57381,57493,57556,57608,64094,79602,79733,79805,80155,83478,90627,92140,154810,161742,168667,196528,204851,221981,284217 | ABL1,ACVR1,JAG1,ANGPT1,RHOA,PRDM1,BMPR1A,BMPR2,ZFP36L1,KLF5,BTG1,COL3A1,COL4A1,COL8A2,ATF2,NKX2-5,CCN2,CTNNB1,DCN,S1PR1,ELK3,EPHB2,ETS1,EYA1,PTK2B,FGF10,FGFR2,VEGFD,FOXF1,FN1,GAB1,GATA4,B4GALT1,HAS2,HGF,HOXA1,HOXA3,HOXA5,CCN1,ITGB1,ITGB8,JUN,LRP5,SMAD1,MAP3K3,MEOX2,MYH9,MYLK,MYO1E,NF1,NOTCH1,CCN3,NRCAM,NTRK2,PDE3B,PDGFRA,ENPP2,PDPK1,PIK3CB,PKNOX1,MED1,PRKCA,PRKX,PROX1,PTEN,PTPRM,RASA1,ROBO1,ROCK1,RORA,SAT1,SGCD,SOS1,SRF,SRPK2,TBX2,TFAP2B,TGFB2,TGFBR1,TGFBR2,THBS1,NR2E1,TWIST1,VEGFC,XBP1,VEZF1,CXCR4,FZD8,RECK,FGF18,NRP2,NRP1,SEMA5A,KLF4,SLIT2,QKI,EIF2AK3,ROCK2,TBX4,RAPGEF2,SEMA3E,HDAC9,AKT3,GJC1,SPRY2,SEC24B,GNA13,GLMN,SULF1,MINAR1,SASH1,SIRT1,ZFPM2,HEY2,DDAH1,LEMD3,CEMIP2,AGO1,DLL1,HIPK2,SETD2,DLL4,EGLN1,AGGF1,FBXW7,CHD7,FOXJ2,ZMIZ1,RHOJ,HEG1,SEMA6A,JCAD,SMOC2,ADIPOR2,E2F8,VASH2,NAA15,ARHGAP24,STARD13,MTDH,AMOTL1,SPRED1,BMPER,ARID2,HIPK1,THSD7A,LAMA1 |
| 19_Member | GO Biological Processes | GO:0050673 | epithelial cell proliferation | -15.31944283 | -13.156 | 106/446 | 92,284,367,650,654,657,659,677,687,858,1021,1027,1028,1296,1386,1499,1739,1956,2081,2138,2252,2255,2263,2277,2475,2625,2683,3037,3082,3146,3202,3398,3725,3815,3915,4163,4208,4763,4781,4851,4853,4856,5155,5170,5241,5291,5469,5578,5594,5604,5613,5629,5727,5728,5796,5797,5813,6091,6195,6239,6431,6657,6664,6928,6934,7042,7046,7057,7291,7421,7424,7432,7494,8013,8573,8817,8828,8829,9037,9788,10000,10818,11171,11221,23213,23411,51804,54206,54567,55109,55294,55366,55959,57142,57521,57599,57608,64919,79577,79805,80004,83452,93986,130497,168667,253260 | ACVR2A,ANGPT1,AR,BMP2,BMP6,BMPR1A,BMPR2,ZFP36L1,KLF9,CAV2,CDK6,CDKN1B,CDKN1C,COL8A2,ATF2,CTNNB1,DLG1,EGFR,ERN1,EYA1,FGF7,FGF10,FGFR2,VEGFD,MTOR,GATA3,B4GALT1,HAS2,HGF,HMGB1,HOXA5,ID2,JUN,KIT,LAMC1,MCC,MEF2C,NF1,NFIB,NOTCH1,NOTCH2,CCN3,PDGFB,PDPK1,PGR,PIK3CB,MED1,PRKCA,MAPK1,MAP2K1,PRKX,PROX1,PTCH1,PTEN,PTPRK,PTPRM,PURA,ROBO1,RPS6KA1,RREB1,SRSF6,SOX2,SOX11,HNF1B,TCF7L2,TGFB2,TGFBR1,THBS1,TWIST1,VDR,VEGFC,VIP,XBP1,NR4A3,CASK,FGF18,NRP2,NRP1,SEMA5A,MTSS1,AKT3,FRS2,STRAP,DUSP10,SULF1,SIRT1,SIX4,ERRFI1,DLL4,AGGF1,FBXW7,LGR4,SULF2,RTN4,RPTOR,WDR48,JCAD,BCL11B,CDC73,VASH2,ESRP2,RAB33B,FOXP2,OSR1,BMPER,RICTOR |
| 19_Member | GO Biological Processes | GO:0001667 | ameboidal-type cell migration | -14.50278814 | -12.410 | 107/464 | 25,90,284,387,411,655,659,1634,2066,2113,2185,2252,2255,2335,2475,2625,2668,3037,3146,3688,3725,3815,3964,4163,4208,4215,4223,4627,4763,4851,4856,5048,5063,5155,5168,5170,5308,5578,5581,5586,5613,5629,5728,5793,5797,5801,5829,6091,6239,6546,6722,6781,7042,7046,7048,7057,7101,7114,7291,7424,8766,8817,8828,8829,9037,9314,9353,9448,9475,9590,9723,9734,9839,9922,10000,10507,10982,11171,11221,23042,23122,23189,23328,23332,23411,23603,26060,27302,30812,54443,54567,55294,57142,57381,57556,57608,60626,64094,64750,65979,80031,84159,90627,130399,154810,161742,168667 | ABL1,ACVR1,ANGPT1,RHOA,ARSB,BMP7,BMPR2,DCN,ERBB4,ETS1,PTK2B,FGF7,FGF10,FN1,MTOR,GATA3,GDNF,HAS2,HMGB1,ITGB1,JUN,KIT,LGALS8,MCC,MEF2C,MAP3K3,MEOX2,MYH9,NF1,NOTCH1,CCN3,PAFAH1B1,PAK3,PDGFB,ENPP2,PDPK1,PITX2,PRKCA,PRKCE,PKN2,PRKX,PROX1,PTEN,PTPRG,PTPRM,PTPRR,PXN,ROBO1,RREB1,SLC8A1,SRF,STC1,TGFB2,TGFBR1,TGFBR2,THBS1,NR2E1,TMSB4X,TWIST1,VEGFC,RAB11A,FGF18,NRP2,NRP1,SEMA5A,KLF4,SLIT2,MAP4K4,ROCK2,AKAP12,SEMA3E,HDAC9,ZEB2,IQSEC1,AKT3,SEMA4D,MAPRE2,STRAP,DUSP10,PDXDC1,CLASP2,KANK1,SASH1,CLASP1,SIRT1,CORO1C,APPL1,BMP10,SOX8,ANLN,DLL4,FBXW7,RTN4,RHOJ,SEMA6A,JCAD,RIC8A,SMOC2,SMURF2,PHACTR4,SEMA6D,ARID5B,STARD13,ACVR1C,AMOTL1,SPRED1,BMPER |
| 19_Member | GO Biological Processes | GO:0090130 | tissue migration | -12.53782349 | -10.569 | 86/362 | 25,284,387,411,659,1634,2113,2185,2252,2255,2294,2475,2625,3037,3146,3688,3725,3815,3964,4163,4208,4215,4223,4627,4763,4851,4856,5155,5168,5170,5578,5581,5586,5613,5629,5728,5793,5797,5801,5829,6091,6239,6722,6781,7042,7046,7048,7057,7101,7114,7424,8766,8817,8828,8829,9037,9314,9353,9448,9475,9734,9839,9922,10000,10982,11171,11221,23122,23189,23328,23332,23411,23603,27302,54443,54567,55294,57142,57381,57608,64094,79977,90627,154810,161742,168667 | ABL1,ANGPT1,RHOA,ARSB,BMPR2,DCN,ETS1,PTK2B,FGF7,FGF10,FOXF1,MTOR,GATA3,HAS2,HMGB1,ITGB1,JUN,KIT,LGALS8,MCC,MEF2C,MAP3K3,MEOX2,MYH9,NF1,NOTCH1,CCN3,PDGFB,ENPP2,PDPK1,PRKCA,PRKCE,PKN2,PRKX,PROX1,PTEN,PTPRG,PTPRM,PTPRR,PXN,ROBO1,RREB1,SRF,STC1,TGFB2,TGFBR1,TGFBR2,THBS1,NR2E1,TMSB4X,VEGFC,RAB11A,FGF18,NRP2,NRP1,SEMA5A,KLF4,SLIT2,MAP4K4,ROCK2,HDAC9,ZEB2,IQSEC1,AKT3,MAPRE2,STRAP,DUSP10,CLASP2,KANK1,SASH1,CLASP1,SIRT1,CORO1C,BMP10,ANLN,DLL4,FBXW7,RTN4,RHOJ,JCAD,SMOC2,GRHL2,STARD13,AMOTL1,SPRED1,BMPER |
| 19_Member | GO Biological Processes | GO:0090132 | epithelium migration | -12.52782666 | -10.563 | 85/356 | 25,284,387,411,659,1634,2113,2185,2252,2255,2475,2625,3037,3146,3688,3725,3815,3964,4163,4208,4215,4223,4627,4763,4851,4856,5155,5168,5170,5578,5581,5586,5613,5629,5728,5793,5797,5801,5829,6091,6239,6722,6781,7042,7046,7048,7057,7101,7114,7424,8766,8817,8828,8829,9037,9314,9353,9448,9475,9734,9839,9922,10000,10982,11171,11221,23122,23189,23328,23332,23411,23603,27302,54443,54567,55294,57142,57381,57608,64094,79977,90627,154810,161742,168667 | ABL1,ANGPT1,RHOA,ARSB,BMPR2,DCN,ETS1,PTK2B,FGF7,FGF10,MTOR,GATA3,HAS2,HMGB1,ITGB1,JUN,KIT,LGALS8,MCC,MEF2C,MAP3K3,MEOX2,MYH9,NF1,NOTCH1,CCN3,PDGFB,ENPP2,PDPK1,PRKCA,PRKCE,PKN2,PRKX,PROX1,PTEN,PTPRG,PTPRM,PTPRR,PXN,ROBO1,RREB1,SRF,STC1,TGFB2,TGFBR1,TGFBR2,THBS1,NR2E1,TMSB4X,VEGFC,RAB11A,FGF18,NRP2,NRP1,SEMA5A,KLF4,SLIT2,MAP4K4,ROCK2,HDAC9,ZEB2,IQSEC1,AKT3,MAPRE2,STRAP,DUSP10,CLASP2,KANK1,SASH1,CLASP1,SIRT1,CORO1C,BMP10,ANLN,DLL4,FBXW7,RTN4,RHOJ,JCAD,SMOC2,GRHL2,STARD13,AMOTL1,SPRED1,BMPER |
| 19_Member | GO Biological Processes | GO:0050678 | regulation of epithelial cell proliferation | -12.45282022 | -10.493 | 90/388 | 284,367,650,654,657,659,677,687,858,1021,1027,1028,1386,1499,1739,1956,2138,2252,2255,2263,2277,2475,2625,2683,3037,3146,3202,3725,3915,4163,4208,4763,4781,4851,4853,5155,5170,5241,5469,5578,5629,5727,5728,5796,5797,6091,6239,6431,6657,6664,6934,7042,7046,7057,7291,7421,7424,7432,7494,8013,8573,8817,8828,8829,9037,9788,10000,10818,11171,11221,23213,23411,51804,54206,54567,55109,55294,55959,57142,57521,57599,57608,64919,79577,79805,80004,83452,93986,130497,253260 | ANGPT1,AR,BMP2,BMP6,BMPR1A,BMPR2,ZFP36L1,KLF9,CAV2,CDK6,CDKN1B,CDKN1C,ATF2,CTNNB1,DLG1,EGFR,EYA1,FGF7,FGF10,FGFR2,VEGFD,MTOR,GATA3,B4GALT1,HAS2,HMGB1,HOXA5,JUN,LAMC1,MCC,MEF2C,NF1,NFIB,NOTCH1,NOTCH2,PDGFB,PDPK1,PGR,MED1,PRKCA,PROX1,PTCH1,PTEN,PTPRK,PTPRM,ROBO1,RREB1,SRSF6,SOX2,SOX11,TCF7L2,TGFB2,TGFBR1,THBS1,TWIST1,VDR,VEGFC,VIP,XBP1,NR4A3,CASK,FGF18,NRP2,NRP1,SEMA5A,MTSS1,AKT3,FRS2,STRAP,DUSP10,SULF1,SIRT1,SIX4,ERRFI1,DLL4,AGGF1,FBXW7,SULF2,RTN4,RPTOR,WDR48,JCAD,BCL11B,CDC73,VASH2,ESRP2,RAB33B,FOXP2,OSR1,RICTOR |
| 19_Member | GO Biological Processes | GO:0010631 | epithelial cell migration | -12.30326338 | -10.356 | 84/353 | 25,284,387,411,659,1634,2113,2185,2252,2255,2475,2625,3037,3146,3688,3725,3815,3964,4163,4208,4215,4223,4627,4763,4851,4856,5155,5168,5170,5578,5581,5586,5613,5629,5728,5793,5797,5801,5829,6091,6239,6722,6781,7042,7046,7048,7057,7101,7114,7424,8766,8817,8828,8829,9037,9314,9353,9448,9475,9734,9839,9922,10000,10982,11171,11221,23122,23189,23328,23332,23411,23603,27302,54443,54567,55294,57142,57381,57608,64094,90627,154810,161742,168667 | ABL1,ANGPT1,RHOA,ARSB,BMPR2,DCN,ETS1,PTK2B,FGF7,FGF10,MTOR,GATA3,HAS2,HMGB1,ITGB1,JUN,KIT,LGALS8,MCC,MEF2C,MAP3K3,MEOX2,MYH9,NF1,NOTCH1,CCN3,PDGFB,ENPP2,PDPK1,PRKCA,PRKCE,PKN2,PRKX,PROX1,PTEN,PTPRG,PTPRM,PTPRR,PXN,ROBO1,RREB1,SRF,STC1,TGFB2,TGFBR1,TGFBR2,THBS1,NR2E1,TMSB4X,VEGFC,RAB11A,FGF18,NRP2,NRP1,SEMA5A,KLF4,SLIT2,MAP4K4,ROCK2,HDAC9,ZEB2,IQSEC1,AKT3,MAPRE2,STRAP,DUSP10,CLASP2,KANK1,SASH1,CLASP1,SIRT1,CORO1C,BMP10,ANLN,DLL4,FBXW7,RTN4,RHOJ,JCAD,SMOC2,STARD13,AMOTL1,SPRED1,BMPER |
| 19_Member | GO Biological Processes | GO:0010632 | regulation of epithelial cell migration | -10.52348296 | -8.687 | 70/292 | 25,284,387,411,659,1634,2113,2185,2252,2255,2475,2625,3037,3146,3725,4163,4208,4215,4223,4763,4851,5155,5168,5170,5578,5581,5629,5728,5793,5797,5801,6239,6781,7042,7048,7057,7101,7114,7424,8766,8817,8828,8829,9037,9314,9353,9448,9475,9734,9922,10000,10982,11171,11221,23122,23328,23332,23411,23603,27302,54567,55294,57142,57381,57608,64094,90627,154810,161742,168667 | ABL1,ANGPT1,RHOA,ARSB,BMPR2,DCN,ETS1,PTK2B,FGF7,FGF10,MTOR,GATA3,HAS2,HMGB1,JUN,MCC,MEF2C,MAP3K3,MEOX2,NF1,NOTCH1,PDGFB,ENPP2,PDPK1,PRKCA,PRKCE,PROX1,PTEN,PTPRG,PTPRM,PTPRR,RREB1,STC1,TGFB2,TGFBR2,THBS1,NR2E1,TMSB4X,VEGFC,RAB11A,FGF18,NRP2,NRP1,SEMA5A,KLF4,SLIT2,MAP4K4,ROCK2,HDAC9,IQSEC1,AKT3,MAPRE2,STRAP,DUSP10,CLASP2,SASH1,CLASP1,SIRT1,CORO1C,BMP10,DLL4,FBXW7,RTN4,RHOJ,JCAD,SMOC2,STARD13,AMOTL1,SPRED1,BMPER |
| 19_Member | GO Biological Processes | GO:0001525 | angiogenesis | -10.44737972 | -8.621 | 115/588 | 25,90,182,284,387,688,694,1282,1296,1386,1490,1499,1634,1901,2004,2048,2113,2185,2255,2263,2277,2335,2549,2626,2683,3082,3200,3202,3491,3688,3696,3725,4086,4215,4223,4627,4763,4851,4856,4897,5140,5156,5168,5170,5291,5316,5469,5578,5613,5728,5797,6091,6093,6095,6303,6722,6733,7042,7046,7048,7057,7101,7291,7424,7494,7716,7852,8325,8434,8817,8828,8829,9037,9314,9353,9451,9475,9496,9723,9734,10000,10253,10672,23213,23251,23328,23411,23576,23592,23670,26523,28514,28996,29072,54567,54583,55109,55294,55810,57381,57556,57608,64094,79602,79733,79805,80155,83478,90627,92140,154810,161742,168667,204851,221981 | ABL1,ACVR1,JAG1,ANGPT1,RHOA,KLF5,BTG1,COL4A1,COL8A2,ATF2,CCN2,CTNNB1,DCN,S1PR1,ELK3,EPHB2,ETS1,PTK2B,FGF10,FGFR2,VEGFD,FN1,GAB1,GATA4,B4GALT1,HGF,HOXA3,HOXA5,CCN1,ITGB1,ITGB8,JUN,SMAD1,MAP3K3,MEOX2,MYH9,NF1,NOTCH1,CCN3,NRCAM,PDE3B,PDGFRA,ENPP2,PDPK1,PIK3CB,PKNOX1,MED1,PRKCA,PRKX,PTEN,PTPRM,ROBO1,ROCK1,RORA,SAT1,SRF,SRPK2,TGFB2,TGFBR1,TGFBR2,THBS1,NR2E1,TWIST1,VEGFC,XBP1,VEZF1,CXCR4,FZD8,RECK,FGF18,NRP2,NRP1,SEMA5A,KLF4,SLIT2,EIF2AK3,ROCK2,TBX4,SEMA3E,HDAC9,AKT3,SPRY2,GNA13,SULF1,MINAR1,SASH1,SIRT1,DDAH1,LEMD3,CEMIP2,AGO1,DLL1,HIPK2,SETD2,DLL4,EGLN1,AGGF1,FBXW7,FOXJ2,RHOJ,SEMA6A,JCAD,SMOC2,ADIPOR2,E2F8,VASH2,NAA15,ARHGAP24,STARD13,MTDH,AMOTL1,SPRED1,BMPER,HIPK1,THSD7A |
| 19_Member | GO Biological Processes | GO:0040017 | positive regulation of locomotion | -9.976379947 | -8.181 | 115/598 | 25,90,102,284,324,351,387,650,655,659,858,1398,1399,1812,1826,1901,1956,2113,2185,2252,2255,2277,2294,2335,2475,2549,2625,3037,3082,3146,3491,3643,3688,3717,3725,3815,4215,4638,4851,4986,5063,5155,5156,5168,5170,5295,5420,5530,5578,5581,5594,5629,5649,5962,6239,6546,7042,7046,7048,7057,7074,7114,7291,7424,7494,7803,7852,8013,8650,8660,8766,8817,8828,8829,9037,9043,9126,9353,9448,9475,9480,9547,9590,9693,9723,9732,9734,9922,10000,10253,10396,10507,10631,10982,22800,22919,23122,23328,23332,23411,55737,55749,56034,57118,57142,57381,57556,57608,64094,64750,65125,80031,128866,154810,158747 | ABL1,ACVR1,ADAM10,ANGPT1,APC,APP,RHOA,BMP2,BMP7,BMPR2,CAV2,CRK,CRKL,DRD1,DSCAM,S1PR1,EGFR,ETS1,PTK2B,FGF7,FGF10,VEGFD,FOXF1,FN1,MTOR,GAB1,GATA3,HAS2,HGF,HMGB1,CCN1,INSR,ITGB1,JAK2,JUN,KIT,MAP3K3,MYLK,NOTCH1,OPRK1,PAK3,PDGFB,PDGFRA,ENPP2,PDPK1,PIK3R1,PODXL,PPP3CA,PRKCA,PRKCE,MAPK1,PROX1,RELN,RDX,RREB1,SLC8A1,TGFB2,TGFBR1,TGFBR2,THBS1,TIAM1,TMSB4X,TWIST1,VEGFC,XBP1,PTP4A1,CXCR4,NR4A3,NUMB,IRS2,RAB11A,FGF18,NRP2,NRP1,SEMA5A,SPAG9,SMC3,SLIT2,MAP4K4,ROCK2,ONECUT2,CXCL14,AKAP12,RAPGEF2,SEMA3E,DOCK4,HDAC9,IQSEC1,AKT3,SPRY2,ATP8A1,SEMA4D,POSTN,MAPRE2,RRAS2,MAPRE1,CLASP2,SASH1,CLASP1,SIRT1,VPS35,CCAR1,PDGFC,CAMK1D,RTN4,RHOJ,SEMA6A,JCAD,SMOC2,SMURF2,WNK1,SEMA6D,CHMP4B,AMOTL1,MOSPD2 |
| 19_Member | GO Biological Processes | GO:0030335 | positive regulation of cell migration | -9.913084882 | -8.124 | 107/544 | 25,90,102,284,324,351,387,650,655,659,1398,1399,1812,1901,1956,2113,2185,2252,2255,2277,2294,2335,2475,2549,2625,3037,3082,3146,3491,3643,3688,3717,3725,3815,4215,4638,4851,5063,5155,5156,5168,5170,5295,5420,5530,5578,5581,5594,5629,5649,5962,6239,6546,7042,7046,7048,7057,7074,7114,7424,7494,7803,7852,8013,8650,8660,8766,8817,8828,8829,9037,9043,9448,9475,9480,9547,9590,9693,9723,9732,9734,9922,10000,10253,10396,10507,10631,10982,22800,22919,23122,23328,23332,23411,55749,56034,57118,57142,57381,57556,57608,64094,64750,65125,80031,154810,158747 | ABL1,ACVR1,ADAM10,ANGPT1,APC,APP,RHOA,BMP2,BMP7,BMPR2,CRK,CRKL,DRD1,S1PR1,EGFR,ETS1,PTK2B,FGF7,FGF10,VEGFD,FOXF1,FN1,MTOR,GAB1,GATA3,HAS2,HGF,HMGB1,CCN1,INSR,ITGB1,JAK2,JUN,KIT,MAP3K3,MYLK,NOTCH1,PAK3,PDGFB,PDGFRA,ENPP2,PDPK1,PIK3R1,PODXL,PPP3CA,PRKCA,PRKCE,MAPK1,PROX1,RELN,RDX,RREB1,SLC8A1,TGFB2,TGFBR1,TGFBR2,THBS1,TIAM1,TMSB4X,VEGFC,XBP1,PTP4A1,CXCR4,NR4A3,NUMB,IRS2,RAB11A,FGF18,NRP2,NRP1,SEMA5A,SPAG9,MAP4K4,ROCK2,ONECUT2,CXCL14,AKAP12,RAPGEF2,SEMA3E,DOCK4,HDAC9,IQSEC1,AKT3,SPRY2,ATP8A1,SEMA4D,POSTN,MAPRE2,RRAS2,MAPRE1,CLASP2,SASH1,CLASP1,SIRT1,CCAR1,PDGFC,CAMK1D,RTN4,RHOJ,SEMA6A,JCAD,SMOC2,SMURF2,WNK1,SEMA6D,AMOTL1,MOSPD2 |
| 19_Member | GO Biological Processes | GO:0051272 | positive regulation of cellular component movement | -9.295776261 | -7.552 | 111/585 | 25,90,102,284,324,351,387,604,650,655,659,1398,1399,1812,1826,1901,1956,2113,2185,2252,2255,2277,2294,2335,2475,2549,2625,3037,3082,3146,3491,3643,3688,3717,3725,3815,4133,4215,4638,4851,5063,5155,5156,5168,5170,5295,5420,5530,5578,5581,5594,5629,5649,5962,6239,6546,7042,7046,7048,7057,7074,7114,7291,7424,7494,7803,7852,8013,8650,8660,8766,8817,8828,8829,9037,9043,9448,9475,9480,9547,9590,9693,9723,9732,9734,9922,10000,10253,10396,10507,10631,10982,22800,22919,23122,23328,23332,23411,55749,56034,57118,57142,57381,57556,57608,64094,64750,65125,80031,154810,158747 | ABL1,ACVR1,ADAM10,ANGPT1,APC,APP,RHOA,BCL6,BMP2,BMP7,BMPR2,CRK,CRKL,DRD1,DSCAM,S1PR1,EGFR,ETS1,PTK2B,FGF7,FGF10,VEGFD,FOXF1,FN1,MTOR,GAB1,GATA3,HAS2,HGF,HMGB1,CCN1,INSR,ITGB1,JAK2,JUN,KIT,MAP2,MAP3K3,MYLK,NOTCH1,PAK3,PDGFB,PDGFRA,ENPP2,PDPK1,PIK3R1,PODXL,PPP3CA,PRKCA,PRKCE,MAPK1,PROX1,RELN,RDX,RREB1,SLC8A1,TGFB2,TGFBR1,TGFBR2,THBS1,TIAM1,TMSB4X,TWIST1,VEGFC,XBP1,PTP4A1,CXCR4,NR4A3,NUMB,IRS2,RAB11A,FGF18,NRP2,NRP1,SEMA5A,SPAG9,MAP4K4,ROCK2,ONECUT2,CXCL14,AKAP12,RAPGEF2,SEMA3E,DOCK4,HDAC9,IQSEC1,AKT3,SPRY2,ATP8A1,SEMA4D,POSTN,MAPRE2,RRAS2,MAPRE1,CLASP2,SASH1,CLASP1,SIRT1,CCAR1,PDGFC,CAMK1D,RTN4,RHOJ,SEMA6A,JCAD,SMOC2,SMURF2,WNK1,SEMA6D,AMOTL1,MOSPD2 |
| 19_Member | GO Biological Processes | GO:2000147 | positive regulation of cell motility | -9.114969055 | -7.382 | 108/568 | 25,90,102,284,324,351,387,650,655,659,1398,1399,1812,1901,1956,2113,2185,2252,2255,2277,2294,2335,2475,2549,2625,3037,3082,3146,3491,3643,3688,3717,3725,3815,4215,4638,4851,5063,5155,5156,5168,5170,5295,5420,5530,5578,5581,5594,5629,5649,5962,6239,6546,7042,7046,7048,7057,7074,7114,7291,7424,7494,7803,7852,8013,8650,8660,8766,8817,8828,8829,9037,9043,9448,9475,9480,9547,9590,9693,9723,9732,9734,9922,10000,10253,10396,10507,10631,10982,22800,22919,23122,23328,23332,23411,55749,56034,57118,57142,57381,57556,57608,64094,64750,65125,80031,154810,158747 | ABL1,ACVR1,ADAM10,ANGPT1,APC,APP,RHOA,BMP2,BMP7,BMPR2,CRK,CRKL,DRD1,S1PR1,EGFR,ETS1,PTK2B,FGF7,FGF10,VEGFD,FOXF1,FN1,MTOR,GAB1,GATA3,HAS2,HGF,HMGB1,CCN1,INSR,ITGB1,JAK2,JUN,KIT,MAP3K3,MYLK,NOTCH1,PAK3,PDGFB,PDGFRA,ENPP2,PDPK1,PIK3R1,PODXL,PPP3CA,PRKCA,PRKCE,MAPK1,PROX1,RELN,RDX,RREB1,SLC8A1,TGFB2,TGFBR1,TGFBR2,THBS1,TIAM1,TMSB4X,TWIST1,VEGFC,XBP1,PTP4A1,CXCR4,NR4A3,NUMB,IRS2,RAB11A,FGF18,NRP2,NRP1,SEMA5A,SPAG9,MAP4K4,ROCK2,ONECUT2,CXCL14,AKAP12,RAPGEF2,SEMA3E,DOCK4,HDAC9,IQSEC1,AKT3,SPRY2,ATP8A1,SEMA4D,POSTN,MAPRE2,RRAS2,MAPRE1,CLASP2,SASH1,CLASP1,SIRT1,CCAR1,PDGFC,CAMK1D,RTN4,RHOJ,SEMA6A,JCAD,SMOC2,SMURF2,WNK1,SEMA6D,AMOTL1,MOSPD2 |
| 19_Member | GO Biological Processes | GO:0050679 | positive regulation of epithelial cell proliferation | -8.829446701 | -7.127 | 52/206 | 367,650,654,657,659,858,1499,1956,2138,2252,2255,2263,2277,2475,2683,3037,3146,3725,3915,4763,4851,4853,5155,5170,5469,5578,5629,6239,6431,6664,6934,7046,7291,7424,7432,7494,8013,8828,8829,9037,10000,23411,55109,57142,57521,57599,57608,79805,80004,93986,130497,253260 | AR,BMP2,BMP6,BMPR1A,BMPR2,CAV2,CTNNB1,EGFR,EYA1,FGF7,FGF10,FGFR2,VEGFD,MTOR,B4GALT1,HAS2,HMGB1,JUN,LAMC1,NF1,NOTCH1,NOTCH2,PDGFB,PDPK1,MED1,PRKCA,PROX1,RREB1,SRSF6,SOX11,TCF7L2,TGFBR1,TWIST1,VEGFC,VIP,XBP1,NR4A3,NRP2,NRP1,SEMA5A,AKT3,SIRT1,AGGF1,RTN4,RPTOR,WDR48,JCAD,VASH2,ESRP2,FOXP2,OSR1,RICTOR |
| 19_Member | GO Biological Processes | GO:0010634 | positive regulation of epithelial cell migration | -8.253241542 | -6.606 | 45/172 | 25,284,659,2113,2185,2252,2255,2475,2625,3037,3146,3725,4215,5155,5168,5170,5578,5581,5629,6239,7042,7048,7057,7114,7424,8766,8817,8828,8829,9037,9448,9475,9734,9922,10000,10982,23122,23328,23332,23411,57142,57381,57608,64094,154810 | ABL1,ANGPT1,BMPR2,ETS1,PTK2B,FGF7,FGF10,MTOR,GATA3,HAS2,HMGB1,JUN,MAP3K3,PDGFB,ENPP2,PDPK1,PRKCA,PRKCE,PROX1,RREB1,TGFB2,TGFBR2,THBS1,TMSB4X,VEGFC,RAB11A,FGF18,NRP2,NRP1,SEMA5A,MAP4K4,ROCK2,HDAC9,IQSEC1,AKT3,MAPRE2,CLASP2,SASH1,CLASP1,SIRT1,RTN4,RHOJ,JCAD,SMOC2,AMOTL1 |
| 19_Member | GO Biological Processes | GO:1901342 | regulation of vasculature development | -6.169948146 | -4.726 | 78/424 | 25,102,387,655,694,1386,1499,1634,1958,2113,2185,2277,2549,2626,3082,3202,3688,3696,3815,4086,4215,4223,4763,4773,4775,4851,5140,5155,5168,5170,5578,5797,6093,7042,7048,7057,7101,7291,7424,7494,7852,8434,8817,8829,9037,9314,9475,9693,9723,9734,10000,10253,23213,23251,23328,23411,23493,23576,23670,26523,27250,28514,28996,54567,54583,55109,55294,55810,57381,57556,57608,64094,79805,90627,92140,161742,168667,204851 | ABL1,ADAM10,RHOA,BMP7,BTG1,ATF2,CTNNB1,DCN,EGR1,ETS1,PTK2B,VEGFD,GAB1,GATA4,HGF,HOXA5,ITGB1,ITGB8,KIT,SMAD1,MAP3K3,MEOX2,NF1,NFATC2,NFATC3,NOTCH1,PDE3B,PDGFB,ENPP2,PDPK1,PRKCA,PTPRM,ROCK1,TGFB2,TGFBR2,THBS1,NR2E1,TWIST1,VEGFC,XBP1,CXCR4,RECK,FGF18,NRP1,SEMA5A,KLF4,ROCK2,RAPGEF2,SEMA3E,HDAC9,AKT3,SPRY2,SULF1,MINAR1,SASH1,SIRT1,HEY2,DDAH1,CEMIP2,AGO1,PDCD4,DLL1,HIPK2,DLL4,EGLN1,AGGF1,FBXW7,FOXJ2,RHOJ,SEMA6A,JCAD,SMOC2,VASH2,STARD13,MTDH,SPRED1,BMPER,HIPK1 |
| 19_Member | GO Biological Processes | GO:0043542 | endothelial cell migration | -5.655134245 | -4.270 | 55/275 | 25,284,387,659,1634,2113,2185,2625,3146,3688,3964,4208,4215,4223,4627,4763,4851,4856,5155,5170,5578,5613,5629,5728,5797,5829,6091,6722,6781,7046,7057,7101,7114,7424,8817,8828,8829,9037,9314,9353,9475,9734,10000,23328,23411,27302,54567,55294,57381,57608,64094,90627,154810,161742,168667 | ABL1,ANGPT1,RHOA,BMPR2,DCN,ETS1,PTK2B,GATA3,HMGB1,ITGB1,LGALS8,MEF2C,MAP3K3,MEOX2,MYH9,NF1,NOTCH1,CCN3,PDGFB,PDPK1,PRKCA,PRKX,PROX1,PTEN,PTPRM,PXN,ROBO1,SRF,STC1,TGFBR1,THBS1,NR2E1,TMSB4X,VEGFC,FGF18,NRP2,NRP1,SEMA5A,KLF4,SLIT2,ROCK2,HDAC9,AKT3,SASH1,SIRT1,BMP10,DLL4,FBXW7,RHOJ,JCAD,SMOC2,STARD13,AMOTL1,SPRED1,BMPER |
| 19_Member | GO Biological Processes | GO:0045765 | regulation of angiogenesis | -4.891448772 | -3.585 | 68/384 | 25,387,694,1386,1499,1634,2113,2185,2277,2549,2626,3082,3202,3688,3696,4086,4215,4223,4763,4851,5140,5168,5170,5578,5797,6093,7042,7048,7057,7101,7291,7424,7494,7852,8434,8817,8829,9037,9314,9475,9723,9734,10000,10253,23213,23251,23328,23411,23576,23670,26523,28514,28996,54567,54583,55109,55294,55810,57381,57556,57608,64094,79805,90627,92140,161742,168667,204851 | ABL1,RHOA,BTG1,ATF2,CTNNB1,DCN,ETS1,PTK2B,VEGFD,GAB1,GATA4,HGF,HOXA5,ITGB1,ITGB8,SMAD1,MAP3K3,MEOX2,NF1,NOTCH1,PDE3B,ENPP2,PDPK1,PRKCA,PTPRM,ROCK1,TGFB2,TGFBR2,THBS1,NR2E1,TWIST1,VEGFC,XBP1,CXCR4,RECK,FGF18,NRP1,SEMA5A,KLF4,ROCK2,SEMA3E,HDAC9,AKT3,SPRY2,SULF1,MINAR1,SASH1,SIRT1,DDAH1,CEMIP2,AGO1,DLL1,HIPK2,DLL4,EGLN1,AGGF1,FBXW7,FOXJ2,RHOJ,SEMA6A,JCAD,SMOC2,VASH2,STARD13,MTDH,SPRED1,BMPER,HIPK1 |
| 19_Member | GO Biological Processes | GO:0010594 | regulation of endothelial cell migration | -4.502898613 | -3.236 | 45/230 | 25,284,387,659,1634,2113,2185,2625,3146,4208,4215,4223,4763,4851,5155,5170,5578,5629,5797,6781,7057,7101,7114,7424,8817,8828,8829,9037,9314,9353,9475,9734,10000,23328,23411,27302,54567,55294,57381,57608,64094,90627,154810,161742,168667 | ABL1,ANGPT1,RHOA,BMPR2,DCN,ETS1,PTK2B,GATA3,HMGB1,MEF2C,MAP3K3,MEOX2,NF1,NOTCH1,PDGFB,PDPK1,PRKCA,PROX1,PTPRM,STC1,THBS1,NR2E1,TMSB4X,VEGFC,FGF18,NRP2,NRP1,SEMA5A,KLF4,SLIT2,ROCK2,HDAC9,AKT3,SASH1,SIRT1,BMP10,DLL4,FBXW7,RHOJ,JCAD,SMOC2,STARD13,AMOTL1,SPRED1,BMPER |
| 19_Member | GO Biological Processes | GO:1904018 | positive regulation of vasculature development | -4.502898613 | -3.236 | 45/230 | 25,694,1958,2113,2185,2277,2549,2626,3082,3688,3696,3815,4086,4215,4851,5155,5170,5578,7048,7057,7101,7291,7424,7494,7852,8817,8829,9037,9314,9693,9734,10000,23328,23411,23576,28514,28996,55109,57381,57608,64094,79805,92140,168667,204851 | ABL1,BTG1,EGR1,ETS1,PTK2B,VEGFD,GAB1,GATA4,HGF,ITGB1,ITGB8,KIT,SMAD1,MAP3K3,NOTCH1,PDGFB,PDPK1,PRKCA,TGFBR2,THBS1,NR2E1,TWIST1,VEGFC,XBP1,CXCR4,FGF18,NRP1,SEMA5A,KLF4,RAPGEF2,HDAC9,AKT3,SASH1,SIRT1,DDAH1,DLL1,HIPK2,AGGF1,RHOJ,JCAD,SMOC2,VASH2,MTDH,BMPER,HIPK1 |
| 19_Member | GO Biological Processes | GO:0045766 | positive regulation of angiogenesis | -4.097379437 | -2.884 | 40/204 | 25,694,2113,2185,2277,2549,2626,3082,3688,3696,4086,4215,5170,5578,7048,7057,7101,7291,7424,7494,7852,8817,8829,9037,9314,9734,10000,23328,23411,23576,28514,28996,55109,57381,57608,64094,79805,92140,168667,204851 | ABL1,BTG1,ETS1,PTK2B,VEGFD,GAB1,GATA4,HGF,ITGB1,ITGB8,SMAD1,MAP3K3,PDPK1,PRKCA,TGFBR2,THBS1,NR2E1,TWIST1,VEGFC,XBP1,CXCR4,FGF18,NRP1,SEMA5A,KLF4,HDAC9,AKT3,SASH1,SIRT1,DDAH1,DLL1,HIPK2,AGGF1,RHOJ,JCAD,SMOC2,VASH2,MTDH,BMPER,HIPK1 |
| 19_Member | GO Biological Processes | GO:0001938 | positive regulation of endothelial cell proliferation | -4.087048281 | -2.879 | 26/112 | 650,654,659,858,2277,2475,3146,3725,4763,5155,5170,5578,5629,7046,7424,7432,8828,8829,9037,10000,23411,55109,57521,57608,79805,253260 | BMP2,BMP6,BMPR2,CAV2,VEGFD,MTOR,HMGB1,JUN,NF1,PDGFB,PDPK1,PRKCA,PROX1,TGFBR1,VEGFC,VIP,NRP2,NRP1,SEMA5A,AKT3,SIRT1,AGGF1,RPTOR,JCAD,VASH2,RICTOR |
| 19_Member | GO Biological Processes | GO:0001935 | endothelial cell proliferation | -4.006351629 | -2.811 | 39/199 | 284,650,654,659,858,1739,2081,2277,2475,3146,3725,4208,4763,5155,5170,5291,5578,5613,5629,5797,7046,7057,7424,7432,7494,8817,8828,8829,9037,10000,23213,23411,54567,55109,57521,57608,79805,168667,253260 | ANGPT1,BMP2,BMP6,BMPR2,CAV2,DLG1,ERN1,VEGFD,MTOR,HMGB1,JUN,MEF2C,NF1,PDGFB,PDPK1,PIK3CB,PRKCA,PRKX,PROX1,PTPRM,TGFBR1,THBS1,VEGFC,VIP,XBP1,FGF18,NRP2,NRP1,SEMA5A,AKT3,SULF1,SIRT1,DLL4,AGGF1,RPTOR,JCAD,VASH2,BMPER,RICTOR |
| 19_Member | GO Biological Processes | GO:0043534 | blood vessel endothelial cell migration | -3.881267525 | -2.699 | 36/181 | 25,284,387,2113,2185,3146,3688,4208,4215,4223,4627,4763,4851,5155,5170,5578,6091,6722,7057,7101,7114,7424,8817,8829,9314,9353,9734,10000,23411,54567,55294,57381,57608,90627,154810,161742 | ABL1,ANGPT1,RHOA,ETS1,PTK2B,HMGB1,ITGB1,MEF2C,MAP3K3,MEOX2,MYH9,NF1,NOTCH1,PDGFB,PDPK1,PRKCA,ROBO1,SRF,THBS1,NR2E1,TMSB4X,VEGFC,FGF18,NRP1,KLF4,SLIT2,HDAC9,AKT3,SIRT1,DLL4,FBXW7,RHOJ,JCAD,STARD13,AMOTL1,SPRED1 |
| 19_Member | GO Biological Processes | GO:0010595 | positive regulation of endothelial cell migration | -3.811287741 | -2.643 | 28/129 | 25,284,659,2113,2185,2625,3146,4215,5155,5170,5578,5629,7057,7114,7424,8817,8828,8829,9037,9475,9734,10000,23328,23411,57381,57608,64094,154810 | ABL1,ANGPT1,BMPR2,ETS1,PTK2B,GATA3,HMGB1,MAP3K3,PDGFB,PDPK1,PRKCA,PROX1,THBS1,TMSB4X,VEGFC,FGF18,NRP2,NRP1,SEMA5A,ROCK2,HDAC9,AKT3,SASH1,SIRT1,RHOJ,JCAD,SMOC2,AMOTL1 |
| 19_Member | GO Biological Processes | GO:0002040 | sprouting angiogenesis | -3.636560796 | -2.491 | 36/186 | 25,284,387,2185,2277,3688,4086,4215,4223,4851,5170,6091,6722,7057,7101,7424,8434,8817,8829,9037,9314,9353,9723,9734,10000,23670,28514,54567,55294,57381,57556,57608,79733,90627,161742,168667 | ABL1,ANGPT1,RHOA,PTK2B,VEGFD,ITGB1,SMAD1,MAP3K3,MEOX2,NOTCH1,PDPK1,ROBO1,SRF,THBS1,NR2E1,VEGFC,RECK,FGF18,NRP1,SEMA5A,KLF4,SLIT2,SEMA3E,HDAC9,AKT3,CEMIP2,DLL1,DLL4,FBXW7,RHOJ,SEMA6A,JCAD,E2F8,STARD13,SPRED1,BMPER |
| 19_Member | GO Biological Processes | GO:0043536 | positive regulation of blood vessel endothelial cell migration | -3.281039194 | -2.182 | 19/80 | 25,284,2113,3146,4215,5155,5170,5578,7057,7114,7424,8817,8829,9734,10000,23411,57381,57608,154810 | ABL1,ANGPT1,ETS1,HMGB1,MAP3K3,PDGFB,PDPK1,PRKCA,THBS1,TMSB4X,VEGFC,FGF18,NRP1,HDAC9,AKT3,SIRT1,RHOJ,JCAD,AMOTL1 |
| 19_Member | GO Biological Processes | GO:0043535 | regulation of blood vessel endothelial cell migration | -3.039955618 | -1.982 | 30/157 | 25,284,387,2113,3146,4208,4215,4223,4763,4851,5155,5170,5578,7057,7101,7114,7424,8817,8829,9314,9734,10000,23411,54567,55294,57381,57608,90627,154810,161742 | ABL1,ANGPT1,RHOA,ETS1,HMGB1,MEF2C,MAP3K3,MEOX2,NF1,NOTCH1,PDGFB,PDPK1,PRKCA,THBS1,NR2E1,TMSB4X,VEGFC,FGF18,NRP1,KLF4,HDAC9,AKT3,SIRT1,DLL4,FBXW7,RHOJ,JCAD,STARD13,AMOTL1,SPRED1 |
| 20_Summary | GO Biological Processes | GO:0030029 | actin filament-based process | -23.80853507 | -21.223 | 176/760 | 25,27,60,118,120,287,357,375,387,390,395,604,775,776,781,783,817,829,830,998,1073,1398,1482,1490,1730,1739,1756,1901,1946,2017,2035,2044,2059,2185,2195,2252,2255,2475,2549,2626,2764,2824,2885,3688,3717,3759,3781,3815,3845,3984,4082,4205,4208,4430,4627,4628,4643,4763,4853,5048,5063,5142,5144,5156,5170,5239,5295,5358,5573,5581,5584,5592,5629,5756,5829,5898,5910,5911,5921,5962,5999,6093,6323,6444,6709,6711,6722,6781,7046,7114,7168,7170,7248,7430,8440,8452,8470,8476,8500,8829,8851,8936,8976,9037,9124,9353,9475,9620,9723,9788,9873,9886,9922,10006,10052,10092,10109,10152,10178,10395,10420,10458,10529,10565,10580,10787,10810,11252,22843,23002,23122,23189,23242,23327,23332,23603,23607,23704,26053,27032,27063,27302,29119,29766,30011,51804,54443,54822,54874,55114,55435,55607,55704,55740,55761,55785,56907,57381,57551,57619,57822,58480,65979,81873,84665,84978,93663,94134,121512,129446,133746,136319,154810,171024,221981,253260,324,351,667,1027,1278,1281,1289,1303,3267,3796,4131,4133,4534,5352,6683,7042,8766,9508,9748,9793,10048,11076,22919,23271,55137,57606,79649,79875,81565,89795,157922,221074,344148,488,636,1213,1499,1540,3192,5563,5594,6491,7514,9126,10274,10735,10982,23077,23299,26054,55521,60312,128866,137886,361,659,1173,1182,1385,1808,1826,1874,2045,2335,4478,4803,4897,5728,5868,6558,7224,8301,9706,9798,9821,10000,10507,23011,23108,23394,25942,50809,57142,57403,57468,57521,57556,80031,655,2048,2332,2823,2895,2965,3064,3725,4286,4848,4915,5305,5578,5789,6928,7291,7879,8027,8408,8678,9231,9378,9448,9908,10146,10497,11124,11141,22871,22873,22883,23064,23613,23767,23768,25940,26045,50807,51100,55512,55832,57472,57600,57633,84189,84631,114798,139065,158038,246175,255967,347731,375790,546,664,1634,1869,2625,3181,3184,3643,3720,3799,4041,4216,4762,5155,5534,5588,5599,6421,6857,7027,7159,7161,7323,7334,7529,7531,7532,7533,7534,8726,8867,9252,9555,9927,10891,10971,22823,23168,23236,23272,23368,23411,25793,54737,55023,55187,55196,55294,55729,55737,56947,57154,79618,80312,140609,490,1812,1816,1889,1956,2982,3156,5797,6546,7432,9474,9732,10242,753,1654,1810,2033,3146,3638,4091,5494,6311,6653,8780,10427,23493,27072,29091,51128,56998 | ABL1,ABL2,ACTB,ADD1,ADD3,ANK2,SHROOM2,ARF1,RHOA,RND3,ARHGAP6,BCL6,CACNA1C,CACNA1D,CACNA2D1,CACNB2,CAMK2D,CAPZA1,CAPZA2,CDC42,CFL2,CRK,NKX2-5,CCN2,DIAPH2,DLG1,DMD,S1PR1,EFNA5,CTTN,EPB41,EPHA5,EPS8,PTK2B,FAT1,FGF7,FGF10,MTOR,GAB1,GATA4,GMFB,GPM6B,GRB2,ITGB1,JAK2,KCNJ2,KCNN2,KIT,KRAS,LIMK1,MARCKS,MEF2A,MEF2C,MYO1B,MYH9,MYH10,MYO1E,NF1,NOTCH2,PAFAH1B1,PAK3,PDE4B,PDE4D,PDGFRA,PDPK1,PGM5,PIK3R1,PLS3,PRKAR1A,PRKCE,PRKCI,PRKG1,PROX1,TWF1,PXN,RALA,RAP1GDS1,RAP2A,RASA1,RDX,RGS4,ROCK1,SCN1A,SGCD,SPTAN1,SPTBN1,SRF,STC1,TGFBR1,TMSB4X,TPM1,TPM3,TSC1,EZR,NCK2,CUL3,SORBS2,CDC42BPA,PPFIA1,NRP1,CDK5R1,WASF1,WASL,SEMA5A,PDLIM1,SLIT2,ROCK2,CELSR1,SEMA3E,MTSS1,FCHSD2,RHOBTB1,IQSEC1,ABI1,GJC1,ARPC5,ARPC2,ABI2,TENM1,DLC1,TESK2,BAIAP2,NEBL,ARFGEF1,SORBS1,NCKAP1,WASF3,PACSIN2,PPM1E,DAAM1,CLASP2,KANK1,COBL,NEDD4L,CLASP1,CORO1C,CD2AP,KCNE4,AUTS2,ATP2C1,ANKRD1,BMP10,CTNNA3,TMOD3,SH3KBP1,SIX4,ANLN,TRPM7,FNBP1L,ARHGAP17,AP1AR,PPP1R9A,CCDC88A,ENAH,TTC17,FGD6,SPIRE1,RHOJ,TAOK1,SHROOM3,GRHL3,RHOU,PHACTR4,ARPC5L,MYPN,FRMD5,ARHGAP18,ARHGAP12,FGD4,XIRP2,JMY,MTPN,AMOTL1,SYNPO2,THSD7A,RICTOR,APC,APP,DST,CDKN1B,COL1A2,COL3A1,COL5A1,COL12A1,AGFG1,KIF2A,MAP1B,MAP2,MTM1,PLOD2,SPAST,TGFB2,RAB11A,ADAMTS3,SLK,CKAP5,RANBP9,TPPP,MAPRE1,CAMSAP2,FIGN,SLAIN2,MAP7D3,THSD4,NDEL1,NAV3,CAMSAP1,SLC39A12,NCKAP5,ATP2A2,BICD1,CLTC,CTNNB1,CYLD,HNRNPU,PRKAA2,MAPK1,STIL,XPO1,SMC3,STAG1,STAG2,MAPRE2,MYCBP2,BICD2,SENP6,TRIM36,AFAP1,CHMP4B,UBXN2B,AQP4,BMPR2,AP2M1,CLCN3,CREB1,DPYSL2,DSCAM,E2F4,EPHA7,FN1,MSN,NGF,NRCAM,PTEN,RAB5A,SLC12A2,TRPC5,PICALM,ULK2,IST1,RB1CC1,AKT3,SEMA4D,RAB21,RAP1GAP2,ADNP,SIN3A,HP1BP3,RTN4,RAB22A,SLC12A5,RPTOR,SEMA6A,SEMA6D,BMP7,EPHB2,FMR1,GPM6A,GRID2,GTF2H1,HTT,JUN,MITF,CNOT2,NTRK2,PIP4K2A,PRKCA,PTPRD,HNF1B,TWIST1,RAB7A,STAM,ULK1,BECN1,DLG5,NRXN1,MAP4K4,G3BP2,G3BP1,UNC13B,FAF1,IL1RAPL1,NLGN1,DZIP1,CLSTN1,SETX,ZMYND8,FLRT3,FLRT2,FAM98A,LRRTM2,ASAP1,SH3GLB1,SMPD3,CAND1,CNOT6,FNIP2,LRRN1,SLITRK6,SLITRK2,SLITRK1,SLITRK4,LINGO2,CNOT6L,PAN3,LRRTM3,AGRN,ATRX,BNIP3,DCN,E2F1,GATA3,HNRNPA2B1,HNRNPD,INSR,JARID2,KIF5B,LRP5,MAP3K4,NEUROG1,PDGFB,PPP3R1,PRKCQ,MAPK8,SFPQ,SYT1,TFDP1,TP53BP2,TP73,UBE2D3,UBE2N,YWHAB,YWHAE,YWHAG,YWHAH,YWHAZ,EED,SYNJ1,RPS6KA5,MACROH2A1,MFN2,PPARGC1A,YWHAQ,MTF2,RTF1,PLCB1,TASOR,PPP1R13B,SIRT1,FBXO7,MPHOSPH8,PHIP,VPS13D,RESF1,FBXW7,ATF7IP,VPS35,MFF,SMURF1,HMBOX1,TET1,NEK7,ATP2B1,DRD1,DRD5,ECE1,EGFR,GUCY1A1,HMGCR,PTPRM,SLC8A1,VIP,ATG5,DOCK4,KCNMB2,LDLRAD4,DDX3X,DR1,EP300,HMGB1,INSIG1,SMAD6,PPM1A,ATXN2,SORL1,RIOK3,SEC24B,HEY2,VPS41,STXBP6,SAR1B,CTNNBIP1 |
| 20_Member | GO Biological Processes | GO:0030029 | actin filament-based process | -23.80853507 | -21.223 | 176/760 | 25,27,60,118,120,287,357,375,387,390,395,604,775,776,781,783,817,829,830,998,1073,1398,1482,1490,1730,1739,1756,1901,1946,2017,2035,2044,2059,2185,2195,2252,2255,2475,2549,2626,2764,2824,2885,3688,3717,3759,3781,3815,3845,3984,4082,4205,4208,4430,4627,4628,4643,4763,4853,5048,5063,5142,5144,5156,5170,5239,5295,5358,5573,5581,5584,5592,5629,5756,5829,5898,5910,5911,5921,5962,5999,6093,6323,6444,6709,6711,6722,6781,7046,7114,7168,7170,7248,7430,8440,8452,8470,8476,8500,8829,8851,8936,8976,9037,9124,9353,9475,9620,9723,9788,9873,9886,9922,10006,10052,10092,10109,10152,10178,10395,10420,10458,10529,10565,10580,10787,10810,11252,22843,23002,23122,23189,23242,23327,23332,23603,23607,23704,26053,27032,27063,27302,29119,29766,30011,51804,54443,54822,54874,55114,55435,55607,55704,55740,55761,55785,56907,57381,57551,57619,57822,58480,65979,81873,84665,84978,93663,94134,121512,129446,133746,136319,154810,171024,221981,253260 | ABL1,ABL2,ACTB,ADD1,ADD3,ANK2,SHROOM2,ARF1,RHOA,RND3,ARHGAP6,BCL6,CACNA1C,CACNA1D,CACNA2D1,CACNB2,CAMK2D,CAPZA1,CAPZA2,CDC42,CFL2,CRK,NKX2-5,CCN2,DIAPH2,DLG1,DMD,S1PR1,EFNA5,CTTN,EPB41,EPHA5,EPS8,PTK2B,FAT1,FGF7,FGF10,MTOR,GAB1,GATA4,GMFB,GPM6B,GRB2,ITGB1,JAK2,KCNJ2,KCNN2,KIT,KRAS,LIMK1,MARCKS,MEF2A,MEF2C,MYO1B,MYH9,MYH10,MYO1E,NF1,NOTCH2,PAFAH1B1,PAK3,PDE4B,PDE4D,PDGFRA,PDPK1,PGM5,PIK3R1,PLS3,PRKAR1A,PRKCE,PRKCI,PRKG1,PROX1,TWF1,PXN,RALA,RAP1GDS1,RAP2A,RASA1,RDX,RGS4,ROCK1,SCN1A,SGCD,SPTAN1,SPTBN1,SRF,STC1,TGFBR1,TMSB4X,TPM1,TPM3,TSC1,EZR,NCK2,CUL3,SORBS2,CDC42BPA,PPFIA1,NRP1,CDK5R1,WASF1,WASL,SEMA5A,PDLIM1,SLIT2,ROCK2,CELSR1,SEMA3E,MTSS1,FCHSD2,RHOBTB1,IQSEC1,ABI1,GJC1,ARPC5,ARPC2,ABI2,TENM1,DLC1,TESK2,BAIAP2,NEBL,ARFGEF1,SORBS1,NCKAP1,WASF3,PACSIN2,PPM1E,DAAM1,CLASP2,KANK1,COBL,NEDD4L,CLASP1,CORO1C,CD2AP,KCNE4,AUTS2,ATP2C1,ANKRD1,BMP10,CTNNA3,TMOD3,SH3KBP1,SIX4,ANLN,TRPM7,FNBP1L,ARHGAP17,AP1AR,PPP1R9A,CCDC88A,ENAH,TTC17,FGD6,SPIRE1,RHOJ,TAOK1,SHROOM3,GRHL3,RHOU,PHACTR4,ARPC5L,MYPN,FRMD5,ARHGAP18,ARHGAP12,FGD4,XIRP2,JMY,MTPN,AMOTL1,SYNPO2,THSD7A,RICTOR |
| 20_Member | GO Biological Processes | GO:0030036 | actin cytoskeleton organization | -21.14851731 | -18.712 | 155/667 | 25,27,60,118,120,357,375,387,390,395,604,829,830,998,1073,1398,1482,1490,1730,1739,1901,1946,2017,2035,2044,2059,2185,2195,2252,2255,2475,2549,2764,2824,2885,3688,3717,3815,3845,3984,4082,4205,4208,4430,4627,4628,4763,4853,5048,5063,5156,5170,5239,5295,5358,5573,5581,5584,5592,5629,5756,5829,5898,5910,5911,5921,5962,5999,6093,6709,6711,6722,7046,7114,7168,7170,7248,7430,8440,8452,8470,8476,8500,8829,8851,8936,8976,9037,9124,9353,9475,9620,9723,9788,9873,9886,9922,10006,10092,10109,10152,10178,10395,10420,10458,10529,10565,10580,10787,10810,11252,22843,23002,23122,23189,23242,23332,23603,23607,26053,27032,27063,27302,29766,30011,51804,54443,54822,55114,55435,55607,55704,55740,55761,55785,56907,57381,57551,57619,57822,58480,65979,81873,84665,84978,93663,94134,121512,129446,133746,136319,154810,171024,221981,253260 | ABL1,ABL2,ACTB,ADD1,ADD3,SHROOM2,ARF1,RHOA,RND3,ARHGAP6,BCL6,CAPZA1,CAPZA2,CDC42,CFL2,CRK,NKX2-5,CCN2,DIAPH2,DLG1,S1PR1,EFNA5,CTTN,EPB41,EPHA5,EPS8,PTK2B,FAT1,FGF7,FGF10,MTOR,GAB1,GMFB,GPM6B,GRB2,ITGB1,JAK2,KIT,KRAS,LIMK1,MARCKS,MEF2A,MEF2C,MYO1B,MYH9,MYH10,NF1,NOTCH2,PAFAH1B1,PAK3,PDGFRA,PDPK1,PGM5,PIK3R1,PLS3,PRKAR1A,PRKCE,PRKCI,PRKG1,PROX1,TWF1,PXN,RALA,RAP1GDS1,RAP2A,RASA1,RDX,RGS4,ROCK1,SPTAN1,SPTBN1,SRF,TGFBR1,TMSB4X,TPM1,TPM3,TSC1,EZR,NCK2,CUL3,SORBS2,CDC42BPA,PPFIA1,NRP1,CDK5R1,WASF1,WASL,SEMA5A,PDLIM1,SLIT2,ROCK2,CELSR1,SEMA3E,MTSS1,FCHSD2,RHOBTB1,IQSEC1,ABI1,ARPC5,ARPC2,ABI2,TENM1,DLC1,TESK2,BAIAP2,NEBL,ARFGEF1,SORBS1,NCKAP1,WASF3,PACSIN2,PPM1E,DAAM1,CLASP2,KANK1,COBL,CLASP1,CORO1C,CD2AP,AUTS2,ATP2C1,ANKRD1,BMP10,TMOD3,SH3KBP1,SIX4,ANLN,TRPM7,ARHGAP17,AP1AR,PPP1R9A,CCDC88A,ENAH,TTC17,FGD6,SPIRE1,RHOJ,TAOK1,SHROOM3,GRHL3,RHOU,PHACTR4,ARPC5L,MYPN,FRMD5,ARHGAP18,ARHGAP12,FGD4,XIRP2,JMY,MTPN,AMOTL1,SYNPO2,THSD7A,RICTOR |
| 20_Member | GO Biological Processes | GO:0097435 | supramolecular fiber organization | -17.40131996 | -15.119 | 147/674 | 25,118,120,324,351,357,375,387,390,395,667,829,830,998,1027,1073,1278,1281,1289,1303,1482,1490,1730,1739,1901,2017,2059,2185,2195,2475,2764,2885,3267,3688,3717,3796,3984,4082,4131,4133,4205,4208,4430,4534,4628,4763,5048,5063,5156,5239,5295,5352,5358,5573,5581,5584,5629,5756,5829,5921,5962,5999,6093,6683,6709,6711,6722,7042,7046,7114,7168,7170,7248,7430,8440,8452,8470,8500,8766,8829,8851,8936,8976,9037,9353,9475,9508,9748,9793,9873,9886,10006,10048,10092,10109,10152,10178,10395,10458,10529,10565,10580,10787,10810,11076,22843,22919,23122,23189,23242,23271,23332,23603,23607,27063,27302,29766,30011,51804,55114,55137,55435,55607,55704,55740,55761,56907,57381,57551,57606,57619,58480,79649,79875,81565,81873,84665,89795,93663,94134,133746,136319,157922,171024,221074,253260,344148 | ABL1,ADD1,ADD3,APC,APP,SHROOM2,ARF1,RHOA,RND3,ARHGAP6,DST,CAPZA1,CAPZA2,CDC42,CDKN1B,CFL2,COL1A2,COL3A1,COL5A1,COL12A1,NKX2-5,CCN2,DIAPH2,DLG1,S1PR1,CTTN,EPS8,PTK2B,FAT1,MTOR,GMFB,GRB2,AGFG1,ITGB1,JAK2,KIF2A,LIMK1,MARCKS,MAP1B,MAP2,MEF2A,MEF2C,MYO1B,MTM1,MYH10,NF1,PAFAH1B1,PAK3,PDGFRA,PGM5,PIK3R1,PLOD2,PLS3,PRKAR1A,PRKCE,PRKCI,PROX1,TWF1,PXN,RASA1,RDX,RGS4,ROCK1,SPAST,SPTAN1,SPTBN1,SRF,TGFB2,TGFBR1,TMSB4X,TPM1,TPM3,TSC1,EZR,NCK2,CUL3,SORBS2,PPFIA1,RAB11A,NRP1,CDK5R1,WASF1,WASL,SEMA5A,SLIT2,ROCK2,ADAMTS3,SLK,CKAP5,FCHSD2,RHOBTB1,ABI1,RANBP9,ARPC5,ARPC2,ABI2,TENM1,DLC1,BAIAP2,NEBL,ARFGEF1,SORBS1,NCKAP1,WASF3,TPPP,PPM1E,MAPRE1,CLASP2,KANK1,COBL,CAMSAP2,CLASP1,CORO1C,CD2AP,ANKRD1,BMP10,TMOD3,SH3KBP1,SIX4,ARHGAP17,FIGN,AP1AR,PPP1R9A,CCDC88A,ENAH,TTC17,SPIRE1,RHOJ,TAOK1,SLAIN2,SHROOM3,RHOU,MAP7D3,THSD4,NDEL1,ARPC5L,MYPN,NAV3,ARHGAP18,ARHGAP12,JMY,MTPN,CAMSAP1,SYNPO2,SLC39A12,RICTOR,NCKAP5 |
| 20_Member | GO Biological Processes | GO:0032970 | regulation of actin filament-based process | -16.92195345 | -14.665 | 100/389 | 25,27,118,120,287,375,387,390,395,488,775,817,829,830,998,1073,1398,1490,1739,1901,1946,2017,2044,2059,2185,2475,2626,2764,2824,2885,3759,3984,4208,4627,4853,5063,5142,5144,5156,5295,5581,5629,5756,5829,5921,5962,5999,6093,6709,6711,6781,7046,7114,7168,7248,7430,8440,8500,8829,8851,8936,8976,9037,9353,9475,9620,9723,9873,9886,10092,10109,10152,10178,10395,10458,10565,10787,22843,23122,23189,23332,23607,27302,29119,29766,55114,55435,55607,55704,56907,57381,57551,57822,58480,81873,93663,133746,136319,171024,253260 | ABL1,ABL2,ADD1,ADD3,ANK2,ARF1,RHOA,RND3,ARHGAP6,ATP2A2,CACNA1C,CAMK2D,CAPZA1,CAPZA2,CDC42,CFL2,CRK,CCN2,DLG1,S1PR1,EFNA5,CTTN,EPHA5,EPS8,PTK2B,MTOR,GATA4,GMFB,GPM6B,GRB2,KCNJ2,LIMK1,MEF2C,MYH9,NOTCH2,PAK3,PDE4B,PDE4D,PDGFRA,PIK3R1,PRKCE,PROX1,TWF1,PXN,RASA1,RDX,RGS4,ROCK1,SPTAN1,SPTBN1,STC1,TGFBR1,TMSB4X,TPM1,TSC1,EZR,NCK2,PPFIA1,NRP1,CDK5R1,WASF1,WASL,SEMA5A,SLIT2,ROCK2,CELSR1,SEMA3E,FCHSD2,RHOBTB1,ARPC5,ARPC2,ABI2,TENM1,DLC1,BAIAP2,ARFGEF1,NCKAP1,PPM1E,CLASP2,KANK1,CLASP1,CD2AP,BMP10,CTNNA3,TMOD3,ARHGAP17,AP1AR,PPP1R9A,CCDC88A,SPIRE1,RHOJ,TAOK1,GRHL3,RHOU,ARPC5L,ARHGAP18,JMY,MTPN,SYNPO2,RICTOR |
| 20_Member | GO Biological Processes | GO:0051493 | regulation of cytoskeleton organization | -15.71160485 | -13.534 | 121/534 | 25,27,118,120,324,375,387,390,395,636,829,830,998,1027,1073,1213,1398,1490,1499,1540,1739,1901,1946,2017,2044,2059,2185,2475,2764,2824,2885,3192,3984,4131,4133,4208,4853,5048,5063,5156,5295,5563,5581,5594,5629,5756,5829,5921,5962,5999,6093,6491,6683,6709,6711,7046,7114,7168,7248,7430,7514,8440,8500,8829,8851,8936,8976,9037,9126,9353,9475,9620,9723,9873,9886,10092,10109,10152,10178,10274,10395,10458,10565,10735,10787,10982,22843,22919,23077,23122,23189,23271,23299,23332,23607,26054,27302,29766,55114,55435,55521,55607,55704,56907,57381,57551,57606,57822,58480,60312,81873,89795,93663,128866,133746,136319,137886,157922,171024,221074,253260 | ABL1,ABL2,ADD1,ADD3,APC,ARF1,RHOA,RND3,ARHGAP6,BICD1,CAPZA1,CAPZA2,CDC42,CDKN1B,CFL2,CLTC,CRK,CCN2,CTNNB1,CYLD,DLG1,S1PR1,EFNA5,CTTN,EPHA5,EPS8,PTK2B,MTOR,GMFB,GPM6B,GRB2,HNRNPU,LIMK1,MAP1B,MAP2,MEF2C,NOTCH2,PAFAH1B1,PAK3,PDGFRA,PIK3R1,PRKAA2,PRKCE,MAPK1,PROX1,TWF1,PXN,RASA1,RDX,RGS4,ROCK1,STIL,SPAST,SPTAN1,SPTBN1,TGFBR1,TMSB4X,TPM1,TSC1,EZR,XPO1,NCK2,PPFIA1,NRP1,CDK5R1,WASF1,WASL,SEMA5A,SMC3,SLIT2,ROCK2,CELSR1,SEMA3E,FCHSD2,RHOBTB1,ARPC5,ARPC2,ABI2,TENM1,STAG1,DLC1,BAIAP2,ARFGEF1,STAG2,NCKAP1,MAPRE2,PPM1E,MAPRE1,MYCBP2,CLASP2,KANK1,CAMSAP2,BICD2,CLASP1,CD2AP,SENP6,BMP10,TMOD3,ARHGAP17,AP1AR,TRIM36,PPP1R9A,CCDC88A,SPIRE1,RHOJ,TAOK1,SLAIN2,GRHL3,RHOU,AFAP1,ARPC5L,NAV3,ARHGAP18,CHMP4B,JMY,MTPN,UBXN2B,CAMSAP1,SYNPO2,SLC39A12,RICTOR |
| 20_Member | GO Biological Processes | GO:0032535 | regulation of cellular component size | -15.58942856 | -13.415 | 94/370 | 25,118,120,361,375,387,659,829,830,1073,1173,1182,1385,1739,1808,1826,1874,1946,2017,2045,2059,2185,2335,2475,2764,2885,3984,4131,4133,4478,4803,4897,5048,5063,5581,5728,5756,5868,5921,5962,6558,6709,6711,6722,7114,7224,7248,7430,8301,8440,8766,8829,8851,8936,8976,9037,9353,9706,9723,9798,9821,9873,9886,10000,10092,10109,10152,10178,10458,10507,10565,10787,23011,23108,23189,23394,25942,29766,50809,55435,55607,56907,57142,57403,57468,57521,57556,80031,81565,81873,93663,133746,136319,253260 | ABL1,ADD1,ADD3,AQP4,ARF1,RHOA,BMPR2,CAPZA1,CAPZA2,CFL2,AP2M1,CLCN3,CREB1,DLG1,DPYSL2,DSCAM,E2F4,EFNA5,CTTN,EPHA7,EPS8,PTK2B,FN1,MTOR,GMFB,GRB2,LIMK1,MAP1B,MAP2,MSN,NGF,NRCAM,PAFAH1B1,PAK3,PRKCE,PTEN,TWF1,RAB5A,RASA1,RDX,SLC12A2,SPTAN1,SPTBN1,SRF,TMSB4X,TRPC5,TSC1,EZR,PICALM,NCK2,RAB11A,NRP1,CDK5R1,WASF1,WASL,SEMA5A,SLIT2,ULK2,SEMA3E,IST1,RB1CC1,FCHSD2,RHOBTB1,AKT3,ARPC5,ARPC2,ABI2,TENM1,BAIAP2,SEMA4D,ARFGEF1,NCKAP1,RAB21,RAP1GAP2,KANK1,ADNP,SIN3A,TMOD3,HP1BP3,AP1AR,PPP1R9A,SPIRE1,RTN4,RAB22A,SLC12A5,RPTOR,SEMA6A,SEMA6D,NDEL1,ARPC5L,ARHGAP18,JMY,MTPN,RICTOR |
| 20_Member | GO Biological Processes | GO:0032956 | regulation of actin cytoskeleton organization | -15.31223989 | -13.152 | 89/344 | 25,27,118,120,375,387,390,395,829,830,998,1073,1398,1490,1739,1901,1946,2017,2044,2059,2185,2475,2764,2824,2885,3984,4208,4853,5063,5156,5295,5581,5629,5756,5829,5921,5962,5999,6093,6709,6711,7046,7114,7168,7248,7430,8440,8500,8829,8851,8936,8976,9037,9353,9475,9620,9723,9873,9886,10092,10109,10152,10178,10395,10458,10565,10787,22843,23122,23189,23332,23607,27302,29766,55114,55435,55607,55704,56907,57381,57551,57822,58480,81873,93663,133746,136319,171024,253260 | ABL1,ABL2,ADD1,ADD3,ARF1,RHOA,RND3,ARHGAP6,CAPZA1,CAPZA2,CDC42,CFL2,CRK,CCN2,DLG1,S1PR1,EFNA5,CTTN,EPHA5,EPS8,PTK2B,MTOR,GMFB,GPM6B,GRB2,LIMK1,MEF2C,NOTCH2,PAK3,PDGFRA,PIK3R1,PRKCE,PROX1,TWF1,PXN,RASA1,RDX,RGS4,ROCK1,SPTAN1,SPTBN1,TGFBR1,TMSB4X,TPM1,TSC1,EZR,NCK2,PPFIA1,NRP1,CDK5R1,WASF1,WASL,SEMA5A,SLIT2,ROCK2,CELSR1,SEMA3E,FCHSD2,RHOBTB1,ARPC5,ARPC2,ABI2,TENM1,DLC1,BAIAP2,ARFGEF1,NCKAP1,PPM1E,CLASP2,KANK1,CLASP1,CD2AP,BMP10,TMOD3,ARHGAP17,AP1AR,PPP1R9A,CCDC88A,SPIRE1,RHOJ,TAOK1,GRHL3,RHOU,ARPC5L,ARHGAP18,JMY,MTPN,SYNPO2,RICTOR |
| 20_Member | GO Biological Processes | GO:0007015 | actin filament organization | -14.96383299 | -12.846 | 98/402 | 25,118,120,357,375,387,390,395,829,830,998,1073,1490,1730,1739,1901,2017,2059,2185,2195,2475,2764,2885,3688,3717,3984,4082,4430,5063,5295,5358,5581,5584,5629,5756,5829,5921,5962,5999,6093,6709,6711,6722,7046,7114,7168,7170,7248,7430,8440,8452,8470,8500,8829,8936,8976,9037,9353,9475,9873,9886,10006,10092,10109,10152,10178,10395,10458,10529,10565,10580,10787,10810,22843,23122,23189,23242,23332,23603,23607,29766,30011,55114,55435,55607,55740,55761,56907,57381,57619,58480,81873,93663,94134,133746,136319,171024,253260 | ABL1,ADD1,ADD3,SHROOM2,ARF1,RHOA,RND3,ARHGAP6,CAPZA1,CAPZA2,CDC42,CFL2,CCN2,DIAPH2,DLG1,S1PR1,CTTN,EPS8,PTK2B,FAT1,MTOR,GMFB,GRB2,ITGB1,JAK2,LIMK1,MARCKS,MYO1B,PAK3,PIK3R1,PLS3,PRKCE,PRKCI,PROX1,TWF1,PXN,RASA1,RDX,RGS4,ROCK1,SPTAN1,SPTBN1,SRF,TGFBR1,TMSB4X,TPM1,TPM3,TSC1,EZR,NCK2,CUL3,SORBS2,PPFIA1,NRP1,WASF1,WASL,SEMA5A,SLIT2,ROCK2,FCHSD2,RHOBTB1,ABI1,ARPC5,ARPC2,ABI2,TENM1,DLC1,BAIAP2,NEBL,ARFGEF1,SORBS1,NCKAP1,WASF3,PPM1E,CLASP2,KANK1,COBL,CLASP1,CORO1C,CD2AP,TMOD3,SH3KBP1,ARHGAP17,AP1AR,PPP1R9A,ENAH,TTC17,SPIRE1,RHOJ,SHROOM3,RHOU,ARPC5L,ARHGAP18,ARHGAP12,JMY,MTPN,SYNPO2,RICTOR |
| 20_Member | GO Biological Processes | GO:0044089 | positive regulation of cellular component biogenesis | -14.85827867 | -12.753 | 120/541 | 25,324,375,387,655,998,1027,1385,1482,1490,1499,1739,1946,2017,2048,2059,2185,2332,2475,2764,2823,2885,2895,2965,3064,3725,3815,3984,4131,4286,4478,4848,4915,5305,5578,5581,5629,5789,5829,5898,6093,6722,6928,7046,7168,7248,7291,7879,8027,8408,8440,8678,8829,8851,8936,8976,9231,9378,9448,9475,9873,9886,9908,10092,10109,10146,10152,10178,10458,10497,10507,10787,11076,11124,11141,22843,22871,22873,22883,22919,23064,23122,23242,23332,23394,23613,23767,23768,25940,26045,26053,27302,50807,51100,54443,54874,55435,55512,55704,55832,56907,57472,57600,57606,57633,81565,81873,84189,84631,89795,114798,133746,139065,158038,171024,246175,253260,255967,347731,375790 | ABL1,APC,ARF1,RHOA,BMP7,CDC42,CDKN1B,CREB1,NKX2-5,CCN2,CTNNB1,DLG1,EFNA5,CTTN,EPHB2,EPS8,PTK2B,FMR1,MTOR,GMFB,GPM6A,GRB2,GRID2,GTF2H1,HTT,JUN,KIT,LIMK1,MAP1B,MITF,MSN,CNOT2,NTRK2,PIP4K2A,PRKCA,PRKCE,PROX1,PTPRD,PXN,RALA,ROCK1,SRF,HNF1B,TGFBR1,TPM1,TSC1,TWIST1,RAB7A,STAM,ULK1,NCK2,BECN1,NRP1,CDK5R1,WASF1,WASL,DLG5,NRXN1,MAP4K4,ROCK2,FCHSD2,RHOBTB1,G3BP2,ARPC5,ARPC2,G3BP1,ABI2,TENM1,BAIAP2,UNC13B,SEMA4D,NCKAP1,TPPP,FAF1,IL1RAPL1,PPM1E,NLGN1,DZIP1,CLSTN1,MAPRE1,SETX,CLASP2,COBL,CLASP1,ADNP,ZMYND8,FLRT3,FLRT2,FAM98A,LRRTM2,AUTS2,BMP10,ASAP1,SH3GLB1,ANLN,FNBP1L,AP1AR,SMPD3,CCDC88A,CAND1,SPIRE1,CNOT6,FNIP2,SLAIN2,LRRN1,NDEL1,ARPC5L,SLITRK6,SLITRK2,NAV3,SLITRK1,JMY,SLITRK4,LINGO2,SYNPO2,CNOT6L,RICTOR,PAN3,LRRTM3,AGRN |
| 20_Member | GO Biological Processes | GO:0010638 | positive regulation of organelle organization | -13.82851533 | -11.785 | 130/624 | 25,375,387,546,604,664,998,1027,1073,1490,1499,1634,1739,1869,2017,2185,2332,2475,2625,2764,2885,3064,3181,3184,3192,3643,3720,3799,3984,4041,4131,4216,4478,4762,4848,5155,5305,5534,5581,5588,5594,5599,5629,5829,6421,6683,6857,7027,7046,7159,7161,7168,7248,7323,7334,7529,7531,7532,7533,7534,8408,8440,8452,8678,8726,8829,8851,8867,8936,8976,9037,9252,9475,9555,9873,9886,9908,9927,10092,10109,10146,10152,10178,10458,10787,10891,10971,22823,22843,22873,22919,23168,23236,23272,23332,23368,23411,25793,25942,26053,27302,50807,51100,54737,55023,55187,55196,55294,55435,55512,55704,55729,55737,56907,56947,57154,57472,57606,58480,79618,80312,81873,89795,133746,137886,140609,171024,246175,253260,255967 | ABL1,ARF1,RHOA,ATRX,BCL6,BNIP3,CDC42,CDKN1B,CFL2,CCN2,CTNNB1,DCN,DLG1,E2F1,CTTN,PTK2B,FMR1,MTOR,GATA3,GMFB,GRB2,HTT,HNRNPA2B1,HNRNPD,HNRNPU,INSR,JARID2,KIF5B,LIMK1,LRP5,MAP1B,MAP3K4,MSN,NEUROG1,CNOT2,PDGFB,PIP4K2A,PPP3R1,PRKCE,PRKCQ,MAPK1,MAPK8,PROX1,PXN,SFPQ,SPAST,SYT1,TFDP1,TGFBR1,TP53BP2,TP73,TPM1,TSC1,UBE2D3,UBE2N,YWHAB,YWHAE,YWHAG,YWHAH,YWHAZ,ULK1,NCK2,CUL3,BECN1,EED,NRP1,CDK5R1,SYNJ1,WASF1,WASL,SEMA5A,RPS6KA5,ROCK2,MACROH2A1,FCHSD2,RHOBTB1,G3BP2,MFN2,ARPC5,ARPC2,G3BP1,ABI2,TENM1,BAIAP2,NCKAP1,PPARGC1A,YWHAQ,MTF2,PPM1E,DZIP1,MAPRE1,RTF1,PLCB1,TASOR,CLASP1,PPP1R13B,SIRT1,FBXO7,SIN3A,AUTS2,BMP10,ASAP1,SH3GLB1,MPHOSPH8,PHIP,VPS13D,RESF1,FBXW7,AP1AR,SMPD3,CCDC88A,ATF7IP,VPS35,SPIRE1,MFF,SMURF1,CNOT6,SLAIN2,RHOU,HMBOX1,TET1,ARPC5L,NAV3,JMY,UBXN2B,NEK7,SYNPO2,CNOT6L,RICTOR,PAN3 |
| 20_Member | GO Biological Processes | GO:0090066 | regulation of anatomical structure size | -13.77684705 | -11.736 | 112/507 | 25,118,120,361,375,387,490,659,829,830,1073,1173,1182,1385,1739,1808,1812,1816,1826,1874,1889,1946,1956,2017,2045,2059,2185,2335,2475,2764,2885,2982,3156,3984,4131,4133,4478,4803,4897,5048,5063,5581,5592,5728,5756,5797,5868,5910,5921,5962,6093,6546,6558,6709,6711,6722,6928,7114,7224,7248,7430,7432,8301,8440,8766,8829,8851,8936,8976,9037,9353,9474,9475,9706,9723,9732,9798,9821,9873,9886,10000,10092,10109,10152,10178,10242,10458,10507,10565,10787,23011,23108,23189,23394,25942,29766,50809,55435,55607,56907,57142,57403,57468,57521,57556,80031,81565,81873,93663,133746,136319,253260 | ABL1,ADD1,ADD3,AQP4,ARF1,RHOA,ATP2B1,BMPR2,CAPZA1,CAPZA2,CFL2,AP2M1,CLCN3,CREB1,DLG1,DPYSL2,DRD1,DRD5,DSCAM,E2F4,ECE1,EFNA5,EGFR,CTTN,EPHA7,EPS8,PTK2B,FN1,MTOR,GMFB,GRB2,GUCY1A1,HMGCR,LIMK1,MAP1B,MAP2,MSN,NGF,NRCAM,PAFAH1B1,PAK3,PRKCE,PRKG1,PTEN,TWF1,PTPRM,RAB5A,RAP1GDS1,RASA1,RDX,ROCK1,SLC8A1,SLC12A2,SPTAN1,SPTBN1,SRF,HNF1B,TMSB4X,TRPC5,TSC1,EZR,VIP,PICALM,NCK2,RAB11A,NRP1,CDK5R1,WASF1,WASL,SEMA5A,SLIT2,ATG5,ROCK2,ULK2,SEMA3E,DOCK4,IST1,RB1CC1,FCHSD2,RHOBTB1,AKT3,ARPC5,ARPC2,ABI2,TENM1,KCNMB2,BAIAP2,SEMA4D,ARFGEF1,NCKAP1,RAB21,RAP1GAP2,KANK1,ADNP,SIN3A,TMOD3,HP1BP3,AP1AR,PPP1R9A,SPIRE1,RTN4,RAB22A,SLC12A5,RPTOR,SEMA6A,SEMA6D,NDEL1,ARPC5L,ARHGAP18,JMY,MTPN,RICTOR |
| 20_Member | GO Biological Processes | GO:0043254 | regulation of protein complex assembly | -12.54969534 | -10.578 | 103/469 | 25,118,120,375,387,753,829,830,998,1027,1385,1482,1499,1654,1739,1810,2017,2033,2059,2185,2475,2764,2885,2965,3146,3638,3725,4091,4131,4133,4286,4478,4848,5063,5494,5563,5581,5756,5921,5962,6311,6653,6709,6711,6722,6928,7114,7291,8408,8440,8780,8851,8936,8976,9353,9873,9886,9908,10092,10109,10146,10152,10178,10427,10458,10497,10565,10787,11076,11124,22919,23064,23189,23271,23332,23493,26054,27072,29091,29766,50807,51100,51128,55435,55607,55729,55737,55832,56907,56998,57472,57600,57606,81873,89795,93663,133746,136319,157922,221074,246175,253260,255967 | ABL1,ADD1,ADD3,ARF1,RHOA,LDLRAD4,CAPZA1,CAPZA2,CDC42,CDKN1B,CREB1,NKX2-5,CTNNB1,DDX3X,DLG1,DR1,CTTN,EP300,EPS8,PTK2B,MTOR,GMFB,GRB2,GTF2H1,HMGB1,INSIG1,JUN,SMAD6,MAP1B,MAP2,MITF,MSN,CNOT2,PAK3,PPM1A,PRKAA2,PRKCE,TWF1,RASA1,RDX,ATXN2,SORL1,SPTAN1,SPTBN1,SRF,HNF1B,TMSB4X,TWIST1,ULK1,NCK2,RIOK3,CDK5R1,WASF1,WASL,SLIT2,FCHSD2,RHOBTB1,G3BP2,ARPC5,ARPC2,G3BP1,ABI2,TENM1,SEC24B,BAIAP2,UNC13B,ARFGEF1,NCKAP1,TPPP,FAF1,MAPRE1,SETX,KANK1,CAMSAP2,CLASP1,HEY2,SENP6,VPS41,STXBP6,TMOD3,ASAP1,SH3GLB1,SAR1B,AP1AR,PPP1R9A,ATF7IP,VPS35,CAND1,SPIRE1,CTNNBIP1,CNOT6,FNIP2,SLAIN2,ARPC5L,NAV3,ARHGAP18,JMY,MTPN,CAMSAP1,SLC39A12,CNOT6L,RICTOR,PAN3 |
| 20_Member | GO Biological Processes | GO:1902903 | regulation of supramolecular fiber organization | -12.37482422 | -10.420 | 84/352 | 25,118,120,324,351,375,387,395,829,830,998,1027,1073,1490,1739,1901,2017,2059,2185,2475,2764,2885,3984,4131,4133,4208,5063,5295,5581,5629,5756,5829,5921,5962,5999,6093,6683,6709,6711,7046,7114,7168,7248,8440,8500,8829,8851,8936,8976,9037,9353,9475,9873,9886,10092,10109,10152,10178,10395,10458,10565,10787,22843,22919,23122,23189,23271,23332,27302,29766,55435,55607,56907,57551,57606,81873,89795,93663,133746,136319,157922,171024,221074,253260 | ABL1,ADD1,ADD3,APC,APP,ARF1,RHOA,ARHGAP6,CAPZA1,CAPZA2,CDC42,CDKN1B,CFL2,CCN2,DLG1,S1PR1,CTTN,EPS8,PTK2B,MTOR,GMFB,GRB2,LIMK1,MAP1B,MAP2,MEF2C,PAK3,PIK3R1,PRKCE,PROX1,TWF1,PXN,RASA1,RDX,RGS4,ROCK1,SPAST,SPTAN1,SPTBN1,TGFBR1,TMSB4X,TPM1,TSC1,NCK2,PPFIA1,NRP1,CDK5R1,WASF1,WASL,SEMA5A,SLIT2,ROCK2,FCHSD2,RHOBTB1,ARPC5,ARPC2,ABI2,TENM1,DLC1,BAIAP2,ARFGEF1,NCKAP1,PPM1E,MAPRE1,CLASP2,KANK1,CAMSAP2,CLASP1,BMP10,TMOD3,AP1AR,PPP1R9A,SPIRE1,TAOK1,SLAIN2,ARPC5L,NAV3,ARHGAP18,JMY,MTPN,CAMSAP1,SYNPO2,SLC39A12,RICTOR |
| 20_Member | GO Biological Processes | GO:0110053 | regulation of actin filament organization | -11.52794446 | -9.631 | 67/261 | 25,118,120,375,387,395,829,830,998,1073,1490,1739,1901,2017,2059,2185,2475,2764,2885,3984,5063,5295,5581,5756,5829,5921,5962,5999,6093,6709,6711,7046,7114,7168,7248,8440,8500,8829,8936,8976,9037,9353,9475,9873,9886,10092,10109,10152,10178,10395,10458,10565,10787,22843,23122,23189,23332,29766,55435,55607,56907,81873,93663,133746,136319,171024,253260 | ABL1,ADD1,ADD3,ARF1,RHOA,ARHGAP6,CAPZA1,CAPZA2,CDC42,CFL2,CCN2,DLG1,S1PR1,CTTN,EPS8,PTK2B,MTOR,GMFB,GRB2,LIMK1,PAK3,PIK3R1,PRKCE,TWF1,PXN,RASA1,RDX,RGS4,ROCK1,SPTAN1,SPTBN1,TGFBR1,TMSB4X,TPM1,TSC1,NCK2,PPFIA1,NRP1,WASF1,WASL,SEMA5A,SLIT2,ROCK2,FCHSD2,RHOBTB1,ARPC5,ARPC2,ABI2,TENM1,DLC1,BAIAP2,ARFGEF1,NCKAP1,PPM1E,CLASP2,KANK1,CLASP1,TMOD3,AP1AR,PPP1R9A,SPIRE1,ARPC5L,ARHGAP18,JMY,MTPN,SYNPO2,RICTOR |
| 20_Member | GO Biological Processes | GO:0032271 | regulation of protein polymerization | -10.25158547 | -8.434 | 57/218 | 25,118,120,375,387,829,830,1027,1739,2017,2059,2185,2475,2764,2885,4131,4133,5063,5581,5756,5921,5962,6709,6711,7114,8440,8851,8936,8976,9353,9873,9886,10092,10109,10152,10178,10458,10565,10787,11076,22919,23189,23271,23332,29766,55435,55607,56907,57606,81873,89795,93663,133746,136319,157922,221074,253260 | ABL1,ADD1,ADD3,ARF1,RHOA,CAPZA1,CAPZA2,CDKN1B,DLG1,CTTN,EPS8,PTK2B,MTOR,GMFB,GRB2,MAP1B,MAP2,PAK3,PRKCE,TWF1,RASA1,RDX,SPTAN1,SPTBN1,TMSB4X,NCK2,CDK5R1,WASF1,WASL,SLIT2,FCHSD2,RHOBTB1,ARPC5,ARPC2,ABI2,TENM1,BAIAP2,ARFGEF1,NCKAP1,TPPP,MAPRE1,KANK1,CAMSAP2,CLASP1,TMOD3,AP1AR,PPP1R9A,SPIRE1,SLAIN2,ARPC5L,NAV3,ARHGAP18,JMY,MTPN,CAMSAP1,SLC39A12,RICTOR |
| 20_Member | GO Biological Processes | GO:0051258 | protein polymerization | -9.780984233 | -8.000 | 67/284 | 25,118,120,375,387,395,829,830,1027,1739,2017,2059,2185,2475,2764,2885,3717,4131,4133,5063,5581,5756,5921,5962,6709,6711,7114,8440,8851,8936,8976,9353,9793,9873,9886,10048,10092,10109,10152,10178,10458,10565,10787,10810,11076,22919,23122,23189,23242,23271,23332,29766,55435,55607,55761,56907,57606,79649,81565,81873,89795,93663,133746,136319,157922,221074,253260 | ABL1,ADD1,ADD3,ARF1,RHOA,ARHGAP6,CAPZA1,CAPZA2,CDKN1B,DLG1,CTTN,EPS8,PTK2B,MTOR,GMFB,GRB2,JAK2,MAP1B,MAP2,PAK3,PRKCE,TWF1,RASA1,RDX,SPTAN1,SPTBN1,TMSB4X,NCK2,CDK5R1,WASF1,WASL,SLIT2,CKAP5,FCHSD2,RHOBTB1,RANBP9,ARPC5,ARPC2,ABI2,TENM1,BAIAP2,ARFGEF1,NCKAP1,WASF3,TPPP,MAPRE1,CLASP2,KANK1,COBL,CAMSAP2,CLASP1,TMOD3,AP1AR,PPP1R9A,TTC17,SPIRE1,SLAIN2,MAP7D3,NDEL1,ARPC5L,NAV3,ARHGAP18,JMY,MTPN,CAMSAP1,SLC39A12,RICTOR |
| 20_Member | GO Biological Processes | GO:0030041 | actin filament polymerization | -9.384634677 | -7.628 | 49/182 | 118,120,375,387,395,829,830,1739,2017,2059,2185,2475,2764,2885,3717,5063,5581,5756,5921,5962,6709,6711,7114,8440,8936,8976,9353,9873,9886,10092,10109,10152,10178,10458,10565,10787,10810,23189,23242,29766,55435,55607,55761,56907,81873,93663,133746,136319,253260 | ADD1,ADD3,ARF1,RHOA,ARHGAP6,CAPZA1,CAPZA2,DLG1,CTTN,EPS8,PTK2B,MTOR,GMFB,GRB2,JAK2,PAK3,PRKCE,TWF1,RASA1,RDX,SPTAN1,SPTBN1,TMSB4X,NCK2,WASF1,WASL,SLIT2,FCHSD2,RHOBTB1,ARPC5,ARPC2,ABI2,TENM1,BAIAP2,ARFGEF1,NCKAP1,WASF3,KANK1,COBL,TMOD3,AP1AR,PPP1R9A,TTC17,SPIRE1,ARPC5L,ARHGAP18,JMY,MTPN,RICTOR |
| 20_Member | GO Biological Processes | GO:0008154 | actin polymerization or depolymerization | -9.062394482 | -7.340 | 53/209 | 118,120,375,387,395,829,830,1073,1739,2017,2059,2185,2475,2764,2885,3717,5063,5581,5756,5921,5962,6709,6711,7114,8440,8936,8976,9037,9353,9873,9886,10006,10092,10109,10152,10178,10458,10565,10787,10810,23189,23242,29766,55435,55607,55740,55761,56907,81873,93663,133746,136319,253260 | ADD1,ADD3,ARF1,RHOA,ARHGAP6,CAPZA1,CAPZA2,CFL2,DLG1,CTTN,EPS8,PTK2B,MTOR,GMFB,GRB2,JAK2,PAK3,PRKCE,TWF1,RASA1,RDX,SPTAN1,SPTBN1,TMSB4X,NCK2,WASF1,WASL,SEMA5A,SLIT2,FCHSD2,RHOBTB1,ABI1,ARPC5,ARPC2,ABI2,TENM1,BAIAP2,ARFGEF1,NCKAP1,WASF3,KANK1,COBL,TMOD3,AP1AR,PPP1R9A,ENAH,TTC17,SPIRE1,ARPC5L,ARHGAP18,JMY,MTPN,RICTOR |
| 20_Member | GO Biological Processes | GO:0030833 | regulation of actin filament polymerization | -8.62902354 | -6.944 | 44/162 | 118,120,375,387,829,830,1739,2017,2059,2185,2475,2764,2885,5063,5581,5756,5921,5962,6709,6711,7114,8440,8936,8976,9353,9873,9886,10092,10109,10152,10178,10458,10565,10787,23189,29766,55435,55607,56907,81873,93663,133746,136319,253260 | ADD1,ADD3,ARF1,RHOA,CAPZA1,CAPZA2,DLG1,CTTN,EPS8,PTK2B,MTOR,GMFB,GRB2,PAK3,PRKCE,TWF1,RASA1,RDX,SPTAN1,SPTBN1,TMSB4X,NCK2,WASF1,WASL,SLIT2,FCHSD2,RHOBTB1,ARPC5,ARPC2,ABI2,TENM1,BAIAP2,ARFGEF1,NCKAP1,KANK1,TMOD3,AP1AR,PPP1R9A,SPIRE1,ARPC5L,ARHGAP18,JMY,MTPN,RICTOR |
| 20_Member | GO Biological Processes | GO:0008064 | regulation of actin polymerization or depolymerization | -8.154826865 | -6.519 | 46/179 | 118,120,375,387,829,830,1073,1739,2017,2059,2185,2475,2764,2885,5063,5581,5756,5921,5962,6709,6711,7114,8440,8936,8976,9037,9353,9873,9886,10092,10109,10152,10178,10458,10565,10787,23189,29766,55435,55607,56907,81873,93663,133746,136319,253260 | ADD1,ADD3,ARF1,RHOA,CAPZA1,CAPZA2,CFL2,DLG1,CTTN,EPS8,PTK2B,MTOR,GMFB,GRB2,PAK3,PRKCE,TWF1,RASA1,RDX,SPTAN1,SPTBN1,TMSB4X,NCK2,WASF1,WASL,SEMA5A,SLIT2,FCHSD2,RHOBTB1,ARPC5,ARPC2,ABI2,TENM1,BAIAP2,ARFGEF1,NCKAP1,KANK1,TMOD3,AP1AR,PPP1R9A,SPIRE1,ARPC5L,ARHGAP18,JMY,MTPN,RICTOR |
| 20_Member | GO Biological Processes | GO:0030832 | regulation of actin filament length | -8.072883792 | -6.445 | 46/180 | 118,120,375,387,829,830,1073,1739,2017,2059,2185,2475,2764,2885,5063,5581,5756,5921,5962,6709,6711,7114,8440,8936,8976,9037,9353,9873,9886,10092,10109,10152,10178,10458,10565,10787,23189,29766,55435,55607,56907,81873,93663,133746,136319,253260 | ADD1,ADD3,ARF1,RHOA,CAPZA1,CAPZA2,CFL2,DLG1,CTTN,EPS8,PTK2B,MTOR,GMFB,GRB2,PAK3,PRKCE,TWF1,RASA1,RDX,SPTAN1,SPTBN1,TMSB4X,NCK2,WASF1,WASL,SEMA5A,SLIT2,FCHSD2,RHOBTB1,ARPC5,ARPC2,ABI2,TENM1,BAIAP2,ARFGEF1,NCKAP1,KANK1,TMOD3,AP1AR,PPP1R9A,SPIRE1,ARPC5L,ARHGAP18,JMY,MTPN,RICTOR |
| 20_Member | GO Biological Processes | GO:1902905 | positive regulation of supramolecular fiber organization | -8.057943442 | -6.432 | 50/204 | 25,351,375,387,998,1027,1073,1490,1739,2017,2185,2475,2764,2885,3984,4131,5581,5629,5829,6683,7046,7168,7248,8440,8829,8851,8936,8976,9037,9475,9873,9886,10092,10109,10152,10178,10458,10787,22843,22919,23332,27302,55435,56907,57606,81873,89795,133746,171024,253260 | ABL1,APP,ARF1,RHOA,CDC42,CDKN1B,CFL2,CCN2,DLG1,CTTN,PTK2B,MTOR,GMFB,GRB2,LIMK1,MAP1B,PRKCE,PROX1,PXN,SPAST,TGFBR1,TPM1,TSC1,NCK2,NRP1,CDK5R1,WASF1,WASL,SEMA5A,ROCK2,FCHSD2,RHOBTB1,ARPC5,ARPC2,ABI2,TENM1,BAIAP2,NCKAP1,PPM1E,MAPRE1,CLASP1,BMP10,AP1AR,SPIRE1,SLAIN2,ARPC5L,NAV3,JMY,SYNPO2,RICTOR |
| 20_Member | GO Biological Processes | GO:0051495 | positive regulation of cytoskeleton organization | -6.550102113 | -5.063 | 50/226 | 25,375,387,998,1027,1073,1490,1739,2017,2185,2475,2764,2885,3984,4131,5581,5629,5829,6683,7046,7168,7248,8440,8829,8851,8936,8976,9037,9475,9873,9886,10092,10109,10152,10178,10458,10787,22843,22919,23332,27302,55435,56907,57606,81873,89795,133746,137886,171024,253260 | ABL1,ARF1,RHOA,CDC42,CDKN1B,CFL2,CCN2,DLG1,CTTN,PTK2B,MTOR,GMFB,GRB2,LIMK1,MAP1B,PRKCE,PROX1,PXN,SPAST,TGFBR1,TPM1,TSC1,NCK2,NRP1,CDK5R1,WASF1,WASL,SEMA5A,ROCK2,FCHSD2,RHOBTB1,ARPC5,ARPC2,ABI2,TENM1,BAIAP2,NCKAP1,PPM1E,MAPRE1,CLASP1,BMP10,AP1AR,SPIRE1,SLAIN2,ARPC5L,NAV3,JMY,UBXN2B,SYNPO2,RICTOR |
| 20_Member | GO Biological Processes | GO:0031334 | positive regulation of protein complex assembly | -6.324653334 | -4.865 | 56/269 | 375,387,1027,1385,1482,1499,1739,2017,2185,2475,2764,2885,2965,3725,4131,4286,4478,4848,5581,6722,6928,7291,8440,8851,8936,8976,9873,9886,9908,10092,10109,10146,10152,10178,10458,10497,10787,11076,11124,22919,23064,23332,50807,51100,55435,55832,56907,57472,57600,57606,81873,89795,133746,246175,253260,255967 | ARF1,RHOA,CDKN1B,CREB1,NKX2-5,CTNNB1,DLG1,CTTN,PTK2B,MTOR,GMFB,GRB2,GTF2H1,JUN,MAP1B,MITF,MSN,CNOT2,PRKCE,SRF,HNF1B,TWIST1,NCK2,CDK5R1,WASF1,WASL,FCHSD2,RHOBTB1,G3BP2,ARPC5,ARPC2,G3BP1,ABI2,TENM1,BAIAP2,UNC13B,NCKAP1,TPPP,FAF1,MAPRE1,SETX,CLASP1,ASAP1,SH3GLB1,AP1AR,CAND1,SPIRE1,CNOT6,FNIP2,SLAIN2,ARPC5L,NAV3,JMY,CNOT6L,RICTOR,PAN3 |
| 20_Member | GO Biological Processes | GO:0032273 | positive regulation of protein polymerization | -5.603294939 | -4.221 | 33/134 | 375,387,1027,1739,2017,2185,2475,2764,2885,4131,5581,8440,8851,8936,8976,9873,9886,10092,10109,10152,10178,10458,10787,11076,22919,23332,55435,56907,57606,81873,89795,133746,253260 | ARF1,RHOA,CDKN1B,DLG1,CTTN,PTK2B,MTOR,GMFB,GRB2,MAP1B,PRKCE,NCK2,CDK5R1,WASF1,WASL,FCHSD2,RHOBTB1,ARPC5,ARPC2,ABI2,TENM1,BAIAP2,NCKAP1,TPPP,MAPRE1,CLASP1,AP1AR,SPIRE1,SLAIN2,ARPC5L,NAV3,JMY,RICTOR |
| 20_Member | GO Biological Processes | GO:0030838 | positive regulation of actin filament polymerization | -4.60154138 | -3.325 | 25/99 | 375,387,1739,2017,2185,2475,2764,2885,5581,8440,8936,8976,9873,9886,10092,10109,10152,10178,10458,10787,55435,56907,81873,133746,253260 | ARF1,RHOA,DLG1,CTTN,PTK2B,MTOR,GMFB,GRB2,PRKCE,NCK2,WASF1,WASL,FCHSD2,RHOBTB1,ARPC5,ARPC2,ABI2,TENM1,BAIAP2,NCKAP1,AP1AR,SPIRE1,ARPC5L,JMY,RICTOR |
