# Supplementary material for: MiRNA Profiling in Pectoral Muscle Throughout Pre- to Post-Natal Stages of Chicken Development
Source: Front Genet. 2020 Jun 23;11:570. doi: 10.3389/fgene.2020.00570 (PMC7324647; doi:10.3389/fgene.2020.00570)
Supplement: Supplementary file 1 [file Data_Sheet_1.ZIP › Supplementary Material/table S6 KEGG metascape_result.docx]

| **GroupID** | **Category** | **Term** | **Description** | **LogP** | **Log(q-value)** | **InTerm_InList** | **Genes** | **Symbols** |
| --- | --- | --- | --- | --- | --- | --- | --- | --- |
| 1_Summary | KEGG Pathway | hsa04010 | MAPK signaling pathway | -18.58278682 | -15.889 | 79/255 | 775,776,781,783,998,1326,1386,1398,1399,1843,1847,1848,1956,2005,2122,2252,2255,2259,2261,2263,2353,2885,3725,3845,4208,4214,4215,4216,4763,4775,4803,4915,5062,5155,5156,5494,5495,5530,5534,5567,5578,5594,5599,5604,5801,5908,5921,5922,5924,6195,6196,6197,6416,6654,6655,6722,6788,6885,7042,7046,7048,7186,8491,8817,9252,9254,9448,9693,10000,10746,11221,22800,23118,23162,26281,27330,51701,55799,57551 | CACNA1C,CACNA1D,CACNA2D1,CACNB2,CDC42,MAP3K8,ATF2,CRK,CRKL,DUSP1,DUSP5,DUSP6,EGFR,ELK4,MECOM,FGF7,FGF10,FGF14,FGFR3,FGFR2,FOS,GRB2,JUN,KRAS,MEF2C,MAP3K1,MAP3K3,MAP3K4,NF1,NFATC3,NGF,NTRK2,PAK2,PDGFB,PDGFRA,PPM1A,PPM1B,PPP3CA,PPP3R1,PRKACB,PRKCA,MAPK1,MAPK8,MAP2K1,PTPRR,RAP1B,RASA1,RASA2,RASGRF2,RPS6KA1,RPS6KA2,RPS6KA3,MAP2K4,SOS1,SOS2,SRF,STK3,MAP3K7,TGFB2,TGFBR1,TGFBR2,TRAF2,MAP4K3,FGF18,RPS6KA5,CACNA2D2,MAP4K4,RAPGEF2,AKT3,MAP3K2,DUSP10,RRAS2,TAB2,MAPK8IP3,FGF20,RPS6KA6,NLK,CACNA2D3,TAOK1 |
| 1_Member | KEGG Pathway | hsa04010 | MAPK signaling pathway | -18.58278682 | -15.889 | 79/255 | 775,776,781,783,998,1326,1386,1398,1399,1843,1847,1848,1956,2005,2122,2252,2255,2259,2261,2263,2353,2885,3725,3845,4208,4214,4215,4216,4763,4775,4803,4915,5062,5155,5156,5494,5495,5530,5534,5567,5578,5594,5599,5604,5801,5908,5921,5922,5924,6195,6196,6197,6416,6654,6655,6722,6788,6885,7042,7046,7048,7186,8491,8817,9252,9254,9448,9693,10000,10746,11221,22800,23118,23162,26281,27330,51701,55799,57551 | CACNA1C,CACNA1D,CACNA2D1,CACNB2,CDC42,MAP3K8,ATF2,CRK,CRKL,DUSP1,DUSP5,DUSP6,EGFR,ELK4,MECOM,FGF7,FGF10,FGF14,FGFR3,FGFR2,FOS,GRB2,JUN,KRAS,MEF2C,MAP3K1,MAP3K3,MAP3K4,NF1,NFATC3,NGF,NTRK2,PAK2,PDGFB,PDGFRA,PPM1A,PPM1B,PPP3CA,PPP3R1,PRKACB,PRKCA,MAPK1,MAPK8,MAP2K1,PTPRR,RAP1B,RASA1,RASA2,RASGRF2,RPS6KA1,RPS6KA2,RPS6KA3,MAP2K4,SOS1,SOS2,SRF,STK3,MAP3K7,TGFB2,TGFBR1,TGFBR2,TRAF2,MAP4K3,FGF18,RPS6KA5,CACNA2D2,MAP4K4,RAPGEF2,AKT3,MAP3K2,DUSP10,RRAS2,TAB2,MAPK8IP3,FGF20,RPS6KA6,NLK,CACNA2D3,TAOK1 |
| 2_Summary | KEGG Pathway | hsa04140 | Autophagy - animal | -14.45736501 | -12.065 | 47/128 | 664,1965,2081,2475,3146,3708,3845,3920,5170,5291,5295,5515,5516,5563,5567,5588,5594,5599,5604,5728,6009,6198,6885,7248,7879,8408,8660,8678,8897,9110,9451,9474,9706,9821,10000,10533,22800,29982,51100,53349,55054,55062,55102,57521,58476,64422,83452 | BNIP3,EIF2S1,ERN1,MTOR,HMGB1,ITPR1,KRAS,LAMP2,PDPK1,PIK3CB,PIK3R1,PPP2CA,PPP2CB,PRKAA2,PRKACB,PRKCQ,MAPK1,MAPK8,MAP2K1,PTEN,RHEB,RPS6KB1,MAP3K7,TSC1,RAB7A,ULK1,IRS2,BECN1,MTMR3,MTMR4,EIF2AK3,ATG5,ULK2,RB1CC1,AKT3,ATG7,RRAS2,NRBF2,SH3GLB1,ZFYVE1,ATG16L1,WIPI1,ATG2B,RPTOR,TP53INP2,ATG3,RAB33B |
| 2_Member | KEGG Pathway | hsa04140 | Autophagy - animal | -14.45736501 | -12.065 | 47/128 | 664,1965,2081,2475,3146,3708,3845,3920,5170,5291,5295,5515,5516,5563,5567,5588,5594,5599,5604,5728,6009,6198,6885,7248,7879,8408,8660,8678,8897,9110,9451,9474,9706,9821,10000,10533,22800,29982,51100,53349,55054,55062,55102,57521,58476,64422,83452 | BNIP3,EIF2S1,ERN1,MTOR,HMGB1,ITPR1,KRAS,LAMP2,PDPK1,PIK3CB,PIK3R1,PPP2CA,PPP2CB,PRKAA2,PRKACB,PRKCQ,MAPK1,MAPK8,MAP2K1,PTEN,RHEB,RPS6KB1,MAP3K7,TSC1,RAB7A,ULK1,IRS2,BECN1,MTMR3,MTMR4,EIF2AK3,ATG5,ULK2,RB1CC1,AKT3,ATG7,RRAS2,NRBF2,SH3GLB1,ZFYVE1,ATG16L1,WIPI1,ATG2B,RPTOR,TP53INP2,ATG3,RAB33B |
| 2_Member | KEGG Pathway | hsa04136 | Autophagy - other | -4.264881057 | -3.469 | 12/32 | 2475,5515,5516,8678,9474,9706,10533,55054,55062,55102,57521,64422 | MTOR,PPP2CA,PPP2CB,BECN1,ATG5,ULK2,ATG7,ATG16L1,WIPI1,ATG2B,RPTOR,ATG3 |
| 3_Summary | KEGG Pathway | hsa04390 | Hippo signaling pathway | -14.16975803 | -11.953 | 52/154 | 60,324,650,654,655,657,659,894,1490,1499,1739,1740,1857,2736,3398,4086,5501,5515,5516,5520,5522,5584,6657,6788,6934,7003,7042,7046,7048,7159,7161,7529,7531,7532,7533,7534,8312,8313,8321,8325,10207,10971,23286,23291,23418,25937,26524,29119,55844,64398,84612,85407 | ACTB,APC,BMP2,BMP6,BMP7,BMPR1A,BMPR2,CCND2,CCN2,CTNNB1,DLG1,DLG2,DVL3,GLI2,ID2,SMAD1,PPP1CC,PPP2CA,PPP2CB,PPP2R2A,PPP2R2C,PRKCI,SOX2,STK3,TCF7L2,TEAD1,TGFB2,TGFBR1,TGFBR2,TP53BP2,TP73,YWHAB,YWHAE,YWHAG,YWHAH,YWHAZ,AXIN1,AXIN2,FZD1,FZD8,PATJ,YWHAQ,WWC1,FBXW11,CRB1,WWTR1,LATS2,CTNNA3,PPP2R2D,MPP5,PARD6B,NKD1 |
| 3_Member | KEGG Pathway | hsa04390 | Hippo signaling pathway | -14.16975803 | -11.953 | 52/154 | 60,324,650,654,655,657,659,894,1490,1499,1739,1740,1857,2736,3398,4086,5501,5515,5516,5520,5522,5584,6657,6788,6934,7003,7042,7046,7048,7159,7161,7529,7531,7532,7533,7534,8312,8313,8321,8325,10207,10971,23286,23291,23418,25937,26524,29119,55844,64398,84612,85407 | ACTB,APC,BMP2,BMP6,BMP7,BMPR1A,BMPR2,CCND2,CCN2,CTNNB1,DLG1,DLG2,DVL3,GLI2,ID2,SMAD1,PPP1CC,PPP2CA,PPP2CB,PPP2R2A,PPP2R2C,PRKCI,SOX2,STK3,TCF7L2,TEAD1,TGFB2,TGFBR1,TGFBR2,TP53BP2,TP73,YWHAB,YWHAE,YWHAG,YWHAH,YWHAZ,AXIN1,AXIN2,FZD1,FZD8,PATJ,YWHAQ,WWC1,FBXW11,CRB1,WWTR1,LATS2,CTNNA3,PPP2R2D,MPP5,PARD6B,NKD1 |
| 4_Summary | KEGG Pathway | hsa05200 | Pathways in cancer | -13.69131564 | -11.600 | 94/395 | 25,108,324,367,387,650,862,868,998,1021,1027,1164,1282,1287,1387,1398,1399,1499,1857,1869,1871,1956,2033,2113,2122,2252,2255,2259,2261,2263,2277,2308,2335,2353,2475,2736,2737,2776,2782,2885,3082,3688,3725,3815,3845,3915,4286,5155,5156,5291,5295,5332,5567,5578,5594,5599,5604,5727,5728,5898,5900,6093,6256,6502,6654,6655,6921,6934,7042,7046,7048,7170,7186,7424,7852,8030,8312,8313,8321,8325,8817,9475,10000,10342,10672,10928,23236,26060,26281,29119,54583,59345,64399,284217,27,284,801,805,1946,2549,2903,2904,3643,4301,4763,4803,5062,5063,5868,5878,5908,5921,5922,5924,7074,8036,9462,9771,22800,23179,29110,56034,399694,60,1073,1730,3680,3696,3984,4478,4627,4628,4638,4659,5305,5501,5829,5962,7114,7430,8395,8936,8976,10092,10109,10152,10458,10787,26999,55740,81873,1268,5584,7057,9223,9693,9732,9863,10636,57568,83660,84612,260425,894,1278,1297,1299,1385,1386,1977,2998,3717,5105,5170,5515,5516,5520,5522,5523,5527,5529,5563,5586,5618,5649,6009,6198,7248,7529,7531,7532,7533,7534,9586,10971,23239,55844,57521,64764,287,288,817,818,858,960,1634,1655,2017,2066,3708,6383,7291,10818,27250,29102,81578,394,2534 | ABL1,ADCY2,APC,AR,RHOA,BMP2,RUNX1T1,CBLB,CDC42,CDK6,CDKN1B,CKS2,COL4A1,COL4A5,CREBBP,CRK,CRKL,CTNNB1,DVL3,E2F1,E2F3,EGFR,EP300,ETS1,MECOM,FGF7,FGF10,FGF14,FGFR3,FGFR2,VEGFD,FOXO1,FN1,FOS,MTOR,GLI2,GLI3,GNAQ,GNB1,GRB2,HGF,ITGB1,JUN,KIT,KRAS,LAMC1,MITF,PDGFB,PDGFRA,PIK3CB,PIK3R1,PLCB4,PRKACB,PRKCA,MAPK1,MAPK8,MAP2K1,PTCH1,PTEN,RALA,RALGDS,ROCK1,RXRA,SKP2,SOS1,SOS2,ELOC,TCF7L2,TGFB2,TGFBR1,TGFBR2,TPM3,TRAF2,VEGFC,CXCR4,CCDC6,AXIN1,AXIN2,FZD1,FZD8,FGF18,ROCK2,AKT3,TFG,GNA13,RALBP1,PLCB1,APPL1,FGF20,CTNNA3,EGLN1,GNB4,HHIP,LAMA1,ABL2,ANGPT1,CALM1,CALM2,EFNA5,GAB1,GRIN2A,GRIN2B,INSR,AFDN,NF1,NGF,PAK2,PAK3,RAB5A,RAB5C,RAP1B,RASA1,RASA2,RASGRF2,TIAM1,SHOC2,RASAL2,RAPGEF5,RRAS2,RGL1,TBK1,PDGFC,SHC4,ACTB,CFL2,DIAPH2,ITGA9,ITGB8,LIMK1,MSN,MYH9,MYH10,MYLK,PPP1R12A,PIP4K2A,PPP1CC,PXN,RDX,TMSB4X,EZR,PIP5K1B,WASF1,WASL,ARPC5,ARPC2,ABI2,BAIAP2,NCKAP1,CYFIP2,ENAH,ARPC5L,CNR1,PRKCI,THBS1,MAGI1,RAPGEF2,DOCK4,MAGI2,RGS14,SIPA1L2,TLN2,PARD6B,MAGI3,CCND2,COL1A2,COL9A1,COL9A3,CREB1,ATF2,EIF4E,GYS2,JAK2,PCK1,PDPK1,PPP2CA,PPP2CB,PPP2R2A,PPP2R2C,PPP2R3A,PPP2R5C,PPP2R5E,PRKAA2,PKN2,PRLR,RELN,RHEB,RPS6KB1,TSC1,YWHAB,YWHAE,YWHAG,YWHAH,YWHAZ,CREB5,YWHAQ,PHLPP1,PPP2R2D,RPTOR,CREB3L2,ANK2,ANK3,CAMK2D,CAMK2G,CAV2,CD44,DCN,DDX5,CTTN,ERBB4,ITPR1,SDC2,TWIST1,FRS2,PDCD4,DROSHA,COL21A1,ARHGAP5,FYN |
| 4_Member | KEGG Pathway | hsa05200 | Pathways in cancer | -13.69131564 | -11.600 | 94/395 | 25,108,324,367,387,650,862,868,998,1021,1027,1164,1282,1287,1387,1398,1399,1499,1857,1869,1871,1956,2033,2113,2122,2252,2255,2259,2261,2263,2277,2308,2335,2353,2475,2736,2737,2776,2782,2885,3082,3688,3725,3815,3845,3915,4286,5155,5156,5291,5295,5332,5567,5578,5594,5599,5604,5727,5728,5898,5900,6093,6256,6502,6654,6655,6921,6934,7042,7046,7048,7170,7186,7424,7852,8030,8312,8313,8321,8325,8817,9475,10000,10342,10672,10928,23236,26060,26281,29119,54583,59345,64399,284217 | ABL1,ADCY2,APC,AR,RHOA,BMP2,RUNX1T1,CBLB,CDC42,CDK6,CDKN1B,CKS2,COL4A1,COL4A5,CREBBP,CRK,CRKL,CTNNB1,DVL3,E2F1,E2F3,EGFR,EP300,ETS1,MECOM,FGF7,FGF10,FGF14,FGFR3,FGFR2,VEGFD,FOXO1,FN1,FOS,MTOR,GLI2,GLI3,GNAQ,GNB1,GRB2,HGF,ITGB1,JUN,KIT,KRAS,LAMC1,MITF,PDGFB,PDGFRA,PIK3CB,PIK3R1,PLCB4,PRKACB,PRKCA,MAPK1,MAPK8,MAP2K1,PTCH1,PTEN,RALA,RALGDS,ROCK1,RXRA,SKP2,SOS1,SOS2,ELOC,TCF7L2,TGFB2,TGFBR1,TGFBR2,TPM3,TRAF2,VEGFC,CXCR4,CCDC6,AXIN1,AXIN2,FZD1,FZD8,FGF18,ROCK2,AKT3,TFG,GNA13,RALBP1,PLCB1,APPL1,FGF20,CTNNA3,EGLN1,GNB4,HHIP,LAMA1 |
| 4_Member | KEGG Pathway | hsa04014 | Ras signaling pathway | -13.0358311 | -11.120 | 64/227 | 25,27,284,387,801,805,998,1946,1956,2113,2252,2255,2259,2261,2263,2277,2549,2782,2885,2903,2904,3082,3643,3815,3845,4301,4763,4803,5062,5063,5155,5156,5291,5295,5567,5578,5594,5599,5604,5868,5878,5898,5900,5908,5921,5922,5924,6654,6655,7074,7424,8036,8817,9462,9771,10000,10928,22800,23179,26281,29110,56034,59345,399694 | ABL1,ABL2,ANGPT1,RHOA,CALM1,CALM2,CDC42,EFNA5,EGFR,ETS1,FGF7,FGF10,FGF14,FGFR3,FGFR2,VEGFD,GAB1,GNB1,GRB2,GRIN2A,GRIN2B,HGF,INSR,KIT,KRAS,AFDN,NF1,NGF,PAK2,PAK3,PDGFB,PDGFRA,PIK3CB,PIK3R1,PRKACB,PRKCA,MAPK1,MAPK8,MAP2K1,RAB5A,RAB5C,RALA,RALGDS,RAP1B,RASA1,RASA2,RASGRF2,SOS1,SOS2,TIAM1,VEGFC,SHOC2,FGF18,RASAL2,RAPGEF5,AKT3,RALBP1,RRAS2,RGL1,FGF20,TBK1,PDGFC,GNB4,SHC4 |
| 4_Member | KEGG Pathway | hsa04810 | Regulation of actin cytoskeleton | -12.34477661 | -10.554 | 60/212 | 60,324,387,998,1073,1398,1399,1730,1956,2252,2255,2259,2261,2263,2335,3680,3688,3696,3845,3984,4478,4627,4628,4638,4659,5062,5063,5155,5156,5291,5295,5305,5501,5594,5604,5829,5962,6093,6654,6655,7074,7114,7430,8395,8817,8936,8976,9475,10092,10109,10152,10458,10672,10787,22800,26281,26999,55740,56034,81873 | ACTB,APC,RHOA,CDC42,CFL2,CRK,CRKL,DIAPH2,EGFR,FGF7,FGF10,FGF14,FGFR3,FGFR2,FN1,ITGA9,ITGB1,ITGB8,KRAS,LIMK1,MSN,MYH9,MYH10,MYLK,PPP1R12A,PAK2,PAK3,PDGFB,PDGFRA,PIK3CB,PIK3R1,PIP4K2A,PPP1CC,MAPK1,MAP2K1,PXN,RDX,ROCK1,SOS1,SOS2,TIAM1,TMSB4X,EZR,PIP5K1B,FGF18,WASF1,WASL,ROCK2,ARPC5,ARPC2,ABI2,BAIAP2,GNA13,NCKAP1,RRAS2,FGF20,CYFIP2,ENAH,PDGFC,ARPC5L |
| 4_Member | KEGG Pathway | hsa04015 | Rap1 signaling pathway | -12.00218713 | -10.308 | 59/210 | 60,108,284,387,801,805,998,1268,1398,1399,1499,1946,1956,2252,2255,2259,2261,2263,2277,2776,2903,2904,3082,3643,3688,3815,3845,4301,4803,5155,5156,5291,5295,5332,5578,5584,5594,5604,5898,5900,5908,7057,7074,7424,8817,9223,9693,9732,9771,9863,10000,10636,23236,26281,56034,57568,83660,84612,260425 | ACTB,ADCY2,ANGPT1,RHOA,CALM1,CALM2,CDC42,CNR1,CRK,CRKL,CTNNB1,EFNA5,EGFR,FGF7,FGF10,FGF14,FGFR3,FGFR2,VEGFD,GNAQ,GRIN2A,GRIN2B,HGF,INSR,ITGB1,KIT,KRAS,AFDN,NGF,PDGFB,PDGFRA,PIK3CB,PIK3R1,PLCB4,PRKCA,PRKCI,MAPK1,MAP2K1,RALA,RALGDS,RAP1B,THBS1,TIAM1,VEGFC,FGF18,MAGI1,RAPGEF2,DOCK4,RAPGEF5,MAGI2,AKT3,RGS14,PLCB1,FGF20,PDGFC,SIPA1L2,TLN2,PARD6B,MAGI3 |
| 4_Member | KEGG Pathway | hsa04151 | PI3K-Akt signaling pathway | -11.77386995 | -10.122 | 81/342 | 284,894,1021,1027,1278,1282,1287,1297,1299,1385,1386,1946,1956,1977,2252,2255,2259,2261,2263,2277,2335,2475,2782,2885,2998,3082,3643,3680,3688,3696,3717,3815,3845,3915,4803,5105,5155,5156,5170,5291,5295,5515,5516,5520,5522,5523,5527,5529,5563,5578,5586,5594,5604,5618,5649,5728,6009,6198,6256,6654,6655,7057,7248,7424,7529,7531,7532,7533,7534,8817,9586,10000,10971,23239,26281,55844,56034,57521,59345,64764,284217 | ANGPT1,CCND2,CDK6,CDKN1B,COL1A2,COL4A1,COL4A5,COL9A1,COL9A3,CREB1,ATF2,EFNA5,EGFR,EIF4E,FGF7,FGF10,FGF14,FGFR3,FGFR2,VEGFD,FN1,MTOR,GNB1,GRB2,GYS2,HGF,INSR,ITGA9,ITGB1,ITGB8,JAK2,KIT,KRAS,LAMC1,NGF,PCK1,PDGFB,PDGFRA,PDPK1,PIK3CB,PIK3R1,PPP2CA,PPP2CB,PPP2R2A,PPP2R2C,PPP2R3A,PPP2R5C,PPP2R5E,PRKAA2,PRKCA,PKN2,MAPK1,MAP2K1,PRLR,RELN,PTEN,RHEB,RPS6KB1,RXRA,SOS1,SOS2,THBS1,TSC1,VEGFC,YWHAB,YWHAE,YWHAG,YWHAH,YWHAZ,FGF18,CREB5,AKT3,YWHAQ,PHLPP1,FGF20,PPP2R2D,PDGFC,RPTOR,GNB4,CREB3L2,LAMA1 |
| 4_Member | KEGG Pathway | hsa05205 | Proteoglycans in cancer | -11.07946627 | -9.562 | 56/203 | 60,287,288,387,817,818,858,868,960,998,1499,1634,1655,1956,2017,2066,2335,2475,2549,2885,3082,3688,3708,3845,4478,4659,5170,5291,5295,5501,5567,5578,5594,5604,5727,5829,5962,6093,6198,6383,6654,6655,7042,7057,7074,7291,7430,8321,8325,9475,10000,10818,22800,27250,29102,81578 | ACTB,ANK2,ANK3,RHOA,CAMK2D,CAMK2G,CAV2,CBLB,CD44,CDC42,CTNNB1,DCN,DDX5,EGFR,CTTN,ERBB4,FN1,MTOR,GAB1,GRB2,HGF,ITGB1,ITPR1,KRAS,MSN,PPP1R12A,PDPK1,PIK3CB,PIK3R1,PPP1CC,PRKACB,PRKCA,MAPK1,MAP2K1,PTCH1,PXN,RDX,ROCK1,RPS6KB1,SDC2,SOS1,SOS2,TGFB2,THBS1,TIAM1,TWIST1,EZR,FZD1,FZD8,ROCK2,AKT3,FRS2,RRAS2,PDCD4,DROSHA,COL21A1 |
| 4_Member | KEGG Pathway | hsa04510 | Focal adhesion | -10.41037318 | -8.994 | 54/199 | 60,387,394,858,894,998,1278,1282,1287,1297,1299,1398,1399,1499,1956,2277,2335,2534,2885,3082,3680,3688,3696,3725,3915,4638,4659,5062,5063,5155,5156,5170,5291,5295,5501,5578,5594,5599,5604,5649,5728,5829,5908,6093,6654,6655,7057,7424,9475,10000,56034,83660,284217,399694 | ACTB,RHOA,ARHGAP5,CAV2,CCND2,CDC42,COL1A2,COL4A1,COL4A5,COL9A1,COL9A3,CRK,CRKL,CTNNB1,EGFR,VEGFD,FN1,FYN,GRB2,HGF,ITGA9,ITGB1,ITGB8,JUN,LAMC1,MYLK,PPP1R12A,PAK2,PAK3,PDGFB,PDGFRA,PDPK1,PIK3CB,PIK3R1,PPP1CC,PRKCA,MAPK1,MAPK8,MAP2K1,RELN,PTEN,PXN,RAP1B,ROCK1,SOS1,SOS2,THBS1,VEGFC,ROCK2,AKT3,PDGFC,TLN2,LAMA1,SHC4 |
| 5_Summary | KEGG Pathway | hsa04360 | Axon guidance | -13.44195195 | -11.447 | 55/175 | 25,387,655,659,817,818,998,1073,1808,1946,2043,2044,2045,2048,2534,3688,3845,3983,3984,4773,4775,5062,5063,5291,5295,5361,5362,5530,5534,5578,5594,5727,5921,6091,6092,6093,7223,7224,7852,8440,8633,8829,9037,9353,9475,9723,10507,22854,55740,57522,57556,57689,59277,80031,84612 | ABL1,RHOA,BMP7,BMPR2,CAMK2D,CAMK2G,CDC42,CFL2,DPYSL2,EFNA5,EPHA4,EPHA5,EPHA7,EPHB2,FYN,ITGB1,KRAS,ABLIM1,LIMK1,NFATC2,NFATC3,PAK2,PAK3,PIK3CB,PIK3R1,PLXNA1,PLXNA2,PPP3CA,PPP3R1,PRKCA,MAPK1,PTCH1,RASA1,ROBO1,ROBO2,ROCK1,TRPC4,TRPC5,CXCR4,NCK2,UNC5C,NRP1,SEMA5A,SLIT2,ROCK2,SEMA3E,SEMA4D,NTNG1,ENAH,SRGAP1,SEMA6A,LRRC4C,NTN4,SEMA6D,PARD6B |
| 5_Member | KEGG Pathway | hsa04360 | Axon guidance | -13.44195195 | -11.447 | 55/175 | 25,387,655,659,817,818,998,1073,1808,1946,2043,2044,2045,2048,2534,3688,3845,3983,3984,4773,4775,5062,5063,5291,5295,5361,5362,5530,5534,5578,5594,5727,5921,6091,6092,6093,7223,7224,7852,8440,8633,8829,9037,9353,9475,9723,10507,22854,55740,57522,57556,57689,59277,80031,84612 | ABL1,RHOA,BMP7,BMPR2,CAMK2D,CAMK2G,CDC42,CFL2,DPYSL2,EFNA5,EPHA4,EPHA5,EPHA7,EPHB2,FYN,ITGB1,KRAS,ABLIM1,LIMK1,NFATC2,NFATC3,PAK2,PAK3,PIK3CB,PIK3R1,PLXNA1,PLXNA2,PPP3CA,PPP3R1,PRKCA,MAPK1,PTCH1,RASA1,ROBO1,ROBO2,ROCK1,TRPC4,TRPC5,CXCR4,NCK2,UNC5C,NRP1,SEMA5A,SLIT2,ROCK2,SEMA3E,SEMA4D,NTNG1,ENAH,SRGAP1,SEMA6A,LRRC4C,NTN4,SEMA6D,PARD6B |
| 6_Summary | KEGG Pathway | hsa04144 | Endocytosis | -12.59483469 | -10.746 | 69/260 | 163,375,387,829,830,858,868,998,1173,1213,1956,2066,2261,2263,2869,3482,3799,3800,3815,4734,5156,5584,5868,5878,6642,7042,7046,7048,7852,7879,8027,8395,8411,8766,8976,9267,9559,9798,9815,9829,9922,10092,10109,10564,10565,10890,11021,22841,23327,23362,27131,30011,30845,50807,51100,51324,55737,57154,57403,60682,64750,81873,83737,84612,89853,112936,116987,128866,137492 | AP2B1,ARF1,RHOA,CAPZA1,CAPZA2,CAV2,CBLB,CDC42,AP2M1,CLTC,EGFR,ERBB4,FGFR3,FGFR2,GRK5,IGF2R,KIF5B,KIF5C,KIT,NEDD4,PDGFRA,PRKCI,RAB5A,RAB5C,SNX1,TGFB2,TGFBR1,TGFBR2,CXCR4,RAB7A,STAM,PIP5K1B,EEA1,RAB11A,WASL,CYTH1,VPS26A,IST1,GIT2,DNAJC6,IQSEC1,ARPC5,ARPC2,ARFGEF2,ARFGEF1,RAB10,RAB35,RAB11FIP2,NEDD4L,PSD3,SNX5,SH3KBP1,EHD3,ASAP1,SH3GLB1,SPG21,VPS35,SMURF1,RAB22A,SMAP1,SMURF2,ARPC5L,ITCH,PARD6B,MVB12B,VPS26B,AGAP1,CHMP4B,VPS37A |
| 6_Member | KEGG Pathway | hsa04144 | Endocytosis | -12.59483469 | -10.746 | 69/260 | 163,375,387,829,830,858,868,998,1173,1213,1956,2066,2261,2263,2869,3482,3799,3800,3815,4734,5156,5584,5868,5878,6642,7042,7046,7048,7852,7879,8027,8395,8411,8766,8976,9267,9559,9798,9815,9829,9922,10092,10109,10564,10565,10890,11021,22841,23327,23362,27131,30011,30845,50807,51100,51324,55737,57154,57403,60682,64750,81873,83737,84612,89853,112936,116987,128866,137492 | AP2B1,ARF1,RHOA,CAPZA1,CAPZA2,CAV2,CBLB,CDC42,AP2M1,CLTC,EGFR,ERBB4,FGFR3,FGFR2,GRK5,IGF2R,KIF5B,KIF5C,KIT,NEDD4,PDGFRA,PRKCI,RAB5A,RAB5C,SNX1,TGFB2,TGFBR1,TGFBR2,CXCR4,RAB7A,STAM,PIP5K1B,EEA1,RAB11A,WASL,CYTH1,VPS26A,IST1,GIT2,DNAJC6,IQSEC1,ARPC5,ARPC2,ARFGEF2,ARFGEF1,RAB10,RAB35,RAB11FIP2,NEDD4L,PSD3,SNX5,SH3KBP1,EHD3,ASAP1,SH3GLB1,SPG21,VPS35,SMURF1,RAB22A,SMAP1,SMURF2,ARPC5L,ITCH,PARD6B,MVB12B,VPS26B,AGAP1,CHMP4B,VPS37A |
| 7_Summary | KEGG Pathway | hsa04152 | AMPK signaling pathway | -12.17868275 | -10.439 | 42/120 | 890,1080,1374,1385,1994,2308,2475,2998,3156,3643,5105,5170,5214,5291,5295,5515,5516,5520,5522,5523,5527,5529,5563,6009,6198,6319,6885,7248,8408,8660,9586,10000,10890,10891,23411,51552,51719,55844,57521,64764,79602,79966,801,805,868,1398,1399,1977,2885,3845,5140,5501,5567,5573,5576,5577,5584,5594,5599,5604,5792,6654,6655,9021,10580,79660,399694,387,526,528,1857,2887,4040,4041,5578,5728,6195,6196,6197,6502,8321,8325,9706,27330,55004,57600,81929,253260,108,1386,9474,9821,26060,27244,143686,2673,5581,5588,9945,3170 | CCNA2,CFTR,CPT1A,CREB1,ELAVL1,FOXO1,MTOR,GYS2,HMGCR,INSR,PCK1,PDPK1,PFKP,PIK3CB,PIK3R1,PPP2CA,PPP2CB,PPP2R2A,PPP2R2C,PPP2R3A,PPP2R5C,PPP2R5E,PRKAA2,RHEB,RPS6KB1,SCD,MAP3K7,TSC1,ULK1,IRS2,CREB5,AKT3,RAB10,PPARGC1A,SIRT1,RAB14,CAB39,PPP2R2D,RPTOR,CREB3L2,ADIPOR2,SCD5,CALM1,CALM2,CBLB,CRK,CRKL,EIF4E,GRB2,KRAS,PDE3B,PPP1CC,PRKACB,PRKAR1A,PRKAR2A,PRKAR2B,PRKCI,MAPK1,MAPK8,MAP2K1,PTPRF,SOS1,SOS2,SOCS3,SORBS1,PPP1R3B,SHC4,RHOA,ATP6V1B2,ATP6V1C1,DVL3,GRB10,LRP6,LRP5,PRKCA,PTEN,RPS6KA1,RPS6KA2,RPS6KA3,SKP2,FZD1,FZD8,ULK2,RPS6KA6,LAMTOR1,FNIP2,SEH1L,RICTOR,ADCY2,ATF2,ATG5,RB1CC1,APPL1,SESN1,SESN3,GFPT1,PRKCE,PRKCQ,GFPT2,FOXA2 |
| 7_Member | KEGG Pathway | hsa04152 | AMPK signaling pathway | -12.17868275 | -10.439 | 42/120 | 890,1080,1374,1385,1994,2308,2475,2998,3156,3643,5105,5170,5214,5291,5295,5515,5516,5520,5522,5523,5527,5529,5563,6009,6198,6319,6885,7248,8408,8660,9586,10000,10890,10891,23411,51552,51719,55844,57521,64764,79602,79966 | CCNA2,CFTR,CPT1A,CREB1,ELAVL1,FOXO1,MTOR,GYS2,HMGCR,INSR,PCK1,PDPK1,PFKP,PIK3CB,PIK3R1,PPP2CA,PPP2CB,PPP2R2A,PPP2R2C,PPP2R3A,PPP2R5C,PPP2R5E,PRKAA2,RHEB,RPS6KB1,SCD,MAP3K7,TSC1,ULK1,IRS2,CREB5,AKT3,RAB10,PPARGC1A,SIRT1,RAB14,CAB39,PPP2R2D,RPTOR,CREB3L2,ADIPOR2,SCD5 |
| 7_Member | KEGG Pathway | hsa04910 | Insulin signaling pathway | -9.363058391 | -8.012 | 41/138 | 801,805,868,1398,1399,1977,2308,2475,2885,2998,3643,3845,5105,5140,5170,5291,5295,5501,5563,5567,5573,5576,5577,5584,5594,5599,5604,5792,6009,6198,6654,6655,7248,8660,9021,10000,10580,10891,57521,79660,399694 | CALM1,CALM2,CBLB,CRK,CRKL,EIF4E,FOXO1,MTOR,GRB2,GYS2,INSR,KRAS,PCK1,PDE3B,PDPK1,PIK3CB,PIK3R1,PPP1CC,PRKAA2,PRKACB,PRKAR1A,PRKAR2A,PRKAR2B,PRKCI,MAPK1,MAPK8,MAP2K1,PTPRF,RHEB,RPS6KB1,SOS1,SOS2,TSC1,IRS2,SOCS3,AKT3,SORBS1,PPARGC1A,RPTOR,PPP1R3B,SHC4 |
| 7_Member | KEGG Pathway | hsa04150 | mTOR signaling pathway | -8.085100761 | -6.897 | 41/151 | 387,526,528,1857,1977,2475,2885,2887,3643,3845,4040,4041,5170,5291,5295,5563,5578,5594,5604,5728,6009,6195,6196,6197,6198,6502,6654,6655,7248,8321,8325,8408,9706,10000,27330,51719,55004,57521,57600,81929,253260 | RHOA,ATP6V1B2,ATP6V1C1,DVL3,EIF4E,MTOR,GRB2,GRB10,INSR,KRAS,LRP6,LRP5,PDPK1,PIK3CB,PIK3R1,PRKAA2,PRKCA,MAPK1,MAP2K1,PTEN,RHEB,RPS6KA1,RPS6KA2,RPS6KA3,RPS6KB1,SKP2,SOS1,SOS2,TSC1,FZD1,FZD8,ULK1,ULK2,AKT3,RPS6KA6,CAB39,LAMTOR1,RPTOR,FNIP2,SEH1L,RICTOR |
| 7_Member | KEGG Pathway | hsa04211 | Longevity regulating pathway | -7.817272149 | -6.680 | 29/89 | 108,1385,1386,1977,2308,2475,3643,3845,5291,5295,5563,5567,6009,6198,7248,8408,8660,9474,9586,9821,10000,10891,23411,26060,27244,57521,64764,79602,143686 | ADCY2,CREB1,ATF2,EIF4E,FOXO1,MTOR,INSR,KRAS,PIK3CB,PIK3R1,PRKAA2,PRKACB,RHEB,RPS6KB1,TSC1,ULK1,IRS2,ATG5,CREB5,RB1CC1,AKT3,PPARGC1A,SIRT1,APPL1,SESN1,RPTOR,CREB3L2,ADIPOR2,SESN3 |
| 7_Member | KEGG Pathway | hsa04931 | Insulin resistance | -6.973793113 | -5.961 | 31/107 | 1374,1385,2308,2475,2673,2998,3643,5105,5170,5291,5295,5501,5563,5581,5588,5599,5728,5792,6195,6196,6197,6198,8660,9021,9586,9945,10000,10891,27330,64764,79660 | CPT1A,CREB1,FOXO1,MTOR,GFPT1,GYS2,INSR,PCK1,PDPK1,PIK3CB,PIK3R1,PPP1CC,PRKAA2,PRKCE,PRKCQ,MAPK8,PTEN,PTPRF,RPS6KA1,RPS6KA2,RPS6KA3,RPS6KB1,IRS2,SOCS3,CREB5,GFPT2,AKT3,PPARGC1A,RPS6KA6,CREB3L2,PPP1R3B |
| 7_Member | KEGG Pathway | hsa04213 | Longevity regulating pathway - multiple species | -3.27429922 | -2.572 | 16/62 | 108,2308,2475,3170,3643,3845,5291,5295,5563,5567,6198,8660,9474,10000,23411,57521 | ADCY2,FOXO1,MTOR,FOXA2,INSR,KRAS,PIK3CB,PIK3R1,PRKAA2,PRKACB,RPS6KB1,IRS2,ATG5,AKT3,SIRT1,RPTOR |
| 8_Summary | KEGG Pathway | hsa04728 | Dopaminergic synapse | -11.49840936 | -9.884 | 43/130 | 406,775,776,801,805,817,818,1385,1386,1812,1816,2353,2774,2776,2782,2891,2903,2904,3708,3799,3800,5332,5501,5515,5516,5520,5522,5523,5527,5529,5530,5567,5578,5599,6323,6571,9575,9586,10000,23236,55844,59345,64764,1387,2033,2911,2915,3845,5534,5594,5604,5908,6195,6196,6197,27330,108,291,293,387,481,483,488,490,493,2626,2977,2982,3643,4205,4208,4638,4659,4773,4775,5139,5140,5581,5592,6093,6546,6722,8660,9475,10242,10672,781,783,7168,7170,9252,9254,55799,60,1956,3725,3759,3768,5563,57118,998,2185,2885,4214,4215,4216,6416,6654,6655,10746,1374,2308,2998,5105,10891,23411,55671,57223,150094,3358,5155,5156,7082,56034,5291,5295,9568,399694,2534,3717,56479,60482,182,1490,1958,2475,3714,6198,7046,8678,22800,1080,3772,3776,7430,54207,747,4929,51305,2781,2895,1278,1281,3688,5584,83660,1268,2554,2557,2558,2565,22999,57084,1499,1857,3815,4286,6934,8321,8325,2898,2917,9455,9456,22941,54407,800,5588,3781,6616,9699,27445,2066,57620,90550,4803 | ARNTL,CACNA1C,CACNA1D,CALM1,CALM2,CAMK2D,CAMK2G,CREB1,ATF2,DRD1,DRD5,FOS,GNAL,GNAQ,GNB1,GRIA2,GRIN2A,GRIN2B,ITPR1,KIF5B,KIF5C,PLCB4,PPP1CC,PPP2CA,PPP2CB,PPP2R2A,PPP2R2C,PPP2R3A,PPP2R5C,PPP2R5E,PPP3CA,PRKACB,PRKCA,MAPK8,SCN1A,SLC18A2,CLOCK,CREB5,AKT3,PLCB1,PPP2R2D,GNB4,CREB3L2,CREBBP,EP300,GRM1,GRM5,KRAS,PPP3R1,MAPK1,MAP2K1,RAP1B,RPS6KA1,RPS6KA2,RPS6KA3,RPS6KA6,ADCY2,SLC25A4,SLC25A6,RHOA,ATP1B1,ATP1B3,ATP2A2,ATP2B1,ATP2B4,GATA4,GUCY1A2,GUCY1A1,INSR,MEF2A,MEF2C,MYLK,PPP1R12A,NFATC2,NFATC3,PDE3A,PDE3B,PRKCE,PRKG1,ROCK1,SLC8A1,SRF,IRS2,ROCK2,KCNMB2,GNA13,CACNA2D1,CACNB2,TPM1,TPM3,RPS6KA5,CACNA2D2,CACNA2D3,ACTB,EGFR,JUN,KCNJ2,KCNJ12,PRKAA2,CAMK1D,CDC42,PTK2B,GRB2,MAP3K1,MAP3K3,MAP3K4,MAP2K4,SOS1,SOS2,MAP3K2,CPT1A,FOXO1,GYS2,PCK1,PPARGC1A,SIRT1,PPP4R3A,PPP4R3B,SIK1,HTR2C,PDGFB,PDGFRA,TJP1,PDGFC,PIK3CB,PIK3R1,GABBR2,SHC4,FYN,JAK2,KCNQ5,SLC5A7,JAG1,CCN2,EGR1,MTOR,JAG2,RPS6KB1,TGFBR1,BECN1,RRAS2,CFTR,KCNJ15,KCNK2,EZR,KCNK10,DAGLA,NR4A2,KCNK9,GNAZ,GRID2,COL1A2,COL3A1,ITGB1,PRKCI,TLN2,CNR1,GABRA1,GABRA4,GABRA5,GABRG1,RIMS1,SLC17A6,CTNNB1,DVL3,KIT,MITF,TCF7L2,FZD1,FZD8,GRIK2,GRM7,HOMER2,HOMER1,SHANK2,SLC38A2,CALD1,PRKCQ,KCNN2,SNAP25,RIMS2,PCLO,ERBB4,STIM2,MCU,NGF |
| 8_Member | KEGG Pathway | hsa04728 | Dopaminergic synapse | -11.49840936 | -9.884 | 43/130 | 406,775,776,801,805,817,818,1385,1386,1812,1816,2353,2774,2776,2782,2891,2903,2904,3708,3799,3800,5332,5501,5515,5516,5520,5522,5523,5527,5529,5530,5567,5578,5599,6323,6571,9575,9586,10000,23236,55844,59345,64764 | ARNTL,CACNA1C,CACNA1D,CALM1,CALM2,CAMK2D,CAMK2G,CREB1,ATF2,DRD1,DRD5,FOS,GNAL,GNAQ,GNB1,GRIA2,GRIN2A,GRIN2B,ITPR1,KIF5B,KIF5C,PLCB4,PPP1CC,PPP2CA,PPP2CB,PPP2R2A,PPP2R2C,PPP2R3A,PPP2R5C,PPP2R5E,PPP3CA,PRKACB,PRKCA,MAPK8,SCN1A,SLC18A2,CLOCK,CREB5,AKT3,PLCB1,PPP2R2D,GNB4,CREB3L2 |
| 8_Member | KEGG Pathway | hsa04720 | Long-term potentiation | -11.26220227 | -9.715 | 29/67 | 775,801,805,817,818,1387,2033,2776,2891,2903,2904,2911,2915,3708,3845,5332,5501,5530,5534,5567,5578,5594,5604,5908,6195,6196,6197,23236,27330 | CACNA1C,CALM1,CALM2,CAMK2D,CAMK2G,CREBBP,EP300,GNAQ,GRIA2,GRIN2A,GRIN2B,GRM1,GRM5,ITPR1,KRAS,PLCB4,PPP1CC,PPP3CA,PPP3R1,PRKACB,PRKCA,MAPK1,MAP2K1,RAP1B,RPS6KA1,RPS6KA2,RPS6KA3,PLCB1,RPS6KA6 |
| 8_Member | KEGG Pathway | hsa04022 | cGMP-PKG signaling pathway | -10.68712206 | -9.198 | 48/163 | 108,291,293,387,481,483,488,490,493,775,776,801,805,1385,1386,2626,2776,2977,2982,3643,3708,4205,4208,4638,4659,4773,4775,5139,5140,5332,5501,5530,5534,5581,5592,5594,5604,6093,6546,6722,8660,9475,9586,10000,10242,10672,23236,64764 | ADCY2,SLC25A4,SLC25A6,RHOA,ATP1B1,ATP1B3,ATP2A2,ATP2B1,ATP2B4,CACNA1C,CACNA1D,CALM1,CALM2,CREB1,ATF2,GATA4,GNAQ,GUCY1A2,GUCY1A1,INSR,ITPR1,MEF2A,MEF2C,MYLK,PPP1R12A,NFATC2,NFATC3,PDE3A,PDE3B,PLCB4,PPP1CC,PPP3CA,PPP3R1,PRKCE,PRKG1,MAPK1,MAP2K1,ROCK1,SLC8A1,SRF,IRS2,ROCK2,CREB5,AKT3,KCNMB2,GNA13,PLCB1,CREB3L2 |
| 8_Member | KEGG Pathway | hsa04261 | Adrenergic signaling in cardiomyocytes | -8.212858924 | -6.996 | 40/144 | 108,481,483,488,490,493,775,776,781,783,801,805,817,818,1385,1386,2776,5332,5501,5515,5516,5520,5522,5523,5527,5529,5567,5578,5594,6546,7168,7170,9252,9254,9586,10000,23236,55799,55844,64764 | ADCY2,ATP1B1,ATP1B3,ATP2A2,ATP2B1,ATP2B4,CACNA1C,CACNA1D,CACNA2D1,CACNB2,CALM1,CALM2,CAMK2D,CAMK2G,CREB1,ATF2,GNAQ,PLCB4,PPP1CC,PPP2CA,PPP2CB,PPP2R2A,PPP2R2C,PPP2R3A,PPP2R5C,PPP2R5E,PRKACB,PRKCA,MAPK1,SLC8A1,TPM1,TPM3,RPS6KA5,CACNA2D2,CREB5,AKT3,PLCB1,CACNA2D3,PPP2R2D,CREB3L2 |
| 8_Member | KEGG Pathway | hsa04921 | Oxytocin signaling pathway | -7.994720608 | -6.832 | 41/152 | 60,108,387,775,776,781,783,801,805,817,818,1956,2353,2776,2977,2982,3708,3725,3759,3768,3845,4208,4638,4659,4773,4775,5332,5501,5530,5534,5563,5567,5578,5594,5604,6093,9254,9475,23236,55799,57118 | ACTB,ADCY2,RHOA,CACNA1C,CACNA1D,CACNA2D1,CACNB2,CALM1,CALM2,CAMK2D,CAMK2G,EGFR,FOS,GNAQ,GUCY1A2,GUCY1A1,ITPR1,JUN,KCNJ2,KCNJ12,KRAS,MEF2C,MYLK,PPP1R12A,NFATC2,NFATC3,PLCB4,PPP1CC,PPP3CA,PPP3R1,PRKAA2,PRKACB,PRKCA,MAPK1,MAP2K1,ROCK1,CACNA2D2,ROCK2,PLCB1,CACNA2D3,CAMK1D |
| 8_Member | KEGG Pathway | hsa04912 | GnRH signaling pathway | -7.454611591 | -6.374 | 29/92 | 108,775,776,801,805,817,818,998,1956,2185,2776,2885,3708,3725,3845,4214,4215,4216,5332,5567,5578,5594,5599,5604,6416,6654,6655,10746,23236 | ADCY2,CACNA1C,CACNA1D,CALM1,CALM2,CAMK2D,CAMK2G,CDC42,EGFR,PTK2B,GNAQ,GRB2,ITPR1,JUN,KRAS,MAP3K1,MAP3K3,MAP3K4,PLCB4,PRKACB,PRKCA,MAPK1,MAPK8,MAP2K1,MAP2K4,SOS1,SOS2,MAP3K2,PLCB1 |
| 8_Member | KEGG Pathway | hsa04922 | Glucagon signaling pathway | -6.82705383 | -5.824 | 30/103 | 108,801,805,817,818,1374,1385,1386,1387,2033,2308,2776,2998,3708,5105,5140,5332,5530,5534,5563,5567,9586,10000,10891,23236,23411,55671,57223,64764,150094 | ADCY2,CALM1,CALM2,CAMK2D,CAMK2G,CPT1A,CREB1,ATF2,CREBBP,EP300,FOXO1,GNAQ,GYS2,ITPR1,PCK1,PDE3B,PLCB4,PPP3CA,PPP3R1,PRKAA2,PRKACB,CREB5,AKT3,PPARGC1A,PLCB1,SIRT1,PPP4R3A,PPP4R3B,CREB3L2,SIK1 |
| 8_Member | KEGG Pathway | hsa04540 | Gap junction | -6.138477581 | -5.152 | 26/88 | 108,1812,1956,2776,2885,2911,2915,2977,2982,3358,3708,3845,5155,5156,5332,5567,5578,5592,5594,5604,6654,6655,7082,10746,23236,56034 | ADCY2,DRD1,EGFR,GNAQ,GRB2,GRM1,GRM5,GUCY1A2,GUCY1A1,HTR2C,ITPR1,KRAS,PDGFB,PDGFRA,PLCB4,PRKACB,PRKCA,PRKG1,MAPK1,MAP2K1,SOS1,SOS2,TJP1,MAP3K2,PLCB1,PDGFC |
| 8_Member | KEGG Pathway | hsa04915 | Estrogen signaling pathway | -5.691480373 | -4.769 | 27/98 | 108,801,805,1385,1386,1956,2353,2776,2885,2911,3708,3725,3845,5291,5295,5332,5567,5594,5604,6654,6655,9568,9586,10000,23236,64764,399694 | ADCY2,CALM1,CALM2,CREB1,ATF2,EGFR,FOS,GNAQ,GRB2,GRM1,ITPR1,JUN,KRAS,PIK3CB,PIK3R1,PLCB4,PRKACB,MAPK1,MAP2K1,SOS1,SOS2,GABBR2,CREB5,AKT3,PLCB1,CREB3L2,SHC4 |
| 8_Member | KEGG Pathway | hsa04725 | Cholinergic synapse | -5.466563894 | -4.558 | 29/112 | 108,775,776,817,818,1385,2353,2534,2776,2782,3708,3717,3759,3768,3845,5291,5295,5332,5567,5578,5594,5604,9586,10000,23236,56479,59345,60482,64764 | ADCY2,CACNA1C,CACNA1D,CAMK2D,CAMK2G,CREB1,FOS,FYN,GNAQ,GNB1,ITPR1,JAK2,KCNJ2,KCNJ12,KRAS,PIK3CB,PIK3R1,PLCB4,PRKACB,PRKCA,MAPK1,MAP2K1,CREB5,AKT3,PLCB1,KCNQ5,GNB4,SLC5A7,CREB3L2 |
| 8_Member | KEGG Pathway | hsa04371 | Apelin signaling pathway | -4.870409585 | -4.021 | 32/138 | 108,182,801,805,1490,1958,2475,2776,2782,3708,3714,3845,4205,4208,4638,5140,5332,5563,5567,5581,5594,5604,6198,6546,7046,8678,10000,10672,10891,22800,23236,59345 | ADCY2,JAG1,CALM1,CALM2,CCN2,EGR1,MTOR,GNAQ,GNB1,ITPR1,JAG2,KRAS,MEF2A,MEF2C,MYLK,PDE3B,PLCB4,PRKAA2,PRKACB,PRKCE,MAPK1,MAP2K1,RPS6KB1,SLC8A1,TGFBR1,BECN1,AKT3,GNA13,PPARGC1A,RRAS2,PLCB1,GNB4 |
| 8_Member | KEGG Pathway | hsa04713 | Circadian entrainment | -4.849414503 | -4.007 | 25/96 | 108,775,776,801,805,817,818,1385,2353,2776,2782,2891,2903,2904,2977,2982,3708,5332,5567,5578,5592,5594,9252,23236,59345 | ADCY2,CACNA1C,CACNA1D,CALM1,CALM2,CAMK2D,CAMK2G,CREB1,FOS,GNAQ,GNB1,GRIA2,GRIN2A,GRIN2B,GUCY1A2,GUCY1A1,ITPR1,PLCB4,PRKACB,PRKCA,PRKG1,MAPK1,RPS6KA5,PLCB1,GNB4 |
| 8_Member | KEGG Pathway | hsa04971 | Gastric acid secretion | -4.685943718 | -3.850 | 21/75 | 60,108,481,483,801,805,817,818,1080,2776,3708,3759,3772,3776,4638,5332,5567,5578,7430,23236,54207 | ACTB,ADCY2,ATP1B1,ATP1B3,CALM1,CALM2,CAMK2D,CAMK2G,CFTR,GNAQ,ITPR1,KCNJ2,KCNJ15,KCNK2,MYLK,PLCB4,PRKACB,PRKCA,EZR,PLCB1,KCNK10 |
| 8_Member | KEGG Pathway | hsa04925 | Aldosterone synthesis and secretion | -4.560222294 | -3.736 | 22/82 | 108,747,775,776,801,805,817,818,1385,1386,2776,3708,4929,5332,5567,5578,5581,9586,23236,51305,57118,64764 | ADCY2,DAGLA,CACNA1C,CACNA1D,CALM1,CALM2,CAMK2D,CAMK2G,CREB1,ATF2,GNAQ,ITPR1,NR4A2,PLCB4,PRKACB,PRKCA,PRKCE,CREB5,PLCB1,KCNK9,CAMK1D,CREB3L2 |
| 8_Member | KEGG Pathway | hsa04730 | Long-term depression | -4.543679812 | -3.727 | 18/60 | 2776,2781,2891,2895,2911,2977,2982,3708,3845,5332,5515,5516,5578,5592,5594,5604,10672,23236 | GNAQ,GNAZ,GRIA2,GRID2,GRM1,GUCY1A2,GUCY1A1,ITPR1,KRAS,PLCB4,PPP2CA,PPP2CB,PRKCA,PRKG1,MAPK1,MAP2K1,GNA13,PLCB1 |
| 8_Member | KEGG Pathway | hsa04611 | Platelet activation | -4.191360764 | -3.406 | 28/123 | 60,108,387,1278,1281,2534,2776,2977,2982,3688,3708,4638,4659,5291,5295,5332,5501,5567,5584,5592,5594,5908,6093,9475,10000,10672,23236,83660 | ACTB,ADCY2,RHOA,COL1A2,COL3A1,FYN,GNAQ,GUCY1A2,GUCY1A1,ITGB1,ITPR1,MYLK,PPP1R12A,PIK3CB,PIK3R1,PLCB4,PPP1CC,PRKACB,PRKCI,PRKG1,MAPK1,RAP1B,ROCK1,ROCK2,AKT3,GNA13,PLCB1,TLN2 |
| 8_Member | KEGG Pathway | hsa04723 | Retrograde endocannabinoid signaling | -3.989028498 | -3.225 | 24/101 | 108,747,775,776,1268,2554,2557,2558,2565,2776,2782,2891,2911,2915,3708,5332,5567,5578,5594,5599,22999,23236,57084,59345 | ADCY2,DAGLA,CACNA1C,CACNA1D,CNR1,GABRA1,GABRA4,GABRA5,GABRG1,GNAQ,GNB1,GRIA2,GRM1,GRM5,ITPR1,PLCB4,PRKACB,PRKCA,MAPK1,MAPK8,RIMS1,PLCB1,SLC17A6,GNB4 |
| 8_Member | KEGG Pathway | hsa04916 | Melanogenesis | -3.989028498 | -3.225 | 24/101 | 108,801,805,817,818,1385,1387,1499,1857,2033,2776,3815,3845,4286,5332,5567,5578,5594,5604,6934,8321,8325,23236,64764 | ADCY2,CALM1,CALM2,CAMK2D,CAMK2G,CREB1,CREBBP,CTNNB1,DVL3,EP300,GNAQ,KIT,KRAS,MITF,PLCB4,PRKACB,PRKCA,MAPK1,MAP2K1,TCF7L2,FZD1,FZD8,PLCB1,CREB3L2 |
| 8_Member | KEGG Pathway | hsa04724 | Glutamatergic synapse | -3.951521566 | -3.192 | 26/114 | 108,775,776,2776,2782,2891,2898,2903,2904,2911,2915,2917,3708,5332,5530,5534,5567,5578,5594,9455,9456,22941,23236,54407,57084,59345 | ADCY2,CACNA1C,CACNA1D,GNAQ,GNB1,GRIA2,GRIK2,GRIN2A,GRIN2B,GRM1,GRM5,GRM7,ITPR1,PLCB4,PPP3CA,PPP3R1,PRKACB,PRKCA,MAPK1,HOMER2,HOMER1,SHANK2,PLCB1,SLC38A2,SLC17A6,GNB4 |
| 8_Member | KEGG Pathway | hsa04270 | Vascular smooth muscle contraction | -3.908625705 | -3.154 | 27/121 | 108,387,775,776,800,801,805,2776,2977,2982,3708,4638,4659,5332,5501,5567,5578,5581,5588,5592,5594,5604,6093,9475,10242,10672,23236 | ADCY2,RHOA,CACNA1C,CACNA1D,CALD1,CALM1,CALM2,GNAQ,GUCY1A2,GUCY1A1,ITPR1,MYLK,PPP1R12A,PLCB4,PPP1CC,PRKACB,PRKCA,PRKCE,PRKCQ,PRKG1,MAPK1,MAP2K1,ROCK1,ROCK2,KCNMB2,GNA13,PLCB1 |
| 8_Member | KEGG Pathway | hsa04911 | Insulin secretion | -3.819923637 | -3.071 | 21/85 | 108,481,483,775,776,817,818,1385,1386,2776,3781,5332,5567,5578,6616,9586,9699,10242,23236,27445,64764 | ADCY2,ATP1B1,ATP1B3,CACNA1C,CACNA1D,CAMK2D,CAMK2G,CREB1,ATF2,GNAQ,KCNN2,PLCB4,PRKACB,PRKCA,SNAP25,CREB5,RIMS2,KCNMB2,PLCB1,PCLO,CREB3L2 |
| 8_Member | KEGG Pathway | hsa04924 | Renin secretion | -3.517480296 | -2.790 | 17/65 | 775,776,801,805,1385,2776,2977,2982,3708,3759,5139,5140,5332,5530,5534,5567,23236 | CACNA1C,CACNA1D,CALM1,CALM2,CREB1,GNAQ,GUCY1A2,GUCY1A1,ITPR1,KCNJ2,PDE3A,PDE3B,PLCB4,PPP3CA,PPP3R1,PRKACB,PLCB1 |
| 8_Member | KEGG Pathway | hsa04020 | Calcium signaling pathway | -3.498024251 | -2.777 | 35/182 | 108,291,293,488,490,493,775,776,801,805,817,818,1812,1816,1956,2066,2185,2774,2776,2903,2911,2915,3358,3708,4638,5156,5332,5530,5534,5567,5578,6546,23236,57620,90550 | ADCY2,SLC25A4,SLC25A6,ATP2A2,ATP2B1,ATP2B4,CACNA1C,CACNA1D,CALM1,CALM2,CAMK2D,CAMK2G,DRD1,DRD5,EGFR,ERBB4,PTK2B,GNAL,GNAQ,GRIN2A,GRM1,GRM5,HTR2C,ITPR1,MYLK,PDGFRA,PLCB4,PPP3CA,PPP3R1,PRKACB,PRKCA,SLC8A1,PLCB1,STIM2,MCU |
| 8_Member | KEGG Pathway | hsa04750 | Inflammatory mediator regulation of TRP channels | -2.25357909 | -1.650 | 19/97 | 108,801,805,817,818,2776,3358,3708,4803,5291,5295,5332,5501,5567,5578,5581,5588,5599,23236 | ADCY2,CALM1,CALM2,CAMK2D,CAMK2G,GNAQ,HTR2C,ITPR1,NGF,PIK3CB,PIK3R1,PLCB4,PPP1CC,PRKACB,PRKCA,PRKCE,PRKCQ,MAPK8,PLCB1 |
| 9_Summary | KEGG Pathway | hsa04350 | TGF-beta signaling pathway | -11.30199137 | -9.722 | 33/84 | 90,92,387,650,654,655,657,659,1387,1634,1874,1875,2033,3398,4052,4086,4091,5308,5515,5516,5594,6093,6198,7027,7042,7046,7048,7050,7057,8454,57154,64750,130399 | ACVR1,ACVR2A,RHOA,BMP2,BMP6,BMP7,BMPR1A,BMPR2,CREBBP,DCN,E2F4,E2F5,EP300,ID2,LTBP1,SMAD1,SMAD6,PITX2,PPP2CA,PPP2CB,MAPK1,ROCK1,RPS6KB1,TFDP1,TGFB2,TGFBR1,TGFBR2,TGIF1,THBS1,CUL1,SMURF1,SMURF2,ACVR1C |
| 9_Member | KEGG Pathway | hsa04350 | TGF-beta signaling pathway | -11.30199137 | -9.722 | 33/84 | 90,92,387,650,654,655,657,659,1387,1634,1874,1875,2033,3398,4052,4086,4091,5308,5515,5516,5594,6093,6198,7027,7042,7046,7048,7050,7057,8454,57154,64750,130399 | ACVR1,ACVR2A,RHOA,BMP2,BMP6,BMP7,BMPR1A,BMPR2,CREBBP,DCN,E2F4,E2F5,EP300,ID2,LTBP1,SMAD1,SMAD6,PITX2,PPP2CA,PPP2CB,MAPK1,ROCK1,RPS6KB1,TFDP1,TGFB2,TGFBR1,TGFBR2,TGIF1,THBS1,CUL1,SMURF1,SMURF2,ACVR1C |
| 10_Summary | KEGG Pathway | hsa05224 | Breast cancer | -10.44149437 | -8.994 | 44/144 | 182,324,1021,1499,1857,1869,1871,1956,2252,2255,2259,2353,2475,2885,3714,3725,3815,3845,4040,4041,4851,4853,5241,5291,5295,5594,5604,5728,6198,6654,6655,6934,8202,8312,8313,8321,8325,8817,10000,23493,26281,28514,54567,399694,1119,1607,5155,5156,5170,5578,5599,5900,6009,7248,8395,8936,8976,10434,10810,23446,56034,56261,60482,160851,204962,25,27,817,818,868,1027,1398,1399,2066,2549,3084,5062,5063,6416,8440,387,801,805,998,4214,4215,4803,4915,5664,5908,6195,6196,6197,7161,7531,9252,10818,27330,108,375,2185,2534,2911,2915,2917,3643,5332,5898,9267,10672,22800,23236,55326,56895,1387,2033,2113,3082,6921,7042,54583,367,1385,2263,2308,9586,64764,604,664,894,1901,5105,5563,6502,7046,7048,7874,8660,9455,9456,23411,51701,80854,114907,2120,22849,56907,64506,132864,5469,5567,9611,1977,2261,3717,4763,29119,2122,4286,1326,1739,4773,4775,5530,5534,5588,6885,6256,5618,9021,9306,9655,862,5292,3417,5214,284,2023,2782,2869,5829,6093,7074,7852,9475,59345 | JAG1,APC,CDK6,CTNNB1,DVL3,E2F1,E2F3,EGFR,FGF7,FGF10,FGF14,FOS,MTOR,GRB2,JAG2,JUN,KIT,KRAS,LRP6,LRP5,NOTCH1,NOTCH2,PGR,PIK3CB,PIK3R1,MAPK1,MAP2K1,PTEN,RPS6KB1,SOS1,SOS2,TCF7L2,NCOA3,AXIN1,AXIN2,FZD1,FZD8,FGF18,AKT3,HEY2,FGF20,DLL1,DLL4,SHC4,CHKA,DGKB,PDGFB,PDGFRA,PDPK1,PRKCA,MAPK8,RALGDS,RHEB,TSC1,PIP5K1B,WASF1,WASL,LYPLA1,WASF3,SLC44A1,PDGFC,GPCPD1,SLC5A7,DGKH,SLC44A5,ABL1,ABL2,CAMK2D,CAMK2G,CBLB,CDKN1B,CRK,CRKL,ERBB4,GAB1,NRG1,PAK2,PAK3,MAP2K4,NCK2,RHOA,CALM1,CALM2,CDC42,MAP3K1,MAP3K3,NGF,NTRK2,PSEN2,RAP1B,RPS6KA1,RPS6KA2,RPS6KA3,TP73,YWHAE,RPS6KA5,FRS2,RPS6KA6,ADCY2,ARF1,PTK2B,FYN,GRM1,GRM5,GRM7,INSR,PLCB4,RALA,CYTH1,GNA13,RRAS2,PLCB1,AGPAT5,AGPAT4,CREBBP,EP300,ETS1,HGF,ELOC,TGFB2,EGLN1,AR,CREB1,FGFR2,FOXO1,CREB5,CREB3L2,BCL6,BNIP3,CCND2,S1PR1,PCK1,PRKAA2,SKP2,TGFBR1,TGFBR2,USP7,IRS2,HOMER2,HOMER1,SIRT1,NLK,SETD7,FBXO32,ETV6,CPEB3,SPIRE1,CPEB1,CPEB2,MED1,PRKACB,NCOR1,EIF4E,FGFR3,JAK2,NF1,CTNNA3,MECOM,MITF,MAP3K8,DLG1,NFATC2,NFATC3,PPP3CA,PPP3R1,PRKCQ,MAP3K7,RXRA,PRLR,SOCS3,SOCS6,SOCS5,RUNX1T1,PIM1,IDH1,PFKP,ANGPT1,ENO1,GNB1,GRK5,PXN,ROCK1,TIAM1,CXCR4,ROCK2,GNB4 |
| 10_Member | KEGG Pathway | hsa05224 | Breast cancer | -10.44149437 | -8.994 | 44/144 | 182,324,1021,1499,1857,1869,1871,1956,2252,2255,2259,2353,2475,2885,3714,3725,3815,3845,4040,4041,4851,4853,5241,5291,5295,5594,5604,5728,6198,6654,6655,6934,8202,8312,8313,8321,8325,8817,10000,23493,26281,28514,54567,399694 | JAG1,APC,CDK6,CTNNB1,DVL3,E2F1,E2F3,EGFR,FGF7,FGF10,FGF14,FOS,MTOR,GRB2,JAG2,JUN,KIT,KRAS,LRP6,LRP5,NOTCH1,NOTCH2,PGR,PIK3CB,PIK3R1,MAPK1,MAP2K1,PTEN,RPS6KB1,SOS1,SOS2,TCF7L2,NCOA3,AXIN1,AXIN2,FZD1,FZD8,FGF18,AKT3,HEY2,FGF20,DLL1,DLL4,SHC4 |
| 10_Member | KEGG Pathway | hsa05231 | Choline metabolism in cancer | -10.40879075 | -8.994 | 35/99 | 1119,1607,1956,2353,2475,2885,3725,3845,5155,5156,5170,5291,5295,5578,5594,5599,5604,5900,6009,6198,6654,6655,7248,8395,8936,8976,10000,10434,10810,23446,56034,56261,60482,160851,204962 | CHKA,DGKB,EGFR,FOS,MTOR,GRB2,JUN,KRAS,PDGFB,PDGFRA,PDPK1,PIK3CB,PIK3R1,PRKCA,MAPK1,MAPK8,MAP2K1,RALGDS,RHEB,RPS6KB1,SOS1,SOS2,TSC1,PIP5K1B,WASF1,WASL,AKT3,LYPLA1,WASF3,SLC44A1,PDGFC,GPCPD1,SLC5A7,DGKH,SLC44A5 |
| 10_Member | KEGG Pathway | hsa04012 | ErbB signaling pathway | -9.542790241 | -8.171 | 31/86 | 25,27,817,818,868,1027,1398,1399,1956,2066,2475,2549,2885,3084,3725,3845,5062,5063,5291,5295,5578,5594,5599,5604,6198,6416,6654,6655,8440,10000,399694 | ABL1,ABL2,CAMK2D,CAMK2G,CBLB,CDKN1B,CRK,CRKL,EGFR,ERBB4,MTOR,GAB1,GRB2,NRG1,JUN,KRAS,PAK2,PAK3,PIK3CB,PIK3R1,PRKCA,MAPK1,MAPK8,MAP2K1,RPS6KB1,MAP2K4,SOS1,SOS2,NCK2,AKT3,SHC4 |
| 10_Member | KEGG Pathway | hsa04722 | Neurotrophin signaling pathway | -9.130634235 | -7.799 | 37/119 | 25,387,801,805,817,818,998,1398,1399,2549,2885,3725,3845,4214,4215,4803,4915,5170,5291,5295,5594,5599,5604,5664,5908,6195,6196,6197,6654,6655,7161,7531,9252,10000,10818,27330,399694 | ABL1,RHOA,CALM1,CALM2,CAMK2D,CAMK2G,CDC42,CRK,CRKL,GAB1,GRB2,JUN,KRAS,MAP3K1,MAP3K3,NGF,NTRK2,PDPK1,PIK3CB,PIK3R1,MAPK1,MAPK8,MAP2K1,PSEN2,RAP1B,RPS6KA1,RPS6KA2,RPS6KA3,SOS1,SOS2,TP73,YWHAE,RPS6KA5,AKT3,FRS2,RPS6KA6,SHC4 |
| 10_Member | KEGG Pathway | hsa04072 | Phospholipase D signaling pathway | -8.55324116 | -7.257 | 41/146 | 108,375,387,1607,1956,2185,2475,2534,2549,2885,2911,2915,2917,3643,3815,3845,5155,5156,5291,5295,5332,5578,5594,5604,5898,5900,6009,6654,6655,7248,8395,9267,10000,10672,22800,23236,55326,56034,56895,160851,399694 | ADCY2,ARF1,RHOA,DGKB,EGFR,PTK2B,MTOR,FYN,GAB1,GRB2,GRM1,GRM5,GRM7,INSR,KIT,KRAS,PDGFB,PDGFRA,PIK3CB,PIK3R1,PLCB4,PRKCA,MAPK1,MAP2K1,RALA,RALGDS,RHEB,SOS1,SOS2,TSC1,PIP5K1B,CYTH1,AKT3,GNA13,RRAS2,PLCB1,AGPAT5,PDGFC,AGPAT4,DGKH,SHC4 |
| 10_Member | KEGG Pathway | hsa05211 | Renal cell carcinoma | -8.501352266 | -7.239 | 25/65 | 998,1387,1398,1399,2033,2113,2549,2885,3082,3725,3845,5062,5063,5155,5291,5295,5594,5604,5908,6654,6655,6921,7042,10000,54583 | CDC42,CREBBP,CRK,CRKL,EP300,ETS1,GAB1,GRB2,HGF,JUN,KRAS,PAK2,PAK3,PDGFB,PIK3CB,PIK3R1,MAPK1,MAP2K1,RAP1B,SOS1,SOS2,ELOC,TGFB2,AKT3,EGLN1 |
| 10_Member | KEGG Pathway | hsa05215 | Prostate cancer | -8.070537956 | -6.895 | 29/87 | 367,1027,1385,1387,1499,1869,1871,1956,2033,2263,2308,2475,2885,3845,5155,5156,5170,5291,5295,5594,5604,5728,6654,6655,6934,9586,10000,56034,64764 | AR,CDKN1B,CREB1,CREBBP,CTNNB1,E2F1,E2F3,EGFR,EP300,FGFR2,FOXO1,MTOR,GRB2,KRAS,PDGFB,PDGFRA,PDPK1,PIK3CB,PIK3R1,MAPK1,MAP2K1,PTEN,SOS1,SOS2,TCF7L2,CREB5,AKT3,PDGFC,CREB3L2 |
| 10_Member | KEGG Pathway | hsa04068 | FoxO signaling pathway | -7.764334367 | -6.639 | 37/132 | 604,664,894,1027,1387,1901,1956,2033,2308,2885,2911,3643,3845,5105,5170,5291,5295,5563,5594,5599,5604,5728,6502,6654,6655,7042,7046,7048,7874,8660,9455,9456,10000,23411,51701,80854,114907 | BCL6,BNIP3,CCND2,CDKN1B,CREBBP,S1PR1,EGFR,EP300,FOXO1,GRB2,GRM1,INSR,KRAS,PCK1,PDPK1,PIK3CB,PIK3R1,PRKAA2,MAPK1,MAPK8,MAP2K1,PTEN,SKP2,SOS1,SOS2,TGFB2,TGFBR1,TGFBR2,USP7,IRS2,HOMER2,HOMER1,AKT3,SIRT1,NLK,SETD7,FBXO32 |
| 10_Member | KEGG Pathway | hsa04320 | Dorso-ventral axis formation | -7.687225728 | -6.573 | 15/28 | 1956,2113,2120,2885,3845,4851,4853,5594,5604,6654,6655,22849,56907,64506,132864 | EGFR,ETS1,ETV6,GRB2,KRAS,NOTCH1,NOTCH2,MAPK1,MAP2K1,SOS1,SOS2,CPEB3,SPIRE1,CPEB1,CPEB2 |
| 10_Member | KEGG Pathway | hsa01522 | Endocrine resistance | -7.591441534 | -6.489 | 30/96 | 108,182,1027,1869,1871,1956,2353,2475,2885,3714,3725,3845,4851,4853,5291,5295,5469,5567,5594,5599,5604,6198,6654,6655,8202,9611,10000,28514,54567,399694 | ADCY2,JAG1,CDKN1B,E2F1,E2F3,EGFR,FOS,MTOR,GRB2,JAG2,JUN,KRAS,NOTCH1,NOTCH2,PIK3CB,PIK3R1,MED1,PRKACB,MAPK1,MAPK8,MAP2K1,RPS6KB1,SOS1,SOS2,NCOA3,NCOR1,AKT3,DLL1,DLL4,SHC4 |
| 10_Member | KEGG Pathway | hsa05214 | Glioma | -7.225833222 | -6.166 | 23/64 | 801,805,817,818,1021,1869,1871,1956,2475,2885,3845,5155,5156,5291,5295,5578,5594,5604,5728,6654,6655,10000,399694 | CALM1,CALM2,CAMK2D,CAMK2G,CDK6,E2F1,E2F3,EGFR,MTOR,GRB2,KRAS,PDGFB,PDGFRA,PIK3CB,PIK3R1,PRKCA,MAPK1,MAP2K1,PTEN,SOS1,SOS2,AKT3,SHC4 |
| 10_Member | KEGG Pathway | hsa01521 | EGFR tyrosine kinase inhibitor resistance | -7.178049685 | -6.128 | 26/79 | 1956,1977,2261,2263,2475,2549,2885,3082,3084,3717,3845,4763,5155,5156,5291,5295,5578,5594,5604,5728,6198,6654,6655,10000,56034,399694 | EGFR,EIF4E,FGFR3,FGFR2,MTOR,GAB1,GRB2,HGF,NRG1,JAK2,KRAS,NF1,PDGFB,PDGFRA,PIK3CB,PIK3R1,PRKCA,MAPK1,MAP2K1,PTEN,RPS6KB1,SOS1,SOS2,AKT3,PDGFC,SHC4 |
| 10_Member | KEGG Pathway | hsa05213 | Endometrial cancer | -5.805598376 | -4.836 | 18/50 | 324,1499,1956,2885,3845,5170,5291,5295,5594,5604,5728,6654,6655,6934,8312,8313,10000,29119 | APC,CTNNB1,EGFR,GRB2,KRAS,PDPK1,PIK3CB,PIK3R1,MAPK1,MAP2K1,PTEN,SOS1,SOS2,TCF7L2,AXIN1,AXIN2,AKT3,CTNNA3 |
| 10_Member | KEGG Pathway | hsa05220 | Chronic myeloid leukemia | -5.675010955 | -4.759 | 22/71 | 25,868,1021,1027,1398,1399,1869,1871,2122,2885,3845,5291,5295,5594,5604,6654,6655,7042,7046,7048,10000,399694 | ABL1,CBLB,CDK6,CDKN1B,CRK,CRKL,E2F1,E2F3,MECOM,GRB2,KRAS,PIK3CB,PIK3R1,MAPK1,MAP2K1,SOS1,SOS2,TGFB2,TGFBR1,TGFBR2,AKT3,SHC4 |
| 10_Member | KEGG Pathway | hsa05218 | Melanoma | -5.307591217 | -4.406 | 21/69 | 1021,1869,1871,1956,2252,2255,2259,3082,3845,4286,5155,5156,5291,5295,5594,5604,5728,8817,10000,26281,56034 | CDK6,E2F1,E2F3,EGFR,FGF7,FGF10,FGF14,HGF,KRAS,MITF,PDGFB,PDGFRA,PIK3CB,PIK3R1,MAPK1,MAP2K1,PTEN,FGF18,AKT3,FGF20,PDGFC |
| 10_Member | KEGG Pathway | hsa04660 | T cell receptor signaling pathway | -5.243367752 | -4.356 | 27/103 | 387,868,998,1326,1739,2353,2534,2885,3725,3845,4773,4775,5062,5063,5170,5291,5295,5530,5534,5588,5594,5604,6654,6655,6885,8440,10000 | RHOA,CBLB,CDC42,MAP3K8,DLG1,FOS,FYN,GRB2,JUN,KRAS,NFATC2,NFATC3,PAK2,PAK3,PDPK1,PIK3CB,PIK3R1,PPP3CA,PPP3R1,PRKCQ,MAPK1,MAP2K1,SOS1,SOS2,MAP3K7,NCK2,AKT3 |
| 10_Member | KEGG Pathway | hsa05223 | Non-small cell lung cancer | -3.631577331 | -2.892 | 16/58 | 1021,1869,1871,1956,2885,3845,5170,5291,5295,5578,5594,5604,6256,6654,6655,10000 | CDK6,E2F1,E2F3,EGFR,GRB2,KRAS,PDPK1,PIK3CB,PIK3R1,PRKCA,MAPK1,MAP2K1,RXRA,SOS1,SOS2,AKT3 |
| 10_Member | KEGG Pathway | hsa04917 | Prolactin signaling pathway | -3.590022965 | -2.855 | 18/70 | 894,2353,2885,3717,3845,5291,5295,5594,5599,5604,5618,6654,6655,9021,9306,9655,10000,399694 | CCND2,FOS,GRB2,JAK2,KRAS,PIK3CB,PIK3R1,MAPK1,MAPK8,MAP2K1,PRLR,SOS1,SOS2,SOCS3,SOCS6,SOCS5,AKT3,SHC4 |
| 10_Member | KEGG Pathway | hsa05221 | Acute myeloid leukemia | -3.381838958 | -2.670 | 15/55 | 862,2475,2885,3815,3845,5291,5292,5295,5594,5604,6198,6654,6655,6934,10000 | RUNX1T1,MTOR,GRB2,KIT,KRAS,PIK3CB,PIM1,PIK3R1,MAPK1,MAP2K1,RPS6KB1,SOS1,SOS2,TCF7L2,AKT3 |
| 10_Member | KEGG Pathway | hsa05230 | Central carbon metabolism in cancer | -2.57863198 | -1.926 | 15/65 | 1956,2261,2263,2475,3417,3815,3845,5156,5214,5291,5295,5594,5604,5728,10000 | EGFR,FGFR3,FGFR2,MTOR,IDH1,KIT,KRAS,PDGFRA,PFKP,PIK3CB,PIK3R1,MAPK1,MAP2K1,PTEN,AKT3 |
| 10_Member | KEGG Pathway | hsa04066 | HIF-1 signaling pathway | -2.399080412 | -1.762 | 20/101 | 284,817,818,1027,1387,1956,1977,2023,2033,2475,3643,5291,5295,5578,5594,5604,6198,6921,10000,54583 | ANGPT1,CAMK2D,CAMK2G,CDKN1B,CREBBP,EGFR,EIF4E,ENO1,EP300,MTOR,INSR,PIK3CB,PIK3R1,PRKCA,MAPK1,MAP2K1,RPS6KB1,ELOC,AKT3,EGLN1 |
| 10_Member | KEGG Pathway | hsa04662 | B cell receptor signaling pathway | -2.195262323 | -1.598 | 15/71 | 2353,2885,3725,3845,4773,4775,5291,5295,5530,5534,5594,5604,6654,6655,10000 | FOS,GRB2,JUN,KRAS,NFATC2,NFATC3,PIK3CB,PIK3R1,PPP3CA,PPP3R1,MAPK1,MAP2K1,SOS1,SOS2,AKT3 |
| 10_Member | KEGG Pathway | hsa04062 | Chemokine signaling pathway | -2.052922156 | -1.473 | 30/182 | 108,387,998,1398,1399,2185,2782,2869,2885,3717,3845,5291,5295,5332,5567,5594,5604,5829,5908,6093,6654,6655,7074,7852,8976,9475,10000,23236,59345,399694 | ADCY2,RHOA,CDC42,CRK,CRKL,PTK2B,GNB1,GRK5,GRB2,JAK2,KRAS,PIK3CB,PIK3R1,PLCB4,PRKACB,MAPK1,MAP2K1,PXN,RAP1B,ROCK1,SOS1,SOS2,TIAM1,CXCR4,WASL,ROCK2,AKT3,PLCB1,GNB4,SHC4 |
| 11_Summary | KEGG Pathway | hsa04919 | Thyroid hormone signaling pathway | -10.10376118 | -8.711 | 38/116 | 60,481,483,488,1387,1499,1734,2033,2308,2475,2626,3845,4851,4853,5170,5214,5291,5295,5332,5469,5567,5578,5594,5604,6009,6256,7068,8202,8841,9611,9969,10000,10231,10499,23236,23389,25942,116931 | ACTB,ATP1B1,ATP1B3,ATP2A2,CREBBP,CTNNB1,DIO2,EP300,FOXO1,MTOR,GATA4,KRAS,NOTCH1,NOTCH2,PDPK1,PFKP,PIK3CB,PIK3R1,PLCB4,MED1,PRKACB,PRKCA,MAPK1,MAP2K1,RHEB,RXRA,THRB,NCOA3,HDAC3,NCOR1,MED13,AKT3,RCAN2,NCOA2,PLCB1,MED13L,SIN3A,MED12L |
| 11_Member | KEGG Pathway | hsa04919 | Thyroid hormone signaling pathway | -10.10376118 | -8.711 | 38/116 | 60,481,483,488,1387,1499,1734,2033,2308,2475,2626,3845,4851,4853,5170,5214,5291,5295,5332,5469,5567,5578,5594,5604,6009,6256,7068,8202,8841,9611,9969,10000,10231,10499,23236,23389,25942,116931 | ACTB,ATP1B1,ATP1B3,ATP2A2,CREBBP,CTNNB1,DIO2,EP300,FOXO1,MTOR,GATA4,KRAS,NOTCH1,NOTCH2,PDPK1,PFKP,PIK3CB,PIK3R1,PLCB4,MED1,PRKACB,PRKCA,MAPK1,MAP2K1,RHEB,RXRA,THRB,NCOA3,HDAC3,NCOR1,MED13,AKT3,RCAN2,NCOA2,PLCB1,MED13L,SIN3A,MED12L |
| 12_Summary | KEGG Pathway | hsa04114 | Oocyte meiosis | -8.576233669 | -7.263 | 37/124 | 108,367,801,805,817,818,996,3708,5241,5501,5515,5516,5527,5529,5530,5534,5567,5594,5604,6195,6196,6197,7529,7531,7532,7533,7534,8454,9126,9748,10393,10971,22849,23291,27330,64506,132864,890,3845,5140,5291,5295,5599,10000 | ADCY2,AR,CALM1,CALM2,CAMK2D,CAMK2G,CDC27,ITPR1,PGR,PPP1CC,PPP2CA,PPP2CB,PPP2R5C,PPP2R5E,PPP3CA,PPP3R1,PRKACB,MAPK1,MAP2K1,RPS6KA1,RPS6KA2,RPS6KA3,YWHAB,YWHAE,YWHAG,YWHAH,YWHAZ,CUL1,SMC3,SLK,ANAPC10,YWHAQ,CPEB3,FBXW11,RPS6KA6,CPEB1,CPEB2,CCNA2,KRAS,PDE3B,PIK3CB,PIK3R1,MAPK8,AKT3 |
| 12_Member | KEGG Pathway | hsa04114 | Oocyte meiosis | -8.576233669 | -7.263 | 37/124 | 108,367,801,805,817,818,996,3708,5241,5501,5515,5516,5527,5529,5530,5534,5567,5594,5604,6195,6196,6197,7529,7531,7532,7533,7534,8454,9126,9748,10393,10971,22849,23291,27330,64506,132864 | ADCY2,AR,CALM1,CALM2,CAMK2D,CAMK2G,CDC27,ITPR1,PGR,PPP1CC,PPP2CA,PPP2CB,PPP2R5C,PPP2R5E,PPP3CA,PPP3R1,PRKACB,MAPK1,MAP2K1,RPS6KA1,RPS6KA2,RPS6KA3,YWHAB,YWHAE,YWHAG,YWHAH,YWHAZ,CUL1,SMC3,SLK,ANAPC10,YWHAQ,CPEB3,FBXW11,RPS6KA6,CPEB1,CPEB2 |
| 12_Member | KEGG Pathway | hsa04914 | Progesterone-mediated oocyte maturation | -3.056379584 | -2.363 | 21/96 | 108,890,996,3845,5140,5241,5291,5295,5567,5594,5599,5604,6195,6196,6197,10000,10393,22849,27330,64506,132864 | ADCY2,CCNA2,CDC27,KRAS,PDE3B,PGR,PIK3CB,PIK3R1,PRKACB,MAPK1,MAPK8,MAP2K1,RPS6KA1,RPS6KA2,RPS6KA3,AKT3,ANAPC10,CPEB3,RPS6KA6,CPEB1,CPEB2 |
| 13_Summary | KEGG Pathway | hsa04024 | cAMP signaling pathway | -8.523503932 | -7.245 | 50/198 | 51,108,387,481,483,488,490,493,775,776,801,805,817,818,1080,1385,1387,1812,1816,2033,2353,2737,2891,2903,2904,3725,4301,4659,5139,5140,5142,5144,5291,5295,5501,5567,5594,5599,5604,5727,5908,6093,7074,9475,9568,9586,10000,22800,64399,64764,1386,5173,5530,5534,5578,6571,23411,8851 | ACOX1,ADCY2,RHOA,ATP1B1,ATP1B3,ATP2A2,ATP2B1,ATP2B4,CACNA1C,CACNA1D,CALM1,CALM2,CAMK2D,CAMK2G,CFTR,CREB1,CREBBP,DRD1,DRD5,EP300,FOS,GLI3,GRIA2,GRIN2A,GRIN2B,JUN,AFDN,PPP1R12A,PDE3A,PDE3B,PDE4B,PDE4D,PIK3CB,PIK3R1,PPP1CC,PRKACB,MAPK1,MAPK8,MAP2K1,PTCH1,RAP1B,ROCK1,TIAM1,ROCK2,GABBR2,CREB5,AKT3,RRAS2,HHIP,CREB3L2,ATF2,PDYN,PPP3CA,PPP3R1,PRKCA,SLC18A2,SIRT1,CDK5R1 |
| 13_Member | KEGG Pathway | hsa04024 | cAMP signaling pathway | -8.523503932 | -7.245 | 50/198 | 51,108,387,481,483,488,490,493,775,776,801,805,817,818,1080,1385,1387,1812,1816,2033,2353,2737,2891,2903,2904,3725,4301,4659,5139,5140,5142,5144,5291,5295,5501,5567,5594,5599,5604,5727,5908,6093,7074,9475,9568,9586,10000,22800,64399,64764 | ACOX1,ADCY2,RHOA,ATP1B1,ATP1B3,ATP2A2,ATP2B1,ATP2B4,CACNA1C,CACNA1D,CALM1,CALM2,CAMK2D,CAMK2G,CFTR,CREB1,CREBBP,DRD1,DRD5,EP300,FOS,GLI3,GRIA2,GRIN2A,GRIN2B,JUN,AFDN,PPP1R12A,PDE3A,PDE3B,PDE4B,PDE4D,PIK3CB,PIK3R1,PPP1CC,PRKACB,MAPK1,MAPK8,MAP2K1,PTCH1,RAP1B,ROCK1,TIAM1,ROCK2,GABBR2,CREB5,AKT3,RRAS2,HHIP,CREB3L2 |
| 13_Member | KEGG Pathway | hsa05031 | Amphetamine addiction | -7.336202478 | -6.266 | 24/68 | 775,776,801,805,817,818,1385,1386,1812,2353,2891,2903,2904,3725,5173,5501,5530,5534,5567,5578,6571,9586,23411,64764 | CACNA1C,CACNA1D,CALM1,CALM2,CAMK2D,CAMK2G,CREB1,ATF2,DRD1,FOS,GRIA2,GRIN2A,GRIN2B,JUN,PDYN,PPP1CC,PPP3CA,PPP3R1,PRKACB,PRKCA,SLC18A2,CREB5,SIRT1,CREB3L2 |
| 13_Member | KEGG Pathway | hsa05030 | Cocaine addiction | -2.883481534 | -2.207 | 13/49 | 1385,1386,1812,2891,2903,2904,3725,5173,5567,6571,8851,9586,64764 | CREB1,ATF2,DRD1,GRIA2,GRIN2A,GRIN2B,JUN,PDYN,PRKACB,SLC18A2,CDK5R1,CREB5,CREB3L2 |
| 14_Summary | KEGG Pathway | hsa04530 | Tight junction | -8.425154398 | -7.179 | 45/170 | 60,387,776,998,1080,1739,1740,2017,2626,3688,3725,4214,4301,4478,4627,4628,4734,5010,5515,5516,5520,5522,5563,5567,5581,5584,5599,5962,6093,7074,7082,7430,8976,9223,9414,9475,9693,10207,23327,51762,55114,55844,64398,84612,154810 | ACTB,RHOA,CACNA1D,CDC42,CFTR,DLG1,DLG2,CTTN,GATA4,ITGB1,JUN,MAP3K1,AFDN,MSN,MYH9,MYH10,NEDD4,CLDN11,PPP2CA,PPP2CB,PPP2R2A,PPP2R2C,PRKAA2,PRKACB,PRKCE,PRKCI,MAPK8,RDX,ROCK1,TIAM1,TJP1,EZR,WASL,MAGI1,TJP2,ROCK2,RAPGEF2,PATJ,NEDD4L,RAB8B,ARHGAP17,PPP2R2D,MPP5,PARD6B,AMOTL1 |
| 14_Member | KEGG Pathway | hsa04530 | Tight junction | -8.425154398 | -7.179 | 45/170 | 60,387,776,998,1080,1739,1740,2017,2626,3688,3725,4214,4301,4478,4627,4628,4734,5010,5515,5516,5520,5522,5563,5567,5581,5584,5599,5962,6093,7074,7082,7430,8976,9223,9414,9475,9693,10207,23327,51762,55114,55844,64398,84612,154810 | ACTB,RHOA,CACNA1D,CDC42,CFTR,DLG1,DLG2,CTTN,GATA4,ITGB1,JUN,MAP3K1,AFDN,MSN,MYH9,MYH10,NEDD4,CLDN11,PPP2CA,PPP2CB,PPP2R2A,PPP2R2C,PRKAA2,PRKACB,PRKCE,PRKCI,MAPK8,RDX,ROCK1,TIAM1,TJP1,EZR,WASL,MAGI1,TJP2,ROCK2,RAPGEF2,PATJ,NEDD4L,RAB8B,ARHGAP17,PPP2R2D,MPP5,PARD6B,AMOTL1 |
| 15_Summary | KEGG Pathway | hsa04310 | Wnt signaling pathway | -8.308057209 | -7.077 | 40/143 | 324,387,817,818,894,1387,1499,1857,2033,3725,4040,4041,4773,4775,5332,5530,5534,5567,5578,5599,6477,6885,6907,6934,8312,8313,8321,8325,8454,9475,23002,23236,23291,51701,56998,79718,81839,85407,144165,166336 | APC,RHOA,CAMK2D,CAMK2G,CCND2,CREBBP,CTNNB1,DVL3,EP300,JUN,LRP6,LRP5,NFATC2,NFATC3,PLCB4,PPP3CA,PPP3R1,PRKACB,PRKCA,MAPK8,SIAH1,MAP3K7,TBL1X,TCF7L2,AXIN1,AXIN2,FZD1,FZD8,CUL1,ROCK2,DAAM1,PLCB1,FBXW11,NLK,CTNNBIP1,TBL1XR1,VANGL1,NKD1,PRICKLE1,PRICKLE2 |
| 15_Member | KEGG Pathway | hsa04310 | Wnt signaling pathway | -8.308057209 | -7.077 | 40/143 | 324,387,817,818,894,1387,1499,1857,2033,3725,4040,4041,4773,4775,5332,5530,5534,5567,5578,5599,6477,6885,6907,6934,8312,8313,8321,8325,8454,9475,23002,23236,23291,51701,56998,79718,81839,85407,144165,166336 | APC,RHOA,CAMK2D,CAMK2G,CCND2,CREBBP,CTNNB1,DVL3,EP300,JUN,LRP6,LRP5,NFATC2,NFATC3,PLCB4,PPP3CA,PPP3R1,PRKACB,PRKCA,MAPK8,SIAH1,MAP3K7,TBL1X,TCF7L2,AXIN1,AXIN2,FZD1,FZD8,CUL1,ROCK2,DAAM1,PLCB1,FBXW11,NLK,CTNNBIP1,TBL1XR1,VANGL1,NKD1,PRICKLE1,PRICKLE2 |
| 16_Summary | KEGG Pathway | hsa04520 | Adherens junction | -8.132822196 | -6.930 | 26/72 | 60,387,998,1387,1499,1956,2033,2534,3643,4008,4301,5594,5792,5797,6885,6934,7046,7048,7082,8936,8976,10458,10580,10810,29119,51701 | ACTB,RHOA,CDC42,CREBBP,CTNNB1,EGFR,EP300,FYN,INSR,LMO7,AFDN,MAPK1,PTPRF,PTPRM,MAP3K7,TCF7L2,TGFBR1,TGFBR2,TJP1,WASF1,WASL,BAIAP2,SORBS1,WASF3,CTNNA3,NLK |
| 16_Member | KEGG Pathway | hsa04520 | Adherens junction | -8.132822196 | -6.930 | 26/72 | 60,387,998,1387,1499,1956,2033,2534,3643,4008,4301,5594,5792,5797,6885,6934,7046,7048,7082,8936,8976,10458,10580,10810,29119,51701 | ACTB,RHOA,CDC42,CREBBP,CTNNB1,EGFR,EP300,FYN,INSR,LMO7,AFDN,MAPK1,PTPRF,PTPRM,MAP3K7,TCF7L2,TGFBR1,TGFBR2,TJP1,WASF1,WASL,BAIAP2,SORBS1,WASF3,CTNNA3,NLK |
| 17_Summary | KEGG Pathway | hsa04120 | Ubiquitin mediated proteolysis | -7.818666496 | -6.680 | 38/137 | 868,996,4214,4734,6477,6502,6921,7321,7322,7323,7325,7326,7328,7329,7334,7337,8065,8452,8454,8925,9021,9039,9320,9886,10054,10277,10393,10477,23291,23327,55284,55294,57154,57448,64326,64750,83737,90293 | CBLB,CDC27,MAP3K1,NEDD4,SIAH1,SKP2,ELOC,UBE2D1,UBE2D2,UBE2D3,UBE2E2,UBE2G1,UBE2H,UBE2I,UBE2N,UBE3A,CUL5,CUL3,CUL1,HERC1,SOCS3,UBA3,TRIP12,RHOBTB1,UBA2,UBE4B,ANAPC10,UBE2E3,FBXW11,NEDD4L,UBE2W,FBXW7,SMURF1,BIRC6,COP1,SMURF2,ITCH,KLHL13 |
| 17_Member | KEGG Pathway | hsa04120 | Ubiquitin mediated proteolysis | -7.818666496 | -6.680 | 38/137 | 868,996,4214,4734,6477,6502,6921,7321,7322,7323,7325,7326,7328,7329,7334,7337,8065,8452,8454,8925,9021,9039,9320,9886,10054,10277,10393,10477,23291,23327,55284,55294,57154,57448,64326,64750,83737,90293 | CBLB,CDC27,MAP3K1,NEDD4,SIAH1,SKP2,ELOC,UBE2D1,UBE2D2,UBE2D3,UBE2E2,UBE2G1,UBE2H,UBE2I,UBE2N,UBE3A,CUL5,CUL3,CUL1,HERC1,SOCS3,UBA3,TRIP12,RHOBTB1,UBA2,UBE4B,ANAPC10,UBE2E3,FBXW11,NEDD4L,UBE2W,FBXW7,SMURF1,BIRC6,COP1,SMURF2,ITCH,KLHL13 |
| 18_Summary | KEGG Pathway | hsa05166 | HTLV-I infection | -7.471143408 | -6.379 | 57/256 | 108,291,293,324,894,996,1385,1386,1387,1499,1739,1857,1869,1871,1958,2005,2033,2113,2353,3725,3845,4214,4215,4603,4773,4775,5155,5156,5291,5295,5530,5534,5567,5599,5901,6416,6722,6929,7042,7046,7048,7412,7494,7514,8295,8321,8325,8498,8829,9519,10000,10393,10714,22800,23373,83660,94241 | ADCY2,SLC25A4,SLC25A6,APC,CCND2,CDC27,CREB1,ATF2,CREBBP,CTNNB1,DLG1,DVL3,E2F1,E2F3,EGR1,ELK4,EP300,ETS1,FOS,JUN,KRAS,MAP3K1,MAP3K3,MYBL1,NFATC2,NFATC3,PDGFB,PDGFRA,PIK3CB,PIK3R1,PPP3CA,PPP3R1,PRKACB,MAPK8,RAN,MAP2K4,SRF,TCF3,TGFB2,TGFBR1,TGFBR2,VCAM1,XBP1,XPO1,TRRAP,FZD1,FZD8,RANBP3,NRP1,TBPL1,AKT3,ANAPC10,POLD3,RRAS2,CRTC1,TLN2,TP53INP1 |
| 18_Member | KEGG Pathway | hsa05166 | HTLV-I infection | -7.471143408 | -6.379 | 57/256 | 108,291,293,324,894,996,1385,1386,1387,1499,1739,1857,1869,1871,1958,2005,2033,2113,2353,3725,3845,4214,4215,4603,4773,4775,5155,5156,5291,5295,5530,5534,5567,5599,5901,6416,6722,6929,7042,7046,7048,7412,7494,7514,8295,8321,8325,8498,8829,9519,10000,10393,10714,22800,23373,83660,94241 | ADCY2,SLC25A4,SLC25A6,APC,CCND2,CDC27,CREB1,ATF2,CREBBP,CTNNB1,DLG1,DVL3,E2F1,E2F3,EGR1,ELK4,EP300,ETS1,FOS,JUN,KRAS,MAP3K1,MAP3K3,MYBL1,NFATC2,NFATC3,PDGFB,PDGFRA,PIK3CB,PIK3R1,PPP3CA,PPP3R1,PRKACB,MAPK8,RAN,MAP2K4,SRF,TCF3,TGFB2,TGFBR1,TGFBR2,VCAM1,XBP1,XPO1,TRRAP,FZD1,FZD8,RANBP3,NRP1,TBPL1,AKT3,ANAPC10,POLD3,RRAS2,CRTC1,TLN2,TP53INP1 |
| 19_Summary | KEGG Pathway | hsa05202 | Transcriptional misregulation in cancer | -7.139854791 | -6.099 | 44/180 | 604,860,862,894,905,1027,1655,1848,2005,2078,2115,2119,2120,2138,2308,2313,3206,3398,4005,4086,4094,4208,4299,4300,4613,5079,5090,6256,6760,6929,6935,7048,7403,8013,8464,9611,25942,51274,51804,55589,64083,64332,84444,221037 | BCL6,RUNX2,RUNX1T1,CCND2,CCNT2,CDKN1B,DDX5,DUSP6,ELK4,ERG,ETV1,ETV5,ETV6,EYA1,FOXO1,FLI1,HOXA10,ID2,LMO2,SMAD1,MAF,MEF2C,AFF1,MLLT3,MYCN,PAX5,PBX3,RXRA,SS18,TCF3,ZEB1,TGFBR2,KDM6A,NR4A3,SUPT3H,NCOR1,SIN3A,KLF3,SIX4,BMP2K,GOLPH3,NFKBIZ,DOT1L,JMJD1C |
| 19_Member | KEGG Pathway | hsa05202 | Transcriptional misregulation in cancer | -7.139854791 | -6.099 | 44/180 | 604,860,862,894,905,1027,1655,1848,2005,2078,2115,2119,2120,2138,2308,2313,3206,3398,4005,4086,4094,4208,4299,4300,4613,5079,5090,6256,6760,6929,6935,7048,7403,8013,8464,9611,25942,51274,51804,55589,64083,64332,84444,221037 | BCL6,RUNX2,RUNX1T1,CCND2,CCNT2,CDKN1B,DDX5,DUSP6,ELK4,ERG,ETV1,ETV5,ETV6,EYA1,FOXO1,FLI1,HOXA10,ID2,LMO2,SMAD1,MAF,MEF2C,AFF1,MLLT3,MYCN,PAX5,PBX3,RXRA,SS18,TCF3,ZEB1,TGFBR2,KDM6A,NR4A3,SUPT3H,NCOR1,SIN3A,KLF3,SIX4,BMP2K,GOLPH3,NFKBIZ,DOT1L,JMJD1C |
| 20_Summary | KEGG Pathway | hsa04550 | Signaling pathways regulating pluripotency of stem cells | -7.12089103 | -6.090 | 37/139 | 90,92,324,463,657,659,1499,1857,2261,2263,2885,3398,3717,3720,3845,3977,4086,4762,5291,5295,5594,5604,5978,6657,6929,7547,7994,8312,8313,8321,8325,9314,10000,10336,56916,84333,130399 | ACVR1,ACVR2A,APC,ZFHX3,BMPR1A,BMPR2,CTNNB1,DVL3,FGFR3,FGFR2,GRB2,ID2,JAK2,JARID2,KRAS,LIFR,SMAD1,NEUROG1,PIK3CB,PIK3R1,MAPK1,MAP2K1,REST,SOX2,TCF3,ZIC3,KAT6A,AXIN1,AXIN2,FZD1,FZD8,KLF4,AKT3,PCGF3,SMARCAD1,PCGF5,ACVR1C |
| 20_Member | KEGG Pathway | hsa04550 | Signaling pathways regulating pluripotency of stem cells | -7.12089103 | -6.090 | 37/139 | 90,92,324,463,657,659,1499,1857,2261,2263,2885,3398,3717,3720,3845,3977,4086,4762,5291,5295,5594,5604,5978,6657,6929,7547,7994,8312,8313,8321,8325,9314,10000,10336,56916,84333,130399 | ACVR1,ACVR2A,APC,ZFHX3,BMPR1A,BMPR2,CTNNB1,DVL3,FGFR3,FGFR2,GRB2,ID2,JAK2,JARID2,KRAS,LIFR,SMAD1,NEUROG1,PIK3CB,PIK3R1,MAPK1,MAP2K1,REST,SOX2,TCF3,ZIC3,KAT6A,AXIN1,AXIN2,FZD1,FZD8,KLF4,AKT3,PCGF3,SMARCAD1,PCGF5,ACVR1C |
