# Supplementary figures and images for: MiRNA Profiling in Pectoral Muscle Throughout Pre- to Post-Natal Stages of Chicken Development
Source: Front Genet. 2020 Jun 23;11:570. doi: 10.3389/fgene.2020.00570 (PMC7324647; doi:10.3389/fgene.2020.00570)

Fig S1. The count of miRNA species

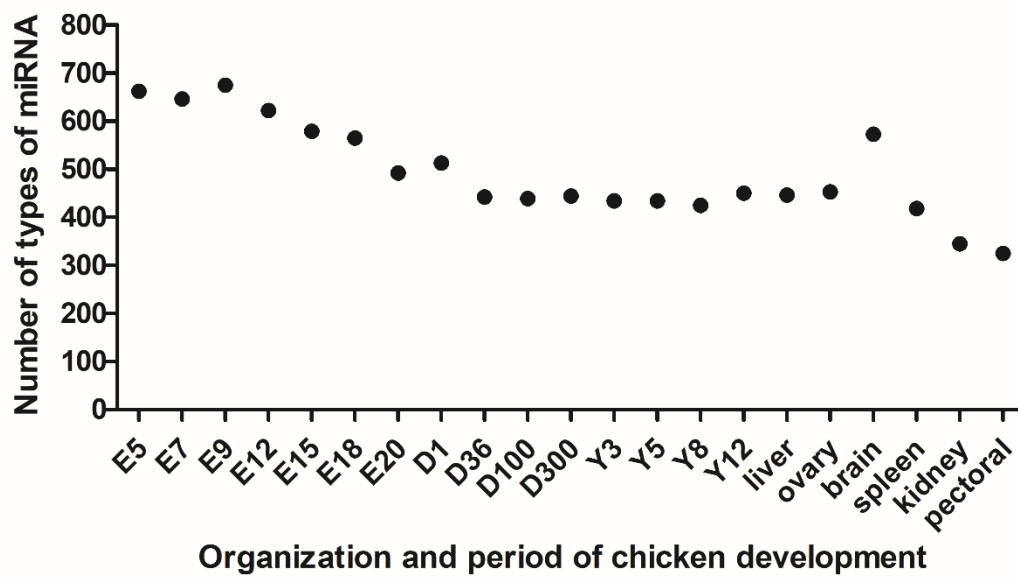

Supplement: Supplementary file 1 [file Data_Sheet_1.ZIP › Supplementary Material/Figure S1.pdf]

Fig S2. Length distribution of total clean reads

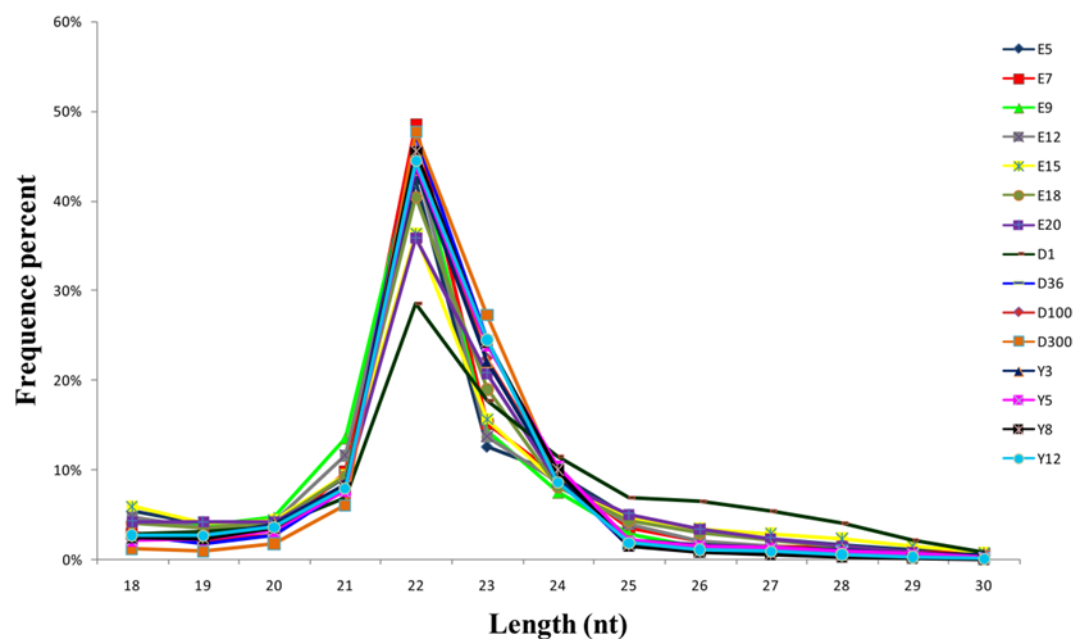

Supplement: Supplementary file 1 [file Data_Sheet_1.ZIP › Supplementary Material/Figure S2.pdf]
